# The seventh blind test of crystal structure prediction: structure ranking methods

**DOI:** 10.1107/S2052520624008679

**Published:** 2024-10-17

**Authors:** Lily M. Hunnisett, Nicholas Francia, Jonas Nyman, Nathan S. Abraham, Srinivasulu Aitipamula, Tamador Alkhidir, Mubarak Almehairbi, Andrea Anelli, Dylan M. Anstine, John E. Anthony, Joseph E. Arnold, Faezeh Bahrami, Michael A. Bellucci, Gregory J. O. Beran, Rajni M. Bhardwaj, Raffaello Bianco, Joanna A. Bis, A. Daniel Boese, James Bramley, Doris E. Braun, Patrick W. V. Butler, Joseph Cadden, Stephen Carino, Ctirad Červinka, Eric J. Chan, Chao Chang, Sarah M. Clarke, Simon J. Coles, Cameron J. Cook, Richard I. Cooper, Tom Darden, Graeme M. Day, Wenda Deng, Hanno Dietrich, Antonio DiPasquale, Bhausaheb Dhokale, Bouke P. van Eijck, Mark R. J. Elsegood, Dzmitry Firaha, Wenbo Fu, Kaori Fukuzawa, Nikolaos Galanakis, Hitoshi Goto, Chandler Greenwell, Rui Guo, Jürgen Harter, Julian Helfferich, Johannes Hoja, John Hone, Richard Hong, Michal Hušák, Yasuhiro Ikabata, Olexandr Isayev, Ommair Ishaque, Varsha Jain, Yingdi Jin, Aling Jing, Erin R. Johnson, Ian Jones, K. V. Jovan Jose, Elena A. Kabova, Adam Keates, Paul F. Kelly, Jiří Klimeš, Veronika Kostková, He Li, Xiaolu Lin, Alexander List, Congcong Liu, Yifei Michelle Liu, Zenghui Liu, Ivor Lončarić, Joseph W. Lubach, Jan Ludík, Noa Marom, Hiroyuki Matsui, Alessandra Mattei, R. Alex Mayo, John W. Melkumov, Bruno Mladineo, Sharmarke Mohamed, Zahrasadat Momenzadeh Abardeh, Hari S. Muddana, Naofumi Nakayama, Kamal Singh Nayal, Marcus A. Neumann, Rahul Nikhar, Shigeaki Obata, Dana O’Connor, Artem R. Oganov, Koji Okuwaki, Alberto Otero-de-la-Roza, Sean Parkin, Antonio Parunov, Rafał Podeszwa, Alastair J. A. Price, Louise S. Price, Sarah L. Price, Michael R. Probert, Angeles Pulido, Gunjan Rajendra Ramteke, Atta Ur Rehman, Susan M. Reutzel-Edens, Jutta Rogal, Marta J. Ross, Adrian F. Rumson, Ghazala Sadiq, Zeinab M. Saeed, Alireza Salimi, Kiran Sasikumar, Sivakumar Sekharan, Kenneth Shankland, Baimei Shi, Xuekun Shi, Kotaro Shinohara, A. Geoffrey Skillman, Hongxing Song, Nina Strasser, Jacco van de Streek, Isaac J. Sugden, Guangxu Sun, Krzysztof Szalewicz, Lu Tan, Kehan Tang, Frank Tarczynski, Christopher R. Taylor, Alexandre Tkatchenko, Rithwik Tom, Petr Touš, Mark E. Tuckerman, Pablo A. Unzueta, Yohei Utsumi, Leslie Vogt-Maranto, Jake Weatherston, Luke J. Wilkinson, Robert D. Willacy, Lukasz Wojtas, Grahame R. Woollam, Yi Yang, Zhuocen Yang, Etsuo Yonemochi, Xin Yue, Qun Zeng, Tian Zhou, Yunfei Zhou, Roman Zubatyuk, Jason C. Cole

**Affiliations:** a The Cambridge Crystallographic Data Centre 12 Union Road Cambridge CB2 1EZ UK; b AbbVie Inc., Research & Development, 1 N Waukegan Road, North Chicago, IL 60064, USA; c Crystallization and Particle Sciences Institute of Chemical and Engineering Sciences 1 Pesek Road Singapore 627833 Singapore; d Green Chemistry and Materials Modelling Laboratory Khalifa University of Science and Technology PO Box 127788 Abu Dhabi United Arab Emirates; e Roche Pharma Research and Early Development Therapeutic Modalities Roche Innovation Center Basel F Hoffmann-La Roche Ltd Grenzacherstrasse 124 4070 Basel Switzerland; f Department of Chemistry, Carnegie Mellon University, 4400 Fifth Avenue, Pittsburgh, PA 15213, USA; g Department of Chemistry University of Kentucky Lexington KY 40506 USA; h School of Chemistry University of Southampton Southampton SO17 1BJ UK; i Department of Chemistry Faculty of Science Ferdowsi University of Mashhad Mashhad Iran; j XtalPi Inc, 245 Main Street, Cambridge, MA 02142, USA; k Department of Chemistry University of California Riverside CA 92521 USA; l Ruđer Bošković Institute, Bijenička cesta 54, Zagreb, Croatia; m Catalent Pharma Solutions 160 Pharma Drive Morrisville NC 27560 USA; n Department of Chemistry University of Graz Heinrichstrasse 28 Graz Austria; o University of Innsbruck Institute of Pharmacy Innrain 52c A-6020 Innsbruck Austria; p Department of Physical Chemistry University of Chemistry and Technology Technická 5 16628 Prague Czech Republic; q Department of Chemistry New York University New York NY 10003 USA; r XtalPi Inc., International Biomedical Innovation Park II 3F 2 Hongliu Road, Futian District, Shenzhen, Guangdong, China; s Department of Chemistry Dalhousie University 6274 Coburg Road Dalhousie Halifax Canada; t Department of Chemistry University of Oxford 12 Mansfield Road Oxford OX1 3TA UK; u OpenEye Scientific Software, 9 Bisbee Court, Santa Fe, NM 87508, USA; v Department of Materials Science and Engineering, Carnegie Mellon University, 5000 Forbes Avenue, Pittsburgh, PA 15213, USA; w Avant-garde Materials Simulation, Alte Strasse 2, 79249 Merzhausen, Germany; x Genentech, Inc., 1 DNA Way, South San Francisco, CA 94080, USA; y Department of Chemistry University of Wyoming Laramie Wyoming 82071 USA; z University of Utrecht (Retired), Department of Crystal and Structural Chemistry, Padualaan 8, 3584 CH Utrecht, The Netherlands; aa Chemistry Department Loughborough University Loughborough LE11 3TU UK; bb Graduate School of Pharmaceutical Sciences Osaka University 1-6 Yamadaoka Suita Osaka 656-0871 Japan; cc School of Pharmacy and Pharmaceutical Sciences Hoshi University 2-4-41 Ebara Shinagawa-ku Tokyo 142-8501 Japan; dd Information and Media Center Toyohashi University of Technology 1-1 Hibarigaoka Tempaku-cho Toyohashi Aichi 441-8580 Japan; ee CONFLEX Corporation, Shinagawa Center building 6F, 3-23-17 Takanawa, Minato-ku, Tokyo 108-0074, Japan; ff Department of Chemistry University College London 20 Gordon Street London WC1H 0AJ UK; gg Syngenta Ltd., Jealott’s Hill International Research Station, Berkshire, RG42 6EY, UK; hh Department of Solid State Chemistry University of Chemistry and Technology Technická 5 16628 Prague Czech Republic; ii Department of Physics and Astronomy University of Delaware Newark DE 19716 USA; jj School of Chemistry University of Hyderabad Professor CR Rao Road Gachibowli Hyderabad 500046 Telangana India; kk School of Pharmacy University of Reading Whiteknights Reading RG6 6AD UK; ll Department of Chemical Physics and Optics Faculty of Mathematics and Physics Charles University Ke Karlovu 3 121 16 Prague Czech Republic; mm Skolkovo Institute of Science and Technology Bolshoy Boulevard 30 121205 Moscow Russia; nn Department of Physics, Carnegie Mellon University, 5000 Forbes Avenue, Pittsburgh, PA 15213, USA; oo Graduate School of Organic Materials Science Yamagata University 4-3-16 Jonan Yonezawa 992-8510 Yamagata Japan; pp Center for Catalysis and Separations Khalifa University of Science and Technology PO Box 127788 Abu Dhabi United Arab Emirates; qq Department of Analytical and Physical Chemistry Faculty of Chemistry University of Oviedo Julián Clavería 8 33006 Oviedo Spain; rr Institute of Chemistry University of Silesia in Katowice Szkolna 9 40-006 Katowice Poland; ss School of Natural and Environmental Sciences Newcastle University Kings Road Newcastle NE1 7RU UK; tt SuRE Pharma Consulting, LLC, 7163 Whitestown Parkway - Suite 305, Zionsville, IN 46077, USA; uu Fachbereich Physik, Freie Universität, Berlin, 14195, Germany; vv Department of Physics and Materials Science University of Luxembourg 1511 Luxembourg City Luxembourg; ww Courant Institute of Mathematical Sciences New York University New York NY 10012 USA; xx NYU-ECNU Center for Computational Chemistry at NYU Shanghai, 3663 Zhongshan Road North, Shanghai 200062, China; yy Department of Chemistry University of South Florida USF Research Park 3720 Spectrum Blvd IDRB 202 Tampa FL 33612 USA; zz Novartis Pharma AG, Basel 4002, Switzerland; CSIR–National Chemical Laboratory, India

**Keywords:** crystal structure prediction, polymorphism, lattice energy, Cambridge Structural Database, blind test

## Abstract

A seventh blind test of crystal structure prediction has been organized by the Cambridge Crystallographic Data Centre. The results are presented in two parts, with this second part focusing on methods for ranking crystal structures in order of stability. The exercise involved standardized sets of structures seeded from a range of structure generation methods. Participants from 22 groups applied several periodic DFT-D methods, machine learned potentials, force fields derived from empirical data or quantum chemical calculations, and various combinations of the above. In addition, one non-energy-based scoring function was used. Results showed that periodic DFT-D methods overall agreed with experimental data within expected error margins, while one machine learned model, applying system-specific AIMnet potentials, agreed with experiment in many cases demonstrating promise as an efficient alternative to DFT-based methods. For target XXXII, a consensus was reached across periodic DFT methods, with consistently high predicted energies of experimental forms relative to the global minimum (above 4 kJ mol^−1^ at both low and ambient temperatures) suggesting a more stable polymorph is likely not yet observed. The calculation of free energies at ambient temperatures offered improvement of predictions only in some cases (for targets XXVII and XXXI). Several avenues for future research have been suggested, highlighting the need for greater efficiency considering the vast amounts of resources utilized in many cases.

## Abstract 2

The results of the seventh blind test of crystal structure prediction are presented, focusing on structure ranking methods.

## 1. Introduction

### 1.1. Background

The Cambridge Crystallographic Data Centre (CCDC) has been organizing a set of blind tests to assess the predictive ability of existing methods for molecular crystal structure prediction (CSP), and to stimulate the development of novel approaches. The results of the seventh blind test are reported in two articles. Part one (Hunnisett *et al.*, 2024 ▸) focuses on structure generation, while part two (this article) describes the final ranking of crystal structures.

CSP aims to predict the crystal structure of any given compound using computer simulations. CSP techniques have gained much attention due to its potential applications in fields such as pharmaceuticals, materials science, and solid-state chemistry, where a thermodynamically stable material is normally sought. A key challenge in CSP is therefore the accurate ranking of predicted crystal structures by their relative stabilities (free energies). A CSP study is often gauged by the successful prediction of the thermodynamically stable crystal structure. Ranking methods are also often employed as a standalone method for assessing relative stabilities when multiple forms are obtained from experiment.

The accurate determination of the most stable polymorph is crucial in many applications. For example, in the pharmaceutical industry, the solubility and bioavailability of a drug can be significantly affected by its crystal structure (Bauer *et al.*, 2001 ▸). Predicting the most stable polymorph can guide experimental efforts to optimize drug formulation, manufacturing, and storage. Conversely, in cases where a metastable form is chosen or the stable form has not crystallized, an accurate energy ranking can be used to assess the risk that a late appearing, more stable form poses to the performance (bioavailability, shelf life) of the drug product. In materials science, the properties of a material, such as its electronic, optical, or mechanical behaviour, can be strongly influenced by its crystal structure. Therefore, accurate CSP methods can inform the design of materials with tailored properties for various applications (Tom *et al.*, 2023 ▸).

Recently, the CCDC conducted the seventh CSP blind test, providing a valuable opportunity to review and benchmark the performance of current crystal structure energy ranking methods. In this article, we present a detailed analysis of the results of the seventh blind test, focusing on understanding the strengths and weaknesses of various stability ranking techniques.

This report includes three distinct supplementary information (SI) sections. SI-A offers more information, tables, and figures on the analysis of the generated sets of structures and the preparation of structure lists. In SI-B, participating groups define their approach and possibly provide additional analysis of their landscape and results. Finally, SI-C contains the theoretically generated structures (and metadata) from each group, and any experimental structures that are not available through the CSD in the Crystallographic Information File (CIF) file format. Detailed experimental reports are provided in the supplementary information attached to phase one of this study (Hunnisett *et al.*, 2024 ▸).

### 1.2. Previous blind tests of CSP

Here, we provide a brief summary of the first six CSP blind tests, focusing on the ranking methods, showing how the methods have evolved over the years and highlighting important developments. Computational methods are often referred to by acronyms, we have therefore included a dictionary of abbreviations at the end of this paper to aid the reader.

The first blind test in 1999 (Lommerse *et al.*, 2000 ▸) featured primarily various empirical force fields), and force fields where the electrostatic model was parameterized to electronic structure calculations on the isolated molecule using Hartree–Fock or second-order Møller–Plesset perturbation theory (MP2) charge densities. Both atomic point charges and multipoles were used for the electrostatics. Besides force fields, statistical fitness functions based on probability distributions derived from the Cambridge Structural Database (CSD) were also used (Apostolakis *et al.*, 2001 ▸), demonstrating that the scoring function used to assess predicted crystal structures is not necessarily a direct estimate of the structures’ energy or thermodynamic stability.

The second blind test (Motherwell *et al.*, 2002 ▸) featured a wide range of force fields that were used to calculate lattice energies, and in one case the lattice vibrational contribution to the free energy, *F*_vib_. A couple of participants used statistical fitness functions. Since the participants were only allowed to submit three crystal structures per target compound in the first five blind tests, more subjective assessments occasionally affected the selection of the final candidates. Besides the lattice energy, the predicted morphology, density, elastic constants and ‘chemical intuition’ were used to influence the selection in some cases.

The third blind test saw participation from 18 groups, most of which used energy-based methods to rank their predicted structures (Day *et al.*, 2005*a* ▸). Several potentials were more sophisticated than generic off-the-shelf force fields, featuring anisotropic repulsion for halogen atoms and distributed multipoles (Stone, 1981 ▸; Stone & Price, 1988 ▸; Coombes *et al.*, 1996 ▸; Day & Price, 2003 ▸; Day *et al.*, 2005*b* ▸). Angelo Gavezzotti used his PIXEL method, which calculates intermolecular interaction energies by direct numerical integration over electron densities (Gavezzotti, 2002 ▸, 2005 ▸). Detlef Hofmann, similarly to the first and second blind tests, used a non-energy based statistical potential trained on experimental structures in the CSD (Hofmann & Apostolakis, 2003 ▸).

The fourth blind test (Day *et al.*, 2009 ▸) featured the first use of periodic dispersion-corrected density functional theory (DFT-D), which has since become very common. Marcus A. Neumann, Frank J. J. Leusen and John Kendrick used periodic PBE calculations supplemented with an empirically parameterized atom–atom *C*_6_ dispersion correction for their final energy minimization to successfully predict all four structures as the global minimum on the potential energy surface (Perdew *et al.*, 1996*a* ▸; Neumann & Perrin, 2005 ▸; Neumann *et al.*, 2008 ▸). The more sophisticated methodology used for the final lattice energy minimization produced far better results than the general purpose force fields with isotropic atom–atom interaction and atomic point charges (Day *et al.*, 2009 ▸). It was, however, also noted that this method was by far the most computationally demanding of the methods used for the final optimization and it could only be applied to a limited number of crystal structures for each molecule. This DFT-D method was further validated by retrospectively applying it to structures from the first three blind tests, demonstrating that it ranked eight out of ten target crystal structures as the global minimum and, in general, also reproduced the experimentally observed geometry more accurately than other methods (Asmadi *et al.*, 2009 ▸).

The fifth blind test saw a wider adoption of quantum chemical methods, periodic DFT-D in particular, following the impressive performance in the previous blind test (Bardwell *et al.*, 2011 ▸). It was also the first blind test that featured a large, flexible drug-like molecule, which catalysed the adoption of CSP methods in the pharmaceutical industry (Nyman & Reutzel-Edens, 2018 ▸).

The sixth blind test involved five target compounds, including challenging flexible molecules and a multi-component crystal, and participants were allowed to submit two sets of structures for each target compound, ranked with different methods (Reilly *et al.*, 2016 ▸). This seems to have encouraged experimentation and most groups submitted structures ranked with methods not used in previous blind tests. Various DFT-D electronic structure calculations were used on the crystals, molecules and multimers. There were pure periodic electronic structure methods (periodic PBE with a variety of dispersion corrections), mixed quantum chemical plus force field methods (Ψ_mol_), and potentials fitted to results of symmetry adapted perturbation theory (SAPT) (Misquitta *et al.*, 2005 ▸). Several groups took the opportunity to innovate corrections to the lattice energy, accounting for lattice vibrations (van Eijck, 2001 ▸; Nyman & Day, 2015 ▸), polarization (Welch *et al.*, 2008 ▸; Mennucci *et al.*, 2002 ▸), and even nucleation using kinetic Monte Carlo simulations to determine critical-nucleus sizes (Boerrigter *et al.*, 2004 ▸; Deij *et al.*, 2007 ▸). One method based on Monte Carlo parallel tempering for structure generation and final ranking by periodic DFT-D successfully predicted all of the experimental target structures (Reilly *et al.*, 2016 ▸; Kendrick *et al.*, 2011 ▸).

### 1.3. Contributions to energy rankings

#### 1.3.1. The Gibbs free energy

The relative thermodynamic stability between two polymorphs can be calculated as the difference in Gibbs free energy. Effects due to thermal expansion are often neglected, and most CSP practitioners use some variant of these generalized expressions: 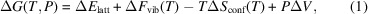
where the difference in vibrational energy between structures is 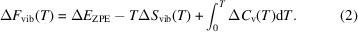
Here, *E*_latt_ is the lattice energy, *E*_ZPE_ the vibrational zero point energy, *C*_v_ the heat capacity, *S*_vib_ and *S*_conf_ the vibrational and configurational entropies, respectively, and *V* the specific volume. We discuss the intricacies of calculating some of these contributions in some detail below.

#### 1.3.2. The lattice energy

Calculating the relative stabilities of the many hypothetical structures generated in a CSP investigation is difficult for several reasons. Firstly, experimental evidence and computational investigations have shown that (free) energy differences between alternative crystal structures of the same compound are small, typically 1–2 kJ mol^−1^, and almost always less than 8 kJ mol^−1^ (Yu, 1995 ▸; Gavezzotti & Filippini, 1995 ▸; Yu *et al.*, 2005 ▸; Nyman & Day, 2015 ▸; Cruz-Cabeza *et al.*, 2015 ▸). Secondly, the thermodynamic stability at realistic temperatures depends on several contributing factors such as intermolecular interactions, conformational energy, lattice vibrational energy, and other subtle effects like the morphology of polar crystals (van Eijck & Kroon, 1997 ▸), thermal expansion and the entropic contribution from crystallographic disorder (Heit & Beran, 2016 ▸; Nyman *et al.*, 2016 ▸; Woollam *et al.*, 2018 ▸; O’Connor *et al.*, 2022 ▸; Pokorný *et al.*, 2022 ▸; Tous & Červinka, 2023 ▸).

The static cohesive energy between the crystal’s constituents, the ‘lattice energy’, is often the largest and most important contribution. There are a vast number of energy methods that can be used to calculate it. General purpose force fields like GAFF, COMPASS and OPLS are quick and computationally the most affordable (Wang *et al.*, 2004 ▸; Sun, 1998 ▸; Jorgensen *et al.*, 1996 ▸). To improve the reliability of a force field, it may be re-parameterized or tailored to the compound(s) of interest (Neumann, 2008 ▸; Metz *et al.*, 2016 ▸; Zhang *et al.*, 2018 ▸; Mattei *et al.*, 2022 ▸; Nikhar & Szalewicz, 2022 ▸).

Density functional tight binding (DFTB) approximations may be used as an intermediate energy model during CSP. The computational cost lies between force fields and DFT-D. Empirical corrections to improve the modelling of hydrogen bonding interactions, dispersion, and halogen bonds in DFTB are generally recommended (Brandenburg & Grimme, 2014*a* ▸; Řezáč, 2017 ▸; Iuzzolino *et al.*, 2018 ▸; Mortazavi *et al.*, 2018 ▸; Řezáč, 2019 ▸; Bannwarth *et al.*, 2019 ▸).

In the more recent blind tests, energy rankings based on dispersion-corrected generalized gradient approximations (GGAs) to the exchange-correlation functional of DFT demonstrated a remarkably consistent high predictive ability (Neumann & Perrin, 2005 ▸; Grimme, 2006 ▸; Day *et al.*, 2009 ▸; Bardwell *et al.*, 2011 ▸). The PBE (Perdew *et al.*, 1996*a* ▸) and B86bPBE (Becke, 1986 ▸) exchange-correlation functionals are the most commonly used for molecular crystals and are widely considered transferable and reliable, reaching a tolerable error in relative lattice energies of about 3 kJ mol^−1^ (Moellmann & Grimme, 2014 ▸; Abramov *et al.*, 2021 ▸; Firaha *et al.*, 2023 ▸) for most electrically neutral species when care is taken to properly converge the calculation and use a *k*-point sampling that compensates for the different unit cell sizes of CSP structures. Reliable dispersion corrections include the variations of Grimme’s D3 and D4 methods, many body dispersion (MBD) and the exchange hole dipole model (XDM) (Grimme *et al.*, 2010 ▸; Grimme *et al.*, 2011 ▸; Tkatchenko *et al.*, 2012 ▸; Caldeweyher *et al.*, 2020 ▸; Otero-de-la-Roza & Johnson, 2013 ▸; Reilly & Tkatchenko, 2015 ▸; Whittleton *et al.*, 2016 ▸; Price *et al.*, 2023*b* ▸).

There are also non-local density functionals that include dispersion contributions in the functional itself (Vydrov & van Voorhis, 2010 ▸; Schröder *et al.*, 2017 ▸; Chakraborty *et al.*, 2020 ▸), rather than as a correction applied after the convergence of the charge density. Such functionals have not been used in previous blind tests, but one such functional, optPBE-vdW (Klimeš *et al.*, 2009 ▸), features here for the first time.

Improved accuracy in the electronic density and energy may be achieved with density functionals, known as meta-GGA functionals, that account for the second derivative of the charge density, or the kinetic energy density, in addition to the density and its gradient. Popular variants include TPSS and SCAN, and numerically stable variants thereof (Tao *et al.*, 2003 ▸; Sun *et al.*, 2015 ▸; Bartók & Yates, 2019 ▸; Mejía-Rodríguez & Trickey, 2019 ▸; Ehlert *et al.*, 2021 ▸; Brandenburg *et al.*, 2016 ▸).

Computationally efficient local and semi-local exchange-correlation functionals, including GGA and meta-GGA functionals, suffer from self-interaction errors (SIE), the spurious Coulomb repulsion of an electron from its own density (Perdew & Zunger, 1981 ▸), which can cause large unpredictable problems in certain cases (LeBlanc *et al.*, 2018 ▸; Nyman *et al.*, 2019 ▸; Greenwell & Beran, 2020 ▸; Beran *et al.*, 2022 ▸; O’Connor *et al.*, 2023 ▸). SIE can be mitigated by hybrid functionals, such as the PBE-based hybrid PBE0, which include a fraction of Hartree–Fock exchange (Becke, 1993 ▸; Perdew *et al.*, 1996*b* ▸; Adamo & Barone, 1999 ▸; Reilly & Tkatchenko, 2013 ▸). This reduces their tendency to exaggerate electron delocalization (Cohen *et al.*, 2012 ▸). However, owing to the non-locality of the exact exchange, the computational cost of hybrid functionals is higher than that of (semi-)local functionals by an order of magnitude, making them impractical for ranking a large number of putative crystal structures. Other methods for improving the accuracy of GGA DFT-D include the application of a monomer correction, based on, for instance, MP2 (Greenwell *et al.*, 2022 ▸), or using density corrected DFT (Rana *et al.*, 2022 ▸). In addition to monomer corrections, dimer and multimer corrections have also been used in order to approximate hybrid functionals (Loboda *et al.*, 2018 ▸; Hoja *et al.*, 2023 ▸).

There are also mixed energy models that combine molecular *ab initio* calculations with an intermolecular force field, referred to as Ψ_mol_ (Price *et al.*, 2010 ▸; Kazantsev *et al.*, 2010 ▸; Wen & Beran, 2011 ▸; Williams, 2001*b* ▸; Williams, 2001*a* ▸; Pyzer-Knapp *et al.*, 2016 ▸). The molecular wavefunction can be used to obtain distributed multipoles, greatly improving the modelling of intermolecular electrostatic interactions (Stone, 1981 ▸; Coombes *et al.*, 1996 ▸; Mooij & Leusen, 2001 ▸; Day *et al.*, 2005*b* ▸). Such models have featured prominently throughout the blind tests, and have produced several successful predictions (Kazantsev *et al.*, 2011 ▸).

To refine the lattice energies, high-level *ab initio* calculations, including CCSD(T), on molecular clusters can also be coupled with lower-level QM methods, such as periodic DFT (Pokorný *et al.*, 2022 ▸) or HF (Červinka & Beran, 2018 ▸), yielding efficient QM:QM fragmentation frameworks for molecular crystals (Herbert, 2019 ▸).

Machine learned potentials have recently gained considerable attention since a carefully trained model should be able to achieve DFT-D level accuracy and be orders of magnitude faster (Musil *et al.*, 2018 ▸). A previously limiting factor for why machine learned potentials have not been suitable for crystal structure prediction was their lack of long-range interaction components. The models included only strictly local physics and chemistry, representing the crystalline environment by SOAP kernels and similar methods (Bartók *et al.*, 2013 ▸). In recent years, a number of methods have emerged for the treatment of long-range dispersion, polarization, and electrostatic interactions in machine learning (Anstine & Isayev, 2023 ▸; Grisafi & Ceriotti, 2019 ▸; Ko *et al.*, 2021 ▸; Yue *et al.*, 2021 ▸; Zhang *et al.*, 2022 ▸; Phuc Tu *et al.*, 2023 ▸).

The 2018 Faraday Discussion on crystal structure prediction featured an insightful session on energy ranking methods, covering many of the aspects touched upon here in greater detail (Addicoat *et al.*, 2018 ▸), as well as several benchmarking studies, often using the X23 benchmark set (Otero-de-la-Roza & Johnson, 2012 ▸; Reilly & Tkatchenko, 2013 ▸; Reilly & Tkatchenko, 2015 ▸; Cutini *et al.*, 2016 ▸; Hoja *et al.*, 2017 ▸; Hoja & Tkatchenko, 2018 ▸; Loboda *et al.*, 2018 ▸; Hoja *et al.*, 2019 ▸). An alternative benchmark set focusing on energetic materials has since been proposed (O’Connor *et al.*, 2022 ▸), which highlighted the need for further method development for applications in this field. The need for further benchmark data, highlighted during the discussions, has since resulted in the ongoing BEST-CSP COST action^1^. Accuracy of first-principles methods and their potential for reliable polymorph ranking at finite temperatures can be consistently benchmarked against critically assessed sublimation enthalpies or pressures for organic molecular materials (Červinka & Fulem, 2017 ▸; Červinka & Fulem, 2018 ▸). Experimental state-of-the-art in this field enables one to reach an uncertainty of the sublimation enthalpy around 0.5 kJ mol^−1^ for volatile organic materials (Fulem *et al.*, 2014 ▸) and below 4 kJ mol^−1^ for extremely low-volatile materials (Červinka *et al.*, 2019 ▸), allowing the identification of computational methods which provide uncertainties of the predicted enthalpic data well within the chemical accuracy threshold.

#### 1.3.3. Geometry optimization

Geometry optimizations by minimizing the lattice energy are generally performed with one of the Broyden-Fletcher-Goldfarb-Shanno quasi-Newton algorithms (Broyden, 1967 ▸; Head & Zerner, 1985 ▸; Liu & Nocedal, 1989 ▸), but these can converge to saddle points or arbitrarily shallow minima, leading to the prediction of crystal structures that cannot be observed experimentally (Price, 2013 ▸). The FIRE optimization algorithm, implemented in the Atomic Simulation Environment (ASE), passes over stationary points and shallow minima and often finds deeper energy minima faster than quasi-Newton methods (Bitzek *et al.*, 2006 ▸; Larsen *et al.*, 2017 ▸). Since the computational cost of geometry optimizations with quantum chemical methods is substantial, it is important to use efficient algorithms. Techniques such as preconditioners (Packwood *et al.*, 2016 ▸) and using internal coordinates (Bučko *et al.*, 2005 ▸) may speed up the calculations. Force fields, machine-learned potentials or semi-empirical methods such as DFTB can be employed to pre-optimize structures to drastically speed up geometry optimizations.

#### 1.3.4. Thermal effects

In efforts to improve upon static lattice energies, many groups include effects due to temperature in their most accurate rankings. By free energy calculations, we mean methods that explicitly calculate a thermodynamic ensemble of some kind. That can be an ensemble of microstates from a Monte Carlo or Molecular Dynamics simulation, an ensemble of configurations in a disordered crystal, or an ensemble of phonons from lattice dynamics.

Lattice vibrational contributions to the stability are commonly calculated in the harmonic approximation, leading to a temperature-dependent Helmholtz vibrational free energy (Fultz, 2010 ▸; Day *et al.*, 2003 ▸). For such calculations, it is important to consider phonon dispersion by sampling several *k*-points in the Brillouin zone, or modelling the dispersion by some other means (Gilat & Alder, 1976 ▸; Nyman *et al.*, 2016 ▸; Kamencek *et al.*, 2020 ▸). The harmonic approximation neglects thermal expansion, and although it is a small contribution, it can be significant for the accurate ranking of CSP structures, or for high accuracy calculations of temperature-dependent properties of molecular crystals (Heit & Beran, 2016 ▸; Heit *et al.*, 2016 ▸; O’Connor *et al.*, 2022 ▸).

Thermal expansion can be split into a finite temperature contribution and a zero-temperature contribution due to the atoms’ zero-point motion. The latter has been found to amount to 2% on average for the X23 set of molecular crystals (Dolgonos *et al.*, 2019 ▸). The quasi-harmonic approximation is a convenient way to model anisotropic thermal expansion (Nyman *et al.*, 2016 ▸; O’Connor *et al.*, 2022 ▸), but still fails to capture the true anharmonicity of the atomic vibrations. The latter may be modelled by molecular dynamics (Gray *et al.*, 2004 ▸; Rossi *et al.*, 2016 ▸).

An alternative way to calculate polymorph free energy differences is the Einstein crystal method (Frenkel & Ladd, 1984 ▸; Frenkel & Smit, 2001 ▸). The method calculates the relative free energy difference between two crystals by thermodynamic integration over the path to a common ideal reference state, for which the free energy can be calculated analytically. This reference state is an Einstein crystal, which consists of a set of non-interacting atoms tethered to their positions by harmonic restraints (Yang *et al.*, 2020 ▸).

#### 1.3.5. Disorder

With improvements in laboratory diffraction hardware and greater access to high energy synchrotron facilities, it is increasingly common to see disorder in experimentally determined structures, and disorder was an important factor in this blind test challenge, as four of the target crystal structures were disordered (XXVII, XXX, XXXI, XXXII).

It is also common to find clusters of similar crystal structures in CSP landscapes, which have the same overall packing except for some minor conformational change (Braun *et al.*, 2019 ▸). In many cases, such clusters of lattice energy minima correspond to a single disordered structure. In this blind test, two groups (20, 24) correctly predicted the occurrence of disorder in a target crystal structure for the first time.

Configurational disorder gives rise to a small, but possibly significant entropic contribution to the crystal’s free energy, which can be calculated in several ways, but perhaps most efficiently with symmetry-adapted ensemble theory (Grau-Crespo & Hamad, 2015 ▸; Habgood *et al.*, 2011 ▸; Woollam *et al.*, 2018 ▸).

## 2. Motivation, organization and approach

### 2.1. Motivation

Over the years, the blind tests of CSP have showcased the evolution of CSP techniques, highlighting the increasing accuracy of energy models, the expanding role of DFT calculations, and the need to consider many subtle effects that contribute to the stability ranking. The lessons learned from these blind tests have informed ongoing research efforts and continue to inspire advancements in the field of crystal structure prediction.

Given the importance of structure ranking to the success of a CSP study and the emergence of different methodologies in recent years, it was decided that structure ranking would be benchmarked separately in a controlled exercise, one which would provide a consistent starting point for all ranking methods. This would hopefully give us valuable insights into the current state of crystal structure energy ranking methods, their limitations, and potential directions for further research.

### 2.2. Organization

The seventh blind test was a two-phase initiative and was coordinated by Lily M. Hunnisett (CCDC). The first phase focused on structure generation methods and the second on structure ranking methods. The choice of this format was heavily influenced by feedback received by the CCDC following the sixth blind test. Running from October 2020 until June 2022, the challenges presented were intended to test methods considered state-of-the-art and, in doing so, provoke innovation and continued development of CSP methods.

The structure ranking phase took place over December 2021 to June 2022 and involved the CCDC providing participants with prepared sets of structures to rank in order of likelihood of observation. The prepared sets contained either 100 or 500 structures (dependent on the target compound, see Table 1 ▸); the former to provide a tractable challenge for those with limited resources, and the latter to pose a more realistic ranking exercise than that of previous blind tests. Whilst this allowed a more informative and controlled analysis, it did not mimic how a real-world CSP calculation is performed, where structure ranking is carried out on a larger scale. To participate, it was not a requirement for participants to have taken part in the first phase of the test.

### 2.3. Target compounds

The second phase involved ranking the structures of five target compounds that fit under one of two categories: methods development (XXVII and XXVIII) and pharmaceutical or agrochemical applications (XXXI, XXXII, and XXXIII), see Table 1 ▸, labelled according to the scheme set by previous blind tests (see SI-A Section 6). The methods development category presented systems with diverse chemistry and applications, while the pharmaceutical/agrochemical category aimed to test computational efficiency. A detailed explanation behind the choice of systems and individual descriptions of systems are provided in the preceding report on the first phase of the test. Since targets XXIX and XXX were presented as bespoke challenges – powder X-ray diffraction (PXRD) structure determination and co-crystal stoichiometry prediction – and involved their own ranking exercise (see phase one report), they were not included in this phase.

From extensive experimental investigations (see supplementary information of the first phase of this blind test), the following crystal structures are known: XXVII; one polymorph (Form A, *Z*′ = 1, 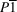), XXVIII; one polymorph (Form A, *Z*′ = 0.5, 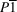), XXXI; two polymorphs (Form A, *Z*′ = 1, *P*2_1_/*c*, and Form B, *Z*′ = 1, *P*2_1_/*c*) in addition to a solvate which was not a target for this exercise, XXXII; two polymorphs (Form A, *Z*′ = 1, 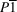, and Form B, *Z*′ = 2, 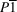), XXXIII; two polymorphs (Form A, *Z*′ = 1, *C*2/*c*, and Form B, *Z*′ = 1, *Pna*2_1_).

It was emphasized to all participating groups that not all target compounds needed to be attempted.

### 2.4. Format of phase two: structure ranking

Similar to the first phase of the test, we invited all those interested in taking part to provide details of their intended ranking method beforehand to, (*a*) avoid duplication of ranking methods, and (*b*) ensure ranking methods were novel and/or benchmarked as demonstrated by reports or published research. In December 2021, participants were provided with sets of structures prepared by the CCDC organizers as described below. Participants were required to rank structures in order of likelihood of observation using their own ranking method and to return results within six months. Organizers analysed which structure(s) matched the experimental forms of each target system and the associated rank. For targets XXXI–XXXIII, where relevant experimental data was available (available in the supplementary information of the first phase of this blind test), the accuracy of predicted thermodynamic relationships was also assessed. An in-person meeting was held in September 2022 in Cambridge, UK, to present and discuss the results, challenges, and outlooks of the test.

### 2.5. Structure set preparation

The structures that were provided to the participants for the ranking exercise were sampled from datasets obtained in the structure generation phase of the seventh blind test. To compare crystal structures, organizers employed the molecular overlay method commonly known as COMPACK (Chisholm & Motherwell, 2005 ▸), since implemented as *Crystal Packing Similarity*, available through *Mercury 2022.2.1* and the CSD Python API 3.0.15 (Macrae *et al.*, 2020 ▸; Groom *et al.*, 2016 ▸). This method, hereafter referred to as ‘COMPACK’, overlays within given distance and angle tolerances, clusters of molecules taken from each crystal and minimizes the root mean-square distance (RMSD) between atoms, typically omitting hydrogen atoms. The method thus returns the number of molecules that could be overlaid and the RMSD.

The structure sets were first populated with structures of interest: experimental representatives and potential matches to undetermined forms showed by PXRD data. For each target system, the closest matches (lowest RMSD upon COMPACK comparison) predicted from CSP were selected as representatives for the experimentally known form(s). For targets XXVII and XXXII where additional polymorphs were showed by PXRD only and crystal structures had not been conclusively determined, a search by PXRD similarity was carried out across all CSP structures using the PXRD similarity measure by de Gelder *et al.* (2001 ▸), as implemented in the CSD Python API. The structures with the largest similarity score were included in the set (15 structures for XXVII, and 53 structures for XXXII), see SI-A Tables 20 and 21. Each of the selected structures was then optimized under constraints using a CSD knowledge-based force field, see item (4) in the protocol below. A structural comparison using COMPACK was carried out to ensure each experimental representative structure matched the original experimentally determined crystal structure.

Experimental forms of targets XXVII, XXXI and XXXII contained disorder. For this ranking exercise, the major and minor components of disorder were included as separate structures for XXXI, which exhibited disorder of the fluorinated ring combining two components of 0.6:0.4 occupancy. The structures included in the list were generated by CSP in the first phase of this blind test. However, separate components were not included for XXVII and XXXII; the disorder of XXVII was not known until after the test, see Section 5.1. It was decided by the organizers not to include the minor component of XXXII Form A since it was not generated by CSP in the first phase, so would require manual input to recreate the disorder, a rotation of a terminal difluoromethyl group, which could unintentionally have provided a clue to the experimental crystal structure.

The sampling process for the remainder of the structure sets followed the below protocol for each target molecule:

(1) All predicted structures from each group were combined into a single global dataset.

(2) The global dataset was clustered with COMPACK to form groups of similar structures using a leaders clustering approach (Spath, 1980 ▸) and sorted in order of cluster size.

(3) An initial sample of 2000 − *n* structures (where *n* is the number of structures of interest already selected as described above) were selected to include in the set: clusters with the largest number of common structures identified in the previous step of which a single structure was selected at random. These represented the most frequently predicted structures across all groups.

(4) The sampled structures were optimized under constraints using a CSD knowledge-based force field (Cole *et al.*, 2016 ▸), and fitness score and density were calculated. Unit-cell parameters, global molecular rotation and translation, and internal atomic torsional rotations were optimized in this step, constraining each parameter from changing by more than 3% of its start value. This allowed slight perturbation of the original structural geometry to avoid easy identification by a group of a structure generated by their own method, while preventing a significant structural change that could push the structure out of the local potential energy basin.

(5) The final sample (containing 100 or 500 structures depending on the target compound) were selected according to the calculated fitness score of the CSD-based force field. Of the overall energy range (indicated by fitness score), 50% of structures represented the lowest third, and 25% of structures in each of the remaining thirds. The structures were iterated through in order of frequency of observation across the CSP landscapes when populating the sample until the desired energy ranges were sufficiently populated.

(6) A full optimization (no constraints) using the CSD force field was carried out on each sampled structure and compared against its starting structure to ensure the structure had not deviated from the corresponding energy well.

(7) To ensure anonymity of participants, atom labels and the order of the atoms in each CIF file were standardized using the CSD Python API. Each structure was also named and numbered in a consistent way.

## 3. Computational methods used in this blind test

### 3.1. Categorization of computational methods

In total, 28 groups participated in at least one part of this blind test. Of those, 22 took part in the structure ranking phase of the test. An overview of the methods applied by the various groups is given in Table 2 ▸.

The seventh blind test ranking exercise introduces several new energy-based approaches, particularly dimer or multimer electronic structure calculations and machine learned potentials, alongside developments of the methods used in previous blind tests and preliminary results with a novel method not based on thermodynamics. The computational methods applied in this exercise can be coarsely divided into three main categories based on the level of theory primarily applied. Category A: periodic DFT-D methods (Groups 2, 3, 4, 5, 9, 10, 11, 12, 14, 20, 22); B: methods based on dividing the crystal into molecules (6, 18, 19, 21, 24, 26 and 27); C: Any other method (Groups 7, 12, 15, 16 and 17). These can be further sorted into nine subcategories, which will now be described.

### 3.2. A. Periodic DFT-D methods

#### 3.2.1. GGA density functionals

Generalized gradient approximation functionals were employed by Groups 4, 5 and 22.

Group 4 applied the D3BJ (Grimme *et al.*, 2011 ▸) dispersion-corrected PBE functional with the projector augmented wave (PAW) method (Kresse & Joubert, 1999 ▸), with a 500 eV plane wave kinetic energy cutoff and sampling only the Γ-point of the Brillouin zone. Group 4 additionally calculated a lattice-vibrational contribution to the free energy for the lowest energy structures with harmonic phonons by D4 dispersion-corrected third-order self-consistent DFTB theory with 3ob-3-1 parameterization (Červinka *et al.*, 2016 ▸; Gaus *et al.*, 2013 ▸).

Group 5 applied a three-stage approach, optimizing structures with plane wave PBE-D3BJ while (i) fixing the unit cell (500 eV cutoff), (ii) relaxing the cell (500 eV cutoff), (3) relaxing the cell with tighter convergence criteria and a larger 600 eV cutoff.

Group 22 first assessed the performance of a few different DFT-D methods (PBE-D3, PBE-MBD, PBE0-MBD) against results from a synthon approach (Sarma & Desiraju, 2002 ▸; Abardeh *et al.*, 2022 ▸), before choosing to optimize all structures at the PBE-D3 level of theory (Grimme *et al.*, 2010 ▸). The PBE-D3 ranking was considered the more reliable because its low-energy structures more often contained synthons detected by a CSD search in the experimental crystal structures of similar compounds.

#### 3.2.2. Beyond GGA functionals

Ranking methods from a further four groups (9, 10, 11 and 14) primarily applied functionals considered to be above GGA functionals in the ‘Jacob’s ladder’ of density functional approximations (Perdew & Schmidt, 2001 ▸).

Group 9 fully optimized all structures using the rSCAN metaGGA functional and MBD dispersion correction and on-the-fly generated ultrasoft pseudopotentials, with kinetic energy cutoff values dependent on the system.

Group 10 performed a hierarchical ranking process where lattice energies for all structures were first calculated with the non-local dispersion-inclusive optPBE-vdW density functional (Klimeš *et al.*, 2009 ▸), then re-evaluated using a level of theory chosen by a decision tree to best match MP2 energies (Abramov *et al.*, 2021 ▸). For molecule XXVII the final lattice energies were calculated with optPBE-vdW, for molecule XXVIII, the r^2^SCAN-D4 metaGGA functional was used, and for molecules XXXI–XXXIII PBE0-MBD was used.

Free energies for all targets except XXVIII were calculated by applying a modified version of the Einstein crystal method with a pseudo-supercritical path approach (Frenkel & Ladd, 1984 ▸; Eike *et al.*, 2005 ▸). For molecule XXVIII, harmonic phonon frequencies were calculated with third-order self-consistent charge DFTB in the *DFTB*+ program (Hourahine *et al.*, 2020 ▸), using custom Slater–Koster parameters for Cu together with the 3ob-3-1 set.

Group 11 first carried out geometry optimizations with the GGA functional B86bPBE and XDM dispersion in two steps of increasing strictness of relaxation convergence. Final rankings were based on subsequent single point energies calculated with a hybrid functional that combines B86bPBE-XDM with either 25% or 50% Hartree–Fock exchange (Otero-de-la-Roza *et al.*, 2019 ▸; Price *et al.*, 2023*a* ▸).

Group 14 initially optimized structures using the optPBE-vdW level of theory, first fully optimizing all structures setting a criterion for the largest force on atoms to 0.02 eV Å^−1^, then optimizing atomic positions only with a force cut-off of 0.001 eV Å^−1^. Final energies were calculated using the Random Phase Approximation (RPA) and SCAN functional, applying PAW potentials.

#### 3.2.3. Periodic DFT-D with monomer or multimer corrections

In addition to applying DFT-D methods, Groups 2, 3 and 20 applied energy corrections based on monomer or multimer energies.

Group 2 primarily used the B86bPBE-XDM GGA functional with PAW potentials. Final energies incorporated a conformational energy correction: the energy difference between DFT and a higher level of theory, either domain-based local pair-natural orbital (DLPNO) coupled cluster theory (CCSD) or spin-component-scaled dispersion-corrected second-order Møller–Plesset perturbation theory (SCS-MP2D) (Greenwell *et al.*, 2022 ▸), calculated using gas phase calculations on each monomer in the unit cell. Specifically for molecule XXXII, pre-optimizations were also carried out using dispersion- and basis set superposition error-corrected Hartree–Fock calculations in a minimal basis set, the so-called HF-3c method (Brandenburg & Grimme, 2014*b* ▸).

Group 3 applied a multi-step approach, applying geometry optimizations at the PBE-MBD level of theory followed by optimizations that embedded multimers with PBE0-MBD into PBE-MBD (PBE0-MBD:PBE-MBD) (Loboda *et al.*, 2018 ▸; Hoja *et al.*, 2023 ▸); a subtractive multimer embedding scheme where PBE-MBD periodic calculations are first carried out, then monomer and dimer energies are replaced with values from PBE0-MBD calculations. Final rankings were based on energies calculated at this level of theory with tight convergence criteria. Additionally, free energies were calculated from harmonic phonons at either the PBE-MBD (XXVII, XXVIII, XXXII) or PBE0-MBD:PBE-MBD (XXXI, XXXIII) level of theory.

Group 20 applied a multi-step approach of increasing level of theory. Tailor-made force fields and machine learned algorithms were applied to filter out high-energy structures prior to the most computationally demanding calculations. The final energies are free energies at room temperature as calculated with the TRHu(ST) method (Firaha *et al.*, 2023 ▸), with the exceptions that for compounds XXVII and XXVIII no monomer MP2D correction was added and for XXVIII, *ab initio* minimizations and phonon calculations were done with periodic PBE+MBD.

### 3.3. B. Mixed intra- and intermolecular models

Periodic DFT methods are fairly accurate in predicting relative energies, but they come with significant computational costs. On the other hand, force fields can be useful due to their speed but they may not be optimal for CSP applications where relative energies need to be calculated very accurately, with errors on the order of 1 kJ mol^−1^. In between these two methods, there are a series of approaches that limit *ab initio* quantum mechanical calculations to a certain subgroup of the crystal, such as dimers, single molecules or fragments. The resulting lattice energy is made up of two main components. The dominant contribution to the lattice energy, the intermolecular energy (*U*_inter_) is modelled by summing up the interactions within the crystals, as obtained from electronic structure calculations on the multimers, or by atomistic calculations using analytical anisotropic force fields, which are parameterized from electronic structure calculations on molecules or dimers, or by empirical fitting.

#### 3.3.1. Electronic structure calculations on multimers

After an initial step of conformer and crystal geometry optimization, using atoms in the asymmetric unit as a reference, a molecular cluster of finite size defined by a distance cutoff is created. The approaches adopted by Groups 19 and 21, the dimer expansion and the Fragment Molecular Orbital (FMO) method (Kitaura *et al.*, 1999 ▸), calculate the intermolecular term of the cluster as a sum of the energies of dimers and inter-fragments, respectively. Single-point calculations are performed for pairs of the reference and any other molecule or fragment within the cluster. In the case of FMO method, the calculations are performed in the presence of environmental electrostatic potential to take into account contributions from other fragments (Nakano *et al.*, 2002 ▸). The dimer energy and FMO calculations can be performed in parallel, taking advantage of modern high-performance computers.

Group 19 calculates dimer energies with B3LYP-D3BJ for dimers at less than 6 Å from each other and HF-3c for those up to 12 Å distance.

Group 21 calculates inter-fragment interaction energies with FMO at MP2 level of theory (FMO-MP2) (Mochizuki *et al.*, 2004*a* ▸, Mochizuki *et al.*, 2004*b* ▸) using molecular clusters within a radius of 12 Å.

#### 3.3.2. Force fields fitted to quantum chemical calculations

Improvements in structures’ energy evaluations are reached when a force field is parameterized specifically for the target compound instead of adopting common transferable force fields. In this blind test, Groups 26 and 27 used either symmetry-adapted perturbation theory based on DFT description of monomers, SAPT(DFT) (Misquitta *et al.*, 2005 ▸), or supermolecular DFT-D in parameterizing *ab initio*-based intermolecular force fields (Nikhar & Szalewicz, 2022 ▸). Thousands of dimer configurations were generated to evaluate intermolecular interaction energies and fit a system-specific intermolecular potential using the autoPES codes (Metz *et al.*, 2016 ▸). In most cases, the intramolecular term of the lattice energy was determined by the DFT-D energy difference relative to the most stable conformer. For target XXXI, GAFF monomer deformation penalties were used. Monomers in CCDC-provided lists of polymorphs were constraint-optimized, with the soft dihedral angles determining the shape of molecule fixed at their original values. All structures were optimized using either rigid monomers or flexible monomers with modified GAFF intramonomer energies.

The parameterization of intermolecular force fields was based on supermolecular PBE0-D3BJ for compounds XXVII, XXVIII, and XXXII and on SAPT(DFT) for compounds XXXI and XXXIII. An additional step of molecular dynamics simulations was performed for targets XXXI and XXXIII to assess structures’ stability at finite temperatures. For these, the intramolecular term was represented by reparameterized GAFF. For XXXI, the equilibrium bond lengths and angles were replaced by the *ab initio* values from the equilibrium conformer. For XXXIII, in addition the force constants and torsional parameters were fitted to *ab initio* calculations on a grid of 2000 ▸ monomer conformations.

#### 3.3.3. Electronic structure calculation on individual molecules

In the Ψ_*mol*_ method (Price *et al.*, 2010 ▸) used by Groups 6, 18 and 24, *ab initio* calculations at the molecular level are used to both estimate the energy penalty of the different conformers, Δ*E*_*intra*_, and model the electrostatic term of *U*_inter_. The charge density of each conformer is used to calculate either atomic point charges from the RHF/6-31 G(d,p) charge density (Group 6) or more sophisticated distributed multipoles from B97/6-31G(d,p) or PBE0/6-31G(d,p) charge densities (Groups 18 and 24, respectively). *U*_inter_ is completed with an empirical repulsion-dispersion potential, often based on the FIT parameterization (Coombes *et al.*, 1996 ▸).

Group 6 used RHF/6-31G(d,p) calculations to fit atomic point charges while Groups 18 and 24 generated multipoles starting from B97D/6-31G(d,p) and PBE0/6-31G(d,p), respectively.

#### 3.3.4. General purpose force field models

Group 17 was alone in using a general purpose atom–atom potential, namely the Dreiding force field (Mayo *et al.*, 1990 ▸) and atomic point charges derived by electrostatic fitting to B3LYP/6-311G(d,p) charge densities. With this energy model, they performed quasi-harmonic approximation lattice dynamics with *GULP* (Gale & Rohl, 2003 ▸) to obtain the free energies used for ranking the structures of molecule XXVII.

### 3.4. C. Alternative approaches

#### 3.4.1. Machine learned models

Machine learning methods were used by several groups and this constitutes a major development in the field of CSP. While Group 12 used a relatively simple machine learning method, a Gaussian process regression (GPR) (Deringer *et al.*, 2021 ▸) trained on DFTB results, more advanced methods for ranking the structures with machine learning were used by Groups 15 and 16. Group 15 used transfer learning to enhance the ANI-2x model. The training data consisted of single point r^2^SCAN calculations on the sets of crystal structures provided by the CCDC. The ANI-2x neural network potential was retrained with torchANI on the new data, using both energies and atomic forces (Devereux *et al.*, 2020 ▸; Gao *et al.*, 2020 ▸). Structure optimization and fully anharmonic vibrational free energy calculations were then performed with the stochastic self-consistent harmonic approximation (SSCHA) using the SSCHA software (Monacelli *et al.*, 2021 ▸).

Group 16 performed unit cell relaxations and calculated lattice energies and quasi-harmonic lattice vibrational free energies with system-specific AIMNet neural network potentials trained to each blind test target compound (Zubatyuk *et al.*, 2021 ▸; Anstine *et al.*, 2023 ▸). Training data for the target specific AIMNet models were based on molecular clusters extracted from the crystal structures, which contained the reference molecule and up to ten of its neighbours. Additional sampling of out-of-equilibrium configurations was performed by running short MD trajectories on the molecular clusters. To accelerate convergence, the models were pre-trained using GFN-xTB (Bannwarth *et al.*, 2019 ▸). Subsequently, transfer learning was performed to DFT data for smaller clusters containing up to three molecules, calculated using PBE-D4/def2-TZVPP. When applied to crystal structures, the AIMNet model accounts for long-range interaction with Ewald sum approximation (Ewald, 1921 ▸) to the Coulomb energy of the crystal, and pairwise *C*_6_ and *C*_8_ dispersion energy terms. The many-body dispersion terms were calculated using the Axilrod–Teller–Muto formula with *DFT-D4* software (Caldeweyher *et al.*, 2017 ▸). Additional details and analyses are available in SI-B Section 13.

#### 3.4.2. Non-energy methods

A newly developed non-energy based method was applied by Group 7, ranking crystal structures of molecule XXXI based on a topological analysis approach. For each structure a number of vectors are calculated, including the molecular inertial eigenvectors, ring plane normal vectors, vectors based on the positions of atoms with substantial Gasteiger partial charges (Gasteiger & Marsili, 1978 ▸) (|*q*| > 0.1 *e*), and between atoms forming close contacts. The scoring function is based on observed correlations in the angles between these vectors and the crystal’s Miller planes (Tuckerman & Galanakis, 2023 ▸).

## 4. Assessment of results

Geometry-optimized crystal structures submitted by the participants were compared against experimental data using COMPACK. A 30-molecule cluster was applied with distance and angle tolerances of 25% and 25°, respectively, to compare the predictions against the experimental structures. In comparison with the structure generation phase of the blind test (which applied tolerances of 35% and 35°), stricter tolerances were set since the more accurate methods used in this phase can be assumed to yield structures that closely match the experimental reference structures. Tolerances were, however, looser than those used in previous blind tests since it has been shown that matching structures may exhibit a large degree of structural difference (Sacchi *et al.*, 2020 ▸; Mayo & Johnson, 2021 ▸; Mayo *et al.*, 2022 ▸). Predicted structures demonstrating a 30 out of 30 molecule match and RMSD < 1 Å were visualized using *Mercury* to confirm each match.

## 5. Results and discussion

Here, we discuss and compare several energy ranking methods, including force fields, density functional theory (DFT), tight binding approximations, and machine learning techniques. The performance of these methods in the context of the seventh blind test are assessed, highlighting factors that contribute to their ability to predict experimentally observed polymorphs and facilitate the rational design of materials with desired properties and functions.

Solid-form screening has been carried out for all target systems to increase the likelihood of the thermodynamically stable forms being present among the target structures for this blind test. It is important to note that such a screening does not constitute a guarantee for the observation of the stable forms. If it was otherwise, late-appearing forms and disappearing polymorphs would not be a substantial risk pharmaceutical companies are confronted with in late development. Indeed, it has been reported that for 15–45% of the pharmaceutical compounds in late development, *i.e.* after a significant amount of experimental screening, the stable form has not been discovered yet (Neumann & van de Streek, 2018 ▸). For target systems with multiple experimentally observed forms (XXXI, XXXII and XXXIII have two structurally characterized polymorphs each), we have analysed whether the predicted relative stabilities qualitatively reflected those observed experimentally.

Overall, methods employing periodic dispersion-corrected DFT – PBE0 with MBD or D3; B86bPBE-XDM; rSCAN-MBD – were found to most consistently rank the experimental forms amongst the lowest in energy. Mixed results were found for machine learning methods; System-specific AIMNet machine learned potentials (Zubatyuk *et al.*, 2021 ▸) performed consistently well, while other ML methods, including a Gaussian process regression and free energies calculated with ML interatomic potentials performed inconsistently, as would be expected from the extent to which the specific molecules’ intermolecular interactions can be approximated as molecularly pairwise additive and the extent to which the different approaches accounted for the non-pairwise additivity. Methods that consisted of a combination of an intermolecular force field and intramolecular quantum-chemical components also performed inconsistently. In agreement with previous blind tests, general purpose force fields and a non-energy-based method did not perform well.

Comparisons of the submitted structures versus the CCDC-provided set were carried out by the organizers using the pointwise distance distribution approach (Widdowson *et al.*, 2021 ▸), see Table 10 in SI-A. Structural differences indicated that all groups except 7 and 12 optimized the structures using their own methods. A few groups directly optimized the structures provided by CCDC with the model used for the final energy evaluation, see Table 2 ▸. Step 4 of the structure set preparation (Section 2.5) produced some molecules with higher conformational energies than would have been sampled in many CSP workflows. This meant that many methods that divided the crystal into molecules had to first adapt the conformations as a novel step. This resulted in multiple cases where geometry optimizations led away from the experimentally observed structure. The use of periodic DFT-D always maintained the structure.

In predicting the thermodynamic stability relationship of polymorphs, the overall accuracy of CSP structure ranking methods was mixed. However, in many cases where the incorrect stability order was predicted, the experimental energy difference between polymorphs is likely to be small as was strongly indicated from calculated relative lattice energies.

The greatest consistency was observed for target compound XXXIII, with the majority of methods correctly predicting the stability relationship between the two experimental forms. Additionally, periodic DFT-D methods proved to be the most accurate in this case, with the majority of groups predicting the most stable form as the global minimum in the crystal energy landscape.

Results are reported below per target system. The raw data for each submission is available in SI-C.

### 5.1. XXVII

There exist two experimentally determined crystal structures for one form of XXVII [Form A, CSD: XIFZOF (290 K), XIFXOF01 (100 K)]. During structure set preparation for molecule XXVII, a large amount of void space was found in structures generated by some groups. After analysis of overall void space, see SI-A Table 8, it was decided to exclude structures from Groups 12 and 17 in the sampling process due to many structures having unreasonably low densities.

The compilation of 100 structures provided to all participant groups was seeded with a CSP structure representative of the experimentally determined crystal structure of Form A at 90 K. Due to a limit on the number of comparisons due to topological symmetry in the CCDC implementation of COMPACK, the closest match to Form A (90 K) was originally incorrectly identified (analysis from Oct 2021), meaning the selected representative structure of the experimental form was a structural variant in terms of the isopropyl group conformation. The CCDC implementation has since been updated.

Based on discussions between organizers and participants, it was agreed for CCDC organizers to investigate the nature of disorder further using molecular dynamics and metadynamics simulations to determine whether an ensemble of varying triisopropylsilane (TIPS) group conformations needed to be considered in the final analysis. The subsequent work suggested that there is dynamic disorder related to the rotation of the isopropyl groups with respect to the pentacene and a possible static disorder related to the change in conformation of the two TIPS groups (see the supplementary information of phase one of this blind test). The results were therefore analysed based on the molecular ‘core’ only (excluding the triisopropyl groups).

**Figure 1:**
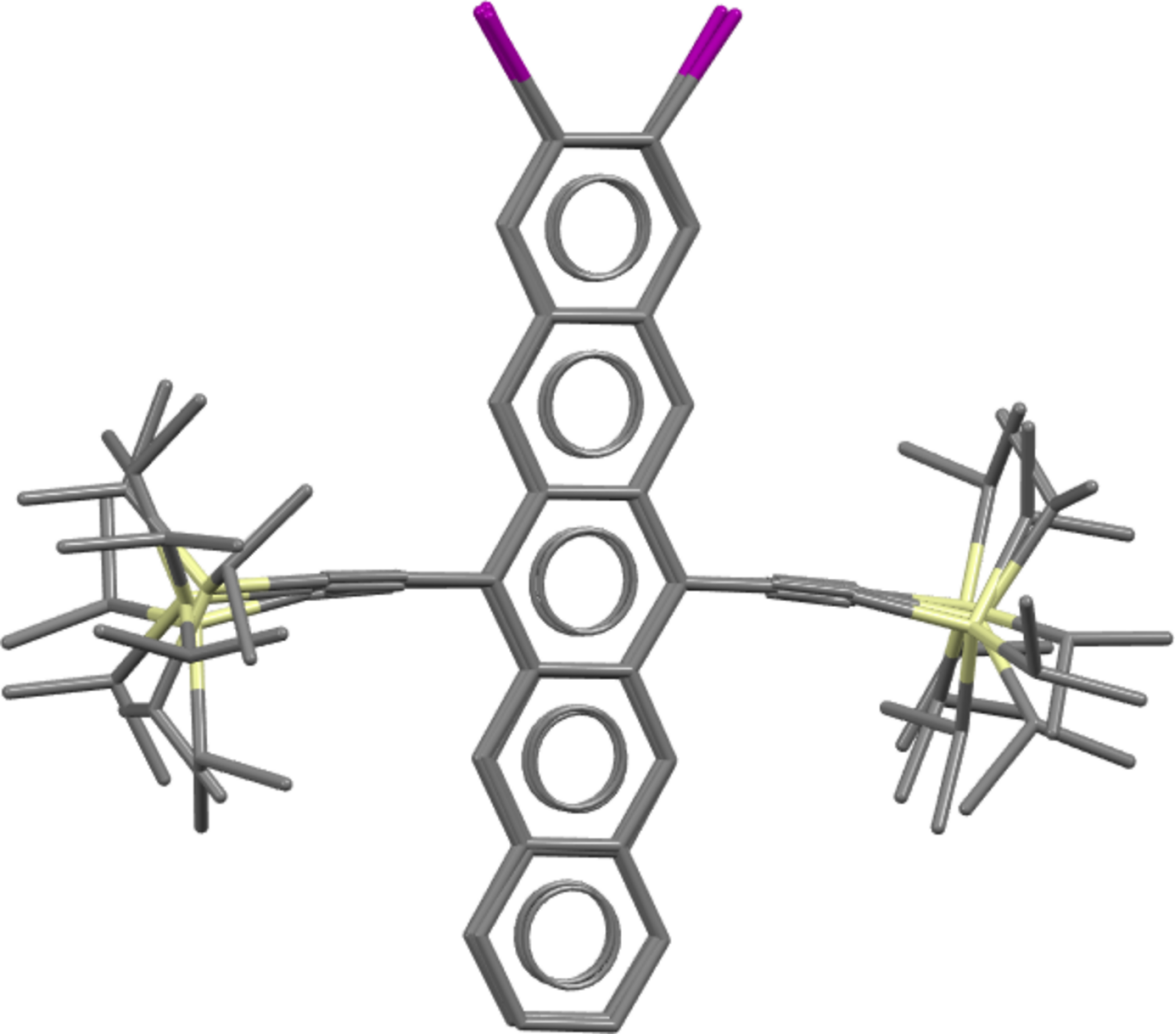
Structure overlay of structures 28, 38, and 59 from the provided structure set for target XXVII, demonstrating the variation in TIPS conformation.

Analysis of the set of 100 structures prepared and provided by the CCDC indicates that the structure of the experimental Form A (90 K) was not present in the list, but four structures (28, 38, 59 and 61 matching with 0.53, 0.80, 0.83 and 0.57 Å RMSD_30_, respectively) exhibit the same crystal packing with varied isopropyl conformations (matches identified upon comparisons excluding isopropyl groups). Molecular overlays were carried out (allowing inversion) comparing all four of the ‘core’-matching structures. This demonstrated that structures 28 and 61 represent the same structure (also confirmed with COMPACK), whilst structures 38 and 59 represent different structural variants due to differing isopropyl conformations, see Fig. 1 ▸.

**Figure 2:**
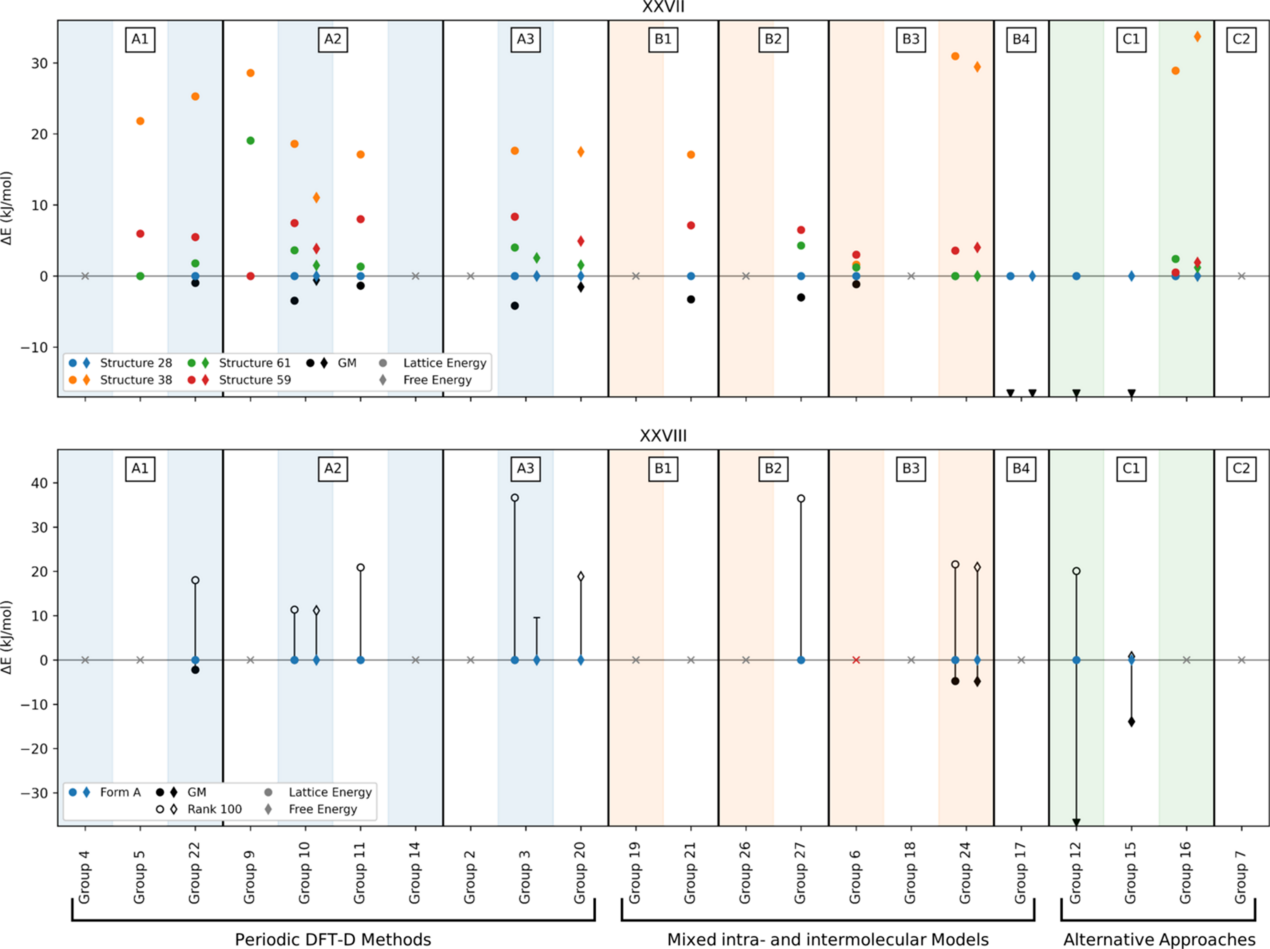
(Top) Lattice and free energy difference between structure 28 of molecule XXVII and structures 38, 61 and 59 which share the same core packing of the experimental form. The global minimum (black filled circle) of each group has been included to show if other packings were found to be more stable within an energy model. (Bottom) Lattice and free energy difference with respect to Form A of molecule XXVIII. The energy range between the global minimum (black filled circle) and the 100th ranked structure (open circle) is shown to highlight the position of the experimental structure within the CSP set. If a subset of less than 100 structures was used in the energy calculation, filled circle is used instead of an open circle. As the initial set of structures includes 500 structures, the experimental one can lie outside of the 1st–100th range. In both plots, groups are organized as in Table 2 ▸, with the methodology class shown at the top. Groups that did not participate in the ranking of these two compounds are shown with a grey cross, while those that did not reproduce the geometry of the most stable polymorph are displayed with a red cross. If any of the structures’ energies lie outside of the energy range considered, this is shown with an arrow.

Analysis of the ranks and calculated relative energies of these structural variants submitted by each group demonstrates that, despite exhibiting common core crystal packing, the difference in isopropyl conformation translates to a large variation in energy, see Fig. 2 ▸. Of the 15 participating methods, 11 (Groups 3, 5, 6, 10, 11, 16, 20, 21, 22, 24, 27) ranked structure 28 as lowest in energy amongst the four conformational variants, while all (except Group 6) ranked structure 38 as the highest in energy, see SI-A Table 11. The lowest in energy of the four ‘core’-matching structures was ranked as the global minimum by methods from four groups (5, 9, 16, 24) at 0 K, and three groups (3, 16, 24) at room temperature, see Table 3 ▸ and SI-A Table 11. Since the experimental structure can be regarded as a dynamic ensemble rather than a single point, the previous statement does not discredit methods that have not ranked any of the ‘core’-matching structures as the global minimum. Group 20 demonstrated from *post hoc* calculations (see SI-B Section 17) that an alternative isopropyl conformation ranked at the global minimum if taken into account (corresponding to one of the experimental structure determinations), as could be the case for other groups if similar post-analysis were carried out.

Where both lattice and free energies were calculated (Groups 3, 10, 16, 17, 24), free energies brought the experimental crystal packing closer in energy to the global minimum – becoming the global minimum for Group 3 – while Groups 16 and 24 ranked it as the global minimum with both lattice and free energies.

### 5.2. XXVIII

There exists one experimentally determined crystal structure of XXVIII (Form A, CSD: OJIGOG01). The experimental crystal structure of XXVIII was coincidentally published by an external group during the first phase of this blind test. Despite this, XXVIII was still included in the structure ranking exercise with disclosure that all ten participating groups had access to the experimentally determined form.

It was reported that most of the polymorph screen experiments resulted in oxidative dimerization of the ligand with no observed crystallization of the desired complex. This could indicate kinetic factors may be involved and so there is a degree of uncertainty whether Form A is the thermodynamically most stable form.

The majority (71%) of the provided structure set represent the experimentally observed *trans* square planar geometry, while 25% of the structures are *cis* square planar, and 4% exhibit the see-saw conformation, see Fig. 2 ▸.

**Figure 3:**
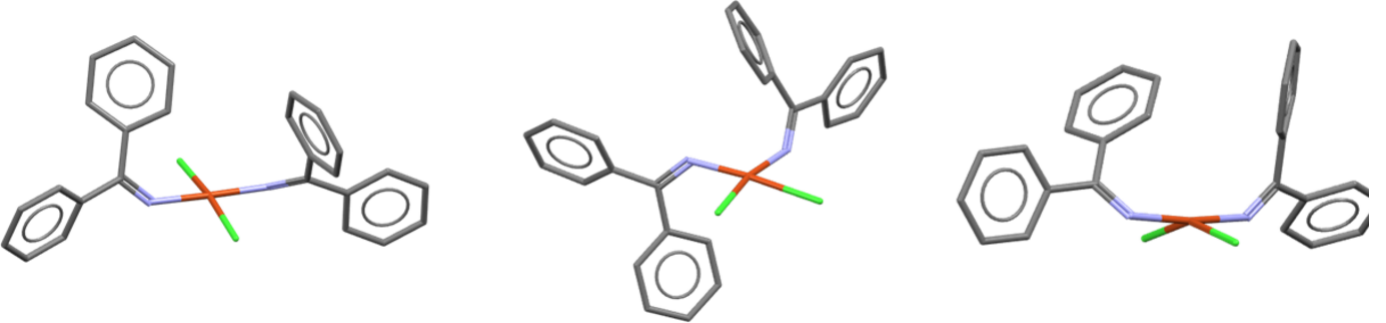
Examples of *trans*-square planar (left), *cis*-square planar (centre), and see-saw geometries of target XXVIII (right).

The experimental structure was ranked relatively low in energy in the majority of cases with five of the ten participating groups (3, 10, 11, 20, and 27) ranking the observed form as the most stable, see Fig. 3 ▸. Methods that ranked the experimental form as the most stable applied dispersion-corrected DFT; PBE0-MBD was applied in some form by three groups, and PBE0-D3 and B86bPBE-XDM by the remainder.

No differences were observed in the ranking of Form A at low *versus* ambient temperatures for those groups that calculated both lattice and free energies, with Groups 3 and 10 predicting Form A to be the most stable structure while Group 24 calculated a relative energy of around +4.8 kJ mol^−1^ from the global minimum in both cases.

### 5.3. XXXI

There exist three experimentally determined polymorphs of XXXI: Forms A (CSD: ZEHFUR02), B (CSD: ZEHFUR) and C (CSD: ZEHFUR01). Form C is a channel-type solvate containing unresolved solvent (see supplementary information from phase one) and therefore falls outside the scope of this ranking exercise. Form A contained disorder of the fluorinated ring (see SI-A Fig. 1) resulting in two disorder components, both of which were represented by structures in the lists provided to participants. Competitive slurry experiments have demonstrated an enantiotropic relationship between Form A (more stable above 55°C) and Form B (more stable below 55°C). Since no group calculated the properties of the crystals at temperatures higher than 55°C, results are discussed with respect to the stability relationship at lower temperatures, with Form B being the most stable form.

Many methods rank Forms A and B within the lowest 5 kJ mol^−1^ of structures: eight out of 20 methods at 0 K (the periodic DFT methods of Groups 2, 3, 5, 9, 10, 14 and 22, and the machine learned model of Group 16) and three out of seven methods at ambient temperature (Groups 3, 10, 20).

All periodic DFT-D methods calculated Forms A and B to be within 5.7 kJ mol^−1^ from the global minimum. Of those methods, that of Group 10 (at 0 K) and Group 3 (at ambient temperature) ranked Form B as the global minimum, while Groups 2, 5, 9, 20 and 22 ranked both forms within the lowest 3 kJ mol^−1^ region. The machine learned model of Group 16 also predicted the observed structures within the same region at 0 K, although energies were higher at ambient temperature.

**Figure 4:**
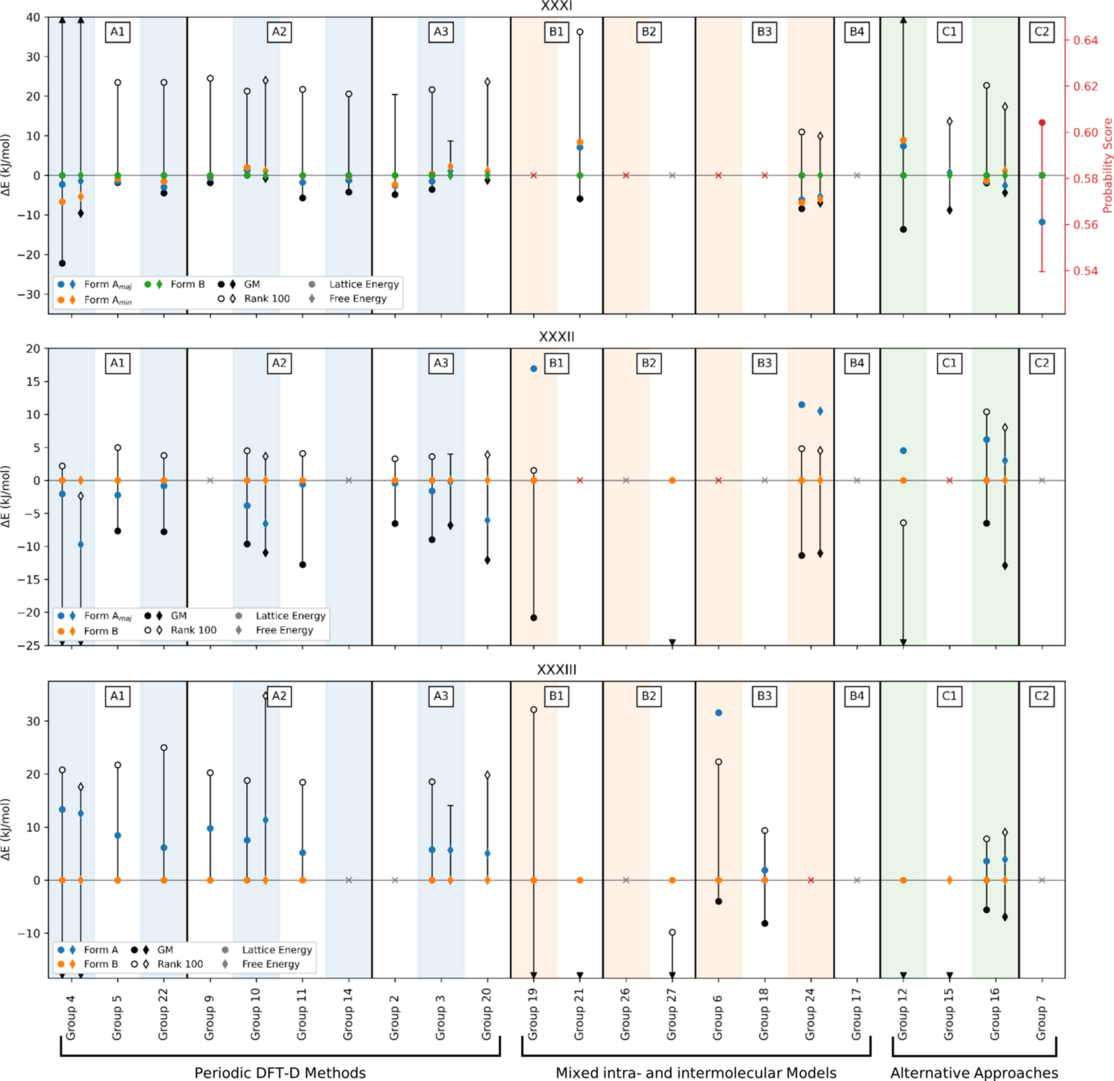
Lattice and free energy difference of the experimental structures with respect to the most stable polymorph of molecule XXXI (top), XXXII (middle) and XXXIII (bottom). The energy range between the global minimum (black filled circle) and the 100th-ranked structure (open circle) is shown to highlight the position of the experimental structures within the CSP set. If a subset of less than 100 structures was used in the energy calculation, the filled circle is used instead of an open circle. As the initial set of structures of compounds XXXII and XXXIII includes 500 structures, the experimental one can lie outside of the 1st–100th range. Groups are organized as in Table 2 ▸, with the methodology class shown at the top of each plot. Groups that did not participate in the ranking of these compounds are shown with a grey cross, while those that did not reproduce the geometry of the most stable polymorph are displayed with a red cross. If any of the structures’ energies lie outside of the energy range considered, this is shown with an arrow. For molecule XXXI, Group 7 used a ranking method not based on thermodynamics but on topological probabilities (highlighted in red). In this case the higher the score, the more probable it is to observe a structure.

Of the 20 groups that submitted results for XXXI, five groups ranked Form B as the most stable of the observed forms; two groups ranked Form B as the most stable of all theoretical structures (Group 10 at 0 K and Group 3 at 300 K), while Groups 12 and 21 ranked the structure at 12th (+13.6 kJ mol^−1^) and 4th (+5.9 kJ mol^−1^) respectively, and Group 7 at 26th (where a geometric-based scoring function was used), see Fig. 4 ▸.

Periodic DFT-D methods predicted Forms A and B to be close in energy (within 3 kJ mol^−1^ in nearly all cases, falling within the error bars of accuracy of most DFT methods (Abramov *et al.*, 2021 ▸; Firaha *et al.*, 2023 ▸), an indicator that the two are likely of such similar stability that a correct prediction of the relationship may be beyond the accuracy capable of periodic DFT-D in this case.

Periodic DFT-D methods of Groups 3, 4 and 10, the machine learned model of Group 16, and the Δ*E*_intra_/*U*_inter_ method of Group 24 provided both lattice and free energies. The incorporation of temperature effects was important for this system, resulting in stabilized relative energies for the experimental Forms by Groups 3, 4 and 24. Significantly, for Group 3, thermal contributions resulted in a different (correct) prediction of the relative stability relationship.

It is noted for Groups 6, 18, 19, and 26 (Δ*E*_intra_/*U*_inter_ methods) geometry optimization of the experimental representative of Form B resulted in a different structure. Notably, this did not occur for any periodic DFT-D methods. Interestingly, of those groups, Group 26 ranked the deviated structure as the lowest in energy on the solid-form landscape.

### 5.4. XXXII

There exist two known crystal structures of XXXII determined from single crystal X-ray diffraction at 90 K; Form A (CSD: JEKVII) and Form B (CSD: JEKVII01). Experimental slurry experiments have demonstrated that Form B is more stable than Form A at room temperature (RT) and above (see the experimental report in the supplementary information of phase one of this blind test). Form B was also determined to be the most stable of all known anhydrous forms (including those showed by PXRD alone, though not included in this study due to no structural determination).

An additional structure of Form B at RT was determined from PXRD. The prepared set of structures supplied to participants contained this structural determination. However, upon analysing the results, it was found in all cases that the structure no longer resembled the starting structure after geometry optimization. A redetermination of the crystal structure of Form B at RT was later provided by Group 20 based on a predicted structure and the original PXRD data. This showed greater agreement with the PXRD data and was subsequently corroborated by solid-state nuclear magnetic resonance (NMR) data using ^13^C CPMAS and ^1^H–^13^C CP-HETCOR experiments (see the experimental report in the supplementary information of phase one of this blind test). Structural comparisons by the organizers concluded that, despite a small difference in symmetry (from space group 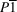 at 90 K to *P*2_1_/*c* at RT) between the two structures due to a minor conformational change, the structural difference was not large enough for any structural comparison tools to differentiate unambiguously when assigning structural matches to either one. Visual crystal packing and molecule overlays of the two structures are provided in SI-A Figs. 2–4. This relates to a wider issue on the definition of isostructurality which is raised in the report of the first phase of this blind test. The analysis therefore only involved one of the two structures (Form B at 90 K).

For this exercise, there were two structures present in the list to analyse: the major disorder component of Form A (referred to as Form A), and the low temperature structure of Form B (referred to as Form B). There were two cases where the geometry optimized structure no longer matched the experimental form (the mixed method of Group 6 for Form A, and the machine learned model of Group 15 for Form B). Similarly to what was observed for molecule XXXI, this did not occur for periodic DFT methods.

No method predicted any of the experimental forms to reside within 3 kJ mol^−1^ of the global minimum. Furthermore, only four groups (machine-learned models of Groups 12 and 16, and Δ*E*_intra_/*U*_inter_ methods of Groups 19 and 24) predicted Form B to be more stable than Form A in line with reported experimental data, see Fig. 4 ▸. For results where both lattice and free energies are reported, temperature corrections seemingly offer no clear improvement on either ranking experimental structures close to the global minimum or the correct ranking of the stability relationship between Forms A and B with only Groups 3, 16 and 24 showing a smaller energy difference between these two observed forms.

Attempts by the experimental providers of this system to reproduce and determine the structures of the unresolved forms showed previously by low-quality PXRD patterns were unsuccessful. The several additional unknown forms of XXXII raise uncertainty on whether the true global minimum structure has been observed experimentally, with the overall predictions – particularly the consensus of the periodic DFT-D results – further fuelling this uncertainty. Indeed, it has been predicted previously (Neumann & van de Streek, 2018 ▸) that for between 15–45% of all chemical compounds, the thermodynamically most stable form has not yet been found because it is kinetically hindered. It is possible that XXXII is one of those cases.

The CCDC-prepared structure sets contained many structures (see SI-A Table 21) which showed high similarity to PXRD patterns of unresolved polymorphs of XXXII. These patterns (labelled H, K, L, N, P, and R) are available in the supplementary information experimental report of phase one of this blind test. Analysis of results submitted for this structure ranking exercise (see SI-A Tables 22–24) found that structures with large PXRD similarity to pattern H were ranked within 3 kJ mol^−1^ of Form B. Additionally, structures with substantial PXRD similarity to N were ranked lower in energy than Form B in the majority of cases. Comparisons of structures with high PXRD similarity to patterns H and N show nearly identical packings but molecules with either or both the difluoromethyl and the oxazine groups in different conformations, see SI-A Figs. 5 and 6, suggesting disorder could be present in these two forms. Further analyses, although desired, are beyond the scope of this study, but the initial observations outlined here serve as further evidence for the possibility of a more stable structure of XXXII yet to be observed experimentally.

### 5.5. XXXIII

Two crystal structures of XXXIII are known to exist; Form A (CSD: ZEGWAN) and Form B (CSD: ZEGWAN01). Experimental studies show Form A is a disappearing polymorph (see the experimental report in the supplementary information of phase one of this blind test).

In comparison with other target systems, the most consistency observed across all methods was seen for target XXXIII, with 14 out of 17 groups correctly ranking Form B as more stable than Form A, see Fig. 4 ▸.

All periodic DFT methods ranked Form B as the lowest energy structure on the landscape via both 0 K lattice energies (Groups 3, 5, 9, 10, 11, and 22), and free energies at room temperature (Groups 3, 10, and 20). Notably, Group 20 predicted the stable Form B as rank 1 and the metastable Form A as rank 2. None of the remaining methods (machine learning-based, force field, or mixed methods) predicted either experimental form as the global minimum: Form B, the most stable form, was ranked at 20th (+5.6 kJ mol^−1^) by Group 16 (machine learning-based), 22nd (+8.1 kJ mol^−1^) and 14th (+53.4 kJ mol^−1^) by Groups 18 and 19 (mixed methods), and third (+4.0 kJ mol^−1^) by Group 6 (force field-based). This possibly reflects that charge distributions of ions in crystals can differ significantly from those of the isolated ions, making the molecular pairwise additive approximation less appropriate.

There was one case of the experimental representative no longer resembling the known structure following geometry optimization (Form B by Group 24, attributed to human error – see SI-B Section 20). All methods, except for the machine learned model of Group 12 and the Δ*E*_intra_/*U*_inter_ method of Group 21, predicted Form B to be more stable than Form A at both 0 K and room temperature, in agreement with the experimental observation that Form A became difficult to crystallize once Form B had been isolated.

### 5.6. Free energy results

In cases where both lattice energies and free energies were reported, overall little advantage was gained for targets XXXII and XXXIII in terms of improvement in predicted rank or relative energies of experimental forms. Cases have been reported previously in which state-of-the-art dispersion-inclusive DFT methods, even with finite temperature corrections, failed to reproduce the experimentally observed order of stability, for example for the α and β forms of the energetic material HMX (O’Connor *et al.*, 2023 ▸) The influence on predictions can be observed for target XXXI where the majority of free energy methods from classes A and B (Groups 3, 4, 24) predicted experimental forms to be closer to the global minimum. In one case (Group 3), free energies provided the correct relative stability relationship of polymorphs in contrast to energies without temperature corrections. Additionally, improvements were observed in relative energies for structures matching the experimental packing for XXVII. However, in the case of the machine-learned method from Group 16, calculated free energies worsened the relative energies and ranks of experimental forms for molecules XXXI and XXXII, whilst little impact was observed for XXXIII.

The results here demonstrate that, while free energies may not offer a clear improvement in predictions for some systems, the application of methods in classes A and B for target XXXI has demonstrated the benefit that temperature corrections can have on predicted relative stabilities. Free energies are essential for predicting whether the relative stability of polymorphs changes with temperature.

### 5.7. Resource utilization

All participants were required to report an estimate of the number of central processing unit (CPU) core hours utilized for ranking each list of structures, shown in Table 4 ▸. While the reported numbers provide an idea of method efficiency, they should be handled in the context of used hardware.

It is evident from the resources reported for XXVII (where only 100 structures were ranked compared with 500 for XXVIII, XXXII, and XXXIII) that calculation intensity heavily depends on the chemistry and complexity of the system. Furthermore, there is a large variation in the reported computational costs, even amongst those within the same category of theory. This is a reflection of whether multiple rounds of optimizations or energy calculations were applied. The calculation of vibrational frequencies for obtaining free energies, especially for large molecules, is for instance extremely time-demanding when performed with periodic DFT methods. While some groups applied multiple rounds of calculations on all structures, others have made efforts towards limiting the number of structures undergoing calculations at the highest levels of theory. Free energies were calculated in addition to lattice energies by Groups 3, 4, 10, 16, and 24, creating further variation in resources utilized.

Another consideration for variation in resources utilized is the likely effect of structure anonymization by the organizers in structure list preparation (see step four in the protocol outlined in Section 2.5) on methods based on dividing the crystal into molecules (categories B1–B3, Table 2 ▸). Groups 24, 26 and 27 reported high intramolecular energies in many of the supplied structures (see SI-B Sections 20 and 21), requiring corrections at the molecular level and leading to the use of further resources. The effects of this extended beyond resource utilization as raised by Groups 26 and 27 at the blind test meeting (see Section 6).

Positive observations to note are the machine learning-based approach from Group 16 (AIMNet) which offers an efficient alternative to DFT methods. Additionally, the topological analysis method (Group 7) represents an interesting new approach to performing extremely fast ranking by completely avoiding energy ranking. However, this method cannot be used for geometry optimization and its current performance leaves much to be desired. These demonstrate that genuinely new ideas are still being developed as efficient alternatives to periodic DFT methods.

It is difficult to judge the progress made in efficiency of ranking methods alone as we have not previously analysed the separate components of CSP methods in such a way. Considering this point, we acknowledge that the workflows applied here may differ vastly due to the difficulty in drawing a line between the structure generation and ranking parts of any one CSP method, and due to the different interpretations of the ranking exercise by different groups as a result of the synthetic nature of the structure lists provided by organizers. Nevertheless, we hope the numbers reported here serve as a useful indication and motivation for future research into efficient structure ranking approaches.

## 6. Blind Test meeting

A two-day in-person meeting was held in Cambridge, UK in September 2022, following the final results submissions. This provided an opportunity for participants to present their results to fellow investigators, blind test organizers, and active researchers in the CSP community from both industry and academia. A session was also held between participants and organizers to discuss any issues that arose during the test and to reflect on the current and possible future blind test initiatives. We include here some topics from the discussions which were important with respect to either the test results or future initiatives and research.

The structure ranking exercise constructed by the CCDC organizers, whilst providing a valuable opportunity to benchmark and compare ranking methods alone, did not reflect how CSP is carried out in reality. This point was highlighted during discussions, and it was emphasized that the relatively small numbers of structures provided were better suited to more intensive methods and did not provide an incentive to use recently developed more efficient methods (such as those based on machine learning). This was acknowledged by the organizers and serves as valuable feedback to guide future such initiatives.

In the case of molecule XXVII where the experimental form was not provided in the ranking list due to problems encountered with COMPACK (described in Section 5.1), many groups expressed interest in being provided with the experimental structure to calculate relative energy and add to their results. The likelihood of dynamic disorder in the system was discussed which motivated subsequent MD and metadynamics work outlined in Section 5.1, culminating in the decision to analyse the results based on the molecular ‘core’ only (excluding the TIPS groups).

During the meeting, Groups 26 and 27 (a collaboration of individuals across the two groups) presented an issue with the guidance of the exercise due to the assumption that the organizers deformed the crystal structures during structure list preparation. This was assumed due to gas phase calculations of monomers revealing a large majority to exhibit high energies relative to the global minimum. This deformation is suspected to be due to step 4 of preparation, see Section 2.5. For transparency and understanding, comparisons of the structures before and after this preparation step were carried out by the organizers and results are reported in Tables 3–7 in SI-A. At the same time, the experimental representatives were very close to experimental crystals: the monomers’ RMSD was between 0.0 Å and 0.28 Å, while the crystal RMSD_30_ values were between 0.180 Å and 0.332 Å, except for target XXVII. All representatives were within the acceptance criteria. The analysis of monomer energies led the group to carry out a workflow which significantly changed the provided crystal structures and consequently worsened their outcomes. It was agreed amongst participants and organizers that this was attributed to an unfortunate assumption by the participating group rather than ambiguous instructions for the exercise from the organizers.

## 7. Conclusions of the ranking exercise

In this paper, we have presented the computational methods for crystal structure energy ranking employed in the seventh blind test of crystal structure prediction organized by the CCDC. Allowing for a more effective and fair analysis of ranking methods via two separate assessments, this second phase of the test involved 22 of the total 28 participant groups. The results of this study offer valuable insights into the performance of various current approaches, including force field based methods, density functional theory (DFT) calculations, and machine learning techniques.

Assessing the accuracy of CSP ranking methods for this constructed exercise can be simplified into two questions: (i) Are the experimentally observed structures ranked at or close to the global minimum of the structure set (with consideration of the expected error bars in keeping with the limitations of the method applied (Abramov *et al.*, 2021 ▸; Firaha *et al.*, 2023 ▸)? (ii) Did the method correctly reproduce the relative stability relationship observed experimentally in the case of multiple observed polymorphs? The periodic dispersion-corrected GGA density functional-based methods of Groups 3, 10, and 20 produced results in excellent agreement with experimental data satisfying both assessments outlined above for all other targets, with the exception of target XXXII where confidence is low in the completeness of the experimental solid-form observations (discussed in Section 5.4). All three methods employed the calculation of free energies. The results overall showed that temperature corrections, where calculated, provided an improvement of calculated energies relative to global minima for targets XXVII and XXXI, but offered no clear improvement for the remainder of target compounds. Taking error into account for the prediction of stability relationships, Groups 2, 5, 9 and 22 also produced results in line with experimental data. A highly challenging exercise for ranking methods was predicting the relative stability of Forms A and B of XXXI (where Form B is more stable than Form A) which were consistently calculated to have 1–2 kJ mol^−1^ difference in many cases.

When periodic GGA DFT-D is used, it is probably often worthwhile to add corrections such as monomer or dimer calculations with MP2D or (doubly) hybrid functionals, or single point periodic calculations at a higher level of theory, such as a metaGGA or hybrid functional. Such approaches make the predictions less sensitive to spurious self-interaction errors. However, a comparison of the GGA and hybrid functional rankings suggests that none of the compounds considered in the current blind test exhibited significant self-interaction errors.

Machine learning techniques show promise as an emerging approach for crystal structure energy ranking. By training on existing data, these methods can provide rapid and sometimes accurate predictions. However, their performance is contingent upon the quality of the training data and the choice of machine learning algorithm. Machine learned potentials were utilized by three groups, one of which (the system-specific AIMNet potentials used by Group 16) was applied for the calculation of final ranking free energies yielding reasonable results in agreement with the experiment in many cases, thus demonstrating a viable alternative to DFT that, once trained, is orders of magnitude faster.

A new fast-ranking method based solely on crystal structure geometry was proposed by Group 7 and applied in this test, and similar statistical methods have been used in several previous tests. These methods still face the challenge of accurate predictions. Despite overall poor predictive ability, it is still valuable to explore alternative routes to ranking, particularly now that the question is being raised on whether we are reaching the limits of DFT capabilities.

The use of the Dreiding force field as the sole ranking method showed poor performance. Although a valuable tool when dealing with large numbers of structures in the structure generation stages of CSP workflows, general purpose force fields are not suitable for the accurate calculation of relative polymorph stability.

The resources utilized by CSP methods have continued to increase as methods have developed, as showed by the reported CPU hours here and in previous blind tests. This exercise encouraged development for methods focusing on high accuracy given the relatively small numbers of structures provided to rank. Since environmental impact and method efficiency are important considerations for future developments, such future initiatives should aim to provide a suitable platform to test and benchmark methods aiming to optimize speed and resource utilization.

Based on these findings, we propose several avenues for future research and development in the field of crystal structure energy ranking:

(1) Exploration of more advanced DFT approaches, including hybrid functionals and monomer corrections to enhance the reliability of DFT-based calculations.

(2) Increase the accuracy of low-cost alternatives to periodic DFT methods. Machine-learning techniques or gas-phase *ab initio* calculations can help develop a robust workflow to parameterize more accurate force fields tailored to the molecule or design new potentials. Larger and more diverse data sets can facilitate the training of machine learning approaches and speed up CSP calculations.

(3) Integration of multiple energy ranking methods, leveraging their respective strengths, to develop more robust and accurate hybrid approaches for crystal structure prediction.

(4) The computation of free energies at ambient temperature appears to be beneficial for obtaining the right answer for the right reason; *i.e.* modelling real physical effects. Whether computed free energies add value, though, is also heavily dependent on the system as showed in this exercise. However, free energy corrections improve the predictive ability only when calculated with already accurate lattice energy methods. The development of efficient free energy methods is desirable.

(5) Increasing the efficiency of CSP methods with serious considerations for resources utilized and environmental impact. New benchmarking tests with a more focused assessment, on geometric optimization algorithms for example, would be beneficial.

A number of groups have provided post-analysis of their own results, involving further calculations on experimental forms, benchmarking against alternative methods, and providing explanations for any unexpected or unreasonable results. We urge the reader to see SI-B for further reading. Additionally, all participant groups were encouraged to provide additional explanations and assessments via their own peer-reviewed reports which we also refer the reader to.

## 8. Overall conclusions of the blind test and outlook

The seventh blind test fulfilled its objective of allowing the participants to benchmark and improve their methodologies. It can be seen in the supplementary information (SI-B) from many participating groups that they did have to adjust their approaches to meet the challenges posed by the different target compounds. Many groups have already further developed their methods in response to the results. For example, improvements in the non-thermodynamic topological prediction results for compound XXXI tackled by Group 7 are given explicitly in SI-B Section 6.

The seventh blind test aimed at being more realistic, going beyond asking whether it was possible to predict a carefully selected structure of defined *Z*′ with no disorder. The introduction of polymorph screening was intended to support the likelihood that the most thermodynamically stable form was among the targets. The extensive experimental work during this blind test led to a valuable interplay between experiment and theory, revealing the complexity of the organic solid state. The computational investigations revealed that the target structure XXVII was dynamically disordered, which considerably complicated the analysis of the results. One group proposed a better crystal structure for XXXII Form B than originally provided. This is a good example of how computational methods can lead to a reinterpretation of experimental data.

It is impressive that the *Z*′ = 3 crystal structure of XXIX could be solved by comparing simulated laboratory PXRD data. It highlights the now widely used and successful cross-correlation-based PXRD pattern similarity measure, and the necessity of optimizing the lattice parameters in order to maximize the pattern similarity.

The detection of disorder in XXVII, XXXI Form A, the XXX 2:1 cocrystal and XXXII Form A, raises important problems in the distinction between static and dynamic disorder, whether CSP can predict such disorder, and how the thermodynamic effects of disorder should be calculated.

The splitting of the blind test into two phases, structure generation and ranking, had the great advantage of enabling a wider range of methods to be applied and more effective comparisons to be made. However, while it is clear that improving the computational efficiency of CSP is a worthwhile aim, the disruption of established CSP workflows prevented much meaningful analysis of success in terms of CPU efficiency. The selection and anonymization process of the 100 or 500 structures also resulted in jumping to another minimum on some potential surfaces. Also implicit in the huge range of structures generated in part one of this blind test is the need to tackle the over-prediction problem, so that structures which are effectively duplicates at experimental temperatures are eliminated.

The overall conclusion of the first phase is that there are search methods that can successfully locate the experimental structures, provided the search is exhaustive enough. For example, the *Z*′ = 3 structure of XXIX would not have been found in a standard CSP search. These show the value of using experimental information to tailor the CSP search to the system.

The results of the energy ranking submissions are extremely encouraging as they suggest some very different approaches to balancing the changes in intramolecular conformation with the various types of intermolecular forces in a system are converging. Predicting the observed structures to reside within the likely energy range of solution-grown polymorphs proved challenging for some systems. Highly sophisticated methods are clearly needed to be confident that a specific structure is the most thermodynamically stable, but the importance of different contributions will depend on the molecule and the types of crystal packing that are thermodynamically competitive. The choice of systems for this blind test has pushed many participants beyond their comfort zone. However, many of the expensive corrections, such as going beyond the PBE GGA functional in periodic DFT-D, using a highly converged monomer energy for correction, or evaluating the free energy with or without anharmonicity, appear less important in these than other polymorphic systems. To put recent developments in the field of energy calculations of organic polymorphs to the test, future blind tests should probe the ability to predict enthalpy differences between polymorphs and the transition temperatures of enantiotropic systems. Building on the co-crystal challenge of system XXX, future blind tests should also assess the ability to compare different compositions, generalizing to other multi-component systems such as hydrates. It will be interesting to see whether alternative ranking methods can reduce the number of structures where state-of-the-art thermodynamic calculation methods need to be used.

Thus the seventh blind test has significantly increased the diversity and size of the target systems and the nature of the challenges. By including polymorph screening results, it is clear that the solid state landscape of some of these molecules is more complex than as described by a *Z*′ = 1 structure without disorder. The best methods have been very successful, and the progress since the sixth blind test is remarkable. However, the computational cost is so large that right-sizing and balancing the computational and experimental effort in studying solid form landscapes will be very dependent on the aim of the study.

## 9. Glossary

**Ψ_mol_** A method that combines a quantum chemical model of individual molecules and an atomistic force field model for the intermolecular interactions in the crystalline environment.

**ANI-2x** A neural network type of machine learning atomic potential

**API** Application programming interface

**ASE** Atomic Simulation Environment, a Python library

**B86bPBE** A GGA density functional consisting of the exchange functional proposed by Becke in 1986 and the PBE correlation functional

**B97D** A variation of Becke’s GGA functional introduced in 1997 ▸, including Grimme’s dispersion correction

**B3LYP** A variation of Becke’s three-parameter hybrid functional with the LYP (Lee–Yang–Parr) correlation term

**CCSD(T)** Coupled cluster theory with full single and double excitations and noniterated triple excitations

**CIF** Crystallographic Information File, a standardized file format for crystallographic data

**COMPACK** An algorithm for calculating crystal structure similarity based on atomic distances

**COMPASS** Condensed-phase Optimized Molecular Potentials for Atomistic Simulation Studies, a force field

**D3** Grimme’s dispersion correction, version 3.

**D3BJ** The D3 dispersion correction with Becke–Johnson damping

**D4** Grimme’s dispersion correction, version 4

**DFT-D** Dispersion-corrected density functional theory

**DFTB** Density functional tight binding

***F*_vib_** The lattice vibrational contribution to the free energy

**FIRE** Fast Inertial Relaxation Engine, an optimization algorithm

**GAFF** General Amber Force Field

**GFN-xTB** A self-consistent and dispersion-corrected DFTB method

**GGA** Generalized gradient approximation

**GPR** Gaussian process regression, a machine learning method

**HF** The Hartree–Fock method

**MBD** Many body dispersion, a dispersion correction

**MD** Molecular dynamics, a simulation method

**ML** Machine learning

**MP2** Second-order Møller–Plesset perturbation theory

**MP2D** Dispersion-corrected second-order Møller–Plesset perturbation theory

**OPLS** Optimized potentials for liquid simulations, a force field

**PBE** The GGA exchange correlation functional by Perdew, Burke and Ernzerhof

**PBE0** A hybrid exchange-correlation functional, PBE with 25% Hartree–Fock exchange

**PIXEL** A method for calculating intermolecular interaction energies by direct numerical integration over electron densities

**RHF** The Restricted Hartree–Fock method

**SAPT** Symmetry-adapted perturbation theory

**SCAN** The strongly constrained and appropriately normed meta-GGA density functional

**SOAP** Smooth overlap of atomic positions, a descriptor that encodes regions of atomic geometries

**TIPS** Triisopropylsilane, a functional group

**X23** A benchmark dataset consisting of 23 crystal structures of small organic molecules

**XDM** The exchange-hole dipole moment dispersion correction

## Supplementary Material

SI-A. Additional information, tables and figures. DOI: 10.1107/S2052520624008679/aw5094sup1.pdf

SI-B. Methods SI per group. DOI: 10.1107/S2052520624008679/aw5094sup2.pdf

SI-C. Theoretically generated structures, CCDC lists and predictions. DOI: 10.1107/S2052520624008679/aw5094sup3.zip

## Figures and Tables

**Table 1:** Two-dimensional chemical structures of the target compounds investigated, and the number of structures provided to participants for the ranking exercise

Target	Chemical diagram	Experimental structures	Number of structures provided	Experimental investigators
XXVII	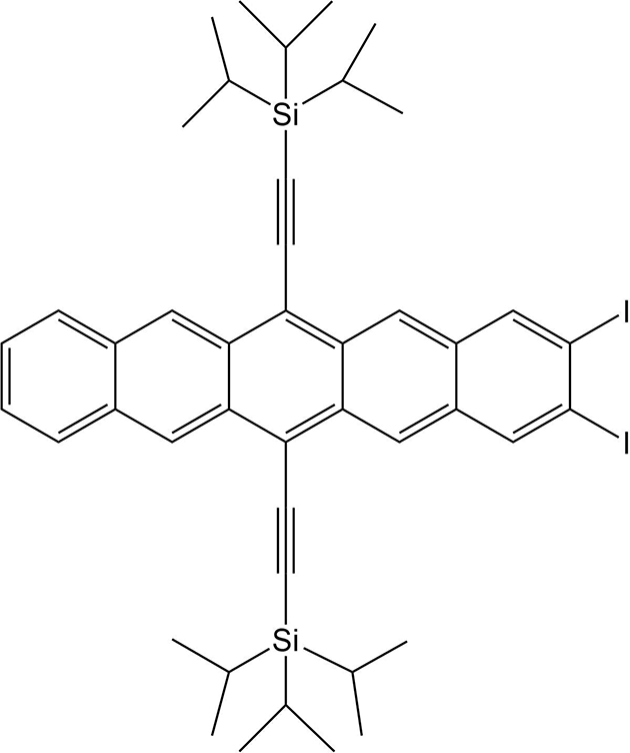	Form A (known)	100	J. A. Anthony, S. Parkin (F. Tarczynski)
		Form B (unknown)		
XXVIII	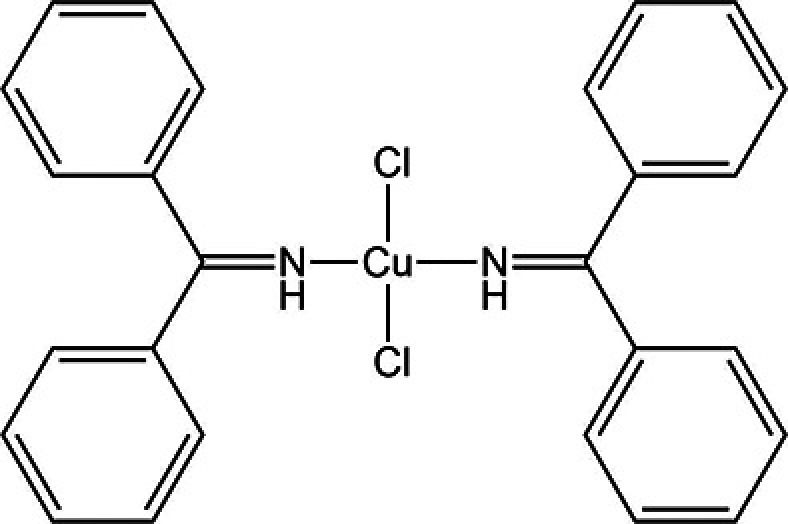	Form A (known)	500	M. R. J. Elsegood, P. F. Kelly, L. Wilkinson (M. R. Probert, J. Weatherston)
XXXI	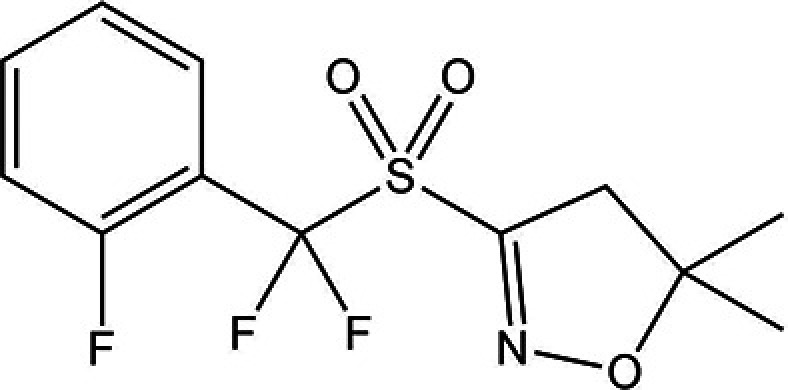	Form A (known)	100	J. Hone, A. Keates, I. Jones
		Form B (known)		
XXXII	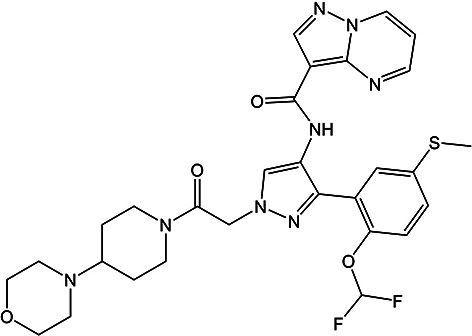	Form A (known)	500	A. DiPasquale, J. W. Lubach
		Form B (known)		
		Form C (unknown)		
		Form D (unknown)		
		Form E (unknown)		
		Form F (unknown)		
		Form G (unknown)		
		Form H (unknown)		
XXXIII	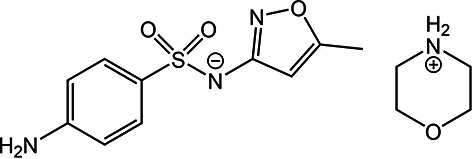	Form A (known)	500	S. Coles, S. Aitipamula, J. Cadden
		Form B (known)		

**Table 2:** Summary of the structure ranking methods utilized by each participating group * indicates the principal investigator of each group.

Class	Subclass	Group	Group members	Ranking method	Free energy
A. Periodic DFT-D methods	A1. GGA density functionals	4	Červinka*, Kostková, Ludík, Touš	PBE-D3/PAW electronic energy	Harmonic DFTB3-D phonons
		5	Day*, Arnold, Bramley, Butler, Taylor	PBE+GD3BJ	–
		22	Oganov*, Maryewski, Momenzadeh Abardeh, Bahrami, Salimi	PBE-D3	–
	A2. Beyond GGA functionals	9	Hušák*	rSCAN+MBD	–
		10	Jin*, Yang, L. Tan, Chang, Sun, X. Shi, C. Liu, Yue, Fu, Lin, Y. Zhou, Z. Liu, Zeng, Li, B. Shi, T. Zhou, Greenwell, Bellucci, Sekharan	XXVII: optPBE-vdW, XXVIII: r^2^SCAN-D4, XXXI-XXXIII: PBE0-MBD	Einstein crystals
		11	Johnson*, Otero-de-la-Roza*, Clarke, Rumson, Mayo, A. J. A. Price	B86bPBE-XDM/NAO optimization; 25% and 50% hybrid single points	
		14	Klimeš*	RPA(SCAN) on optPBE-vdW structures	–
	A3. DFT-D with monomer or multimer corrections	2	Beran*, Cook, Unzueta	B86bPBE-XDM + monomer energy corrections	–
		3	Boese*, List, Strasser, Hoja, Braun	PBE0+MBD multimers embedded into periodic PBE+MBD	Harmonic PBE-MBD or PBE0-MBD:PBE-MBD
		20	Neumann*, Anelli, Woollam, Abraham, Dietrich, Firaha, Helfferich, Y. M. Liu, Mattei, Sasikumar, Tkatchenko, van de Streek	Cascade of DFT methods of increasing accuracy	PBE(0)+MBD+MP2D+*F*_vib_

B. Intra (Δ*E*_intra_) and inter (*U*_inter_) molecular contributions to lattice energy	B1. Electronic structure calculations on multimers	19	Muddana*, Jain, Darden, Skillman	Atomic multipole force field, IEFF / HF-3c + DFT	–
		21	Obata*, Goto*, Utsumi, Ikabata, Okuwaki, Fukuzawa, Nakayama, Yonemochi	XXVII: PBE-D3; XXXI, XXXII: FMO-MP2/6-31G†; XXXIII: FMO-MP2/6-31G(d)	–
	B2. Force fields fitted to SAPT calculations	26	Szalewicz*, Ishaque, Nikhar, Podeszwa, Rogal, Vogt-Maranto	XXXI: SAPT(DFT) fitted potentials (intermolecular), modified GAFF (intramolecular), flexible-monomer minimizations and simulations, only GAFF monomer energies used	XXXI: MD simulations in NPT ensemble
		27	Szalewicz*, Tuckerman*, Bhardwaj, Chan, Hong, Ishaque, Jing, Melkumov, Nikhar, Podeszwa, Rehman, Rogal, Song, Vogt-Maranto	SAPT(DFT) or PBE0-D3 fitted potentials (intermolecular), PBE0-D3 monomer deformation energy penalties, modified GAFF (intramolecular) in flexible-monomer simulations for XXXIII	XXXIII: MD simulations in NPT ensemble
	B3. Electronic structure calculations on individual molecules	6	van Eijck*	Price-Williams exp-6 potential; RHF/6-31G(d,p) point charges and intramolecular energies	–
		18	Mohamed*, Dhokale, Saeed, Alkhidir, Almehairbi	Atomic multipoles and exp-6	–
		24	S. L. Price*, L. S. Price, Guo	Molecular Ψ_mol_ model, atomic multipoles + empirical exp-6 for intermolecular, Ψ for intramolecular	Rigid-body harmonic phonons
	B4. General purpose force field models	17	Matsui*, Shinohara	Dreiding force field	Quasi-harmonic

C. Alternative approaches	C1. Machine learned models	12	Jose*, Ramteke	Cardinality and Gaussian process regression potential	–
		15	Lončarić*, Bianco, Mladineo, Parunov	ANI-2x retrained on r^2^SCAN single point energies and forces on CCDC structures	Anharmonic by SSCHA
		16	Marom*, Isayev*, Anstine, Deng, Nayal, O’Connor, Tang, Yang, Zubatyuk	System-specific AIMNet machine learned potentials trained on PBE-D4/def2-TZVPP calculations on *N*-mers, up to trimers	Quasi-harmonic
	C2. Ranking not based on thermodynamics	7	Tuckerman*, Galanakis	Topological scoring function	–

**Table 3:** Summary of results for target systems XXVII, XXVIII, XXXI, XXXII, and XXXIII, where numbers are the predicted rank at 0 K and those in brackets at ambient temperature ‘–’ indicates no structure was found to match the experimental form.

		XXVII†	XXVIII‡	XXXI			XXXII		XXXIII	
Class	Group	Form A	Form A	Form A_maj_	Form A_min_	Form B	Form A	Form B	Form A	Form B
A1	4			6 [6]	3 [2]	9 [7]	64 [30]	76 [147]	50 [60]	28 [30]
	5	1		2	3	6	9	24	4	1
A2	22	2	3	2	5	10	21	30	3	1
	9	1		5	7	8			9	1
	10	4 [3]	1 [1]	8 [7]	11 [8]	1 [3]	13 [5]	30 [51]	7 [4]	1 [1]
	11	2	1	8	12	13	31	37	5	1
	14			6	10	11				
A3	2			7	9	17	27	30		
	3	7 [1]	1 [1]	3 [3]	10 [5]	6 [1]	18 [24]	22 [25]	6 [4]	1 [1]
	20	[2]	[1]	[10]	[11]	[6]	[11]	[35]	[2]	[1]
B1	19			2	3	–	337	82	214	14
	21	4		15	22	4	62	–	33	302
B2	26			22	34	–				
	27	3	1				23	487	349	132
B3	6	2	–	–	14	–	–	–	205	3
	18			4	5	–			29	22
	24	1 [1]	6 [6]	5 [10]	2 [2]	47 [43]	209 [195]	41 [42]	4 [6]	–
B4	17	23								
C1	12	25	63	33	38	12	490	129	90	470
	15	[56]	[85§]	[18]	[13]	[12]	[18]	–	–	[288]
	16	1		2 [3]	4 [18]	10 [10]	41 [38]	3 [15]	60 [56]	20 [20]
C2	7			–	36	26				

† Ranks reported correspond to the lowest ranked predicted structure matching the crystal packing of XXVII excluding the isopropyl groups.

‡ The experimental structure of XXVIII was available to all groups due to a coincidental publication, so the exercise was not a true blind test.

§ An alternative originating structure was identified to match the experimental form.

**Table 4:** Summary of CPU core hours reported per target molecule for each group where predictions were attempted

Class	Group	XXVII	XXVIII	XXXI	XXXII	XXXIII	Total	Hardware details
A1	4			30,000	600,000	180,000	810,000	AMD Zen 2 EPYC 7H12
	5	492,544		26,777	448,829	959,728	1,927,878	Intel Skylake 2.0 GHz
	22	48,120	46,080	18,432	34,560	69,120	216,312	Intel Xeon Gold 6230
A2	9	350,000		200,000		1,500,000	2,050,000	AMD Zen 2 EPYC 7H12
	10	786,380	1,689,100	240,000	1,700,000	1,824,016	6,239,496	Intel Xeon Platinum 8124M
	11	442,488	1,444,036	40,124	580,001	349,766	2,856,415	Intel Xeon E5-2683 v4
	14			1,400,000			1,400,000	AMD EPYC 7351
A3	2			?	?		0	AMD 64 GPUs / bespoke cluster
	3	3,000,000	1,100,000	400,000	4,000,000	1,500,000	10,000,000	Intel Xeon X5650, E5-2650 v3, Silver 4214R, Platinum 8174
	20	385,229	379,699	64,512	776,909	77,414	1,683,763	Intel Xeon E5-2650 v4
B1	19			80,000	2,000,000	656,000	2,736,000	Intel Xeon Hasswell E5-2666 v3
	21	163,278		640,875	4,361,740	2,155,256	7,321,149	Intel Xeon Gold 6154, 6258R / FUJITSU A64FX
B2	26			11,458			11,458	Intel Xeon Gold
	27	30,880	54,769		132,606	12,586	230,841	Intel Xeon Platinum, Gold-6132, Xeon E5-2695 v3
B3	6	455	10	55	1,200	195	1,915	Various computers, all CPU times standardized to one 2.66 GHz processor Intel Quad 9400
	18			715		2,666	3,381	Intel Xeon Gold 6230R
	24	1,963	50,266	1,023	86,668	70,193	210,113	Intel Xeon E5-2650v3, L5630 / E5-2660v4 mixed clusters
B4	17	2,732					2,732	Intel Xeon Gold 6154
C1	12	10,000	10,000	10,000	10,000	10,000	50,000	Intel Xeon Gold 6132
	15	36,277	248,763	7,449	148,942	67,289	508,720	AMD EPYC 7401
	16	755,000		80,000	47,000	530,000	1,412,000	AMD EPYC 7742 / Intel Platinum 8280 / Nvidia RTX 3090, GTX 1080, GTX 1080ti / Tesla V100S
C2	7			10			10	Intel Core i7-10750H

## Footnotes

1 www.cost.eu/actions/CA22107
