# Supplementary material for: The seventh blind test of crystal structure prediction: structure ranking methods
Source: Acta Crystallogr B Struct Sci Cryst Eng Mater. 2024 Oct 17;80(Pt 6):548–74. doi: 10.1107/S2052520624008679 (PMC11789160; doi:10.1107/S2052520624008679)
Supplement: Supplementary file 1 [file b-80-00548-sup1.pdf]

Supplementary Information A. The Seventh Blind Test of Crystal Structure Prediction: Structure Ranking Methods

Lily M. Hunnisett, *et al.* *

The Cambridge Crystallographic Data Centre, 12 Union Road, Cambridge CB2 1EZ, UK. E-mail: lhunnisett@ccdc.cam.ac.uk

Contents

1 Additional Details on the 7th Blind Test Target Compounds **3**

2 Structure Selection from Phase 1 **8**

3 Additional Tables **44**

4 Rankings of the Optimised Structures **46**

5 Unknown XXVII and XXXII Forms **55**

6 Previous Blind Tests Target Compounds **62**

7 DOI links for submitted sets of structures **66**

1. Additional Details on the 7th Blind Test Target Compounds

Table 1. *Summary of single crystal structures obtained for each target system.*

Target system	Form	CSD Refcode	Description	Comments
XXVII	A	N/A*	Collected at 90 K, no disorder.	Initial crystal structure obtained prior to the start of test (September 2020). Bromine contamination not correctly included.
XXVII	A	XIGYUL	Collected at 90 K, contains bromine contamination.	Re-refinement of initial crystal structure to include known bromine contamination.
XXVII	A	XIFZOF01	Collected at 100 K, disorder of both TIPS groups.	Redetermination of initial crystal structure, no bromine contamination.
XXVII	A	XIFZOF	Collected at 290 K, disorder of one TIPS group.	Room temperature crystal structure, obtained at end of test (July 2022).
XXVIII	A	OJIGOG01	Collected at 150 K.	Original crystal structure obtained prior to start of test (September 2020).
XXXI	A	ZEHFUR02	Collected at 120 K, disorder of fluorinated ring.	
XXXI	B	ZEHFUR	Collected at 200 K.	
XXXI	C	ZEHFUR01	Collected at 120 K	A presumed solvate. Contains channel-type voids with unresolved solvent.
XXXII	A	JEKVII	Collected at 90 K, disorder of the difluoromethyl group.	
XXXII	B	JEKVII01	Collected at 90 K.	Referred to as 'Form M' in experimental report from the providers.
XXXIII	A	ZEGWAN	Collected at 296 K.	Disappearing polymorph.
XXXIII	B	ZEGWAN01	Collected at 297 K.	

*Structures have not been deposited in the CSD at the time of submission and are instead provided as CIF files.

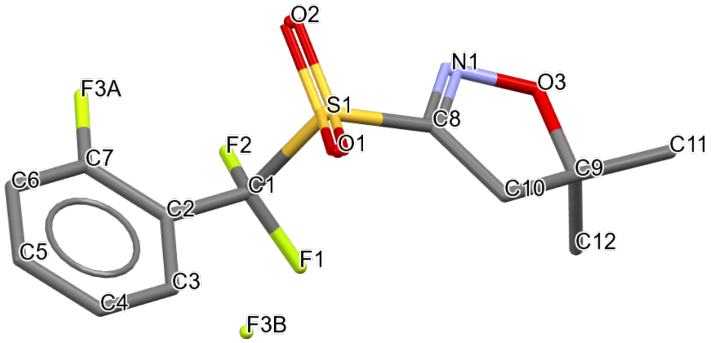

Fig. 1. Visualisation of the disorder present in XXXI Form A, resulting in two possible positions of the fluorine atom at F3A and F3B (hydrogen atoms omitted for clarity).

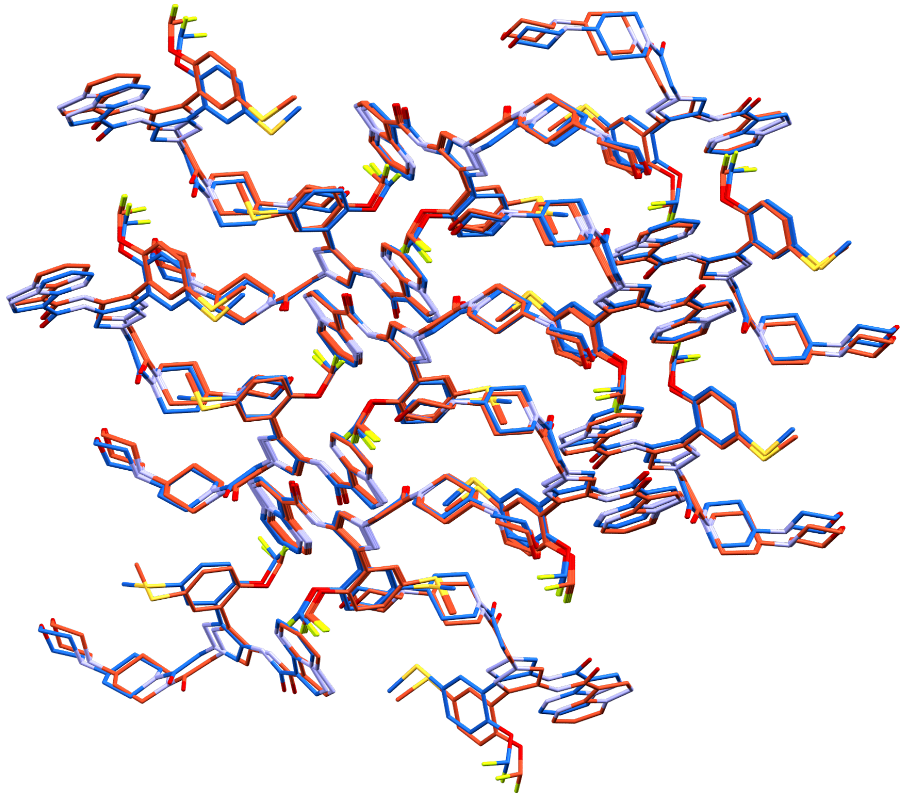

Fig. 2. Visualisation of the comparison of XXXII Form B at 90 K (blue) and Form B at room temperature (red) using Crystal Packing similarity in Mercury. The comparison resulted in a 30/30 molecule match and 0.499 Å RMSD applying applying 25% and 25° distance/angle tolerances.

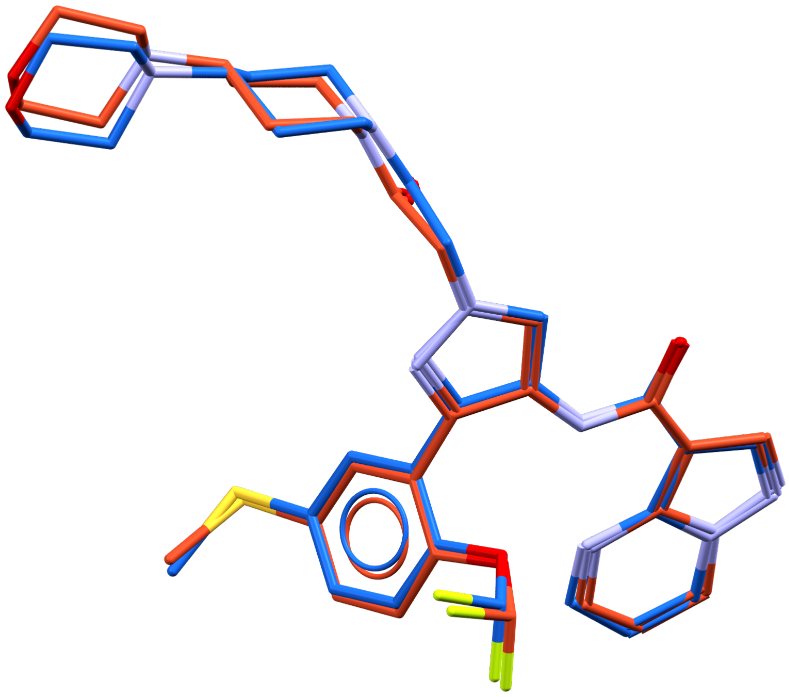

Fig. 3. Visualisation of the molecule overlay of the first molecule in the asymmetric unit of Form B at 90 K versus the single molecule of Form B at room temperature, demonstrating 0.2737 Å RMSD and 0.671 maximum displacement when allowing inversion.

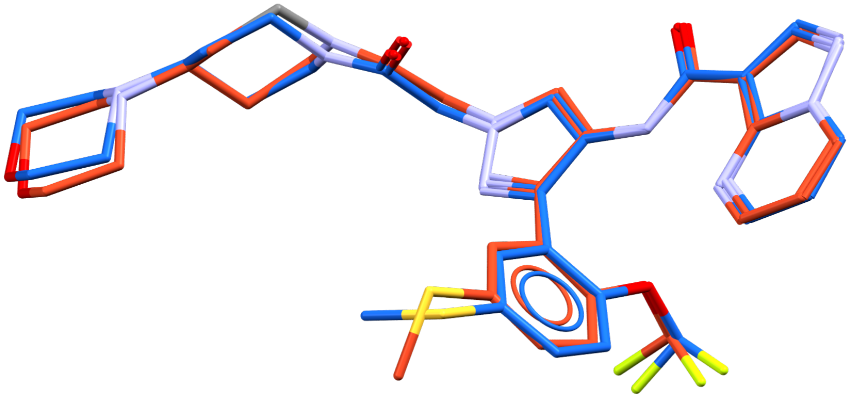

Fig. 4. Visualisation of the molecule overlay of the second molecule in the asymmetric unit of Form B at 90 K versus the single molecule of Form B at room temperature, demonstrating 0.3478 Å RMSD and 1.5381 maximum displacement when allowing inversion.

2. Structure Selection from Phase 1

Table 2. *The structures present in the provided lists to rank which represented the known experimental forms of each target compound (original CSP structures are those submitted by groups in the first phase of the 7th Blind Test and were optimised under heavy constraints to produce the corresponding representative structure).*

Target system	Form	Experimental representative CIF name (in ranking list)	Original CSP structure (from phase one of the 7th blind test)
XXVII	A	XXVII_structure_28.cif,<br>XXVII_structure_38.cif,<br>XXVII_structure_59.cif,<br>XXVII_structure_61.cif	structure_21.cif (Group 20),<br>XXVII-1178.cif (Group 21),<br>structure_632.cif (Group 20),<br>structure_25.cif (Group 20)
XXVIII	A	XXVIII_structure_144.cif	structure_1.cif (Group 20)
XXXI	A (Major)	XXXI_structure_98.cif	BTXXXI_df1_1.cif (Group 24)
	A (Minor)	XXXI_structure_1.cif	VASP_MIN_3.cif (Group 1)
	B	XXXI_structure_25.cif	MI_0046_dft_3199da20.cif (Group 16)
	C	XXXI_structure_89.cif	-
XXXII	A (Major)	XXXII_structure_317.cif	structure_159.cif (Group 20)
	A (Minor)	N/A	-
	B (Low temperature)	XXXII_structure_232.cif	structure_72.cif (Group 20)
XXXIII	A	XXXIII_structure_233.cif	XXXIII-0011.cif (Group 21)
	B	XXXIII_structure_452.cif	structure_1.cif (Group 20)

Table 3: (Target XXVII) The CIF names of the CCDC-prepared structures provided to participants for the structure ranking exercise, the corresponding CIF sampled from the first phase (structure generation exercise) of the seventh blind test, and the results (number of molecules matched in a cluster of 30 and RMSD) from COMPACK comparisons (applying 25% and 25° distance/angle tolerances) between the two to assess the structural change resultant from constrained optimisations carried out by the organisers in the structure list preparation. (** indicates structures that match the core packing, excluding isopropyl groups, of experimental Form A of XXVII.) (Originating CIF names contain an added prefix of the form '{target}-{phase}-{group name/label}-')

CCDC-prepared CIF name	Originating CIF name	Molecules matched	RMSD (Å)
XXVII_structure_1.cif	XXVII-1-MNeumann-structure_876.cif	30	0.242
XXVII_structure_2.cif	XXVII-1-XtalPi-data_719_Z2_st_YUqI9l_5MQA_UF-Eu.cif	30	0.261
XXVII_structure_3.cif	XXVII-1-XtalPi-data_498_Z1_st_YT8AefePXwAwm-o8H.cif	30	0.264
XXVII_structure_4.cif	XXVII-1-SOHG-XXVII-0036.cif	30	0.298
XXVII_structure_5.cif	XXVII-1-GDay-27_XXVII_348_gopt-QR-2-3420-3.cif	30	0.202
XXVII_structure_6.cif	XXVII-1-SLPrice-BXXVII_i1mp_ab246.cif	30	0.561
XXVII_structure_7.cif	XXVII-1-XtalPi-data_382_Z1_st_YT8AefePXwAwm-oq5.cif	30	0.248
XXVII_structure_8.cif	XXVII-1-BEijck-{}data_vaneijck-XXVII.0512.cif	30	0.114
XXVII_structure_9.cif	XXVII-1-XtalPi-data_1220_Z1_st_YT8AefePXwAw-mo6O.cif	30	0.203
XXVII_structure_10.cif	XXVII-1-GDay-27_XXVII_366_gopt-QR-2-951-3.cif	30	0.498
XXVII_structure_11.cif	XXVII-1-XtalPi-data_318_Z1_st_YT8AefePXwAwm-oyZ.cif	30	0.212
XXVII_structure_12.cif	XXVII-1-MNeumann-structure_413.cif	30	0.210
XXVII_structure_13.cif	XXVII-1-XtalPi-data_524_Z1_st_YT8AefePXwAwm-opR.cif	30	0.199
XXVII_structure_14.cif	XXVII-1-CShang-XXVII_840.cif	30	0.128
XXVII_structure_15.cif	XXVII-1-GDay-27_XXVII_93_gopt-QR-2-16077-3.cif	30	0.115
XXVII_structure_16.cif	XXVII-1-XtalPi-data_266_Z2_st_YUqI9l_5MQA-_UFPT.cif	30	0.238
XXVII_structure_17.cif	XXVII-1-XtalPi-data_341_Z1_st_YT8AefePXwA-wmpDQ.cif	30	0.210
XXVII_structure_18.cif	XXVII-1-XtalPi-data_687_Z1_st_YT8AefePXwA-wmoox.cif	30	0.243
XXVII_structure_19.cif	XXVII-1-MNeumann-structure_792.cif	30	0.195
XXVII_structure_20.cif	XXVII-1-BEijck-{}data_vaneijck-XXVII.1426.cif	30	0.119
XXVII_structure_21.cif	XXVII-1-XtalPi-data_734_Z1_st_YT8AefePXwA-wmo4C.cif	30	0.259
XXVII_structure_22.cif	XXVII-1-SOHG-XXVII-0413.cif	30	0.293
XXVII_structure_23.cif	XXVII-1-MNeumann-structure_109.cif	30	0.214
XXVII_structure_24.cif	XXVII-1-XtalPi-data_1076_Z1_st_YT8AefePXw-Awmo7r.cif	30	0.265
XXVII_structure_25.cif	XXVII-1-BEijck-{}data_vaneijck-XXVII.1303.cif	30	0.103
XXVII_structure_26.cif	XXVII-1-GDay-27_XXVII_456_gopt-QR-2-4194-3.cif	30	0.143
XXVII_structure_27.cif	XXVII-1-GDay-27_XXVII_89_gopt-QR-15-29723-3.cif	30	0.118
XXVII_structure_28.cif	**XXVII-1-MNeumann-structure_21.cif	30	0.192
XXVII_structure_29.cif	XXVII-1-SOHG-XXVII-0906.cif	30	0.253
XXVII_structure_30.cif	XXVII-1-MNeumann-structure_264.cif	30	0.296
XXVII_structure_31.cif	XXVII-1-CShang-XXVII_370.cif	30	0.208
XXVII_structure_32.cif	XXVII-1-SOHG-XXVII-0998.cif	30	0.304
XXVII_structure_33.cif	XXVII-1-XtalPi-data_648_Z2_st_YUqI9l_5MQA-_UFPV.cif	30	0.234
XXVII_structure_34.cif	XXVII-1-MNeumann-structure_322.cif	30	0.229
XXVII_structure_35.cif	XXVII-1-SOHG-XXVII-0401.cif	30	0.282
XXVII_structure_36.cif	XXVII-1-QZhu-data_43-SG-4.cif	30	0.376
XXVII_structure_37.cif	XXVII-1-CShang-XXVII_1134.cif	30	0.133
XXVII_structure_38.cif	**XXVII-1-SOHG-XXVII-1178.cif	30	0.177
XXVII_structure_39.cif	XXVII-1-QZhu-data_1161-SG-7.cif	30	0.562

XXVII_structure_40.cif	XXVII-1-GDay-27_XXVII_89_gopt-QR-14-29216-3.cif	30	0.205
XXVII_structure_41.cif	XXVII-1-MNeumann-structure_689.cif	30	0.239
XXVII_structure_42.cif	XXVII-1-SOHG-XXVII-1089.cif	30	0.284
XXVII_structure_43.cif	XXVII-1-GDay-27_XXVII_531_gopt-QR-2-873-3.cif	30	0.152
XXVII_structure_44.cif	XXVII-1-QZhu-data_243-SG-14.cif	30	0.127
XXVII_structure_45.cif	XXVII-1-SOHG-XXVII-0667.cif	30	0.236
XXVII_structure_46.cif	XXVII-1-MNeumann-structure_300.cif	30	0.223
XXVII_structure_47.cif	XXVII-1-CShang-XXVII_1340.cif	30	0.180
XXVII_structure_48.cif	XXVII-1-BEijck-{}data_vaneijck-XXVII.1034.cif	30	0.112
XXVII_structure_49.cif	XXVII-1-GDay-27_XXVII_11_gopt-QR-2-16532-3.cif	30	0.146
XXVII_structure_50.cif	XXVII-1-BEijck-{}data_vaneijck-XXVII.0284.cif	30	0.150
XXVII_structure_51.cif	XXVII-1-AOganov-data_584.cif	30	0.489
XXVII_structure_52.cif	XXVII-1-MNeumann-structure_815.cif	30	0.348
XXVII_structure_53.cif	XXVII-1-BEijck-{}data_vaneijck-XXVII.0726.cif	30	0.075
XXVII_structure_54.cif	XXVII-1-MNeumann-structure_1445.cif	30	0.204
XXVII_structure_55.cif	XXVII-1-MNeumann-structure_281.cif	30	0.193
XXVII_structure_56.cif	XXVII-1-MNeumann-structure_911.cif	30	0.190
XXVII_structure_57.cif	XXVII-1-GDay-27_XXVII_89_gopt-QR-2-12169-3.cif	30	0.350
XXVII_structure_58.cif	XXVII-1-MNeumann-structure_7.cif	30	0.207
XXVII_structure_59.cif	**XXVII-1-MNeumann-structure_632.cif	30	0.149
XXVII_structure_60.cif	XXVII-1-MNeumann-structure_741.cif	30	0.237
XXVII_structure_61.cif	**XXVII-1-MNeumann-structure_25.cif	30	0.185
XXVII_structure_62.cif	XXVII-1-SOHG-XXVII-1335.cif	30	0.255
XXVII_structure_63.cif	XXVII-1-MNeumann-structure_744.cif	30	0.231
XXVII_structure_64.cif	XXVII-1-MNeumann-structure_633.cif	30	0.134
XXVII_structure_65.cif	XXVII-1-BEijck-{}data_vaneijck-XXVII.0227.cif	30	0.078
XXVII_structure_66.cif	XXVII-1-SOHG-XXVII-1344.cif	30	0.231
XXVII_structure_67.cif	XXVII-1-GDay-27_XXVII_531_gopt-QR-2-4094-3.cif	30	0.152
XXVII_structure_68.cif	XXVII-1-BEijck-{}data_vaneijck-XXVII.1070.cif	30	0.140
XXVII_structure_69.cif	XXVII-1-AOganov-data_1439.cif	30	0.209
XXVII_structure_70.cif	XXVII-1-MNeumann-structure_1356.cif	30	0.316
XXVII_structure_71.cif	XXVII-1-SOHG-XXVII-0047.cif	30	0.363
XXVII_structure_72.cif	XXVII-1-BEijck-{}data_vaneijck-XXVII.0407.cif	30	0.031
XXVII_structure_73.cif	XXVII-1-GDay-27_XXVII_592_gopt-QR-14-3619-3.cif	30	0.212
XXVII_structure_74.cif	XXVII-1-BEijck-{}data_vaneijck-XXVII.0628.cif	23	0.336
XXVII_structure_75.cif	XXVII-1-BEijck-{}data_vaneijck-XXVII.1194.cif	30	0.176
XXVII_structure_76.cif	XXVII-1-MNeumann-structure_400.cif	30	0.211
XXVII_structure_77.cif	XXVII-1-MNeumann-structure_722.cif	30	0.256
XXVII_structure_78.cif	XXVII-1-MNeumann-structure_1344.cif	30	0.206
XXVII_structure_79.cif	XXVII-1-CShang-XXVII_401.cif	30	0.147
XXVII_structure_80.cif	XXVII-1-CShang-XXVII_591.cif	30	0.238
XXVII_structure_81.cif	XXVII-1-CShang-XXVII_292.cif	30	0.122
XXVII_structure_82.cif	XXVII-1-CShang-XXVII_245.cif	30	0.201
XXVII_structure_83.cif	XXVII-1-BEijck-{}data_vaneijck-XXVII.0673.cif	30	0.100
XXVII_structure_84.cif	XXVII-1-MNeumann-structure_688.cif	30	0.224
XXVII_structure_85.cif	XXVII-1-BEijck-{}data_vaneijck-XXVII.0618.cif	30	0.115
XXVII_structure_86.cif	**XXVII-1-CShang-XXVII_1164.cif	30	0.129
XXVII_structure_87.cif	XXVII-1-XtalPi-data_1246_Z1_st_YT8AefePXwA-wmo34.cif	30	0.233
XXVII_structure_88.cif	XXVII-1-SOHG-XXVII-0724.cif	30	0.257
XXVII_structure_89.cif	XXVII-1-XtalPi-data_885_Z1_st_YT8AefePXwA-wmo3s.cif	30	0.212
XXVII_structure_90.cif	XXVII-1-MNeumann-structure_925.cif	30	0.186
XXVII_structure_91.cif	XXVII-1-GDay-27_XXVII_89_gopt-QR-9-5837-3.cif	30	0.157
XXVII_structure_92.cif	XXVII-1-BEijck-{}data_vaneijck-XXVII.0800.cif	30	0.098
XXVII_structure_93.cif	XXVII-1-SOHG-XXVII-0137.cif	30	0.272
XXVII_structure_94.cif	XXVII-1-SOHG-XXVII-0492.cif	30	0.164
XXVII_structure_95.cif	XXVII-1-SOHG-XXVII-0275.cif	30	0.237
XXVII_structure_96.cif	XXVII-1-BEijck-{}data_vaneijck-XXVII.0883.cif	30	0.105
XXVII_structure_97.cif	XXVII-1-BEijck-{}data_vaneijck-XXVII.0918.cif	30	0.165
XXVII_structure_98.cif	XXVII-1-CShang-XXVII_945.cif	30	0.161
XXVII_structure_99.cif	XXVII-1-MNeumann-structure_820.cif	30	0.226
XXVII_structure_100.cif	XXVII-1-CShang-XXVII_1446.cif	30	0.139

Table 4: (Target XXVIII) The CIF names of the CCDC-prepared structures provided to participants for the structure ranking exercise, the corresponding CIF sampled from the first phase (structure generation exercise) of the seventh blind test, and the results (number of molecules matched in a cluster of 30 and RMSD) from COMPACK comparisons (applying 25% and 25° distance/angle tolerances) between the two to assess the structural change resultant from constrained optimisations carried out by the organisers in the structure list preparation. (* indicates the structures representing experimental Form A of XXVIII.) (Originating CIF names contain an added prefix of the form '{target}-{phase}-{group name/label}-')

CCDC-prepared CIF name	Originating CIF name	Molecules matched	RMSD (Å)
XXVIII_structure_1.cif	XXVIII-1-SLPrice-XXVIII_dfFB63_FB63.cif	30	0.163
XXVIII_structure_2.cif	XXVIII-1-MNeumann-structure_810.cif	30	0.243
XXVIII_structure_3.cif	XXVIII-1-SLPrice-XXVIII_dfCC517_CC517.cif	30	0.280
XXVIII_structure_4.cif	XXVIII-1-SLPrice-XXVIII_dfCA77_CA77.cif	30	0.417
XXVIII_structure_5.cif	XXVIII-1-MNeumann-structure_538.cif	30	0.227
XXVIII_structure_6.cif	XXVIII-1-XtalPi-data_861_T1_st_YTl1-fW7BwAySpDM.cif	30	0.131
XXVIII_structure_7.cif	XXVIII-1-MNeumann-structure_1030.cif	30	0.226
XXVIII_structure_8.cif	XXVIII-1-MNeumann-structure_378.cif	30	0.246
XXVIII_structure_9.cif	XXVIII-1-SLPrice-XXVIII_dfFC492_FC492.cif	30	0.307
XXVIII_structure_10.cif	XXVIII-1-SLPrice-XXVIII_dfFA232_FA232.cif	30	0.222
XXVIII_structure_11.cif	XXVIII-1-MNeumann-structure_919.cif	30	0.258
XXVIII_structure_12.cif	XXVIII-1-SLPrice-XXVIII_dfFA566_FA566.cif	30	0.238
XXVIII_structure_13.cif	XXVIII-1-XtalPi-data_741_C1_st_YKRuUk3fRgAoi1EC.cif	30	0.409
XXVIII_structure_14.cif	XXVIII-1-SLPrice-XXVIII_dfFA1736_FA1736.cif	30	0.202
XXVIII_structure_15.cif	XXVIII-1-MNeumann-structure_305.cif	30	0.321
XXVIII_structure_16.cif	XXVIII-1-SLPrice-XXVIII_dfCC16_CC16.cif	30	0.265
XXVIII_structure_17.cif	XXVIII-1-SLPrice-XXVIII_dfFA966_FA966.cif	30	0.335
XXVIII_structure_18.cif	XXVIII-1-SLPrice-XXVIII_dfFA96_FA96.cif	30	0.208
XXVIII_structure_19.cif	XXVIII-1-XtalPi-data_842_T1_st_YTl1-fW7BwAySpDj.cif	30	0.229
XXVIII_structure_20.cif	XXVIII-1-SLPrice-XXVIII_dfCC282_CC282.cif	30	0.244
XXVIII_structure_21.cif	XXVIII-1-XtalPi-data_953_T1_st_YTl1-fW7BwAySpBn.cif	30	0.160
XXVIII_structure_22.cif	XXVIII-1-SLPrice-XXVIII_dfFA331_FA331.cif	30	0.255
XXVIII_structure_23.cif	XXVIII-1-XtalPi-data_997_T1_st_YTl1-fW7BwAySo4p.cif	30	0.243
XXVIII_structure_24.cif	XXVIII-1-SLPrice-XXVIII_dfFA71_FA71.cif	30	0.140
XXVIII_structure_25.cif	XXVIII-1-MNeumann-structure_1307.cif	30	0.221
XXVIII_structure_26.cif	XXVIII-1-CShang-XXVIII_572.cif	30	0.312
XXVIII_structure_27.cif	XXVIII-1-CShang-XXVIII_633.cif	30	0.288
XXVIII_structure_28.cif	XXVIII-1-XtalPi-data_458_T1_st_YTl1-fW7BwAySo5Z.cif	30	0.238
XXVIII_structure_29.cif	XXVIII-1-MNeumann-structure_48.cif	30	0.266
XXVIII_structure_30.cif	XXVIII-1-SLPrice-XXVIII_dfFA1969_FA1969.cif	30	0.223
XXVIII_structure_31.cif	XXVIII-1-MNeumann-structure_258.cif	30	0.419
XXVIII_structure_32.cif	XXVIII-1-SLPrice-XXVIII_dfFA1139_FA1139.cif	30	0.192
XXVIII_structure_33.cif	XXVIII-1-MNeumann-structure_403.cif	30	0.293
XXVIII_structure_34.cif	XXVIII-1-MNeumann-structure_1085.cif	30	0.420
XXVIII_structure_35.cif	XXVIII-1-CShang-XXVIII_1222.cif	30	0.267
XXVIII_structure_36.cif	XXVIII-1-SLPrice-XXVIII_dfCC173_CC173.cif	30	0.272
XXVIII_structure_37.cif	XXVIII-1-MNeumann-structure_878.cif	30	0.266
XXVIII_structure_38.cif	XXVIII-1-MNeumann-structure_1140.cif	30	0.169
XXVIII_structure_39.cif	XXVIII-1-MNeumann-structure_395.cif	30	0.357
XXVIII_structure_40.cif	XXVIII-1-MNeumann-structure_486.cif	30	0.251
XXVIII_structure_41.cif	XXVIII-1-MNeumann-structure_1495.cif	30	0.296
XXVIII_structure_42.cif	XXVIII-1-SLPrice-XXVIII_dfAA55_AA55.cif	30	0.151
XXVIII_structure_43.cif	XXVIII-1-XtalPi-data_504_T1_st_YTl1-fW7BwAySo31.cif	30	0.206
XXVIII_structure_44.cif	XXVIII-1-MNeumann-structure_844.cif	30	0.375
XXVIII_structure_45.cif	XXVIII-1-SLPrice-XXVIII_dfFA1110_FA1110.cif	30	0.219

XXVIII_structure_46.cif	XXVIII-1-SLPrice-XXVIII_dfFA2088_FA2088.cif	30	0.373
XXVIII_structure_47.cif	XXVIII-1-MNeumann-structure_456.cif	30	0.340
XXVIII_structure_48.cif	XXVIII-1-MNeumann-structure_1087.cif	30	0.527
XXVIII_structure_49.cif	XXVIII-1-MNeumann-structure_110.cif	30	0.370
XXVIII_structure_50.cif	XXVIII-1-MNeumann-structure_945.cif	30	0.296
XXVIII_structure_51.cif	XXVIII-1-SLPrice-XXVIII_dfCB209_CB209.cif	30	0.194
XXVIII_structure_52.cif	XXVIII-1-MNeumann-structure_875.cif	30	0.264
XXVIII_structure_53.cif	XXVIII-1-MNeumann-structure_1186.cif	30	0.247
XXVIII_structure_54.cif	XXVIII-1-CShang-XXVIII_375.cif	30	0.515
XXVIII_structure_55.cif	XXVIII-1-MNeumann-structure_501.cif	30	0.233
XXVIII_structure_56.cif	XXVIII-1-SLPrice-XXVIII_dfFB2313_FB2313.cif	30	0.327
XXVIII_structure_57.cif	XXVIII-1-MNeumann-structure_948.cif	30	0.290
XXVIII_structure_58.cif	XXVIII-1-MNeumann-structure_586.cif	30	0.240
XXVIII_structure_59.cif	XXVIII-1-SLPrice-XXVIII_dfCC767_CC767.cif	30	0.129
XXVIII_structure_60.cif	XXVIII-1-MNeumann-structure_1191.cif	30	0.222
XXVIII_structure_61.cif	XXVIII-1-MNeumann-structure_1438.cif	30	0.392
XXVIII_structure_62.cif	XXVIII-1-MNeumann-structure_186.cif	23	0.419
XXVIII_structure_63.cif	XXVIII-1-XtalPi-data_1494_T1_st_YTl1-fW7BwAySo9k.cif	30	0.143
XXVIII_structure_64.cif	XXVIII-1-XtalPi-data_177_T1_st_YK2w9oAjAAAnSf6h.cif	30	0.127
XXVIII_structure_65.cif	XXVIII-1-SLPrice-XXVIII_dfCC186_CC186.cif	30	0.222
XXVIII_structure_66.cif	XXVIII-1-MNeumann-structure_703.cif	30	0.250
XXVIII_structure_67.cif	XXVIII-1-MNeumann-structure_624.cif	30	0.282
XXVIII_structure_68.cif	XXVIII-1-MNeumann-structure_207.cif	30	0.340
XXVIII_structure_69.cif	XXVIII-1-MNeumann-structure_1396.cif	30	0.390
XXVIII_structure_70.cif	XXVIII-1-MNeumann-structure_1426.cif	30	0.302
XXVIII_structure_71.cif	XXVIII-1-DWMHofmann-00000002.cif	15	2.020
XXVIII_structure_72.cif	XXVIII-1-MNeumann-structure_748.cif	30	0.278
XXVIII_structure_73.cif	XXVIII-1-SLPrice-XXVIII_dfAA50_AA50.cif	30	0.175
XXVIII_structure_74.cif	XXVIII-1-MNeumann-structure_35.cif	30	0.388
XXVIII_structure_75.cif	XXVIII-1-SLPrice-XXVIII_dfFB131_FB131.cif	30	0.739
XXVIII_structure_76.cif	XXVIII-1-MNeumann-structure_1244.cif	30	0.305
XXVIII_structure_77.cif	XXVIII-1-MNeumann-structure_1477.cif	30	0.250
XXVIII_structure_78.cif	XXVIII-1-MNeumann-structure_247.cif	30	0.229
XXVIII_structure_79.cif	XXVIII-1-SLPrice-XXVIII_dfAA26_AA26.cif	30	0.169
XXVIII_structure_80.cif	XXVIII-1-XtalPi-data_961_T1_st_YTl1-fW7BwAySpAr.cif	30	0.175
XXVIII_structure_81.cif	XXVIII-1-SLPrice-XXVIII_dfFA659_FA659.cif	30	0.167
XXVIII_structure_82.cif	XXVIII-1-MNeumann-structure_574.cif	30	0.270
XXVIII_structure_83.cif	XXVIII-1-SLPrice-XXVIII_dfFA863_FA863.cif	30	0.194
XXVIII_structure_84.cif	XXVIII-1-MNeumann-structure_430.cif	30	0.314
XXVIII_structure_85.cif	XXVIII-1-CShang-XXVIII_1061.cif	30	0.275
XXVIII_structure_86.cif	XXVIII-1-MNeumann-structure_988.cif	30	0.338
XXVIII_structure_87.cif	XXVIII-1-SLPrice-XXVIII_dfCC276_CC276.cif	30	0.346
XXVIII_structure_88.cif	XXVIII-1-CShang-XXVIII_1078.cif	30	0.387
XXVIII_structure_89.cif	XXVIII-1-CShang-XXVIII_602.cif	30	0.508
XXVIII_structure_90.cif	XXVIII-1-SLPrice-XXVIII_dfAA45_AA45.cif	30	0.164
XXVIII_structure_91.cif	XXVIII-1-SLPrice-XXVIII_dfFA1374_FA1374.cif	30	0.423
XXVIII_structure_92.cif	XXVIII-1-SLPrice-XXVIII_dfFC193_FC193.cif	30	0.246
XXVIII_structure_93.cif	XXVIII-1-XtalPi-data_157_T1_st_YK2w9oAjAAAnSf7A.cif	30	0.200
XXVIII_structure_94.cif	XXVIII-1-MNeumann-structure_204.cif	30	0.281
XXVIII_structure_95.cif	XXVIII-1-MNeumann-structure_297.cif	30	0.297
XXVIII_structure_96.cif	XXVIII-1-SLPrice-XXVIII_dfFA2216_FA2216.cif	30	0.331
XXVIII_structure_97.cif	XXVIII-1-SLPrice-XXVIII_dfFA97_FA97.cif	30	0.187
XXVIII_structure_98.cif	XXVIII-1-MNeumann-structure_1422.cif	30	0.360
XXVIII_structure_99.cif	XXVIII-1-SLPrice-XXVIII_dfAC3_AC3.cif	30	0.158
XXVIII_structure_100.cif	XXVIII-1-XtalPi-data_666_T1_st_YTl1-fW7BwAySo4V.cif	30	0.405
XXVIII_structure_101.cif	XXVIII-1-MNeumann-structure_1194.cif	30	0.363
XXVIII_structure_102.cif	XXVIII-1-XtalPi-data_76_T1_st_YK2w9oAjAAAnSf51.cif	30	0.085
XXVIII_structure_103.cif	XXVIII-1-MNeumann-structure_392.cif	30	0.200
XXVIII_structure_104.cif	XXVIII-1-MNeumann-structure_522.cif	30	0.327
XXVIII_structure_105.cif	XXVIII-1-MNeumann-structure_221.cif	30	0.203
XXVIII_structure_106.cif	XXVIII-1-MNeumann-structure_71.cif	30	0.275
XXVIII_structure_107.cif	XXVIII-1-MNeumann-structure_1051.cif	30	0.312

XXVIII_structure_108.cif	XXVIII-1-SLPrice-XXVIII_dfFA38_FA38.cif	30	0.102
XXVIII_structure_109.cif	XXVIII-1-XtalPi-data_750_T1_st_YTl1-fW7BwAySpAw.cif	30	0.059
XXVIII_structure_110.cif	XXVIII-1-SLPrice-XXVIII_dfFC48_FC48.cif	30	0.478
XXVIII_structure_111.cif	XXVIII-1-XtalPi-data_222_T1_st_YTl1-fW7BwAySpA0.cif	30	0.249
XXVIII_structure_112.cif	XXVIII-1-MNeumann-structure_1318.cif	30	0.288
XXVIII_structure_113.cif	XXVIII-1-MNeumann-structure_352.cif	30	0.395
XXVIII_structure_114.cif	XXVIII-1-MNeumann-structure_536.cif	30	0.262
XXVIII_structure_115.cif	XXVIII-1-XtalPi-data_966_C1_st_YTl0Nsor_gAwR_Wd.cif	30	0.129
XXVIII_structure_116.cif	XXVIII-1-MNeumann-structure_78.cif	30	0.298
XXVIII_structure_117.cif	XXVIII-1-SLPrice-XXVIII_dfFA2_FA2.cif	30	0.185
XXVIII_structure_118.cif	XXVIII-1-SLPrice-XXVIII_dfCA18_CA18.cif	30	0.326
XXVIII_structure_119.cif	XXVIII-1-MNeumann-structure_564.cif	30	0.303
XXVIII_structure_120.cif	XXVIII-1-SLPrice-XXVIII_dfFA1054_FA1054.cif	30	0.237
XXVIII_structure_121.cif	XXVIII-1-MNeumann-structure_227.cif	30	0.342
XXVIII_structure_122.cif	XXVIII-1-SLPrice-XXVIII_dfFA28_FA28.cif	30	0.266
XXVIII_structure_123.cif	XXVIII-1-CShang-XXVIII_1128.cif	30	0.384
XXVIII_structure_124.cif	XXVIII-1-SLPrice-XXVIII_dfFA1106_FA1106.cif	30	0.490
XXVIII_structure_125.cif	XXVIII-1-SLPrice-XXVIII_dfAC6_AC6.cif	30	0.299
XXVIII_structure_126.cif	XXVIII-1-MNeumann-structure_353.cif	30	0.414
XXVIII_structure_127.cif	XXVIII-1-SLPrice-XXVIII_dfFA59_FA59.cif	30	0.127
XXVIII_structure_128.cif	XXVIII-1-MNeumann-structure_1198.cif	30	0.329
XXVIII_structure_129.cif	XXVIII-1-SLPrice-XXVIII_dfCB1046_CB1046.cif	30	0.259
XXVIII_structure_130.cif	XXVIII-1-SLPrice-XXVIII_dfFA35_FA35.cif	30	0.100
XXVIII_structure_131.cif	XXVIII-1-MNeumann-structure_493.cif	30	0.315
XXVIII_structure_132.cif	XXVIII-1-MNeumann-structure_1311.cif	30	0.344
XXVIII_structure_133.cif	XXVIII-1-MNeumann-structure_64.cif	30	0.317
XXVIII_structure_134.cif	XXVIII-1-MNeumann-structure_283.cif	30	0.228
XXVIII_structure_135.cif	XXVIII-1-CShang-XXVIII_417.cif	30	0.448
XXVIII_structure_136.cif	XXVIII-1-SLPrice-XXVIII_dfCC564_CC564.cif	30	0.405
XXVIII_structure_137.cif	XXVIII-1-MNeumann-structure_135.cif	30	0.494
XXVIII_structure_138.cif	XXVIII-1-SLPrice-XXVIII_dfFC362_FC362.cif	30	0.378
XXVIII_structure_139.cif	XXVIII-1-MNeumann-structure_1205.cif	30	0.330
XXVIII_structure_140.cif	XXVIII-1-MNeumann-structure_663.cif	30	0.268
XXVIII_structure_141.cif	XXVIII-1-SLPrice-XXVIII_dfFA273_FA273.cif	30	0.187
XXVIII_structure_142.cif	XXVIII-1-MNeumann-structure_1462.cif	30	0.329
XXVIII_structure_143.cif	XXVIII-1-XtalPi-data_737_C1_st_YTl0Nsor_gAwR_Uo.cif	30	0.228
XXVIII_structure_144.cif	*XXVIII-1-MNeumann-structure_1.cif	30	0.301
XXVIII_structure_145.cif	XXVIII-1-CShang-XXVIII_1192.cif	30	0.522
XXVIII_structure_146.cif	XXVIII-1-CShang-XXVIII_554.cif	30	0.656
XXVIII_structure_147.cif	XXVIII-1-MNeumann-structure_679.cif	30	0.285
XXVIII_structure_148.cif	XXVIII-1-MNeumann-structure_1053.cif	30	0.286
XXVIII_structure_149.cif	XXVIII-1-MNeumann-structure_1206.cif	30	0.301
XXVIII_structure_150.cif	XXVIII-1-SLPrice-XXVIII_dfFA159_FA159.cif	30	0.231
XXVIII_structure_151.cif	XXVIII-1-XtalPi-data_1078_T1_st_YTl1-fW7BwAySo3J.cif	30	0.471
XXVIII_structure_152.cif	XXVIII-1-MNeumann-structure_254.cif	30	0.267
XXVIII_structure_153.cif	XXVIII-1-MNeumann-structure_1418.cif	30	0.790
XXVIII_structure_154.cif	XXVIII-1-XtalPi-data_719_T1_st_YK2w9oAjAAAnSf7K.cif	30	0.208
XXVIII_structure_155.cif	XXVIII-1-SLPrice-XXVIII_dfFB136_FB136.cif	30	0.202
XXVIII_structure_156.cif	XXVIII-1-MNeumann-structure_123.cif	30	0.450
XXVIII_structure_157.cif	XXVIII-1-MNeumann-structure_764.cif	30	0.339
XXVIII_structure_158.cif	XXVIII-1-SLPrice-XXVIII_dfFA1478_FA1478.cif	30	0.174
XXVIII_structure_159.cif	XXVIII-1-MNeumann-structure_1119.cif	30	0.204
XXVIII_structure_160.cif	XXVIII-1-MNeumann-structure_275.cif	30	0.445
XXVIII_structure_161.cif	XXVIII-1-SLPrice-XXVIII_dfFB334_FB334.cif	30	0.323
XXVIII_structure_162.cif	XXVIII-1-SLPrice-XXVIII_dfAA40_AA40.cif	30	0.208
XXVIII_structure_163.cif	XXVIII-1-MNeumann-structure_359.cif	30	0.515
XXVIII_structure_164.cif	XXVIII-1-SLPrice-XXVIII_dfFA158_FA158.cif	30	0.193
XXVIII_structure_165.cif	XXVIII-1-MNeumann-structure_1202.cif	30	0.536
XXVIII_structure_166.cif	XXVIII-1-MNeumann-structure_410.cif	30	0.376
XXVIII_structure_167.cif	XXVIII-1-SLPrice-XXVIII_dfFA2243_FA2243.cif	30	0.471
XXVIII_structure_168.cif	XXVIII-1-SLPrice-XXVIII_dfAC228_AC228.cif	30	0.210
XXVIII_structure_169.cif	XXVIII-1-SLPrice-XXVIII_dfFA204_FA204.cif	30	0.180

XXVIII_structure_170.cif	XXVIII-1-SLPrice-XXVIII_dfFA170_FA170.cif	30	0.154
XXVIII_structure_171.cif	XXVIII-1-CShang-XXVIII_199.cif	30	0.401
XXVIII_structure_172.cif	XXVIII-1-SLPrice-XXVIII_dfAA63_AA63.cif	30	0.394
XXVIII_structure_173.cif	XXVIII-1-XtalPi-data_1285_T1_st_YTl1-fW7BwAySo8N.cif	30	0.309
XXVIII_structure_174.cif	XXVIII-1-SLPrice-XXVIII_dfFA766_FA766.cif	30	0.159
XXVIII_structure_175.cif	XXVIII-1-XtalPi-data_730_T1_st_YTl1-fW7BwAySo8J.cif	30	0.191
XXVIII_structure_176.cif	XXVIII-1-SLPrice-XXVIII_dfCA47_CA47.cif	30	0.249
XXVIII_structure_177.cif	XXVIII-1-MNeumann-structure_459.cif	30	0.284
XXVIII_structure_178.cif	XXVIII-1-CShang-XXVIII_79.cif	30	0.299
XXVIII_structure_179.cif	XXVIII-1-MNeumann-structure_1381.cif	30	0.275
XXVIII_structure_180.cif	XXVIII-1-MNeumann-structure_464.cif	30	0.298
XXVIII_structure_181.cif	XXVIII-1-MNeumann-structure_1479.cif	30	0.204
XXVIII_structure_182.cif	XXVIII-1-SLPrice-XXVIII_dfFA422_FA422.cif	30	0.228
XXVIII_structure_183.cif	XXVIII-1-SLPrice-XXVIII_dfCA809_CA809.cif	30	0.432
XXVIII_structure_184.cif	XXVIII-1-SLPrice-XXVIII_dfFA196_FA196.cif	30	0.244
XXVIII_structure_185.cif	XXVIII-1-MNeumann-structure_1420.cif	30	0.275
XXVIII_structure_186.cif	XXVIII-1-MNeumann-structure_594.cif	30	0.337
XXVIII_structure_187.cif	XXVIII-1-MNeumann-structure_504.cif	30	0.438
XXVIII_structure_188.cif	XXVIII-1-SLPrice-XXVIII_dfFA1567_FA1567.cif	30	0.276
XXVIII_structure_189.cif	XXVIII-1-SLPrice-XXVIII_dfCB971_CB971.cif	30	0.533
XXVIII_structure_190.cif	XXVIII-1-SLPrice-XXVIII_dfCC617_CC617.cif	30	0.294
XXVIII_structure_191.cif	XXVIII-1-XtalPi-data_249_T1_st_YTl1-fW7BwAySpBs.cif	30	0.183
XXVIII_structure_192.cif	XXVIII-1-SLPrice-XXVIII_dfBA58_BA58.cif	30	0.305
XXVIII_structure_193.cif	XXVIII-1-CShang-XXVIII_1060.cif	30	0.729
XXVIII_structure_194.cif	XXVIII-1-MNeumann-structure_60.cif	30	0.314
XXVIII_structure_195.cif	XXVIII-1-MNeumann-structure_693.cif	30	0.215
XXVIII_structure_196.cif	XXVIII-1-XtalPi-data_329_T1_st_YK2w9oAjAAAnSf4n.cif	30	0.275
XXVIII_structure_197.cif	XXVIII-1-MNeumann-structure_118.cif	30	0.243
XXVIII_structure_198.cif	XXVIII-1-SLPrice-XXVIII_dfCA103_CA103.cif	30	0.403
XXVIII_structure_199.cif	XXVIII-1-SLPrice-XXVIII_dfCC259_CC259.cif	30	0.257
XXVIII_structure_200.cif	XXVIII-1-XtalPi-data_858_T1_st_YTl1-fW7BwAySo6I.cif	30	0.209
XXVIII_structure_201.cif	XXVIII-1-MNeumann-structure_83.cif	30	0.369
XXVIII_structure_202.cif	XXVIII-1-SLPrice-XXVIII_dfCC428_CC428.cif	30	0.165
XXVIII_structure_203.cif	XXVIII-1-MNeumann-structure_578.cif	30	0.351
XXVIII_structure_204.cif	XXVIII-1-XtalPi-data_435_C1_st_YTl0Nsor_gAwR_S3.cif	30	0.174
XXVIII_structure_205.cif	XXVIII-1-CShang-XXVIII_1466.cif	30	0.492
XXVIII_structure_206.cif	XXVIII-1-XtalPi-data_357_T1_st_YTl1-fW7BwAySo2y.cif	30	0.429
XXVIII_structure_207.cif	XXVIII-1-CShang-XXVIII_675.cif	30	0.421
XXVIII_structure_208.cif	XXVIII-1-SLPrice-XXVIII_dfBC9_BC9.cif	30	0.162
XXVIII_structure_209.cif	XXVIII-1-DWMHofmann-00000028.cif	30	0.674
XXVIII_structure_210.cif	XXVIII-1-XtalPi-data_948_T1_st_YTl1-fW7BwAySpBC.cif	30	0.137
XXVIII_structure_211.cif	XXVIII-1-DWMHofmann-00000006.cif	30	1.215
XXVIII_structure_212.cif	XXVIII-1-CShang-XXVIII_209.cif	30	0.757
XXVIII_structure_213.cif	XXVIII-1-DWMHofmann-00000017.cif	30	1.017
XXVIII_structure_214.cif	XXVIII-1-MNeumann-structure_691.cif	30	0.298
XXVIII_structure_215.cif	XXVIII-1-SLPrice-XXVIII_dfFA858_FA858.cif	30	0.270
XXVIII_structure_216.cif	XXVIII-1-MNeumann-structure_863.cif	30	0.244
XXVIII_structure_217.cif	XXVIII-1-SLPrice-XXVIII_dfCA31_CA31.cif	30	0.179
XXVIII_structure_218.cif	XXVIII-1-XtalPi-data_125_T1_st_YTl1-fW7BwAySo5r.cif	30	0.598
XXVIII_structure_219.cif	XXVIII-1-MNeumann-structure_1304.cif	30	0.281
XXVIII_structure_220.cif	XXVIII-1-MNeumann-structure_74.cif	30	0.331
XXVIII_structure_221.cif	XXVIII-1-MNeumann-structure_565.cif	30	0.284
XXVIII_structure_222.cif	XXVIII-1-SLPrice-XXVIII_dfFA83_FA83.cif	30	0.105
XXVIII_structure_223.cif	XXVIII-1-MNeumann-structure_1212.cif	30	0.311
XXVIII_structure_224.cif	XXVIII-1-MNeumann-structure_817.cif	30	0.345
XXVIII_structure_225.cif	XXVIII-1-MNeumann-structure_1353.cif	30	0.350
XXVIII_structure_226.cif	XXVIII-1-MNeumann-structure_1339.cif	30	0.298
XXVIII_structure_227.cif	XXVIII-1-SLPrice-XXVIII_dfCA362_CA362.cif	30	0.335
XXVIII_structure_228.cif	XXVIII-1-MNeumann-structure_1150.cif	30	0.293
XXVIII_structure_229.cif	XXVIII-1-MNeumann-structure_467.cif	30	0.250
XXVIII_structure_230.cif	XXVIII-1-MNeumann-structure_675.cif	30	0.267

XXVIII_structure_231.cif	XXVIII-1-MNeumann-structure_1344.cif	30	0.411
XXVIII_structure_232.cif	XXVIII-1-XtalPi-data_1143_T1_st_YTl1-fW7BwAySo-t.cif	30	0.117
XXVIII_structure_233.cif	XXVIII-1-SLPrice-XXVIII_dfFA200_FA200.cif	30	0.187
XXVIII_structure_234.cif	XXVIII-1-SLPrice-XXVIII_dfAA572_AA572.cif	30	0.154
XXVIII_structure_235.cif	XXVIII-1-SLPrice-XXVIII_dfFA410_FA410.cif	30	0.044
XXVIII_structure_236.cif	XXVIII-1-MNeumann-structure_387.cif	30	0.231
XXVIII_structure_237.cif	XXVIII-1-MNeumann-structure_288.cif	30	0.464
XXVIII_structure_238.cif	XXVIII-1-MNeumann-structure_1432.cif	30	0.320
XXVIII_structure_239.cif	XXVIII-1-XtalPi-data_556_T1_st_YTl1-fW7BwAySo89.cif	30	0.578
XXVIII_structure_240.cif	XXVIII-1-SLPrice-XXVIII_dfCB446_CB446.cif	30	0.371
XXVIII_structure_241.cif	XXVIII-1-MNeumann-structure_1129.cif	30	0.331
XXVIII_structure_242.cif	XXVIII-1-MNeumann-structure_1373.cif	30	0.340
XXVIII_structure_243.cif	XXVIII-1-CShang-XXVIII_379.cif	30	0.457
XXVIII_structure_244.cif	XXVIII-1-MNeumann-structure_429.cif	30	0.380
XXVIII_structure_245.cif	XXVIII-1-SLPrice-XXVIII_dfFA792_FA792.cif	30	0.291
XXVIII_structure_246.cif	XXVIII-1-SLPrice-XXVIII_dfFC136_FC136.cif	30	0.232
XXVIII_structure_247.cif	XXVIII-1-CShang-XXVIII_844.cif	30	0.300
XXVIII_structure_248.cif	XXVIII-1-MNeumann-structure_1419.cif	30	0.278
XXVIII_structure_249.cif	XXVIII-1-MNeumann-structure_29.cif	30	0.375
XXVIII_structure_250.cif	XXVIII-1-MNeumann-structure_1279.cif	30	0.212
XXVIII_structure_251.cif	XXVIII-1-SLPrice-XXVIII_dfFA796_FA796.cif	30	0.247
XXVIII_structure_252.cif	XXVIII-1-MNeumann-structure_57.cif	30	0.329
XXVIII_structure_253.cif	XXVIII-1-MNeumann-structure_835.cif	30	0.331
XXVIII_structure_254.cif	XXVIII-1-XtalPi-data_756_T1_st_YTl1-fW7BwAySpCN.cif	30	0.217
XXVIII_structure_255.cif	XXVIII-1-CShang-XXVIII_892.cif	30	0.396
XXVIII_structure_256.cif	XXVIII-1-MNeumann-structure_972.cif	30	0.261
XXVIII_structure_257.cif	XXVIII-1-SLPrice-XXVIII_dfFA519_FA519.cif	30	0.158
XXVIII_structure_258.cif	XXVIII-1-XtalPi-data_1263_T1_st_YTl1-fW7BwAySpCf.cif	30	0.195
XXVIII_structure_259.cif	XXVIII-1-SLPrice-XXVIII_dfCC181_CC181.cif	30	0.309
XXVIII_structure_260.cif	XXVIII-1-SLPrice-XXVIII_dfCC288_CC288.cif	30	0.163
XXVIII_structure_261.cif	XXVIII-1-MNeumann-structure_100.cif	30	0.380
XXVIII_structure_262.cif	XXVIII-1-XtalPi-data_181_T1_st_YK2w9oAjAAAnSf4w.cif	30	0.225
XXVIII_structure_263.cif	XXVIII-1-SLPrice-XXVIII_dfFA943_FA943.cif	30	0.380
XXVIII_structure_264.cif	XXVIII-1-MNeumann-structure_1166.cif	30	0.384
XXVIII_structure_265.cif	XXVIII-1-XtalPi-data_871_C1_st_YTl0Nsor_gAwR_Vt.cif	30	0.129
XXVIII_structure_266.cif	XXVIII-1-SLPrice-XXVIII_dfFC114_FC114.cif	30	0.120
XXVIII_structure_267.cif	XXVIII-1-SLPrice-XXVIII_dfFB3_FB3.cif	30	0.349
XXVIII_structure_268.cif	XXVIII-1-MNeumann-structure_1428.cif	30	0.283
XXVIII_structure_269.cif	XXVIII-1-SLPrice-XXVIII_dfFA518_FA518.cif	30	0.284
XXVIII_structure_270.cif	XXVIII-1-SLPrice-XXVIII_dfFB65_FB65.cif	30	0.361
XXVIII_structure_271.cif	XXVIII-1-SLPrice-XXVIII_dfCA83_CA83.cif	30	0.332
XXVIII_structure_272.cif	XXVIII-1-MNeumann-structure_477.cif	30	0.364
XXVIII_structure_273.cif	XXVIII-1-XtalPi-data_137_T1_st_YTl1-fW7BwAySo5L.cif	30	0.299
XXVIII_structure_274.cif	XXVIII-1-MNeumann-structure_911.cif	30	0.233
XXVIII_structure_275.cif	XXVIII-1-MNeumann-structure_1047.cif	30	0.378
XXVIII_structure_276.cif	XXVIII-1-MNeumann-structure_1092.cif	30	0.499
XXVIII_structure_277.cif	XXVIII-1-MNeumann-structure_803.cif	30	0.254
XXVIII_structure_278.cif	XXVIII-1-MNeumann-structure_592.cif	30	0.290
XXVIII_structure_279.cif	XXVIII-1-MNeumann-structure_1491.cif	30	0.097
XXVIII_structure_280.cif	XXVIII-1-XtalPi-data_1393_C1_st_YKRuUk3fRgAoi1Dk.cif	30	0.142
XXVIII_structure_281.cif	XXVIII-1-MNeumann-structure_368.cif	30	0.300
XXVIII_structure_282.cif	XXVIII-1-SLPrice-XXVIII_dfAA1855_AA1855.cif	30	0.275
XXVIII_structure_283.cif	XXVIII-1-SLPrice-XXVIII_dfFC315_FC315.cif	30	0.153
XXVIII_structure_284.cif	XXVIII-1-SLPrice-XXVIII_dfFA1970_FA1970.cif	30	0.144
XXVIII_structure_285.cif	XXVIII-1-SLPrice-XXVIII_dfFB481_FB481.cif	30	0.205
XXVIII_structure_286.cif	XXVIII-1-XtalPi-data_416_C1_st_YTl0Nsor_gAwR_VA.cif	30	0.224
XXVIII_structure_287.cif	XXVIII-1-MNeumann-structure_1050.cif	30	0.263
XXVIII_structure_288.cif	XXVIII-1-SLPrice-XXVIII_dfAC55_AC55.cif	30	0.251
XXVIII_structure_289.cif	XXVIII-1-MNeumann-structure_192.cif	30	0.255
XXVIII_structure_290.cif	XXVIII-1-SLPrice-XXVIII_dfFC324_FC324.cif	30	0.188

XXVIII_structure_291.cif	XXVIII-1-SLPrice-XXVIII_dfCA251_CA251.cif	30	0.403
XXVIII_structure_292.cif	XXVIII-1-SLPrice-XXVIII_dfFB183_FB183.cif	30	0.276
XXVIII_structure_293.cif	XXVIII-1-SLPrice-XXVIII_dfFB435_FB435.cif	30	0.153
XXVIII_structure_294.cif	XXVIII-1-MNeumann-structure_650.cif	30	0.371
XXVIII_structure_295.cif	XXVIII-1-XtalPi-data_762_C1_st_YTl0Nsor_gAwR_Sb.cif	30	0.120
XXVIII_structure_296.cif	XXVIII-1-DWMHofmann-00000025.cif	14	1.417
XXVIII_structure_297.cif	XXVIII-1-SLPrice-XXVIII_dfFA4_FA4.cif	30	0.265
XXVIII_structure_298.cif	XXVIII-1-MNeumann-structure_709.cif	30	0.184
XXVIII_structure_299.cif	XXVIII-1-MNeumann-structure_452.cif	30	0.259
XXVIII_structure_300.cif	XXVIII-1-MNeumann-structure_442.cif	30	0.307
XXVIII_structure_301.cif	XXVIII-1-MNeumann-structure_1055.cif	30	0.410
XXVIII_structure_302.cif	XXVIII-1-MNeumann-structure_1320.cif	30	0.252
XXVIII_structure_303.cif	XXVIII-1-SLPrice-XXVIII_dfFA1912_FA1912.cif	30	0.167
XXVIII_structure_304.cif	XXVIII-1-MNeumann-structure_1019.cif	30	0.416
XXVIII_structure_305.cif	XXVIII-1-XtalPi-data_930_T1_st_YTl1-fW7BwAySpCv.cif	30	0.148
XXVIII_structure_306.cif	XXVIII-1-SLPrice-XXVIII_dfCA3_CA3.cif	30	0.354
XXVIII_structure_307.cif	XXVIII-1-XtalPi-data_57_T1_st_YTl1-fW7BwAySo7c.cif	30	0.105
XXVIII_structure_308.cif	XXVIII-1-MNeumann-structure_9.cif	30	0.490
XXVIII_structure_309.cif	XXVIII-1-SLPrice-XXVIII_dfFA1490_FA1490.cif	30	0.156
XXVIII_structure_310.cif	XXVIII-1-SLPrice-XXVIII_dfFA793_FA793.cif	30	0.323
XXVIII_structure_311.cif	XXVIII-1-SLPrice-XXVIII_dfFA873_FA873.cif	30	0.514
XXVIII_structure_312.cif	XXVIII-1-MNeumann-structure_612.cif	30	0.312
XXVIII_structure_313.cif	XXVIII-1-MNeumann-structure_829.cif	30	0.286
XXVIII_structure_314.cif	XXVIII-1-XtalPi-data_205_C1_st_YTl0Nsor_gAwR_Ua.cif	30	0.205
XXVIII_structure_315.cif	XXVIII-1-MNeumann-structure_648.cif	30	0.300
XXVIII_structure_316.cif	XXVIII-1-MNeumann-structure_1456.cif	30	0.252
XXVIII_structure_317.cif	XXVIII-1-MNeumann-structure_644.cif	30	0.299
XXVIII_structure_318.cif	XXVIII-1-MNeumann-structure_604.cif	30	0.265
XXVIII_structure_319.cif	XXVIII-1-SLPrice-XXVIII_dfCB344_CB344.cif	30	0.297
XXVIII_structure_320.cif	XXVIII-1-SLPrice-XXVIII_dfCB453_CB453.cif	30	0.438
XXVIII_structure_321.cif	XXVIII-1-MNeumann-structure_164.cif	30	0.554
XXVIII_structure_322.cif	XXVIII-1-MNeumann-structure_1114.cif	30	0.218
XXVIII_structure_323.cif	XXVIII-1-SLPrice-XXVIII_dfFA321_FA321.cif	30	0.375
XXVIII_structure_324.cif	XXVIII-1-SLPrice-XXVIII_dfCC352_CC352.cif	30	0.189
XXVIII_structure_325.cif	XXVIII-1-MNeumann-structure_1312.cif	30	0.333
XXVIII_structure_326.cif	XXVIII-1-XtalPi-data_127_T1_st_YK2w9oAjAAAnSf7Z.cif	30	0.110
XXVIII_structure_327.cif	XXVIII-1-SLPrice-XXVIII_dfCB104_CB104.cif	30	0.253
XXVIII_structure_328.cif	XXVIII-1-MNeumann-structure_1089.cif	30	0.253
XXVIII_structure_329.cif	XXVIII-1-SLPrice-XXVIII_dfCB397_CB397.cif	30	0.320
XXVIII_structure_330.cif	XXVIII-1-MNeumann-structure_289.cif	30	0.300
XXVIII_structure_331.cif	XXVIII-1-XtalPi-data_1132_T1_st_YTl1-fW7BwAySo4E.cif	30	0.172
XXVIII_structure_332.cif	XXVIII-1-MNeumann-structure_1278.cif	30	0.324
XXVIII_structure_333.cif	XXVIII-1-XtalPi-data_287_T1_st_YK2w9oAjAAAnSf5Q.cif	30	0.556
XXVIII_structure_334.cif	XXVIII-1-MNeumann-structure_277.cif	30	0.299
XXVIII_structure_335.cif	XXVIII-1-SLPrice-XXVIII_dfFA511_FA511.cif	30	0.161
XXVIII_structure_336.cif	XXVIII-1-SLPrice-XXVIII_dfFA152_FA152.cif	30	0.141
XXVIII_structure_337.cif	XXVIII-1-MNeumann-structure_49.cif	30	0.442
XXVIII_structure_338.cif	XXVIII-1-XtalPi-data_230_C1_st_YTl0Nsor_gAwR_Vk.cif	30	0.113
XXVIII_structure_339.cif	XXVIII-1-SLPrice-XXVIII_dfCC400_CC400.cif	30	0.401
XXVIII_structure_340.cif	XXVIII-1-MNeumann-structure_674.cif	30	0.263
XXVIII_structure_341.cif	XXVIII-1-MNeumann-structure_363.cif	30	0.228
XXVIII_structure_342.cif	XXVIII-1-MNeumann-structure_721.cif	30	0.280
XXVIII_structure_343.cif	XXVIII-1-SLPrice-XXVIII_dfFA869_FA869.cif	30	0.207
XXVIII_structure_344.cif	XXVIII-1-MNeumann-structure_823.cif	30	0.244
XXVIII_structure_345.cif	XXVIII-1-MNeumann-structure_249.cif	30	0.240
XXVIII_structure_346.cif	XXVIII-1-MNeumann-structure_747.cif	30	0.378
XXVIII_structure_347.cif	XXVIII-1-XtalPi-data_566_T1_st_YTl1-fW7BwAySo81.cif	30	0.214
XXVIII_structure_348.cif	XXVIII-1-MNeumann-structure_371.cif	30	0.329
XXVIII_structure_349.cif	XXVIII-1-SLPrice-XXVIII_dfFB103_FB103.cif	30	0.325
XXVIII_structure_350.cif	XXVIII-1-MNeumann-structure_807.cif	30	0.402
XXVIII_structure_351.cif	XXVIII-1-XtalPi-data_144_C1_st_YKRuUk3fRgAoi1Ds.cif	30	0.228
XXVIII_structure_352.cif	XXVIII-1-MNeumann-structure_1401.cif	30	0.261

XXVIII_structure_353.cif	XXVIII-1-SLPrice-XXVIII_dfFA1579_FA1579.cif	30	0.187
XXVIII_structure_354.cif	XXVIII-1-SLPrice-XXVIII_dfBA51_BA51.cif	30	0.552
XXVIII_structure_355.cif	XXVIII-1-XtalPi-data_1347_T1_st_YTl1-fW7BwAySo2N.cif	30	0.320
XXVIII_structure_356.cif	XXVIII-1-MNeumann-structure_1260.cif	30	0.267
XXVIII_structure_357.cif	XXVIII-1-MNeumann-structure_271.cif	30	0.212
XXVIII_structure_358.cif	XXVIII-1-XtalPi-data_201_T1_st_YK2w9oAjAAAnSf6x.cif	30	0.119
XXVIII_structure_359.cif	XXVIII-1-MNeumann-structure_1488.cif	30	0.405
XXVIII_structure_360.cif	XXVIII-1-MNeumann-structure_737.cif	30	0.332
XXVIII_structure_361.cif	XXVIII-1-SLPrice-XXVIII_dfCB576_CB576.cif	30	0.247
XXVIII_structure_362.cif	XXVIII-1-SLPrice-XXVIII_dfFC43_FC43.cif	30	0.287
XXVIII_structure_363.cif	XXVIII-1-MNeumann-structure_884.cif	30	0.361
XXVIII_structure_364.cif	XXVIII-1-MNeumann-structure_1146.cif	30	0.268
XXVIII_structure_365.cif	XXVIII-1-SLPrice-XXVIII_dfFB167_FB167.cif	30	0.211
XXVIII_structure_366.cif	XXVIII-1-XtalPi-data_1271_T1_st_YTl1-fW7BwAySpCH.cif	30	0.448
XXVIII_structure_367.cif	XXVIII-1-SLPrice-XXVIII_dfCC411_CC411.cif	30	0.396
XXVIII_structure_368.cif	XXVIII-1-MNeumann-structure_815.cif	30	0.278
XXVIII_structure_369.cif	XXVIII-1-MNeumann-structure_587.cif	30	0.302
XXVIII_structure_370.cif	XXVIII-1-MNeumann-structure_1139.cif	30	0.245
XXVIII_structure_371.cif	XXVIII-1-MNeumann-structure_1251.cif	30	0.267
XXVIII_structure_372.cif	XXVIII-1-CShang-XXVIII_693.cif	30	0.281
XXVIII_structure_373.cif	XXVIII-1-MNeumann-structure_971.cif	30	0.280
XXVIII_structure_374.cif	XXVIII-1-MNeumann-structure_499.cif	30	0.369
XXVIII_structure_375.cif	XXVIII-1-SLPrice-XXVIII_dfFA1225_FA1225.cif	30	0.306
XXVIII_structure_376.cif	XXVIII-1-MNeumann-structure_856.cif	30	0.382
XXVIII_structure_377.cif	XXVIII-1-SLPrice-XXVIII_dfFB178_FB178.cif	30	0.139
XXVIII_structure_378.cif	XXVIII-1-MNeumann-structure_318.cif	30	0.254
XXVIII_structure_379.cif	XXVIII-1-SLPrice-XXVIII_dfFA643_FA643.cif	30	0.263
XXVIII_structure_380.cif	XXVIII-1-SLPrice-XXVIII_dfFB14_FB14.cif	30	0.229
XXVIII_structure_381.cif	XXVIII-1-MNeumann-structure_698.cif	30	0.334
XXVIII_structure_382.cif	XXVIII-1-MNeumann-structure_492.cif	30	0.350
XXVIII_structure_383.cif	XXVIII-1-MNeumann-structure_143.cif	30	0.354
XXVIII_structure_384.cif	XXVIII-1-CShang-XXVIII_316.cif	30	0.486
XXVIII_structure_385.cif	XXVIII-1-MNeumann-structure_661.cif	30	0.428
XXVIII_structure_386.cif	XXVIII-1-SLPrice-XXVIII_dfFA355_FA355.cif	30	0.360
XXVIII_structure_387.cif	XXVIII-1-SLPrice-XXVIII_dfFA1608_FA1608.cif	30	0.272
XXVIII_structure_388.cif	XXVIII-1-MNeumann-structure_837.cif	30	0.223
XXVIII_structure_389.cif	XXVIII-1-SLPrice-XXVIII_dfFB240_FB240.cif	30	0.274
XXVIII_structure_390.cif	XXVIII-1-SLPrice-XXVIII_dfFA945_FA945.cif	30	0.135
XXVIII_structure_391.cif	XXVIII-1-MNeumann-structure_43.cif	30	0.399
XXVIII_structure_392.cif	XXVIII-1-SLPrice-XXVIII_dfCC578_CC578.cif	30	0.251
XXVIII_structure_393.cif	XXVIII-1-MNeumann-structure_1261.cif	30	0.324
XXVIII_structure_394.cif	XXVIII-1-MNeumann-structure_104.cif	30	0.351
XXVIII_structure_395.cif	XXVIII-1-MNeumann-structure_697.cif	30	0.440
XXVIII_structure_396.cif	XXVIII-1-SLPrice-XXVIII_dfFA264_FA264.cif	30	0.091
XXVIII_structure_397.cif	XXVIII-1-MNeumann-structure_710.cif	30	0.258
XXVIII_structure_398.cif	XXVIII-1-MNeumann-structure_496.cif	30	0.376
XXVIII_structure_399.cif	XXVIII-1-MNeumann-structure_1387.cif	30	0.312
XXVIII_structure_400.cif	XXVIII-1-MNeumann-structure_724.cif	30	0.213
XXVIII_structure_401.cif	XXVIII-1-MNeumann-structure_18.cif	30	0.323
XXVIII_structure_402.cif	XXVIII-1-XtalPi-data_261_T1_st_YTl1-fW7BwAySo50.cif	30	0.117
XXVIII_structure_403.cif	XXVIII-1-SLPrice-XXVIII_dfFA1155_FA1155.cif	30	0.176
XXVIII_structure_404.cif	XXVIII-1-SLPrice-XXVIII_dfCB213_CB213.cif	30	0.236
XXVIII_structure_405.cif	XXVIII-1-MNeumann-structure_938.cif	30	0.466
XXVIII_structure_406.cif	XXVIII-1-MNeumann-structure_895.cif	30	0.462
XXVIII_structure_407.cif	XXVIII-1-SLPrice-XXVIII_dfFA557_FA557.cif	30	0.225
XXVIII_structure_408.cif	XXVIII-1-MNeumann-structure_610.cif	30	0.308
XXVIII_structure_409.cif	XXVIII-1-CShang-XXVIII_409.cif	30	0.408
XXVIII_structure_410.cif	XXVIII-1-SLPrice-XXVIII_dfFA1280_FA1280.cif	30	0.186
XXVIII_structure_411.cif	XXVIII-1-MNeumann-structure_776.cif	30	0.289
XXVIII_structure_412.cif	XXVIII-1-XtalPi-data_640_T1_st_YTl1-fW7BwAySpCr.cif	22	0.392
XXVIII_structure_413.cif	XXVIII-1-SLPrice-XXVIII_dfFC222_FC222.cif	30	0.572

XXVIII_structure_414.cif	XXVIII-1-XtalPi-data_979_T1_st_YTl1-fW7BwAySpDK.cif	30	0.145
XXVIII_structure_415.cif	XXVIII-1-MNeumann-structure_527.cif	30	0.351
XXVIII_structure_416.cif	XXVIII-1-SLPrice-XXVIII_dfCB250_CB250.cif	30	0.277
XXVIII_structure_417.cif	XXVIII-1-MNeumann-structure_1015.cif	30	0.364
XXVIII_structure_418.cif	XXVIII-1-SLPrice-XXVIII_dfFA834_FA834.cif	30	0.096
XXVIII_structure_419.cif	XXVIII-1-SLPrice-XXVIII_dfFA2513_FA2513.cif	30	0.223
XXVIII_structure_420.cif	XXVIII-1-SLPrice-XXVIII_dfCB352_CB352.cif	30	0.219
XXVIII_structure_421.cif	XXVIII-1-SLPrice-XXVIII_dfFA43_FA43.cif	30	0.372
XXVIII_structure_422.cif	XXVIII-1-MNeumann-structure_1230.cif	30	0.209
XXVIII_structure_423.cif	XXVIII-1-SLPrice-XXVIII_dfFA867_FA867.cif	30	0.145
XXVIII_structure_424.cif	XXVIII-1-XtalPi-data_660_T1_st_YTl1-fW7BwAySo-l.cif	30	0.508
XXVIII_structure_425.cif	XXVIII-1-SLPrice-XXVIII_dfFC161_FC161.cif	30	0.237
XXVIII_structure_426.cif	XXVIII-1-MNeumann-structure_1132.cif	30	0.324
XXVIII_structure_427.cif	XXVIII-1-XtalPi-data_391_C1_st_YKRuUk3fRgAoi1De.cif	30	0.354
XXVIII_structure_428.cif	XXVIII-1-MNeumann-structure_1243.cif	30	0.213
XXVIII_structure_429.cif	XXVIII-1-SLPrice-XXVIII_dfFA22_FA22.cif	30	0.285
XXVIII_structure_430.cif	XXVIII-1-SLPrice-XXVIII_dfFA110_FA110.cif	30	0.180
XXVIII_structure_431.cif	XXVIII-1-XtalPi-data_699_T1_st_YTl1-fW7BwAySpAu.cif	30	0.237
XXVIII_structure_432.cif	XXVIII-1-MNeumann-structure_1252.cif	30	0.424
XXVIII_structure_433.cif	XXVIII-1-MNeumann-structure_800.cif	30	0.251
XXVIII_structure_434.cif	XXVIII-1-XtalPi-data_148_T1_st_YTl1-fW7BwAySpA7.cif	30	0.196
XXVIII_structure_435.cif	XXVIII-1-SLPrice-XXVIII_dfFC101_FC101.cif	30	0.196
XXVIII_structure_436.cif	XXVIII-1-MNeumann-structure_680.cif	30	0.286
XXVIII_structure_437.cif	XXVIII-1-XtalPi-data_47_T1_st_YK2w9oAjAAAnSf43.cif	29	0.787
XXVIII_structure_438.cif	XXVIII-1-MNeumann-structure_1217.cif	30	0.317
XXVIII_structure_439.cif	XXVIII-1-SLPrice-XXVIII_dfFB214_FB214.cif	30	0.405
XXVIII_structure_440.cif	XXVIII-1-MNeumann-structure_338.cif	30	0.418
XXVIII_structure_441.cif	XXVIII-1-CShang-XXVIII_590.cif	30	0.000
XXVIII_structure_442.cif	XXVIII-1-XtalPi-data_311_T1_st_YTl1-fW7BwAySo4P.cif	30	0.131
XXVIII_structure_443.cif	XXVIII-1-SLPrice-XXVIII_dfFB101_FB101.cif	30	0.356
XXVIII_structure_444.cif	XXVIII-1-MNeumann-structure_444.cif	30	0.299
XXVIII_structure_445.cif	XXVIII-1-SLPrice-XXVIII_dfAA27_AA27.cif	30	0.168
XXVIII_structure_446.cif	XXVIII-1-XtalPi-data_165_T1_st_YK2w9oAjAAAnSf57.cif	30	0.136
XXVIII_structure_447.cif	XXVIII-1-MNeumann-structure_461.cif	30	0.364
XXVIII_structure_448.cif	XXVIII-1-SLPrice-XXVIII_dfFA122_FA122.cif	30	0.134
XXVIII_structure_449.cif	XXVIII-1-MNeumann-structure_210.cif	30	0.388
XXVIII_structure_450.cif	XXVIII-1-MNeumann-structure_50.cif	30	0.306
XXVIII_structure_451.cif	XXVIII-1-XtalPi-data_616_T1_st_YTl1-fW7BwAySpBe.cif	30	0.145
XXVIII_structure_452.cif	XXVIII-1-MNeumann-structure_1340.cif	30	0.280
XXVIII_structure_453.cif	XXVIII-1-XtalPi-data_90_T1_st_YTl1-fW7BwAySo4y.cif	30	0.400
XXVIII_structure_454.cif	XXVIII-1-XtalPi-data_255_T1_st_YTl1-fW7BwAySo5g.cif	30	0.082
XXVIII_structure_455.cif	XXVIII-1-MNeumann-structure_476.cif	30	0.258
XXVIII_structure_456.cif	XXVIII-1-CShang-XXVIII_1220.cif	30	0.333
XXVIII_structure_457.cif	XXVIII-1-MNeumann-structure_593.cif	30	0.279
XXVIII_structure_458.cif	XXVIII-1-MNeumann-structure_526.cif	30	0.273
XXVIII_structure_459.cif	XXVIII-1-XtalPi-data_1181_C1_st_YTl0Nsor_gAwR_T-.cif	30	0.369
XXVIII_structure_460.cif	XXVIII-1-MNeumann-structure_1010.cif	30	0.334
XXVIII_structure_461.cif	XXVIII-1-MNeumann-structure_404.cif	30	0.319
XXVIII_structure_462.cif	XXVIII-1-MNeumann-structure_862.cif	30	0.303
XXVIII_structure_463.cif	XXVIII-1-SLPrice-XXVIII_dfFA1436_FA1436.cif	30	0.208
XXVIII_structure_464.cif	XXVIII-1-SLPrice-XXVIII_dfFA33_FA33.cif	30	0.243
XXVIII_structure_465.cif	XXVIII-1-SLPrice-XXVIII_dfCB273_CB273.cif	30	0.402
XXVIII_structure_466.cif	XXVIII-1-SLPrice-XXVIII_dfFA68_FA68.cif	30	0.074
XXVIII_structure_467.cif	XXVIII-1-SLPrice-XXVIII_dfFA1984_FA1984.cif	30	0.166
XXVIII_structure_468.cif	XXVIII-1-MNeumann-structure_922.cif	30	0.275
XXVIII_structure_469.cif	XXVIII-1-SLPrice-XXVIII_dfFA891_FA891.cif	30	0.396
XXVIII_structure_470.cif	XXVIII-1-SLPrice-XXVIII_dfFA1065_FA1065.cif	30	0.828
XXVIII_structure_471.cif	XXVIII-1-SLPrice-XXVIII_dfFA1295_FA1295.cif	30	0.238
XXVIII_structure_472.cif	XXVIII-1-XtalPi-data_757_C1_st_YTl0Nsor_gAwR_SS.cif	30	0.119

XXVIII_structure_473.cif	XXVIII-1-MNeumann-structure_827.cif	30	0.298
XXVIII_structure_474.cif	XXVIII-1-CShang-XXVIII_1227.cif	30	0.000
XXVIII_structure_475.cif	XXVIII-1-MNeumann-structure_162.cif	30	0.262
XXVIII_structure_476.cif	XXVIII-1-MNeumann-structure_1386.cif	30	0.400
XXVIII_structure_477.cif	XXVIII-1-MNeumann-structure_1355.cif	30	0.245
XXVIII_structure_478.cif	XXVIII-1-XtalPi-data_187_T1_st_YTl1-fW7BwAySo4W.cif	30	0.349
XXVIII_structure_479.cif	XXVIII-1-SLPrice-XXVIII_dfFB291_FB291.cif	30	0.220
XXVIII_structure_480.cif	XXVIII-1-SLPrice-XXVIII_dfFC40_FC40.cif	30	0.098
XXVIII_structure_481.cif	XXVIII-1-MNeumann-structure_59.cif	30	0.489
XXVIII_structure_482.cif	XXVIII-1-SLPrice-XXVIII_dfFC409_FC409.cif	30	0.175
XXVIII_structure_483.cif	XXVIII-1-MNeumann-structure_38.cif	30	0.301
XXVIII_structure_484.cif	XXVIII-1-MNeumann-structure_21.cif	30	0.415
XXVIII_structure_485.cif	XXVIII-1-SLPrice-XXVIII_dfCB628_CB628.cif	30	0.213
XXVIII_structure_486.cif	XXVIII-1-MNeumann-structure_687.cif	30	0.317
XXVIII_structure_487.cif	XXVIII-1-MNeumann-structure_518.cif	23	0.284
XXVIII_structure_488.cif	XXVIII-1-SLPrice-XXVIII_dfFA167_FA167.cif	30	0.230
XXVIII_structure_489.cif	XXVIII-1-MNeumann-structure_260.cif	30	0.345
XXVIII_structure_490.cif	XXVIII-1-SLPrice-XXVIII_dfFC10_FC10.cif	30	0.143
XXVIII_structure_491.cif	XXVIII-1-SLPrice-XXVIII_dfCC609_CC609.cif	30	0.334
XXVIII_structure_492.cif	XXVIII-1-MNeumann-structure_380.cif	30	0.300
XXVIII_structure_493.cif	XXVIII-1-MNeumann-structure_431.cif	30	0.302
XXVIII_structure_494.cif	XXVIII-1-MNeumann-structure_299.cif	30	0.252
XXVIII_structure_495.cif	XXVIII-1-SLPrice-XXVIII_dfCB173_CB173.cif	30	0.227
XXVIII_structure_496.cif	XXVIII-1-SLPrice-XXVIII_dfCC471_CC471.cif	30	0.206
XXVIII_structure_497.cif	XXVIII-1-SLPrice-XXVIII_dfFA721_FA721.cif	30	0.235
XXVIII_structure_498.cif	XXVIII-1-MNeumann-structure_879.cif	30	0.294
XXVIII_structure_499.cif	XXVIII-1-MNeumann-structure_1103.cif	30	0.297
XXVIII_structure_500.cif	XXVIII-1-SLPrice-XXVIII_dfFA1299_FA1299.cif	30	0.265

Table 5: (Target XXXI) The CIF names of the CCDC-prepared structures provided to participants for the structure ranking exercise, the corresponding CIF sampled from the first phase (structure generation exercise) of the seventh blind test, and the results (number of molecules matched in a cluster of 30 and RMSD) from COMPACK comparisons (applying 25% and 25° distance/angle tolerances) between the two to assess the structural change resultant from constrained optimisations carried out by the organisers in the structure list preparation. (* indicates structures representing experimental forms of XXXI.) (Originating CIF names contain an added prefix of the form '{target}-{phase}-{group name/label}-')

CCDC-prepared CIF name	Originating CIF name	Molecules matched	RMSD (Å)
*XXXI_structure_1.cif	*XXXI-1-SLPrice-BTXXXI_df1_1.cif	30	0.189
XXXI_structure_2.cif	XXXI-1-MaromIsayev-MI_0129_dft_087c7ed0.cif	30	0.180
XXXI_structure_3.cif	XXXI-1-XtalPi-data_476_z1_st_YNrfI1Za63KAghh0.cif	30	0.144
XXXI_structure_4.cif	XXXI-1-MNeumann-structure_1019.cif	30	0.254
XXXI_structure_5.cif	XXXI-1-GDay-XXXI_conf_54_opt-QR-14-21525-3_dftbopt_final.cif	30	0.366
XXXI_structure_6.cif	XXXI-1-SMohamed-data_1362.cif	30	0.334
XXXI_structure_7.cif	XXXI-1-OpenEye_structure_351.cif	30	0.166
XXXI_structure_8.cif	XXXI-1-XtalPi-data_540_z1_st_YNrfI1Za63KAghiw.cif	30	0.198
XXXI_structure_9.cif	XXXI-1-CSAdjiman-CSOFM_00544.cif	30	0.230
XXXI_structure_10.cif	XXXI-1-MNeumann-structure_300.cif	30	0.126
XXXI_structure_11.cif	XXXI-1-MNeumann-structure_27.cif	30	0.192
XXXI_structure_12.cif	XXXI-1-DBoese-xxxi-ca39_e0-s1079-co-f-477.cif	30	0.202
XXXI_structure_13.cif	XXXI-1-DBoese-xxxi-ca269_e0-s1181-co-f-454.cif	30	0.197
XXXI_structure_14.cif	XXXI-1-DBoese-xxxi-ca269_e0-s93-co-276.cif	30	0.071
XXXI_structure_15.cif	XXXI-1-DBoese-xxxi-ca120_e0-s364-co-275.cif	30	0.164
XXXI_structure_16.cif	XXXI-1-OpenEye_structure_845.cif	30	0.147
XXXI_structure_17.cif	XXXI-1-GDay-XXXI_conf_54_opt.XXXI_conf_54i_opt-QR-4-1390-3_dftbopt_final.cif	30	0.203
XXXI_structure_18.cif	XXXI-1-GDay-XXXI_conf_16_optx2-QR-2-868-3_dftbopt_final.cif	30	0.197
XXXI_structure_19.cif	XXXI-1-DBoese-xxxi-ca269_e0-s7626-co-f-446.cif	30	0.107
XXXI_structure_20.cif	XXXI-1-XtalPi-data_102_z1_st_YNrfI1Za63KAghh3.cif	30	0.120
XXXI_structure_21.cif	XXXI-1-CSAdjiman-CSOFM_00594.cif	30	0.256
XXXI_structure_22.cif	XXXI-1-MaromIsayev-MI_0040_dft_30775be7.cif	30	0.225
XXXI_structure_23.cif	XXXI-1-OpenEye_structure_1224.cif	30	0.242
XXXI_structure_24.cif	XXXI-1-DBoese-xxxi-ca269_e0-s1169-co-f-363.cif	30	0.305
XXXI_structure_25.cif	*XXXI-1-MaromIsayev-MI_0046_dft_3199da20.cif	30	0.309
XXXI_structure_26.cif	XXXI-1-MaromIsayev-MI_0994_dft_a761311d.cif	30	0.220
XXXI_structure_27.cif	XXXI-1-OpenEye_structure_104.cif	30	0.249
XXXI_structure_28.cif	XXXI-1-OpenEye_structure_57.cif	30	0.262
XXXI_structure_29.cif	XXXI-1-DBoese-xxxi-ca120_e0-s667-co-f-538.cif	30	0.095
XXXI_structure_30.cif	XXXI-1-DBoese-xxxi-ca269_e0-s9-co-f-36.cif	30	0.359
XXXI_structure_31.cif	XXXI-1-CSAdjiman-CSOFM_00777.cif	30	0.168
XXXI_structure_32.cif	XXXI-1-XtalPi-data_226_z1_st_YNrfI1Za63KAghiq.cif	30	0.194
XXXI_structure_33.cif	XXXI-1-XtalPi-data_933_z1_st_YIk-iQwufAABFO_C.cif	30	0.178
XXXI_structure_34.cif	XXXI-1-MaromIsayev-MI_0014_dft_b2c518c8.cif	30	0.177
XXXI_structure_35.cif	XXXI-1-OpenEye_structure_496.cif	30	0.247
XXXI_structure_36.cif	XXXI-1-MNeumann-structure_732.cif	30	0.221
XXXI_structure_37.cif	XXXI-1-DBoese-xxxi-ca269_e0-s1332-co-f-674.cif	30	0.399
XXXI_structure_38.cif	XXXI-1-DBoese-xxxi-ca358_e0-s1659-co-100.cif	30	0.202
XXXI_structure_39.cif	XXXI-1-MNeumann-structure_433.cif	30	0.169
XXXI_structure_40.cif	XXXI-1-MNeumann-structure_724.cif	30	0.162
XXXI_structure_41.cif	XXXI-1-OpenEye_structure_43.cif	30	0.440
XXXI_structure_42.cif	XXXI-1-MaromIsayev-MI_0369_aimnet_3ba8b34f.cif	30	0.269
XXXI_structure_43.cif	XXXI-1-DBoese-xxxi-ca39_e0-s8016-co-713.cif	30	0.153
XXXI_structure_44.cif	XXXI-1-CSAdjiman-CSOFM_00890.cif	30	0.310
XXXI_structure_45.cif	XXXI-1-MaromIsayev-MI_1159_dft_30c8cdd4.cif	30	0.176
XXXI_structure_46.cif	XXXI-1-OpenEye_structure_646.cif	30	0.247
XXXI_structure_47.cif	XXXI-1-OpenEye_structure_609.cif	30	0.378
XXXI_structure_48.cif	XXXI-1-XtalPi-data_64_z1_st_YNrfI1Za63KAghiD.cif	30	0.159

XXXI_structure_49.cif	XXXI-1-DBoese-xxxi-ca39_e0-s456-co-f-730.cif	30	0.222
XXXI_structure_50.cif	XXXI-1-MaromIsayev-MI_0334_dft_dd12e4fc.cif	30	0.268
XXXI_structure_51.cif	XXXI-1-OpenEye_structure_774.cif	30	0.353
XXXI_structure_52.cif	XXXI-1-DBoese-xxxi-ca269_e0-s973-co-213.cif	30	0.166
XXXI_structure_53.cif	XXXI-1-GDay-scan_12-QR-2-3006-3_dftbopt_final.cif	30	0.384
XXXI_structure_54.cif	XXXI-1-CSAdjiman-CSOFM_01268.cif	30	0.276
XXXI_structure_55.cif	XXXI-1-OpenEye_structure_149.cif	30	0.267
XXXI_structure_56.cif	XXXI-1-MaromIsayev-MI_0766_dft_39437f1e.cif	30	0.145
XXXI_structure_57.cif	XXXI-1-MNeumann-structure_205.cif	30	0.276
XXXI_structure_58.cif	XXXI-1-MaromIsayev-MI_0258_dft_7f9c6dde.cif	30	0.217
XXXI_structure_59.cif	XXXI-1-MaromIsayev-MI_0001_dft_f825ab4f.cif	30	0.105
XXXI_structure_60.cif	XXXI-1-DBoese-xxxi-ca39_e0-s1029-co-f-497.cif	30	0.283
XXXI_structure_61.cif	XXXI-1-XtalPi-data_475_z1_st_YNrfI1Za63KAghiP.cif	30	0.299
XXXI_structure_62.cif	XXXI-1-CSAdjiman-CSOFM_00702.cif	30	0.343
XXXI_structure_63.cif	XXXI-1-MNeumann-structure_1180.cif	30	0.234
XXXI_structure_64.cif	XXXI-1-CSAdjiman-CSOFM_00904.cif	30	0.281
XXXI_structure_65.cif	XXXI-1-CSAdjiman-CSOFM_00389.cif	30	0.298
XXXI_structure_66.cif	XXXI-1-DBoese-xxxi-ca39_e0-s935-co-f-729.cif	30	0.201
XXXI_structure_67.cif	XXXI-1-SMohamed-data_1226.cif	30	0.303
XXXI_structure_68.cif	XXXI-1-XtalPi-data_353_z1_st_YDMR5hjU-QABVnk0.cif	30	0.125
XXXI_structure_69.cif	XXXI-1-OpenEye_structure_130.cif	30	0.269
XXXI_structure_70.cif	XXXI-1-MNeumann-structure_99.cif	30	0.196
XXXI_structure_71.cif	XXXI-1-OpenEye_structure_312.cif	30	0.437
XXXI_structure_72.cif	XXXI-1-MNeumann-structure_936.cif	30	0.187
XXXI_structure_73.cif	XXXI-1-MaromIsayev-MI_0118_aimnet_3e8fe5c6.cif	30	0.231
XXXI_structure_74.cif	XXXI-1-CSAdjiman-VASP_MIN_45.cif	30	0.129
XXXI_structure_75.cif	XXXI-1-GDay-XXXI_conf_7_opt-QR-2-15190-3_dftbopt_final.cif	30	0.786
XXXI_structure_76.cif	XXXI-1-CSAdjiman-CSOFM_01092.cif	30	0.163
XXXI_structure_77.cif	XXXI-1-GDay-XXXI_conf_61_opt-QR-14-26871-3_dftbopt_final.cif	30	0.249
XXXI_structure_78.cif	XXXI-1-MaromIsayev-MI_0454_dft_c1ddec1d.cif	30	0.252
XXXI_structure_79.cif	XXXI-1-OpenEye_structure_293.cif	30	0.264
XXXI_structure_80.cif	XXXI-1-MaromIsayev-MI_1434_dft_8c41b222.cif	30	0.322
XXXI_structure_81.cif	XXXI-1-XtalPi-data_812_z1_st_YDMR5hjU-QABVniu.cif	30	0.113
XXXI_structure_82.cif	XXXI-1-GDay-scan_2-QR-2-2591-3_dftbopt_final.cif	30	0.612
XXXI_structure_83.cif	XXXI-1-MaromIsayev-MI_0829_dft_e4dd5d29.cif	30	0.224
XXXI_structure_84.cif	XXXI-1-OpenEye_structure_1266.cif	30	0.323
XXXI_structure_85.cif	XXXI-1-DBoese-xxxi-ca269_e0-s208-co-f-348.cif	30	0.092
XXXI_structure_86.cif	XXXI-1-GDay-XXXI_conf_0_opt-QR-14-12714-3_dftbopt_final.cif	30	0.294
XXXI_structure_87.cif	XXXI-1-GDay-XXXI_conf_0_opt-QR-2-4050-3_dftbopt_final.cif	30	0.340
XXXI_structure_88.cif	XXXI-1-DBoese-xxxi-ca269_e0-s176-co-f-767.cif	30	0.376
XXXI_structure_89.cif	*ZEHFUR01	30	0.200
XXXI_structure_90.cif	XXXI-1-GDay-XXXI_conf_7_opt-QR-4-2196-3_dftbopt_final.cif	30	0.256
XXXI_structure_91.cif	XXXI-1-GDay-XXXI_conf_0_optx2-QR-2-45153-3_dftbopt_final.cif	30	0.205
XXXI_structure_92.cif	XXXI-1-MaromIsayev-MI_1363_dft_0f540d8f.cif	30	0.155
XXXI_structure_93.cif	XXXI-1-OpenEye_structure_264.cif	30	0.153
XXXI_structure_94.cif	XXXI-1-DBoese-xxxi-ca120_e0-s842-co-158.cif	30	0.174
XXXI_structure_95.cif	XXXI-1-OpenEye_structure_474.cif	30	0.126
XXXI_structure_96.cif	XXXI-1-OpenEye_structure_344.cif	30	0.198
XXXI_structure_97.cif	XXXI-1-GDay-XXXI_conf_7_opt-QR-14-34783-3_dftbopt_final.cif	30	0.196
XXXI_structure_98.cif	*XXXI-1-CSAdjiman-VASP_MIN_3.cif	30	0.169
XXXI_structure_99.cif	XXXI-1-XtalPi-data_648_z1_st_YDMR5hjU-QABVnj7.cif	30	0.175
XXXI_structure_100.cif	XXXI-1-SMohamed-data_1334.cif	30	0.182

Table 6: (Target XXXII) The CIF names of the CCDC-prepared structures provided to participants for the structure ranking exercise, the corresponding CIF sampled from the first phase (structure generation exercise) of the seventh blind test, and the results (number of molecules matched in a cluster of 30 and RMSD) from COMPACK comparisons (applying 25% and 25° distance/angle tolerances) between the two to assess the structural change resultant from constrained optimisations carried out by the organisers in the structure list preparation. (* indicates structures representing experimental forms of XXXII. ˆ indicates structures which showed highest PXRD similarity to the unknown forms of XXXII, see table 21. (Originating CIF names contain an added prefix of the form '{target}-{phase}-{group name/label}-')

CCDC-prepared CIF name	Originating CIF name	Molecules matched	RMSD (Å)
XXXII_structure_1.cif	XXXII-1-XtalPi-data_87_z1_st_YUl4CeqPPieGr_3K.cif	30	0.168
XXXII_structure_2.cif	XXXII-1-XtalPi-data_395_z1_st_YML9yQwufAABcYZj.cif	30	0.170
XXXII_structure_3.cif	XXXII-1-MNeumann-structure_369.cif	30	0.237
ˆXXXII_structure_4.cif	ˆXXXII-1-CShang-XXXII_64.cif	30	0.215
ˆXXXII_structure_5.cif	ˆXXXII-1-DBoese-xxxii-ca53_e150-s33-co-497.cif	30	0.128
XXXII_structure_6.cif	XXXII-1-XtalPi-data_449_z1_st_YUl4CeqPPieGr_37.cif	30	0.211
XXXII_structure_7.cif	XXXII-1-MNeumann-structure_338.cif	30	0.178
XXXII_structure_8.cif	XXXII-1-MNeumann-structure_1395.cif	30	0.200
XXXII_structure_9.cif	XXXII-1-XtalPi-data_5_z1_st_YD8t7RjU-QABeTPp.cif	30	0.207
XXXII_structure_10.cif	XXXII-1-XtalPi-data_1106_z1_st_YUl4CeqPPieGr_5I.cif	30	0.187
XXXII_structure_11.cif	XXXII-1-MNeumann-structure_1149.cif	30	0.353
XXXII_structure_12.cif	XXXII-1-XtalPi-data_778_z1_st_YUl4CeqPPieGr_4M.cif	30	0.170
XXXII_structure_13.cif	XXXII-1-MNeumann-structure_874.cif	30	0.105
XXXII_structure_14.cif	XXXII-1-XtalPi-data_181_z1_st_YML9yQwufAABcYX7.cif	30	0.172
XXXII_structure_15.cif	XXXII-1-XtalPi-data_1025_z1_st_YUl4CeqPPieGr_5o.cif	30	0.202
XXXII_structure_16.cif	XXXII-1-XtalPi-data_185_z1_st_YD8t7RjU-QABeTTA.cif	30	0.146
XXXII_structure_17.cif	XXXII-1-XtalPi-data_1142_z1_st_YUl4CeqPPieGr_1b.cif	30	0.205
XXXII_structure_18.cif	XXXII-1-XtalPi-data_351_z1_st_YVRyUeqPPis2KF4H.cif	30	0.169
XXXII_structure_19.cif	XXXII-1-XtalPi-data_653_z1_st_YUl4CeqPPieGr_6X.cif	30	0.203
XXXII_structure_20.cif	XXXII-1-XtalPi-data_595_z1_st_YD8t7RjU-QABeTf6.cif	30	0.180
XXXII_structure_21.cif	XXXII-1-XtalPi-data_967_z1_st_YUl4CeqPPieGr_25.cif	30	0.227
XXXII_structure_22.cif	XXXII-1-XtalPi-data_1018_z1_st_YD8t7RjU-QABeTXS.cif	30	0.229
XXXII_structure_23.cif	XXXII-1-XtalPi-data_701_z1_st_YD8t7RjU-QABeTUF.cif	30	0.239
XXXII_structure_24.cif	XXXII-1-XtalPi-data_517_z1_st_YVRyUeqPPis2KF5C.cif	30	0.161
XXXII_structure_25.cif	XXXII-1-XtalPi-data_1496_z1_st_YXPXcuqPPis2KGVf.cif	30	0.143
XXXII_structure_26.cif	XXXII-1-XtalPi-data_607_z1_st_YD8t7RjU-QABeTTb.cif	30	0.133
XXXII_structure_27.cif	XXXII-1-XtalPi-data_1036_z1_st_YD8t7RjU-QABeTX8.cif	30	0.351
XXXII_structure_28.cif	XXXII-1-XtalPi-data_899_z1_st_YUl4CeqPPieGr_zF.cif	30	0.196
XXXII_structure_29.cif	XXXII-1-XtalPi-data_681_z1_st_YD8t7RjU-QABeTeE.cif	30	0.176
XXXII_structure_30.cif	*XXXII_FormB_FormM _RT_incorrect_determination.cif	30	0.482
XXXII_structure_31.cif	XXXII-1-MNeumann-structure_960.cif	30	0.177
XXXII_structure_32.cif	XXXII-1-XtalPi-data_733_z1_st_YD8t7RjU-QABeTZd.cif	30	0.162
XXXII_structure_33.cif	XXXII-1-XtalPi-data_415_z1_st_YVRyUeqPPis2KF5J.cif	30	0.158
XXXII_structure_34.cif	XXXII-1-XtalPi-data_1021_z1_st_YUl4CeqPPieGr_6K.cif	30	0.156
XXXII_structure_35.cif	XXXII-1-XtalPi-data_294_z1_st_YUl4CeqPPieGr_38.cif	30	0.184
XXXII_structure_36.cif	XXXII-1-MNeumann-structure_321.cif	30	0.235
XXXII_structure_37.cif	XXXII-1-MNeumann-structure_922.cif	30	0.130
XXXII_structure_38.cif	XXXII-1-XtalPi-data_928_z1_st_YD8t7RjU-QABeTfu.cif	30	0.184
XXXII_structure_39.cif	XXXII-1-XtalPi-data_662_z1_st_YD8t7RjU-QABeTct.cif	30	0.213
XXXII_structure_40.cif	XXXII-1-XtalPi-data_267_z1_st_YVRyUeqPPis2KF43.cif	30	0.239
XXXII_structure_41.cif	XXXII-1-MNeumann-structure_752.cif	30	0.178
XXXII_structure_42.cif	XXXII-1-XtalPi-data_856_z1_st_YUl4CeqPPieGr_4w.cif	30	0.194
XXXII_structure_43.cif	XXXII-1-XtalPi-data_603_z1_st_YUl4CeqPPieGr_0J.cif	30	0.171
XXXII_structure_44.cif	XXXII-1-MNeumann-structure_447.cif	30	0.202
XXXII_structure_45.cif	XXXII-1-MNeumann-structure_1338.cif	30	0.259
XXXII_structure_46.cif	XXXII-1-XtalPi-data_359_z1_st_YML9yQwufAABcYZc.cif	30	0.143
XXXII_structure_47.cif	XXXII-1-XtalPi-data_248_z1_st_YVRyUeqPPis2KF4O.cif	30	0.186

ˆXXXII_structure_48.cif	ˆXXXII-1-AOganov-data_362.cif	30	0.195
XXXII_structure_49.cif	XXXII-1-XtalPi-data_409_z1_st_YVRyUeqPPis2KF4E.cif	30	0.241
ˆXXXII_structure_50.cif	ˆXXXII-1-AOganov-data_552.cif	30	0.276
XXXII_structure_51.cif	XXXII-1-XtalPi-data_591_z1_st_YUl4CeqPPieGr_yt.cif	30	0.203
ˆXXXII_structure_52.cif	ˆXXXII-1-CSAdjiman-CSOFM_01100.cif	30	0.156
XXXII_structure_53.cif	XXXII-1-XtalPi-data_116_z1_st_YVRyUeqPPis2KF3Q.cif	30	0.199
XXXII_structure_54.cif	XXXII-1-XtalPi-data_1078_z1_st_YUl4CeqPPieGr_1Y.cif	30	0.177
XXXII_structure_55.cif	XXXII-1-XtalPi-data_336_z1_st_YML9yQwufAABcYYM.cif	30	0.218
XXXII_structure_56.cif	XXXII-1-XtalPi-data_693_z1_st_YML9yQwufAABcYaM.cif	30	0.191
XXXII_structure_57.cif	XXXII-1-XtalPi-data_168_z1_st_YVRyUeqPPis2KF3U.cif	30	0.196
ˆXXXII_structure_58.cif	ˆXXXII-1-MNeumann-structure_558.cif	30	0.182
XXXII_structure_59.cif	XXXII-1-MNeumann-structure_1312.cif	30	0.156
XXXII_structure_60.cif	XXXII-1-MNeumann-structure_994.cif	30	0.135
XXXII_structure_61.cif	XXXII-1-XtalPi-data_1138_z1_st_YML9yQwufAABcYaF.cif	30	0.200
XXXII_structure_62.cif	XXXII-1-XtalPi-data_1125_z1_st_YD8t7RjU-QABeTUn.cif	30	0.265
XXXII_structure_63.cif	XXXII-1-XtalPi-data_708_z1_st_YML9yQwufAABcYY8.cif	30	0.183
XXXII_structure_64.cif	XXXII-1-XtalPi-data_1052_z1_st_YD8t7RjU-QABeTdc.cif	30	0.222
XXXII_structure_65.cif	XXXII-1-XtalPi-data_1150_z1_st_YUl4CeqPPieGr_13.cif	30	0.182
ˆXXXII_structure_66.cif	ˆXXXII-1-MNeumann-structure_620.cif	30	0.181
XXXII_structure_67.cif	XXXII-1-XtalPi-data_1047_z1_st_YD8t7RjU-QABeTNQ.cif	30	0.223
XXXII_structure_68.cif	XXXII-1-MNeumann-structure_1238.cif	30	0.234
XXXII_structure_69.cif	XXXII-1-XtalPi-data_612_z1_st_YVRyUeqPPis2KF5H.cif	30	0.198
XXXII_structure_70.cif	XXXII-1-XtalPi-data_556_z1_st_YML9yQwufAABcYZa.cif	30	0.187
XXXII_structure_71.cif	XXXII-1-XtalPi-data_932_z1_st_YUl4CeqPPieGr_1s.cif	30	0.171
XXXII_structure_72.cif	XXXII-1-XtalPi-data_608_z1_st_YD8t7RjU-QABeTV8.cif	30	0.181
XXXII_structure_73.cif	XXXII-1-XtalPi-data_890_z1_st_YUl4CeqPPieGr_5M.cif	30	0.255
XXXII_structure_74.cif	XXXII-1-XtalPi-data_670_z1_st_YVRyUeqPPis2KF45.cif	30	0.197
XXXII_structure_75.cif	XXXII-1-XtalPi-data_555_z1_st_YML9yQwufAABcYX0.cif	30	0.202
XXXII_structure_76.cif	XXXII-1-XtalPi-data_361_z1_st_YVRyUeqPPis2KF3o.cif	30	0.234
XXXII_structure_77.cif	XXXII-1-MNeumann-structure_1055.cif	30	0.140
XXXII_structure_78.cif	XXXII-1-XtalPi-data_901_z1_st_YML9yQwufAABcYYt.cif	30	0.184
XXXII_structure_79.cif	XXXII-1-XtalPi-data_696_z1_st_YD8t7RjU-QABeTch.cif	30	0.146
XXXII_structure_80.cif	XXXII-1-XtalPi-data_1020_z1_st_YUl4CeqPPieGr_5z.cif	30	0.182
XXXII_structure_81.cif	XXXII-1-XtalPi-data_1139_z1_st_YD8t7RjU-QABeTRe.cif	30	0.152
XXXII_structure_82.cif	XXXII-1-MNeumann-structure_116.cif	30	0.276
XXXII_structure_83.cif	XXXII-1-XtalPi-data_1103_z1_st_YUl4CeqPPieGr_2w.cif	30	0.172
XXXII_structure_84.cif	XXXII-1-XtalPi-data_1122_z1_st_YD8t7RjU-QABeTSS.cif	30	0.124
XXXII_structure_85.cif	XXXII-1-MNeumann-structure_719.cif	30	0.257
XXXII_structure_86.cif	XXXII-1-XtalPi-data_513_z1_st_YVRyUeqPPis2KF23.cif	30	0.255
XXXII_structure_87.cif	XXXII-1-XtalPi-data_942_z1_st_YD8t7RjU-QABeTf7.cif	30	0.193
XXXII_structure_88.cif	XXXII-1-XtalPi-data_714_z1_st_YUl4CeqPPieGr_yH.cif	30	0.175
XXXII_structure_89.cif	XXXII-1-XtalPi-data_150_z1_st_YVRyUeqPPis2KF2f.cif	30	0.115
XXXII_structure_90.cif	XXXII-1-XtalPi-data_401_z1_st_YVRyUeqPPis2KF3x.cif	30	0.222
XXXII_structure_91.cif	XXXII-1-XtalPi-data_404_z1_st_YVRyUeqPPis2KF30.cif	30	0.359
XXXII_structure_92.cif	XXXII-1-XtalPi-data_1062_z1_st_YD8t7RjU-QABeTex.cif	30	0.200
XXXII_structure_93.cif	XXXII-1-MNeumann-structure_1339.cif	30	0.152
XXXII_structure_94.cif	XXXII-1-XtalPi-data_910_z1_st_YUl4CeqPPieGr_5l.cif	30	0.360
XXXII_structure_95.cif	XXXII-1-MNeumann-structure_794.cif	30	0.209
XXXII_structure_96.cif	XXXII-1-MNeumann-structure_891.cif	30	0.275
XXXII_structure_97.cif	XXXII-1-XtalPi-data_1099_z1_st_YUl4CeqPPieGr_05.cif	30	0.118
XXXII_structure_98.cif	XXXII-1-XtalPi-data_474_z1_st_YVRyUeqPPis2KF5X.cif	30	0.270
XXXII_structure_99.cif	XXXII-1-MNeumann-structure_806.cif	30	0.184
XXXII_structure_100.cif	XXXII-1-XtalPi-data_936_z1_st_YD8t7RjU-QABeTfT.cif	30	0.132
XXXII_structure_101.cif	XXXII-1-XtalPi-data_552_z1_st_YD8t7RjU-QABeTXP.cif	30	0.184
ˆXXXII_structure_102.cif	ˆXXXII-1-XtalPi-data_1437_z2_st_YXE5cuqPPis2KGEy.cif	30	0.225
XXXII_structure_103.cif	XXXII-1-XtalPi-data_703_z1_st_YD8t7RjU-QABeTTx.cif	30	0.311
XXXII_structure_104.cif	XXXII-1-XtalPi-data_630_z1_st_YUl4CeqPPieGr_44.cif	30	0.175
ˆXXXII_structure_105.cif	ˆXXXII-1-MNeumann-structure_784.cif	30	0.230
XXXII_structure_106.cif	XXXII-1-XtalPi-data_878_z1_st_YD8t7RjU-QABeTOi.cif	30	0.186
XXXII_structure_107.cif	XXXII-1-MNeumann-structure_332.cif	30	0.158

XXXII_structure_108.cif	XXXII-1-XtalPi-data_377_z1_st_YVRyUeqPPis2KF3_.cif	30	0.183
XXXII_structure_109.cif	XXXII-1-XtalPi-data_319_z1_st_YVRyUeqPPis2KF2Z.cif	30	0.176
XXXII_structure_110.cif	XXXII-1-XtalPi-data_957_z1_st_YD8t7RjU-QABeTf9.cif	30	0.215
XXXII_structure_111.cif	XXXII-1-XtalPi-data_843_z1_st_YVRyUeqPPis2KF28.cif	30	0.217
XXXII_structure_112.cif	XXXII-1-XtalPi-data_392_z1_st_YVRyUeqPPis2KF36.cif	30	0.142
XXXII_structure_113.cif	XXXII-1-XtalPi-data_648_z1_st_YVRyUeqPPis2KF5I.cif	30	0.210
XXXII_structure_114.cif	XXXII-1-MNeumann-structure_93.cif	30	0.255
XXXII_structure_115.cif	XXXII-1-XtalPi-data_209_z1_st_YVRyUeqPPis2KF3t.cif	30	0.101
XXXII_structure_116.cif	XXXII-1-XtalPi-data_836_z1_st_YML9yQwufAABcYaX.cif	30	0.162
XXXII_structure_117.cif	XXXII-1-XtalPi-data_419_z1_st_YUl4CeqPPieGr_3b.cif	30	0.268
XXXII_structure_118.cif	XXXII-1-MNeumann-structure_963.cif	30	0.194
XXXII_structure_119.cif	XXXII-1-XtalPi-data_196_z1_st_YVRyUeqPPis2KF4A.cif	30	0.193
XXXII_structure_120.cif	XXXII-1-MNeumann-structure_443.cif	30	0.245
XXXII_structure_121.cif	XXXII-1-XtalPi-data_144_z1_st_YD8t7RjU-QABeTPh.cif	30	0.176
XXXII_structure_122.cif	XXXII-1-MNeumann-structure_652.cif	30	0.200
XXXII_structure_123.cif	XXXII-1-XtalPi-data_820_z1_st_YD8t7RjU-QABeTam.cif	30	0.157
XXXII_structure_124.cif	XXXII-1-XtalPi-data_546_z1_st_YVRyUeqPPis2KF5Y.cif	30	0.189
XXXII_structure_125.cif	XXXII-1-XtalPi-data_1121_z1_st_YD8t7RjU-QABeTOt.cif	30	0.127
XXXII_structure_126.cif	XXXII-1-XtalPi-data_80_z1_st_YVRyUeqPPis2KF3B.cif	30	0.166
XXXII_structure_127.cif	XXXII-1-XtalPi-data_801_z1_st_YD8t7RjU-QABeTfv.cif	30	0.154
XXXII_structure_128.cif	XXXII-1-XtalPi-data_1206_z2_st_YXJ6muqPPis2KGRo.cif	30	0.184
XXXII_structure_129.cif	XXXII-1-XtalPi-data_83_z1_st_YD8t7RjU-QABeTSa.cif	30	0.138
XXXII_structure_130.cif	XXXII-1-XtalPi-data_531_z1_st_YML9yQwufAABcYYg.cif	30	0.266
XXXII_structure_131.cif	XXXII-1-XtalPi-data_981_z1_st_YD8t7RjU-QABeTSz.cif	30	0.177
XXXII_structure_132.cif	XXXII-1-XtalPi-data_1167_z1_st_YXPXcuqPPis2KGVa.cif	30	0.233
XXXII_structure_133.cif	XXXII-1-XtalPi-data_813_z1_st_YUl4CeqPPieGr_0B.cif	30	0.143
XXXII_structure_134.cif	XXXII-1-XtalPi-data_444_z1_st_YD8t7RjU-QABeTRy.cif	30	0.159
XXXII_structure_135.cif	XXXII-1-MNeumann-structure_425.cif	30	0.200
XXXII_structure_136.cif	XXXII-1-MNeumann-structure_1411.cif	30	0.213
XXXII_structure_137.cif	XXXII-1-XtalPi-data_892_z1_st_YML9yQwufAABcYYc.cif	30	0.194
XXXII_structure_138.cif	XXXII-1-XtalPi-data_427_z1_st_YD8t7RjU-QABeTYM.cif	30	0.170
XXXII_structure_139.cif	XXXII-1-XtalPi-data_1002_z1_st_YUl4CeqPPieGr_5n.cif	30	0.190
XXXII_structure_140.cif	XXXII-1-XtalPi-data_37_z1_st_YVRyUeqPPis2KF4w.cif	30	0.228
XXXII_structure_141.cif	XXXII-1-XtalPi-data_470_z1_st_YD8t7RjU-QABeTbd.cif	30	0.156
XXXII_structure_142.cif	XXXII-1-MNeumann-structure_873.cif	30	0.191
XXXII_structure_143.cif	XXXII-1-XtalPi-data_950_z1_st_YML9yQwufAABcYYT.cif	30	0.209
XXXII_structure_144.cif	XXXII-1-XtalPi-data_97_z1_st_YVRyUeqPPis2KF2y.cif	30	0.269
XXXII_structure_145.cif	XXXII-1-XtalPi-data_1022_z1_st_YML9yQwufAABcYXt.cif	30	0.187
XXXII_structure_146.cif	XXXII-1-XtalPi-data_700_z1_st_YML9yQwufAABcYYf.cif	30	0.188
XXXII_structure_147.cif	XXXII-1-XtalPi-data_974_z1_st_YD8t7RjU-QABeTRP.cif	30	0.146
XXXII_structure_148.cif	XXXII-1-XtalPi-data_913_z1_st_YD8t7RjU-QABeTXC.cif	30	0.204
XXXII_structure_149.cif	XXXII-1-XtalPi-data_473_z1_st_YVRyUeqPPis2KF5M.cif	30	0.142
XXXII_structure_150.cif	XXXII-1-XtalPi-data_946_z1_st_YD8t7RjU-QABeTcL.cif	30	0.181
XXXII_structure_151.cif	XXXII-1-XtalPi-data_613_z1_st_YD8t7RjU-QABeTfx.cif	30	0.329
XXXII_structure_152.cif	XXXII-1-XtalPi-data_416_z1_st_YUl4CeqPPieGr_zZ.cif	30	0.138
XXXII_structure_153.cif	XXXII-1-XtalPi-data_805_z1_st_YD8t7RjU-QABeTe1.cif	30	0.147
ˆXXXII_structure_154.cif	ˆXXXII-1-XtalPi-data_598_z1_st_YD8t7RjU-QABeTdK.cif	30	0.300
XXXII_structure_155.cif	XXXII-1-XtalPi-data_504_z1_st_YVRyUeqPPis2KF3S.cif	30	0.223
XXXII_structure_156.cif	XXXII-1-XtalPi-data_1094_z1_st_YUl4CeqPPieGr_4P.cif	30	0.198
ˆXXXII_structure_157.cif	ˆXXXII-1-SLPrice-BXXXII_dfGAE30_GAE30.cif	30	0.494
XXXII_structure_158.cif	XXXII-1-XtalPi-data_943_z1_st_YUl4CeqPPieGr_1F.cif	30	0.259
XXXII_structure_159.cif	XXXII-1-XtalPi-data_829_z1_st_YUl4CeqPPieGr_zl.cif	30	0.178
XXXII_structure_160.cif	XXXII-1-XtalPi-data_840_z1_st_YD8t7RjU-QABeTdV.cif	30	0.233
XXXII_structure_161.cif	XXXII-1-XtalPi-data_665_z1_st_YUl4CeqPPieGr_ys.cif	30	0.191
XXXII_structure_162.cif	XXXII-1-XtalPi-data_1109_z1_st_YUl4CeqPPieGr_6R.cif	30	0.182
XXXII_structure_163.cif	XXXII-1-XtalPi-data_133_z1_st_YVRyUeqPPis2KF5h.cif	30	0.217
XXXII_structure_164.cif	XXXII-1-XtalPi-data_487_z1_st_YVRyUeqPPis2KF4V.cif	30	0.188
XXXII_structure_165.cif	XXXII-1-XtalPi-data_98_z1_st_YVRyUeqPPis2KF4z.cif	30	0.228
XXXII_structure_166.cif	XXXII-1-XtalPi-data_941_z1_st_YVRyUeqPPis2KF26.cif	30	0.283
XXXII_structure_167.cif	XXXII-1-XtalPi-data_654_z1_st_YUl4CeqPPieGr_3f.cif	30	0.299
XXXII_structure_168.cif	XXXII-1-XtalPi-data_1_z1_st_YD8t7RjU-QABeTaL.cif	30	0.310

^XXXII_structure_169.cif	^XXXII-1-MNeumann-structure_1405.cif	30	0.271
XXXII_structure_170.cif	XXXII-1-XtalPi-data_430_z1_st_YVRyUeqPPis2KF5k.cif	30	0.234
^XXXII_structure_171.cif	^XXXII-1-BEijck-data_vaneijck-XXXII.0663.cif	30	0.162
XXXII_structure_172.cif	XXXII-1-XtalPi-data_796_z1_st_YUl4CeqPPieGr_x9.cif	30	0.226
XXXII_structure_173.cif	XXXII-1-XtalPi-data_977_z1_st_YUl4CeqPPieGr_4X.cif	30	0.266
XXXII_structure_174.cif	XXXII-1-MNeumann-structure_345.cif	30	0.138
XXXII_structure_175.cif	XXXII-1-XtalPi-data_129_z1_st_YVRyUeqPPis2KF2b.cif	30	0.274
XXXII_structure_176.cif	XXXII-1-XtalPi-data_100_z1_st_YVRyUeqPPis2KF3C.cif	30	0.229
XXXII_structure_177.cif	XXXII-1-XtalPi-data_1169_z1_st_YXPXcuqPPis2KGVA.cif	30	0.436
XXXII_structure_178.cif	XXXII-1-XtalPi-data_1059_z1_st_YD8t7RjU-QABeTZz.cif	30	0.193
XXXII_structure_179.cif	XXXII-1-XtalPi-data_854_z1_st_YD8t7RjU-QABeTVo.cif	30	0.159
XXXII_structure_180.cif	XXXII-1-XtalPi-data_581_z1_st_YVRyUeqPPis2KF5i.cif	30	0.123
^XXXII_structure_181.cif	^XXXII-1-BEijck-data_vaneijck-XXXII.1063.cif	30	0.192
XXXII_structure_182.cif	XXXII-1-XtalPi-data_919_z1_st_YUl4CeqPPieGr_6Z.cif	30	0.204
XXXII_structure_183.cif	XXXII-1-XtalPi-data_964_z1_st_YUl4CeqPPieGr_0T.cif	30	0.212
XXXII_structure_184.cif	XXXII-1-XtalPi-data_764_z1_st_YUl4CeqPPieGr_4-.cif	30	0.207
XXXII_structure_185.cif	XXXII-1-MNeumann-structure_113.cif	30	0.258
XXXII_structure_186.cif	XXXII-1-XtalPi-data_724_z1_st_YVRyUeqPPis2KF3m.cif	30	0.197
XXXII_structure_187.cif	XXXII-1-MNeumann-structure_939.cif	30	0.246
XXXII_structure_188.cif	XXXII-1-MNeumann-structure_141.cif	30	0.307
XXXII_structure_189.cif	XXXII-1-MNeumann-structure_361.cif	30	0.265
XXXII_structure_190.cif	XXXII-1-MNeumann-structure_485.cif	30	0.278
XXXII_structure_191.cif	XXXII-1-XtalPi-data_1372_z1_st_YXPXcuqPPis2KGVP.cif	30	0.204
XXXII_structure_192.cif	XXXII-1-XtalPi-data_537_z1_st_YD8t7RjU-QABeTSH.cif	30	0.243
XXXII_structure_193.cif	XXXII-1-XtalPi-data_67_z1_st_YUl4CeqPPieGr_2-.cif	30	0.217
XXXII_structure_194.cif	XXXII-1-XtalPi-data_861_z1_st_YUl4CeqPPieGr_4L.cif	30	0.206
XXXII_structure_195.cif	XXXII-1-XtalPi-data_649_z1_st_YWlxjOqPPis2KF5q.cif	30	0.259
XXXII_structure_196.cif	XXXII-1-XtalPi-data_905_z1_st_YUl4CeqPPieGr_0a.cif	30	0.139
XXXII_structure_197.cif	XXXII-1-XtalPi-data_760_z1_st_YUl4CeqPPieGr_3n.cif	30	0.184
XXXII_structure_198.cif	XXXII-1-XtalPi-data_40_z1_st_YVRyUeqPPis2KF3h.cif	30	0.170
XXXII_structure_199.cif	XXXII-1-MNeumann-structure_446.cif	30	0.263
XXXII_structure_200.cif	XXXII-1-XtalPi-data_949_z1_st_YUl4CeqPPieGr_3x.cif	30	0.213
XXXII_structure_201.cif	XXXII-1-XtalPi-data_1179_z1_st_YXPXcuqPPis2KGVK.cif	30	0.226
XXXII_structure_202.cif	XXXII-1-XtalPi-data_1032_z1_st_YD8t7RjU-QABeTRM.cif	30	0.195
XXXII_structure_203.cif	XXXII-1-MNeumann-structure_1356.cif	30	0.124
XXXII_structure_204.cif	XXXII-1-XtalPi-data_894_z1_st_YD8t7RjU-QABeTcy.cif	30	0.154
XXXII_structure_205.cif	XXXII-1-XtalPi-data_429_z1_st_YD8t7RjU-QABeTV0.cif	30	0.240
XXXII_structure_206.cif	XXXII-1-XtalPi-data_668_z1_st_YML9yQwufAABcYYZ.cif	30	0.190
XXXII_structure_207.cif	XXXII-1-XtalPi-data_792_z1_st_YVRyUeqPPis2KF3R.cif	30	0.205
XXXII_structure_208.cif	XXXII-1-MNeumann-structure_1046.cif	30	0.155
XXXII_structure_209.cif	XXXII-1-XtalPi-data_904_z1_st_YUl4CeqPPieGr_0E.cif	30	0.166
XXXII_structure_210.cif	XXXII-1-XtalPi-data_1064_z1_st_YD8t7RjU-QABeTUo.cif	30	0.159
XXXII_structure_211.cif	XXXII-1-XtalPi-data_109_z1_st_YD8t7RjU-QABeTVV.cif	30	0.165
XXXII_structure_212.cif	XXXII-1-XtalPi-data_141_z1_st_YVRyUeqPPis2KF2c.cif	30	0.229
XXXII_structure_213.cif	XXXII-1-XtalPi-data_707_z1_st_YD8t7RjU-QABeTTr.cif	30	0.197
XXXII_structure_214.cif	XXXII-1-XtalPi-data_846_z1_st_YD8t7RjU-QABeTcw.cif	30	0.238
XXXII_structure_215.cif	XXXII-1-XtalPi-data_877_z1_st_YD8t7RjU-QABeTXB.cif	30	0.164
XXXII_structure_216.cif	XXXII-1-XtalPi-data_417_z1_st_YUl4CeqPPieGr_6H.cif	30	0.268
XXXII_structure_217.cif	XXXII-1-XtalPi-data_551_z1_st_YVRyUeqPPis2KF3z.cif	30	0.169
XXXII_structure_218.cif	XXXII-1-XtalPi-data_1110_z1_st_YML9yQwufAABcYZm.cif	30	0.222
XXXII_structure_219.cif	XXXII-1-XtalPi-data_804_z1_st_YD8t7RjU-QABeTdN.cif	30	0.199
XXXII_structure_220.cif	XXXII-1-XtalPi-data_998_z1_st_YD8t7RjU-QABeTec.cif	30	0.256
^XXXII_structure_221.cif	^XXXII-1-AOganov-data_1131.cif	30	0.252
XXXII_structure_222.cif	XXXII-1-XtalPi-data_951_z1_st_YD8t7RjU-QABeTdh.cif	30	0.202
XXXII_structure_223.cif	XXXII-1-XtalPi-data_969_z1_st_YD8t7RjU-QABeTXt.cif	30	0.179
XXXII_structure_224.cif	XXXII-1-XtalPi-data_834_z1_st_YUl4CeqPPieGr_zU.cif	30	0.173
XXXII_structure_225.cif	XXXII-1-XtalPi-data_844_z1_st_YD8t7RjU-QABeTgW.cif	30	0.171
XXXII_structure_226.cif	XXXII-1-MNeumann-structure_797.cif	30	0.135
XXXII_structure_227.cif	XXXII-1-XtalPi-data_925_z1_st_YD8t7RjU-QABeTZw.cif	30	0.157

XXXII_structure_228.cif	XXXII-1-XtalPi-data_403_z1_st_YVRyUeqPPis2KF5d.cif	30	0.141
XXXII_structure_229.cif	XXXII-1-XtalPi-data_198_z1_st_YVRyUeqPPis2KF4R.cif	30	0.168
XXXII_structure_230.cif	XXXII-1-XtalPi-data_1050_z1_st_YD8t7RjU-QABeTbR.cif	30	0.132
XXXII_structure_231.cif	XXXII-1-XtalPi-data_975_z1_st_YUl4CeqPPieGr_5-.cif	30	0.242
XXXII_structure_232.cif	*XXXII-1-MNeumann-structure_72.cif	30	0.186
XXXII_structure_233.cif	XXXII-1-XtalPi-data_906_z1_st_YD8t7RjU-QABeTfo.cif	30	0.234
XXXII_structure_234.cif	XXXII-1-XtalPi-data_741_z1_st_YD8t7RjU-QABeTa0.cif	30	0.156
XXXII_structure_235.cif	XXXII-1-XtalPi-data_1086_z1_st_YD8t7RjU-QABeTej.cif	30	0.214
XXXII_structure_236.cif	XXXII-1-XtalPi-data_21_z1_st_YD8t7RjU-QABeTPi.cif	30	0.181
XXXII_structure_237.cif	XXXII-1-MNeumann-structure_567.cif	30	0.267
XXXII_structure_238.cif	XXXII-1-XtalPi-data_344_z1_st_YXPXcuqPPis2KGUv.cif	30	0.236
^XXXII_structure_239.cif	^XXXII-1-CSAdjiman-CSOFM_00018.cif	30	0.219
XXXII_structure_240.cif	XXXII-1-XtalPi-data_177_z1_st_YVRyUeqPPis2KF4T.cif	30	0.327
XXXII_structure_241.cif	XXXII-1-XtalPi-data_973_z1_st_YD8t7RjU-QABeTS9.cif	30	0.150
XXXII_structure_242.cif	XXXII-1-XtalPi-data_1141_z1_st_YUl4CeqPPieGr_5f.cif	30	0.160
XXXII_structure_243.cif	XXXII-1-XtalPi-data_839_z1_st_YUl4CeqPPieGr_yY.cif	30	0.165
XXXII_structure_244.cif	XXXII-1-XtalPi-data_744_z1_st_YD8t7RjU-QABeTXR.cif	30	0.216
XXXII_structure_245.cif	XXXII-1-XtalPi-data_827_z1_st_YD8t7RjU-QABeTYC.cif	30	0.173
XXXII_structure_246.cif	XXXII-1-XtalPi-data_480_z1_st_YVRyUeqPPis2KF44.cif	30	0.187
XXXII_structure_247.cif	XXXII-1-XtalPi-data_547_z1_st_YD8t7RjU-QABeTcI.cif	30	0.193
XXXII_structure_248.cif	XXXII-1-XtalPi-data_1003_z1_st_YML9yQwufAABcYZM.cif	30	0.194
XXXII_structure_249.cif	XXXII-1-XtalPi-data_1004_z1_st_YD8t7RjU-QABeTRG.cif	30	0.187
XXXII_structure_250.cif	XXXII-1-MNeumann-structure_430.cif	30	0.248
XXXII_structure_251.cif	XXXII-1-XtalPi-data_262_z1_st_YVRyUeqPPis2KF41.cif	30	0.243
XXXII_structure_252.cif	XXXII-1-XtalPi-data_992_z1_st_YML9yQwufAABcYZv.cif	30	0.310
XXXII_structure_253.cif	XXXII-1-XtalPi-data_530_z1_st_YUl4CeqPPieGr_3i.cif	30	0.198
XXXII_structure_254.cif	XXXII-1-XtalPi-data_997_z1_st_YD8t7RjU-QABeTXf.cif	30	0.156
XXXII_structure_255.cif	XXXII-1-XtalPi-data_884_z1_st_YD8t7RjU-QABeTTw.cif	30	0.367
XXXII_structure_256.cif	XXXII-1-XtalPi-data_471_z1_st_YD8t7RjU-QABeTWE.cif	30	0.210
XXXII_structure_257.cif	XXXII-1-XtalPi-data_1075_z1_st_YD8t7RjU-QABeTap.cif	30	0.208
XXXII_structure_258.cif	XXXII-1-XtalPi-data_223_z1_st_YVRyUeqPPis2KF3N.cif	30	0.107
XXXII_structure_259.cif	XXXII-1-XtalPi-data_1249_z1_st_YXPXcuqPPis2KGUy.cif	30	0.224
XXXII_structure_260.cif	XXXII-1-XtalPi-data_549_z1_st_YUl4CeqPPieGr_2x.cif	30	0.174
XXXII_structure_261.cif	XXXII-1-XtalPi-data_428_z1_st_YD8t7RjU-QABeTfO.cif	30	0.225
XXXII_structure_262.cif	XXXII-1-XtalPi-data_849_z1_st_YD8t7RjU-QABeTZZ.cif	30	0.212
XXXII_structure_263.cif	XXXII-1-XtalPi-data_1084_z1_st_YD8t7RjU-QABeTPH.cif	30	0.146
XXXII_structure_264.cif	XXXII-1-XtalPi-data_738_z1_st_YML9yQwufAABcYXU.cif	30	0.243
^XXXII_structure_265.cif	^XXXII-1-AOganov-data_794.cif	30	0.238
XXXII_structure_266.cif	XXXII-1-XtalPi-data_1076_z1_st_YD8t7RjU-QABeTYG.cif	30	0.186
XXXII_structure_267.cif	XXXII-1-XtalPi-data_445_z1_st_YML9yQwufAABcYYr.cif	30	0.189
XXXII_structure_268.cif	XXXII-1-XtalPi-data_771_z1_st_YD8t7RjU-QABeTSC.cif	30	0.213
XXXII_structure_269.cif	XXXII-1-XtalPi-data_284_z1_st_YD8t7RjU-QABeTS2.cif	30	0.145
XXXII_structure_270.cif	XXXII-1-MNeumann-structure_35.cif	30	0.143
XXXII_structure_271.cif	XXXII-1-SLPrice-BXXXII_dfGAE24_GAE24.cif	30	0.517
XXXII_structure_272.cif	XXXII-1-XtalPi-data_596_z1_st_YVRyUeqPPis2KF46.cif	30	0.229
XXXII_structure_273.cif	XXXII-1-XtalPi-data_888_z1_st_YD8t7RjU-QABeTa1.cif	30	0.165
XXXII_structure_274.cif	XXXII-1-XtalPi-data_550_z1_st_YML9yQwufAABcYWf.cif	30	0.170
XXXII_structure_275.cif	XXXII-1-XtalPi-data_1019_z1_st_YD8t7RjU-QABeTdB.cif	30	0.120
XXXII_structure_276.cif	XXXII-1-XtalPi-data_1081_z1_st_YD8t7RjU-QABeTfy.cif	30	0.261
XXXII_structure_277.cif	XXXII-1-XtalPi-data_886_z1_st_YD8t7RjU-QABeTPK.cif	30	0.227
XXXII_structure_278.cif	XXXII-1-MNeumann-structure_1349.cif	30	0.161
XXXII_structure_279.cif	XXXII-1-XtalPi-data_1049_z1_st_YUl4CeqPPieGr_08.cif	30	0.170
XXXII_structure_280.cif	XXXII-1-XtalPi-data_923_z1_st_YD8t7RjU-QABeTXX.cif	30	0.310

XXXII_structure_281.cif	XXXII-1-XtalPi-data_1085_z1_st_YD8t7RjU-QABeTYx.cif	30	0.254
XXXII_structure_282.cif	XXXII-1-XtalPi-data_948_z1_st_YD8t7RjU-QABeTU0.cif	30	0.157
XXXII_structure_283.cif	XXXII-1-XtalPi-data_996_z1_st_YD8t7RjU-QABeTTo.cif	30	0.186
XXXII_structure_284.cif	XXXII-1-XtalPi-data_573_z1_st_YUl4CeqPPieGr_yy.cif	30	0.210
XXXII_structure_285.cif	XXXII-1-XtalPi-data_970_z1_st_YUl4CeqPPieGr_0O.cif	30	0.175
^XXXII_structure_286.cif	^XXXII-1-CSAdjiman-CSOFM_00716.cif	30	0.259
XXXII_structure_287.cif	XXXII-1-XtalPi-data_1012_z1_st_YUl4CeqPPieGr_3y.cif	30	0.222
XXXII_structure_288.cif	XXXII-1-XtalPi-data_673_z1_st_YD8t7RjU-QABeTUg.cif	30	0.190
XXXII_structure_289.cif	XXXII-1-XtalPi-data_1130_z1_st_YD8t7RjU-QABeTTE.cif	30	0.241
XXXII_structure_290.cif	XXXII-1-XtalPi-data_1147_z1_st_YD8t7RjU-QABeTU2.cif	30	0.213
XXXII_structure_291.cif	XXXII-1-XtalPi-data_291_z1_st_YVRyUeqPPis2KF3L.cif	30	0.267
XXXII_structure_292.cif	XXXII-1-XtalPi-data_1123_z1_st_YUl4CeqPPieGr_4u.cif	30	0.265
XXXII_structure_293.cif	XXXII-1-XtalPi-data_606_z1_st_YML9yQwufAABcYXI.cif	30	0.180
XXXII_structure_294.cif	XXXII-1-XtalPi-data_356_z1_st_YUl4CeqPPieGr_zg.cif	30	0.205
XXXII_structure_295.cif	XXXII-1-XtalPi-data_830_z1_st_YD8t7RjU-QABeTP5.cif	30	0.202
^XXXII_structure_296.cif	^XXXII-1-SLPrice-BXXXII_dfDDF11_DDF11.cif	30	0.232
XXXII_structure_297.cif	XXXII-1-XtalPi-data_1128_z1_st_YUl4CeqPPieGr_zD.cif	30	0.194
^XXXII_structure_298.cif	^XXXII-1-BEijck-data_vaneijck-XXXII.0973.cif	30	0.153
XXXII_structure_299.cif	XXXII-1-XtalPi-data_65_z1_st_YML9yQwufAABcYXp.cif	30	0.225
^XXXII_structure_300.cif	^XXXII-1-CShang-XXXII_730.cif	30	0.218
XXXII_structure_301.cif	XXXII-1-XtalPi-data_321_z1_st_YVRyUeqPPis2KF2a.cif	30	0.241
^XXXII_structure_302.cif	^XXXII-1-SLPrice-BXXXII_dfBAT24_BAT24.cif	30	0.181
XXXII_structure_303.cif	XXXII-1-XtalPi-data_799_z1_st_YML9yQwufAABcYaT.cif	30	0.199
XXXII_structure_304.cif	XXXII-1-XtalPi-data_208_z1_st_YUl4CeqPPieGr_5c.cif	30	0.086
XXXII_structure_305.cif	XXXII-1-XtalPi-data_1043_z1_st_YD8t7RjU-QABeTWk.cif	30	0.273
XXXII_structure_306.cif	XXXII-1-XtalPi-data_944_z1_st_YD8t7RjU-QABeTY0.cif	30	0.286
XXXII_structure_307.cif	XXXII-1-XtalPi-data_452_z1_st_YVRyUeqPPis2KF39.cif	30	0.181
XXXII_structure_308.cif	XXXII-1-MNeumann-structure_705.cif	30	0.231
XXXII_structure_309.cif	XXXII-1-XtalPi-data_1046_z1_st_YVRyUeqPPis2KF4e.cif	30	0.192
^XXXII_structure_310.cif	^XXXII-1-CSAdjiman-CSOFM_00216.cif	30	0.205
XXXII_structure_311.cif	XXXII-1-XtalPi-data_1087_z1_st_YD8t7RjU-QABeTNP.cif	30	0.196
XXXII_structure_312.cif	XXXII-1-XtalPi-data_639_z1_st_YD8t7RjU-QABeTZa.cif	30	0.270
XXXII_structure_313.cif	XXXII-1-XtalPi-data_1131_z1_st_YUl4CeqPPieGr_40.cif	30	0.211
XXXII_structure_314.cif	XXXII-1-XtalPi-data_982_z1_st_YD8t7RjU-QABeTZR.cif	30	0.255
XXXII_structure_315.cif	XXXII-1-MNeumann-structure_928.cif	30	0.160
XXXII_structure_316.cif	XXXII-1-XtalPi-data_909_z1_st_YD8t7RjU-QABeTZi.cif	30	0.244
XXXII_structure_317.cif	*XXXII-1-MNeumann-structure_159.cif	30	0.244
^XXXII_structure_318.cif	^XXXII-1-AOganov-data_837.cif	30	0.231
XXXII_structure_319.cif	XXXII-1-XtalPi-data_783_z1_st_YD8t7RjU-QABeTYb.cif	30	0.135
XXXII_structure_320.cif	XXXII-1-XtalPi-data_23_z1_st_YML9yQwufAABcYW4.cif	30	0.191
^XXXII_structure_321.cif	^XXXII-1-AOganov-data_1221.cif	30	0.289
XXXII_structure_322.cif	XXXII-1-MNeumann-structure_1009.cif	30	0.138
XXXII_structure_323.cif	XXXII-1-MNeumann-structure_1143.cif	30	0.208
XXXII_structure_324.cif	XXXII-1-XtalPi-data_954_z1_st_YUl4CeqPPieGr_zM.cif	30	0.157
XXXII_structure_325.cif	XXXII-1-XtalPi-data_748_z1_st_YVRyUeqPPis2KF4l.cif	30	0.147
XXXII_structure_326.cif	XXXII-1-MNeumann-structure_1456.cif	30	0.290
XXXII_structure_327.cif	XXXII-1-XtalPi-data_915_z1_st_YD8t7RjU-QABeTfJ.cif	30	0.208
XXXII_structure_328.cif	XXXII-1-XtalPi-data_635_z1_st_YML9yQwufAABcYXH.cif	30	0.249
XXXII_structure_329.cif	XXXII-1-XtalPi-data_753_z1_st_YVRyUeqPPis2KF4f.cif	30	0.208
XXXII_structure_330.cif	XXXII-1-XtalPi-data_1053_z1_st_YUl4CeqPPieGr_00.cif	30	0.129
XXXII_structure_331.cif	XXXII-1-XtalPi-data_12_z1_st_YVRyUeqPPis2KF5L.cif	30	0.182
XXXII_structure_332.cif	XXXII-1-XtalPi-data_500_z1_st_YD8t7RjU-QABeTTK.cif	30	0.151
XXXII_structure_333.cif	XXXII-1-XtalPi-data_1113_z1_st_YD8t7RjU-QABeTO_.cif	30	0.178
XXXII_structure_334.cif	XXXII-1-XtalPi-data_28_z1_st_YVRyUeqPPis2KF3l.cif	30	0.166
XXXII_structure_335.cif	XXXII-1-XtalPi-data_298_z1_st_YUl4CeqPPieGr_3A.cif	30	0.222
XXXII_structure_336.cif	XXXII-1-XtalPi-data_1017_z1_st_YUl4CeqPPieGr_3Z.cif	30	0.199
XXXII_structure_337.cif	XXXII-1-XtalPi-data_979_z1_st_YUl4CeqPPieGr_4h.cif	30	0.229
XXXII_structure_338.cif	XXXII-1-XtalPi-data_865_z1_st_YD8t7RjU-QABeTcz.cif	30	0.164
XXXII_structure_339.cif	XXXII-1-XtalPi-data_734_z1_st_YD8t7RjU-QABeTS0.cif	30	0.181

ˆXXXII_structure_340.cif	ˆXXXII-1-AOganov-data_1322.cif	30	0.217
XXXII_structure_341.cif	XXXII-1-XtalPi-data_575_z1_st_YD8t7RjU-QABeTVa.cif	30	0.196
XXXII_structure_342.cif	XXXII-1-MNeumann-structure_59.cif	30	0.276
XXXII_structure_343.cif	XXXII-1-XtalPi-data_1118_z1_st_YD8t7RjU-QABeTaY.cif	30	0.260
XXXII_structure_344.cif	XXXII-1-XtalPi-data_77_z1_st_YVRyUeqPPis2KF3V.cif	30	0.174
XXXII_structure_345.cif	XXXII-1-MNeumann-structure_1236.cif	30	0.189
XXXII_structure_346.cif	XXXII-1-XtalPi-data_871_z1_st_YVRyUeqPPis2KF2_.cif	30	0.190
XXXII_structure_347.cif	XXXII-1-MNeumann-structure_1061.cif	30	0.202
XXXII_structure_348.cif	XXXII-1-MNeumann-structure_1204.cif	30	0.161
ˆXXXII_structure_349.cif	ˆXXXII-1-KSzalewicz-data_294.cif	30	0.231
XXXII_structure_350.cif	XXXII-1-XtalPi-data_953_z1_st_YD8t7RjU-QABeTfg.cif	30	0.187
XXXII_structure_351.cif	XXXII-1-MNeumann-structure_1076.cif	30	0.165
ˆXXXII_structure_352.cif	ˆXXXII-1-CSAdjiman-CSOFM_00744.cif	30	0.257
ˆXXXII_structure_353.cif	ˆXXXII-1-AOganov-data_339.cif	30	0.240
XXXII_structure_354.cif	XXXII-1-XtalPi-data_1060_z1_st_YD8t7RjU-QABeTO7.cif	30	0.168
XXXII_structure_355.cif	XXXII-1-XtalPi-data_751_z1_st_YML9yQwufAABcYX2.cif	30	0.184
ˆXXXII_structure_356.cif	ˆXXXII-1-QZhu-data_1210-SG-14.cif	30	0.178
ˆXXXII_structure_357.cif	ˆXXXII-1-BEijck-data_vaneijck-XXXII.1045.cif	30	0.178
ˆXXXII_structure_358.cif	ˆXXXII-1-QZhu-data_785-SG-4.cif	30	0.222
XXXII_structure_359.cif	XXXII-1-XtalPi-data_855_z1_st_YML9yQwufAABcYaQ.cif	30	0.194
XXXII_structure_360.cif	XXXII-1-XtalPi-data_482_z1_st_YML9yQwufAABcYZE.cif	30	0.151
XXXII_structure_361.cif	XXXII-1-XtalPi-data_170_z1_st_YML9yQwufAABcYXv.cif	30	0.176
XXXII_structure_362.cif	XXXII-1-XtalPi-data_887_z1_st_YD8t7RjU-QABeTOI.cif	30	0.190
XXXII_structure_363.cif	XXXII-1-XtalPi-data_862_z1_st_YD8t7RjU-QABeTdo.cif	30	0.197
XXXII_structure_364.cif	XXXII-1-MNeumann-structure_496.cif	30	0.199
XXXII_structure_365.cif	XXXII-1-XtalPi-data_780_z1_st_YUl4CeqPPieGr_zr.cif	30	0.141
XXXII_structure_366.cif	XXXII-1-XtalPi-data_454_z1_st_YVRyUeqPPis2KF5Z.cif	30	0.269
XXXII_structure_367.cif	XXXII-1-MNeumann-structure_505.cif	30	0.174
XXXII_structure_368.cif	XXXII-1-XtalPi-data_1124_z1_st_YD8t7RjU-QABeTTU.cif	30	0.127
XXXII_structure_369.cif	XXXII-1-XtalPi-data_644_z1_st_YVRyUeqPPis2KF3n.cif	30	0.113
ˆXXXII_structure_370.cif	ˆXXXII-1-SLPrice-BXXXII_dfJAA25_JAA25.cif	30	0.218
XXXII_structure_371.cif	XXXII-1-XtalPi-data_742_z1_st_YVRyUeqPPis2KF4P.cif	30	0.135
XXXII_structure_372.cif	XXXII-1-XtalPi-data_462_z1_st_YVRyUeqPPis2KF49.cif	30	0.245
XXXII_structure_373.cif	XXXII-1-XtalPi-data_858_z1_st_YVRyUeqPPis2KF3M.cif	30	0.167
XXXII_structure_374.cif	XXXII-1-XtalPi-data_933_z1_st_YD8t7RjU-QABeTak.cif	30	0.156
XXXII_structure_375.cif	XXXII-1-XtalPi-data_525_z1_st_YD8t7RjU-QABeTPa.cif	30	0.205
XXXII_structure_376.cif	XXXII-1-XtalPi-data_47_z1_st_YVRyUeqPPis2KF5R.cif	30	0.217
XXXII_structure_377.cif	XXXII-1-MNeumann-structure_865.cif	30	0.193
XXXII_structure_378.cif	XXXII-1-XtalPi-data_1154_z1_st_YD8t7RjU-QABeTUM.cif	30	0.228
XXXII_structure_379.cif	XXXII-1-XtalPi-data_520_z1_st_YD8t7RjU-QABeTaR.cif	30	0.173
XXXII_structure_380.cif	XXXII-1-XtalPi-data_787_z1_st_YUl4CeqPPieGr_2Y.cif	30	0.207
XXXII_structure_381.cif	XXXII-1-XtalPi-data_467_z1_st_YML9yQwufAABcYY4.cif	30	0.179
XXXII_structure_382.cif	XXXII-1-XtalPi-data_242_z1_st_YML9yQwufAABcYW5.cif	30	0.270
XXXII_structure_383.cif	XXXII-1-MNeumann-structure_1199.cif	30	0.154
XXXII_structure_384.cif	XXXII-1-XtalPi-data_863_z1_st_YD8t7RjU-QABeTb0.cif	30	0.223
XXXII_structure_385.cif	XXXII-1-XtalPi-data_1126_z1_st_YUl4CeqPPieGr_zX.cif	30	0.230
XXXII_structure_386.cif	XXXII-1-XtalPi-data_961_z1_st_YD8t7RjU-QABeTNx.cif	30	0.224
XXXII_structure_387.cif	XXXII-1-XtalPi-data_287_z1_st_YD8t7RjU-QABeTPO.cif	30	0.137
XXXII_structure_388.cif	XXXII-1-XtalPi-data_3_z1_st_YD8t7RjU-QABeTXa.cif	30	0.255
XXXII_structure_389.cif	XXXII-1-XtalPi-data_528_z1_st_YD8t7RjU-QABeTUf.cif	30	0.242
XXXII_structure_390.cif	XXXII-1-XtalPi-data_1272_z1_st_YXPXcuqPPis2KGUo.cif	30	0.300
XXXII_structure_391.cif	XXXII-1-XtalPi-data_991_z1_st_YML9yQwufAABcYXu.cif	30	0.313
XXXII_structure_392.cif	XXXII-1-XtalPi-data_1144_z1_st_YUl4CeqPPieGr_30.cif	30	0.229
XXXII_structure_393.cif	XXXII-1-XtalPi-data_280_z1_st_YUl4CeqPPieGr_3X.cif	30	0.164
XXXII_structure_394.cif	XXXII-1-XtalPi-data_624_z1_st_YD8t7RjU-QABeTWv.cif	30	0.189
XXXII_structure_395.cif	XXXII-1-XtalPi-data_893_z1_st_YD8t7RjU-QABeTdj.cif	30	0.203
XXXII_structure_396.cif	XXXII-1-XtalPi-data_848_z1_st_YUl4CeqPPieGr_5k.cif	30	0.194
XXXII_structure_397.cif	XXXII-1-XtalPi-data_851_z1_st_YD8t7RjU-QABeTZg.cif	30	0.178
XXXII_structure_398.cif	XXXII-1-XtalPi-data_1116_z1_st_YD8t7RjU-QABeTe3.cif	30	0.159

XXXII_structure_399.cif	XXXII-1-MNeumann-structure_650.cif	30	0.204
XXXII_structure_400.cif	XXXII-1-XtalPi-data_509_z1_st_YVRyUeqPPis2KF25.cif	30	0.180
^XXXII_structure_401.cif	^XXXII-1-AOganov-data_402.cif	30	0.286
XXXII_structure_402.cif	XXXII-1-XtalPi-data_929_z1_st_YD8t7RjU-QABeTb3.cif	30	0.148
XXXII_structure_403.cif	XXXII-1-XtalPi-data_664_z1_st_YUl4CeqPPieGr_49.cif	30	0.164
XXXII_structure_404.cif	XXXII-1-XtalPi-data_746_z1_st_YML9yQwufAABcYZL.cif	30	0.125
XXXII_structure_405.cif	XXXII-1-XtalPi-data_960_z1_st_YD8t7RjU-QABeTQ4.cif	30	0.174
XXXII_structure_406.cif	XXXII-1-XtalPi-data_1105_z1_st_YUl4CeqPPieGr_ye.cif	30	0.035
XXXII_structure_407.cif	XXXII-1-XtalPi-data_1005_z1_st_YD8t7RjU-QABeTe8.cif	30	0.161
XXXII_structure_408.cif	XXXII-1-XtalPi-data_705_z1_st_YUl4CeqPPieGr_0-.cif	30	0.205
^XXXII_structure_409.cif	^XXXII-1-CSAdjiman-CSOFM_00178.cif	30	0.250
XXXII_structure_410.cif	XXXII-1-XtalPi-data_900_z1_st_YVRyUeqPPis2KF4d.cif	30	0.159
XXXII_structure_411.cif	XXXII-1-MNeumann-structure_871.cif	30	0.230
XXXII_structure_412.cif	XXXII-1-XtalPi-data_564_z1_st_YD8t7RjU-QABeTW-.cif	30	0.132
XXXII_structure_413.cif	XXXII-1-XtalPi-data_1074_z1_st_YUl4CeqPPieGr_zE.cif	30	0.174
XXXII_structure_414.cif	XXXII-1-XtalPi-data_728_z1_st_YD8t7RjU-QABeTWG.cif	30	0.243
XXXII_structure_415.cif	XXXII-1-XtalPi-data_367_z1_st_YVRyUeqPPis2KF3u.cif	30	0.156
XXXII_structure_416.cif	XXXII-1-XtalPi-data_123_z1_st_YD8t7RjU-QABeTO3.cif	30	0.214
XXXII_structure_417.cif	XXXII-1-XtalPi-data_1030_z1_st_YD8t7RjU-QABeTYI.cif	30	0.158
XXXII_structure_418.cif	XXXII-1-XtalPi-data_872_z1_st_YD8t7RjU-QABeTUP.cif	30	0.243
XXXII_structure_419.cif	XXXII-1-XtalPi-data_682_z1_st_YD8t7RjU-QABeTWY.cif	30	0.239
XXXII_structure_420.cif	XXXII-1-XtalPi-data_838_z1_st_YVRyUeqPPis2KF3G.cif	30	0.192
^XXXII_structure_421.cif	^XXXII-1-AOganov-data_60.cif	30	0.243
XXXII_structure_422.cif	XXXII-1-XtalPi-data_994_z1_st_YD8t7RjU-QABeTgH.cif	30	0.138
XXXII_structure_423.cif	XXXII-1-MNeumann-structure_2.cif	30	0.207
XXXII_structure_424.cif	XXXII-1-MNeumann-structure_704.cif	30	0.235
^XXXII_structure_425.cif	^XXXII-1-SLPrice-BXXXII_dfGAE35_GAE35.cif	30	0.235
XXXII_structure_426.cif	XXXII-1-XtalPi-data_1151_z1_st_YD8t7RjU-QABeTTj.cif	30	0.206
XXXII_structure_427.cif	XXXII-1-XtalPi-data_338_z1_st_YVRyUeqPPis2KF27.cif	30	0.131
XXXII_structure_428.cif	XXXII-1-XtalPi-data_1045_z1_st_YUl4CeqPPieGr_3G.cif	30	0.208
XXXII_structure_429.cif	XXXII-1-XtalPi-data_1033_z1_st_YD8t7RjU-QABeTNN.cif	30	0.240
XXXII_structure_430.cif	XXXII-1-XtalPi-data_135_z1_st_YML9yQwufAABcYXY.cif	30	0.199
XXXII_structure_431.cif	XXXII-1-XtalPi-data_593_z1_st_YUl4CeqPPieGr_3w.cif	30	0.151
XXXII_structure_432.cif	XXXII-1-XtalPi-data_727_z1_st_YUl4CeqPPieGr_6Q.cif	30	0.180
XXXII_structure_433.cif	XXXII-1-XtalPi-data_483_z1_st_YVRyUeqPPis2KF4_.cif	30	0.161
XXXII_structure_434.cif	XXXII-1-XtalPi-data_104_z1_st_YVRyUeqPPis2KF4v.cif	30	0.204
XXXII_structure_435.cif	XXXII-1-XtalPi-data_1083_z1_st_YD8t7RjU-QABeTYo.cif	30	0.176
XXXII_structure_436.cif	XXXII-1-XtalPi-data_110_z1_st_YML9yQwufAABcYaI.cif	30	0.238
^XXXII_structure_437.cif	^XXXII-1-QZhu-data_208-SG-7.cif	30	0.121
XXXII_structure_438.cif	XXXII-1-XtalPi-data_731_z1_st_YD8t7RjU-QABeTZc.cif	30	0.223
XXXII_structure_439.cif	XXXII-1-XtalPi-data_422_z1_st_YML9yQwufAABcYXy.cif	30	0.199
XXXII_structure_440.cif	XXXII-1-MNeumann-structure_262.cif	30	0.124
XXXII_structure_441.cif	XXXII-1-XtalPi-data_750_z1_st_YVRyUeqPPis2KF48.cif	30	0.122
^XXXII_structure_442.cif	^XXXII-1-MNeumann-structure_664.cif	30	0.215
^XXXII_structure_443.cif	^XXXII-1-QZhu-data_408-SG-14.cif	30	0.204
XXXII_structure_444.cif	XXXII-1-XtalPi-data_1114_z1_st_YD8t7RjU-QABeTPu.cif	30	0.198
XXXII_structure_445.cif	XXXII-1-MNeumann-structure_775.cif	30	0.339
XXXII_structure_446.cif	XXXII-1-XtalPi-data_51_z1_st_YVRyUeqPPis2KF5A.cif	30	0.167
XXXII_structure_447.cif	XXXII-1-XtalPi-data_1051_z1_st_YD8t7RjU-QABeTeo.cif	30	0.167
XXXII_structure_448.cif	XXXII-1-XtalPi-data_27_z1_st_YVRyUeqPPis2KF4M.cif	30	0.203
XXXII_structure_449.cif	XXXII-1-XtalPi-data_828_z1_st_YD8t7RjU-QABeTeg.cif	30	0.239
XXXII_structure_450.cif	XXXII-1-XtalPi-data_720_z1_st_YVRyUeqPPis2KF4b.cif	30	0.126
XXXII_structure_451.cif	XXXII-1-XtalPi-data_1029_z1_st_YUl4CeqPPieGr_55.cif	30	0.274
XXXII_structure_452.cif	XXXII-1-XtalPi-data_1127_z1_st_YUl4CeqPPieGr_3j.cif	30	0.239
XXXII_structure_453.cif	XXXII-1-XtalPi-data_867_z1_st_YD8t7RjU-QABeTcf.cif	30	0.229
XXXII_structure_454.cif	XXXII-1-XtalPi-data_795_z1_st_YD8t7RjU-QABeTbu.cif	30	0.256
XXXII_structure_455.cif	XXXII-1-MNeumann-structure_342.cif	30	0.173
XXXII_structure_456.cif	XXXII-1-XtalPi-data_1080_z1_st_YD8t7RjU-QABeTdQ.cif	30	0.128

XXXII_structure_457.cif	XXXII-1-XtalPi-data_853_z1_st_YML9yQwufAABcYYq.cif	30	0.354
XXXII_structure_458.cif	XXXII-1-XtalPi-data_1492_z1_st_YXPXcuqPPis2KGVZ.cif	30	0.163
XXXII_structure_459.cif	XXXII-1-XtalPi-data_1104_z1_st_YD8t7RjU-QABeTUZ.cif	30	0.141
XXXII_structure_460.cif	XXXII-1-XtalPi-data_1037_z1_st_YUl4CeqPPieGr_5R.cif	30	0.158
XXXII_structure_461.cif	XXXII-1-XtalPi-data_459_z1_st_YVRyUeqPPis2KF5N.cif	30	0.214
XXXII_structure_462.cif	XXXII-1-XtalPi-data_1093_z1_st_YD8t7RjU-QABeTV5.cif	30	0.165
XXXII_structure_463.cif	XXXII-1-XtalPi-data_1120_z1_st_YUl4CeqPPieGr_42.cif	30	0.185
XXXII_structure_464.cif	XXXII-1-XtalPi-data_491_z1_st_YD8t7RjU-QABeTZS.cif	30	0.195
XXXII_structure_465.cif	XXXII-1-XtalPi-data_958_z1_st_YXPXcuqPPis2KGVb.cif	30	0.199
XXXII_structure_466.cif	XXXII-1-XtalPi-data_604_z1_st_YUl4CeqPPieGr_yT.cif	30	0.151
XXXII_structure_467.cif	XXXII-1-XtalPi-data_938_z1_st_YML9yQwufAABcYWn.cif	30	0.214
XXXII_structure_468.cif	XXXII-1-XtalPi-data_995_z1_st_YD8t7RjU-QABeTVH.cif	30	0.300
XXXII_structure_469.cif	XXXII-1-XtalPi-data_583_z1_st_YD8t7RjU-QABeTXQ.cif	30	0.134
ˆXXXII_structure_470.cif	ˆXXXII-1-CSAdjiman-CSOFM_00578.cif	30	0.320
XXXII_structure_471.cif	XXXII-1-XtalPi-data_676_z1_st_YD8t7RjU-QABeTYE.cif	30	0.232
XXXII_structure_472.cif	XXXII-1-XtalPi-data_1140_z1_st_YUl4CeqPPieGr_zv.cif	30	0.181
XXXII_structure_473.cif	XXXII-1-XtalPi-data_812_z1_st_YD8t7RjU-QABeTcD.cif	30	0.321
XXXII_structure_474.cif	XXXII-1-XtalPi-data_690_z1_st_YVRyUeqPPis2KF21.cif	30	0.257
XXXII_structure_475.cif	XXXII-1-XtalPi-data_814_z1_st_YD8t7RjU-QABeTUS.cif	30	0.237
XXXII_structure_476.cif	XXXII-1-XtalPi-data_939_z1_st_YUl4CeqPPieGr_1U.cif	30	0.187
XXXII_structure_477.cif	XXXII-1-XtalPi-data_1058_z1_st_YML9yQwufAABcYYD.cif	30	0.174
XXXII_structure_478.cif	XXXII-1-XtalPi-data_1446_z1_st_YXPXcuqPPis2KGVc.cif	30	0.163
XXXII_structure_479.cif	XXXII-1-XtalPi-data_716_z1_st_YVRyUeqPPis2KF38.cif	30	0.248
XXXII_structure_480.cif	XXXII-1-XtalPi-data_518_z1_st_YD8t7RjU-QABeTTX.cif	30	0.214
XXXII_structure_481.cif	XXXII-1-XtalPi-data_485_z1_st_YUl4CeqPPieGr_5W.cif	30	0.204
XXXII_structure_482.cif	XXXII-1-XtalPi-data_255_z1_st_YML9yQwufAABcYZR.cif	30	0.170
XXXII_structure_483.cif	XXXII-1-XtalPi-data_966_z1_st_YUl4CeqPPieGr_3B.cif	30	0.165
XXXII_structure_484.cif	XXXII-1-XtalPi-data_631_z1_st_YD8t7RjU-QABeTZG.cif	30	0.213
ˆXXXII_structure_485.cif	ˆXXXII-1-XtalPi-data_492_z1_st_YD8t7RjU-QABeTau.cif	30	0.266
XXXII_structure_486.cif	XXXII-1-XtalPi-data_937_z1_st_YD8t7RjU-QABeTZb.cif	30	0.175
XXXII_structure_487.cif	XXXII-1-XtalPi-data_852_z1_st_YD8t7RjU-QABeTge.cif	30	0.179
ˆXXXII_structure_488.cif	ˆXXXII-1-MNeumann-structure_1403.cif	30	0.249
XXXII_structure_489.cif	XXXII-1-XtalPi-data_7_z1_st_YVRyUeqPPis2KF2d.cif	30	0.165
XXXII_structure_490.cif	XXXII-1-XtalPi-data_952_z1_st_YML9yQwufAABcYWr.cif	30	0.148
XXXII_structure_491.cif	XXXII-1-XtalPi-data_1240_z1_st_YXPXcuqPPis2KGVF.cif	30	0.155
XXXII_structure_492.cif	XXXII-1-XtalPi-data_1314_z1_st_YXPXcuqPPis2KGVV.cif	30	0.147
XXXII_structure_493.cif	XXXII-1-XtalPi-data_955_z1_st_YD8t7RjU-QABeTNS.cif	30	0.222
XXXII_structure_494.cif	XXXII-1-XtalPi-data_927_z1_st_YUl4CeqPPieGr_3R.cif	30	0.246
XXXII_structure_495.cif	XXXII-1-XtalPi-data_460_z1_st_YVRyUeqPPis2KF3Z.cif	30	0.175
XXXII_structure_496.cif	XXXII-1-XtalPi-data_911_z1_st_YUl4CeqPPieGr_6Y.cif	30	0.169
XXXII_structure_497.cif	XXXII-1-MNeumann-structure_1124.cif	30	0.240
ˆXXXII_structure_498.cif	ˆXXXII-1-AOganov-data_1159.cif	30	0.257
XXXII_structure_499.cif	XXXII-1-XtalPi-data_585_z1_st_YD8t7RjU-QABeTPr.cif	30	0.197
XXXII_structure_500.cif	XXXII-1-MNeumann-structure_60.cif	30	0.220

Table 7: (Target XXXIII) The CIF names of the CCDC-prepared structures provided to participants for the structure ranking exercise, the corresponding CIF sampled from the first phase (structure generation exercise) of the seventh blind test, and the results (number of molecules matched in a cluster of 30 and RMSD) from COMPACK comparisons (applying 25% and 25° distance/angle tolerances) between the two to assess the structural change resultant from constrained optimisations carried out by the organisers in the structure list preparation. (* indicates a structure representing experimental Form A of XXVIII.) (Originating CIF names contain an added prefix of the form '{target}-{phase}-{group name/label}-')

CCDC-prepared CIF name	Originating CIF name	Molecules matched	RMSD (Å)
XXXIII_structure_1.cif	XXXIII-1-MNeumann-structure_1206.cif	30	0.361
XXXIII_structure_2.cif	XXXIII-1-MNeumann-structure_165.cif	30	0.729
XXXIII_structure_3.cif	XXXIII-1-XtalPi-data_512_Z1_st_YD2kLi1FJgABE1KI.cif	30	0.426
XXXIII_structure_4.cif	XXXIII-1-GDay-33_XXXIII_a_0_opt.33_XXXIII_c_0_opt-QRBH-14-28167-3-1_pl.cif	30	0.689
XXXIII_structure_5.cif	XXXIII-1-GDay-33_XXXIII_a_0_opt.33_XXXIII_c_0_opt-QRBH-14-13709-3-0.cif	30	0.478
XXXIII_structure_6.cif	XXXIII-1-GDay-33_XXXIII_a_0_opt.33_XXXIII_c_0_opt-QRBH-19-20832-3-0.cif	30	0.462
XXXIII_structure_7.cif	XXXIII-1-GDay-33_XXXIII_a_8_opt.33_XXXIII_c_0_opt-QRBH-19-21237-3-0.cif	30	0.646
XXXIII_structure_8.cif	XXXIII-1-GDay-33_XXXIII_a_2_opt.33_XXXIII_c_0_opt-QRBH-14-9744-3-2.cif	30	0.538
XXXIII_structure_9.cif	XXXIII-1-MNeumann-structure_1195.cif	30	0.346
XXXIII_structure_10.cif	XXXIII-1-GDay-33_XXXIII_a_0_opt.33_XXXIII_c_0_opt-QRBH-14-800-3-3_pl.cif	30	0.582
XXXIII_structure_11.cif	XXXIII-1-GDay-33_XXXIII_a_0_opt.33_XXXIII_c_0_opt-QRBH-14-4685-3-0.cif	23	0.595
XXXIII_structure_12.cif	XXXIII-1-SLPrice-XXXIII_dfBb414_Bb414.cif	30	0.376
XXXIII_structure_13.cif	XXXIII-1-GDay-33_XXXIII_a_10_opt.33_XXXIII_c_0_opt-QRBH-14-44892-3-0.cif	30	0.387
XXXIII_structure_14.cif	XXXIII-1-GDay-33_XXXIII_a_0_opt.33_XXXIII_c_0_opt-QRBH-2-19936-3-0.cif	23	0.700
XXXIII_structure_15.cif	XXXIII-1-GDay-33_XXXIII_a_0_opt.33_XXXIII_c_0_opt-QRBH-14-9644-3-0.cif	26	0.476
XXXIII_structure_16.cif	XXXIII-1-GDay-33_XXXIII_a_0_opt.33_XXXIII_c_0_opt-QRBH-2-34059-3-0.cif	23	0.290
XXXIII_structure_17.cif	XXXIII-1-XtalPi-data_247_Z1_st_YD2kLi1FJgABE1Wb.cif	30	0.267
XXXIII_structure_18.cif	XXXIII-1-GDay-33_XXXIII_a_2_opt.33_XXXIII_c_0_opt-QRBH-19-164-3-1.cif	30	0.544
XXXIII_structure_19.cif	XXXIII-1-GDay-33_XXXIII_a_6_opt.33_XXXIII_c_0_opt-QRBH-14-47424-3-0.cif	30	0.324
XXXIII_structure_20.cif	XXXIII-1-GDay-33_XXXIII_a_0_opt.33_XXXIII_c_0_opt-QRBH-2-2936-3-8.cif	30	0.539
XXXIII_structure_21.cif	XXXIII-1-GDay-33_XXXIII_a_0_opt.33_XXXIII_c_0_opt-QRBH-14-46-3-13.cif	25	0.470
XXXIII_structure_22.cif	XXXIII-1-GDay-33_XXXIII_a_0_opt.33_XXXIII_c_0_opt-QRBH-15-55-3-77.cif	30	0.514
XXXIII_structure_23.cif	XXXIII-1-GDay-33_XXXIII_a_10_opt.33_XXXIII_c_0_opt-QRBH-14-47004-3-2.cif	30	0.598
XXXIII_structure_24.cif	XXXIII-1-SLPrice-XXXIII_dfBa457_Ba457.cif	30	0.580
XXXIII_structure_25.cif	XXXIII-1-GDay-33_XXXIII_a_0_opt.33_XXXIII_c_0_opt-QRBH-14-800-3-3.cif	25	0.576
XXXIII_structure_26.cif	XXXIII-1-GDay-33_XXXIII_a_16_opt.33_XXXIII_c_0_opt-QRBH-33-256-3-2_pl.cif	30	0.485
XXXIII_structure_27.cif	XXXIII-1-GDay-33_XXXIII_a_0_opt.33_XXXIII_c_0_opt-QRBH-2-21117-3-0.cif	30	0.546
XXXIII_structure_28.cif	XXXIII-1-GDay-33_XXXIII_a_0_opt.33_XXXIII_c_0_opt-QRBH-2-563-3-1.cif	26	0.643
XXXIII_structure_29.cif	XXXIII-1-MNeumann-structure_1240.cif	30	0.279
XXXIII_structure_30.cif	XXXIII-1-XtalPi-data_434_Z1_st_YUW1ABsYUp3Zdb0f.cif	30	0.340

XXXIII_structure_31.cif	XXXIII-1-GDay-33_XXXIII_a_10_opt.33_XXXIII_c_0_opt-QRBH-19-18713-3-0.cif	30	0.430
XXXIII_structure_32.cif	XXXIII-1-GDay-33_XXXIII_a_6_opt.33_XXXIII_c_0_opt-QRBH-14-2690-3-17.cif	30	0.491
XXXIII_structure_33.cif	XXXIII-1-XtalPi-data_307_Z1_st_YD2kLi1FJgABE1Cq.cif	30	0.439
XXXIII_structure_34.cif	XXXIII-1-SLPrice-XXXIII_dfBa28_Ba28.cif	30	0.591
XXXIII_structure_35.cif	XXXIII-1-GDay-33_XXXIII_a_0_opt.33_XXXIII_c_0_opt-QRBH-4-11138-3-0.cif	30	0.412
XXXIII_structure_36.cif	XXXIII-1-XtalPi-data_44_Z1_st_YD2kLi1FJgABE1KR.cif	30	0.485
XXXIII_structure_37.cif	XXXIII-1-GDay-33_XXXIII_a_0_opt.33_XXXIII_c_0_opt-QRBH-1-972-3-0.cif	16	0.970
XXXIII_structure_38.cif	XXXIII-1-GDay-33_XXXIII_a_0_opt.33_XXXIII_c_0_opt-QRBH-14-40797-3-0.cif	30	0.360
XXXIII_structure_39.cif	XXXIII-1-SLPrice-XXXIII_dfBb619_Bb619.cif	30	0.504
XXXIII_structure_40.cif	XXXIII-1-GDay-33_XXXIII_a_0_opt.33_XXXIII_c_0_opt-QRBH-14-135-3-17.cif	24	0.643
XXXIII_structure_41.cif	XXXIII-1-GDay-33_XXXIII_a_6_opt.33_XXXIII_c_0_opt-QRBH-61-471-3-15.cif	30	0.541
XXXIII_structure_42.cif	XXXIII-1-MNeumann-structure_691.cif	30	0.414
XXXIII_structure_43.cif	XXXIII-1-MNeumann-structure_1014.cif	30	0.303
XXXIII_structure_44.cif	XXXIII-1-XtalPi-data_1292_Z1_st_YD2kLi1FJgABE1JX.cif	30	0.316
XXXIII_structure_45.cif	XXXIII-1-GDay-33_XXXIII_a_6_opt.33_XXXIII_c_0_opt-QRBH-61-38-3-58.cif	24	0.698
XXXIII_structure_46.cif	XXXIII-1-MNeumann-structure_246.cif	30	0.386
XXXIII_structure_47.cif	XXXIII-1-GDay-33_XXXIII_a_8_opt.33_XXXIII_c_0_opt-QRBH-14-1847-3-6.cif	30	0.409
XXXIII_structure_48.cif	XXXIII-1-MNeumann-structure_556.cif	30	0.554
XXXIII_structure_49.cif	XXXIII-1-GDay-33_XXXIII_a_10_opt.33_XXXIII_c_0_opt-QRBH-33-458-3-5.cif	30	0.379
XXXIII_structure_50.cif	XXXIII-1-GDay-33_XXXIII_a_6_opt.33_XXXIII_c_0_opt-QRBH-19-16429-3-0.cif	30	0.454
XXXIII_structure_51.cif	XXXIII-1-GDay-33_XXXIII_a_15_opt.33_XXXIII_c_0_opt-QRBH-14-29902-3-0_pl.cif	30	0.465
XXXIII_structure_52.cif	XXXIII-1-GDay-33_XXXIII_a_0_opt.33_XXXIII_c_0_opt-QRBH-61-903-3-8.cif	30	0.377
XXXIII_structure_53.cif	XXXIII-1-GDay-33_XXXIII_a_0_opt.33_XXXIII_c_0_opt-QRBH-15-133-3-21.cif	30	0.661
XXXIII_structure_54.cif	XXXIII-1-GDay-33_XXXIII_a_8_opt.33_XXXIII_c_0_opt-QRBH-14-25753-3-0.cif	30	0.343
XXXIII_structure_55.cif	XXXIII-1-GDay-33_XXXIII_a_0_opt.33_XXXIII_c_0_opt-QRBH-14-14881-3-0.cif	30	0.420
XXXIII_structure_56.cif	XXXIII-1-GDay-33_XXXIII_a_10_opt.33_XXXIII_c_0_opt-QRBH-14-3171-3-5.cif	30	0.356
XXXIII_structure_57.cif	XXXIII-1-GDay-33_XXXIII_a_2_opt.33_XXXIII_c_0_opt-QRBH-2-29218-3-0.cif	24	0.549
XXXIII_structure_58.cif	XXXIII-1-MNeumann-structure_1266.cif	30	0.505
XXXIII_structure_59.cif	XXXIII-1-GDay-33_XXXIII_a_0_opt.33_XXXIII_c_0_opt-QRBH-15-40-3-17.cif	30	0.428
XXXIII_structure_60.cif	XXXIII-1-SLPrice-XXXIII_dfBb331_Bb331.cif	30	0.496
XXXIII_structure_61.cif	XXXIII-1-GDay-33_XXXIII_a_2_opt.33_XXXIII_c_0_opt-QRBH-2-15519-3-0.cif	30	0.309
XXXIII_structure_62.cif	XXXIII-1-GDay-33_XXXIII_a_0_opt.33_XXXIII_c_0_opt-QRBH-14-81-3-43.cif	26	0.703
XXXIII_structure_63.cif	XXXIII-1-GDay-33_XXXIII_a_0_opt.33_XXXIII_c_0_opt-QRBH-33-256-3-14.cif	30	0.547
XXXIII_structure_64.cif	XXXIII-1-GDay-33_XXXIII_a_0_opt.33_XXXIII_c_0_opt-QRBH-14-29730-3-0.cif	30	0.421
XXXIII_structure_65.cif	XXXIII-1-MNeumann-structure_1281.cif	30	0.590
XXXIII_structure_66.cif	XXXIII-1-SLPrice-XXXIII_dfBb107_Bb107.cif	30	0.459
XXXIII_structure_67.cif	XXXIII-1-GDay-33_XXXIII_a_10_opt.33_XXXIII_c_0_opt-QRBH-14-31944-3-0.cif	30	0.388
XXXIII_structure_68.cif	XXXIII-1-GDay-33_XXXIII_a_2_opt.33_XXXIII_c_0_opt-QRBH-2-14323-3-0_pl.cif	24	0.626
XXXIII_structure_69.cif	XXXIII-1-GDay-33_XXXIII_a_0_opt.33_XXXIII_c_0_opt-QRBH-14-174-3-0.cif	21	0.737
XXXIII_structure_70.cif	XXXIII-1-SLPrice-XXXIII_dfBa53_Ba53.cif	30	0.687

XXXIII_structure_71.cif	XXXIII-1-GDay-33_XXXIII_a_0_opt.33_XXXIII_c_0_opt-QRBH-14-26425-3-0.cif	30	0.366
XXXIII_structure_72.cif	XXXIII-1-MNeumann-structure_1013.cif	30	0.570
XXXIII_structure_73.cif	XXXIII-1-MNeumann-structure_530.cif	30	0.488
XXXIII_structure_74.cif	XXXIII-1-GDay-33_XXXIII_a_0_opt.33_XXXIII_c_0_opt-QRBH-2-1189-3-0.cif	24	0.777
XXXIII_structure_75.cif	XXXIII-1-GDay-33_XXXIII_a_0_opt.33_XXXIII_c_0_opt-QRBH-14-8-3-87.cif	30	0.505
XXXIII_structure_76.cif	XXXIII-1-GDay-33_XXXIII_a_2_opt.33_XXXIII_c_0_opt-QRBH-2-1641-3-0.cif	21	0.739
XXXIII_structure_77.cif	XXXIII-1-MNeumann-structure_1375.cif	30	0.534
XXXIII_structure_78.cif	XXXIII-1-GDay-33_XXXIII_a_2_opt.33_XXXIII_c_0_opt-QRBH-14-32653-3-0.cif	30	0.362
XXXIII_structure_79.cif	XXXIII-1-GDay-33_XXXIII_a_0_opt.33_XXXIII_c_0_opt-QRBH-14-10445-3-0.cif	30	0.591
XXXIII_structure_80.cif	XXXIII-1-MNeumann-structure_1462.cif	30	0.455
XXXIII_structure_81.cif	XXXIII-1-GDay-33_XXXIII_a_10_opt.33_XXXIII_c_0_opt-QRBH-14-2171-3-1.cif	30	0.569
XXXIII_structure_82.cif	XXXIII-1-GDay-33_XXXIII_a_0_opt.33_XXXIII_c_0_opt-QRBH-61-2180-3-4_pl.cif	30	0.350
XXXIII_structure_83.cif	XXXIII-1-GDay-33_XXXIII_a_2_opt.33_XXXIII_c_0_opt-QRBH-2-280-3-0.cif	30	0.596
XXXIII_structure_84.cif	XXXIII-1-GDay-33_XXXIII_a_0_opt.33_XXXIII_c_0_opt-QRBH-14-45-3-59.cif	30	0.366
XXXIII_structure_85.cif	XXXIII-1-GDay-33_XXXIII_a_10_opt.33_XXXIII_c_0_opt-QRBH-61-174-3-0.cif	30	0.522
XXXIII_structure_86.cif	XXXIII-1-GDay-33_XXXIII_a_10_opt.33_XXXIII_c_0_opt-QRBH-4-4984-3-0.cif	30	0.478
XXXIII_structure_87.cif	XXXIII-1-GDay-33_XXXIII_a_0_opt.33_XXXIII_c_0_opt-QRBH-61-1226-3-2.cif	30	0.382
XXXIII_structure_88.cif	XXXIII-1-GDay-33_XXXIII_a_27_opt.33_XXXIII_c_0_opt-QRBH-14-7616-3-1_pl.cif	30	0.431
XXXIII_structure_89.cif	XXXIII-1-GDay-33_XXXIII_a_10_opt.33_XXXIII_c_0_opt-QRBH-15-61-3-36.cif	30	0.446
XXXIII_structure_90.cif	XXXIII-1-MNeumann-structure_738.cif	30	0.470
XXXIII_structure_91.cif	XXXIII-1-GDay-33_XXXIII_a_11_opt.33_XXXIII_c_0_opt-QRBH-14-38498-3-0.cif	30	0.496
XXXIII_structure_92.cif	XXXIII-1-MNeumann-structure_1353.cif	30	0.367
XXXIII_structure_93.cif	XXXIII-1-SLPrice-XXXIII_dfBb229_Bb229.cif	30	0.690
XXXIII_structure_94.cif	XXXIII-1-GDay-33_XXXIII_a_0_opt.33_XXXIII_c_0_opt-QRBH-61-177-3-15.cif	30	0.442
XXXIII_structure_95.cif	XXXIII-1-GDay-33_XXXIII_a_14_opt.33_XXXIII_c_0_opt-QRBH-14-15843-3-0_pl.cif	30	0.475
XXXIII_structure_96.cif	XXXIII-1-GDay-33_XXXIII_a_0_opt.33_XXXIII_c_0_opt-QRBH-2-30486-3-0.cif	30	0.506
XXXIII_structure_97.cif	XXXIII-1-SLPrice-XXXIII_dfBa129_Ba129.cif	25	0.553
XXXIII_structure_98.cif	XXXIII-1-MNeumann-structure_474.cif	30	0.588
XXXIII_structure_99.cif	XXXIII-1-GDay-33_XXXIII_a_14_opt.33_XXXIII_c_0_opt-QRBH-14-41390-3-1_pl.cif	30	0.435
XXXIII_structure_100.cif	XXXIII-1-GDay-33_XXXIII_a_10_opt.33_XXXIII_c_0_opt-QRBH-14-8-3-9.cif	30	0.453
XXXIII_structure_101.cif	XXXIII-1-GDay-33_XXXIII_a_11_opt.33_XXXIII_c_0_opt-QRBH-14-40781-3-0.cif	30	0.545
XXXIII_structure_102.cif	XXXIII-1-SLPrice-XXXIII_dfBb819_Bb819.cif	30	0.546
XXXIII_structure_103.cif	XXXIII-1-GDay-33_XXXIII_a_2_opt.33_XXXIII_c_0_opt-QRBH-14-22036-3-2.cif	30	0.756
XXXIII_structure_104.cif	XXXIII-1-GDay-33_XXXIII_a_0_opt.33_XXXIII_c_0_opt-QRBH-14-34995-3-1.cif	30	0.483
XXXIII_structure_105.cif	XXXIII-1-GDay-33_XXXIII_a_10_opt.33_XXXIII_c_0_opt-QRBH-14-4475-3-0.cif	30	0.439
XXXIII_structure_106.cif	XXXIII-1-GDay-33_XXXIII_a_2_opt.33_XXXIII_c_0_opt-QRBH-61-427-3-4.cif	30	0.429
XXXIII_structure_107.cif	XXXIII-1-GDay-33_XXXIII_a_12_opt.33_XXXIII_c_0_opt-QRBH-14-21621-3-0.cif	30	0.413
XXXIII_structure_108.cif	XXXIII-1-GDay-33_XXXIII_a_2_opt.33_XXXIII_c_0_opt-QRBH-2-17766-3-0_pl.cif	30	0.587

XXXIII_structure_109.cif	XXXIII-1-GDay-33_XXXIII_a_0_opt.33_XXXIII_c_0_opt-QRBH-14-14086-3-0.cif	25	0.509
XXXIII_structure_110.cif	XXXIII-1-GDay-33_XXXIII_a_0_opt.33_XXXIII_c_0_opt-QRBH-19-4568-3-1.cif	30	0.529
XXXIII_structure_111.cif	XXXIII-1-GDay-33_XXXIII_a_0_opt.33_XXXIII_c_0_opt-QRBH-14-11927-3-0.cif	30	0.481
XXXIII_structure_112.cif	XXXIII-1-GDay-33_XXXIII_a_2_opt.33_XXXIII_c_0_opt-QRBH-2-102-3-1.cif	21	0.625
XXXIII_structure_113.cif	XXXIII-1-GDay-33_XXXIII_a_0_opt.33_XXXIII_c_0_opt-QRBH-14-657-3-12.cif	22	0.553
XXXIII_structure_114.cif	XXXIII-1-GDay-33_XXXIII_a_28_opt.33_XXXIII_c_0_opt-QRBH-2-1093-3-0_pl.cif	30	0.519
XXXIII_structure_115.cif	XXXIII-1-XtalPi-data_212_Z1_st_YUW1ABsYUp3Zdb0M.cif	30	0.396
XXXIII_structure_116.cif	XXXIII-1-XtalPi-data_890_Z1_st_YUW1ABsYUp3Zdb1e.cif	30	0.387
XXXIII_structure_117.cif	XXXIII-1-GDay-33_XXXIII_a_10_opt.33_XXXIII_c_0_opt-QRBH-4-971-3-2.cif	30	0.446
XXXIII_structure_118.cif	XXXIII-1-GDay-33_XXXIII_a_2_opt.33_XXXIII_c_0_opt-QRBH-14-25354-3-0.cif	30	0.550
XXXIII_structure_119.cif	XXXIII-1-GDay-33_XXXIII_a_11_opt.33_XXXIII_c_0_opt-QRBH-15-747-3-10.cif	30	0.525
XXXIII_structure_120.cif	XXXIII-1-MNeumann-structure_386.cif	30	0.500
XXXIII_structure_121.cif	XXXIII-1-XtalPi-data_1288_Z1_st_YD2kLi1FJgABE1Ur.cif	30	0.503
XXXIII_structure_122.cif	XXXIII-1-SLPrice-XXXIII_dfBa49_Ba49.cif	30	0.584
XXXIII_structure_123.cif	XXXIII-1-GDay-33_XXXIII_a_0_opt.33_XXXIII_c_0_opt-QRBH-14-1228-3-2.cif	30	0.561
XXXIII_structure_124.cif	XXXIII-1-GDay-33_XXXIII_a_2_opt.33_XXXIII_c_0_opt-QRBH-2-5123-3-0.cif	30	0.575
XXXIII_structure_125.cif	XXXIII-1-GDay-33_XXXIII_a_2_opt.33_XXXIII_c_0_opt-QRBH-2-20592-3-0.cif	26	0.720
XXXIII_structure_126.cif	XXXIII-1-MNeumann-structure_1333.cif	30	0.436
XXXIII_structure_127.cif	XXXIII-1-GDay-33_XXXIII_a_0_opt.33_XXXIII_c_1_opt-QRBH-61-41-3-10.cif	30	0.443
XXXIII_structure_128.cif	XXXIII-1-GDay-33_XXXIII_a_10_opt.33_XXXIII_c_0_opt-QRBH-14-3280-3-0.cif	30	0.384
XXXIII_structure_129.cif	XXXIII-1-GDay-33_XXXIII_a_0_opt.33_XXXIII_c_0_opt-QRBH-61-2180-3-4.cif	30	0.568
XXXIII_structure_130.cif	XXXIII-1-SLPrice-XXXIII_dfBb1691_Bb1691.cif	30	0.464
XXXIII_structure_131.cif	XXXIII-1-GDay-33_XXXIII_a_10_opt.33_XXXIII_c_0_opt-QRBH-14-37255-3-0.cif	30	0.474
XXXIII_structure_132.cif	XXXIII-1-GDay-33_XXXIII_a_0_opt.33_XXXIII_c_0_opt-QRBH-2-101-3-2.cif	16	0.799
XXXIII_structure_133.cif	XXXIII-1-MNeumann-structure_650.cif	30	0.386
XXXIII_structure_134.cif	XXXIII-1-MNeumann-structure_470.cif	27	0.613
XXXIII_structure_135.cif	XXXIII-1-GDay-33_XXXIII_a_0_opt.33_XXXIII_c_0_opt-QRBH-2-19366-3-0.cif	30	0.563
XXXIII_structure_136.cif	XXXIII-1-GDay-33_XXXIII_a_0_opt.33_XXXIII_c_0_opt-QRBH-14-4310-3-0.cif	30	0.553
XXXIII_structure_137.cif	XXXIII-1-XtalPi-data_917_Z1_st_YD2kLi1FJgABE1Y2.cif	30	0.544
XXXIII_structure_138.cif	XXXIII-1-GDay-33_XXXIII_a_2_opt.33_XXXIII_c_0_opt-QRBH-19-1158-3-6.cif	26	0.782
XXXIII_structure_139.cif	XXXIII-1-GDay-33_XXXIII_a_0_opt.33_XXXIII_c_0_opt-QRBH-61-1425-3-1.cif	30	0.517
XXXIII_structure_140.cif	XXXIII-1-SLPrice-XXXIII_dfBa1322_Ba1322.cif	30	0.552
XXXIII_structure_141.cif	XXXIII-1-GDay-33_XXXIII_a_0_opt.33_XXXIII_c_0_opt-QRBH-14-4911-3-5.cif	30	0.488
XXXIII_structure_142.cif	XXXIII-1-GDay-33_XXXIII_a_0_opt.33_XXXIII_c_0_opt-QRBH-14-81-3-12.cif	30	0.422
XXXIII_structure_143.cif	XXXIII-1-GDay-33_XXXIII_a_0_opt.33_XXXIII_c_0_opt-QRBH-14-5991-3-1.cif	30	0.388
XXXIII_structure_144.cif	XXXIII-1-GDay-33_XXXIII_a_10_opt.33_XXXIII_c_0_opt-QRBH-14-51350-3-0.cif	22	0.413
XXXIII_structure_145.cif	XXXIII-1-SLPrice-XXXIII_dfBa715_Ba715.cif	26	0.626
XXXIII_structure_146.cif	XXXIII-1-GDay-33_XXXIII_a_0_opt.33_XXXIII_c_0_opt-QRBH-2-19054-3-0.cif	30	0.555
XXXIII_structure_147.cif	XXXIII-1-MNeumann-structure_335.cif	30	0.498

XXXIII_structure_148.cif	XXXIII-1-GDay-33_XXXIII_a_2_opt.33_XXXIII_c_0_opt-QRBH-14-36933-3-1_pl.cif	30	0.476
XXXIII_structure_149.cif	XXXIII-1-GDay-33_XXXIII_a_10_opt.33_XXXIII_c_0_opt-QRBH-14-5551-3-0.cif	30	0.553
XXXIII_structure_150.cif	XXXIII-1-GDay-33_XXXIII_a_28_opt.33_XXXIII_c_0_opt-QRBH-19-16003-3-1_pl.cif	30	0.244
XXXIII_structure_151.cif	XXXIII-1-SLPrice-XXXIII_dfBa13_Ba13.cif	21	0.727
XXXIII_structure_152.cif	XXXIII-1-GDay-33_XXXIII_a_2_opt.33_XXXIII_c_0_opt-QRBH-2-14944-3-0.cif	30	0.362
XXXIII_structure_153.cif	XXXIII-1-MNeumann-structure_215.cif	30	0.398
XXXIII_structure_154.cif	XXXIII-1-GDay-33_XXXIII_a_28_opt.33_XXXIII_c_0_opt-QRBH-19-7350-3-2_pl.cif	30	0.333
XXXIII_structure_155.cif	XXXIII-1-GDay-33_XXXIII_a_2_opt.33_XXXIII_c_0_opt-QRBH-14-400-3-1.cif	22	0.409
XXXIII_structure_156.cif	XXXIII-1-MNeumann-structure_1358.cif	30	0.383
XXXIII_structure_157.cif	XXXIII-1-SLPrice-XXXIII_dfBb149_Bb149.cif	30	0.614
XXXIII_structure_158.cif	XXXIII-1-GDay-33_XXXIII_a_0_opt.33_XXXIII_c_0_opt-QRBH-14-36774-3-1.cif	30	0.400
XXXIII_structure_159.cif	XXXIII-1-MNeumann-structure_1222.cif	30	0.514
XXXIII_structure_160.cif	XXXIII-1-GDay-33_XXXIII_a_0_opt.33_XXXIII_c_0_opt-QRBH-19-21719-3-0.cif	30	0.429
XXXIII_structure_161.cif	XXXIII-1-GDay-33_XXXIII_a_0_opt.33_XXXIII_c_0_opt-QRBH-14-9177-3-3.cif	30	0.481
XXXIII_structure_162.cif	XXXIII-1-MNeumann-structure_1252.cif	30	0.459
XXXIII_structure_163.cif	XXXIII-1-GDay-33_XXXIII_a_11_opt.33_XXXIII_c_0_opt-QRBH-14-43574-3-0.cif	30	0.503
XXXIII_structure_164.cif	XXXIII-1-GDay-33_XXXIII_a_6_opt.33_XXXIII_c_0_opt-QRBH-14-51434-3-4.cif	30	0.542
XXXIII_structure_165.cif	XXXIII-1-XtalPi-data_916_Z1_st_YD2kLi1FJgABE1bH.cif	21	1.003
XXXIII_structure_166.cif	XXXIII-1-GDay-33_XXXIII_a_4_opt.33_XXXIII_c_0_opt-QRBH-19-47-3-3.cif	25	0.601
XXXIII_structure_167.cif	XXXIII-1-MNeumann-structure_359.cif	30	0.552
XXXIII_structure_168.cif	XXXIII-1-GDay-33_XXXIII_a_10_opt.33_XXXIII_c_0_opt-QRBH-14-1884-3-2.cif	30	0.517
XXXIII_structure_169.cif	XXXIII-1-GDay-33_XXXIII_a_16_opt.33_XXXIII_c_0_opt-QRBH-14-34829-3-0_pl.cif	30	0.365
XXXIII_structure_170.cif	XXXIII-1-MNeumann-structure_1046.cif	30	0.529
XXXIII_structure_171.cif	XXXIII-1-GDay-33_XXXIII_a_0_opt.33_XXXIII_c_0_opt-QRBH-15-113-3-45_pl.cif	30	0.419
XXXIII_structure_172.cif	XXXIII-1-GDay-33_XXXIII_a_0_opt.33_XXXIII_c_0_opt-QRBH-14-27407-3-0.cif	30	0.277
XXXIII_structure_173.cif	XXXIII-1-GDay-33_XXXIII_a_2_opt.33_XXXIII_c_0_opt-QRBH-2-24148-3-0.cif	29	0.505
XXXIII_structure_174.cif	XXXIII-1-MNeumann-structure_2.cif	30	0.537
XXXIII_structure_175.cif	XXXIII-1-XtalPi-data_449_Z1_st_YUW1ABsYUp3Zdb0z.cif	30	0.339
XXXIII_structure_176.cif	XXXIII-1-GDay-33_XXXIII_a_11_opt.33_XXXIII_c_0_opt-QRBH-14-5674-3-0.cif	30	0.594
XXXIII_structure_177.cif	XXXIII-1-MNeumann-structure_1143.cif	30	0.527
XXXIII_structure_178.cif	XXXIII-1-GDay-33_XXXIII_a_4_opt.33_XXXIII_c_0_opt-QRBH-15-374-3-6.cif	30	0.494
XXXIII_structure_179.cif	XXXIII-1-GDay-33_XXXIII_a_0_opt.33_XXXIII_c_0_opt-QRBH-2-11010-3-1_pl.cif	30	0.485
XXXIII_structure_180.cif	XXXIII-1-GDay-33_XXXIII_a_10_opt.33_XXXIII_c_0_opt-QRBH-14-40685-3-0.cif	30	0.365
XXXIII_structure_181.cif	XXXIII-1-XtalPi-data_719_Z1_st_YUW1ABsYUp3Zdb0q.cif	26	0.659
XXXIII_structure_182.cif	XXXIII-1-GDay-33_XXXIII_a_0_opt.33_XXXIII_c_0_opt-QRBH-14-1121-3-22_pl.cif	30	0.442
XXXIII_structure_183.cif	XXXIII-1-GDay-33_XXXIII_a_10_opt.33_XXXIII_c_0_opt-QRBH-61-599-3-0.cif	26	0.617
XXXIII_structure_184.cif	XXXIII-1-MNeumann-structure_759.cif	30	0.580
XXXIII_structure_185.cif	XXXIII-1-GDay-33_XXXIII_a_0_opt.33_XXXIII_c_0_opt-QRBH-14-41163-3-1.cif	22	0.596
XXXIII_structure_186.cif	XXXIII-1-GDay-33_XXXIII_a_2_opt.33_XXXIII_c_0_opt-QRBH-2-2134-3-0.cif	30	0.473
XXXIII_structure_187.cif	XXXIII-1-MNeumann-structure_1313.cif	30	0.334

XXXIII_structure_188.cif	XXXIII-1-GDay-33_XXXIII_a_2_opt.33_XXXIII_c_0_opt-QRBH-2-15892-3-0.cif	30	0.532
XXXIII_structure_189.cif	XXXIII-1-GDay-33_XXXIII_a_0_opt.33_XXXIII_c_0_opt-QRBH-2-2947-3-3.cif	30	0.928
XXXIII_structure_190.cif	XXXIII-1-GDay-33_XXXIII_a_0_opt.33_XXXIII_c_0_opt-QRBH-14-26890-3-0.cif	30	0.544
XXXIII_structure_191.cif	XXXIII-1-GDay-33_XXXIII_a_4_opt.33_XXXIII_c_0_opt-QRBH-14-9-3-84.cif	22	0.432
XXXIII_structure_192.cif	XXXIII-1-XtalPi-data_264_Z1_st_YUW1ABsYUp3Zdb0V.cif	30	0.484
XXXIII_structure_193.cif	XXXIII-1-GDay-33_XXXIII_a_10_opt.33_XXXIII_c_0_opt-QRBH-14-40609-3-0.cif	22	0.441
XXXIII_structure_194.cif	XXXIII-1-GDay-33_XXXIII_a_0_opt.33_XXXIII_c_0_opt-QRBH-61-13-3-117.cif	30	0.422
XXXIII_structure_195.cif	XXXIII-1-GDay-33_XXXIII_a_11_opt.33_XXXIII_c_0_opt-QRBH-14-88-3-42.cif	30	0.612
XXXIII_structure_196.cif	XXXIII-1-MNeumann-structure_1205.cif	30	0.431
XXXIII_structure_197.cif	XXXIII-1-MNeumann-structure_1320.cif	30	0.569
XXXIII_structure_198.cif	XXXIII-1-GDay-33_XXXIII_a_8_opt.33_XXXIII_c_0_opt-QRBH-19-15030-3-0.cif	24	0.608
XXXIII_structure_199.cif	XXXIII-1-GDay-33_XXXIII_a_0_opt.33_XXXIII_c_0_opt-QRBH-15-142-3-38.cif	26	0.478
XXXIII_structure_200.cif	XXXIII-1-GDay-33_XXXIII_a_10_opt.33_XXXIII_c_0_opt-QRBH-4-10111-3-0.cif	22	0.647
XXXIII_structure_201.cif	XXXIII-1-GDay-33_XXXIII_a_10_opt.33_XXXIII_c_0_opt-QRBH-2-12121-3-0.cif	30	0.499
XXXIII_structure_202.cif	XXXIII-1-GDay-33_XXXIII_a_0_opt.33_XXXIII_c_0_opt-QRBH-2-13945-3-0.cif	23	0.811
XXXIII_structure_203.cif	XXXIII-1-SLPrice-XXXIII_dfBb380_Bb380.cif	30	0.425
XXXIII_structure_204.cif	XXXIII-1-SLPrice-XXXIII_dfBa88_Ba88.cif	30	0.480
XXXIII_structure_205.cif	XXXIII-1-GDay-33_XXXIII_a_0_opt.33_XXXIII_c_0_opt-QRBH-2-101-3-2_pl.cif	30	0.387
XXXIII_structure_206.cif	XXXIII-1-GDay-33_XXXIII_a_10_opt.33_XXXIII_c_0_opt-QRBH-33-525-3-0.cif	30	0.278
XXXIII_structure_207.cif	XXXIII-1-MNeumann-structure_1283.cif	30	0.591
XXXIII_structure_208.cif	XXXIII-1-GDay-33_XXXIII_a_2_opt.33_XXXIII_c_0_opt-QRBH-2-17313-3-0.cif	30	0.521
XXXIII_structure_209.cif	XXXIII-1-GDay-33_XXXIII_a_4_opt.33_XXXIII_c_0_opt-QRBH-4-13450-3-0.cif	30	0.394
XXXIII_structure_210.cif	XXXIII-1-XtalPi-data_806_Z1_st_YD2kLi1FJgABE1ar.cif	26	0.400
XXXIII_structure_211.cif	XXXIII-1-GDay-33_XXXIII_a_4_opt.33_XXXIII_c_0_opt-QRBH-14-52712-3-0.cif	30	0.483
XXXIII_structure_212.cif	XXXIII-1-XtalPi-data_319_Z1_st_YUW1ABsYUp3Zdb1h.cif	30	0.681
XXXIII_structure_213.cif	XXXIII-1-MNeumann-structure_1227.cif	30	0.577
XXXIII_structure_214.cif	XXXIII-1-MNeumann-structure_1298.cif	30	0.416
XXXIII_structure_215.cif	XXXIII-1-SLPrice-XXXIII_dfBb372_Bb372.cif	30	0.416
XXXIII_structure_216.cif	XXXIII-1-GDay-33_XXXIII_a_0_opt.33_XXXIII_c_0_opt-QRBH-14-22022-3-1.cif	30	0.368
XXXIII_structure_217.cif	XXXIII-1-GDay-33_XXXIII_a_10_opt.33_XXXIII_c_0_opt-QRBH-2-32440-3-0.cif	30	0.389
XXXIII_structure_218.cif	XXXIII-1-XtalPi-data_38_Z1_st_YUW1ABsYUp3Zdb03.cif	30	0.441
XXXIII_structure_219.cif	XXXIII-1-GDay-33_XXXIII_a_0_opt.33_XXXIII_c_0_opt-QRBH-14-11182-3-8.cif	30	0.499
XXXIII_structure_220.cif	XXXIII-1-MNeumann-structure_717.cif	30	0.521
XXXIII_structure_221.cif	XXXIII-1-XtalPi-data_906_Z1_st_YTW2uwwufAABSknS.cif	30	0.378
XXXIII_structure_222.cif	XXXIII-1-SLPrice-XXXIII_dfBb504_Bb504.cif	30	0.551
XXXIII_structure_223.cif	XXXIII-1-GDay-33_XXXIII_a_10_opt.33_XXXIII_c_0_opt-QRBH-15-375-3-10.cif	30	0.374
XXXIII_structure_224.cif	XXXIII-1-GDay-33_XXXIII_a_0_opt.33_XXXIII_c_0_opt-QRBH-14-21029-3-0.cif	30	0.654
XXXIII_structure_225.cif	XXXIII-1-XtalPi-data_1213_Z1_st_YD2kLi1FJgABE1F3.cif	30	0.282
XXXIII_structure_226.cif	XXXIII-1-GDay-33_XXXIII_a_0_opt.33_XXXIII_c_0_opt-QRBH-14-50878-3-2.cif	30	0.615
XXXIII_structure_227.cif	XXXIII-1-XtalPi-data_51_Z1_st_YUW1ABsYUp3Zdb01.cif	30	0.506
XXXIII_structure_228.cif	XXXIII-1-GDay-33_XXXIII_a_0_opt.33_XXXIII_c_0_opt-QRBH-2-6588-3-0.cif	30	0.382

XXXIII_structure_229.cif	XXXIII-1-GDay-33_XXXIII_a_2_opt.33_XXXIII_c_0_opt-QRBH-14-35722-3-1.cif	26	0.562
XXXIII_structure_230.cif	XXXIII-1-GDay-33_XXXIII_a_0_opt.33_XXXIII_c_0_opt-QRBH-14-7435-3-0.cif	30	0.629
XXXIII_structure_231.cif	XXXIII-1-GDay-33_XXXIII_a_0_opt.33_XXXIII_c_0_opt-QRBH-4-10176-3-0.cif	30	0.342
XXXIII_structure_232.cif	XXXIII-1-MNeumann-structure_725.cif	27	0.554
XXXIII_structure_233.cif	*XXXIII-1-SOHG-XXXIII-0011.cif	30	0.426
XXXIII_structure_234.cif	XXXIII-1-GDay-33_XXXIII_a_11_opt.33_XXXIII_c_0_opt-QRBH-14-8109-3-0.cif	30	0.650
XXXIII_structure_235.cif	XXXIII-1-GDay-33_XXXIII_a_4_opt.33_XXXIII_c_0_opt-QRBH-14-5573-3-0.cif	30	0.374
XXXIII_structure_236.cif	XXXIII-1-MNeumann-structure_214.cif	30	0.374
XXXIII_structure_237.cif	XXXIII-1-GDay-33_XXXIII_a_15_opt.33_XXXIII_c_0_opt-QRBH-2-6165-3-0_pl.cif	30	0.403
XXXIII_structure_238.cif	XXXIII-1-SLPrice-XXXIII_dfBb728_Bb728.cif	30	0.321
XXXIII_structure_239.cif	XXXIII-1-GDay-33_XXXIII_a_10_opt.33_XXXIII_c_0_opt-QRBH-15-289-3-25.cif	30	0.669
XXXIII_structure_240.cif	XXXIII-1-GDay-33_XXXIII_a_6_opt.33_XXXIII_c_0_opt-QRBH-4-5398-3-0.cif	30	0.423
XXXIII_structure_241.cif	XXXIII-1-GDay-33_XXXIII_a_0_opt.33_XXXIII_c_0_opt-QRBH-14-48371-3-0.cif	30	0.371
XXXIII_structure_242.cif	XXXIII-1-XtalPi-data_107_Z1_st_YUW1ABsYUp3Zdb1B.cif	30	0.333
XXXIII_structure_243.cif	XXXIII-1-GDay-33_XXXIII_a_0_opt.33_XXXIII_c_0_opt-QRBH-14-334-3-0.cif	30	0.479
XXXIII_structure_244.cif	XXXIII-1-MNeumann-structure_1016.cif	30	0.390
XXXIII_structure_245.cif	XXXIII-1-GDay-33_XXXIII_a_4_opt.33_XXXIII_c_0_opt-QRBH-14-2959-3-1.cif	30	0.615
XXXIII_structure_246.cif	XXXIII-1-GDay-33_XXXIII_a_10_opt.33_XXXIII_c_0_opt-QRBH-14-7655-3-2.cif	30	0.495
XXXIII_structure_247.cif	XXXIII-1-GDay-33_XXXIII_a_4_opt.33_XXXIII_c_0_opt-QRBH-29-867-3-0.cif	30	0.350
XXXIII_structure_248.cif	XXXIII-1-GDay-33_XXXIII_a_0_opt.33_XXXIII_c_0_opt-QRBH-14-650-3-0.cif	30	0.613
XXXIII_structure_249.cif	XXXIII-1-GDay-33_XXXIII_a_0_opt.33_XXXIII_c_0_opt-QRBH-19-19333-3-0.cif	30	0.564
XXXIII_structure_250.cif	XXXIII-1-MNeumann-structure_1165.cif	23	0.644
XXXIII_structure_251.cif	XXXIII-1-GDay-33_XXXIII_a_4_opt.33_XXXIII_c_0_opt-QRBH-14-8312-3-1.cif	30	0.319
XXXIII_structure_252.cif	XXXIII-1-MNeumann-structure_17.cif	30	0.371
XXXIII_structure_253.cif	XXXIII-1-GDay-33_XXXIII_a_0_opt.33_XXXIII_c_0_opt-QRBH-14-15607-3-3.cif	30	0.550
XXXIII_structure_254.cif	XXXIII-1-GDay-33_XXXIII_a_0_opt.33_XXXIII_c_0_opt-QRBH-15-109-3-70.cif	30	0.410
XXXIII_structure_255.cif	XXXIII-1-GDay-33_XXXIII_a_0_opt.33_XXXIII_c_0_opt-QRBH-14-39421-3-0.cif	30	0.530
XXXIII_structure_256.cif	XXXIII-1-GDay-33_XXXIII_a_2_opt.33_XXXIII_c_0_opt-QRBH-2-18234-3-0.cif	27	0.720
XXXIII_structure_257.cif	XXXIII-1-GDay-33_XXXIII_a_0_opt.33_XXXIII_c_0_opt-QRBH-14-23943-3-0.cif	30	0.603
XXXIII_structure_258.cif	XXXIII-1-GDay-33_XXXIII_a_4_opt.33_XXXIII_c_0_opt-QRBH-15-50-3-31.cif	27	0.644
XXXIII_structure_259.cif	XXXIII-1-MNeumann-structure_401.cif	30	0.497
XXXIII_structure_260.cif	XXXIII-1-GDay-33_XXXIII_a_0_opt.33_XXXIII_c_0_opt-QRBH-14-31107-3-0.cif	26	0.734
XXXIII_structure_261.cif	XXXIII-1-MNeumann-structure_112.cif	25	0.618
XXXIII_structure_262.cif	XXXIII-1-GDay-33_XXXIII_a_0_opt.33_XXXIII_c_0_opt-QRBH-15-45-3-33.cif	30	0.348
XXXIII_structure_263.cif	XXXIII-1-GDay-33_XXXIII_a_27_opt.33_XXXIII_c_0_opt-QRBH-14-26236-3-0_pl.cif	30	0.366
XXXIII_structure_264.cif	XXXIII-1-GDay-33_XXXIII_a_0_opt.33_XXXIII_c_0_opt-QRBH-14-6372-3-1_pl.cif	30	0.497
XXXIII_structure_265.cif	XXXIII-1-GDay-33_XXXIII_a_2_opt.33_XXXIII_c_0_opt-QRBH-14-47783-3-0.cif	30	0.704
XXXIII_structure_266.cif	XXXIII-1-GDay-33_XXXIII_a_10_opt.33_XXXIII_c_0_opt-QRBH-2-1698-3-4.cif	30	0.397

XXXIII_structure_267.cif	XXXIII-1-GDay-33_XXXIII_a_0_opt.33_XXXIII_c_0_opt-QRBH-14-880-3-1.cif	30	0.377
XXXIII_structure_268.cif	XXXIII-1-GDay-33_XXXIII_a_0_opt.33_XXXIII_c_0_opt-QRBH-14-26268-3-0.cif	30	0.489
XXXIII_structure_269.cif	XXXIII-1-GDay-33_XXXIII_a_0_opt.33_XXXIII_c_0_opt-QRBH-14-3808-3-5.cif	30	0.453
XXXIII_structure_270.cif	XXXIII-1-MNeumann-structure_1269.cif	30	0.320
XXXIII_structure_271.cif	XXXIII-1-GDay-33_XXXIII_a_2_opt.33_XXXIII_c_0_opt-QRBH-14-44996-3-0.cif	20	1.166
XXXIII_structure_272.cif	XXXIII-1-GDay-33_XXXIII_a_14_opt.33_XXXIII_c_0_opt-QRBH-14-26740-3-0_pl.cif	30	0.478
XXXIII_structure_273.cif	XXXIII-1-GDay-33_XXXIII_a_0_opt.33_XXXIII_c_0_opt-QRBH-14-11909-3-0.cif	14	0.837
XXXIII_structure_274.cif	XXXIII-1-SLPrice-XXXIII_dfBb1529_Bb1529.cif	30	0.384
XXXIII_structure_275.cif	XXXIII-1-GDay-33_XXXIII_a_8_opt.33_XXXIII_c_0_opt-QRBH-4-19963-3-0.cif	30	0.409
XXXIII_structure_276.cif	XXXIII-1-GDay-33_XXXIII_a_11_opt.33_XXXIII_c_0_opt-QRBH-14-29595-3-0.cif	30	0.388
XXXIII_structure_277.cif	XXXIII-1-GDay-33_XXXIII_a_6_opt.33_XXXIII_c_0_opt-QRBH-14-40341-3-0.cif	30	0.433
XXXIII_structure_278.cif	XXXIII-1-MNeumann-structure_593.cif	30	0.449
XXXIII_structure_279.cif	XXXIII-1-GDay-33_XXXIII_a_10_opt.33_XXXIII_c_0_opt-QRBH-15-178-3-17.cif	23	0.697
XXXIII_structure_280.cif	XXXIII-1-GDay-33_XXXIII_a_2_opt.33_XXXIII_c_0_opt-QRBH-14-4216-3-0.cif	30	0.577
XXXIII_structure_281.cif	XXXIII-1-SLPrice-XXXIII_dfBb1088_Bb1088.cif	18	0.419
XXXIII_structure_282.cif	XXXIII-1-GDay-33_XXXIII_a_0_opt.33_XXXIII_c_0_opt-QRBH-14-28743-3-0.cif	30	0.380
XXXIII_structure_283.cif	XXXIII-1-SLPrice-XXXIII_dfBa1870_Ba1870.cif	30	0.412
XXXIII_structure_284.cif	XXXIII-1-GDay-33_XXXIII_a_2_opt.33_XXXIII_c_0_opt-QRBH-2-2740-3-0.cif	25	0.360
XXXIII_structure_285.cif	XXXIII-1-GDay-33_XXXIII_a_0_opt.33_XXXIII_c_0_opt-QRBH-4-11138-3-0_pl.cif	30	0.555
XXXIII_structure_286.cif	XXXIII-1-GDay-33_XXXIII_a_10_opt.33_XXXIII_c_0_opt-QRBH-2-10052-3-0.cif	21	0.565
XXXIII_structure_287.cif	XXXIII-1-GDay-33_XXXIII_a_8_opt.33_XXXIII_c_0_opt-QRBH-14-52767-3-0_pl.cif	30	0.461
XXXIII_structure_288.cif	XXXIII-1-XtalPi-data_1496_Z1_st_YD2kLi1FJgABE1KX.cif	30	0.393
XXXIII_structure_289.cif	XXXIII-1-GDay-33_XXXIII_a_8_opt.33_XXXIII_c_0_opt-QRBH-14-27318-3-1.cif	30	0.331
XXXIII_structure_290.cif	XXXIII-1-XtalPi-data_19_Z1_st_YD2kLi1FJgABE1GQ.cif	30	0.584
XXXIII_structure_291.cif	XXXIII-1-XtalPi-data_278_Z1_st_YD2kLi1FJgABE1GU.cif	30	0.510
XXXIII_structure_292.cif	XXXIII-1-MNeumann-structure_813.cif	30	0.442
XXXIII_structure_293.cif	XXXIII-1-SLPrice-XXXIII_dfAb340_Ab340.cif	30	0.804
XXXIII_structure_294.cif	XXXIII-1-GDay-33_XXXIII_a_10_opt.33_XXXIII_c_0_opt-QRBH-14-39901-3-0.cif	30	0.433
XXXIII_structure_295.cif	XXXIII-1-SLPrice-XXXIII_dfBb345_Bb345.cif	30	0.392
XXXIII_structure_296.cif	XXXIII-1-GDay-33_XXXIII_a_11_opt.33_XXXIII_c_0_opt-QRBH-14-12755-3-0.cif	26	0.561
XXXIII_structure_297.cif	XXXIII-1-SLPrice-XXXIII_dfBb170_Bb170.cif	30	0.393
XXXIII_structure_298.cif	XXXIII-1-MNeumann-structure_1444.cif	30	0.471
XXXIII_structure_299.cif	XXXIII-1-MNeumann-structure_1148.cif	30	0.380
XXXIII_structure_300.cif	XXXIII-1-GDay-33_XXXIII_a_8_opt.33_XXXIII_c_0_opt-QRBH-61-38-3-45.cif	30	0.521
XXXIII_structure_301.cif	XXXIII-1-GDay-33_XXXIII_a_0_opt.33_XXXIII_c_0_opt-QRBH-14-27617-3-0.cif	27	0.568
XXXIII_structure_302.cif	XXXIII-1-SLPrice-XXXIII_dfBa64_Ba64.cif	30	0.592
XXXIII_structure_303.cif	XXXIII-1-XtalPi-data_1031_Z1_st_YD2kLi1FJgABE1EI.cif	22	0.551
XXXIII_structure_304.cif	XXXIII-1-MNeumann-structure_463.cif	30	0.513
XXXIII_structure_305.cif	XXXIII-1-MNeumann-structure_730.cif	30	0.684
XXXIII_structure_306.cif	XXXIII-1-XtalPi-data_816_Z1_st_YUW1ABsYUp3Zdb0D.cif	30	0.392
XXXIII_structure_307.cif	XXXIII-1-GDay-33_XXXIII_a_10_opt.33_XXXIII_c_0_opt-QRBH-14-655-3-3.cif	23	0.712
XXXIII_structure_308.cif	XXXIII-1-GDay-33_XXXIII_a_0_opt.33_XXXIII_c_0_opt-QRBH-14-998-3-1.cif	30	0.364
XXXIII_structure_309.cif	XXXIII-1-MNeumann-structure_1318.cif	30	0.429

XXXIII_structure_310.cif	XXXIII-1-GDay-33_XXXIII_a_0_opt.33_XXXIII_c_1_opt-QRBH-14-8042-3-0.cif	30	0.566
XXXIII_structure_311.cif	XXXIII-1-XtalPi-data_52_Z1_st_YD2kLi1FJgABE1Cx.cif	30	0.378
XXXIII_structure_312.cif	XXXIII-1-GDay-33_XXXIII_a_0_opt.33_XXXIII_c_0_opt-QRBH-19-20719-3-0.cif	30	0.519
XXXIII_structure_313.cif	XXXIII-1-GDay-33_XXXIII_a_6_opt.33_XXXIII_c_0_opt-QRBH-61-663-3-1.cif	30	0.318
XXXIII_structure_314.cif	XXXIII-1-XtalPi-data_216_Z1_st_YD2kLi1FJgABE1bI.cif	30	0.464
XXXIII_structure_315.cif	XXXIII-1-GDay-33_XXXIII_a_2_opt.33_XXXIII_c_0_opt-QRBH-19-103-3-5.cif	30	0.436
XXXIII_structure_316.cif	XXXIII-1-GDay-33_XXXIII_a_10_opt.33_XXXIII_c_0_opt-QRBH-2-22812-3-0.cif	25	0.658
XXXIII_structure_317.cif	XXXIII-1-SLPrice-XXXIII_dfBb2060_Bb2060.cif	30	0.586
XXXIII_structure_318.cif	XXXIII-1-GDay-33_XXXIII_a_2_opt.33_XXXIII_c_0_opt-QRBH-14-44722-3-0.cif	30	0.585
XXXIII_structure_319.cif	XXXIII-1-GDay-33_XXXIII_a_14_opt.33_XXXIII_c_0_opt-QRBH-14-37208-3-0_pl.cif	30	0.485
XXXIII_structure_320.cif	XXXIII-1-GDay-33_XXXIII_a_6_opt.33_XXXIII_c_0_opt-QRBH-14-29801-3-0.cif	30	0.629
XXXIII_structure_321.cif	XXXIII-1-GDay-33_XXXIII_a_0_opt.33_XXXIII_c_0_opt-QRBH-2-5552-3-0_pl.cif	26	0.664
XXXIII_structure_322.cif	XXXIII-1-GDay-33_XXXIII_a_0_opt.33_XXXIII_c_0_opt-QRBH-61-56-3-49_pl.cif	30	0.502
XXXIII_structure_323.cif	XXXIII-1-GDay-33_XXXIII_a_0_opt.33_XXXIII_c_0_opt-QRBH-14-45731-3-0_pl.cif	30	0.492
XXXIII_structure_324.cif	XXXIII-1-GDay-33_XXXIII_a_10_opt.33_XXXIII_c_0_opt-QRBH-29-877-3-3.cif	30	0.559
XXXIII_structure_325.cif	XXXIII-1-GDay-33_XXXIII_a_10_opt.33_XXXIII_c_0_opt-QRBH-2-33489-3-0.cif	30	0.644
XXXIII_structure_326.cif	XXXIII-1-SLPrice-XXXIII_dfBa246_Ba246.cif	30	0.419
XXXIII_structure_327.cif	XXXIII-1-MNeumann-structure_1247.cif	30	0.431
XXXIII_structure_328.cif	XXXIII-1-SLPrice-XXXIII_dfBa233_Ba233.cif	30	0.405
XXXIII_structure_329.cif	XXXIII-1-SLPrice-XXXIII_dfBa17_Ba17.cif	30	0.466
XXXIII_structure_330.cif	XXXIII-1-GDay-33_XXXIII_a_0_opt.33_XXXIII_c_0_opt-QRBH-61-2118-3-0.cif	30	0.417
XXXIII_structure_331.cif	XXXIII-1-GDay-33_XXXIII_a_0_opt.33_XXXIII_c_0_opt-QRBH-14-23969-3-3.cif	23	0.742
XXXIII_structure_332.cif	XXXIII-1-GDay-33_XXXIII_a_0_opt.33_XXXIII_c_0_opt-QRBH-15-13-3-31.cif	30	0.369
XXXIII_structure_333.cif	XXXIII-1-XtalPi-data_1096_Z1_st_YD2kLi1FJgABE1QU.cif	18	0.784
XXXIII_structure_334.cif	XXXIII-1-GDay-33_XXXIII_a_11_opt.33_XXXIII_c_0_opt-QRBH-14-483-3-13.cif	30	0.490
XXXIII_structure_335.cif	XXXIII-1-GDay-33_XXXIII_a_11_opt.33_XXXIII_c_0_opt-QRBH-14-35364-3-1.cif	30	0.592
XXXIII_structure_336.cif	XXXIII-1-GDay-33_XXXIII_a_4_opt.33_XXXIII_c_0_opt-QRBH-14-60-3-16_pl.cif	27	0.537
XXXIII_structure_337.cif	XXXIII-1-GDay-33_XXXIII_a_0_opt.33_XXXIII_c_0_opt-QRBH-14-28821-3-4.cif	30	0.541
XXXIII_structure_338.cif	XXXIII-1-MNeumann-structure_1383.cif	23	0.449
XXXIII_structure_339.cif	XXXIII-1-XtalPi-data_7_Z1_st_YUW1ABsYUp3Zdb0_.cif	30	0.301
XXXIII_structure_340.cif	XXXIII-1-GDay-33_XXXIII_a_0_opt.33_XXXIII_c_0_opt-QRBH-2-28079-3-0.cif	30	0.441
XXXIII_structure_341.cif	XXXIII-1-GDay-33_XXXIII_a_12_opt.33_XXXIII_c_0_opt-QRBH-14-32061-3-0_pl.cif	30	0.449
XXXIII_structure_342.cif	XXXIII-1-GDay-33_XXXIII_a_4_opt.33_XXXIII_c_0_opt-QRBH-29-55-3-19.cif	30	0.332
XXXIII_structure_343.cif	XXXIII-1-GDay-33_XXXIII_a_15_opt.33_XXXIII_c_0_opt-QRBH-33-756-3-4_pl.cif	30	0.613
XXXIII_structure_344.cif	XXXIII-1-MNeumann-structure_644.cif	30	0.436
XXXIII_structure_345.cif	XXXIII-1-GDay-33_XXXIII_a_10_opt.33_XXXIII_c_0_opt-QRBH-15-780-3-10.cif	30	0.460
XXXIII_structure_346.cif	XXXIII-1-SLPrice-XXXIII_dfAb916_Ab916.cif	30	0.447
XXXIII_structure_347.cif	XXXIII-1-XtalPi-data_456_Z1_st_YUW1ABsYUp3Zdb1K.cif	30	0.383
XXXIII_structure_348.cif	XXXIII-1-GDay-33_XXXIII_a_0_opt.33_XXXIII_c_0_opt-QRBH-14-37311-3-2.cif	30	0.713

XXXIII_structure_349.cif	XXXIII-1-GDay-33_XXXIII_a_12_opt.33_XXXIII_c_0_opt-QRBH-14-13453-3-2.cif	30	0.533
XXXIII_structure_350.cif	XXXIII-1-GDay-33_XXXIII_a_0_opt.33_XXXIII_c_0_opt-QRBH-14-25200-3-1.cif	26	0.649
XXXIII_structure_351.cif	XXXIII-1-GDay-33_XXXIII_a_10_opt.33_XXXIII_c_0_opt-QRBH-29-1590-3-4.cif	30	0.463
XXXIII_structure_352.cif	XXXIII-1-MNeumann-structure_1314.cif	30	0.232
XXXIII_structure_353.cif	XXXIII-1-GDay-33_XXXIII_a_11_opt.33_XXXIII_c_0_opt-QRBH-14-898-3-14.cif	27	0.527
XXXIII_structure_354.cif	XXXIII-1-XtalPi-data_205_Z1_st_YD2kLi1FJgABE1Df.cif	30	0.502
XXXIII_structure_355.cif	XXXIII-1-SLPrice-XXXIII_dfBb118_Bb118.cif	30	0.436
XXXIII_structure_356.cif	XXXIII-1-GDay-33_XXXIII_a_0_opt.33_XXXIII_c_0_opt-QRBH-14-7435-3-0_pl.cif	30	0.491
XXXIII_structure_357.cif	XXXIII-1-GDay-33_XXXIII_a_10_opt.33_XXXIII_c_0_opt-QRBH-14-39-3-28.cif	30	0.582
XXXIII_structure_358.cif	XXXIII-1-XtalPi-data_150_Z1_st_YUW1ABsYUp3Zdb1T.cif	30	0.468
XXXIII_structure_359.cif	XXXIII-1-GDay-33_XXXIII_a_11_opt.33_XXXIII_c_0_opt-QRBH-61-1602-3-3.cif	30	0.659
XXXIII_structure_360.cif	XXXIII-1-GDay-33_XXXIII_a_6_opt.33_XXXIII_c_0_opt-QRBH-14-6998-3-1_pl.cif	30	0.431
XXXIII_structure_361.cif	XXXIII-1-GDay-33_XXXIII_a_0_opt.33_XXXIII_c_0_opt-QRBH-14-9891-3-1.cif	30	0.730
XXXIII_structure_362.cif	XXXIII-1-GDay-33_XXXIII_a_0_opt.33_XXXIII_c_0_opt-QRBH-14-314-3-0.cif	26	0.795
XXXIII_structure_363.cif	XXXIII-1-MNeumann-structure_585.cif	29	0.560
XXXIII_structure_364.cif	XXXIII-1-GDay-33_XXXIII_a_0_opt.33_XXXIII_c_0_opt-QRBH-19-13802-3-0.cif	30	0.611
XXXIII_structure_365.cif	XXXIII-1-SLPrice-XXXIII_dfBb349_Bb349.cif	30	0.447
XXXIII_structure_366.cif	XXXIII-1-GDay-33_XXXIII_a_27_opt.33_XXXIII_c_0_opt-QRBH-14-21909-3-0_pl.cif	30	0.519
XXXIII_structure_367.cif	XXXIII-1-GDay-33_XXXIII_a_0_opt.33_XXXIII_c_0_opt-QRBH-61-840-3-12.cif	30	0.530
XXXIII_structure_368.cif	XXXIII-1-SLPrice-XXXIII_dfBb505_Bb505.cif	30	0.474
XXXIII_structure_369.cif	XXXIII-1-GDay-33_XXXIII_a_2_opt.33_XXXIII_c_0_opt-QRBH-2-26667-3-0.cif	18	0.882
XXXIII_structure_370.cif	XXXIII-1-XtalPi-data_336_Z1_st_YD2kLi1FJgABE1Fz.cif	30	0.423
XXXIII_structure_371.cif	XXXIII-1-SLPrice-XXXIII_dfBa2111_Ba2111.cif	30	0.263
XXXIII_structure_372.cif	XXXIII-1-MNeumann-structure_430.cif	30	0.366
XXXIII_structure_373.cif	XXXIII-1-GDay-33_XXXIII_a_2_opt.33_XXXIII_c_0_opt-QRBH-19-7477-3-0_pl.cif	30	0.525
XXXIII_structure_374.cif	XXXIII-1-GDay-33_XXXIII_a_0_opt.33_XXXIII_c_0_opt-QRBH-2-20542-3-0_pl.cif	30	0.343
XXXIII_structure_375.cif	XXXIII-1-XtalPi-data_574_Z1_st_YD2kLi1FJgABE1Fn.cif	30	0.459
XXXIII_structure_376.cif	XXXIII-1-GDay-33_XXXIII_a_10_opt.33_XXXIII_c_0_opt-QRBH-2-11716-3-0.cif	30	0.354
XXXIII_structure_377.cif	XXXIII-1-XtalPi-data_214_Z1_st_YUW1ABsYUp3Zdb0m.cif	30	0.400
XXXIII_structure_378.cif	XXXIII-1-MNeumann-structure_427.cif	30	0.584
XXXIII_structure_379.cif	XXXIII-1-MNeumann-structure_295.cif	22	0.531
XXXIII_structure_380.cif	XXXIII-1-GDay-33_XXXIII_a_6_opt.33_XXXIII_c_0_opt-QRBH-14-46343-3-0.cif	30	0.447
XXXIII_structure_381.cif	XXXIII-1-GDay-33_XXXIII_a_11_opt.33_XXXIII_c_0_opt-QRBH-19-3603-3-2.cif	30	0.426
XXXIII_structure_382.cif	XXXIII-1-GDay-33_XXXIII_a_27_opt.33_XXXIII_c_0_opt-QRBH-61-93-3-14_pl.cif	30	0.487
XXXIII_structure_383.cif	XXXIII-1-GDay-33_XXXIII_a_0_opt.33_XXXIII_c_0_opt-QRBH-2-14603-3-0.cif	21	0.897
XXXIII_structure_384.cif	XXXIII-1-MNeumann-structure_832.cif	30	0.390
XXXIII_structure_385.cif	XXXIII-1-GDay-33_XXXIII_a_0_opt.33_XXXIII_c_0_opt-QRBH-14-10-3-185.cif	21	0.477
XXXIII_structure_386.cif	XXXIII-1-GDay-33_XXXIII_a_2_opt.33_XXXIII_c_0_opt-QRBH-14-44680-3-1.cif	30	0.484
XXXIII_structure_387.cif	XXXIII-1-GDay-33_XXXIII_a_2_opt.33_XXXIII_c_0_opt-QRBH-2-14323-3-0.cif	30	0.473
XXXIII_structure_388.cif	XXXIII-1-SLPrice-XXXIII_dfBb121_Bb121.cif	25	0.596
XXXIII_structure_389.cif	XXXIII-1-MNeumann-structure_1295.cif	30	0.446

XXXIII_structure_390.cif	XXXIII-1-GDay-33_XXXIII_a_8_opt.33_XXXIII_c_0_opt-QRBH-14-5231-3-0.cif	30	0.453
XXXIII_structure_391.cif	XXXIII-1-GDay-33_XXXIII_a_11_opt.33_XXXIII_c_0_opt-QRBH-14-628-3-23.cif	26	0.676
XXXIII_structure_392.cif	XXXIII-1-GDay-33_XXXIII_a_2_opt.33_XXXIII_c_0_opt-QRBH-2-2561-3-2_pl.cif	30	0.488
XXXIII_structure_393.cif	XXXIII-1-GDay-33_XXXIII_a_10_opt.33_XXXIII_c_0_opt-QRBH-15-666-3-15.cif	30	0.388
XXXIII_structure_394.cif	XXXIII-1-GDay-33_XXXIII_a_10_opt.33_XXXIII_c_0_opt-QRBH-14-49739-3-2.cif	30	0.357
XXXIII_structure_395.cif	XXXIII-1-GDay-33_XXXIII_a_2_opt.33_XXXIII_c_0_opt-QRBH-14-31311-3-2.cif	30	0.599
XXXIII_structure_396.cif	XXXIII-1-GDay-33_XXXIII_a_2_opt.33_XXXIII_c_0_opt-QRBH-14-21415-3-2.cif	26	0.584
XXXIII_structure_397.cif	XXXIII-1-XtalPi-data_1074_Z1_st_YD2kLi1FJgABE1U3.cif	30	0.301
XXXIII_structure_398.cif	XXXIII-1-GDay-33_XXXIII_a_10_opt.33_XXXIII_c_0_opt-QRBH-14-27794-3-1.cif	30	0.429
XXXIII_structure_399.cif	XXXIII-1-GDay-33_XXXIII_a_0_opt.33_XXXIII_c_0_opt-QRBH-15-113-3-52.cif	30	0.439
XXXIII_structure_400.cif	XXXIII-1-GDay-33_XXXIII_a_10_opt.33_XXXIII_c_0_opt-QRBH-14-380-3-17.cif	30	0.441
XXXIII_structure_401.cif	XXXIII-1-GDay-33_XXXIII_a_0_opt.33_XXXIII_c_0_opt-QRBH-14-33488-3-0.cif	30	0.701
XXXIII_structure_402.cif	XXXIII-1-GDay-33_XXXIII_a_8_opt.33_XXXIII_c_0_opt-QRBH-61-1182-3-10.cif	30	0.580
XXXIII_structure_403.cif	XXXIII-1-GDay-33_XXXIII_a_0_opt.33_XXXIII_c_0_opt-QRBH-2-17570-3-1.cif	27	0.598
XXXIII_structure_404.cif	XXXIII-1-GDay-33_XXXIII_a_0_opt.33_XXXIII_c_0_opt-QRBH-14-6086-3-4_pl.cif	30	0.432
XXXIII_structure_405.cif	XXXIII-1-GDay-33_XXXIII_a_8_opt.33_XXXIII_c_0_opt-QRBH-14-112-3-18.cif	30	0.566
XXXIII_structure_406.cif	XXXIII-1-GDay-33_XXXIII_a_0_opt.33_XXXIII_c_0_opt-QRBH-14-19675-3-0.cif	23	0.631
XXXIII_structure_407.cif	XXXIII-1-GDay-33_XXXIII_a_10_opt.33_XXXIII_c_0_opt-QRBH-14-22071-3-1.cif	25	0.549
XXXIII_structure_408.cif	XXXIII-1-GDay-33_XXXIII_a_0_opt.33_XXXIII_c_0_opt-QRBH-19-18700-3-0.cif	30	0.637
XXXIII_structure_409.cif	XXXIII-1-GDay-33_XXXIII_a_0_opt.33_XXXIII_c_0_opt-QRBH-14-3395-3-0_pl.cif	30	0.448
XXXIII_structure_410.cif	XXXIII-1-GDay-33_XXXIII_a_0_opt.33_XXXIII_c_0_opt-QRBH-2-13862-3-0.cif	25	0.536
XXXIII_structure_411.cif	XXXIII-1-GDay-33_XXXIII_a_0_opt.33_XXXIII_c_0_opt-QRBH-14-21113-3-1.cif	22	0.520
XXXIII_structure_412.cif	XXXIII-1-GDay-33_XXXIII_a_4_opt.33_XXXIII_c_0_opt-QRBH-2-29235-3-0.cif	30	0.760
XXXIII_structure_413.cif	XXXIII-1-GDay-33_XXXIII_a_8_opt.33_XXXIII_c_0_opt-QRBH-61-200-3-14_pl.cif	30	0.356
XXXIII_structure_414.cif	XXXIII-1-GDay-33_XXXIII_a_0_opt.33_XXXIII_c_0_opt-QRBH-33-34-3-1.cif	30	0.585
XXXIII_structure_415.cif	XXXIII-1-MNeumann-structure_241.cif	30	0.292
XXXIII_structure_416.cif	XXXIII-1-GDay-33_XXXIII_a_0_opt.33_XXXIII_c_0_opt-QRBH-4-22249-3-0.cif	30	0.476
XXXIII_structure_417.cif	XXXIII-1-GDay-33_XXXIII_a_0_opt.33_XXXIII_c_0_opt-QRBH-14-16852-3-0.cif	30	0.568
XXXIII_structure_418.cif	XXXIII-1-GDay-33_XXXIII_a_6_opt.33_XXXIII_c_0_opt-QRBH-61-230-3-6.cif	24	0.718
XXXIII_structure_419.cif	XXXIII-1-MNeumann-structure_1277.cif	26	0.496
XXXIII_structure_420.cif	XXXIII-1-GDay-33_XXXIII_a_0_opt.33_XXXIII_c_0_opt-QRBH-15-201-3-59.cif	19	0.526
XXXIII_structure_421.cif	XXXIII-1-XtalPi-data_1094_Z1_st_YD2kLi1FJgABE1VK.cif	30	0.494
XXXIII_structure_422.cif	XXXIII-1-GDay-33_XXXIII_a_0_opt.33_XXXIII_c_0_opt-QRBH-14-29844-3-0.cif	17	0.658
XXXIII_structure_423.cif	XXXIII-1-GDay-33_XXXIII_a_0_opt.33_XXXIII_c_0_opt-QRBH-14-21527-3-0.cif	26	0.613
XXXIII_structure_424.cif	XXXIII-1-MNeumann-structure_81.cif	30	0.435

XXXIII_structure_425.cif	XXXIII-1-GDay-33_XXXIII_a_0_opt.33_XXXIII_c_0_opt-QRBH-14-45090-3-0.cif	30	0.450
XXXIII_structure_426.cif	XXXIII-1-GDay-33_XXXIII_a_0_opt.33_XXXIII_c_0_opt-QRBH-14-45251-3-0_pl.cif	30	0.303
XXXIII_structure_427.cif	XXXIII-1-GDay-33_XXXIII_a_11_opt.33_XXXIII_c_0_opt-QRBH-14-15939-3-0.cif	26	0.533
XXXIII_structure_428.cif	XXXIII-1-GDay-33_XXXIII_a_0_opt.33_XXXIII_c_0_opt-QRBH-14-9556-3-2.cif	30	0.460
XXXIII_structure_429.cif	XXXIII-1-GDay-33_XXXIII_a_2_opt.33_XXXIII_c_0_opt-QRBH-19-19837-3-0.cif	30	0.439
XXXIII_structure_430.cif	XXXIII-1-MNeumann-structure_1356.cif	30	0.340
XXXIII_structure_431.cif	XXXIII-1-GDay-33_XXXIII_a_0_opt.33_XXXIII_c_0_opt-QRBH-14-23782-3-1.cif	30	0.433
XXXIII_structure_432.cif	XXXIII-1-SLPrice-XXXIII_dfBb884_Bb884.cif	30	0.437
XXXIII_structure_433.cif	XXXIII-1-GDay-33_XXXIII_a_10_opt.33_XXXIII_c_0_opt-QRBH-14-13171-3-0.cif	30	0.442
XXXIII_structure_434.cif	XXXIII-1-GDay-33_XXXIII_a_0_opt.33_XXXIII_c_0_opt-QRBH-2-8409-3-0.cif	30	0.526
XXXIII_structure_435.cif	XXXIII-1-GDay-33_XXXIII_a_6_opt.33_XXXIII_c_0_opt-QRBH-61-48-3-69.cif	30	0.645
XXXIII_structure_436.cif	XXXIII-1-GDay-33_XXXIII_a_2_opt.33_XXXIII_c_0_opt-QRBH-14-88-3-8.cif	30	0.380
XXXIII_structure_437.cif	XXXIII-1-GDay-33_XXXIII_a_4_opt.33_XXXIII_c_0_opt-QRBH-2-24-3-3.cif	15	0.611
XXXIII_structure_438.cif	XXXIII-1-GDay-33_XXXIII_a_0_opt.33_XXXIII_c_0_opt-QRBH-61-156-3-49.cif	30	0.435
XXXIII_structure_439.cif	XXXIII-1-GDay-33_XXXIII_a_10_opt.33_XXXIII_c_0_opt-QRBH-14-25430-3-0.cif	30	0.372
XXXIII_structure_440.cif	XXXIII-1-GDay-33_XXXIII_a_0_opt.33_XXXIII_c_0_opt-QRBH-19-4515-3-0.cif	30	0.353
XXXIII_structure_441.cif	XXXIII-1-XtalPi-data_448_Z1_st_YD2kLi1FJgABE1ap.cif	30	0.429
XXXIII_structure_442.cif	XXXIII-1-MNeumann-structure_612.cif	30	0.337
XXXIII_structure_443.cif	XXXIII-1-GDay-33_XXXIII_a_2_opt.33_XXXIII_c_0_opt-QRBH-14-30938-3-1.cif	19	0.769
XXXIII_structure_444.cif	XXXIII-1-SLPrice-XXXIII_dfBb1804_Bb1804.cif	16	1.072
XXXIII_structure_445.cif	XXXIII-1-GDay-33_XXXIII_a_0_opt.33_XXXIII_c_0_opt-QRBH-61-671-3-3_pl.cif	30	0.434
XXXIII_structure_446.cif	XXXIII-1-MNeumann-structure_1299.cif	30	0.326
XXXIII_structure_447.cif	XXXIII-1-MNeumann-structure_1204.cif	30	0.406
XXXIII_structure_448.cif	XXXIII-1-GDay-33_XXXIII_a_0_opt.33_XXXIII_c_0_opt-QRBH-14-952-3-6.cif	30	0.407
XXXIII_structure_449.cif	XXXIII-1-SLPrice-XXXIII_dfCb50_Cb50.cif	30	0.387
XXXIII_structure_450.cif	XXXIII-1-MNeumann-structure_133.cif	27	0.640
XXXIII_structure_451.cif	XXXIII-1-GDay-33_XXXIII_a_0_opt.33_XXXIII_c_0_opt-QRBH-19-8152-3-0.cif	30	0.528
XXXIII_structure_452.cif	*XXXIII-1-MNeumann-structure_1.cif	30	0.300
XXXIII_structure_453.cif	XXXIII-1-GDay-33_XXXIII_a_2_opt.33_XXXIII_c_0_opt-QRBH-2-2972-3-5.cif	29	0.627
XXXIII_structure_454.cif	XXXIII-1-SLPrice-XXXIII_dfBa133_Ba133.cif	30	0.485
XXXIII_structure_455.cif	XXXIII-1-MNeumann-structure_405.cif	30	0.699
XXXIII_structure_456.cif	XXXIII-1-GDay-33_XXXIII_a_0_opt.33_XXXIII_c_0_opt-QRBH-14-24838-3-0.cif	25	0.468
XXXIII_structure_457.cif	XXXIII-1-GDay-33_XXXIII_a_6_opt.33_XXXIII_c_0_opt-QRBH-14-5-3-210.cif	30	0.485
XXXIII_structure_458.cif	XXXIII-1-GDay-33_XXXIII_a_11_opt.33_XXXIII_c_0_opt-QRBH-19-4616-3-0.cif	30	0.515
XXXIII_structure_459.cif	XXXIII-1-GDay-33_XXXIII_a_2_opt.33_XXXIII_c_0_opt-QRBH-14-2650-3-0.cif	23	0.681
XXXIII_structure_460.cif	XXXIII-1-GDay-33_XXXIII_a_4_opt.33_XXXIII_c_0_opt-QRBH-2-34753-3-0.cif	30	0.284
XXXIII_structure_461.cif	XXXIII-1-XtalPi-data_1321_Z1_st_YUW1ABsYUp3Zdb1M.cif	30	0.480
XXXIII_structure_462.cif	XXXIII-1-GDay-33_XXXIII_a_2_opt.33_XXXIII_c_0_opt-QRBH-14-42908-3-0.cif	30	0.475
XXXIII_structure_463.cif	XXXIII-1-GDay-33_XXXIII_a_0_opt.33_XXXIII_c_0_opt-QRBH-14-15794-3-0.cif	30	0.407
XXXIII_structure_464.cif	XXXIII-1-SLPrice-XXXIII_dfBb1289_Bb1289.cif	30	0.379

XXXIII_structure_465.cif	XXXIII-1-GDay-33_XXXIII_a_6_opt.33_XXXIII_c_0_opt-QRBH-15-294-3-1.cif	27	0.474
XXXIII_structure_466.cif	XXXIII-1-GDay-33_XXXIII_a_0_opt.33_XXXIII_c_0_opt-QRBH-14-12173-3-0.cif	30	0.735
XXXIII_structure_467.cif	XXXIII-1-GDay-33_XXXIII_a_10_opt.33_XXXIII_c_0_opt-QRBH-14-33844-3-0.cif	26	0.523
XXXIII_structure_468.cif	XXXIII-1-MNeumann-structure_1033.cif	30	0.424
XXXIII_structure_469.cif	XXXIII-1-GDay-33_XXXIII_a_27_opt.33_XXXIII_c_0_opt-QRBH-14-7516-3-0_pl.cif	30	0.380
XXXIII_structure_470.cif	XXXIII-1-GDay-33_XXXIII_a_0_opt.33_XXXIII_c_0_opt-QRBH-14-10982-3-1.cif	26	0.606
XXXIII_structure_471.cif	XXXIII-1-SLPrice-XXXIII_dfBa1252_Ba1252.cif	30	0.459
XXXIII_structure_472.cif	XXXIII-1-GDay-33_XXXIII_a_10_opt.33_XXXIII_c_0_opt-QRBH-15-245-3-10.cif	30	0.545
XXXIII_structure_473.cif	XXXIII-1-GDay-33_XXXIII_a_10_opt.33_XXXIII_c_0_opt-QRBH-14-8526-3-0.cif	30	0.441
XXXIII_structure_474.cif	XXXIII-1-GDay-33_XXXIII_a_11_opt.33_XXXIII_c_0_opt-QRBH-4-841-3-4.cif	19	1.122
XXXIII_structure_475.cif	XXXIII-1-GDay-33_XXXIII_a_0_opt.33_XXXIII_c_0_opt-QRBH-29-99-3-2.cif	30	0.540
XXXIII_structure_476.cif	XXXIII-1-GDay-33_XXXIII_a_2_opt.33_XXXIII_c_0_opt-QRBH-2-4614-3-0.cif	17	0.951
XXXIII_structure_477.cif	XXXIII-1-MNeumann-structure_1009.cif	30	0.433
XXXIII_structure_478.cif	XXXIII-1-GDay-33_XXXIII_a_2_opt.33_XXXIII_c_0_opt-QRBH-2-2659-3-3_pl.cif	30	0.394
XXXIII_structure_479.cif	XXXIII-1-GDay-33_XXXIII_a_0_opt.33_XXXIII_c_0_opt-QRBH-2-14756-3-0.cif	23	0.604
XXXIII_structure_480.cif	XXXIII-1-MNeumann-structure_827.cif	30	0.346
XXXIII_structure_481.cif	XXXIII-1-GDay-33_XXXIII_a_6_opt.33_XXXIII_c_0_opt-QRBH-19-16569-3-1.cif	30	0.467
XXXIII_structure_482.cif	XXXIII-1-MNeumann-structure_897.cif	30	0.366
XXXIII_structure_483.cif	XXXIII-1-GDay-33_XXXIII_a_0_opt.33_XXXIII_c_0_opt-QRBH-14-46448-3-1.cif	30	0.331
XXXIII_structure_484.cif	XXXIII-1-GDay-33_XXXIII_a_0_opt.33_XXXIII_c_0_opt-QRBH-14-33771-3-0.cif	30	0.684
XXXIII_structure_485.cif	XXXIII-1-GDay-33_XXXIII_a_0_opt.33_XXXIII_c_0_opt-QRBH-61-37-3-13.cif	30	0.336
XXXIII_structure_486.cif	XXXIII-1-GDay-33_XXXIII_a_2_opt.33_XXXIII_c_0_opt-QRBH-14-44019-3-0.cif	30	0.535
XXXIII_structure_487.cif	XXXIII-1-GDay-33_XXXIII_a_0_opt.33_XXXIII_c_0_opt-QRBH-14-11-3-124.cif	30	0.435
XXXIII_structure_488.cif	XXXIII-1-GDay-33_XXXIII_a_10_opt.33_XXXIII_c_0_opt-QRBH-14-16868-3-7.cif	30	0.398
XXXIII_structure_489.cif	XXXIII-1-GDay-33_XXXIII_a_6_opt.33_XXXIII_c_0_opt-QRBH-33-906-3-1.cif	30	0.548
XXXIII_structure_490.cif	XXXIII-1-GDay-33_XXXIII_a_0_opt.33_XXXIII_c_0_opt-QRBH-33-82-3-7.cif	30	0.227
XXXIII_structure_491.cif	XXXIII-1-MNeumann-structure_303.cif	30	0.424
XXXIII_structure_492.cif	XXXIII-1-GDay-33_XXXIII_a_0_opt.33_XXXIII_c_0_opt-QRBH-14-23151-3-0.cif	30	0.427
XXXIII_structure_493.cif	XXXIII-1-MNeumann-structure_1446.cif	30	0.397
XXXIII_structure_494.cif	XXXIII-1-MNeumann-structure_1249.cif	27	0.663
XXXIII_structure_495.cif	XXXIII-1-MNeumann-structure_1235.cif	30	0.393
XXXIII_structure_496.cif	XXXIII-1-GDay-33_XXXIII_a_10_opt.33_XXXIII_c_0_opt-QRBH-14-6141-3-3.cif	25	0.415
XXXIII_structure_497.cif	XXXIII-1-MNeumann-structure_1144.cif	30	0.340
XXXIII_structure_498.cif	XXXIII-1-GDay-33_XXXIII_a_10_opt.33_XXXIII_c_0_opt-QRBH-14-20769-3-0.cif	30	0.379
XXXIII_structure_499.cif	XXXIII-1-GDay-33_XXXIII_a_8_opt.33_XXXIII_c_0_opt-QRBH-14-15697-3-0.cif	30	0.493
XXXIII_structure_500.cif	XXXIII-1-SLPrice-XXXIII_dfBb125_Bb125.cif	24	0.552

3. Additional Tables

Table 8. *Analysis of void space present in the predicted structures of compound XXVII generated by each group from the structure generation phase of the blind test (calculated using the CSD Python API with 1.2 Å probe radius and 0.3 Å grid spacing)*

Group	Average (Å^3)	Standard dev. (Å^3)
5	4.67	2.3
6	3.28	2.16
8	14.82	4.76
10	0.18	0.45
12	78.88	11.9
16	1.07	1.44
17	26.3	14.75
19	7.88	3.83
20	0.16	0.38
21	3.47	2.34
22	10.74	5.94
24	5.58	3.65
25	1.46	1.57
26	11.55	4.62
28	2.19	1.95

Table 9. *Structural matches upon comparison of XXVII Form A (core only) with the 100 structures provided by CCDC for submission 2 (structure ranking).*

CIF name	No. molecules matched (/30)	RMSD
XXVII_structure_28.cif	30	0.530013
XXVII_structure_38.cif	30	0.803538
XXVII_structure_59.cif	30	0.828099
XXVII_structure_61.cif	30	0.568538

Table 10. *Average PDD distance (Å) between structures optimised by each group and the original ones provided by the CCDC.*

	XXXI	XXVII	XXVIII	XXXII	XXXIII
Group 2	0.21885			0.25773	
Group 3	0.20999	0.35530	0.34857	0.18206	0.38592
Group 4	0.14929			0.17107	0.39780
Group 5	0.11237	0.18159		0.15926	0.39188
Group 6	0.27048	0.27912	0.39739	0.36455	0.40858
Group 7	0.00000				
Group 9	0.17532	0.25341			0.40400
Group 10	0.21951	0.26533	0.22952	0.20520	0.40885
Group 11	0.19673	0.25042	0.29980	0.17899	0.37397
Group 12	0.00000	0.00000	0.00000	0.00000	0.00000
Group 14	0.19129				
Group 15	0.29477	0.30473	0.39585	0.27276	0.36309
Group 16	0.24998	0.26621		0.23785	0.36437
Group 17		0.31342			
Group 18	0.29312				0.39499
Group 19	0.24388			0.09552	0.00008
Group 20	0.23740	0.28228	0.37974	0.19215	0.41553
Group 21	0.21314	0.26098		0.38013	0.39175
Group 22	0.04404	0.06370	0.13034	0.05821	0.30187
Group 24	0.24013	0.17927	0.26488	0.30651	0.39480
Group 26	0.35269	0.27257	0.33977	0.25431	
Group 27					0.44106

4. Rankings of the Optimised Structures

Table 11. *Summary of relative lattice and free energies of structures 28, 38, 59 and 61 of molecule XXVII. These structures initially matched the core packing of Form A. The number of matching molecules and RMSD is also given to identify possible major distortions of the structure after optimisation. Each Group entry is ordered by the best match to the experimental form ($RMSD_{30}$). Structures 28 and 61 were sometimes considered duplicates.*

Group	Rank (lattice energy)	Relative lattice energy (kJ/mol)	Rank (Free energy)	Relative Free energy (kJ/mol)	Free energy tempera-ture (K)	Structure label	Molecules match (/30)	RMSD (Å)
3	63	17.64				Structure 38	30	0.40
	8	4.18	1	0.00	300	Structure 28	30	0.50
	7	4.02	2	2.55	300	Structure 61	30	0.58
	35	8.36				Structure 59	30	0.78
5	1	0.00				Structures 28, 61	30	0.45
	61	21.83				Structure 38	30	0.59
	8	5.96				Structure 59	30	0.82
6	3	1.22				Structure 61	30	0.62
	2	1.15				Structure 28	30	0.62
	4	1.62				Structure 38	30	0.79
	8	3.02				Structure 59	16	1.52
9	9	12.86				Structure 28	30	0.37
	39	19.06				Structure 61	30	0.40
	67	28.59				Structure 38	30	0.45
	1	0.00				Structure 59	30	0.75
10	5	3.63	3	0.26	300	Structure 61	30	0.42
	4	3.46	4	0.57	300	Structure 28	30	0.43
	59	18.62	41	11.03	300	Structure 38	30	0.43
	23	7.46	15	3.85	300	Structure 59	30	0.82
11	64	17.12				Structure 38	30	0.38
	3	1.34				Structure 28	30	0.43
	2	1.34				Structure 61	30	0.43
	29	8.00				Structure 59	30	0.82
12	42	657.33				Structure 28	30	0.53
	40	655.11				Structure 61	30	0.57
	93	798.96				Structure 38	30	0.80
	25	644.46				Structure 59	30	0.83
15			82	62.04	300	Structure 61	30	0.55
			83	62.06	300	Structure 38	30	0.65
			68	56.37	300	Structure 28	30	0.80
			56	54.30	300	Structure 59	29	0.82
16	1	0.00	1	0.00	300	Structure 28	30	0.54
	4	2.40	3	1.20	300	Structure 61	30	0.57
	3	0.50	4	1.90	300	Structure 59	30	1.13
	78	28.90	80	33.70	300	Structure 38	28	0.53
17	92	893.79	70	156.65	300	Structure 38	30	0.80
	40	664.64	47	123.65	300	Structure 61	30	1.22
	43	671.33	37	109.87	300	Structure 28	30	1.23
	23	598.54	21	64.61	300	Structure 59	16	2.09
20			57	17.47	298.15	Structure 38	30	0.40
			2	1.53	298.15	Structures 28, 61	30	0.44
			95	35.53	298.15	Structure 51*	30	0.51
			14	4.90	298.15	Structure 59	30	0.79
21	4	3.28				Structure 28	30	0.39
	62	17.10				Structure 38	30	0.41
	4	3.28				Structures 28, 61	30	0.41
	27	7.13				Structure 59	30	0.78
22	2	0.97				Structure 28	30	0.52
	3	1.78				Structure 61	30	0.56
	67	25.29				Structure 38	30	0.76
	11	5.49				Structure 59	30	0.82
24	1	0.00	1	0.00	298	Structures 28, 61	30	0.39
	61	30.96	61	29.44	298	Structure 38	30	0.49
	8	3.58	8	4.02	298	Structure 59	30	0.68
26	3	3.00				Structure 28	30	0.44
	4	4.30				Structure 61	30	0.47
	70	49.40				Structure 38	30	0.55
	11	6.50				Structure 59	30	0.84

Table 12. *The predicted lattice energy rank, relative lattice energy, Free energy rank and relative Free energy of structures matching the experimental form A of XXVIII. The results of the comparison, number of molecules matched and RMSD, between the experimental form and reported structure are also reported. In cases where a structural match was not found, the closest match is reported.*

Group	Lattice energy rank	Relative lattice energy	Free energy rank	Relative Free energy	Structure	No. matching molecules	RMSD (Å)
3	1	0	1	0	XXVIII_structure_144.cif	30	0.212088
6	4	2.73	-	-	data_vaneijck-XXVIII_structure_144.cif	3	1.502581
10	1	0	1	0	XXVIII_structure_144_X1.cif	30	0.261232
11	1	0	-	-	XXVIII-144.cif	30	0.129576
12	63	582.7163	-	-	XXVIII_structure_144.cif	30	0.234628
15	-	-	85	13.9027	XXVIII_structure_145.cif	30	0.58148
	-	-	384	32.6809	XXVIII_structure_437.cif	30	0.628299
	-	-	325	25.4641	XXVIII_structure_333.cif	30	0.630653
	-	-	309	24.3045	XXVIII_structure_144.cif	30	0.63528
	-	-	329	25.6059	XXVIII_structure_171.cif	30	0.636703
			331	25.7005	XXVIII_structure_412.cif	30	0.655948
20	1	0	1	0	XXVIII_structure_144.cif	30	0.139829
22	3	2.2	-	-	XXVIII_structure_144.cif	30	0.224219
24	6	4.763	6	4.823	XXVIII_structure_207-_SLPrice.cif (= structure 144)	30	0.416072
26	1	0	-	-	XXVIII_structure_144.cif	30	0.200904
	7	11.9017	-	-	XXVIII_structure_333.cif	30	0.354948
	17	16.5579	-	-	XXVIII_structure_207.cif	30	0.530389
	46	24.9193	-	-	XXVIII_structure_145.cif	30	0.599725

Table 13. *The predicted lattice energy rank, relative lattice energy, Free energy rank and relative Free energy of structures matching the Form A (major disorder component) of XXXI. The results of the comparison, number of molecules matched and RMSD, between the experimental form and reported structure are also reported. In cases where a structural match was not found, the closest match is reported.*

Group	Lattice energy rank	Relative lattice energy	Free energy rank	Relative Free energy	Structure	No. matching molecules	RMSD (Å)
2	7	2.245	-	-	XXXI_structure_98.cif	30	0.10863
3	3	2.07	3	1.06	XXXI_structure_98.cif	30	0.189479
4	6	19.89897	6	8.144228	XXXI-2-CCERVINKA-098.cif	30	0.172978
5	2	0.28218	-	-	XXXI_structure_98.cif	30	0.166449
6	-	-	-	-	data_vaneijck-XXXI_structure_98.cif	8	0.761748
7	*	*	*	*	XXXI_structure_98.cif	30	0.18033
9	5	1.2809	-	-	XXXI_structure_98-out.cif	30	0.201385
10	8	1.32398	7	1.837348	XXXI_structure_98_X8.cif	30	0.136694
11	8	3.9152	-	-	XXXI-98.cif	30	0.128786
12	33	21.0217	-	-	XXXI_structure_98.cif	30	0.18033
14	6	2.972	-	-	XXXI_structure_98.cif	30	0.086232
15	-	-	18	9.4279	XXXI_structure_98.cif	30	0.636414
16	2	0.3	3	1.8	MI_XXXI_structure_98.cif	30	0.214683
18	4	5.9987	-	-	structure_98.cif	30	0.32086
19	2	17.68	-	-	XXXI_structure_98.cif	30	0.398067
20	-	-	10	2.140247	XXXI_structure_98.cif	30	0.108373
21	15	13.004	-	-	XXXI_structure_98_R0015.cif	30	0.131216
22	2	1.47	-	-	XXXI_structure_98.cif	30	0.177209
24	5	2.242	10	1.585	XXXI_structure_98_SLPrice.cif	30	0.17135
26	22	7.534	-	-	098.cif	30	0.422038

Table 14. *The predicted lattice energy rank, relative lattice energy, Free energy rank and relative Free energy of structures matching the Form A (minor disorder component) of XXXI. The results of the comparison, number of molecules matched and RMSD, between the experimental form and reported structure are also reported. In cases where a structural match was not found, the closest match is reported.*

Group	Lattice energy rank	Relative lattice energy	Free energy rank	Relative Free energy	Structure	No. matching molecules	RMSD (Å)
2	9	2.478			XXXI_structure_1.cif	30	0.246677
3	10	3.94	5	2.24	XXXI_structure_1.cif	30	0.184704
4	3	15.53522	2	4.202544	XXXI-2-CCERVINKA-001.cif	30	0.259425
5	3	0.98806			XXXI_structure_1.cif	30	0.279014
6	14	7.641			data_vaneijck-XXXI_structure_1.cif	30	0.264902
7	36	-	-	-	XXXI_structure_1.cif	30	0.332919
9	7	1.7223	-	-	XXXI_structure_1-out.cif	30	0.252629
10	11	1.996238	8	1.864968	XXXI_structure_1_X11.cif	30	0.255272
11	12	5.626	-	-	XXXI-1.cif	30	0.257718
12	38	22.5485	-	-	XXXI_structure_1.cif	30	0.332919
14	10	4.004	-	-	XXXI_structure_1.cif	30	0.254301
15	-	-	13	8.7706	XXXI_structure_1.cif	30	0.307094
16	4	0.6	18	5.5	MI_XXXI_structure_1.cif	30	0.240942
18	5	6.1365	-	-	structure_1.cif	30	0.410694
19	3	21	-	-	-	-	-
20	-	-	11	2.328182	XXXI_structure_1.cif	30	0.260392
21	22	14.316	-	-	XXXI_structure_1_R0022.cif	30	0.280465
22	5	2.95	-	-	XXXI_structure_1.cif	30	0.315046
24	2	1.587	2	0.923	XXXI_structure_1_SLPrice.cif	30	0.328882
25	34	10.191	-	-	001.cif	30	0.484473

Table 15. *The predicted lattice energy rank, relative lattice energy, Free energy rank and relative Free energy of structures matching the Form B of XXXI. The results of the comparison, number of molecules matched and RMSD, between the experimental form and reported structure are also reported. In cases where a structural match was not found, the closest match is reported.*

Group	Lattice energy rank	Relative lattice energy	Free energy rank	Relative Free energy	Structure	No. matching molecules	RMSD (Å)
2	17	4.838			XXXI_structure_25.cif	30	0.201614
3	6	3.56	1	0	XXXI_structure_25.cif	30	0.24353
4	9	22.1985	7	9.563817	XXXI-2-CCERVINKA-025.cif	30	0.24797
5	6	1.84344			XXXI_structure_25.cif	30	0.274936
6	33	12.382	-	-	data_vaneijck-XXXI_structure_25.cif	29	0.760249
7	26	-	-	-	XXXI_structure_25.cif	30	0.247562
9	8	1.8815	-	-	XXXI_structure_25-out.cif	30	0.322147
10	1	0	3	0.740691	XXXI_structure_25_X1.cif	30	0.257551
11	13	5.7032	-	-	XXXI-25.cif	30	0.203334
12	12	13.6381	-	-	XXXI_structure_25.cif	30	0.247562
14	11	4.215	-	-	XXXI_structure_25.cif	30	0.215527
15	-	-	12	8.7619	XXXI_structure_25.cif	30	0.418807
16	10	1.9	10	4.4	MI_XXXI_structure_25.cif	30	0.479741
18	12	7.1191	-	-	structure_25.cif	14	1.308567
19	82	49.07	-	-	XXXI_structure_25.cif	21	0.535955
20	-	-	6	1.217822	XXXI_structure_25.cif	30	0.216433
21	4	5.903	-	-	XXXI_structure_25_R0004.cif	30	0.259745
22	10	4.47	-	-	XXXI_structure_25.cif	30	0.246165
24	47	8.42	43	6.934	XXXI_structure_25_SLPrice.cif	30	0.43205
25	1	0	-	-	025.cif	14	1.95375

Table 16. *The predicted lattice energy rank, relative lattice energy, Free energy rank and relative Free energy of structures matching the Form A (major disorder component) of XXXII. The results of the comparison, number of molecules matched and RMSD, between the experimental form and reported structure are also reported. In cases where a structural match was not found, the closest match is reported.*

Group	Lattice energy rank	Relative lattice energy	Free energy rank	Relative Free energy	Structure	No. matching molecules	RMSD (Å)
2	27	6.096	-	-	XXXII_structure_317.cif	30	0.340572
3	18	7.38	24	6.57	XXXII_structure_317.cif	30	0.147755
4	64	550.9581	30	308.0751	XXXII-2-CCERVINKA-317.cif	30	0.221951
5	9	5.433725	-	-	XXXII_317_final_ccdc.cif	30	0.231347
6	473	167.414			data_vaneijck-XXXII_structure_317.cif	12	0.948142
10	13	5.80893	5	4.394919	XXXII_structure_317_X13.cif	30	0.215423
11	31	12.176	-	-	XXXII-317.cif	30	0.16094
12	490	339.4415	-	-	XXXII_structure_317.cif	30	0.26489
15	18	4.1471	-	-	XXXII_structure_317.cif	30	0.636974
16	41	12.7	38	15.9	MI_XXXII_structure_317.cif	30	0.231971
19	337	37.73	-	-	XXXII_structure_317.cif	30	0.289948
20	-	-	11	6.028429	XXXII_structure_317.cif	30	0.154367
21	62	20.075	-	-	XXXII_structure_317_R0062.cif	30	0.564831
22	21	6.95	-	-	XXXII_structure_317.cif	30	0.25899
24	209	22.844	195	21.521	XXXII_structure_317_SLPrice.cif	30	0.47994
26	23	21.2304			XXXII_structure_317.cif	30	0.261

Table 17. *The predicted lattice energy rank, relative lattice energy, Free energy rank and relative Free energy of structures matching the Form B (low temperature structure) of XXXII. The results of the comparison, number of molecules matched and RMSD, between the experimental form and reported structure are also reported. In cases where a structural match was not found, the closest match is reported.*

Group	Lattice energy rank	Relative lattice energy	Free energy rank	Relative Free energy	Structure	No. matching molecules	RMSD (Å)
2	30	6.531	-	-	XXII_structure_232.cif	30	0.257338
3	22	8.98	25	6.81	XXXII_structure_232.cif	30	0.288666
4	76	552.9992	147	317.7815	XXXII-2-CCERVINKA-232.cif	30	0.267036
5	24	7.66161	-	-	XXXII_232_final_ccdc.cif	30	0.275266
6	86	40.666			data_vaneijck-XXXII_structure_232.cif	17	0.781157
10	30	9.63929	51	10.94875	XXXII_structure_232_X30.cif	30	0.248026
11	37	12.7747	-	-	XXXII-232.cif	30	0.207925
12	129	31.1478	-	-	XXXII_structure_232.cif	30	0.298565
15	6	2.4609			XXXII_structure_232.cif	20	0.46
16	3	6.5	15	12.9	MI_XXXII_structure_232.cif	30	0.353437
19	82	20.8	-	-	XXXII_structure_232.cif	30	0.298565
20	-	-	35	12.05909	XXXII_structure_232.cif	30	0.293622
21	*	*	-	-	XXXII_structure_232_NA.cif	30	0.615035
22	30	7.78	-	-	XXXII_structure_232.cif	30	0.270725
24	41	11.382	42	11.018	XXXII_structure_232_SLPrice.cif	30	0.429981
26	487	284.191	-	-	XXXII_structure_232.cif	30	0.298565

Table 18. *The predicted lattice energy rank, relative lattice energy, Free energy rank and relative Free energy of structures matching the Form A of XXXIII. The results of the comparison, number of molecules matched and RMSD, between the experimental form and reported structure are also reported. In cases where a structural match was not found, the closest match is reported.*

Group	Lattice energy rank	Relative lattice energy	Free energy rank	Relative Free energy	Structure	No. matching molecules	RMSD (Å)
3	6	5.74	4	5.65	XXXIII_structure_233.cif	30	0.166224
4	50	184.4713	60	158.3308	XXXIII-2-CCERVINKA-233.cif	30	0.285447
5	4	8.439	-	-	XXXIII_structure_233.cif	30	0.279526
6	205	35.554	-	-	data_vaneijck-XXXIII_structure_233.cif	30	0.327886
9	9	9.7476	-	-	XXXIII_structure_233-out.cif	30	0.193659
10	7	7.54304	4	11.35059	XXXIII_structure_233_X7.cif	30	0.192441
11	5	5.1651	-	-	XXXIII-233.cif	30	0.223867
12	90	586.0463	-	-	XXXIII_structure_233.cif	30	0.387299
15	202	52.1604			XXXIII_structure_233.cif	17	0.847568
16	60	9.2	56	10.8	MI_XXXIII_structure_233.cif	30	0.359699
18	29	10.015	-	-	structure_233.cif	30	0.306177
19	214	105.92	-	-	XXXIII_structure_233.cif	30	0.404044
20	-	-	2	5.04709	XXXIII_structure_233.cif	30	0.114399
21	33	41.186	-	-	XXXIII_structure_233_R0033.cif	30	0.124116
22	3	6.12	-	-	XXXIII_structure_233.cif	30	0.193811
24	4	2.038	6	2.709	XXXIII_structure_233_SLPrice.cif	30	0.387951
27	349	209.3133	-	-	XXXIII_structure_233.cif	30	0.322987

Table 19. *The predicted lattice energy rank, relative lattice energy, Free energy rank and relative Free energy of structures matching the Form B of XXXIII. The results of the comparison, number of molecules matched and RMSD, between the experimental form and reported structure are also reported. In cases where a structural match was not found, the closest match is reported.*

Group	Lattice energy rank	Relative lattice energy	Free energy rank	Relative Free energy	Structure	No. matching molecules	RMSD (Å)
3	1	0	1	0	XXXIII_structure_452.cif	30	0.20964
4	28	171.138	30	145.7403	XXXIII-2-CCERVINKA-452.cif	30	0.207682
5	1	0	-	-	XXXIII_structure_452.cif	30	0.192714
6	3	3.991	-	-	data_vaneijck-XXXIII_structure_452.cif	30	0.516615
9	1	0	-	-	XXXIII_structure_452-out.cif	30	0.171294
10	1	0	1	0	XXXIII_structure_452_X1.cif	30	0.185689
11	1	0	-	-	XXXIII-452.cif	30	0.194364
12	470	1072.453	-	-	XXXIII_structure_452.cif	30	0.254359
15	288	105.5381	-	-	XXXIII_structure_452.cif	30	0.365467
16	20	5.6	20	6.9	MI_XXXIII_structure_452.cif	30	0.196697
18	22	8.142	-	-	structure_452.cif	30	0.448687
19	14	53.39	-	-	XXXIII_structure_452.cif	30	0.252164
20	-	-	1	0	XXXIII_structure_452.cif	30	0.214609
21	302	79.092	-	-	XXXIII_structure_452_R0302.cif	30	0.202159
22	1	0	-	-	XXXIII_structure_452.cif	30	0.147017
24	20	11.635	19	11.852	XXXIII_structure_236_SLPrice.cif	19	0.720985
27	132	169.9331	-	-	XXXIII_structure_452.cif	30	0.506723

5. Unknown XXVII and XXXII Forms

Table 20: (Target XXVII) The CIFs originating from submissions in phase one (structure generation exercise) of the seventh blind test that showed the highest PXRD similarity to the experimental PXRD patterns B of XXVII, calculated using the PXRD similarity tool in Mercury utilising the de Gelder algorithm. The structures were included in the CCDC-prepared lists for the ranking exercise, see Table 3.

Form	Originating CIF name
	XXVII-1-SOHG-XXVII-0906.cif
	XXVII-1-XtalPi-data_1220_Z1_st_YT8AefePXwAwmo6O.cif
	XXVII-1-XtalPi-data_266_Z2_st_YUqI9l_5MQA_UFPT.cif
	XXVII-1-GDay-27_XXVII_592_gopt-QR-14-3619-3.cif
	XXVII-1-SOHG-XXVII-1335.cif
	XXVII-1-DWMHofmann-conf11_rank3-63.cif
	XXVII-1-BEijck-data_vaneijck-XXVII.1426.cif
B	XXVII-1-BEijck-data_vaneijck-XXVII.0407.cif
	XXVII-1-SOHG-XXVII-0724.cif
	XXVII-1-SOHG-XXVII-0906.cif
	XXVII-1-SOHG-XXVII-1344.cif
	XXVII-1-XtalPi-data_341_Z1_st_YT8AefePXwAwmpDQ.cif
	XXVII-1-XtalPi-data_648_Z2_st_YUqI9l_5MQA_UFPV.cif
	XXVII-1-XtalPi-data_719_Z2_st_YUqI9l_5MQA_UFEu.cif
	XXVII-1-XtalPi-data_734_Z1_st_YT8AefePXwAwmo4C.cif

Table 21: (Target XXXII) The CIFs originating from submissions in phase one (structure generation exercise) of the seventh blind test that showed the highest PXRD similarity to the experimental PXRD patterns H, K, L, N, P and R of XXXII, calculated using the PXRD similarity tool in Mercury utilising the de Gelder algorithm. The structures were included in the CCDC-prepared lists for the ranking exercise, see Table 6.

Form	Originating CIF name
H	XXXII-1-XtalPi-data_492_z1_st_YD8t7RjU-QABeTau.cif<br>XXXII-1-MNeumann-structure_784.cif<br>XXXII-1-XtalPi-data_598_z1_st_YD8t7RjU-QABeTdK.cif
K	XXXII-1-QZhu-data_1210-SG-14.cif<br>XXXII-1-XtalPi-data_1437_z2_st_YXE5cuqPPis2KGEy.cif<br>XXXII-1-AOganov-data_1342.cif<br>XXXII-1-CSAdjiman-CSOFM_01100.cif
L	XXXII-1-CSAdjiman-CSOFM_00178.cif<br>XXXII-1-CShang-XXXII_64.cif<br>XXXII-1-AOganov-data_794.cif<br>XXXII-1-AOganov-data_837.cif<br>XXXII-1-AOganov-data_362.cif<br>XXXII-1-SLPrice-BXXXII_dfBAT24_BAT24.cif<br>XXXII-1-KSzalewicz-data_294.cif<br>XXXII-1-BEijck-data_vaneijck-XXXII.1045.cif<br>XXXII-1-CSAdjiman-CSOFM_00018.cif
N	XXXII-1-BEijck-data_vaneijck-XXXII.1063.cif<br>XXXII-1-BEijck-data_vaneijck-XXXII.0973.cif<br>XXXII-1-QZhu-data_208-SG-7.cif<br>XXXII-1-AOganov-data_1221.cif<br>XXXII-1-MNeumann-structure_664.cif<br>XXXII-1-QZhu-data_408-SG-14.cif<br>XXXII-1-MNeumann-structure_558.cif<br>XXXII-1-MNeumann-structure_620.cif<br>XXXII-1-MNeumann-structure_1405.cif<br>XXXII-1-MNeumann-structure_1403.cif
P	XXXII-1-AOganov-data_1322.cif<br>XXXII-1-CSAdjiman-CSOFM_00216.cif<br>XXXII-1-CSAdjiman-CSOFM_00716.cif<br>XXXII-1-AOganov-data_402.cif<br>XXXII-1-BEijck-data_vaneijck-XXXII.0663.cif<br>XXXII-1-SLPrice-BXXXII_dfGAE35_GAE35.cif<br>XXXII-1-AOganov-data_60.cif<br>XXXII-1-SLPrice-BXXXII_dfGAE24_GAE24.cif<br>XXXII-1-AOganov-data_552.cif
R	XXXII-1-AOganov-data_339.cif<br>XXXII-1-QZhu-data_785-SG-4.cif<br>XXXII-1-CShang-XXXII_730.cif<br>XXXII-1-SLPrice-BXXXII_dfGAE30_GAE30.cif<br>XXXII-1-AOganov-data_1159.cif<br>XXXII-1-SLPrice-BXXXII_dfDDF11_DDF11.cif<br>XXXII-1-AOganov-data_1131.cif<br>XXXII-1-CSAdjiman-CSOFM_00578.cif<br>XXXII-1-SLPrice-BXXXII_dfJAA25_JAA25.cif<br>XXXII-1-DBoese-xxxii-ca53_e150-s33-co-497.cif

Table 22. *Lattice or free energy (FE) difference with Form B of the most stable structures for each group in Tab 21. Most of the approaches show structures from groups H and N with energies similar to or lower than Form B. Groups are divided by the ranking approach used as shown in Tab. 2 of the main paper. Table cells are coloured in blue if the structure is more stable than form B or red if it has higher energy.*

**In these groups the structure initially matching Form B resulted in being distorted or removed from the last step of optimisation, refer to single groups SI for more details.*

ΔE with Form B (kJ mol^{-1})

Class	Group	H	K	L	N	P	R
A1	Group 4	1.591	-346.137	-354.631	-7.141	-443.335	-552.999
A1	Group 4 (FE)	-9.064	59.500	-317.782	-17.676	43.511	22.034
A1	Group 5	2.956	35.126	17.498	1.250	31.455	20.237
A1	Group 22	1.580	63.820	17.630	-1.010	30.510	19.520
A2	Group 10	1.336	36.605	16.894	1.565	32.758	15.717
A2	Group 10 (FE)	-6.296	29.533	9.127	-6.238	28.329	15.026
A2	Group 11	1.019	39.198	15.972	1.927	28.719	9.652
A3	Group 2	1.657	59.403	14.012	-2.286	7.590	-1.442
A3	Group 3	3.040	34.230	17.220	-0.660	29.160	14.680
A3	Group 3 (FE)				-4.340		
A3	Group 20 (FE)	2.560	31.851	16.138	0.494	20.943	10.781
B1	Group 19	1.550	55.970	29.420	-2.570	59.840	19.770
B2	*Group 26**	-257.164	-167.326	-230.166	-258.526	-238.604	-221.342
B3	*Group 6**	11.323	42.914	17.657	-9.800	31.026	-3.038
B3	Group 24	-5.205	28.825	7.918	-5.367	19.090	3.783
B3	Group 24 (FE)	-5.474	28.845	8.067	-5.208	16.796	2.312
C1	Group 12	-21.970	13.560	-8.558	-16.062	-6.409	-15.081
C1	*Group 15**	1.739	30.136	20.151	3.385	20.185	10.487
C1	Group 16	16.400	40.000	29.500	12.100	30.200	18.700
C1	Group 16 (FE)	9.000	39.400	23.700	9.900	32.600	12.700

Table 23. *Lattice or free energy (FE) difference with Form B of all the structures matching the PXRD pattern H. Groups are divided by the ranking approach used as shown in Tab. 2 of the main paper. Table cells are coloured in blue if the structure is more stable than form B or red if it has higher energy.*

**In these groups the structure initially matching Form B resulted in being distorted or removed from the last step of optimisation, refer to single groups SI for more details.*

Group H - ΔE with Form B (kJ mol^{-1})

Class	Group	structure-485	structure-105	structure-154
A1	Group 4	1.591	2.234	4.665
A1	Group 4 (FE)	-9.064	-7.428	0.493
A1	Group 5	2.956	2.956	6.546
A1	Group 22	2.250	1.580	5.300
A2	Group 10	1.631	1.336	4.196
A2	Group 10 (FE)	-6.296	-4.836	-3.143
A2	Group 11	1.130	1.019	1.405
A3	Group 2	2.062	2.062	1.657
A3	Group 3	3.110	3.040	4.420
A3	Group 20 (FE)	2.560	2.560	3.756
B1	Group 19	7.010	1.550	9.810
B2	*Group 26**	-239.524	-257.164	-235.322
B3	*Group 6**	13.891	14.083	11.323
B3	Group 24	-5.205	-5.205	3.177
B3	Group 24 (FE)	-5.474	-5.474	3.162
C1	Group 12	-21.970	-18.291	-16.463
C1	*Group 15**	1.739	2.408	1.784
C1	Group 16	17.900	17.800	16.400
C1	Group 16 (FE)	12.300	12.500	9.000

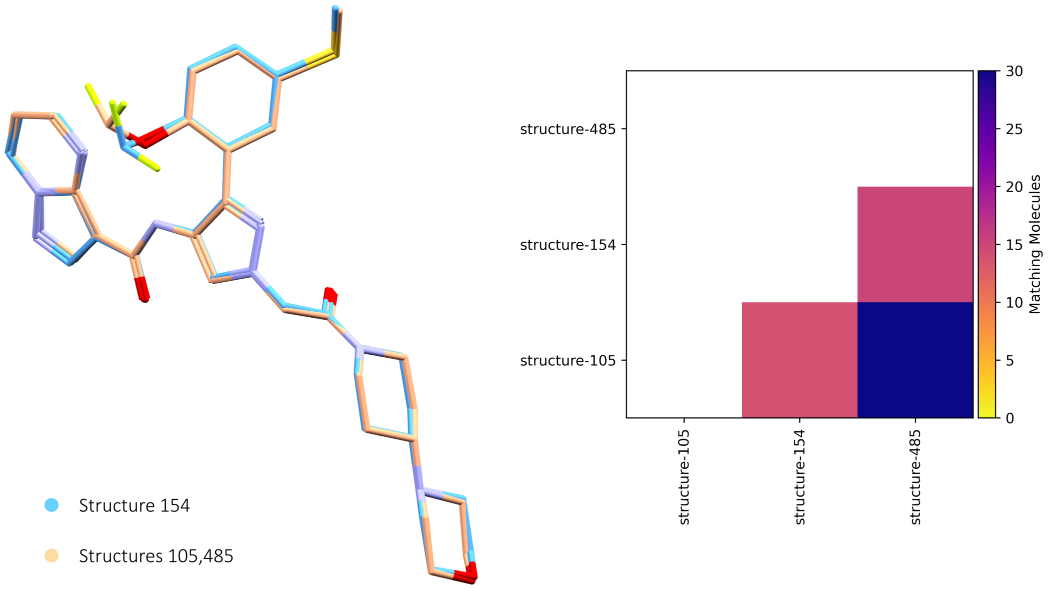

Fig. 5. (left) Main conformations of the molecules of structures 105, 154 and 485. (right) Heatmap showing the number of molecules that match in a 30-molecule overlay with the Crystal Packing Similarity tool, using 25%/25° distance/angle tolerances.

The main difference between structures 105 and 485 and structure 154 is in the conformation of the trifluoromethyl group. As the energies of these three theoretical structures are similar (Tab. 23), this could indicate that the crystals corresponding to pattern H have a similar packing with disorder of the difluoromethyl group.

Table 24. *Lattice or free energy (FE) difference with Form B of the most stable structures matching the PXRD pattern N. Groups are divided by the ranking approach used as shown in Tab. 2 of the main paper. Table cells are coloured in blue if the structure is more stable than form B or red if it has higher energy.*

* *In these groups the structure initially matching Form B resulted in being distorted or removed from the last step of optimisation, refer to single groups SI for more details.*

Group N - ΔE with Form B (kJ mol^{-1})

Class	Group	structure-442	structure-58	structure-66	structure-169	structure-488
A1	Group 4	-4.611	-7.141	-6.117	-2.932	-2.628
A1	Group 4 (FE)	-14.097	-17.676	-17.229	-12.493	-11.717
A1	Group 5	2.962	1.494	1.250	4.257	4.257
A1	Group 22	1.130	-1.010	0.760	4.930	4.140
A2	Group 10	1.891	1.565	2.066	3.357	3.317
A2	Group 10 (FE)	-6.238	-4.387	-5.875	2.507	-1.571
A2	Group 11	1.927	2.035	2.197	5.600	5.417
A3	Group 2	-2.286	-2.286	-2.286	3.295	3.295
A3	Group 3	-0.660	-0.660	-0.660	4.440	4.440
A3	Group 3 (FE)	-4.340	-4.340	-4.340		
A3	Group 20 (FE)	0.494	0.494	0.494	4.069	4.069
B1	Group 19	2.550	3.470	2.220	-1.120	-2.570
B2	*Group 26**	0.000	0.000	0.000	-258.526	-238.747
B3	*Group 6**	-9.800	-9.740	-9.386	31.876	32.571
B3	Group 24	-0.435	-5.367	3.833	1.524	1.524
B3	Group 24 (FE)	0.025	-5.208	4.270	1.871	1.871
C1	Group 12	-3.722	-5.415	-4.795	-16.062	-7.444
C1	*Group 15**	4.254	3.385	4.470	11.193	11.936
C1	Group 16	12.600	12.100	12.200	15.000	15.000
C1	Group 16 (FE)	9.900	10.200	11.200	11.100	10.600

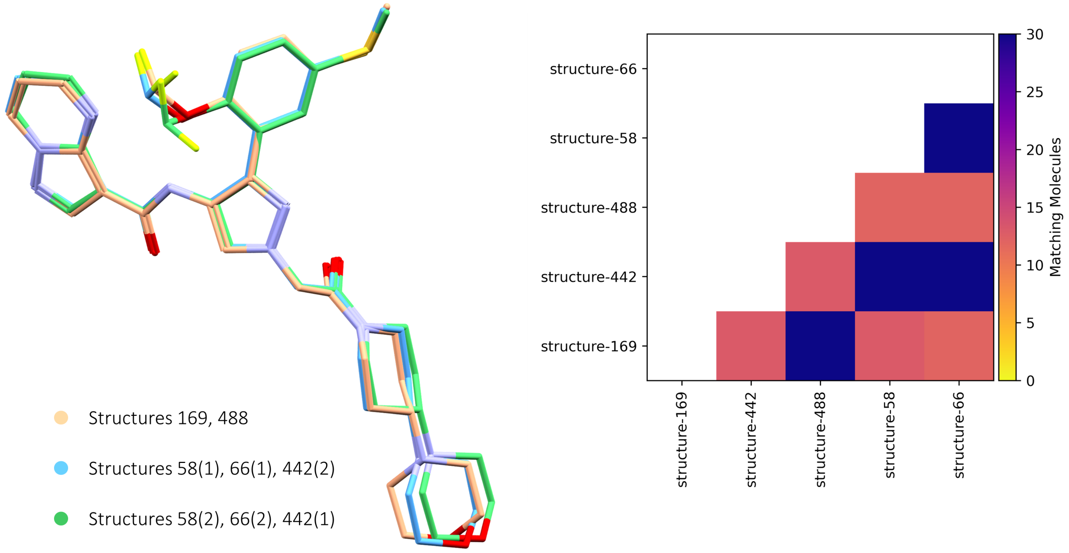

Fig. 6. (left) Main conformations of the molecules of structures 58, 66, 169, 442 and 488. Structures 58, 66 and 442 have $Z' = 2$ with the index of the two conformers specified in parenthesis. (right) Heatmap showing the number of molecules that match in a 30-molecule overlay with the Crystal Packing Similarity tool, using 25%/25° distance/angle tolerances. This shows a good match between structures 169 and 488 and structures 58, 66, and 442. Structures 181, 298, 321, 437 and 443 also have a good PXRD similarity with pattern N but are consistently ranked among the high-energy structures.

The main difference is in the conformation of both the difluoromethyl and the oxazine groups. As the energies of these theoretical structures are similar (Tab. 24), this could indicate that the crystals corresponding to pattern N have a similar packing with disorder of the difluoromethyl and the oxazine groups.

6. Previous Blind Tests Target Compounds

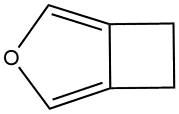

(I)

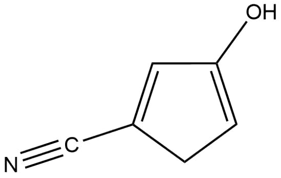

(II)

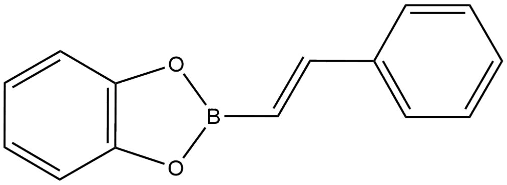

(III)

Fig. 7. Two-dimensional chemical structures of target compounds (I – III) from the first blind test of CSP methods.

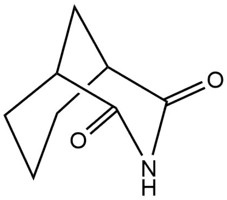

(IV)

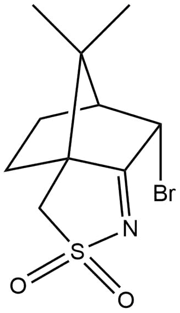

(V)

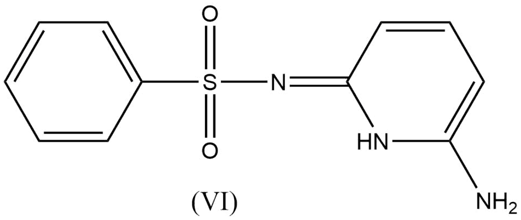

(VI)

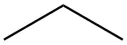

(VII)

Fig. 8. Two-dimensional chemical structures of target compounds (IV – VII) from the second blind test of CSP methods.

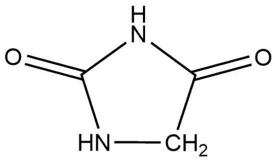

(VIII)

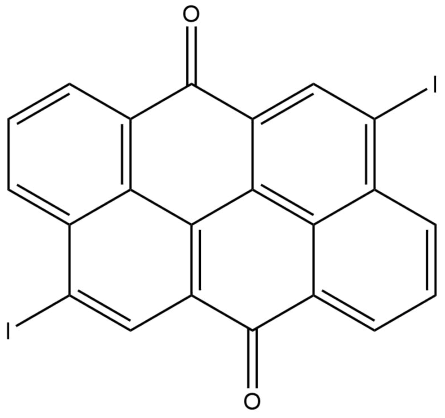

(IX)

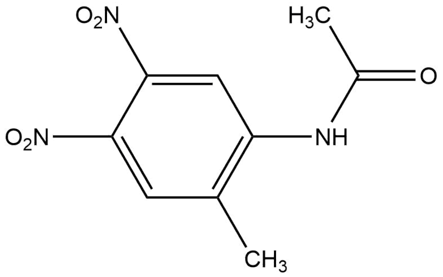

(X)

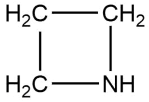

(XI)

Fig. 9. Two-dimensional chemical structures of target compounds (VIII – XI) from the third blind test of CSP methods.

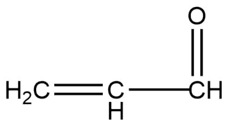

(XII)

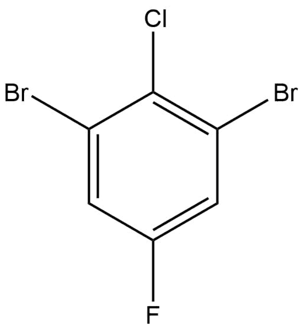

(XIII)

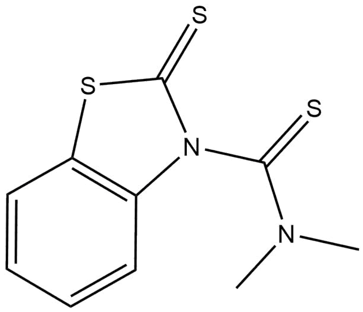

(XIV)

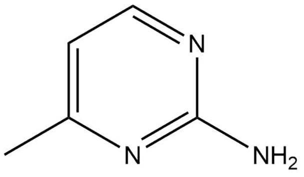

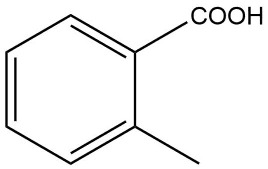

(XV)

Fig. 10. Two-dimensional chemical structures of target compounds (XII – XV) from the fourth blind test of CSP methods.

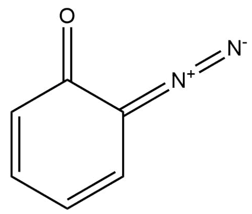

(XVI)

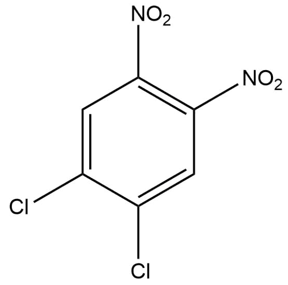

(XVII)

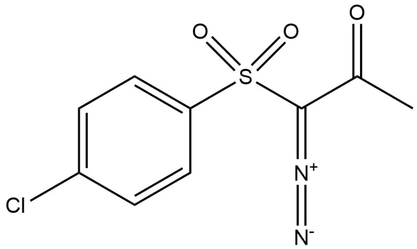

(XVIII)

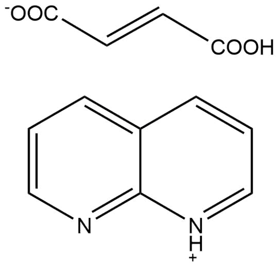

(XIX)

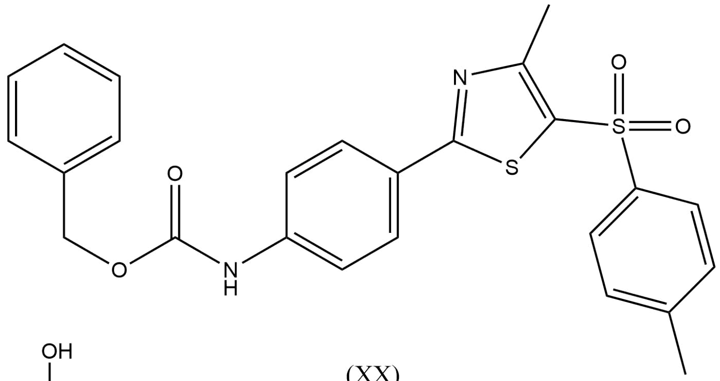

(XX)

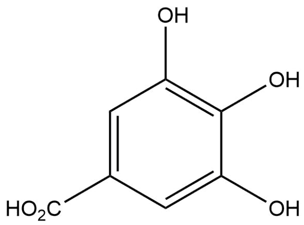

(XXI)

Fig. 11. Two-dimensional chemical structures of target compounds (XVI – XXI) from the fifth blind test of CSP methods.

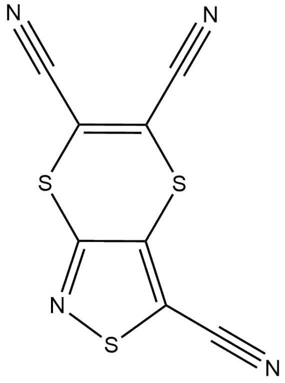

(XXII)

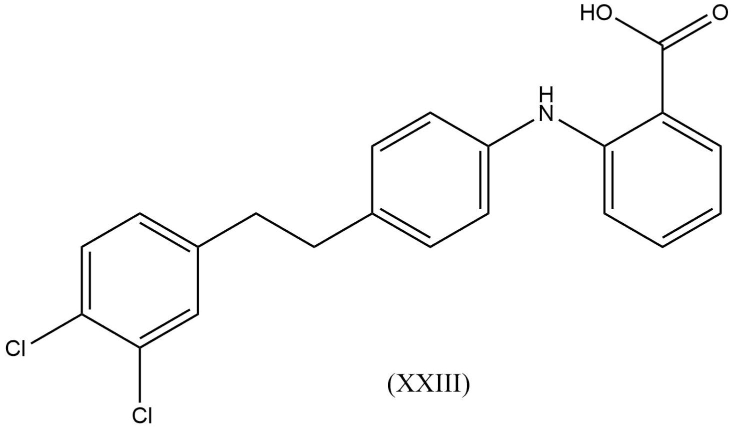

(XXIII)

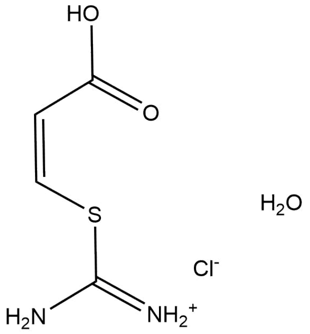

(XXIV)

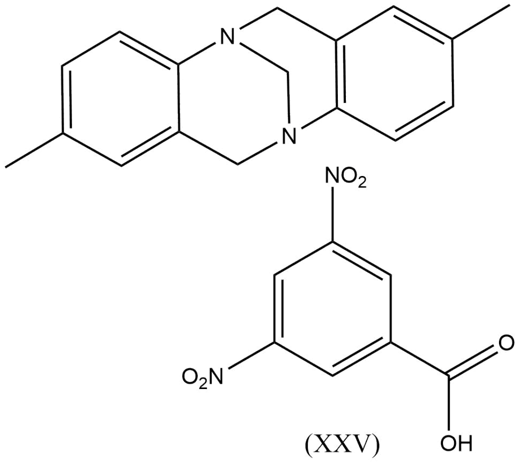

(XXV)

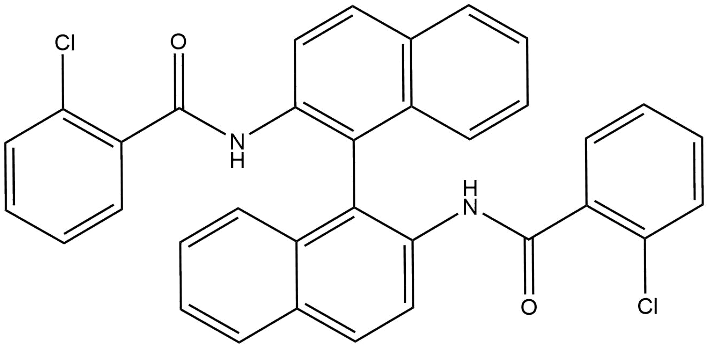

(XXVI)

Fig. 12. Two-dimensional chemical structures of target compounds (XXII – XXVI) from the sixth blind test of CSP methods.

7. DOI links for submitted sets of structures

Table 25. *DOI links for the submitted structure sets from each group for target XXVII.*

Group	DOI
3	https://doi.org/10.5517/ccdc.7bt.g3.xxvii.s2
5	https://doi.org/10.5517/ccdc.7bt.g5.xxvii.s2
6	https://doi.org/10.5517/ccdc.7bt.g6.xxvii.s2
9	https://doi.org/10.5517/ccdc.7bt.g9.xxvii.s2
10	https://doi.org/10.5517/ccdc.7bt.g10.xxvii.s2
11	https://doi.org/10.5517/ccdc.7bt.g11.xxvii.s2
12	https://doi.org/10.5517/ccdc.7bt.g12.xxvii.s2
15	https://doi.org/10.5517/ccdc.7bt.g15.xxvii.s2
16	https://doi.org/10.5517/ccdc.7bt.g16.xxvii.s2
17	https://doi.org/10.5517/ccdc.7bt.g17.xxvii.s2
20	https://doi.org/10.5517/ccdc.7bt.g20.xxvii.s2
21	https://doi.org/10.5517/ccdc.7bt.g21.xxvii.s2
22	https://doi.org/10.5517/ccdc.7bt.g22.xxvii.s2
24	https://doi.org/10.5517/ccdc.7bt.g24.xxvii.s2
27	https://doi.org/10.5517/ccdc.7bt.g27.xxvii.s2

Table 26. *DOI links for the submitted structure sets from each group for target XXVIII.*

Group	DOI
3	https://doi.org/10.5517/ccdc.7bt.g3.xxviii.s2
6	https://doi.org/10.5517/ccdc.7bt.g6.xxviii.s2
10	https://doi.org/10.5517/ccdc.7bt.g10.xxviii.s2
11	https://doi.org/10.5517/ccdc.7bt.g11.xxviii.s2
12	https://doi.org/10.5517/ccdc.7bt.g12.xxviii.s2
15	https://doi.org/10.5517/ccdc.7bt.g15.xxviii.s2
20	https://doi.org/10.5517/ccdc.7bt.g20.xxviii.s2
22	https://doi.org/10.5517/ccdc.7bt.g22.xxviii.s2
24	https://doi.org/10.5517/ccdc.7bt.g24.xxviii.s2
27	https://doi.org/10.5517/ccdc.7bt.g27.xxviii.s2

Table 27. *DOI links for the submitted structure sets from each group for target XXXI.*

Group	DOI
2	https://doi.org/10.5517/ccdc.7bt.g2.xxxi.s2
3	https://doi.org/10.5517/ccdc.7bt.g3.xxxi.s2
4	https://doi.org/10.5517/ccdc.7bt.g4.xxxi.s2
5	https://doi.org/10.5517/ccdc.7bt.g5.xxxi.s2
6	https://doi.org/10.5517/ccdc.7bt.g6.xxxi.s2
7	https://doi.org/10.5517/ccdc.7bt.g7.xxxi.s2
9	https://doi.org/10.5517/ccdc.7bt.g9.xxxi.s2
10	https://doi.org/10.5517/ccdc.7bt.g10.xxxi.s2
11	https://doi.org/10.5517/ccdc.7bt.g11.xxxi.s2
12	https://doi.org/10.5517/ccdc.7bt.g12.xxxi.s2
14	https://doi.org/10.5517/ccdc.7bt.g14.xxxi.s2
15	https://doi.org/10.5517/ccdc.7bt.g15.xxxi.s2
16	https://doi.org/10.5517/ccdc.7bt.g16.xxxi.s2
18	https://doi.org/10.5517/ccdc.7bt.g18.xxxi.s2
19	https://doi.org/10.5517/ccdc.7bt.g19.xxxi.s2
20	https://doi.org/10.5517/ccdc.7bt.g20.xxxi.s2
21	https://doi.org/10.5517/ccdc.7bt.g21.xxxi.s2
22	https://doi.org/10.5517/ccdc.7bt.g22.xxxi.s2
24	https://doi.org/10.5517/ccdc.7bt.g24.xxxi.s2
26	https://doi.org/10.5517/ccdc.7bt.g26.xxxi.s2

Table 28. *DOI links for the submitted structure sets from each group for target XXXII.*

Group	DOI
2	https://doi.org/10.5517/ccdc.7bt.g2.xxxii.s2
3	https://doi.org/10.5517/ccdc.7bt.g3.xxxii.s2
4	https://doi.org/10.5517/ccdc.7bt.g4.xxxii.s2
5	https://doi.org/10.5517/ccdc.7bt.g5.xxxii.s2
6	https://doi.org/10.5517/ccdc.7bt.g6.xxxii.s2
10	https://doi.org/10.5517/ccdc.7bt.g10.xxxii.s2
11	https://doi.org/10.5517/ccdc.7bt.g11.xxxii.s2
12	https://doi.org/10.5517/ccdc.7bt.g12.xxxii.s2
15	https://doi.org/10.5517/ccdc.7bt.g15.xxxii.s2
16	https://doi.org/10.5517/ccdc.7bt.g16.xxxii.s2
19	https://doi.org/10.5517/ccdc.7bt.g19.xxxii.s2
20	https://doi.org/10.5517/ccdc.7bt.g20.xxxii.s2
21	https://doi.org/10.5517/ccdc.7bt.g21.xxxii.s2
22	https://doi.org/10.5517/ccdc.7bt.g22.xxxii.s2
24	https://doi.org/10.5517/ccdc.7bt.g24.xxxii.s2
27	https://doi.org/10.5517/ccdc.7bt.g27.xxxii.s2

Table 29. *DOI links for the submitted structure sets from each group for target XXXIII.*

Group	DOI
3	https://doi.org/10.5517/ccdc.7bt.g3.xxxiii.s2
4	https://doi.org/10.5517/ccdc.7bt.g4.xxxiii.s2
5	https://doi.org/10.5517/ccdc.7bt.g5.xxxiii.s2
6	https://doi.org/10.5517/ccdc.7bt.g6.xxxiii.s2
9	https://doi.org/10.5517/ccdc.7bt.g9.xxxiii.s2
10	https://doi.org/10.5517/ccdc.7bt.g10.xxxiii.s2
11	https://doi.org/10.5517/ccdc.7bt.g11.xxxiii.s2
12	https://doi.org/10.5517/ccdc.7bt.g12.xxxiii.s2
15	https://doi.org/10.5517/ccdc.7bt.g15.xxxiii.s2
16	https://doi.org/10.5517/ccdc.7bt.g16.xxxiii.s2
18	https://doi.org/10.5517/ccdc.7bt.g18.xxxiii.s2
19	https://doi.org/10.5517/ccdc.7bt.g19.xxxiii.s2
20	https://doi.org/10.5517/ccdc.7bt.g20.xxxiii.s2
21	https://doi.org/10.5517/ccdc.7bt.g21.xxxiii.s2
22	https://doi.org/10.5517/ccdc.7bt.g22.xxxiii.s2
24	https://doi.org/10.5517/ccdc.7bt.g24.xxxiii.s2
27	https://doi.org/10.5517/ccdc.7bt.g27.xxxiii.s2
