# Supplementary material for: The seventh blind test of crystal structure prediction: structure ranking methods
Source: Acta Crystallogr B Struct Sci Cryst Eng Mater. 2024 Oct 17;80(Pt 6):548–74. doi: 10.1107/S2052520624008679 (PMC11789160; doi:10.1107/S2052520624008679)
Supplement: Supplementary file 2 [file b-80-00548-sup2.pdf]

Supplementary Information B (Methods SI per group). The Seventh Blind Test of Crystal Structure Prediction: Structure Ranking Methods

Lily M. Hunnisett, *et al.* *

The Cambridge Crystallographic Data Centre, 12 Union Road, Cambridge CB2 1EZ, UK. E-mail: lhunnisett@ccdc.cam.ac.uk

Contents

1 Group 2 **4**

2 Group 3 **10**

3 Group 4 **14**

4 Group 5 **19**

5 Group 6 **26**

6 Group 7 **30**

7 Group 9 **45**

8 Group 10 **47**

9 Group 11 **59**

10 Group 12 **62**

11 Group 14 **67**

12 Group 15 **75**

13 Group 16 **81**

14 Group 17 **103**

15 Group 18 **105**

16 Group 19 **108**

17 Group 20 **111**

18 Group 21 **121**

19 Group 22 **132**

20 Group 24 **135**

21 Groups 26 and 27 **145**

1. Group 2

Beran Group Computational Methods for CSP Blind Test 7, Phase 2

Gregory Beran†, Pablo A. Unzueta, and Cameron Cook

Department of Chemistry, University of California, Riverside, CA 92521. USA.

†gregory.beran@ucr.edu

We participated in the Phase 2 structure re-ranking for Molecules XXXI and XXXII.

Molecule XXXI

Geometry optimization: 99 of the 100 provided crystal structures were optimized using planewave density functional theory (DFT). Structure #89 (Z=18) was skipped due to time constraints and its large unit cell size. Unfortunately, this skipped structure corresponded to experimental form C, so we did not rank that form.

The DFT calculations were performed in Quantum Espresso[1,2] using the B86bPBE functional[3,4] and exchange-hole dipole moment (XDM) dispersion correction.[5] A planewave cutoff of 50 Ry and reciprocal space Monkhorst-Pack k-point grid densities of 0.06 Å^{-1} or better were employed. The projector augmented wave (PAW) approach was employed for the core electrons using potentials produced with Atomic v6.1.[1] Relatively tight geometry optimization convergence criteria of `etot_conv_thr` $= 2 \times 10^{-6}$ a.u. and `forc_conv_thr` $= 6 \times 10^{-4}$ a.u. were employed.

Final single-point energy calculations: To address any potential issues in the B86bPBE-XDM intramolecular conformational energies, a conformational energy correction[6] was applied to the DFT crystal energies:

$$E_{\text{crystal}}^{\text{corrected}} = E_{\text{crystal}}^{\text{DFT}} + \sum_{i}^{Z} \left(E_{\text{mon},i}^{\text{Higher}} - E_{\text{mon},i}^{\text{DFT}} \right) \tag{1}$$

This expression corrects the periodic DFT energy of the crystal using gas-phase calculations on each monomer in the unit cell, taking the difference between the DFT energy of the molecule and the energy computed with a higher level of theory. The correction is evaluated using the molecular geometries extracted directly from each crystal structure. Space group symmetry can be exploited by performing the gas-phase calculations only for the symmetrically-unique molecules (e.g. a Z'=1 structure has only one unique molecular geometry) and then multiplying those results by the number of symmetry-equivalent molecules in the unit cell. Such conformational energy corrections have proved important in previous systems due to DFT delocalization error.[6–11]

For molecule XXXI, domain local pair natural orbital coupled cluster singles and doubles with perturbative triples (DLPNO-CCSD(T0))[12] was used as the "Higher" level of theory. The DLPNO-CCSD(T0) calculations were performed in the aug-cc-pVTZ (aTZ) basis set in Orca v4.2 using TightPNO settings and TCutMKN = 10^{-4}. The results were then extrapolated to the complete basis set (CBS) limit by combining them with MP2/CBS energies according to the focal point method,

$$E_{\text{CBS}}^{\text{DLPNO-CCSD(T)}} = E_{\text{CBS}}^{\text{MP2}} + E_{\text{aTZ}}^{\text{DLPNO-CCSD(T)}} - E_{\text{aTZ}}^{\text{MP2}} \tag{2}$$

The MP2/CBS correlation energies were extrapolated from aug-cc-pVTZ and aug-cc-pVQZ (aQZ) calculations using the standard two-point expression[13] and were combined with Hartree-Fock energies in the aug-cc-pVQZ basis,

$$E_{\text{CBS}}^{\text{MP2}} = E_{\text{aQZ}}^{\text{HF}} + E_{\text{CBS}}^{\text{corr}} = E_{\text{aQZ}}^{\text{HF}} + \frac{4^3 E_{\text{aQZ}}^{\text{corr}} - 3^3 E_{\text{aTZ}}^{\text{corr}}}{4^3 - 3^3} \tag{3}$$

The MP2/CBS calculations were performed with PSI4.[14] Test calculations on a couple dozen of the molecule XXXI conformations employing the more robust iterative T1 triples approximation (DLPNO-CCSD(T1))[15] instead of T0 found differences of 0.1 kJ/mol or less in the relative conformational energies.

The gas-phase B86bPBE-XDM calculations in Eq 1 were again performed in Quantum Espresso with the same settings as the crystal calculations (and Γ-point only k-point sampling). Buffer spacing of at least 20 Å in all directions was employed to minimize interactions between periodic images of the gas-phase molecule. Specifically, the a, b, and c lattice parameters of the orthorhombic cell were determined by computing the "span" of the molecule along each Cartesian axis in Å and adding 20 Å (e.g. $a = x_{max} - x_{min} + 20$ Å).

Table 1 summarizes the final energies and rankings for the experimental Molecule XXXI structures. Notably, the conformational energy correction destabilizes the experimental structures. The DLPNO-CCSD(T0) conformational energies should be more accurate than the B86bPBE-XDM ones, though perhaps improving the intramolecular energies eliminates some error cancellation between the intra- and intermolecular contributions.[10]

Table 1: Summary of the relative energies (kJ/mol) and rankings with DFT before and after the DLPNO-CCSD(T0) conformational energy correction was applied. Structural similarities to the experimental crystal structures (the rmsd20 metric) are also provided.

	rmsd20	B86bPBE-XDM	B86bPBE-XDM +ΔDLPNO-CCSD(T0)
A_{maj}	0.10 Å	+0.0 (#1)	+2.2 (#7)
A_{min}	0.24 Å	+0.5 (#4)	+2.5 (#9)
B	0.17 Å	+3.7 (#16)	+4.8 (#17)
C	n/a	n/a	n/a

Molecule XXXII

A multi-step, hierarchical geometry refinement and energy ranking approach was employed for molecule XXXII.

1) Initial geometry optimization: All 500 crystal structures were first pre-optimized with HF-3c using Crystal17.[16] Since the provenance of the provided structures was unknown and different structures might have been optimized with different energy models (force field, DFT, etc), it was hoped that HF-3c would provide a more uniform starting point for subsequent refinement and ranking efforts.

2) Intermediate geometry optimization: 481 crystal structures were then optimized with periodic DFT. The same B86bPBE-XDM functional, PAW potentials, and planewave cutoff were used. However, looser energy and force convergence criteria were employed (`etot_conv_thr` $= 10^{-4}$ a.u. and `forc_conv_thr` $= 10^{-3}$ a.u) in the geometry optimization, along with slightly looser k-point grids. The remaining 19 structures were skipped due to time constraints; they all had larger unit cells and comparatively high HF-3c energies.

3) Intermediate energy ranking: To obtain intermediate-level energy rankings, a conformational energy correction (Eq 1) was applied to the periodic B86bPBE-XDM energies from Step 2. Given the larger molecular size of molecule XXXII compared to molecule XXXI, spin-component-scaled dispersion-corrected second-order Møller-Plesset perturbation theory (SCS-MP2D)[17] molecular calculations were used instead of DLPNO-CCSD(T0) ones. Benchmark tests for molecule XXXI found that the root-mean-square difference between SCS-MP2D and DLPNO-CCSD(T0) conformational energies was 0.3 kJ/mol; hopefully this good SCS-MP2D performances translates to Molecule XXXII as well. The SCS-MP2D calculations were performed using the MP2D library[18] and MP2 energies from PSI4.

4) Final geometry refinement and energy ranking: All 33 structures within ∼5 kJ/mol of the global minimum according to either DFT or the conformational energy-corrected DFT model were further optimized more tightly with B86bPBE-XDM. The SCS-

MP2D conformational energy correction was then applied to these refined structures to obtain the final DFT + ΔSCS-MP2D crystal energies. Table 2 summarizes the final energies and rankings for the experimental Molecule XXXII structures.

The 5 kJ/mol energy window used to select structures for final refinement was chosen due to time constraints. Unfortunately, the experimental structures lay outside this subset, with the most stable A_{maj} at +6.1 kJ/mol and B_{LT} at +6.5 kJ/mol in Step 3. Therefore, they were excluded from the final refinement. Post-hoc investigation after the conclusion of the Blind Test found that tighter DFT refinement of those structures would have lowered them to +4.7–4.8 kJ/mol above the GM.

Table 2: Summary of the relative energies (kJ/mol) and rankings with DFT before and after the SCS-MP2D conformational energy correction was applied. Structural similarities to the experimental crystal structures (the rmsd20 metric) are also provided.

	rmsd20	B86bPBE-XDM	B86bPBE-XDM +ΔSCS-MP2D
A_{maj}	0.30 Å	+6.4 (#38)	+6.1 (#26)
A_{min}	n/a	n/a	n/a
B_{LT}	0.23 Å	7.0 (#47)	6.5 (#29)
B_{RT}	0.40 Å	+28.2 (#441)	+28.8 (#430)

References

[1] P. Giannozzi, S. Baroni, N. Bonini, M. Calandra, R. Car, C. Cavazzoni, D. Ceresoli, G. L. Chiarotti, M. Cococcioni, I. Dabo, A. Dal Corso, S. de Gironcoli, S. Fabris, G. Fratesi, R. Gebauer, U. Gerstmann, C. Gougoussis, A. Kokalj, M. Lazzeri, L. Martin-Samos, N. Marzari, F. Mauri, R. Mazzarello, S. Paolini, A. Pasquarello, L. Paulatto, C. Sbraccia, S. Scandolo, G. Sclauzero, A. P. Seitsonen, A. Smogunov, P. Umari, and R. M. Wentzcovitch, J. Phys. Condens. Mat. **21**, 395502 (2009).

[2] P. Giannozzi, O. Andreussi, T. Brumme, O. Bunau, M. Buongiorno Nardelli, M. Calandra, R. Car, C. Cavazzoni, D. Ceresoli, M. Cococcioni, N. Colonna, I. Carnimeo, A. Dal Corso, S. de Gironcoli, P. Delugas, R. A. DiStasio, A. Ferretti, A. Floris, G. Fratesi, G. Fugallo, R. Gebauer, U. Gerstmann, F. Giustino, T. Gorni, J. Jia, M. Kawamura, H.-Y. Ko, A. Kokalj, E. Küçükbenli, M. Lazzeri, M. Marsili, N. Marzari, F. Mauri, N. L. Nguyen, H.-V. Nguyen, A. Otero-de-la Roza, L. Paulatto, S. Poncé, D. Rocca, R. Sabatini, B. Santra, M. Schlipf, A. P. Seitsonen, A. Smogunov, I. Timrov, T. Thonhauser, P. Umari, N. Vast, X. Wu, and S. Baroni, J. Phys. Condens. Mat. **29**, 465901 (2017).

[3] A. D. Becke, J. Chem. Phys. **85**, 7184 (1986).

[4] J. P. Perdew, K. Burke, and M. Ernzerhof, Phys. Rev. Lett. **77**, 3865 (1996).

[5] A. Otero-de-la Roza and E. R. Johnson, J. Chem. Phys. **136**, 174109 (2012).

[6] C. Greenwell and G. J. O. Beran, Cryst. Growth Des. **20**, 4875 (2020).

[7] G. J. O. Beran, CrystEngComm **21**, 758 (2019).

[8] C. Greenwell, J. L. McKinley, P. Zhang, Q. Zeng, G. Sun, B. Li, S. Wen, and G. J. O. Beran, Chem. Sci. **11**, 2200 (2020).

[9] G. J. O. Beran, I. J. Sugden, C. Greenwell, D. H. Bowskill, C. C. Pantelides, and C. S. Adjiman, Chem. Sci. **13**, 1288 (2022).

[10] G. J. O. Beran, S. E. Wright, C. Greenwell, and A. J. Cruz-Cabeza, J. Chem. Phys. **156**, 104112 (2022).

[11] B. Rana, G. J. O. Beran, and J. M. Herbert, Mol. Phys. e2138789 (2022).
[12] C. Riplinger, B. Sandhoefer, A. Hansen, and F. Neese, J. Chem. Phys. **139**, 134101 (2013).
[13] T. Helgaker, W. Klopper, H. Koch, and J. Noga, J. Chem. Phys. **106**, 9639 (1997).
[14] D. G. A. Smith, L. A. Burns, A. C. Simmonett, R. M. Parrish, M. C. Schieber, R. Galvelis, P. Kraus, H. Kruse, R. Di Remigio, A. Alenaizan, A. M. James, S. Lehtola, J. P. Misiewicz, M. Scheurer, R. A. Shaw, J. B. Schriber, Y. Xie, Z. L. Glick, D. A. Sirianni, J. S. O'Brien, J. M. Waldrop, A. Kumar, E. G. Hohenstein, B. P. Pritchard, B. R. Brooks, H. F. Schaefer, A. Y. Sokolov, K. Patkowski, A. E. DePrince, U. Bozkaya, R. A. King, F. A. Evangelista, J. M. Turney, T. D. Crawford, and C. D. Sherrill, J. Chem. Phys. **152**, 184108 (2020).
[15] Y. Guo, C. Riplinger, U. Becker, D. G. Liakos, Y. Minenkov, L. Cavallo, and F. Neese, J. Chem. Phys. **148**, 011101 (2018).
[16] R. Dovesi, A. Erba, R. Orlando, C. M. Zicovich-Wilson, B. Civalleri, L. Maschio, M. Rérat, S. Casassa, J. Baima, S. Salustro, and B. Kirtman, WIREs Comput. Mol. Sci. **8**, e1360 (2018).
[17] C. Greenwell, J. Řezáč, and G. J. O. Beran, Phys. Chem. Chem. Phys. **24**, 3695 (2022).
[18] The MP2D software can be downloaded at https://github.com/Chandemonium/MP2D.

2. Group 3

Supplementary Information of Group 3 (DBoese) for Phase Two

Alexander List[a], Nina Strasser[a], Johannes Hoja[a], Doris E. Braun[b], and A. Daniel Boese*[a]

[a] Department of Chemistry, University of Graz, Heinrichstrasse 28/IV, 8010 Graz, Austria
[b] Institute of Pharmacy, University of Innsbruck, Innrain 52c, 6020 Innsbruck, Austria

* Correspondence to: adrian_daniel.boese@uni-graz.at

1 Structure Ranking Procedure

All received crystal structures were first optimized with PBE+MBD[1–3] applying light species default settings using FHI-aims[4–9] (version 210716_2) together with ASE version 3.21.1[10]. As SCF convergence criteria we have used throughout 10^{-6} eV, 10^{-3} eV, 10^{-5} electrons/Å^3, and 10^{-4} eV/Å for the total energy, sum of eigenvalues, charge density, and forces, respectively. For target systems XXVII and XXVIII the scalar-relativistic ZORA (zero-order regular approximation) approach was used.

For these initial optimizations/lattice relaxations we have used some reduced convergence criteria in order to limit computation time. Hence, optimizations were carried out until all forces were less than 0.05 eV/Å and the k-grid was determined in such a way that for each direction $k \cdot l \geq 10$ Å for DFT and $k \cdot l \geq 15$ Å for MBD, where k is the number of k-points and l is the cell length in that direction.

Subsequently, all or a part of the resulting structures were further optimized using tighter convergence settings, i.e., the maximum value for the forces was set to 0.005 eV/Å and k-grids were now determined according to $k \cdot l \geq 18$ Å for DFT and $k \cdot l \geq 25$ Å for MBD. This k-grid definition was also used for all subsequent calculations. Next, PBE+MBD single point calculations were performed on obtained optimized structures utilizing tight species default settings within FHI-aims.

After this step, duplicate structures were removed on the basis of energy differences as well as by considering the overlay of the coordination shell of 20 molecules (RMSD20) within the crystal structure. Structures were assumed to be identical if their energies differed by less than 0.1 (XXXI, XXXII, XXXIII) or 0.5 (XXVII, XXVIII) kJ/mol and if their RMSD20 (distance tolerance: 0.2, angle tolerance: 20 degrees) was less than 0.1 Å.

The most stable structures according to the PBE+MBD/tight ranking were then further optimized by embedding multimers (up to dimers) at the PBE0+MBD/light[11] level into the PBE+MBD/light periodic calculation, labeled with ME2(PBE0+MBD:PBE+MBD)/light. The embedding calculations were performed with a dimer cutoff of 4 Å using the software MEmbed[12,13] together with FHI-aims. For the embedding calculations of target system XXXIII, one cation-anion pair was considered the monomer unit. All optimized structures were symmetrized and converted to CIFs using pymatgen[14]. The final lattice energies were obtained by calculating ME2(PBE0+MBD:PBE+MBD)/tight single points for the structures obtained from the previous step.

Finally, harmonic vibrational free energies (at 300 K) were calculated for the top structures utilizing phonopy[15]. Supercells with displacements of 0.005 Å were created so that the length in every direction exceeds 10 Å and the q-grid used for the evaluation of the vibrational free energy was set to satisfy $q \cdot l \geq$ 50 Å, where q is the number of q-points and l is the cell length in the respective direction. For systems

XXVII, XXVIII, and XXXII, these calculations were performed at the PBE+MBD/light level using the lowest-energy PBE+MBD/light-optimized geometries. For systems XXXI and XXXIII, they were performed at the ME2(PBE0+MBD:PBE+MBD)/light level at the lowest-energy ME2(PBE0+MBD:PBE +MBD)/light-optimized geometries.

The top structures were ranked based on their relative free energy (sum of lattice energy and vibrational free energy at 300 K). The remaining structures, for which no vibrational free energies were obtained, were then ranked based on their relative lattice energies of the best method they were calculated with.

The number of calculated structures at each calculation level is shown for all target systems in Table S1. The PBE+MBD/tight (level 3) energy range, for which subsequent embedding optimizations (level 4) were carried out amounts to 4.6 kJ/mol, 6 kJ/mol, 6 kJ/mol, 8 kJ/mol, and 11 kJ/mol for target systems XXVII, XXVIII, XXXI, XXXII, and XXXIII, respectively.

Table S1: Numbers of structures calculated at each step of the ranking procedure employed in phase two of the blind test.

Level	Method	XXVII	XXVIII	XXXI	XXXII	XXXIII
1	PBE+MBD/light opt. (red. conv. crit.)	100	500	100	500	500
2	PBE+MBD/light opt.	100	100	100	290	500
3	PBE+MBD/tight sp.	100	500	100	290	500
4	ME2(PBE0+MBD:PBE+MBD)/light opt.	11	9	34	25	15
5	ME2(PBE0+MBD:PBE+MBD)/tight sp.	11	9	34	25	15
6a	F_{vib}@PBE+MBD/light	16	9	-	35	-
6b	F_{vib}@ME2(PBE0+MBD:PBE+MBD)/light	-	-	34	-	15

2 Acknowledgments

The computational results presented have been achieved using the Vienna Scientific Cluster (VSC) as well as the HPC facilities at the University of Graz and the University of Innsbruck. This project has received funding from the European Union's Horizon 2020 research and innovation programme under the Marie Skłodowska-Curie grant agreement No 890300.

References

[1] J. P. Perdew, K. Burke and M. Ernzerhof, *Phys. Rev. Lett.*, 1996, **77**, 3865–3868.

[2] A. Tkatchenko, R. A. DiStasio, R. Car and M. Scheffler, *Phys. Rev. Lett.*, 2012, **108**, 236402.

[3] A. Ambrosetti, A. M. Reilly, R. A. DiStasio and A. Tkatchenko, *J. Chem. Phys.*, 2014, **140**, 18A508.

[4] V. Blum, R. Gehrke, F. Hanke, P. Havu, V. Havu, X. Ren, K. Reuter and M. Scheffler, *Comput. Phys. Commun.*, 2009, **180**, 2175–2196.

[5] F. Knuth, C. Carbogno, V. Atalla, V. Blum and M. Scheffler, *Comput. Phys. Commun.*, 2015, **190**, 33–50.

[6] V. W. zhe Yu, F. Corsetti, A. García, W. P. Huhn, M. Jacquelin, W. Jia, B. Lange, L. Lin, J. Lu, W. Mi, A. Seifitokaldani, Á. Vázquez-Mayagoitia, C. Yang, H. Yang and V. Blum, *Comput. Phys. Commun.*, 2018, **222**, 267–285.

[7] V. Havu, V. Blum, P. Havu and M. Scheffler, *J. Comput. Phys.*, 2009, **228**, 8367–8379.

[8] X. Ren, P. Rinke, V. Blum, J. Wieferink, A. Tkatchenko, A. Sanfilippo, K. Reuter and M. Scheffler, *New J. Phys.*, 2012, **14**, 053020.

[9] A. C. Ihrig, J. Wieferink, I. Y. Zhang, M. Ropo, X. Ren, P. Rinke, M. Scheffler and V. Blum, *New J. Phys.*, 2015, **17**, 093020.

[10] A. Hjorth Larsen, J. Jørgen Mortensen, J. Blomqvist, I. E. Castelli, R. Christensen, M. Dułak, J. Friis, M. N. Groves, B. Hammer, C. Hargus, E. D. Hermes, P. C. Jennings, P. Bjerre Jensen, J. Kermode, J. R. Kitchin, E. Leonhard Kolsbjerg, J. Kubal, K. Kaasbjerg, S. Lysgaard, J. Bergmann Maronsson, T. Maxson, T. Olsen, L. Pastewka, A. Peterson, C. Rostgaard, J. Schiøtz, O. Schütt, M. Strange, K. S. Thygesen, T. Vegge, L. Vilhelmsen, M. Walter, Z. Zeng and K. W. Jacobsen, *J. Phys. Condens. Matter*, 2017, **29**, 273002.

[11] C. Adamo and V. Barone, *J. Chem. Phys.*, 1999, **110**, 6158–6170.

[12] J. Hoja, A. List and A. D. Boese, *J. Chem. Theory Comput.*, 2024, **20**, 357–367.

[13] J. Hoja, A. List and A. D. Boese, *MEmbed: Multimer Embedding Code for the Simulation of Molecular Crystals*, 2022, `https://doi.org/10.5281/zenodo.7098559`.

[14] S. P. Ong, W. D. Richards, A. Jain, G. Hautier, M. Kocher, S. Cholia, D. Gunter, V. L. Chevrier, K. A. Persson and G. Ceder, *Comput. Mater. Sci.*, 2013, **68**, 314–319.

[15] A. Togo and I. Tanaka, *Scr. Mater.*, 2015, **108**, 1–5.

3. Group 4

Supporting information to submission from Group 4 for Stage 2 of the 7th CSP blind test

Petr Touš, Jan Ludík, Veronika Kostková, Ctirad Červinka*

Department of Physical Chemistry, University of Chemistry and Technology, Prague, Technická 5, Praha 6, Czechia

Corresponding author: ctirad.cervinka@vscht.cz

Computational methodology used in Stage 2 of the 7th CSP blind test

All candidate crystal structures for target systems XXXI, XXXII, and XXXIII were optimized using the PBE/PAW level of the density-functional theory[1] with the D3(BJ) dispersion correction,[2] as implemented in VASP, version 5.4.4.[3] Plane-wave kinetic energy cut-off 500 eV along with the standard PAW potentials were used within the PAW scheme.[4] The reciprocal space was sampled only at the Γ-point. Only the space group of symmetry was constrained in this initial optimization, which was considered converged after minimizing the residual variation of the total electronic energy per unit cell below 10^{-5} eV. Resulting electronic energies of the optimized structures were used for an initial polymorph ranking. Such a simplified setup of periodic DFT calculations for molecular crystals has been tested in our previous works.[5, 6] Corresponding polymorph landscapes are depicted in Figures 1-3 for individual targets.

Subsequently, all candidate structures were optimized using the third-order density functional tight binding method DFTB3, exploiting the 3ob-3-1 parametrization,[7, 8] as implemented in the DFTB+ code, version 21.1.[9] Again, only the Γ-point was sampled in electronic structure calculations. Convergence criterion of the geometry optimization was set to 10^{-4} Hartree/Bohr. Phonon calculations were performed using the finite-displacement method[10] within the harmonic approximation for each structure for a single unit-cell volume, corresponding to the DFTB3 optimized structure. The code Phonopy, version 2.12,[11] was used for all phonon manipulations. Note that the phonons were calculated including the D4 dispersion model[12] only for subsets of the most promising candidate polymorphs of targets XXXI and XXXIII, which were selected based on the PBE-D3(BJ)/PAW electronic energies.

Vibrational Helmholtz energies at ambient conditions were computed from the harmonic phonons. Together with the PBE-D3(BJ)/PAW electronic energies, these vibrational Helmholtz energies yielded the total finite-temperature Helmholtz energies, which were considered for our final polymorph ranking.[5] This thermodynamic approach is approximate in a way that it neglects the variation of both the electronic energy of the crystal and its phonons on volume,

which further propagates to placing an equality between isochoric and isobaric heat capacities of the crystal, as well as the Helmholtz and Gibbs energies at ambient pressure. These simplifications appreciably reduce the computational cost, but those are not expected to impart large errors to the final ranking of similar polymorphs.[13] Several structures could not be converged so that only real phonon frequencies would occur, excluding those from the free energy rankings. Corresponding polymorph landscapes in terms of the free energy are depicted in Figures 1-3 for individual targets.

Our ranking of target XXXI represents an optimistic computational scenario, as depicted in Figure 1. This molecule does not exhibit any significant chemical degrees of freedom that would be susceptible for miscalculations that can arise from typical flaws of DFT, such as the delocalization error inherent to all lower-rung DFT functionals. Most real polymorphs were identified near the lower edge of the polymorph landscape, especially when the free energy ranking is considered.

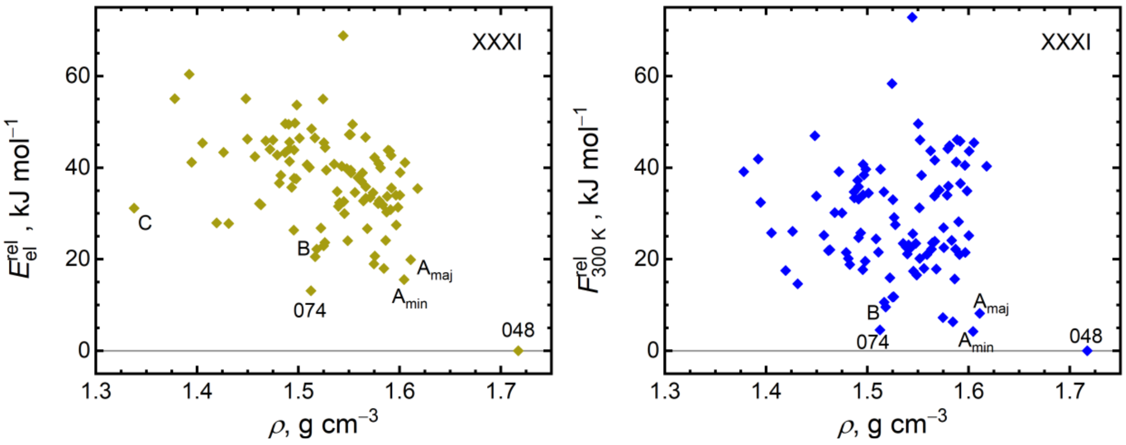

Figure 1. Ranking of candidate structures for target XXXI with the real polymorphs marked (A, B, C). Left – ranking due to electronic energy only; Right – ranking due to free energy at 300 K and 1 bar (C polymorph could not optimized to exhibit only real phonon frequencies).

Target molecule XXXII proved to prone to artificial delocalization of π-electron system over the nitrogen atom within its piperidine ring. This adverse effect of the overestimated sp^2 appeared strong enough to shift many structures with this particular planar nitrogen moiety far above in terms of relative energy, as depicted in Figure 2. The structures that appear as the most stable in our ranking exhibit sp^3 piperidine moiety. The real polymorphs A and B would be found near the lower edge of the central cluster of data points in our landscape. Also note that multiple XXXII structures could not optimized so that only real phonon frequencies would be observed. Some optimized structures could not be identified with any of the initial structures.

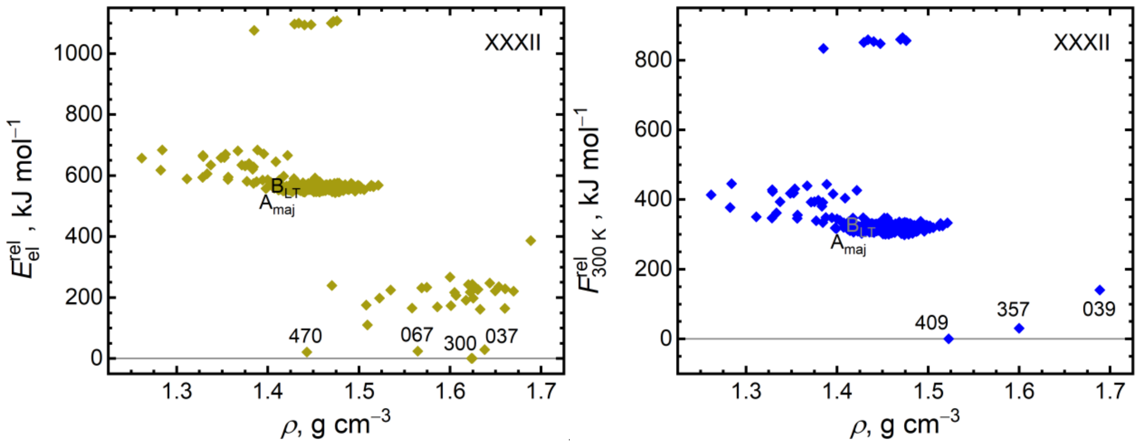

Figure 2. Ranking of candidate structures for target XXXII with the real polymorphs marked (A, B). Left – ranking due to electronic energy only; Right – ranking due to free energy at 300 K and 1 bar.

Target molecule XXXIII, being a protic two-component crystal, is by definition susceptible to proton transfer between the acid and basic component. Furthermore, there is a considerable conformational degree of freedom of the larger component, being able to act as anion. Its conformational changes strongly affect the extent of π-electron delocalization over its two ring moieties. The interplay of hydrogen bonding, optionally turning into proton transfer, and the π-electron delocalization will be clearly extremely vulnerable to any delocalization errors imparted by the nature of DFT. The cluster of data points, corresponding to the artificially destabilized structures, occurs arguably due to computational mistreatment of the proton positions along the hydrogen bonds. The energy off-set of the whole upper cluster corresponds to the typical proton transfer reaction energies that occur in zwitterionic molecular clusters.[13]

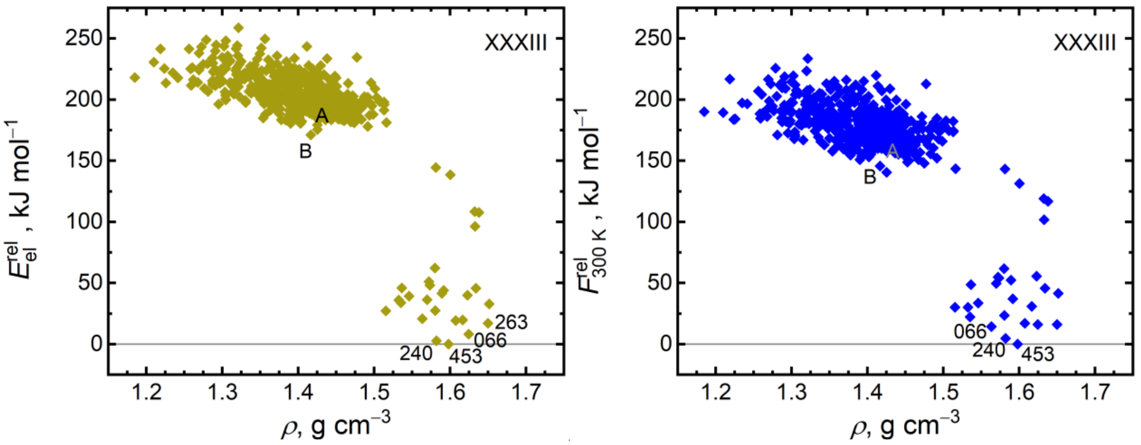

Figure 3. Ranking of candidate structures for target XXXIII with the real polymorphs marked (A, B). Left – ranking due to electronic energy only; Right – ranking due to free energy at 300 K and 1 bar.

References to this submission

1. J. P. Perdew, K. Burke and M. Ernzerhof, *Phys. Rev. Lett.*, 1996, **77**, 3865-3868.
2. S. Grimme, S. Ehrlich and L. Goerigk, *J. Comput. Chem.*, 2011, **32**, 1456-1465.
3. J. K. Hafner, G; Vogtenhuber, D.; Marsman, M., *Vienna Ab-initio Simulation Package*, 5.4.4 edn., 2017.
4. G. Kresse and D. Joubert, *Phys. Rev. B*, 1999, **59**, 1758-1775.
5. C. Červinka, M. Fulem, R. P. Stoffel and R. Dronskowski, *J. Phys. Chem. A*, 2016, **120**, 2022-2034.
6. C. Červinka, M. Klajmon and V. Štejfa, *Journal of Chemical Theory and Computation*, 2019, **15**, 5563-5578.
7. M. Gaus, A. Goez and M. Elstner, *Journal of Chemical Theory and Computation*, 2013, **9**, 338-354.
8. M. Gaus, X. Lu, M. Elstner and Q. Cui, *Journal of Chemical Theory and Computation*, 2014, **10**, 1518-1537.
9. B. Hourahine, B. Aradi, V. Blum, F. Bonafé, A. Buccheri, C. Camacho, C. Cevallos, M. Y. Deshaye, T. Dumitrică, A. Dominguez, S. Ehlert, M. Elstner, T. v. d. Heide, J. Hermann, S. Irle, J. J. Kranz, C. Köhler, T. Kowalczyk, T. Kubař, I. S. Lee, V. Lutsker, R. J. Maurer, S. K. Min, I. Mitchell, C. Negre, T. A. Niehaus, A. M. N. Niklasson, A. J. Page, A. Pecchia, G. Penazzi, M. P. Persson, J. Řezáč, C. G. Sánchez, M. Sternberg, M. Stöhr, F. Stuckenberg, A. Tkatchenko, V. W.-z. Yu and T. Frauenheim, *The Journal of Chemical Physics*, 2020, **152**, 124101.
10. K. Parlinski, Z. Q. Li and Y. Kawazoe, *Phys. Rev. Lett.*, 1997, **78**, 4063-4066.
11. A. Togo and I. Tanaka, *Scr. Mater.*, 2015, **108**, 1-5.
12. E. Caldeweyher, S. Ehlert, A. Hansen, H. Neugebauer, S. Spicher, C. Bannwarth and S. Grimme, *The Journal of Chemical Physics*, 2019, **150**, 154122.
13. C. Červinka and M. Fulem, *Phys. Chem. Chem. Phys.*, 2019, **21**, 18501-18515.

4. Group 5

Blind test methods

Graeme M. Day, Joseph E. Arnold, James Bramley, Patrick W. V. Butler, Ramon Cuadrado, Joseph Glover, Christopher R. Taylor

School of Chemistry, University of Southampton, Southampton, SO17 1BJ, United Kingdom

XXVII

Submission 1

Conformational sampling. Conformers were generated using low-mode sampling [1] with the Macromodel code and the OPLS-3 force field. These were re-optimised using Gaussian09 [2] with the PBE0 functional, GD3BJ dispersion correction and using 6-31G** basis for all atoms except iodine (I), which used the LANL2DZ basis with a core pseudopotential. Duplicate conformers were removed by comparison of all atom positions, with an RMSD limit of 0.3 Å.

Crystal structure generation: Crystal structures were generated and lattice energy minimised in the following space groups: 61, 14, 19, 2, 4, 15, 33, 9, 29, 5, 1, 60, 7, 18, 96, 76, 145, 43, 56, 13, 169, 88, 20, 86 and 148, all with Z`=1. Searches were performed for selected low energy conformers with Z`=2 in space groups : 1, 2, 33, 4, 14, 15, 19, 61 and 29.

Structure generation was performed using a quasi-random search, using the GLEE software [3].

Crystal structure optimisation: Two stages of optimisation were performed for each generated structure: rigid-molecule optimisation using the W99 force field [8] and atomic partial charges (fitted to the electrostatic potential generated from the full set of atomic multipoles); rigid-molecule optimisation using the W99 force field and atomic multipoles generated from a distributed multipole analysis of the DFT charge density. The iodine repulsion was in the force field was treated as anisotropic, based on parameters derived in the third blind test of crystal structure prediction.

Duplicate crystal structures were removed, first by clustering using comparison of simulated powder X-ray diffraction patterns, followed by clustering using the CSD API (with a 30-molecule cluster).

Structures for submission 1 were submitted based on their energy ranking after the second force field optimisation.

Submission 2

The set of structure provided for re-ranking were optimised using solid state DFT in the VASP [11] code. Calculations were performed with the PBE functional and a planewave basis set with pseudopotentials and GD3BJ dispersion correction. Optimisations were performed in three stages: fixed unit cell optimisation, followed by relaxed cell optimisation, both with a 500 eV planewave basis set cutoff. A final stage of optimisation was then performed with a 600 eV basis set cutoff and tighter convergence thresholds (PREC = Accurate, EDIFFG = -0.02). All calculations used a maximum electronic k-point spacing of 0.05 $Å^{-1}$.

XXVIII Calculations were stopped when it was revealed that the crystal structure has been reported.

XXIX

Conformational sampling. Conformers were generated using CREST [9] at the semi-empirical GFN2-xtb level of theory. These were re-optimised using Gaussian09 [2] with the PBE0 functional, GD3BJ dispersion correction and using 6-311G** basis set. Conformers were clustered manually, finding only two conformers.

Crystal structure generation: Crystal structures were generated and lattice energy minimised in the following space groups: 61, 14, 19, 2, 4, 15, 33, 9, 29, 5, 1, 60, 7, 18, 96, 76, 145, 43, 56, 13, 169, 88, 20, 86 and 148, all with Z`=1 using both conformers. Z`=2 searches were performed with all threecombinations of conformers in the following space groups: 1, 2, 33, 4, 14, 15, 19, 61 and 29. Further searchers were performed using distorted conformations generated by rotating torsion angles fromt eh gas phase minimised values.

Structure generation was performed using a quasi-random search, using the GLEE software [3]. All conformers were included in searching.

Crystal structure optimisation: Three stages of optimisation were performed for each generated structure: rigid-molecule optimisation using the FIT force field [5] and atomic partial charges (fitted to the electrostatic potential generated from the full set of atomic multipoles); rigid-molecule optimisation using the FIT force field and atomic multipoles generated from a distributed multipole analysis of the DFT charge density; fully-flexible tight binding DFT [7], using the DFTB 3ob parameter set ad D3 dispersion correction.

Duplicate crystal structures were removed, first by clustering using comparison of simulated powder X-ray diffraction patterns, followed by clustering using the CSD API (with a 30-molecule cluster). Clustering was performed prior to and after DFTB re-optimisation of structures.

Structures for submission 1 were submitted based on their energy ranking after DFTB. Best matches to the provided powder X-ray diffraction pattern were judged manually.

XXX

Conformational sampling . Conformers were generated using low-mode sampling [1] with the Macromodel code and the OPLS-3 force field. Separate searches were also run with CREST [9]. These lists were combined and unique conformers were re-optimised using Gaussian09 [2] with the PBE0 functional, GD3BJ dispersion correction and 6-311G(d,p) basis set. Duplicate conformers were removed by comparison of all atom positions, with an RMSD limit of 0.1 Å.

Crystal structure generation: Quasi-random structure generation [3] was performed with a series of rigid conformations in 2:1, 1:1 and 1:2 stoichiometries. For the larger component, 20 of the generated conformers were used, chosen as the 6 lowest energy conformers + 14 higher energy conformers with extended alkyl chains. To account for hydroxyl group flexibility, 8 OH orientations were sampled per conformer, so that a total of 160 starting molecular geometries were used. CSP in al stoichiometries was performed in space groups 1, 2, 19, 4, 61, 14 and 15.

Crystal structure optimisation: Four stages of optimisation were performed for each generated structure: rigid-molecule optimisation using the FIT force field [5] and atomic partial charges (fitted

to the electrostatic potential generated from the full set of atomic multipoles); rigid-molecule optimisation using the FIT force field and atomic multipoles generated from a distributed multipole analysis of the DFT charge density; fully-flexible tight binding DFT [7], using the DFTB 3ob parameter set ad D3 dispersion correction. Final energies were evaluated by reoptimizing all unique structures after DFTB with the FIT force field + multipoles, and taking the intramolecular energy from a DFT (PBE0/6-311G(d,p)) single-point energy.

Structures were ranked by their energy with respect to the stoichiometric sum of calculated energies for the pure component crystal structures. The pure component structures were obtained from the CSD and optimised with the same procedure as CSP structures.

XXXI

Submission 1

Conformational sampling. Conformers were generated using low-mode sampling [1] with the Macromodel code and the OPLS-3 force field. These were re-optimised using Gaussian09 [2] with the PBE0 functional, GD3BJ dispersion correction and 6-311G(d,p) basis set. Duplicate conformers were removed by comparison of all atom positions, with an RMSD limit of 0.1 Å. This resulted in 10 distinct conformers in the gas phase.

Crystal structure generation: Crystal structures were generated and lattice energy minimised in the following space groups: 61, 14, 19, 2, 4, 15, 33, 9, 29, 5, 1, 60, 7, 18, 96, 76, 145, 43, 56, 13, 169, 88, 20, 86 and 148, all with Z`=1. Z`=2 searches were performed with all combinations of conformers in the following space groups: 1, 2, 33, 4, 14, 15, 19, 61 and 29.

Structure generation was performed using a quasi-random search, using the GLEE software [3]. All conformers were included in searching. In addition, CSP was performed (Z`=1 only) using a set of conformations generated by distorting the gas phase conformers. 125 distorted conformations were generated by distorting the three flexible torsions by +/- 72 degrees and +/- 144 degrees from their phase geometries.

Crystal structure optimisation: Three stages of optimisation were performed for each generated structure: rigid-molecule optimisation using the FIT force field [5] and atomic partial charges (fitted to the electrostatic potential generated from the full set of atomic multipoles); rigid-molecule optimisation using the FIT force field and atomic multipoles generated from a distributed multipole analysis of the DFT charge density; fully-flexible tight binding DFT [7], using the DFTB 3ob parameter set ad D3 dispersion correction.

Duplicate crystal structures were removed, first by clustering using comparison of simulated powder X-ray diffraction patterns, followed by clustering using the CSD API (with a 30-molecule cluster). Clustering was performed prior to and after DFTB re-optimisation of structures.

Structures for submission 1 were submitted based on their energy ranking after DFTB.

Submission 2

The set of structure provided for re-ranking were optimised using solid state DFT in the VASP [11] code. Calculations were performed with the PBE functional and a planewave basis set with pseudopotentials and GD3BJ dispersion correction. Optimisations were performed in

three stages: fixed unit cell optimisation, followed by relaxed cell optimisation, both with a 500 eV planewave basis set cutoff. A final stage of optimisation was then performed with a 600 eV basis set cutoff and tighter convergence thresholds (PREC = Accurate, EDIFFG = -0.02). All calculations used a maximum electronic k-point spacing of 0.05 $Å^{-1}$.

XXXII

Submission 1

Conformational sampling. Conformers were generated using low-mode sampling [1] with the Macromodel code and the OPLS-3 force field. These were re-optimised using Gaussian09 [2] with the PBE0 functional, GD3BJ dispersion correction and 6-31G(d,p) basis set. Duplicate conformers were removed by comparison of all atom positions, with an RMSD limit of 0.1 Å. This resulted in 4089 distinct conformers in the gas phase.

Crystal structure generation: Crystal structures were generated and lattice energy minimised in the following space groups: 61, 14, 19, 2, 4, 15, 33, 9, 29, 5, 1, 60, 7, 18, 96, 76, 145, 43, 56, 13, 169, 88, 20, 86 and 148, all with Z`=1. Structure generation was performed using a quasi-random search, using the GLEE software [3]. All conformers up to 32.5 kJ/mol above the lowest energy gas phase conformer were included in searching. In addition higher energy conformers were also included, where the molecular energy + a molecular surface area bias term were within 22.5 kJ/mol of the global energy minimum conformer. The bias term was calculated as lowering the energy by 0.49 kJ/mol per $Å^2$ of solvent accessible surface area. This was based on ref [6]. A total of 528 conformers were included in CSP.

Crystal structure optimisation: Three stages of optimisation were performed for each generated structure: rigid-molecule optimisation using the FIT force field and atomic partial charges (fitted to the electrostatic potential generated from the full set of atomic multipoles); rigid-molecule optimisation using the FIT force field and atomic multipoles generated from a distributed multipole analysis of the DFT charge density; fully-flexible tight binding DFT, using the DFTB 3ob parameter set ad D3 dispersion correction.

Duplicate crystal structures were removed, first by clustering using comparison of simulated powder X-ray diffraction patterns, followed by clustering using the CSD API (with a 30-molecule cluster). Clustering was performed prior to and after DFTB re-optimisation of structures.

Structures for submission 1 were submitted based on their energy ranking after DFTB.

Submission 2

The set of structure provided for re-ranking were optimised using solid state DFT in the VASP [11] code. Calculations were performed with the PBE functional and a planewave basis set with pseudopotentials and GD3BJ dispersion correction. Optimisations were performed in three stages: fixed unit cell optimisation, followed by relaxed cell optimisation, both with a 500 eV planewave basis set cutoff. A final stage of optimisation was then performed with a 600 eV basis set cutoff and tighter convergence thresholds (PREC = Accurate, EDIFFG = -0.02). All calculations used a maximum electronic k-point spacing of 0.05 $Å^{-1}$.

XXXIII

Submission 1

Conformational sampling. Conformers were generated using low-mode sampling [1] with the Macromodel code and the OPLS-3 force field. These were re-optimised using Gaussian09 [2] with the PBE0 functional, GD3BJ dispersion correction and 6-311G++(d,p) basis set. Duplicate conformers were removed by comparison of all atom positions, with an RMSD limit of 0.1 Å. This resulted in 10 distinct conformers of the anion in the gas phase and 3 distinct conformers of the cation.

Crystal structure generation: Crystal structures were generated and lattice energy minimised in the following space groups: 2, 14, 4, 19, 1, 29, 33, 15 and 61, all with Z`=1. Structure generation was performed using a basin hopping search, using the GLEE software [10]. Searches were performed using all combinations of cation and anion conformers. Additional searchers were performed with the starting anion conformation distorted from the gas phase optimised conformers in 72 degree increments around the three flexible bonds, and performing CSP with all distorted anion conformations within 25 kJ/mol of the lowest energy conformer.

Crystal structure optimisation: Three stages of optimisation were performed for each generated structure: rigid-molecule optimisation using the FIT force field and atomic partial charges (fitted to the electrostatic potential generated from the full set of atomic multipoles); rigid-molecule optimisation using the FIT force field and atomic multipoles generated from a distributed multipole analysis of the DFT charge density; fully-flexible tight binding DFT, using the DFTB 3ob parameter set ad D3 dispersion correction.

Duplicate crystal structures were removed, first by clustering using comparison of simulated powder X-ray diffraction patterns, followed by clustering using the CSD API (with a 30-molecule cluster). Clustering was performed prior to and after DFTB re-optimisation of structures.

DFTB was found to transfer the proton in some crystal structures, resulting in a co-crystal, rather than a salt. Therefore, structures for submission 1 were submitted based on a combination of DFTB structures and structures from the force field stage (FIT + multipoles). Structures from both stages were ranked by relative energy, relative to the global minimum at that stage of calculations. We then took the 1500 structures with lowest relative energies from both sets combined, removing any structures that were duplicated between the two sets. Due to the combination of methods, the relative energies within this set of structures are not reliable.

Submission 2

The set of structure provided for re-ranking were optimised using solid state DFT in the VASP [11] code. Calculations were performed with the PBE functional and a planewave basis set with pseudopotentials and GD3BJ dispersion correction. Optimisations were performed in three stages: fixed unit cell optimisation, followed by relaxed cell optimisation, both with a 500 eV planewave basis set cutoff. A final stage of optimisation was then performed with a 600 eV basis set cutoff and tighter convergence thresholds (PREC = Accurate, EDIFFG = -0.02). All calculations used a maximum electronic k-point spacing of 0.05 $Å^{-1}$.

[1] I. Kolossváry and W. C. Guida , *Biopolymers*, 1996, 7863 , 5011 —5019

[2] Gaussian 09, Revision D.01, M. J. Frisch, G. W. Trucks, H. B. Schlegel, G. E. Scuseria, M. A. Robb, J. R. Cheeseman, G. Scalmani, V. Barone, B. Mennucci, G. A. Petersson, H. Nakatsuji, M. Caricato, X. Li, H. P. Hratchian, A. F. Izmaylov, J. Bloino, G. Zheng, J. L. Sonnenberg, M. Hada, M. Ehara, K. Toyota, R. Fukuda, J. Hasegawa, M. Ishida, T. Nakajima, Y. Honda, O. Kitao, H. Nakai, T. Vreven, J. A. Montgomery, Jr., J. E. Peralta, F. Ogliaro, M. Bearpark, J. J. Heyd, E. Brothers, K. N. Kudin, V. N. Staroverov, T. Keith, R. Kobayashi, J. Normand, K. Raghavachari, A. Rendell, J. C. Burant, S. S. Iyengar, J. Tomasi, M. Cossi, N. Rega, J. M. Millam, M. Klene, J. E. Knox, J. B. Cross, V. Bakken, C. Adamo, J. Jaramillo, R. Gomperts, R. E. Stratmann, O. Yazyev, A. J. Austin, R. Cammi, C. Pomelli, J. W. Ochterski, R. L. Martin, K. Morokuma, V. G. Zakrzewski, G. A. Voth, P. Salvador, J. J. Dannenberg, S. Dapprich, A. D. Daniels, O. Farkas, J. B. Foresman, J. V. Ortiz, J. Cioslowski, and D. J. Fox, Gaussian, Inc., Wallingford CT, 2013.

[3] David H. Case, Josh E. Campbell, Peter J. Bygrave, and Graeme M. Day, Journal of Chemical Theory and Computation 2016 12 (2), 910-924

[4] Sarah L. Price, Maurice Leslie, Gareth W. A. Welch, Matthew Habgood,Louise S. Price,Panagiotis G. Karamertzanis and Graeme M. Day, Phys. Chem. Chem. Phys., 2010,12, 8478-8490

[5] D. S. Coombes , S. L. Price , D. J. Willock and M. Leslie , *J. Phys. Chem.*, 1996, **100** , 7352 —7360

[6] H. P. G. Thompson and G. M. Day, *Chem. Sci.*, 2014,5, 3173-3182

[7] B. Hourahine, B. Aradi, V. Blum, F. Bonafé, A. Buccheri, C. Camacho, C. Cevallos, M. Y. Deshaye, T. Dumitrică, A. Dominguez, S. Ehlert, M. Elstner, T. van der Heide, J. Hermann, S. Irle, J. J. Kranz, C. Köhler, T. Kowalczyk, T. Kubař, I. S. Lee, V. Lutsker, R. J. Maurer, S. K. Min, I. Mitchell, C. Negre, T. A. Niehaus, A. M. N. Niklasson, A. J. Page, A. Pecchia, G. Penazzi, M. P. Persson, J. Řezáč, C. G. Sánchez, M. Sternberg, M. Stöhr, F. Stuckenberg, A. Tkatchenko, V. W.-z. Yu, and T. Frauenheim, "DFTB+, a software package for efficient approximate density functional theory based atomistic simulations", J. Chem. Phys. 152, 124101 (2020) https://doi.org/10.1063/1.5143190

[8] D. E. Williams *J. Comput. Chem.*, 2001, **22** , 1154 —1166

[9] P. Pracht, F. Bohle and S. Grimme, *Phys. Chem. Chem. Phys.*, 2020,22, 7169-7192

[10] S. Yang and G. M. Day, *J. Chem. Theory Comput*. 2021, 17, 3, 1988–1999

[11] G. Kresse and J. Hafner, Phys. Rev. B, 1993, 47, 558; G. Kresse and J. Hafner, Phys. Rev. B, 1994, 49, 14251; G. Kresse and J. Furthmuller, Comput. Mat. Sci., 1996, 6, 15; G. Kresse and J. Furthmuller, Phys. Rev. B, 1996, 54, 11169; G. Kresse and D. Joubert, Phys. Rev., 1999, 59, 1758

5. Group 6

CSP methodology phase2, van Eijck (UPACK)

This document describes the methodology used to prepare the results submitted in phase 2, structure ranking. It is an extract from the full Supplementary Material.

1 Force fields

For each compound an individual force field was constructed. The intramolecular parameters for bond distances and bond angles were taken from comparable structures in de CSD database. The potentials for the dihedral angles were modeled by Fourier terms. Torsional angles involving sp^2 atoms were restrained to planarity with aid of a large twofold term. Methyl groups had a threefold barrier of 0.8 kJ/mol for each combination of end atoms. The other dihedral angles can be essential to determine the conformation, and their choice is often critical for successful modeling. Their barrier was taken as zero unless discussed otherwise below for each target compound separately.

Point charges on the atoms were taken from ESP fittings, using MOLDEN [1] on 6-31G** wave functions calculated by the program GAMESS-UK [2]. For nonbonded interactions the Price-Williams ("PW") force field, with Buckingham repulsion [3-5], was used. If computationally feasible, a final minimization was done where the intramolecular energy was calculated ab-initio (the "PW-AI" method). Here GAMESS-UK [2] was used again, with charges recalculated for each individual structure. A detailed discussion of this procedure and its merits has been given in the Supplementary Material of the sixth blind test [6]. The details of the PW potential can be found in Table 2 of the Supplementary Material for the present test.

The UPACK program [7,8] was used for all energy calculations. Specific force field detauls are described below.

Compound XXVII. The Si–C≡C–C linkages were assumed to be linear with free rotation. To avoid excessive computer effort the two iodine atoms were replaced by chlorine atoms in the AI calculations. Previous experience (compound XIII) has shown that the charges for the halogens are usually not so different.

Compound XXVIII. This is a curious structure, with NH groups linked to a $CuCl_2$ group. Considering more or less comparable structures in the CSD, the C–C=N···Cu group was kept planar. Free rotation was allowed about Cu···N and also for the four phenyl groups. The angles N···Cu···N and Cl–Cu–Cl were set at 155°with the bending force constant reduced by a factor of 10. The stretching force constants for atoms linked to Cu were likewise reduced.

The odd number of electrons around copper prevented ab-initio calculations. Standard force field values were taken for the atomic charges. No effort was made to consult the experimental data that were published during the blind test.

Compound XXXI. No potential was set for the three conformation-determining dihedrals or for the five-membered ring. The latter was found to deviate from planarity.

Compound XXXII. A large molecule with at least 10 internal degrees of freedom. Some dihedrals appear to prefer planarity, but the CSD information is so scanty that it is hardly useful to enumerate the choice of all torsional potentials that were set.

Compound XXXIII. The anion is identical to the one in COKROJ, so details of the geometry could be taken over. No potential was set for the three conformation-determining dihedrals.

2 Submission 2: Structural Ranking

The hypothetical structures received from the CCDC were recalculated with the PW force field, in order to find out quickly whether problems arose. This was indeed the case for about 50 structures, since UPACK can only treat triclinic, monoclinic and orthorhombic structures. The only quick solution was to expand these structures, eliminating symmetry operations not available in the program. In this way two structures in compound XXXIII could not be treated as the number of artificially independent molecules became too large to handle. A few other structures gave diverging energy optimization for unknown reasons. The final numbers of structures studied ($N1$) are given in the table below.

The CCDC results are guaranteed to contain the experimental structure(s), so a comparison with the UPACK-generated structures is interesting. The numbers of CCDC structures in the low-energy range of 15 kJ/mol ($N2$) are given in the table. The net line shows the number of these ($N3$) that occur in the present structure generation submission (CCDC structures obviously taken from that submission excluded). For XXVIII the overlap is excellent, except that just the CCDC structure with lowest energy was not found. At the other extreme, there was no overlap for XXXII where the structure generation was already judged to be hopelessly incomplete.

For the final ranking in submission 2 the energies were recalculated with the PW-AI method. (except XVIII). As explained above, for compound XXVII iodine was replaced by chlorine in the AI calculations. The computing times are also given in the table.

Results for the ranking challenge.

	XXVII	XXVIII	XXXI	XXXII	XXXIII
*N*1 (finally ranked)	99	495	100	499	497
*N*2 (low CCDC structures)	32	29	45	15	36
*N*3 (common structures]	11	25	12	0	23
CPU time (hours)	455	10	55	1200	195

*N*1 is the number of structures included in this challenge.
*N*2 is the number of structures in a PW energy range of 15 kJ/mol.
*N*3 is the number of these that were present in submission 1.
CPU is the computing time (hours) standardized for one 2.66 GHz Intel Quad 9400 processor at Utrecht University.

3 References

[1] G. Schaftenaar and J. H. Noordik, J. Comput.-Aided Mol. Design, **14**, 123-134 (2000).
[2] M. F. Guest et al., Mol. Phys **103**, 719-747 (2005).
[3] D. E. Williams and D. J. Houpt, Acta Cryst. **B42**, 286-295 (1986)
[4] D. S. Coombes, S. L. Price, D. J. Willock, and M. Leslie, J. Phys. Chem. **100**, 7352-7360 (1996).
[5] T. Beyer and S. L. Price, J. Phys. Chem. **B104**, 2647-2655 (2000).
[6] A. M. Reilly *et al.*, Acta Cryst. **B72**, 439-459 (2016)
[7] B. P. van Eijck and J. Kroon, Acta Cryst. **B56** 535-542 (2000).
[8] B. P. van Eijck, http://www.crystal.chem.uu.nl/∼vaneyck/upack.html

4 Acknowledgements and correspondence

Toine Schreurs and Martin Lutz provided computer facilities and assistance.
The author is retired from Utrecht University, The Netherlands.
Correspondence address: b.p.vaneijck@chem.uu.nl or vaneijck@xs4all.nl

6. Group 7

Supplementary Information on Target XXXI
Second Submission

Galanakis, Nikolaos[1*] & Tuckerman, Mark E.[1,2,3*]

[*] Correspondence to: ng1807@nyu.edu, mark.tuckerman@nyu.edu

[1] Department of Chemistry, New York University,
New York, NY 10003 USA.

[2] Courant Institute of Mathematical Sciences,
New York University, New York, NY 10012 USA.

[3] NYU-ECNU Center for Computational Chemistry at NYU Shanghai,
3663 Zhongshan Road North, Shanghai, 200062, China.

Monday, June 6th, 2022

Contents

1 Introduction **3**

2 Topological measures of crystal stability **4**

3 Ranking scheme **8**

3.1 Ranking scheme refinement 10

1 Introduction

Target XXXI ($C_{12}H_{12}NO_3F_3S$) is a closed shell system compound of agrochemical interest, with a total of 32 atoms. The compound has 2 rotational degrees of freedom, along the bonds that connect the sulfide atom with the two neighbouring carbon atoms. For this target, the Cambridge Crystallographic Data Centre (CCDC) set up two different phases for Crystal Structure Prediction (CSP). In phase 1, participating teams where required to submit a landscape of up to 1500 structures. In phase 2, CCDC provided the participants with a list of 100 structures which contained all the known polymorphs of Target XXXI. The participants where asked to rank the structures in this list in terms of energy stability.

2 Topological measures of crystal stability

In the proposed topological scheme for CSP and structure ranking, the governing mathematical principles where derived by performing a statistical analysis on the organic molecular crystals in the Cambridge Structural Database (CSD) [1] and the Crystallography Open Database (COD) [2, 3]. The stability of molecular crystals can be determined by (1) the orientation of the molecule in terms of the principal axes of inertia, (2) the orientation of the rings in terms of the normal ring plane vector, (3) the positions of highly electropositive/electronegative atoms, (4) the separations of atomic pairs consisting of at least one highly electropositive/electronegative atom, and (5) the close contacts formed by overlapping molecules.

The analysis of the 2868 structures in the CSD database composed of C, H, N, O, F, S atoms (CHNOFS structures) revealed a strong connection between the orientation of the principal axes of inertia and the normal ring plane vectors to the cell geometry. For each structure, the eigenvectors $\hat{e}_i$, $i = 1, 2, 3$ of the inertia tensor as well as the normal vector $\hat{k}_c$ representing the average ring plane are almost perpendicular to the crystallographic directions $\mathbf{n} = (n_u, n_v, n_w)$ with components restricted to $0, \pm1, \ldots, \pm n_{\max}$, $n_u|n_v|n_w = n_{\max}$ and $n_u n_v n_w = 0$. Figures 1-2 show respectively the distributions of the minimum angle $\omega_{\min}$ formed by the set of vectors $\mathbf{n}$ and the inertia tensor eigenvectors/normal ring plane vectors, for $n_{\max} = 1, 2, 3, 4, 5$. In each figure, the 95% confidence interval is noted. Additional lower confidence intervals were also calculated to examine the proximity of each proposed structure to the peaks observed in the database. The likelihood of a structure is determined by the number of angles $\omega_{\min}$ that are found within the confidence intervals and the average deviation of the angles $\omega_{\min}$ from the 90°.

Highly electropositive/electronegative atoms have a strong tendency to be found at positions corresponding to the zeroes of a set of geometric functions Ξ_k. For a unit cell containing Z molecules, each having M atoms, consider the crystallographic coordinates $\mathbf{r}_1^{(i)}$ of atom (i) in the reference

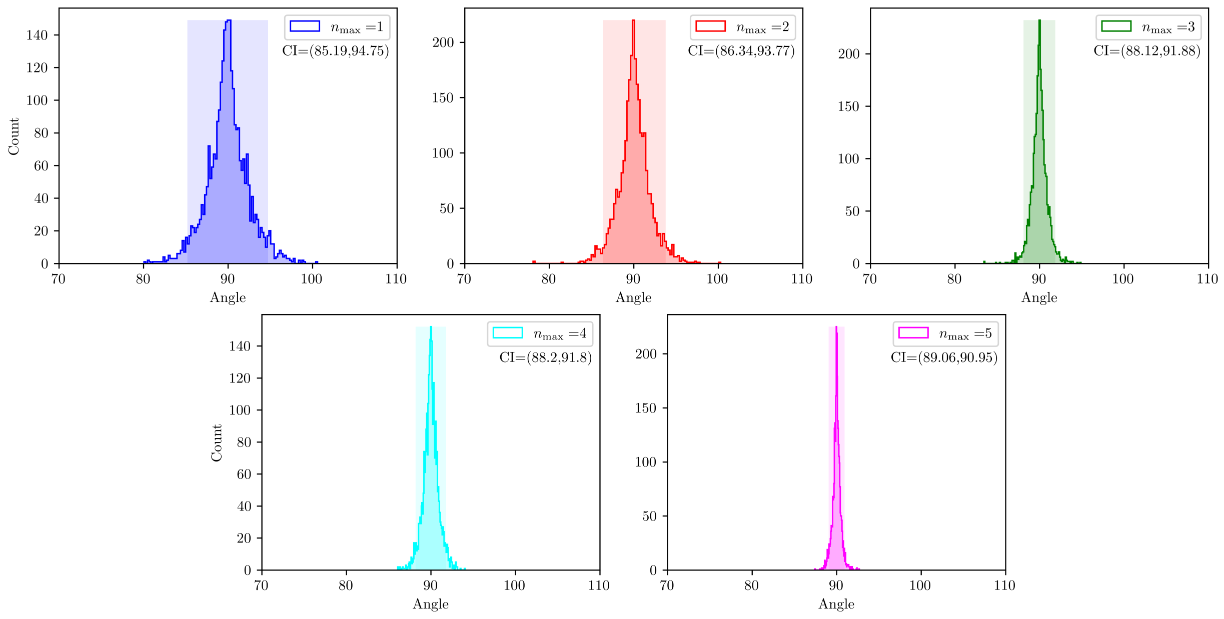

Figure 1: Distributions of the minimum angle formed by the principal axes of inertia to the crystallographic directions $\mathbf{n} = (n_u, n_v, n_w)$ with components restricted to $0, \pm1, \ldots, \pm n_{\max}$, $n_u|n_v|n_w = n_{\max}$ and $n_u n_v n_w = 0$ for all the CHFNOS structures in the CSD database.

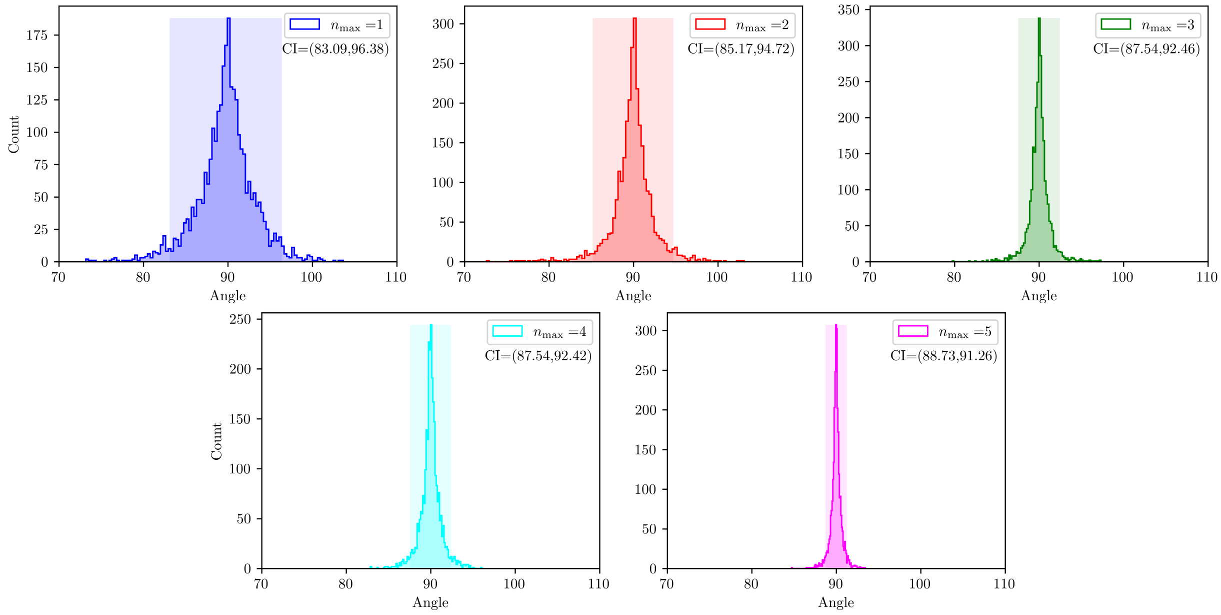

Figure 2: Distributions of the minimum angle formed by the normal ring plane vectors to the crystallographic directions $\mathbf{n} = (n_u, n_v, n_w)$ with components restricted to $0, \pm 1, \ldots, \pm n_{\max}$, $n_u|n_v|n_w = n_{\max}$ and $n_u n_v n_w = 0$ for all the CHFNOS structures in the CSD database.

molecule. Let $\mathbf{r}_m^{(i)} = \mathbf{G}_j \mathbf{r}_m^{(i)}$, $m = 2, \ldots, M$ be its $Z-1$ symmetry-related atomic positions in the unit cell, where $\mathbf{G}_j$, $j = 1, ..., Z$ is a set of space-group generators for the crystal. The aforementioned positions can be calculated by solving the set of equations

$$\Xi_k(\mathbf{G}_1\mathbf{r}_1^{(i)} - \mathbf{r}_p^{(i)}, ..., \mathbf{G}_Z\mathbf{r}_1^{(i)} - \mathbf{r}_p^{(i)}) = 0 \quad (1)$$

where $\mathbf{r}_p^{(i)}$ is the average of the Z positions $\mathbf{r}_m^{(i)}$. The choice of the functions Ξ_k is very important and although various choices might be suitable, we have selected the so-called Zernike parameters $Z_{n\ell m}$ defined as

$$Z_{n\ell m}(\mathbf{r}_1^{(i)}, ..., \mathbf{r}_Z^{(i)}) = \left[\frac{3n+1}{4\pi Z} \sum_{j=1}^{Z} R_{n\ell}(r_j(i)) Y_{\ell,m}(\theta_j(i), \phi_j(i)) \right]^2 \quad (2)$$

where

$$R_{n\ell}(r) = \sum_{k=0}^{\frac{n-\ell}{2}} \frac{(-1)^k (n-k)!}{k! \left(\frac{n+\ell}{2} - k\right)! \left(\frac{n-\ell}{2} - k\right)} r^{n-2k} \quad (3)$$

are the Zernike polynomials. The solutions of these equations are crystallographic planes in the general form

$$\epsilon_z : A_{\text{zzp}} u + B_{\text{zzp}} v + C_{\text{zzp}} w = 0.25 k_{\text{zzp}}, \quad (4)$$

where $A_{\text{zzp}}, B_{\text{zzp}}, C_{\text{zzp}} \in [-1, 0, 1]$, $A_{\text{zzp}} \times B_{\text{zzp}} \times C_{\text{zzp}} = 0$ and $k_{\text{zzp}} \in [0, \pm 1, \pm 2, \pm 3, \pm 4]$. The possible combinations of $A_{\text{zzp}}, B_{\text{zzp}}, C_{\text{zzp}}$ generate 9 possible crystallographic planes that are parallel to the faces of the unit cell (i.e. $u = 0.25 k_{\text{zzp}}$) or parallel to the 2D diagonals (i.e. $u + w = 0.25 k_{\text{zzp}}$). In addition, for more than 98% of the CHNOFS structures in the CSD, there is at least one atomic

pair along each direction ϵ_z, formed by an atom with high partial charge and another atom of the molecule, so that the distance of the two atoms along the direction ϵ_z is equal to $0.25k_{\mathrm{zzp}}$. In Fig. 3 we show the distribution of the charges for each possible pair that are used to determine the likelihood of each structure in terms of the intramolecular crystallographic atomic separations.

The strength of the close contact depends on the atomic species forming the contact and the space group of the structure. In general, the strength d_s varies in the range $[0, d_s^{\max})$. In Fig. 4-5 we show the distributions of the vdW short contact strength for all the possible atomic pairs of the analyzed structures. It is clear that the strength of the close contact also depends on the existence of a hydrogen bond or not. We separate the close contacts into three categories: (1) vdW short contacts in which case none of the atoms is part of a hydrogen bond, (2) Mixed, formed by one atom that is part of a H-Bond and one atom that is not part of a H-Bond and (3) H-Bonds, in which the short contact is formed by two atoms directly related to a hydrogen bond. Other close contacts such as halogen or chalcogen bonds are possible but are not considered here. In the case of the target XXXI structures, all the close contacts are of vdW type. These probability allow us to determine the probability for each target XXXI candidate structure to form the set of close contacts that are specific to the structure.

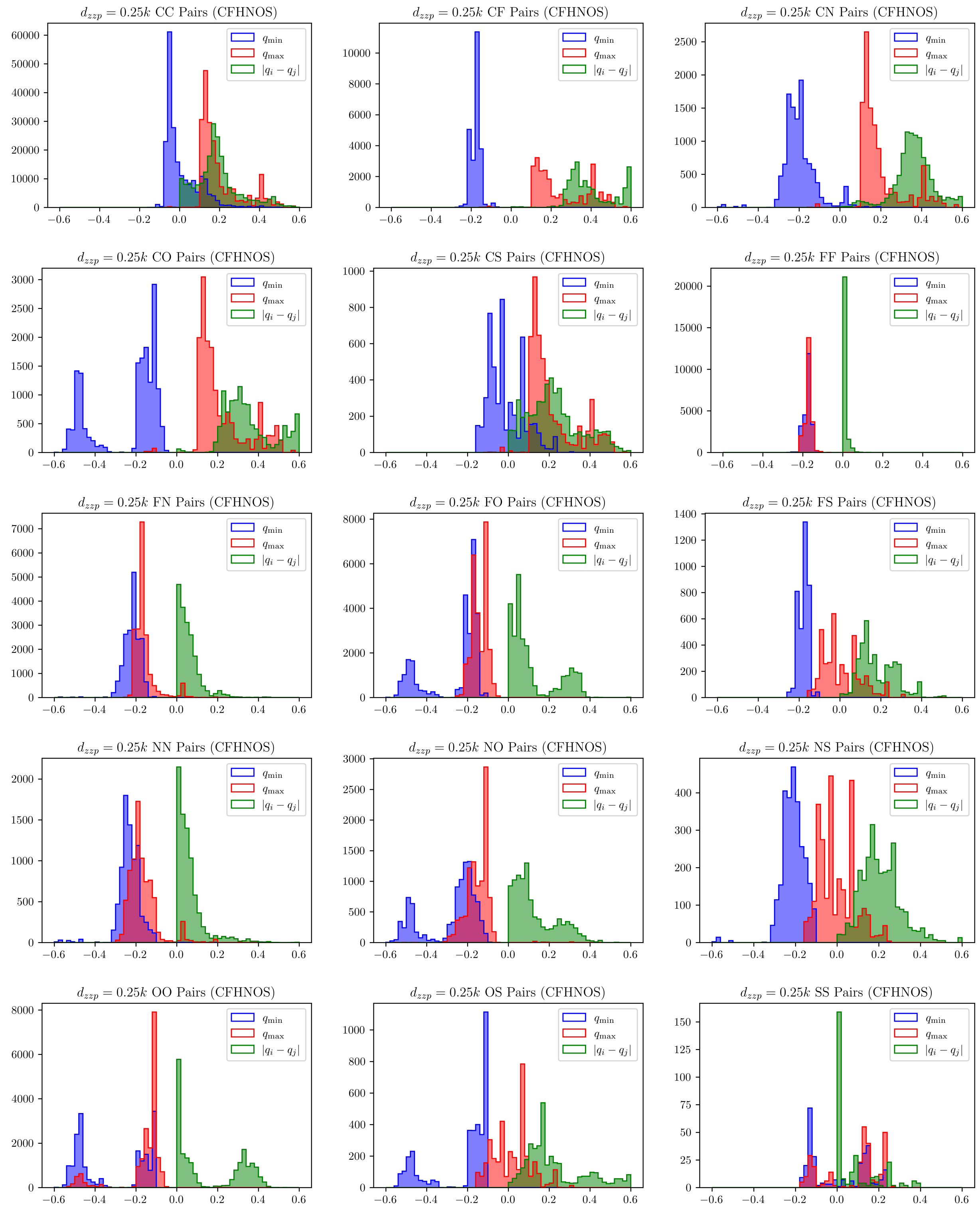

Figure 3: Distribution of the partial charges of the atoms forming pairs at distance $0.25k$ in crystallographic coordinates along the 9 possible ZZP directions for all the structures in the CSD database composed of C, H, F, N, O and S atoms.

3 Ranking scheme

For each structure in the list of 100 structures provided by CSD, the inertial eigenvectors, the normal ring plane vectors, the positions of the highly electropositive/electronegative atoms, the separations of atoms in the reference molecule and the close contacts were calculated. The partial charges for the Target XXXI compound were assigned via the CSD Python API using the Gasteiger method [4]. The process identified 10 highly electropositive/electronegative atoms ($|q| < 0.10$) in the structure, labeled as C_2, C_5, C_6, C_{12}, O_1, O_2, O_3, F_1, F_2 and F_3. The ranking process involves 7 steps:

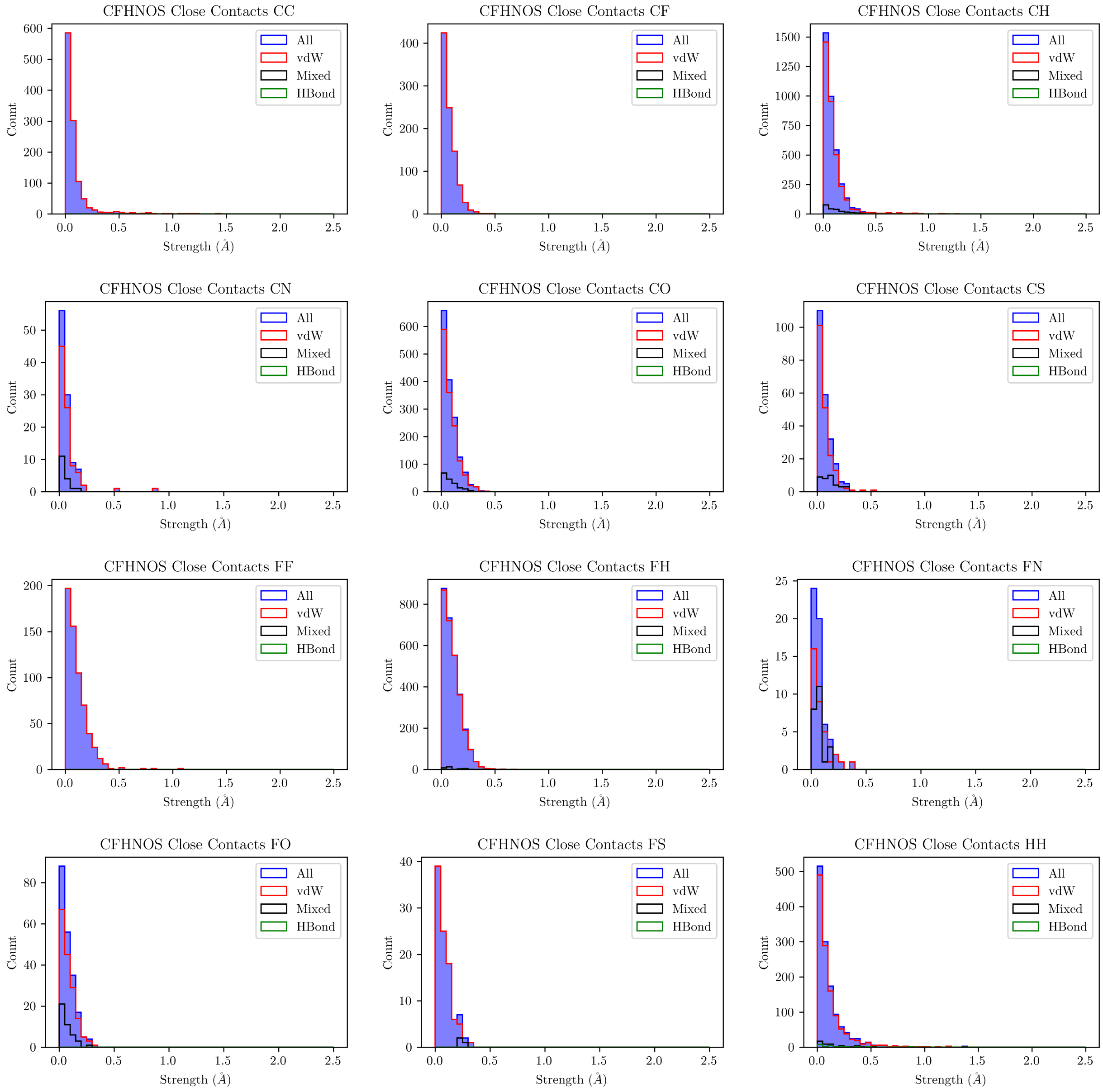

Figure 4: Distribution of the short contact strength (Part 1) for all the structures in the CSD database composed of C, H, F, N, O and S atoms and for the different short contact types.

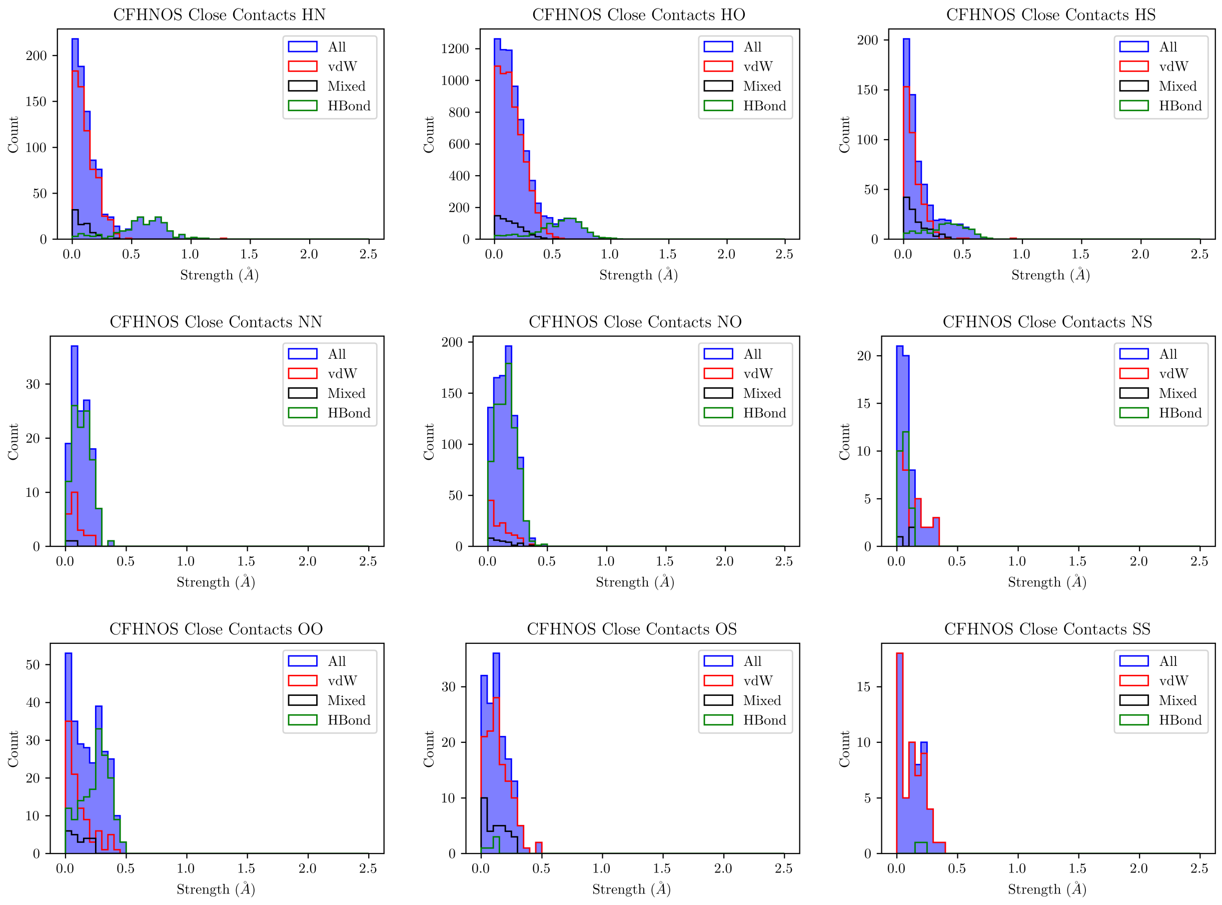

Figure 5: Distribution of the short contact strength (Part 2) for all the structures in the CSD database composed of C, H, F, N, O and S atoms and for the different short contact types.

1. Discard structures for which $n_{\mathrm{X},0.01} = 0$, where $n_{\mathrm{X},0.01}$ is the number of highly electropositive/electronegative atoms of species X$\in \{\mathrm{C}, \mathrm{O}, \mathrm{F}\}$ that are found at distance $d \leq 0.01$ (in crystallographic coordinates) to the nearest plane ϵ_z.

2. Discard structures for which $n_{\mathrm{X},0.025} < n_{\mathrm{X}}$, where $n_{\mathrm{X},0.025}$ is the number of highly electropositive/electronegative atoms that are found at distance $d \leq 0.025$ (in crystallographic coordinates) to the nearest plane ϵ_z and n_{X} is the total number of highly electropositive/electronegative atoms for species X.

3. Discard structures for which more than one of the angles ω_{min} formed by the eigenvectors of the inertia tensor $\hat{e}_i$ and the crystallographic directions $\mathbf{n}$ lies outside the 95% confidence intervals.

4. Discard structures for which more than one of the angles ω_{min} formed by the normal vectors $\hat{k}_c$ of the rings and the crystallographic directions $\mathbf{n}$ lies outside the 95% confidence intervals.

5. Discarding structures for which more than 50% of the angles ω_{min} formed by the eigenvectors of the inertia tensor $\hat{e}_i$ and the crystallographic directions $\mathbf{n}$ lies outside the 50% confidence intervals.

6. Discard structures for which more than 50% of the angles $\omega_{\min}$ formed by the normal vectors $\hat{k}_c$ of the rings and the crystallographic directions $\mathbf{n}$ lies outside the 50% confidence intervals.

7. Rank the structure by calculating the combined probability:

$$P = 0.25(P_{cc} + P_{d_{\text{zzp}}} + P_{\hat{e}_i} + P_{\hat{k}_c}), \tag{5}$$

where

- P_{cc} is the probability of a structure to form the specific set of close contacts.
- P_{cc} is the probability of a structure to have the specific set of atomic pairs at distance $0.25k_{\text{zzp}}$.
- $P_{\hat{e}_i}$ is the probability of a structure to have the angles $\omega_{\min}$ formed by the eigenvectors of the inertia tensor $\hat{e}_i$ and the crystallographic directions $\mathbf{n}$ within the confidence intervals.
- $P_{\hat{k}_c}$ is the probability of a structure to have the angles $\omega_{\min}$ formed by the normal vectors $\hat{k}_c$ of the rings and the crystallographic directions $\mathbf{n}$ within the confidence intervals.

In table 1 we list the 25 most probable structures according to the aforementioned criteria.

3.1 Ranking scheme refinement

At the time of submission, only parts of the topological information described in section 2 was available and as a result, the performance of the above ranking scheme was moderate. Recent developments of the method include the implementation of cost functions that measure with improved accuracy the proximity of a random structure to the topological measurements derived from the analysis of the CSD database. The stability of a structure in terms of the orientation of the principal inertia axes in assessed through the cost functions

$$C_{i,ortho} = \sum_{\substack{i,j=1,2,3 \\ j>i}} \frac{(\mathbf{u}_i \times \mathbf{v}_i) \cdot (\mathbf{u}_j \times \mathbf{v}_j)}{|\mathbf{u}_i \times \mathbf{v}_i||\mathbf{u}_j \times \mathbf{v}_j|} \quad \text{and} \quad C_{i,perp} = \sum_{i=1,2,3} \hat{e}_i \cdot \left(\frac{\mathbf{H}\mathbf{u}_i}{|\mathbf{H}\mathbf{u}_i|} + \frac{\mathbf{H}\mathbf{v}_i}{|\mathbf{H}\mathbf{v}_i|} \right). \tag{6}$$

where $\mathbf{u}_i, \mathbf{v}_i \in \mathbf{n}$ are the two crystallographic directions in the set $\mathbf{b}$ that minimize the quantity

$$\text{dev}_{90} = \frac{1}{2} \left(\widehat{\mathbf{u}_{i,c}, \hat{k}_r} + \widehat{\mathbf{v}_{i,c}, \hat{k}_r} \right) \tag{7}$$

describing the average deviation from 90° of the angles formed by the vectors $\mathbf{u}_i, \mathbf{v}_i$ to the inertia eigenvectors $\hat{e}_i$ and

$$\mathbf{H} = \mathbf{T}^{-1} = \begin{pmatrix} a & b\cos\gamma & c\cos\beta \\ 0 & b\sin\gamma & c\frac{\cos\alpha - \cos\beta\cos\gamma}{\sin\gamma} \\ 0 & 0 & \frac{\Omega}{ab\sin\gamma} \end{pmatrix} \tag{8}$$

in which $\Omega = abc\sqrt{1 - \cos^2\alpha - \cos^2\beta - \cos^2\gamma + 2\cos\alpha\cos\beta\cos\gamma}$ is the unit cell volume.

To assess the atomic separations for specific pairs involving at least one highly electropositive/electronegative atom, we introduce the cost function

$$C_{\text{zzp}} = \sum_{\boldsymbol{\epsilon}_z} \frac{\boldsymbol{\epsilon}_z^T}{|\boldsymbol{\epsilon}_z|} \begin{pmatrix} u_i - u_j \\ v_i - v_j \\ w_i - w_j \end{pmatrix} \tag{9}$$

Table 1: List of the 39 structures ranked that passed though all the topological filters in order of P.

**Structure**	**Space group**	P	P_{cc}	$P_{d_{\text{zzp}}}$	$P_{\hat{e}_i}$	$P_{\hat{k}_c}$
XXXI_structure_24	$P2_1/c$	0.6042	0.1045	0.3124	1.0000	1.0000
XXXI_structure_5	$P2_1/c$	0.6017	0.0949	0.3120	1.0000	1.0000
XXXI_structure_29	$P2_1/c$	0.5993	0.1018	0.2953	1.0000	1.0000
XXXI_structure_38	$P2_1/c$	0.5981	0.1022	0.2901	1.0000	1.0000
XXXI_structure_59	$P2_1/c$	0.5978	0.1010	0.2901	1.0000	1.0000
XXXI_structure_79	$P2_1/c$	0.5960	0.0823	0.3018	1.0000	1.0000
XXXI_structure_19	$P2_1/c$	0.5954	0.0810	0.3006	1.0000	1.0000
XXXI_structure_56	$P2_1/c$	0.5952	0.0904	0.2903	1.0000	1.0000
XXXI_structure_31	$P2_1/c$	0.5938	0.0943	0.2808	1.0000	1.0000
XXXI_structure_70	$P2_1/c$	0.5936	0.0756	0.2990	1.0000	1.0000
XXXI_structure_67	$P2_1/c$	0.5936	0.0764	0.2980	1.0000	1.0000
XXXI_structure_35	$P2_12_12_1$	0.5934	0.1128	0.2607	1.0000	1.0000
XXXI_structure_42	$P-1$	0.5932	0.1378	0.2350	1.0000	1.0000
XXXI_structure_86	$P2_1/c$	0.5925	0.1005	0.2694	1.0000	1.0000
XXXI_structure_96	$P2_1/c$	0.5910	0.0725	0.2915	1.0000	1.0000
XXXI_structure_33	$P2_1/c$	0.5908	0.0711	0.2922	1.0000	1.0000
XXXI_structure_45	$P2_1/c$	0.5895	0.0776	0.2803	1.0000	1.0000
XXXI_structure_7	$C2/c$	0.5886	0.0820	0.2726	1.0000	1.0000
XXXI_structure_97	$P2_1/n$	0.5866	0.1053	0.3077	0.9333	1.0000
XXXI_structure_60	$P2_1/c$	0.5862	0.0725	0.2721	1.0000	1.0000
XXXI_structure_84	$P2_12_12_1$	0.5859	0.0955	0.2482	1.0000	1.0000
XXXI_structure_58	$C2/c$	0.5857	0.0834	0.2596	1.0000	1.0000
XXXI_structure_57	$P2_1/c$	0.5855	0.0893	0.3194	0.9333	1.0000
XXXI_structure_46	$P2_1$	0.5837	0.0810	0.2538	1.0000	1.0000
XXXI_structure_22	$P2_1/c$	0.5828	0.0790	0.3187	0.9333	1.0000
XXXI_structure_25	$P2_1/c$	0.5813	0.0898	0.3020	0.9333	1.0000
XXXI_structure_47	$P-1$	0.5811	0.0848	0.2394	1.0000	1.0000
XXXI_structure_61	$P-1$	0.5802	0.0979	0.2230	1.0000	1.0000
XXXI_structure_12	$P-1$	0.5773	0.1011	0.2083	1.0000	1.0000
XXXI_structure_3	$P-1$	0.5765	0.0906	0.2155	1.0000	1.0000
XXXI_structure_100	$P2_1/c$	0.5764	0.1235	0.2820	1.0000	0.9000
XXXI_structure_66	$P2_1/c$	0.5751	0.0751	0.2919	0.9333	1.0000
XXXI_structure_63	$P2_1/c$	0.5736	0.0855	0.3089	1.0000	0.9000
XXXI_structure_4	$P2_1$	0.5646	0.0771	0.2480	0.9333	1.0000
XXXI_structure_68	$Pbca$	0.5616	0.0929	0.2535	1.0000	0.9000
XXXI_structure_1	$P2_1/c$	0.5611	0.0742	0.2704	1.0000	0.9000
XXXI_structure_23	$P-1$	0.5558	0.0824	0.2073	0.9333	1.0000
XXXI_structure_54	$P-1$	0.5396	0.0866	0.2073	0.9333	0.9000

where $\boldsymbol{\epsilon}_z \in \{(1,0,0),(0,1,0),(0,0,1),(1,\pm1,0),(1,0,\pm1),(0,1,\pm1)\}$. The definition of highly electropositive/electronegative charges was also updated for the different atomic species forming the pair, based on the distributions in presented in Fig. 3. Under this definition, we have three different groups of charges:

- Highly electropositive/electronegative atoms O_1, C_6
- Medium electropositive/electronegative atoms C_2, C_5, C_{12}, O_2, O_3, F_1, F_2, N_1, S_1 and F_3
- Low electropositive/electronegative atoms C_1, C_3, C_4, C_7, C_8, C_9, C_{10}, C_{11}, H_i

Finally, the atomic positions are assessed using the cost functions

$$C_{p,hc} = \sum_{i=1}^{N_{hc}} \sum_{\epsilon_z} (d_z \mod 0.25) \qquad \text{and} \qquad C_{p,mc} = \sum_{i=1}^{N_{mc}} \sum_{\epsilon_z} (d_z \mod 0.25) \tag{10}$$

where N_{hc} is the number of highly electropositive/electronegative atoms, N_{mc} the number of medium electropositive/electronegative and d_z the distance of the atom to the nearest plane ϵ_z.

The updated ranking scheme is as follows:

- Form pairs between highly and medium electropositive/electronegative atoms and calculate the number of pairs n_z that are found at distance $d_z = 0.25k \pm 0.025$ (in crystallographic coordinates) along the 9 planes ϵ_z.
- Discard structures for which the number of pairs n_z is zero, for at least one of the 9 possible directions ϵ_z.
- Discard structures for which at least one of the two highly electropositive/electronegative atoms is found at a distance > 0.025 to the nearest plane ϵ_z.
- Discard structures for which none of the two highly electropositive/electronegative atoms is found at a distance < 0.01 to the nearest plane ϵ_z.
- Discard structures for which at least 25% of the medium electropositive/electronegative atoms is found at a distance > 0.025 to the nearest plane ϵ_z.
- Discard structures for which at least 75% of the medium electropositive/electronegative atoms is found at a distance > 0.01 to the nearest plane ϵ_z.
- Discard structures with unphysical or low probability close contacts.
- Rank structures based on the function

$$C = \frac{1}{2}(C_{i,ortho} + C_{i,perp}) + C_{\text{zzp}} + \frac{1}{2}(C_{p,hc} + C_{mc}) \tag{11}$$

In Table 2 we present the new ranking of the structures estimated with the updated scheme.

Table 2: List of the 20 structures that passed all the topological filters ranked in order of C.

**Structure**	**Space group**	$C_{i,ortho}$	$C_{i,perp}$	C_{zzp}	$C_{p,hc}$	$C_{p,mc}$	C
`XXXI_structure_4`	$P2_1$	0.0155	0.0090	0.0449	0.0612	0.0619	0.1187
`XXXI_structure_25`	$P2_1/c$	0.0179	0.0214	0.0576	0.0599	0.0651	0.1397
`XXXI_structure_97`	$P2_1/n$	0.0144	0.0173	0.0607	0.0631	0.0635	0.1398
`XXXI_structure_69`	$P2_1/c$	0.0184	0.0244	0.0649	0.0496	0.0596	0.1409
`XXXI_structure_3`	$P-1$	0.0099	0.0085	0.0680	0.0701	0.0599	0.1422
`XXXI_structure_22`	$P2_1/c$	0.0209	0.0126	0.0630	0.0727	0.0535	0.1428
`XXXI_structure_49`	$P2_1/c$	0.0223	0.0249	0.0678	0.0523	0.0645	0.1498
`XXXI_structure_34`	$P2_1$	0.0158	0.0134	0.0765	0.0602	0.0582	0.1503
`XXXI_structure_98`	$P2_1/c$	0.0295	0.0400	0.0552	0.0623	0.0586	0.1504
`XXXI_structure_89`	$R-3$	0.0164	0.0118	0.0706	0.0740	0.0640	0.1537
`XXXI_structure_35`	$P2_12_12_1$	0.0072	0.0087	0.0965	0.0432	0.0639	0.1580
`XXXI_structure_67`	$P2_1/c$	0.0345	0.0324	0.0641	0.0646	0.0584	0.1590
`XXXI_structure_64`	$P2_1/c$	0.0189	0.0096	0.0925	0.0588	0.0602	0.1663
`XXXI_structure_24`	$P2_1/c$	0.0134	0.0091	0.0913	0.0617	0.0663	0.1666
`XXXI_structure_92`	$Pccn$	0.0251	0.0265	0.0785	0.0639	0.0642	0.1683
`XXXI_structure_96`	$P2_1/c$	0.0433	0.0479	0.0710	0.0514	0.0616	0.1731
`XXXI_structure_85`	$Pccn$	0.0197	0.0246	0.0990	0.0524	0.0681	0.1814
`XXXI_structure_1`	$P2_1/c$	0.0301	0.0358	0.1035	0.0662	0.0594	0.1992
`XXXI_structure_99`	$P2_1/c$	0.0931	0.0972	0.0504	0.0754	0.0632	0.2149
`XXXI_structure_10`	$Pbca$	0.0725	0.0717	0.0909	0.0446	0.0630	0.2168

References

[1] C. R. Groom, I. J. Bruno, M. P. Lightfoot, S. C. Ward, The Cambridge Structural Database. *Acta Cryst.* **B72**, 171-179 (2016)

[2] S. Grazulis, D. Chateigner, R. T. Downs, A. F. T. Yokochi, M. Quiros, L. Lutterotti, E. Manakova, J. Butkus, P. Moeck, A. Le Bail, Crystallography Open Database - an open-access collection of crystal structures. *Journal of Applied Crystallography*, **42** 726-729 (2009)

[3] S. Grazulis, A. Daskevic, A. Merkys, D. Chateigner, L. Lutterotti, M. Quiros, N. R. Serebryanayam, P. Moeck, R. T. Downs, A. Le Bail, Crystallography Open Database (COD): an open-access collection of crystal structures and platform for world-wide collaboration. *Nucleic Acids Research*, **40**, D420-D427 (2012)

[4] J. Gasteiger, M. Marsili, Iterative partial equalization of orbital electronegativity-a rapid access to atomic charges. *Tetrahedron*, **36** (22) 3219-3228 (1980)

7. Group 9

Michal Hušák

Department of Solid State Chemistry, University of Chemistry and Technology, Prague, Technická 5, Praha 6, 166 28, Czech Republic

Submission for phase 2 - 7-th CSP blind test. DFT ranking based on rSCAN + MBD dispersion correction

The input CIF files were transformed to CASTEP 20.11 (Clark et al., 2005) input files by BIOVIA Material Studio software (BIOVIA, 2020). Structures described in centred cells were transformed to their non-centred equivalent to speed up the calculation. The main calculations were done on "Karolina" national supercomputer. A full geometry optimization (including lattice parameters) was performed. For the energy calculation the rSCAN functional (Bartók et al., 2019) in combination with MBD dispersion correction was used (on the fly ultrasoft pseudopotentials). The calculation was finished when it reached "Fine" criteria presets (see Table 1) or 200 geometry optimization steps. The structures were ranked based on final enthalpy (entropy not used).

Notice for discussion: The target of this work was to evaluate, whatever the meta-GGA functional like rSCAN gives any benefit in comparison to the GGA fucntionals (typically PBE) used in previous blind test. In the post-submission phase we had re-ranked the first 20 structure from XXVII, XXXI, XXXIII by the old-fashioned PBE functional with D3 dispersion correction. The results were better or identical than the rSCAN+MBD use.

Table 1: Key calculation parameters:

Property	value	Units
plane wave basis set cut-off	XXVII : 408.20<br>XXXI,XXXIII: 571.40	eV
k-Points For BZ Sampling scheme	1x2x1	
max ionic |force| tolerance	0.3000E-01	eV/Å
max ionic |displacement| tolerance	0.1000E-02	Å
max |stress component| tolerance	0.5000E-01	GPa
total energy / atom convergence tol. for SCF cycle	0.1000E-05	eV

Reference:

Bartók, A. P. & Yates, J. R. (2019). *J. Chem. Phys.* **150**, 161101-1-161101-5

BIOVIA, Dassault Systems (2020), Material Studio 2020

Clark, S. J. , Segall, M. D. , Pickard, C. J. , Hasnip, P. J. , Probert, M. J. , Refson, K & Payne M. C. (2005). *Z. Kristallogr.* **220(5-6),** 567-570

This work was supported by the Ministry of Education, Youth and Sports of the Czech Republic through the e-INFRA CZ (ID:90254)

8. Group 10

Supplementary information for the 7th Blind Test phase two submission of Group 10

Yingdi Jin, Zhuocen Yang, Lu Tan, Chao Chang, Wenbo Fu, Bochen Li, Yunfei Zhou, Jiuchuang Yuan, and Guangxu Sun

XtalPi, Shenzhen Jingtai Technology Co., Ltd., International Biomedical Innovation Park II 3F, 2 Hongliu Rd, Futian District, Shenzhen, China

Methodology

The polymorph landscape for each compound is predicted with XtalCSP. XtalCSP is an intelligent crystal structure prediction platform for real industrial production practice based on cloud-computing [1]. This platform consists of two main parts as shown in figure 1, one is the automated crystal structure prediction process (the lower part of figure 1.), and the other is a decision tree (the upper part of the process) for controlling the CSP process. The automated CSP process integrates many components, including conformation analyses, force-field parameterization, crystal structure generation, clustering, high-precision ranking, free energy calculation and crystallizability analysis, etc. Each component contains a series of algorithms to adapt to various systems. And the decision tree (the upper part of the process) assigns appropriate algorithms for each component to adapt to the current system. The decision-making basis of the decision tree in each component of the CSP process is different. This document will focus on the zero-temperature ranking and free energy calculation part (orange box in figure 1). The whole stability evaluation process is mainly divided into three steps.

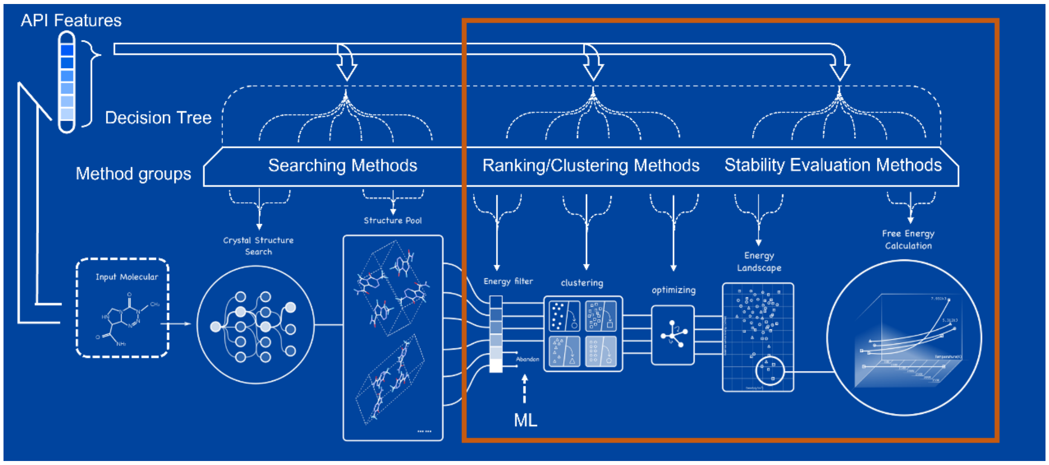

Figure 1

First, XtalCSP uses a hierarchical ranking process and AI correction method to obtain the final landscape. The number of structures generated from the structure searching stage usually ranges from millions to tens of millions, even after clustering. Since the energy correlation between the force field and QM level calculation (usually DFT) is not always perfect, it means that some really low-energy structures may be overestimated, while many high-energy structures may also be underestimated. If a cutoff value is directly set for the force field calculated energy and the high-

energy structures above the cutoff value are eliminated, the cutoff energy must be very high to ensure that no real low-energy structure is thrown away because its energy is calculated too high at force field level. At the same time, the number of structures below the cutoff energy increase exponentially with the increases of the cutoff value. This makes it a huge computational cost if all the remaining structures are calculated with QM accuracy. Therefore, we try to solve this problem from two aspects. On the one hand, the semi-empirical (such as DFTB) method is added between the force field calculation and QM calculation, meanwhile several layers of low precision QM calculation are added before the final high-precision energy calculation to screen out most of the high-energy structures layer by layer, so that the number of structures that finally enter into high-precision calculation is up to hundreds or thousands. On the other hand, an energy corrector is added between adjacent layers to improve the correlation between the energies calculated with different precision. For example, adding an energy corrector between the energies calculated by the force field and DFTB can reduce the cutoff value so that the number of structures entering the DFTB stage will naturally decrease. The energy corrector adopts a deep neural network developed based on Graph Neural Networks (GNN) technology [2,3,4]. The input of the energy corrector is the crystal structure. The preprocessing algorithm extracts the spatial topological structure of the atoms in the crystal and provides it to the subsequent graph neural network for training in the form of a graph. The energy corrector is trained based on a small amount of crystal energy calculated by tight convergency criteria, and the trained model can correct the crystal energy calculated by the less accurate but efficient methods. The lattice energies of the landscape are usually evaluated using the optPBE-vdW [5] exchange-correlation functional corrected for dispersion effects as implemented in the Vienna ab Initio Simulation Package (VASP) [6,7,8].

Then, the decision tree will select one high-precision method to re-evaluate the lattice energy in the landscape based on the MP2-benchmark method [9]. For a specific system, multiple representative molecular pair geometries are extracted from the several most stable virtual forms for intermolecular energy estimations at PBE0-MBD, PBE0-D3, optPBE-vdW, B3LYP-D*, R2SCAN-D4 and SCS-MP2 levels of theory. The method with results closest to MP2 is selected as the high-precision method for calculating the final energy calculation.

Because the structure of phase two of this 7th Blind Test is determined, we didn't screen out any virtual structure during the standard ranking process. However, we use the above layer by layer screening method in process of generating landscape in phase one.

Finally, the free energy at room temperature is calculated. The Einstein Crystal Method (ECM) based on classical molecular dynamics, is an approach for computing the relative free energies of polymorphs using a thermodynamic pathway connecting any two polymorphs. In this method, a series of intermediate states are used to smoothly connect two or more putative polymorphs to the ideal non-interacting Einstein Crystal (EC) state from which the free energy can be computed using thermodynamic integration (TI) or the Bennett Acceptance Ratio (BAR). Then by evaluating the free energy of the non-physical Einstein Crystal (EC) state the free energy difference between the two physical polymorphs can be computed. In respect of the definition of the analytical state and the precise thermodynamic path, we adopt a methodology similar to the pseudo super-critical path (PSCP) method. The advantage of this method is that it can effectively sample the ensemble of

configurations [10]. However, since this method is based on classical molecular dynamics, its accuracy strongly depends on the quality of the force field. To increase the reliability of the results, we used a two steps crossing-validation method to ensure the accuracy of the final output. The first step is to use a variety of different methods to fit the force field parameters, obtain several versions of force field parameters, and calculate the free energies of several low-energy structures. The second step is to implement the free energy calculation process based on QM level lattice dynamics and harmonic approximation method DFT-HA. Then, the decision tree compares the relative free energy trends in the low temperature between the results of PSCP with different force fields and that of DFT-HA, and select the most suitable version of the force field for the current system to execute the free energy calculation of the required virtual crystal forms in the full temperature range. As well known, the DFT-HA method is limited by its resonance approximation and cannot well describe the contribution of anharmonic effect at high temperatures. However, it can be used as a good checkpoint for the selection of a good force field in this implementation. In this way, the uncertainty of the relative free energy caused by the force field can be eliminated.

Table 1. Lattice energy and Free energy method

	High-precision Ranking method	Free energy method
XXVII	optPBE-vdW	PSCP
XXVIII	r^2SCAN	DFTB-HA
XXXI	PBE0-MBD	PSCP
XXXII	PBE0-MBD	PSCP
XXXIII	PBE0-MBD	PSCP

*For XXVIII, we use different force field parameters for the two coordination configurations, which makes the PSCP free energy between them incomparable. Therefore, the DFTB-HA is used here, and only the free energy at low temperature are provided.

Alignment of experimental and predicted structures

Table 2 shows the RMSD20 calculation results and the structure overlays between second round re-ranked crystal structures and the published experimental solved crystal structures of each 7th blind test target. In table 2, we provide the 0K rank and room temperature (300K) corrected rank of each round 2 submitted crystal structure. Similar to the round 1 result comparison, dynamic disordered experimental forms are considered as a single crystal form, and static disordered experimental forms are considered as different crystal forms.

Table 2. Alignment of experimental and predicted structures

Target	0K rank	300K rank	RMSD20	Structure overlay

XXVII Form A	4	4	0.62 Å	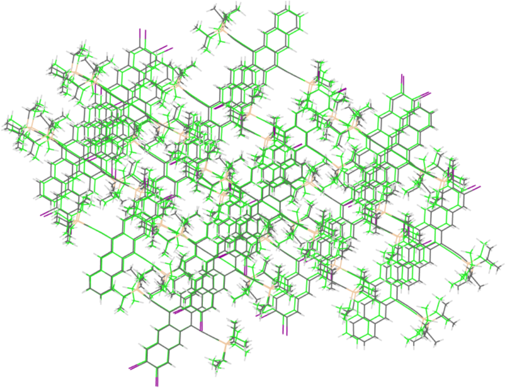
XXVIII Form A	N/A	1	0.181 Å	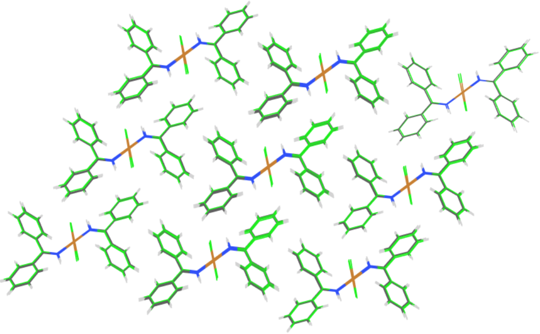
XXXI Form A Major	8	7	0.127 Å	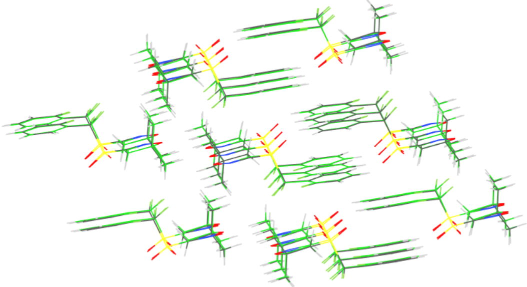
XXXI Form A Minor	11	8	0.244 Å	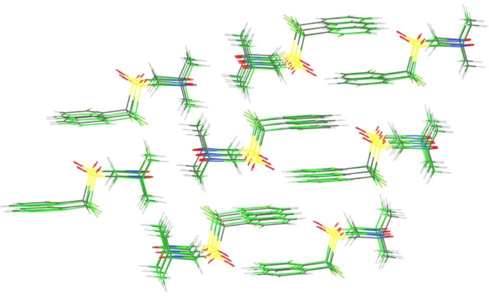

XXXI Form B	1	3	0.227 Å	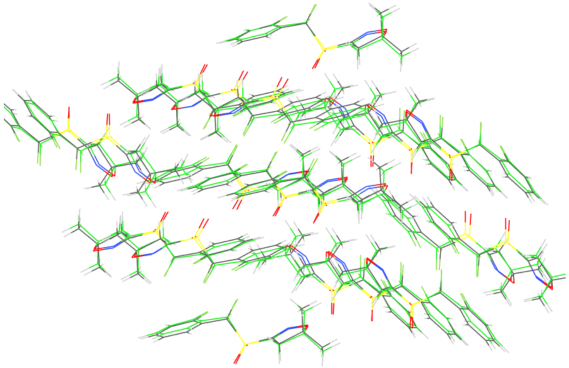
XXXI Form C	42	5	0.119 Å	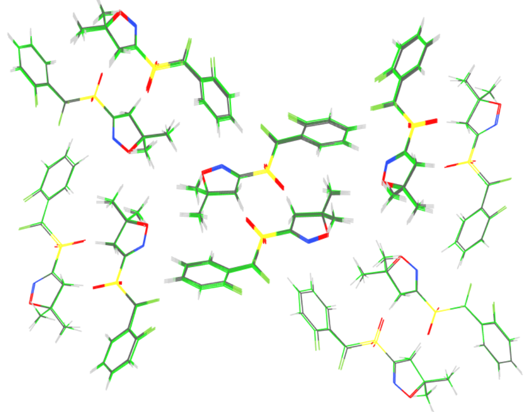
XXXII Form A	13	5	0.189 Å	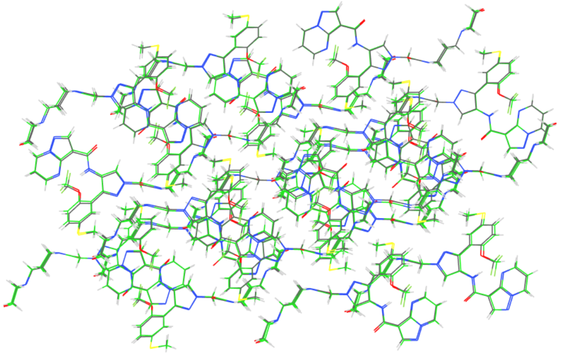
XXXII Form B	30	51	0.226 A	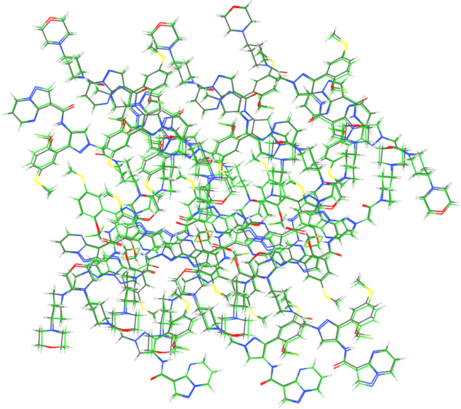

XXXII Form B RT	N/A	N/A		
XXXIII Form A	1	1	0.179 Å	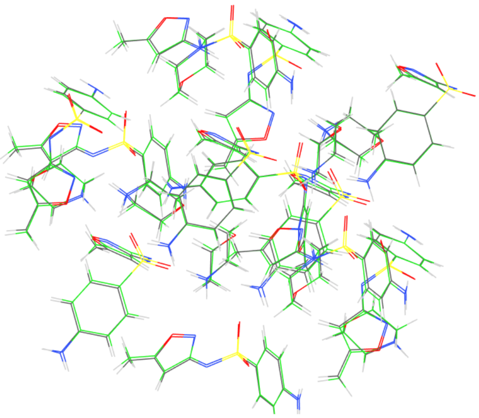
XXXIII Form B	7	4	0.149 A	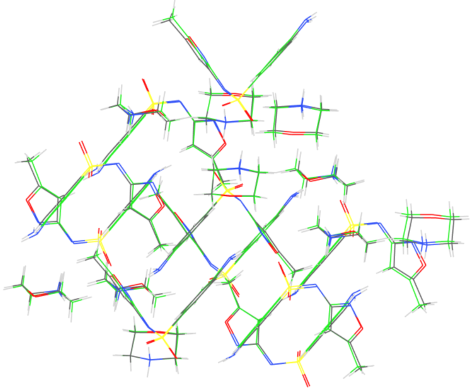

DFT Method for matal-organic system (Target XXVIII)

Based on the density functional theory (DFT) and the projector augmented-wave (PAW) method[11] as implemented in the Vienna Ab-initio Simulation Package (VASP), the r^{2}SCAN-D4 electronic structure method that shows exceptional performance across metal organic compounds and molecular crystals assessed by thousands of high-level data points in a number of comprehensive benchmark sets[12] were used to perform the energy minimization of crystal structures with a force convergence criterion of 0.002 eV/Å. The kinetic energy cutoff of plane-wave basis set[13] and the k-points spacing of the Brillouin zone integration were set to 600 eV and 0.05/Å, respectively.

DFTB Method for silicon and copper system (Target XXVII)

The density functional tight binding model DFTB3[14] as implemented in the DFTB+ package[15] was used to perform the energy and force calculations. The parametrizations of Si (Si-C, Si-H, Si-I)

and Cu (Cu-C, Cu-H, Cu-N, Cu-Cl) were extended from DFTB3/3OB parameter set[16,17]. The electronic parameters for Si and Cu were adopted from the reports of Wahiduzzaman et al. [18] and Gaus et al. [19], respectively. The repulsion potentials for Si and Cu are obtained by solving a system of linear equations formed by combinations of continuity equations, energy equations, force equations, additional equations, and reaction equations using an automatized method [20]. Parameters defining the energy, force, continuity, and additional equations of Si with C, H, and I were listed in the Table 1. V'' is the value of frequency at a point on the potential energy curve.

Table 3. Parameters Defining the Repulsive Potentials of Si

molecules	reference energies (au)	including force
H3Si-SiH3	-0.8894837	YES
SiH4	-0.5320268	YES
H3Si-CH3	-1.05893786	YES
H3Si-C(H)CH2	-1.336806	YES
H3Si-CCH	-1.1077777	YES
H3Si-C(H)(CH3)CH3	-2.0776377	YES
H3Si-I	-0.51173825	YES
**potentials**	**division points (au)**	**additional equations (au)**
Si-Si	(4.4, 4.8, 5.4)	V''(4.449) = 0.104
Si-H	(2.7, 3.0, 3.7)	V''(2.746) = 0.210
Si-C	(3.4, 3.8, 5.4)	V'' (3.414) = 0.241
Si-I	(4.6, 5.0, 5.6)	V'''(4.610) = 0.122

Parameters defining the energy, force, continuity, additional and reaction equations of Cu with C, H, O, N, and Cl were listed in the Table 2.

Table 4. Parameters Defining the Repulsive Potentials of Cu

molecules	reference energy (au)	including force
CuCl2(N(H)CH2)2 cf_1	-2.006	NO
CuCl2(N(H)CH2)2 cf_2	-2.028	NO
(CuCl2(N(H)CH2)2)2 cf_3	-	YES
(CuCl2(N(H)CH2)2)2 cf_4	-	YES
(CuCl2(N(H)CH2)2)2 cf_5	-	YES
CuH2	-0.125	YES
CuC	-0.14	YES
**potential**	**division points (au)**	**additional equations (au)**
Cu-Cu	(6.0, 6.4, 7.0)	V'' (6.047) = 0.106
Cu-C	(5.4, 5.8, 6.0)	-
Cu-H	(2.6, 3.0, 3.6)	V'' (2.801) = 0.165
Cu-N	(3.6, 4.5, 6.0, 6.4)	V'' (3.815) = 0.081
Cu-Cl	(4.2, 4.9, 5.5, 6.4)	V'' (4.318) = 0.079
**reactions**		**delta energy (au)**
2 cf_1 → cf_4		-0.0377
2 cf_1 → cf_5		-0.0324
2 cf_2 → cf_3		-0.0095

Static and Dynamic Disorder Discussion

The disorder phenomenon has been widely observed in the experimental forms of the 7th Blind Test targets. More specifically, based on experimental CIF files provided by the 7th Blind Test team, Target XXVII Form A, Target XXX Form B, Target XXXI Form A, and Target XXXII Form A have disordered atoms/functional groups in the cell. Disorders in the experimental obtained single crystals can be classified as two different types: the static disorder, known as a crystal formed by APIs with different conformations; and the dynamic disorder, shown as terminal groups to be comparably flexible at higher temperatures. It is worth evaluating the disorder type of these disordered experimental forms to better validate the CSP result with experimental data. Molecular dynamic (MD) simulation would be a useful tool to analyze the disorder type since the dynamic disorder is expected to be captured by the simulation at higher temperatures. If the disordered group of a disordered experimental form is not very flexible during the MD simulation at higher temperatures, the corresponding disorder is considered to be a static disorder since the disordered atoms tend to remain unchanged at higher temperatures, supporting that the experimental form is formed by APIs with slightly different conformations. On the contrary, if the disordered terminal groups become more and more flexible as temperature increase, the experimental form is suspected to have a dynamic disorder because the temperature effect contributes to the flexibility of those terminal groups and result in atoms/terminal groups with different occupancy.

We have performed MD simulation for experimental forms with disorders, which are Target XXVII Form A, Target XXX Form B, Target XXXI Form A, and Target XXXII Form A. (MD parameters details). Here, we use the torsion angle distribution to demonstrate the disorder behavior at different temperatures. The change of selected torsion angles is directly correlated to the disorder behavior of each disordered group. Disordered groups and corresponding analyzed torsion angles are shown in table 5.

A significant disorder has been observed in the 290K experimental obtained crystal structure of XXVII Form A. The dihedral distribution of terminal carbon groups suggests that these carbon groups are comparably flexible during 300K MD simulation. This broad torsion angle distribution indicates that target XXVII Form A may have a dynamic disorder rather than a static disorder because the disorder can be captured using MD simulation at higher temperatures. Similar broad dihedral distribution was also observed in the XXX Form B and XXXII Form A. Interestingly, the MD simulation of XXX Form B indicates that the minor component could be the more preferable and observable component at higher temperatures, shown by the torsion angle distribution of XXX Form B major/minor at 300K: the terminal carbon tails in both major and minor component tend to have similar conformation with the minor component in the 300K MD simulation.

On the contrary, 300K MD simulation suggests the fluorobenzene ring is not able to flip to the other side at higher temperatures, stating that the disorder in the experimentally solved crystal structure of target XXXI Form A could be a static disorder since the ring flipping energy barrier is still significant at higher temperatures. Please notice that due to the symmetry operation of the P 21/c space group, half of the APIs in the cells would have torsion angles with negative values compared to the other half of the APIs.

Table 5. Disorder analysis

Target	Disorder Group	Torsion Angle Distribution
XXVII<br>Form A	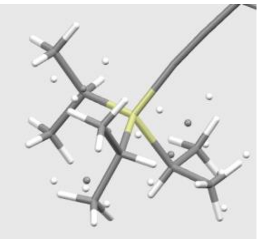	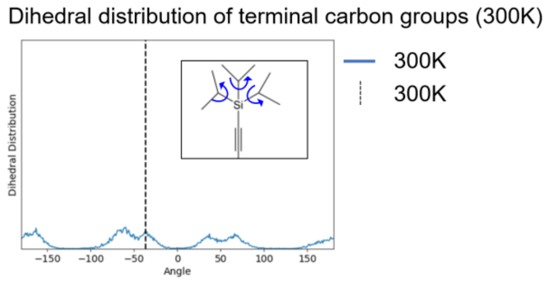
XXX<br>Form B	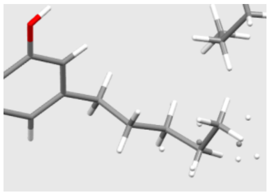	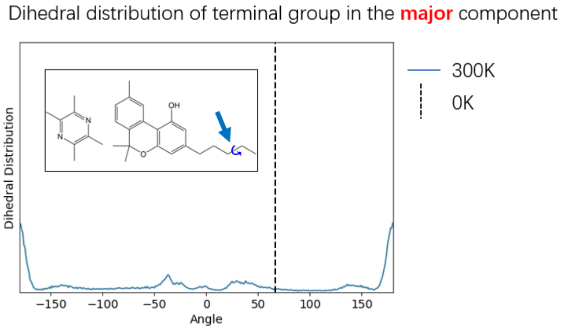<br>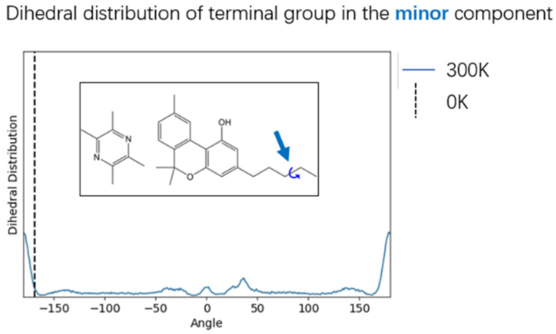

<table>
<tr><td>XXXI Form A</td><td>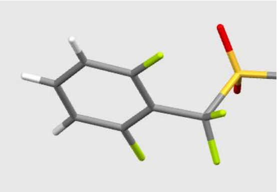</td><td>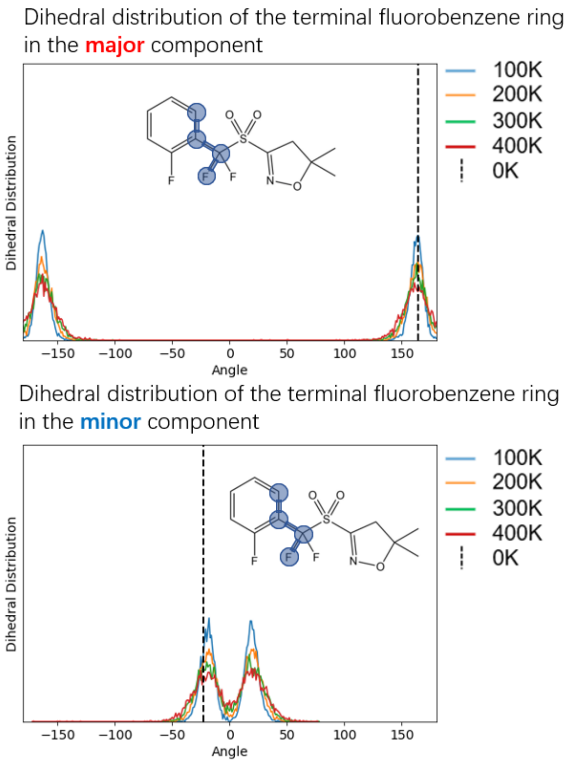
</td></tr>
<tr><td>XXXII Form A</td><td>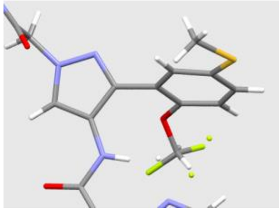</td><td>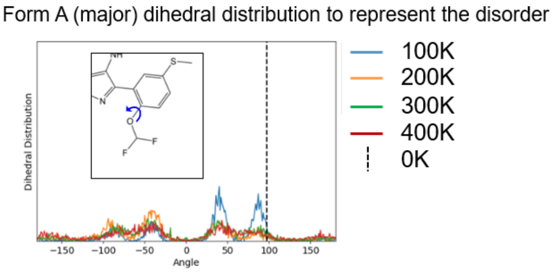
</td></tr>
</table>

Reference:

1. Zhang, Peiyu, Wood, Geoffrey P. F., Ma, Jian, Yang, Mingjun, Liu, Yang, Sun, Guangxu, Jiang, Yide A., Hancock, Bruno C., Wen, Shuhao, Cryst. Growth Des.,18(11), 6891-6900 (2018)
2. Tian Xie and Jeffrey C. Grossman, Crystal Graph Convolutional Neural Networks for an Accurate and Interpretable Prediction of Material Properties, Phys. Rev. Lett. 120, 145301 (2018)
3. Chi Chen, Weike Ye, Yunxing Zuo, Chen Zheng, and Shyue Ping Ong, Graph Networks as a Universal Machine Learning Framework for Molecules and Crystals, Chem. Mater., 31, 9, 3564–3572 (2019)
4. Choudhary, K., DeCost, B. Atomistic Line Graph Neural Network for improved materials property predictions, npj. Comput. Mater. 7, 185 (2021)
5. Klimeš, Jiří, David R. Bowler, and Angelos Michaelides. Van der Waals density functionals

applied to solids, Phys. Rev. B 83.19, 195131 (2011)

6. Kresse, G.; Hafner, J., Ab-initio molecular-dynamics for liquid-metals, Phys. Rev. B, 47, 558 (1993)
7. Kresse, G.; Hafner, J., Ab-initio molecular-dynamics simulation of the liquid-metal amorphous-semiconductor transition in germanium, Phys. Rev. B, 49, 14251 (1994)
8. Kresse G.; Furthmueller J., Efficiency of ab-initio total energy calculations for metals and semiconductors using a plane-wave basis set Comput. Mat. Sci., 6, 15 (1996)
9. Yuriy A. Abramov, Bochen Li, Chao Chang, Qun Zeng, Guangxu Sun, and Gianpaolo Gobbo, Uncertainty Distribution of Crystal Structure Prediction. Cryst. Growth Des., 21, 5496-5502 (2021)
10. Mingjun Yang, Eric Dybeck, guangxu sun, chunwang peng, Brian M. Samas, Virginia Burger, Qun Zeng, Yingdi Jin, Michael A. Bellucci, Yang Liu, Peiyu Zhang, Jian Ma, Alan yide Jiang, Bruno C Hancock, Shu-Hao Wen, and Geoffrey P. F. Wood, Prediction of the Relative Free Energies of Drug Polymorphs Above Zero Kelvin Cryst. Growth Des, 20(8) - 5211 ~ 5224 (2020)
11. Kresse, G. & Joubert, D. From ultrasoft pseudopotentials to the projector augmented-wave method. Phys. Rev. B 59, 1758–1775 (1999).
12. Ehlert, S. et al. r 2 SCAN-D4: Dispersion corrected meta-generalized gradient approximation for general chemical applications. J. Chem. Phys. 154, 061101 (2021).
13. Kresse, G. & Furthmüller, J. Efficiency of ab-initio total energy calculations for metals and semiconductors using a plane-wave basis set. Comput. Mater. Sci. 6, 15–50 (1996).
14. Gaus, M., Cui, Q. & Elstner, M. DFTB3: Extension of the Self-Consistent-Charge Density-Functional Tight-Binding Method (SCC-DFTB). J. Chem. Theory Comput. 7, 931–948 (2011).
15. Hourahine, B. et al. DFTB+, a software package for efficient approximate density functional theory based atomistic simulations. J. Chem. Phys. 152, 124101 (2020).
16. Gaus, M., Goez, A. & Elstner, M. Parametrization and Benchmark of DFTB3 for Organic Molecules. J. Chem. Theory Comput. 9, 338–354 (2013).
17. Kubillus, M., Kubař, T., Gaus, M., Řezáč, J. & Elstner, M. Parameterization of the DFTB3 Method for Br, Ca, Cl, F, I, K, and Na in Organic and Biological Systems. J. Chem. Theory Comput. 11, 332–342 (2015).
18. Wahiduzzaman, M. et al. DFTB Parameters for the Periodic Table: Part 1, Electronic Structure. J. Chem. Theory Comput. 9, 4006–4017 (2013).
19. Gaus, M. et al. DFTB3 Parametrization for Copper: The Importance of Orbital Angular Momentum Dependence of Hubbard Parameters. J. Chem. Theory Comput. 11, 4205–4219 (2015).
20. Gaus, M., Chou, C.-P., Witek, H. & Elstner, M. Automatized Parametrization of SCC-DFTB Repulsive Potentials: Application to Hydrocarbons. J. Phys. Chem. A 113, 11866–11881 (2009).

9. Group 11

Description of Methods used in Phase 2 of the BT7 Submission

Alastair J. A. Price,[†] Alberto Otero-de-la-Roza,[*,‡] and Erin R. Johnson[*,†]

Department of Chemistry, Dalhousie University, 6274 Coburg Rd, Halifax, Nova Scotia, B3H 4R2, Canada, and Departamento de Química Física y Analítica and MALTA Consolider team, Facultad de Química, Universidad de Oviedo, 33006 Oviedo, Spain

E-mail: aoterodelaroza@gmail.com; erin.johnson@dal.ca

All calculations used an in-house modified version of FHI-aims[1] version 210513. Geometry optimisations were performed using the B86bPBE functional[2,3] with the XDM dispersion correction,[4] the "light" basis set, and a "dense" integration grid, with a relaxation convergence threshold of 0.025 eV/Å. For all structures within 1.5 kcal/mol per molecule of the minimum, further geometry optimization was performed with a tighter relaxation threshold of 0.005 eV/Å. Subsequent single-point energy calculations were then performed on all optimised structures using either the hybrid B86bPBE-25X or B86bPBE-50X functionals with XDM dispersion; please refer to Ref. 4 for the damping function parameters. For compound XXVIII, all calculations were run for a ferromagnetic configuration with one unpaired electron per copper atom.

[*]To whom correspondence should be addressed
[†]Dalhousie University
[‡]Universidad de Oviedo

References

(1) Blum, V.; Gehrke, R.; Hanke, F.; Havu, P.; Havu, V.; Ren, X.; Reuter, K.; Scheffler, M. Ab initio molecular simulations with numeric atom-centered orbitals. *Comp. Phys. Comm.* **2009**, *180*, 2175–2196.

(2) Becke, A. D. On the large-gradient behavior of the density functional exchange energy. *J. Chem. Phys.* **1986**, *85*, 7184.

(3) Perdew, J. P.; Burke, K.; Ernzerhof, M. Generalized gradient approximation made simple. *Phys. Rev. Lett.* **1996**, *77*, 3865.

(4) Price, A. J. A.; Otero-de-la-Roza, A.; Johnson, E. R. XDM-corrected hybrid DFT with numerical atomic orbitals predicts molecular crystal lattice energies with unprecedented accuracy. *Chem. Sci.* **2023**, DOI: 10.1039/D2SC05997E.

10. Group 12

Supplementary Information for Seventh CCDC Blind Test

K. V. Jovan Jose and Gunjan R. Ramteke

School of Chemistry, University of Hyderabad, Hyderabad, India

Email: jovanjose@uohyd.ac.in

The wide-ranging distribution of crystal polymorphs over the entire potential energy surface (PES) makes it difficult to determine the most stable. Lattice energy is the measure to quantify the stability of organic molecular crystals. However, its reliable and fast determination is too expensive, even with the assistance of advanced computer hardware and algorithms. Compared to experimental data, periodic density functional theory (DFT) is assumed to yield the lattice energy with a chemical accuracy of nearly 5-7 kJ/mol. Still, its employment in CSP is cost-prohibitive, and the usage of force field methods remains questionable regarding chemical accuracy. This limitation made us adopt an alternative route by employing semi-empirical tight binding procedures, particularly Density Functional based Tight Binding (DFTB) methods; a parameter-specific method for the electronic part and the repulsive energy contribution.(Hourahine *et al.*, 2020) Combining DFTB methods with kernel-based Gaussian process regression (GPR) method speeds up the energy evaluation procedure based on calculating the limited number of sample structures. (Deringer *et al.*, 2021)

GPR is a kernel-based approach; well-suited for small-sized databases. Dataset generation is crucial as the strength of the GPR model depends on the quantity and diversity of structures. We devised a sampling procedure assisted by molecular electrostatic potential (MESP), resulting in diverse geometry and energetics polymorphism. These structures and the corresponding energies are utilized for training the GPR model, enabling energy prediction when the unknown structural geometry is introduced.(Denzel & Kästner, 2018)

It is a nonparametric regression tool to interpolate between data points in the training data set. Here the gaussian process is exploited to fit the energy against the structural geometries constituting the training dataset. The X is configurations in the training data collection, and E is the energy as labeled property.

$$X = (x_1, x_2, \dots, x_N)^T \quad (1)$$

$$E = (E'_1, E'_2, \dots, E'_N)^T \quad (2)$$

The GPR method predicts energy for any structure by deducing the distribution over a function in the equation.

$$p\left(E_p | X, E\right) \quad (3)$$

The gaussian process is defined by the prior mean function $\mu(x)$, which is the initial assumption of the target function; and covariance function $k\left(x_i, x_j\right)$ between two random variables. For the new unknown geometry, the uncertainty σ'_p and energy E'_p is estimated as

$$E_p'(x') = k'^T(K + \sigma_n^2 I)^{-1}[E - \mu(x)] + \mu(x) \quad (4)$$

$$\sigma_p'(x')^2 = k(x', x') - k'^T(K + \sigma_n^2 I)^{-1}k \quad (5)$$

Here, $K = k(X, x)$ and $k' = k(X, x')$, and σ_n^2 is the uncertainty regularization parameter. The mean of a gaussian process is assumed to be a normal distribution and is given by equation 5. The GPR speeds up the search and extends the exploration of PES.

Table 1. E^{lat} (Lattice energy) and E^{tot}(total energy) for XXVIII., here, the monomer energy is -1167.9659 eV.

Target	Polymorphs	E^{tot} (eV)	E^{lat} (kJ/mol)
XXVIII	Form A	-2337.4035	-70.99

Table 2. E^{lat} (Lattice energy) is computed using E^{pbc}, and the PBC contributions are depicted in eV.

Target	Polymorphs	E^{UC} (eV)	E^{pbc}(eV)	E^{lat} (kJ/mol)	PBC contribution (eV)
XXXI	Form A	-5733.3817	-5733.9055	-19.32	-0.52
	Form B	-6161.4405	-6161.8493	-13.74	-0.40
	Form C	-25776.64	-25778.316	-17.38	-1.66
XXXII	Form A	-	-	-	-
	Form B	-11392.4885	-11392.9685	-68.64	-1.48
XXXIII	Form A	-12221.0582	-12232.1460	-102.087	-11.08
	Form B	-	-	-	-

The energy evaluation of the training dataset was carried out by the parameter-based DFTB method. The parameters preferred were specific to the system, involving a particular type of bonded and non-bonded interactions. XXVII comprises atoms I and Si, better interpreted by GFN-xTB self-consistent density functional TB method.(Bannwarth *et al.*, 2019) GFN-xTB describes the non-covalent halogen bond potential and D3 dispersion correction. The same is employed to treat target XXVIII, where Cu-Cl interaction potential is accounted for. The calculations for the experimentally observed structure of XXVIII are depicted in Table 1. The experimentally observed structure is ranked at 62 positions in the list, as illustrated in Figure 4(A). Target XXIX is treated by Slater-Koster parameterization in PBC, which is used mainly for periodic solid-state systems. 3ob parameterization accounts for the electrostatics, charge transfer and polarization which satisfy the targets XXXI, XXXII, XXXIII criteria.(Kubillus *et al.*, 2015)(Gaus *et al.*, 2013)(Gaus *et al.*, 2014)

3ob remains unaccountable of periodic boundary conditions, which inspired us to frame a technique that attempts to mimic the surrounding unit cell image interactions. The energy of a single-unit cell E^{UC} is calculated. To capture its interactions with the surrounding, the unit cell

is periodically replicated in the positive X, Y, and Z crystallographic axes, constructing three different supercells. The performance of energy calculation on each of the supercells results in the energies E^{x}, E^{y}, and E^{Z}, respectively. The energy of a single unit cell is updated with its interactions with the surrounding images using equation 6. The neighbor contributed energy E^{pbc} represent the interactions of the unit cell with neighboring images.(Jose & Raghavachari, 2015)

$$E^{pbc} = (E^{X} + E^{Y} + E^{Z}) - (E^{UC} * 5) \tag{6}$$

Equation 6. can be modified accordingly for the systems such as slabs (two-dimensional), or rods (one-dimensional) and extended for more than one image, allowing the system to capture interactions at an extended range by propagating the calculations in desired dimension. We employed equation 6. to evaluate energy for experimentally located crystals of polymorphic forms of target XXXI, XXXII, and XXXIII, depicted in Table 1. The lattice energies calculated with E^{pbc} and the neighboring image contributions are depicted as well in Table.1. The three polymorphs of XXXI are situated in PES with a difference of 2-6 kJ/mol; they are ranked as 22, 25, and 43, depicted in Figure 4(C). For targets XXXII and XXXIII, only one polymorph is reported, as the other forms are observed to have improper crystallographic parameters. For target XXXII, the experimental polymorph appeared to be the most energetically stable, as shown in Figure 4(A). The observed structure of target XXXIII has ranked at 232 positions, as depicted in Figure 4(D). Figure 1 exhibits the ranking of structures according to the energy and position of the experimentally observed polymorphs illustrated in red. The plots are relative to the lowest energy structure. In the plot, few geometries are predicted to be energetically lower than the experimentally observed ones, implicating uncertainties in the procedure designed for global search.

We look forward to introducing the current models with a more accurate description of dispersion and dipole-dipole interaction facilitated by GPR potentials.

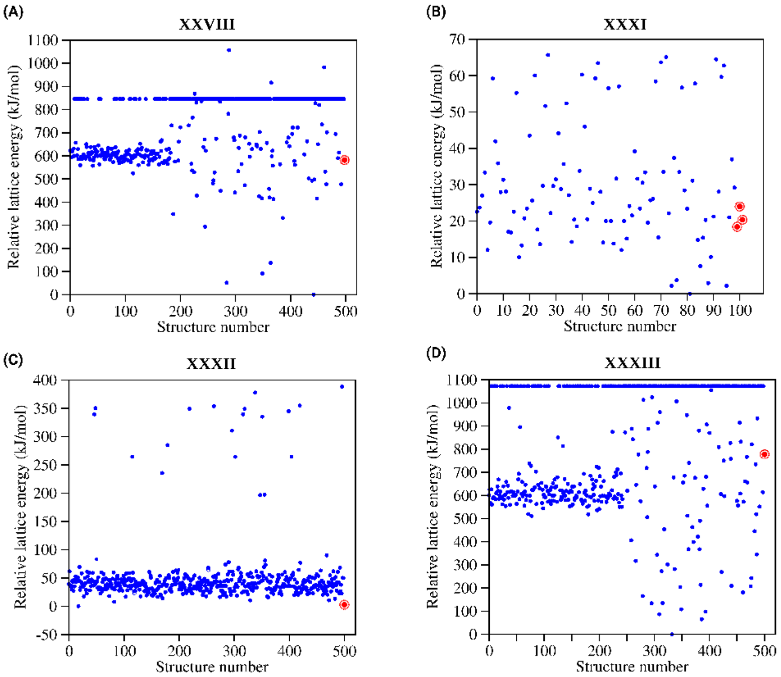

Figure 1. The ranking of structures according to energy ad the experimentally observed structures are depicted in red.

References

Bannwarth, C., Ehlert, S. & Grimme, S. (2019). *J. Chem. Theory Comput.* **15**, 1652–1671.

Denzel, A. & Kästner, J. (2018). *J. Chem. Phys.* **148**, https://doi.org/10.1063/1.5017103.

Deringer, V. L., Bartók, A. P., Bernstein, N., Wilkins, D. M., Ceriotti, M. & Csányi, G. (2021). *Chem. Rev.* **121**, 10073–10141.

Gaus, M., Goez, A. & Elstner, M. (2013). *J. Chem. Theory Comput.* **9**, 338–354.

Gaus, M., Lu, X., Elstner, M. & Cui, Q. (2014). *J. Chem. Theory Comput.* **10**, 1518–1537.

Hourahine, B., Aradi, B., Blum, V., Bonafé, F., Buccheri, A., Camacho, C., Cevallos, C., Deshaye, M. Y., Dumitric, T., Dominguez, A., Ehlert, S., Elstner, M., Van Der Heide, T., Hermann, J., Irle, S., Kranz, J. J., Köhler, C., Kowalczyk, T., Kubař, T., Lee, I. S., Lutsker, V., Maurer, R. J., Min, S. K., Mitchell, I., Negre, C., Niehaus, T. A., Niklasson, A. M. N., Page, A. J., Pecchia, A., Penazzi, G., Persson, M. P., Řezáč, J., Sánchez, C. G., Sternberg, M., Stöhr, M., Stuckenberg, F., Tkatchenko, A., Yu, V. W. Z. & Frauenheim, T. (2020). *J. Chem. Phys.* **152**, https://doi.org/10.1063/1.5143190.

Jose, K. V. J. & Raghavachari, K. (2015). *J. Chem. Theory Comput.* **11**, 950–961.

Kubillus, M., Kubař, T., Gaus, M., Řezáč, J. & Elstner, M. (2015). *J. Chem. Theory Comput.* **11**, 332–342.

11. Group 14

Random phase approximation for polymorph ranking

Jiří Klimeš

Department of Chemical Physics and Optics,

Faculty of Mathematics and Physics, Charles University,

Ke Karlovu 3, CZ-12116 Prague 2, Czech Republic

(Dated: December 20, 2022)

I. COMPUTATIONAL METHODOLOGY

To obtain the energy ranking a two step procedure was performed. First, the structures were optimized using a density functional theory approximation (DFT), specifically the van der Waals density functional (vdW-DF) with the optPBE exchange functional.[1–5] The energy was subsequently obtained using the random phase approximation (RPA). The input states for RPA were calculated with the SCAN functional.[6] The set-up and monitoring of the calculations and the analysis of the results was semi-automated using Python scripts within a Jupyter notebook environment.

All the energy calculations were performed using the Vienna ab-initio simulations package (VASP).[7–9] The `_GW` version of the standard projector-augmented wave (PAW) data-sets were used. The RPA calculations were performed using the cubic-scaling implementation in VASP.[10–12] Due to the large memory demands of the RPA calculations for systems with a large number of k-points we used an older version of the implementation of evaluation of the RPA energy. The older version scales quadratically with the number of k-points, instead of linearly as the version available in VASP 6. This leads to increased computational time but reduces the memory demands which can be more limiting for the systems considered.

The geometry optimization with the optPBE-vdW functional used standard projector augmented wave (PAW) potentials.[9,13] The cut-off energy for the plane-wave basis set was set to 1200 eV in order to reduce Pauli stress to a minimum. The k-point grid was set automatically using a tag `KSPACING=0.3`. The optimization was done in two steps. First, the unit cell and the positions of the atoms were optimised setting a criterion for the largest force on atom to 0.02 eV/Å. Subsequently, only the atomic positions were optimised with a force cut-off of 0.001 eV/Å.

The RPA energy was calculated as a sum of the exact exchange energy (EXX) and the RPA correlation energy. For either of the energy components we used several settings, denoted as `low`, `medium`, and `high` to help to assess the convergence of the energy differences on the numerical parameters. The parameters that are changed by the different settings are listed in Table I. Note that for EXX we used again the `KSPACING` tag while for RPA the k-point grids were obtained with the `autoGR` program[14] and modified manually in case of an undesired output (number of k-points larger than requested). The number of k-points N_k^1 for RPA stated in Table I is for a hypothetical structure with one molecule in the unit

TABLE I. Precision settings and the corresponding values of parameters used for EXX and RPA calculations.

	EXX			RPA		
	`Low`	`Medium`	`High`	`Low`	`Medium`	`High`
Cut-off [eV]	440	550	660	500	500	600
`KSPACING`	0.4	0.3	0.2	–	–	–
N_k^1, No. of k-points for $Z = 1$	–	–	–	8	16	24
`PRECFOCK`	`Norm`	`Norm`	`Norm`	`Fast`	`Norm`	`Norm`

cell ($Z = 1$). The actual number of k-points for a structure with Z molecules in the unit cell, N_k^Z was obtained as $N_k^Z = N_k^1/Z$. This is to obtain comparable energies for unit cells with different Z. The plane-wave cut-off energy for response related properties (`ENCUTGW`) was set to one half of the orbital cut-off (`ENCUT`). The Green's function and response was evaluated on a grid of 8 imaginary frequencies and times for all the precision settings.[11]

The Python workflow uses the Atomistic Simulation Environment (ASE) toolkit to convert the structure format, parse energies, and perform related tasks.[15] Generally, the set up is simple, for each of the structures the different tasks (optimization, EXX, RPA) are performed in a consecutive order and each of them only after the previous finished. When the script is run, checks the results and saves a list of calculations to be run to a file on a remote cluster. This file is then read by another script on the remote machine and the jobs are submitted.

II. CONVERGENCE TESTS

Both the EXX and RPA correlation energies depend more strongly on the numerical parameters than semi-local DFT functionals. The EXX energy depends strongly on the k-point grid, for the RPA correlation the dependence on the basis-set size is also important. Fortunately, the dependence on parameters is smaller for energy differences.

We illustrate the convergence of the EXX energy with the precision settings in Fig. 1 for the molecule XXXI. For each of the 100 structures we take the EXX energies obtained using the Low, Medium, and High precision settings and set the High value as a reference, that

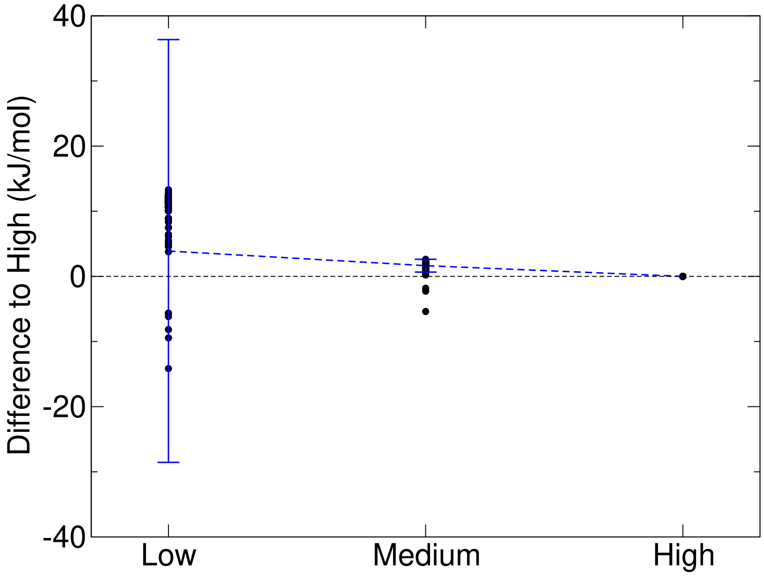

FIG. 1. Convergence of the EXX energy for the different structures of molecule XXXI using three different precision settings. For each structure the value obtained with the High parameters was the reference, i.e. it is zero. The blue dashed line shows the average values and the error bars give standard deviations. For the Low settings, there are three datapoints close to −170 kJ/mol which are outside of the graph.

is as zero. The dots in the graph then show the differences to the energy obtained with the High settings for each of the structures. The dashed blue line connects the mean values for each precision settings and the error bars show standard deviations. The standard deviation is large for the Low settings as there are three values around −170 kJ/mol outside of the graph which increase the standard deviation. There are again three values too low for the Medium settings, they correspond to different structures.

We show a difference between the RPA correlation energies of structures 1 and 12 obtained for different basis-set cut-offs and k-point settings in Fig. 2. The number of k-points for structure 12 is given on the x axis. Structure 1 has four molecules in the unit cell ($Z = 4$) and therefore uses one half of the k-points compared to the structure 12. The results show that a cut-off of 500 eV and 8 k-points lead to a difference converged to a fraction of kJ/mol. The data used in the graph used RPA obtained with the PBE functional, other tests with SCAN show similar conclusions. Finally, the data in the graph used settings `PRECFOCK=Norm`, data with `PRECFOCK=Fast` are more noisy and have a slower convergence.

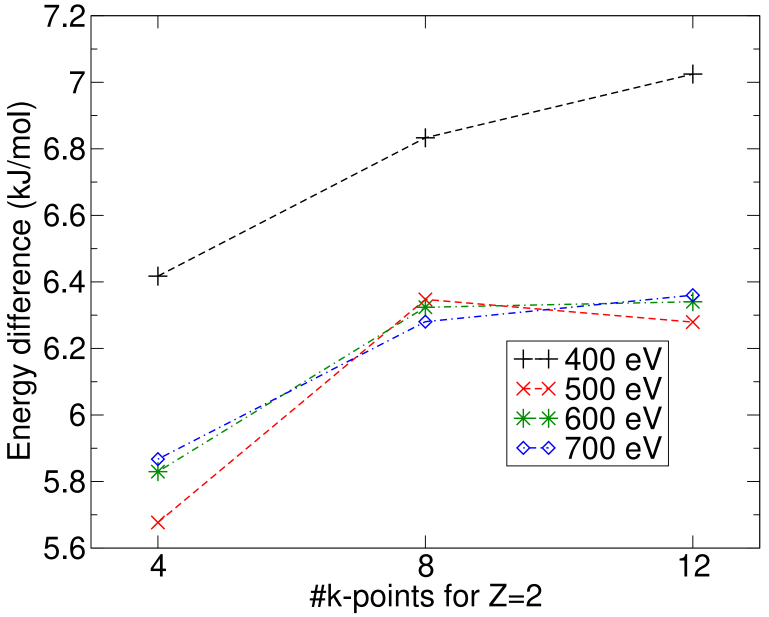

FIG. 2. Convergence of the RPA correlation energy difference between structures 1 and 12 of the molecule XXXI for different plane-wave basis-set cut-offs and number of k-points.

III. COMPUTATIONAL RESOURCES

Here we briefly comment on the computational resources needed to obtain the structures and the energies. The timings below were obtained on dual CPU nodes equipped with AMD EPYC 7351 or AMD EPYC 7302 processors and 256 or 512 GB of RAM. The most demanding step was the structural optimization, for structure 1 around 4000 CPU hours was needed to reduce the forces below 0.02 eV/Å. Similar time was needed to converge the forces to 0.001 eV/Å which would be required for frequency calculations. This is a consequence of using the vdW-dF correlation, dispersion corrections using atom-centered interactions would likely require less time. The total CPU time needed to obtain the EXX energy of structure 1 was 19, 58, and 250 CPU hours respectively for the three precision settings.

For the calculations of RPA energies not only the time but also the memory requirements become important. In Table II we list the Wall and total CPU times as well as the memory requirements of the RPA correlation energy calculations of structures 1 and 12. Note that the values should be taken as examples and not as a rigorous benchmark, as the type of CPU, number of nodes and other details apart from the listed ones differ. The CPU time needed to diagonalize the wavefunction is not listed in Table II, for each settings it is approximately

TABLE II. Time and memory requirements to obtain the RPA correlation energy for structures 1 ($Z = 4$) and 12 ($Z = 2$) of the molecule XXXI for different precision settings.

Structure	Parameter	Low	Medium	High
1	Wall time [h]	1.4	6.4	17.3
	CPU time [h]	185	820	2200
	Memory [GB]	320	670	1400
12	Wall time [h]	2.2	5.4	13.9
	CPU time [h]	70	686	1780
	Memory [GB]	192	270	590

70% of the time needed to obtain the RPA correlation energy.

IV. OPTPBE-VDW RANKING

Finally, we compare the optPBE-vdW ranking to the one obtained by RPA. The optPBE-vdW functional predicts that the structures 98 and 1 have the lowest energy, they are around 1 kJ/mol more stable than the structure 17, the most stable structure predicted by RPA. The structures 98 and 1 correspond to the polymorph A, while the experimentally more stable polymorph B is structure 25. With optPBE-vdW, structure 25 is around 4 kJ/mol less stable than structures 98 and 1. When RPA is used, the difference is reduced to around 1 kJ/mol so that the results get closer to the experimental ordering. However, with RPA the structure 98 is around 3 kJ/mol less stable than structure 17. This could be a consequence of missing terms beyond RPA or lack of free energy corrections in our polymorph ranking.

ACKNOWLEDGMENTS

This work was supported by the European Research Council (ERC) under European Union's Horizon 2020 research and innovation program (grant agreement No 759721). We are grateful for the computational resources supplied by the project "e-Infrastruktura CZ" (e-INFRA CZ LM2018140) supported by the Ministry of Education, Youth and Sports of the Czech Republic.

REFERENCES

---

[1] M. Dion, H. Rydberg, E. Schröder, D. C. Langreth, and B. I. Lundqvist, Phys. Rev. Lett. **92**, 246401 (2004).

[2] G. Román-Pérez and J. M. Soler, Phys. Rev. Lett. **103**, 096102 (2009).

[3] J. Klimeš, D. R. Bowler, and A. Michaelides, J. Phys.: Cond. Matt. **22**, 022201 (2010).

[4] J. Klimeš, D. R. Bowler, and A. Michaelides, Phys. Rev. B **83**, 195131 (2011).

[5] J. P. Perdew, K. Burke, and M. Ernzerhof, Phys. Rev. Lett. **77**, 3865 (1996), *ibid*, **78**, 1396 (1997).

[6] J. Sun, A. Ruzsinszky, and J. P. Perdew, Phys. Rev. Lett. **115**, 036402 (2015).

[7] G. Kresse and J. Hafner, Phys. Rev. B **47**, 558 (1993).

[8] G. Kresse and J. Furthmüller, Comp. Mater. Sci. **6**, 15 (1996).

[9] G. Kresse and J. Joubert, Phys. Rev. B **59**, 1758 (1999).

[10] J. Harl and G. Kresse, Phys. Rev. B **77**, 045136 (2008).

[11] M. Kaltak, J. Klimeš, and G. Kresse, J. Chem. Theory Comput. **10**, 2498 (2014).

[12] M. Kaltak, J. Klimeš, and G. Kresse, Phys. Rev. B **90**, 054115 (2014).

[13] P. E. Blöchl, Phys. Rev. B **50**, 17953 (1994).

[14] W. S. Morgan, J. J. Jorgensen, B. C. Hess, and G. L. Hart, Comput. Mater. Sci. **153**, 424 (2018).

[15] A. H. Larsen, J. J. Mortensen, J. Blomqvist, I. E. Castelli, R. Christensen, M. Duak, J. Friis, M. N. Groves, B. Hammer, C. Hargus, E. D. Hermes, P. C. Jennings, P. B. Jensen, J. Kermode, J. R. Kitchin, E. L. Kolsbjerg, J. Kubal, K. Kaasbjerg, S. Lysgaard, J. B. Maronsson, T. Maxson, T. Olsen, L. Pastewka, A. Peterson, C. Rostgaard, J. Schitz, O. Schtt, M. Strange, K. S. Thygesen, T. Vegge, L. Vilhelmsen, M. Walter, Z. Zeng, and K. W. Jacobsen, J. Phys.: Condens. Matter **29**, 273002 (2017).

12. Group 15

Anharmonic free energies with machine learning interatomic potentials

Raffaello Bianco,[1] Bruno Mladineo,[1] Antonio Parunov,[2] and Ivor Lončarić[1,*]
[1] *Ruđer Bošković Institute, 10000 Zagreb, Croatia*
[2] *Department of Physics, Faculty of Science, University of Zagreb, 10000 Zagreb, Croatia*

I. INTRODUCTION

Our goal is to perform the ranking on anharmonic Gibbs free energies with density functional theory (DFT) accuracy. To reconcile the need for many evaluations (to calculate anharmonic free energies) and the need for accuracy (at the level of DFT) we used machine-learning interatomic potential. Machine learning requires a sufficiently large database for training. To keep the overall computational effort small we used transfer learning from a large existing database of molecules to a small newly computed database of molecular crystals present in the blind test. Our methodology has three steps:

- DFT calculations of molecular crystals present in the blind test
- Transfer learning of interatomic potential
- Calculations of anharmonic free energies for each polymorph in the list

Each of these steps is described in detail below.

II. DFT CALCULATIONS

We have performed single-point DFT calculations for each structure in the CCDC-prepared list for submission 2 of the 7th CSP Blind Test. We have used the Vienna ab-initio simulation package VASP 6.3.0 [1, 2]. We used Gamma-centered k-point mesh with the smallest allowed spacing between k-points set to 0.2 $Å^{-1}$ (KSPACING = 0.2), 440 eV for the plane-wave cutoff (ENCUT = 440; PREC = Accurate), and Gaussian smearing with the width of 0.03 eV (ISMEAR = 0; SIGMA = 0.03). For the exchange-correlation functional we used R^2SCAN [3] (METAGGA = R2SCAN; LASPH = .TRUE.; LMIXTAU = .TRUE.). Selfconsistency was achieved if the total energy and eigenvalues changed less than 10^{-6} eV in two consecutive steps (EDIFF = 1E-6). All other parameters were kept default.

In any converged DFT calculation, the most important choice that determines accuracy is the exchange-correlation functional. Here, there is a complication that the existing database of molecules that was used for machine learning was computed using ωB97X functional [4] that is not feasible to be used on crystals. Due to the associated computational cost, we wanted to avoid hybrid functionals. Recently proposed R^2SCAN functional is computationally affordable, numerically stable, and accurate for a wide range of properties, including molecular crystals for which it can be also coupled with dispersion corrections [3, 5, 6].

Since the CCDC-prepared list was not optimized by this functional, single-point DFT calculations result in a range of energies, forces and stresses that can be used for machine learning. DFT calculations accounted for more than 80% of the total computational cost in our submission.

III. MACHINE LEARNING OF POTENTIALS

Machine-learning interatomic potentials promise to provide the accuracy of the underlying method (usually DFT) and speed comparable to conventional force fields [7]. Thus, they seem like an ideal choice for crystal structure prediction where many evaluations of accurate potential energy surface are needed. The downside is that a training database that spans the configurational space is needed which is again computationally expensive. To avoid this step, we have used an existing large database that explores the configurational space of small molecules and corresponding neural network potential, ANI-2x [8].

* ivor.loncaric@gmail.com

Since ANI-2x was trained on relatively few non-bonded structures and was not trained on structures containing Cu, I, or Si atoms it would not work out-of-box. Still, it is expected that most of the chemical bonding is well described. Therefore, we have performed transfer learning using the above-described DFT data. Starting from the original ANI-2x weights we retrained the model on new DFT data of energies and forces. New DFT data was split to keep 20% of the data for validation. Since Cu, I, or Si atom types are not present in the original ANI-2x model we have used weights of the model for a different atom type. When retraining for target XXVII we used weights of C to initialize the model for Si and weights of Cl to initialize the model for I. When retraining for target XXVIII we used weights of S to initialize the model for Cu. All descriptors and other model parameters were used as in the original ANI-2x. Transfer learning was performed in the torchani code [9]. Since the additional DFT data is relatively small, the retraining on one Nvidia A100 GPU took only up to a few hours.

Since we have used both energies and forces for transfer learning, we have tested the weight of forces and energy loss functions in the total loss function. We had access to only 500 or 100 energy points per target structure and forces on each atom in all of these structures. Results are presented in Table I. Based on these results, we have used 90% of the forces loss function and 10% of the energy loss function for the loss function in retraining.

TABLE I. Weight λ in the loss function $L = \lambda \times \text{MSE(forces)} + (1 - \lambda) \times \text{MSE(energies)}$. Data is for target XXXII.

λ	RMSE forces [eV/ Å]	RMSE energy/atom [eV]
0	0.90252	0.00092
0.1	0.06967	0.00148
0.5	0.06804	0.00158
0.9	0.06730	0.00159
1	0.07065	0.26778

We have checked the influence of database size on validation errors. As shown in Table II, already with 100 structures we can obtain reasonably accurate results. Finally, we have also checked the benefits of transfer learning compared to the original ANI-2x model, as well as training only on the new DFT data. As shown in Table III transfer learning significantly improves the model compared to a model trained only on the new data. Due to different exchange-correlation functional, original ANI-2x and new DFT data cannot be directly compared. Retraining all neural network layers seems to work better than retraining only parts of the network. Transfer learning accuracy can be also seen in Fig. 1 for particularly challenging system XXXIII. Final validation errors for each target system are listed in Table IV.

TABLE II. Validation error as a function of the number of DFT structures considered in the training set. Data is for target XXXII.

Number of structures	RMSE forces [eV/ Å]	RMSE energy/atom [eV]
100	0.07857	0.00163
200	0.07548	0.00161
300	0.07002	0.00160
400	0.06730	0.00159

TABLE III. Validation errors on validation set of new DFT data of different models for target system XXXII.

Type of learning	RMSE forces [eV/ Å]	RMSE energy/atom [eV]
Only new DFT data	0.27907	0.00295
Original ANI2x	0.42062	0.25930
All layers retrained	0.06730	0.00159
First and last layer frozen	0.07823	0.00167

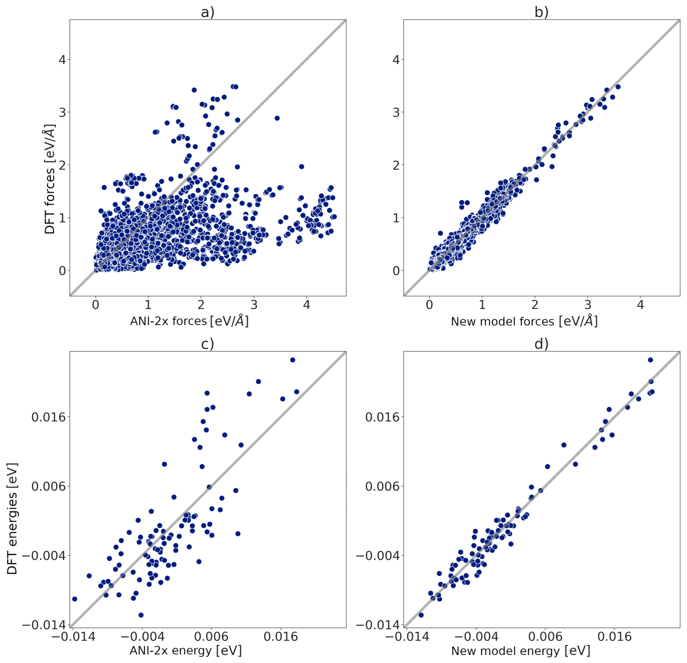

FIG. 1. Validation errors for ANI-2x (a) forces and c) energies) and retrained model (b) forces and d) energies) on new DFT data for target system XXXIII.

TABLE IV. Validation errors (root-mean-square errors) for each target system for final transfer learning models.

Target system	Energy RMSE [eV]	Force RMSE [eV/ Å]
XXVII	0.00316	0.06887
XXVIII	0.00120	0.06153
XXXI	0.00130	0.08117
XXXII	0.00159	0.06730
XXXIII	0.00149	0.09242

IV. ANHARMONIC FREE ENERGIES

The structural relaxation and the estimation of the free energy of the crystals were performed using the Stochastic Self-Consistent Harmonic Approximation (SSCHA) method [10–13], using the code available at `http://sscha.eu/`. The SSCHA is a mean-field method, based on the Gibbs-Bogoliubov free-energy variational principle [14], that allows to incorporate, within the Born-Oppenheimer (BO) approximation, quantum and anharmonic effects in the nuclei dynamics at non-perturbative level. The SSCHA is primarily devised to compute Helmotz free energy $F = U - TS$ of systems (where U is the internal energy, T the temperature, and S the entropy), by minimizing the system-dependent free energy functional

$$\mathcal{F}(\boldsymbol{\mathcal{R}},\boldsymbol{\Phi}) = \langle K + V(\boldsymbol{R})\rangle_{\tilde{\rho}_{\boldsymbol{\mathcal{R}},\boldsymbol{\Phi}}} - Tk_B \left\langle \ln \tilde{\rho}_{\boldsymbol{\mathcal{R}},\boldsymbol{\Phi}}\right\rangle_{\tilde{\rho}_{\boldsymbol{\mathcal{R}},\boldsymbol{\Phi}}} , \tag{1}$$

where K is the kinetic operator, $\boldsymbol{R}$ is a collective nuclear coordinate, $V(\boldsymbol{R})$ is the interatomic potential energy surface (PES), $\tilde{\rho}_{\boldsymbol{\mathcal{R}},\boldsymbol{\Phi}}$ is the nuclear density matrix of an auxiliary harmonic potential parametrized with average nuclear positions $\boldsymbol{\mathcal{R}}$ and effective force constants $\boldsymbol{\Phi}$ (related to the amplitude of the ionic fluctuations around $\boldsymbol{\mathcal{R}}$), and $\langle\square\rangle_{\tilde{\rho}_{\boldsymbol{\mathcal{R}},\boldsymbol{\Phi}}}$ indicates the quantum average of the operator $\square$ computed with $\tilde{\rho}_{\boldsymbol{\mathcal{R}},\boldsymbol{\Phi}}$. At the end of the minimization procedure, performed with respect to the free parameters $\boldsymbol{\mathcal{R}}$ and $\boldsymbol{\Phi}$, the SSCHA gives an estimation of the free energy, $F \simeq \mathcal{F}[\boldsymbol{\mathcal{R}}_{\rm eq},\boldsymbol{\Phi}_{\rm eq}]$, together with the equilibrium auxiliary force constant matrix $\boldsymbol{\Phi}_{\rm eq}$, and the estimated equilibrium atomic configuration at fixed cell $\boldsymbol{\mathcal{R}}_{\rm eq}$. The SSCHA method is based on the fact that $\mathcal{F}(\boldsymbol{\mathcal{R}},\boldsymbol{\Phi})$, and its gradient w.r.t $\boldsymbol{\mathcal{R}}$ and $\boldsymbol{\Phi}$, can be written as averages w.r.t the Gaussian probability density $\tilde{\rho}_{\boldsymbol{\mathcal{R}},\boldsymbol{\Phi}}(\boldsymbol{R}) = \langle \boldsymbol{R} \,|\, \tilde{\rho}_{\boldsymbol{\mathcal{R}},\boldsymbol{\Phi}} \,|\, \boldsymbol{R}\rangle$ of certain functions of the potential energy $V(\boldsymbol{R})$ and of the standard "static" classical BO forces $\overset{(\rm BO)}{\mathbf{f}}(\boldsymbol{R}) = -\partial V/\partial \boldsymbol{R}$ acting

on the nuclei when they are in the configuration $\boldsymbol{R}$ (notice that the BO forces differ from the forces computed with $\mathbf{f}(\boldsymbol{\mathcal{R}}) = -\partial\mathcal{F}/\partial\boldsymbol{\mathcal{R}}$, which include effects from both quantum and thermal nuclear fluctuations). The SSCHA code adopts a supercell-based stochastic Monte Carlo approach to compute these averages: a population with a certain number $\mathcal{N}$ of configurations $\boldsymbol{R}_{\mathcal{I}}$ (enough to reach converged results) is generated in a supercell (large enough to reach the thermodynamic limit) according to the probability distribution $\tilde{\rho}_{\boldsymbol{\mathcal{R}},\boldsymbol{\Phi}}(\boldsymbol{R})$ and, for each element $\boldsymbol{R}_{\mathcal{I}}$ of the population, energy $V(\boldsymbol{R}_{\mathcal{I}})$ and BO forces $\overset{(\mathrm{BO})}{\mathbf{f}}(\boldsymbol{R}_{\mathcal{I}})$ are computed using any external energy-force engine. This allows us to estimate the averages with a finite sampling, the result being exact in the $\mathcal{N} \to +\infty$ limit. In particular, the knowledge of the gradients $\nabla_{\boldsymbol{\mathcal{R}}}\mathcal{F}$ and $\nabla_{\boldsymbol{\Phi}}\mathcal{F}$ is used to minimize the function $\mathcal{F}(\boldsymbol{\mathcal{R}},\boldsymbol{\Phi})$ using a preconditioned gradient-descent approach.

The SSCHA minimization started with an initial guess on $\boldsymbol{\mathcal{R}}$ and $\boldsymbol{\Phi}$, given by the machine learning potential equilibrium nuclear configuration and the harmonic force constants, respectively. Relaxation and harmonic phonon calculation using machine learning potential was performed with the Atomic Simulation Environment (ASE) [15]. Then we proceeded as follows:

1. The trial Gaussian probability distribution function $\tilde{\rho}_{\boldsymbol{\mathcal{R}},\boldsymbol{\Phi}}(\boldsymbol{R})$ was used to generate a population of $\mathcal{N}$ nuclear configurations $\boldsymbol{R}_{\mathcal{I}}$ in a supercell.

2. For each nuclear configuration $\boldsymbol{R}_{\mathcal{I}}$ in the population, total energies $V(\boldsymbol{R}_{\mathcal{I}})$ and forces $\overset{(\mathrm{BO})}{\mathbf{f}}(\boldsymbol{R}_{\mathcal{I}})$ were computed.

3. The total energy and forces computed for the population's elements were used to compute the free energy function $\mathcal{F}(\boldsymbol{\mathcal{R}},\boldsymbol{\Phi})$ and its derivatives $\nabla_{\boldsymbol{\mathcal{R}}}\mathcal{F}$ and $\nabla_{\boldsymbol{\Phi}}\mathcal{F}$ with respect to the free parameters $\boldsymbol{\mathcal{R}}$ and $\boldsymbol{\Phi}$, respectively.

4. $\boldsymbol{\mathcal{R}}$ and $\boldsymbol{\Phi}$ were updated to minimize the free energy.

These steps were repeated until the minimum of the free energy was found. Using an importance sampling technique, the averages for several updated $\tilde{\rho}_{\boldsymbol{\mathcal{R}},\boldsymbol{\Phi}}(\boldsymbol{R})$ were computed using the same population until the statistics were good enough (i.e. as long as the Kong-Liu parameter was not smaller than 0.5). This allowed us to minimize the number of calls to the energy-force engine, this being the bottleneck of the algorithm. At the end of the SSCHA optimization, apart from the temperature-dependent internal positions $\boldsymbol{\mathcal{R}}_{\mathrm{eq}}$, and the equilibrium auxiliary force constant matrix $\boldsymbol{\Phi}_{\mathrm{eq}}$, the code allowed to calculate the anharmonic stress tensor $\boldsymbol{P} = -\frac{1}{\Omega}\partial\mathcal{F}/\partial\boldsymbol{\varepsilon}|_{\boldsymbol{\varepsilon}=0}$ too, where Ω is the simulation-box volume and $\boldsymbol{\varepsilon}$ is the strain tensor. The anharmonic stress tensor $\boldsymbol{P}$, at variance with the standard BO stress tensor $\overset{(\mathrm{BO})}{\boldsymbol{P}} = -\frac{1}{\Omega}\partial V/\partial\boldsymbol{\varepsilon}|_{\boldsymbol{\varepsilon}=0}$, includes effects from both quantum and thermal nuclear fluctuation. As for the free energy function $\mathcal{F}(\boldsymbol{\mathcal{R}},\boldsymbol{\Phi})$ and its gradients, SSCHA computes $\boldsymbol{P}$ using a stochastic approach, evaluating the average of a function of the $\boldsymbol{R}$-dependent BO forces $\overset{(\mathrm{BO})}{\mathbf{f}}(\boldsymbol{R})$ and stress tensors $\overset{(\mathrm{BO})}{\boldsymbol{P}}(\boldsymbol{R})$ (in fact, the employed energy-force engine were able to compute, for a generic nuclear configuration $\boldsymbol{R}$, the BO stress tensor too). Minimizing the Gibbs free energy $G = U - TS + PV$, where $P = -\frac{1}{3}\mathrm{Tr}\boldsymbol{P}$ is the pressure, the SSCHA code allowed to fully relax the crystalline structure accounting for quantum and thermal nuclear fluctuations for both internal and lattice degrees of freedom at the fixed target pressure.

The simulations were performed at $T = 300$ K, and the structures were optimized with zero target pressure $P = 0$. In order to compute the stochastic averages and perform the minimization, first (up to) 10 populations of 500 elements each and, subsequently, in order to refine the results, other (up to) 20 populations with 1000 elements each were generated. The supercells used for the calculations were at least 10.2 Angstrom along the three lattice directions, so as to cover the range of the used machine-learning potential $V(\boldsymbol{R})$.

V. ACKNOWLEDGEMENTS

This work has been supported in part by Croatian Science Foundation under the project UIP-2020–02-5675.

---

[1] G. Kresse and J. Furthmüller, Efficient iterative schemes for ab initio total-energy calculations using a plane-wave basis set, Physical Review B **54**, 11169 (1996).

[2] G. Kresse and D. Joubert, From ultrasoft pseudopotentials to the projector augmented-wave method, Physical Review B **59**, 1758 (1999).

[3] J. W. Furness, A. D. Kaplan, J. Ning, J. P. Perdew, and J. Sun, Accurate and numerically efficient r2scan meta-generalized gradient approximation, The Journal of Physical Chemistry Letters **11**, 8208 (2020).

[4] J.-D. Chai and M. Head-Gordon, Systematic optimization of long-range corrected hybrid density functionals, The Journal of Chemical Physics **128**, 084106 (2008).
[5] S. Grimme, A. Hansen, S. Ehlert, and J.-M. Mewes, r2scan-3c: A "swiss army knife" composite electronic-structure method, The Journal of Chemical Physics **154**, 064103 (2021).
[6] S. Ehlert, U. Huniar, J. Ning, J. W. Furness, J. Sun, A. D. Kaplan, J. P. Perdew, and J. G. Brandenburg, r2scan-d4: Dispersion corrected meta-generalized gradient approximation for general chemical applications, The Journal of Chemical Physics **154**, 061101 (2021).
[7] O. T. Unke, S. Chmiela, H. E. Sauceda, M. Gastegger, I. Poltavsky, K. T. Schütt, A. Tkatchenko, and K.-R. Müller, Machine learning force fields, Chemical Reviews **121**, 10142 (2021).
[8] C. Devereux, J. S. Smith, K. K. Huddleston, K. Barros, R. Zubatyuk, O. Isayev, and A. E. Roitberg, Extending the applicability of the ani deep learning molecular potential to sulfur and halogens, Journal of Chemical Theory and Computation **16**, 4192 (2020).
[9] X. Gao, F. Ramezanghorbani, O. Isayev, J. S. Smith, and A. E. Roitberg, Torchani: A free and open source pytorch-based deep learning implementation of the ani neural network potentials, Journal of Chemical Information and Modeling **60**, 3408 (2020).
[10] I. Errea, M. Calandra, and F. Mauri, Anharmonic free energies and phonon dispersions from the stochastic self-consistent harmonic approximation: Application to platinum and palladium hydrides, Phys. Rev. B **89**, 064302 (2014).
[11] R. Bianco, I. Errea, L. Paulatto, M. Calandra, and F. Mauri, Second-order structural phase transitions, free energy curvature, and temperature-dependent anharmonic phonons in the self-consistent harmonic approximation: Theory and stochastic implementation, Phys. Rev. B **96**, 014111 (2017).
[12] L. Monacelli, I. Errea, M. Calandra, and F. Mauri, Pressure and stress tensor of complex anharmonic crystals within the stochastic self-consistent harmonic approximation, Phys. Rev. B **98**, 024106 (2018).
[13] L. Monacelli, R. Bianco, M. Cherubini, M. Calandra, I. Errea, and F. Mauri, The stochastic self-consistent harmonic approximation: calculating vibrational properties of materials with full quantum and anharmonic effects, Journal of Physics: Condensed Matter **33**, 363001 (2021).
[14] G. Q. Huang, L. F. Chen, M. Liu, and D. Y. Xing, Electronic structure and electron-phonon interaction in the ternary silicides $malsi$ ($m = ca$, sr, and ba), Phys. Rev. B **69**, 064509 (2004).
[15] A. H. Larsen, J. J. Mortensen, J. Blomqvist, I. E. Castelli, R. Christensen, M. Dułak, J. Friis, M. N. Groves, B. Hammer, C. Hargus, E. D. Hermes, P. C. Jennings, P. B. Jensen, J. Kermode, J. R. Kitchin, E. L. Kolsbjerg, J. Kubal, K. Kaasbjerg, S. Lysgaard, J. B. Maronsson, T. Maxson, T. Olsen, L. Pastewka, A. Peterson, C. Rostgaard, J. Schiøtz, O. Schütt, M. Strange, K. S. Thygesen, T. Vegge, L. Vilhelmsen, M. Walter, Z. Zeng, and K. W. Jacobsen, The atomic simulation environment—a python library for working with atoms, Journal of Physics: Condensed Matter **29**, 273002 (2017).

13. Group 16

Supplementary Information

Dylan M. Anstine[1], Dana O'Connor[2], Roman Zubatyuk[1], Rithwik Tom[3], Imanuel Bier[2], Kamal S. Nayal[1], Yi Yang[2], Wenda Deng[2], Kehan Tang[2], Olexandr Isayev[1], and Noa Marom[1,2,3]

[1] Department of Chemistry, Carnegie Mellon University, Pittsburgh, Pennsylvania 15213, USA
[2] Department of Materials Science and Engineering, Carnegie Mellon University, Pittsburgh, Pennsylvania 15213, USA
[3] Department of Physics, Carnegie Mellon University, Pittsburgh, Pennsylvania 15213, USA

1 Overview of Methodology

Ranking the relative stability of polymorph forms of targets XXVII, XXXI, XXXII, and XXXIII was performed according to the calculated Gibbs free energy (G) of the putative crystal structures using the quasi-harmonic approximation (QHA). From a high-level perspective, QHA functions as a series of harmonic approximations (HA) performed at several different volumes to extract physical information about thermally induced volume changes. For each structure, we employed calculations of Helmholtz free energy (F) at 0 K and 300 K using the harmonic approximation for a series of structures with volume changes induced by applying external isotropic pressure. We have selected the minimum at F-V curve as the Gibbs free energy. In order to speed up the calculations of the energies, atomic forces, and force constants for the crystal structures, we employed AIMNet neural network potentials trained to emulate density functional theory (DFT) calculations using the Perdew-Burke-Ernzerhof functional (PBE) with the Grimme D4 dispersion correction and def2-TZVPP basis sets.[1,2] The QHA thermal corrections were calculated using an in-house interface between phonopy[3] and AIMNet.

The set of AIMNet neural network potential [4,5] models used to calculate crystal structure energies, atomic forces, and force constants were trained specifically to each target. Briefly, the AIMNet model belongs to the class of atomistic neural network potentials.[6] It is capable of predicting rotationally and translationally invariant properties of the atoms, such as per-atom energy contribution to the total energy of the system based on the geometry of the local atomic environment. It has been demonstrated that potential models of this class are extensible[7–10], *e.g.*, could be trained on a relatively small molecular configurations and provide accurate predictions when applied to the systems of much larger size. We exploited this feature by training the AIMNet model on the clusters of the molecules extracted from periodic crystal structures. In this way, we avoided performing expensive periodic calculations to collect training data. More information about the AIMNet architecture is available upon request or in the publication of the original model.[4]

2 Computational Details

In this work, our a developmental version of the AIMNet2 model was trained to reproduce DFT-calculated molecular properties: molecular energy, atomic forces, and atom-centered partial charges calculated using Hirshfeld partitioning. Training data for the target specific AIMNet models were based on molecular clusters extracted from the crystal structures, which contained

the reference molecule and up to 10 of its neighbors. We performed additional sampling to capture of out-of-equilibrium conformations for the clusters with short constrained molecular dynamics simulations using the GFNFF force field[11] at 600 K with tethering spring forces applied to atomic positions using force constants of 0.005 eV/Å^2. We found in our initial model testing that training was more stable and efficient by pre-training the AIMNet model with GFN2-xTB[12] computed data, which included large clusters, and then performing transfer learning to DFT data for smaller clusters containing up to 3 molecules. For each target the AIMNet model was trained with approximately 4 x 10^5 GFN2 data points and approximately 2 x 10^4 of DFT data. The DFT calculations were performed with ORCA 5.0.3 software[13,14] at the PBE-D4/def2-TZVPP level of theory. The molecular energies, atomic forces and Hirshfeld partial charges were collected, which were used as targets in model training. When applied to crystal structures, the AIMNet model accounts for long-range interaction with Ewald sum approximation[15] to Coulomb energy of the crystal, and pairwise C_6 and C_8 dispersion energy terms. The many-body dispersion terms were calculated based on the Axilrod–Teller–Muto formula with DFT-D4 software[2]. All calculations with the AIMNet model were performed with an in-house code implemented using the PyTorch framework[16].

For each crystal structure we performed geometry optimization with RMS atomic force convergence criterion of 0.001 eV/Å. During optimization dispersion interactions were accounted for using only two-body terms. The three-body energy term was added for the final crystal geometry. Following the initial optimization with no applied external pressure, a series of 16 fixed-volume optimizations were performed with external isotropic pressure applied to change the unit cell volume in the range of -5% to +15%. For each of these 16 points, thermochemistry analysis using the HA was performed with AIMNet potential to obtain the Helmholtz free energies at 0 K and 300 K for each crystal structure. The lowest Helmholtz free energy with respect to unit cell volume for a target structure was considered to be the Gibbs free energy and used in the final crystal structure ranking for all targets.

3 Post Hoc Analysis

To evaluate the performance of the AIMNet model we ran post-hoc DFT calculations for the experimental forms of Targets XXVII, XXXI, XXXII, and XXXIII. We performed dispersion-inclusive density functional theory (DFT) calculations for structure optimizations, rankings, zero-point, and thermal energy corrections with the FHI-aims code.[17] Previous work has shown that the DFT lattice energy alone often lacks sufficient accuracy for ranking organic molecular crystals;[18–21] as a result, we also calculated zero-point energy (ZPE) and thermal corrections to obtain the sublimation enthalpy for each of the structures in accordance with

$$\Delta H_{sub}(T) = -E_{latt} - E_{ZPE} - \int_0^T C_P dT \qquad (1)$$

where $\Delta H_{sub}(T)$ is the sublimation enthalpy at a temperature T, E_{latt} is the lattice energy, E_{ZPE} is the zero-point energy, and $\int_0^T C_P dT$ is the integral from 0 to T of the isobaric heat capacity integrated with respect to the temperature.[18] It is important to note that Equation 1 only accounts for the solid-state contributions to the sublimation enthalpy because of the imposed assumption that all crystal structures for a target molecule are constructed with the same conformation.

Equation 1 is typically simplified under the rigid-rotor ideal gas harmonic approximation, which assumes that molecular crystals undergo little to no thermal expansion.[18,20,22] However previous work has shown that thermal expansion can have significant contributions for crystal structure ranking in some classes of materials, which motivated us to treat thermal expansion explicitly (as was done with the AIMNet rankings).[23,24]

Post analysis quantum mechanical (QM) calculations were performed with dispersion-inclusive DFT using the FHI-aims electronic structure software. The k-grid was defined such that n×a <= 25 where a is the lattice vector and n is the k-grid setting to sample the Brillouin zone.[7] Here, we define lower-level and higher-level settings for FHI-aims.[17] Lower-level settings correspond to a light basis set with tier 1 settings while higher-level settings correspond to a tight basis set with tier 2 settings. All structures were first optimized with DFT. Initial optimizations were performed with the generalized gradient approximation of Perdew, Burke, and Ernzerhof (PBE)[1] paired with the many-body dispersion (MBD)[25] correction (PBE+MBD) with lower-level settings. Structures were then re-optimized using PBE+MBD with higher-level settings. This sequential procedure was carried out to make more efficient use computational resources. Structures were then re-ranked according to E_{latt} single point energy calculations using the PBE-based hybrid functional, PBE0,[26] paired with the MBD correction using lower-level settings with geometries optimized with PBE+MBD.

ZPE and vibrational contributions were initially calculated via the harmonic approximation (HA) using phonopy, which is an open-source python package with FHI-aims interface.[3] Phonopy calculates the requisite force constants for the HA approximation using a finite displacement method. For phonon calculations, we defined supercells such that extended at least 10 Å in each cartesian direction, which we found in previous testing to be an adequate criterion for limiting force calculation bias.[19,21,24] Displacements (0.005 Å) were performed on the optimized crystal geometries used in the PBE0-MBD single point energy calculation. It is worth noting that our previous work indicates that geometries optimized with lower-level and higher-level settings in FHI-aims were not significantly different, thus, the selection of settings for the initial crystal structure optimization is expected to have a minimal impact on our use of the HA.[21] A similar statement of impact and justification is made for the sensitivity of ZPE values as a function of DFT settings, motivating us to select light settings.[24] The forces of the displaced atoms are calculated in a single point energy calculation with PBE+MBD using lower-level settings.

For all the targets, the experimental forms were reoptimized with PBE+MBD using *lower-level* settings and PBE+MBD using *higher-level* settings. Single point energy calculations were then performed with PBE0+MBD using *lower-level* settings using the geometries optimized with the *lower-level* PBE+MBD settings. For Targets XXXI, XXXII, and XXXIII the ZPE contributions for each experimental form was calculated using the HA with PBE+MBD using *lower-level* settings with the geometries optimized using PBE+MBD at *lower-level* settings. For final post analysis rankings at 300 K, the Kirchoff term is calculated within the QHA with PBE+MBD using *lower-level* settings with geometries optimized with PBE+MBD using *lower-level* settings. Because of ongoing analysis with Target XXVII, we did not perform additional HA and QHA calculations. These post-hoc DFT calculations were used to compare root-mean-squared

deviation (RMSD) values and volumes across ML and DFT methods as well as stability rankings.

Thermal corrections applied in our DFT post analysis were calculated using QHA using phonopy's FHI-aims interface.[3] To obtain different volumes required by QHA, we DFT optimized structures under positive (contraction) and negative (expansion) external pressures: -0.6, -0.4, -0.2, 0.0, 0.2, and 0.4 GPa, in accordance with our previous work.[19,24] For each crystal structure volume, the HA was performed using settings consistent with those described previously. The Gibbs free energy is then extracted as the minimum of the Helmholtz free energy (F) as a function of crystal structure volume (V) at each temperature. To extract the minima, the Birch-Murnaghan equation of state was used to fit the F-V curve at each temperature. Once the Gibbs free energy is obtained the constant pressure heat capacity C_p was calculated[3] according to

$$C_p(T,p) = -T\frac{d^2G(T,p)}{dT^2} \qquad (2)$$

Equation 2 is used in the calculation of the total sublimation enthalpy (Eq. 1).

3.1 Target XXVII

For Target XXVII, one experimental form was given, the A form (structure 28). Figure 1 shows the volume correlation plot for structures optimized with AIMNet and PBE+MBD using *lower-level* settings. As can be seen, AIMNet tends to overestimate in comparison to PBE+MBD. Because AIMNet uses a pairwise dispersion correction, which tends to overestimate the intermolecular interactions, it is expected that PBE+MBD would yield larger volumes than AIMNet.[21,24]

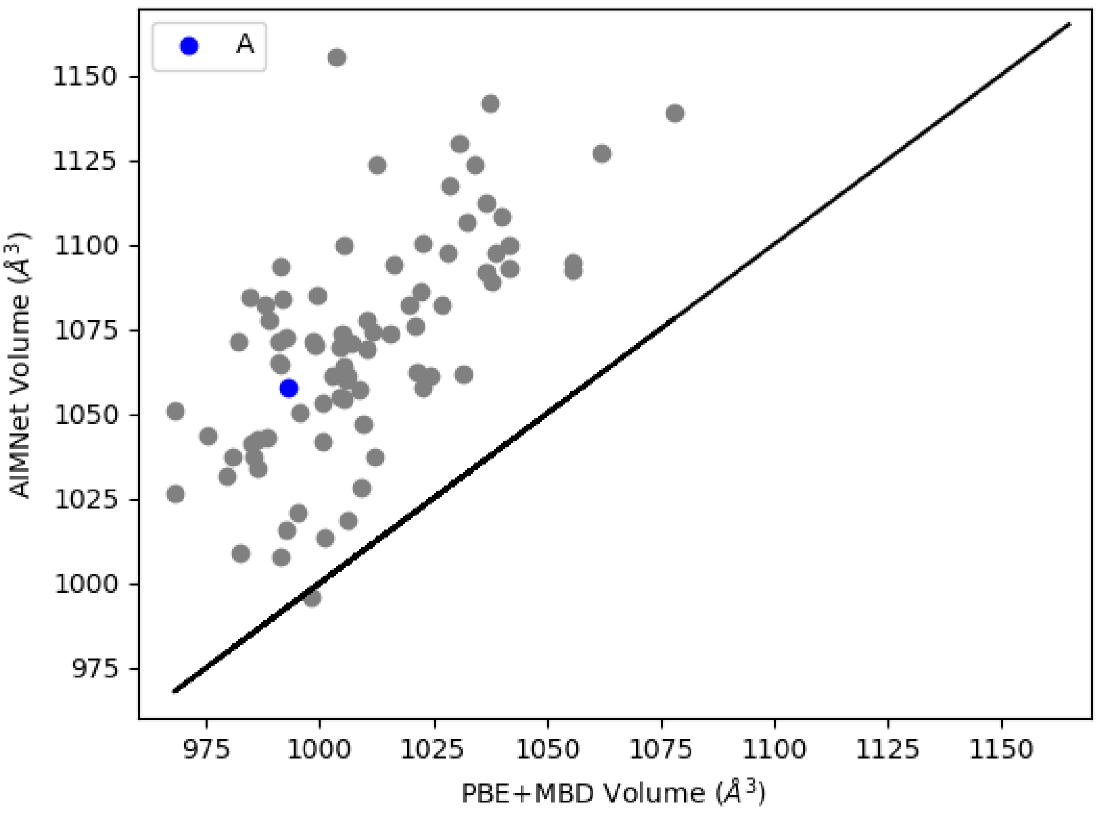

Figure 1: Volume correlation plot for target XXVII crystal structures between AIMNet trained on N-mers PBE/def2-mTZVPP with D4 dispersion corrections and those optimized with DFT PBE+MBD using *lower-level* settings.

3.2 Target XXXI

For Target XXXI, four experimental forms were given: A major (structure 98), A minor (structure 1), B (structure 25), and C (structure 89). First, we compared root mean square displacement (RMSD) for the experimental structures optimized three different methods: PBE+MBD with *lower-level* settings, PBE+MBD with *higher-level* settings, and AIMNet as implemented in pymatgen.[27] The results are shown in Table 1. As can be seen, PBE+MBD with *higher level* settings results in the lowest RMSD values, followed by PBE+MBD with *lower-level* settings, followed by AIMNet. The solvent stabilized form C had the largest RMSD values across all methods.

Table 1: Summary of root mean squared deviations (RMSD) between different methods in comparison to the 4 experimentally characterized ground truths for target XXXI.

Target XXXI			
**A Major Structure**		**B Structure**	
Method	RMSD (Å)	Method	RMSD (Å)
PBE+MBD, lower level	0.065	PBE+MBD, lower level	0.056
PBE+MBD, higher level	0.057	PBE+MBD, higher level	0.055
AIMNet	0.104	AIMNet	0.175
**A Minor Structure**		**C Structure**	
Method	RMSD (Å)	Method	RMSD (Å)
PBE+MBD, lower level	0.079	PBE+MBD, lower level	0.111
PBE+MBD, higher level	0.076	PBE+MBD, higher level	0.111
AIMNet	0.093	AIMNet	0.111

Figure 2 shows the volume correlation plot between structures optimized with AIMNet and PBE+MBD using *lower-level* settings. The experimental forms are colored. As can be seen, AIMNet tends to overestimate the volume in comparison to PBE+MBD for this target. PBE+MBD at 0 K is expected to underestimate unit cell volumes since molecular crystals tend to expand with temperature.[19] With this in mind, we also examined the behavior of the experimental forms when subjected to explicit treatment of thermal expansion. Figure 3 shows the percent error for three of the experimental forms of Target XXXI with four different volume treatments: the electronic volume V_{el} calculated with DFT at 0 K, V_{ZPVE} calculated within the QHA at 0 K, V_{RT} calculated within the QHA at 300 K, and the volume calculated with AIMNet V_{AIMNet}. The C form is not shown for clarity as all DFT methods severely underestimated the volume. As can be seen, V_{el} is the most accurate volumes for XXXI structure 1 and XXXI structure 98 while V_{ZPVE} is the most accurate for XXXI structure 25. For all of the structures PBE+MBD underestimated, which is expected given that molecular crystals expand with increased temperature.[19] Adding thermal effects overestimated the volume at room temperature, which is in agreement with previous work.[21,24]

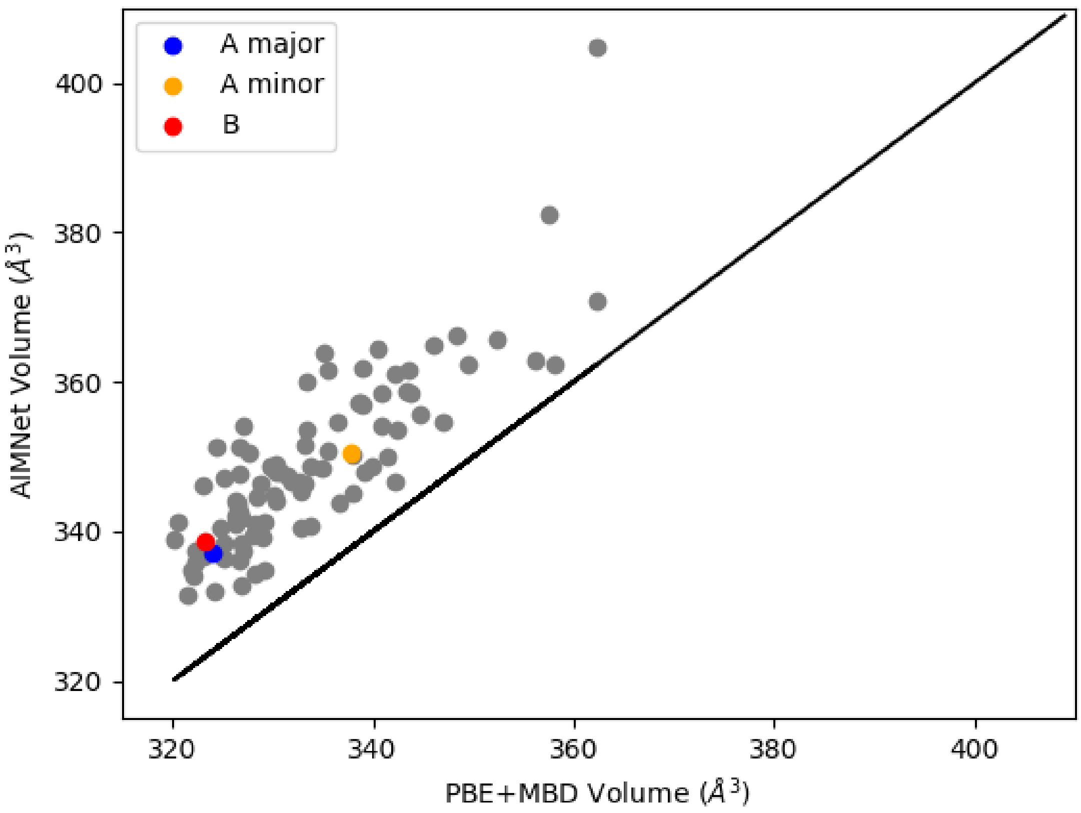

Figure 2: Volume parity plot for target XXXI crystal structures between AIMNet trained on N-mers PBE/def2-mTZVPP with D4 dispersion corrections and those optimized with DFT PBE+MBD.

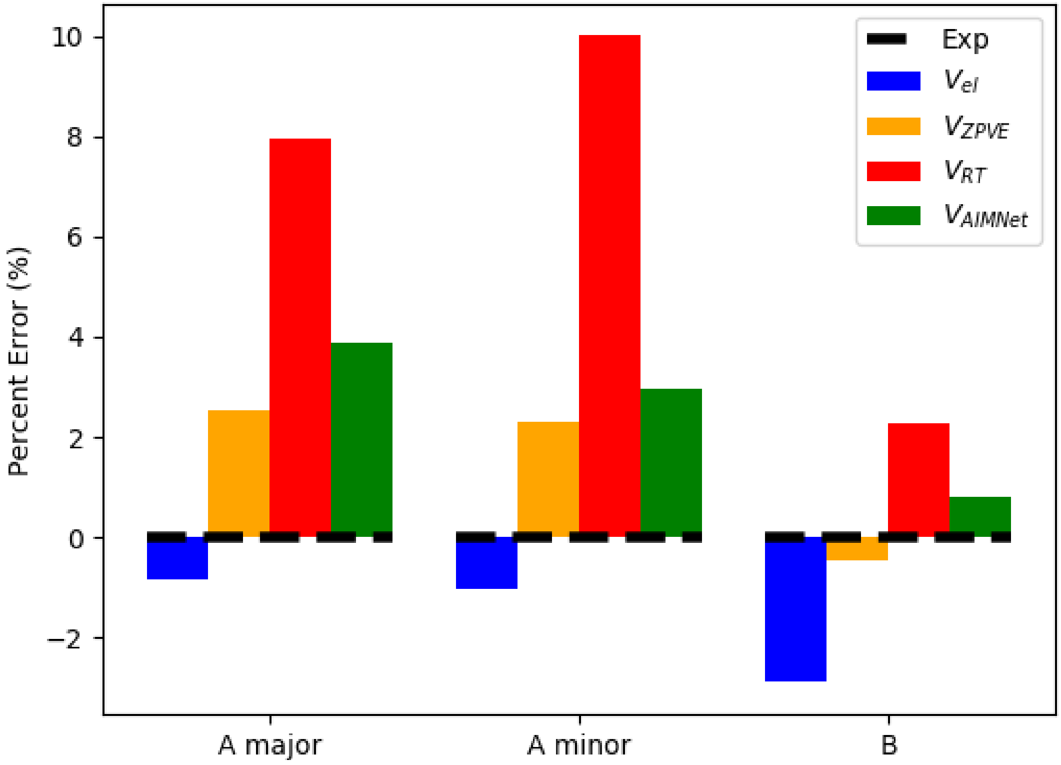

Figure 3: Comparison of volumes calculated with different DFT and ML methods. V_{el} refers to the electronic volume calculated at 0 K with PBE+MBD using *lower-level* settings, V_{ZPVE} refers to the volume including zero-point vibrational effects calculated with PBE+MBD, V_{RT} refers to the volume at room temperature calculated within the quasi-harmonic approximation with PBE+MBD, and V_{AIMNet} refers to the volume calculated with AIMNet at 0 K.

With this in mind, we examined the role of thermal expansion for this target. Previous work has shown that the importance of thermal expansion can be system dependent.[23,24] Figure 4 shows the expansion from V_{el} to V_{ZPVE} to V_{RT} for the experimental forms of Target XXXI. Expansion between V_e to V_{ZPVE} represents expansion due to vibrational effects and expansion between V_{ZPVE} to V_{RT} represents expansion due to thermal effects. In their analysis, Heit and Beran found that vibrational effects were comparable to thermal effects while we found in our previous work that thermal effects were more dominant for energetic materials.[23,24] As can be seen, for these structures thermal effects were more dominant, especially in the A minor form. Additionally, Hoja *et al.* found that their benchmark set of 23 molecular crystals, the X23 set, expanded by roughly 5%. Meanwhile, the structures below expanded by 8.87%, 11.1%, 5.31%, and 7.06% for the A minor, A major, B, and C forms, respectively. Hence explicit treatment of thermal expansion within the QHA is crucial for this target.

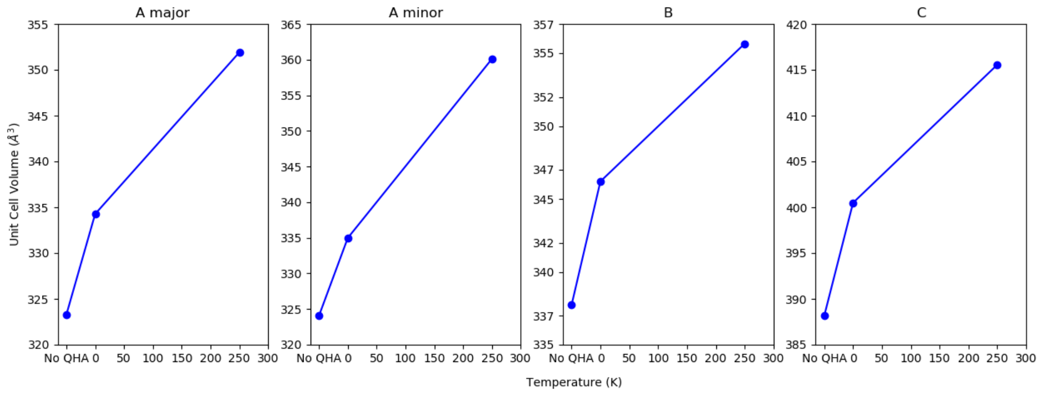

Figure 4: Unit cell volume expansion from V_{el} to V_{ZPVE} to V_{RT} for the experimental forms of Target XXXI.

Figure 5 shows the rankings of the four experimental forms of Target XXXI with different DFT and ML methods. A major is shown in blue, A minor is shown in orange, B is shown in green, and C is shown in orange. Structures are first ranked and optimized with PBE+MBD using *lower-level* settings followed by re-ranking with PBE0+MBD using *lower-level* settings. Structures are then reoptimized and re-ranked with PBE+MBD using higher-level settings (PBE+MBD tight). Zero-point energy (PBE0+MBD+ZPE) and thermal corrections (PBE0+MBD+F_{300}) are calculated with PBE+MBD using *lower-level* settings. All structures were optimized using the AIMNet MLP before additional thermal corrections, also calculated with AIMNet, (AIMNet + T) were added. As can be seen, the A major form is ranked as the most stable by almost every method while the C form is ranked as the least stable. The correct ranking at both 0 and 300 K is B > A > C. None of the 0 K methods obtained the correct ranking, where A > B > C. The only method that obtained the correct ranking is PBE0+MBD+F_{300} which ranked the B form as ~2 kJ/mol more stable than the A form and ~6 kJ/mol more stable than the C form. The A minor form is drastically destabilized in comparison to the B form at this level of theory. The other method that considered thermal corrections, AIMNet+T, did stabilize the B form in relation to the A minor form but still ranked the A major form as the most stable. The destabilization of the A minor form and the stabilization of the B form could be explained by the amount of thermal expansion each material underwent. As seen in Figure 4, the A minor form underwent almost twice as much thermal expansion as the B form. The more thermal expansion a material undergoes, the larger the Kirchoff term in Equation 1, and the more destabilized it will be after thermal corrections are added.

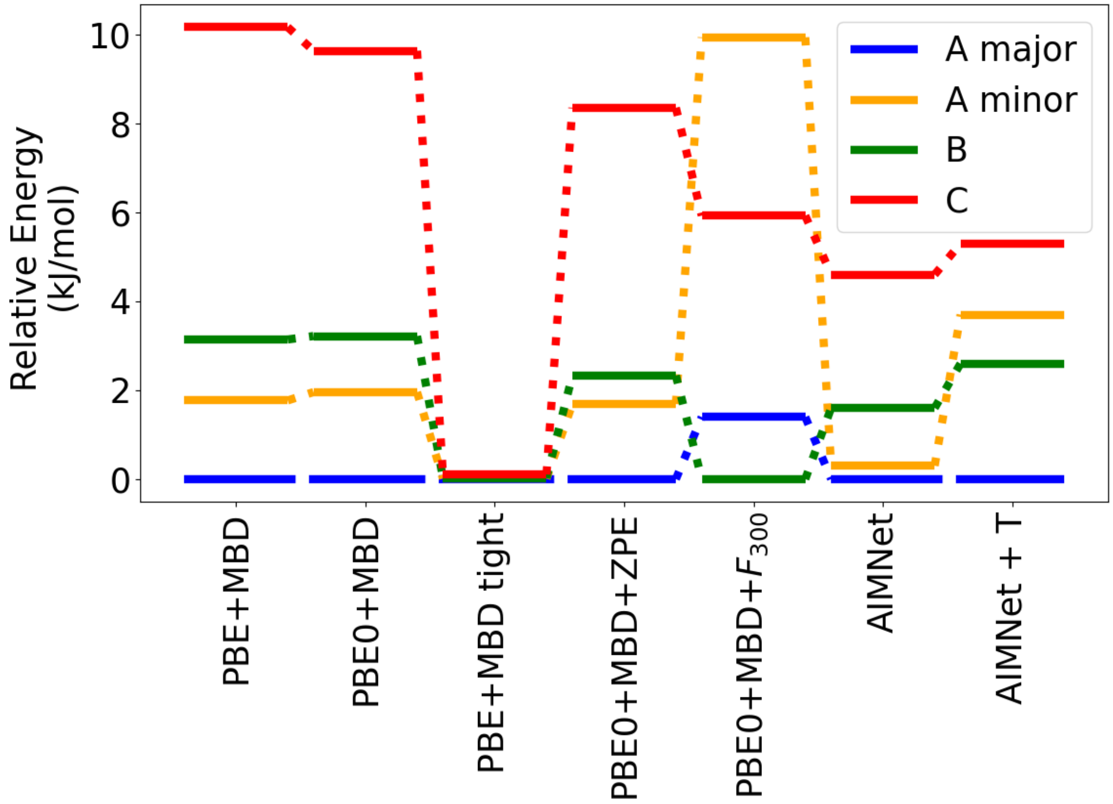

Figure 5: Rankings using different DFT methods and AIMNet for the experimental forms of Target XXXI. All DFT calculations are carried out with lower-level settings unless noted otherwise.

3.3 Target XXXII

For this target, two experimental forms were given: A (structure 317) and B (structure 232). We first compared the RMSD values for the structures optimized with PBE+MBD with *lower-level* settings, PBE+MBD with *higher-level* settings, and AIMNet. The results are shown in Table 2. As can be seen, PBE+MBD with *higher-level* settings yields the lowest RMSD for the A major form whereas AIMNet yields the lowest RMSD for the B form. The B form also generally had higher RMSD values than the A major form. As stated in the main manuscript, there is low confidence in the crystal structure of the B form, which may explain the higher RMSD values observed here.

Table 2 Summary of root mean squared deviations (RMSD) between different methodologies in comparison to the 2 experimentally characterized ground truths for target XXXII.

**Target XXXII**			
**A Major Structure**		**B Structure**	
Method	RMSD (Å)	Method	RMSD (Å)
PBE+MBD, lower level	0.056	PBE+MBD, lower level	0.164
PBE+MBD, higher level	0.047	PBE+MBD, higher level	0.159
AIMNet	0.097	AIMNet	0.126

Because DFT calculations were only conducted post-hoc for Target XXXII there is no volume-parity plot comparing AIMNet to PBE+MBD. Figure 6 shows the percent error for the experimental forms of Target XXXII with four different volume treatments: the electronic volume V_{el} calculated with DFT at 0 K, V_{ZPVE} calculated within the QHA at 0 K, V_{RT} calculated within the QHA at 300 K, and the volume calculated with AIMNet $V_{AIMNet.}$ Similar to Target XXXI, V_{el} for both forms are underestimates while V_{RT} are overestimated, though only by ~1% for both forms. Furthermore, both V_{ZPVE} and V_{AIMNet} underestimate both forms, unlike Target XXXI. From this, it is evident that explicit treatment of thermal expansion provided the best volume estimation for both forms of this target.

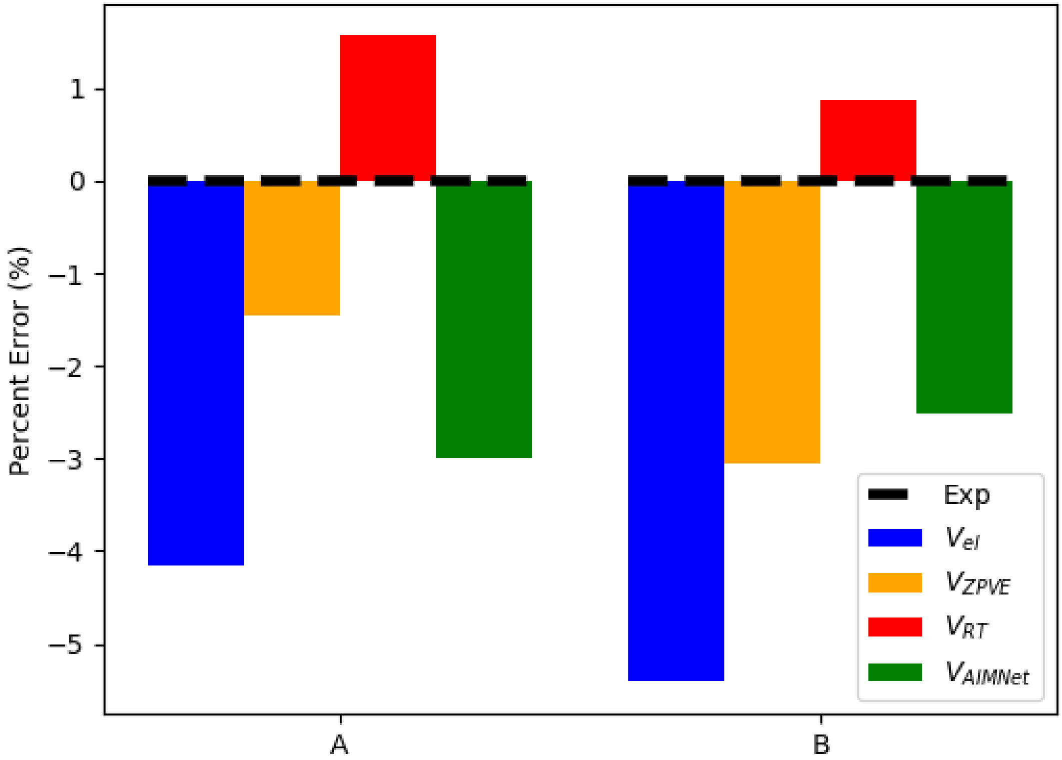

Figure 6: Comparison of volumes calculated with different DFT and ML methods. V_{el} refers to the electronic volume calculated at 0 K calculated with PBE+MBD using *lower-level* settings, V_{ZPVE} refers to the volume including zero-point vibrational effects calculated with PBE+MBD, V_{RT} refers to the volume at room temperature calculated within the quasi-harmonic approximation with PBE+MBD, and V_{AIMNet} refers to the volume calculated with AIMNet at 0 K.

With this in mind, we also examined the role of thermal expansion for this target. Figure 7 shows the expansion from V_{el} to V_{ZPVE} to V_{RT} for the experimental forms of Target XXXII. Expansion between V_e to V_{ZPVE} represents expansion due to vibrational effects and expansion between V_{ZPVE} to V_{RT} represents expansion due to thermal effects. As can be seen, thermal effects played a dominant role in thermal expansion for both forms, with forms A and B expanding by 6.00% and 6.45%, respectively. This is still more than the 5% observed by Hoja *et al.* in the X23 benchmark but less than Target XXXI. Hence, explicit treatment of thermal expansion also appears necessary for this target.

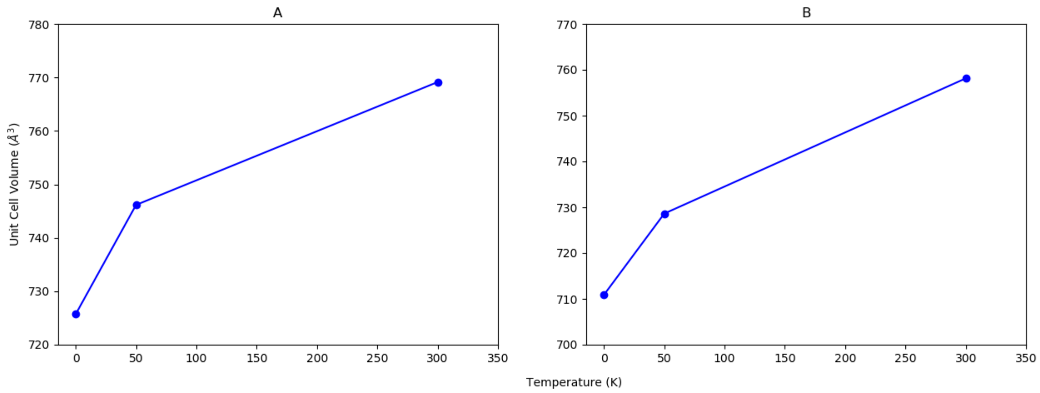

Figure 7: Unit cell volume expansion from V_{el} to V_{ZPVE} to V_{RT} for the experimental forms of Target XXXII.

Figure 8 shows the rankings of the two experimental forms of Target XXXII with different DFT and ML methods. A is shown in orange and B is shown in blue. Structures are first ranked and optimized with PBE+MBD using *lower-level* settings followed by SPE calculations with PBE0+MBD using lower-level settings. Structures are then reoptimized and re-ranked with PBE+MBD using higher-level settings (PBE+MBD tight). This is followed by additional zero-point energy corrections (PBE0+MBD+ZPE). All structures were optimized with the AIMNet MLP before thermal corrections, calculated by AIMNet, were added (AIMNet + T). As can be seen, the B form is ranked as the most stable by every method. Interestingly, the two forms are ranked as almost degenerate with PBE+MBD using *higher-level* settings. On the other hand, the A form is destabilized by ~2 kJ/mol when ZPE contributions are added, by ~6 kJ/mol when using AIMNet, and by ~3 kJ/mol when thermal corrections are added to AIMNet. The correct ranking at 0 K is B > A, which is reflected by all the 0 K methods. The correct ranking at 300 K has still not been determined, so the performance of the methods at this temperature cannot be commented on at this time.

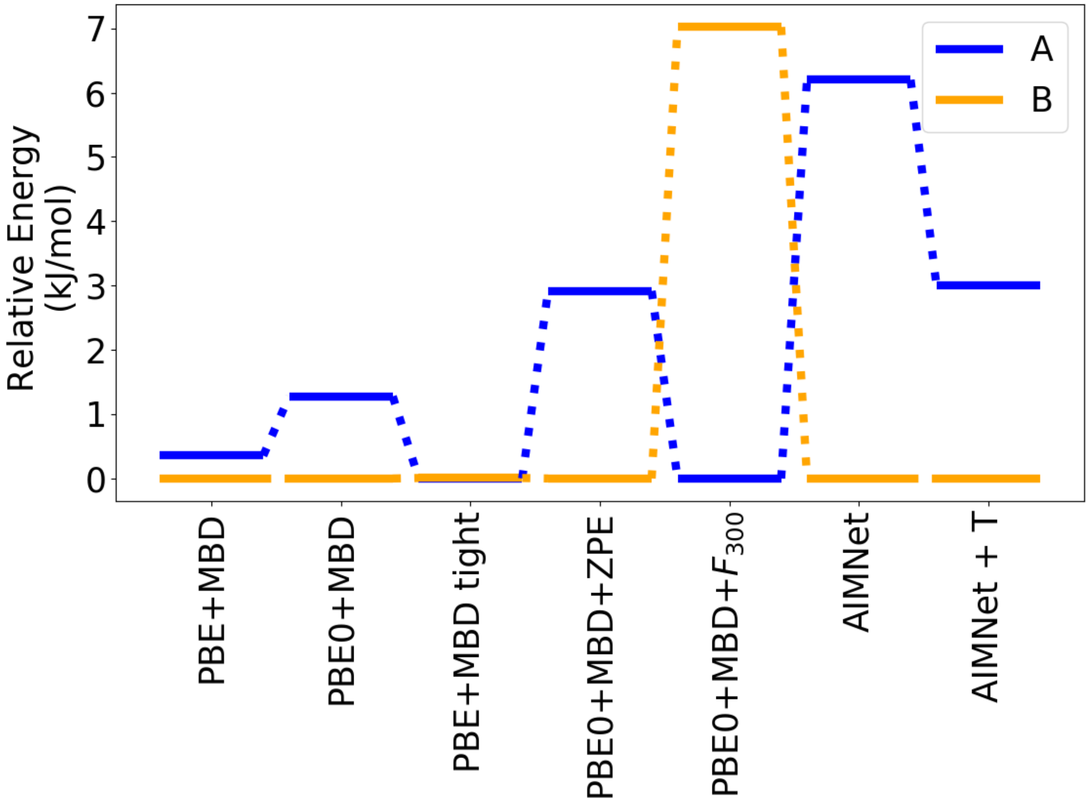

Figure 8: Rankings using different DFT methods and AIMNet for the experimental forms of Target XXXII. All DFT calculations are carried out with lower-level settings unless noted otherwise.

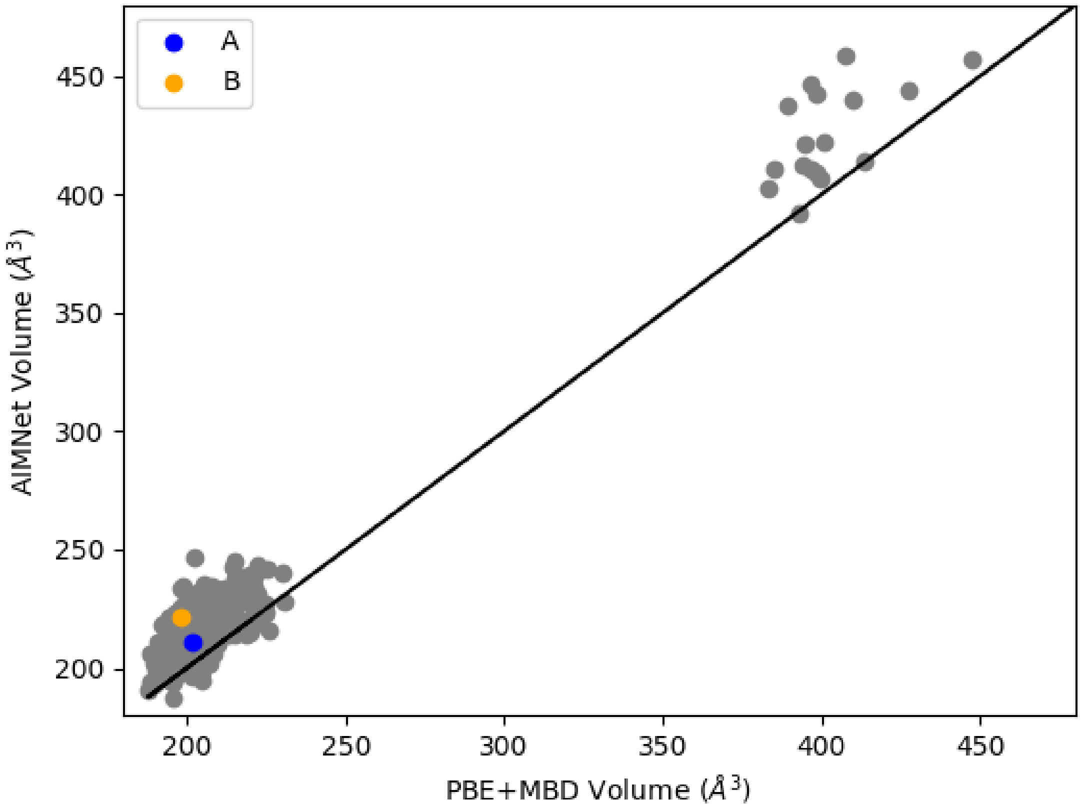

Figure 9: Volume parity plot for target XXXIII crystal structures between AIMNet trained on N-mers PBE/def2-mTZVPP with D4 dispersion corrections and those optimized with DFT PBE+MBD.

3.4 Target XXXIII

For this target two experimental forms were given: A (structure 233) and B (structure 452). Figure 8 is the volume parity plot between structures optimized with AIMNet and PBE+MBD using *lower-level* settings. The experimental forms are colored. As can be seen, AIMNet tends to overestimate the volume in comparison to PBE+MBD, though at higher volumes AIMNet almost achieves parity. This is unexpected because PBE+MBD is expected to yield larger volumes than AIMNet since pairwise dispersion methods tend to overestimate intermolecular interactions which yields smaller volumes.

Following this further, we also examined the estimated volumes when considering zero-point vibrational and thermal effects. Figure 10 shows the percent error for the experimental forms of Target XXXIII with four different volume treatments: the electronic volume V_{el} calculated with DFT at 0 K, V_{ZPVE} calculated within the QHA at 0 K, V_{RT} calculated within the QHA at 300 K, and the volume calculated with AIMNet $V_{AIMNet.}$ Similar to the other two targets, V_{el} is underestimated for both forms. Similar to Target XXXII, V_{ZPVE} also underestimates. However, V_{RT} for both forms underestimates. In fact the only overestimate is V_{AIMNet} for form A. While still an underestimate, the explicit treatment of thermal expansion with DFT still provided the most accurate volume estimations, suggesting that this treatment is necessary for this target.

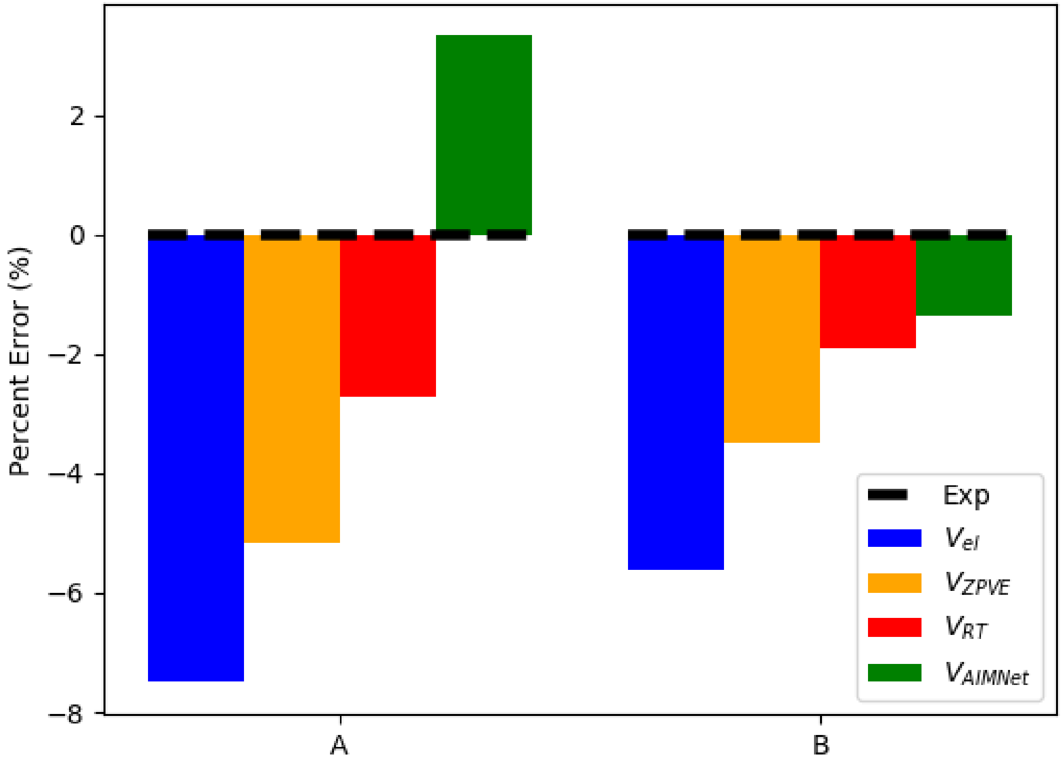

Figure 10: Comparison of volumes calculated with different DFT and ML methods. V_{el} refers to the electronic volume calculated at 0 K calculated with PBE+MBD using lower-level settings, V_{ZPVE} refers to the volume including zero-point vibrational effects calculated with PBE+MBD, V_{RT} refers to the volume at room temperature calculated within the quasi-harmonic approximation with PBE+MBD, and V_{AIMNet} refers to the volume calculated with AIMNet at 0 K.

Figure 7 shows the expansion from V_{el} to V_{ZPVE} to V_{RT} for the experimental forms of Target XXXII. Expansion between V_e to V_{ZPVE} represents expansion due to vibrational effects and expansion between V_{ZPVE} to V_{RT} represents expansion due to thermal effects. As can be seen, expansion due to thermal effects dominates for form A but seems to be almost equal to vibrational effects for form B. This is reflected in the amount of thermal expansion these two forms underwent, where form A had 2.5% and 5.2% for vibrational and thermal effects, respectively, and form B had 2.3% and 3.9% for vibrational and thermal effects, respectively. Additionally, these two forms seem to behave more like the X23 benchmark set, experiencing ~5% thermal expansion. This lack of thermal expansion could also explain the underestimates seen in Figure 10.

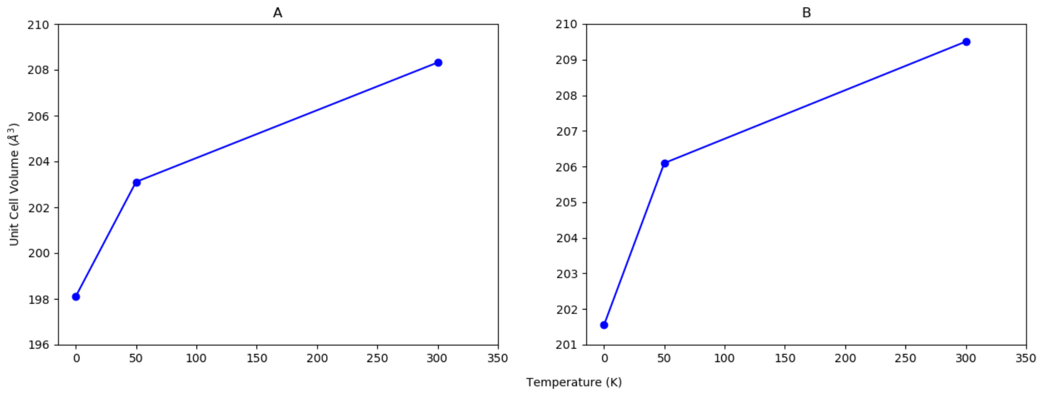

Figure 11: Unit cell volume expansion from V_{el} to V_{ZPVE} to V_{RT} for the experimental forms of Target XXXII.

Figure 12 shows the rankings of the two experimental forms of Target XXXIII with different DFT and ML methods. A is shown in blue and B is shown in orange. Structures are first ranked and optimized with PBE+MBD using *lower-level* settings. SPE calculations with PBE0+MBD using lower-level settings are used to re-rank the structures. Structures are also reoptimized and re-ranked with PBE+MBD using higher-level settings (PBE0+MBD tight). Zero-point energy corrections are then calculated with PBE+MBD using *lower-level* settings (PBE+MBD+ZPE). All structures are optimized with AIMNet before additional thermal corrections, also calculated with AIMNet, are added (AIMNet + T). As can be seen, the B form is ranked as the most stable form by all methods. The A form is increasingly destabilized when ZPE corrections are added as well as evaluation by AIMNet. However, this structure is only destabilized by a maximum of 1 kJ/mol. The correct ranking at both 0 K and 300 K is B > A, which all the methods achieved.

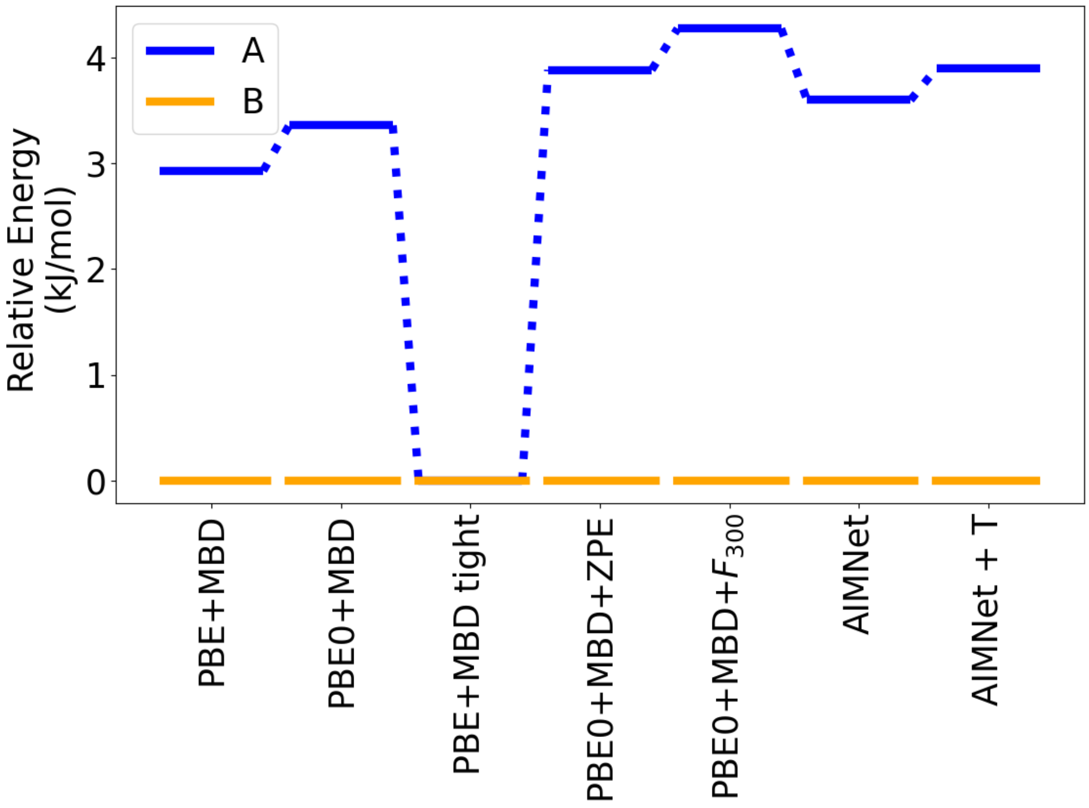

Figure 12: Rankings using different DFT methods and AIMNet for the experimental forms of Target XXXIII. All DFT calculations are carried out with lower-level settings unless noted otherwise.

4 Summary of Computational Resources

The following computational resources were utilized:

- PSC Bridges2 supercomputer (AMD EPYC 7742 CPUs)
- SDSC Expanse supercomputer (V100 GPUs)
- ALCF Theta supercomputer (Intel-Cray XC40, Intel Xeon Phi 7230 CPUs)
- NERSC Cori supercomputer (Intel-Cray XC40, Intel Xeon E5-2698 v3 CPUs)
- NERSC Perlmutter supercomputer (AMD EPYC 7763 (Milan) CPUs)
- PSC Arjuna Cluster (Intel Xeon E5-2683 CPUs)

The total computational time spent came from the following steps:

<u>CSP Blind Test Predictions</u>

- Training data generation at PBE/mTZVPP level: 1.4 x 10^5 CPU core-hours
- AIMNet model training: 250 GPU-hours
- Geometry optimizations, cell volume scans, force constant calculation: 210 GPU-hours

Acknowledgements

O.I. acknowledges support from NSF CHE-1802789 and CHE-2041108. We also acknowledge the Extreme Science and Engineering Discovery Environment (XSEDE) award CHE200122, which is supported by NSF grant number ACI-1053575. This research is part of the Frontera computing project at the Texas Advanced Computing Center. Frontera is made possible by the National Science Foundation award OAC-1818253. This research in part was done using resources provided by the Open Science Grid, which is supported by the award 1148698, and the U.S. DOE Office of Science.

N.M. acknowledges support from National Science Foundation (NSF) through grant DMR-2131944. This research used resources of Argonne Leadership Computing Facility (ALCF), which is a DOE Office of Science User Facility supported under Contract DE-AC02-06CH11357. We also acknowledge the Extreme Science and Engineering Discovery Environment (XSEDE) award MAT210006, which supported 3M CPU core-hours.

References

(1) Perdew, J. P.; Burke, K.; Ernzerhof, M. Generalized Gradient Approximation Made Simple. *Phys Rev Lett* **1996**, *77* (18), 3865–3868. https://doi.org/10.1103/PhysRevLett.77.3865.

(2) Caldeweyher, E.; Bannwarth, C.; Grimme, S. Extension of the D3 Dispersion Coefficient Model. *J Chem Phys* **2017**, *147* (3), 034112. https://doi.org/10.1063/1.4993215.

(3) Togo, A.; Tanaka, I. First Principles Phonon Calculations in Materials Science. *Scr Mater* **2015**, *108*, 1–5. https://doi.org/10.1016/j.scriptamat.2015.07.021.

(4) Zubatyuk, R.; Smith, J. S.; Leszczynski, J.; Isayev, O. Accurate and Transferable Multitask Prediction of Chemical Properties with an Atoms-in-Molecules Neural Network. *Sci Adv* **2022**, *5* (8), eaav6490. https://doi.org/10.1126/sciadv.aav6490.

(5) Zubatyuk, R.; Smith, J. S.; Nebgen, B. T.; Tretiak, S.; Isayev, O. Teaching a Neural Network to Attach and Detach Electrons from Molecules. *Nat Commun* **2021**, *12* (1), 4870. https://doi.org/10.1038/s41467-021-24904-0.

(6) Behler, J. Four Generations of High-Dimensional Neural Network Potentials. *Chem Rev* **2021**, *121* (16), 10037–10072. https://doi.org/10.1021/acs.chemrev.0c00868.

(7) Behler, J. Atom-Centered Symmetry Functions for Constructing High-Dimensional Neural Network Potentials. *J Chem Phys* **2011**, *134* (7), 074106. https://doi.org/10.1063/1.3553717.

(8) Smith, J. S.; Isayev, O.; Roitberg, A. E. ANI-1: An Extensible Neural Network Potential with DFT Accuracy at Force Field Computational Cost. *Chem Sci* **2017**, *8* (4), 3192–3203. https://doi.org/10.1039/C6SC05720A.

(9) Deringer, V. L.; Caro, M. A.; Csányi, G. Machine Learning Interatomic Potentials as Emerging Tools for Materials Science. *Advanced Materials* **2019**, *31* (46), 1902765. https://doi.org/https://doi.org/10.1002/adma.201902765.

(10) Schütt, K. T.; Sauceda, H. E.; Kindermans, P.-J.; Tkatchenko, A.; Müller, K.-R. SchNet – A Deep Learning Architecture for Molecules and Materials. *J Chem Phys* **2018**, *148* (24), 241722. https://doi.org/10.1063/1.5019779.

(11) Spicher, S.; Grimme, S. Robust Atomistic Modeling of Materials, Organometallic, and Biochemical Systems. *Angewandte Chemie International Edition* **2020**, *59* (36), 15665–15673. https://doi.org/10.1002/anie.202004239.

(12) Bannwarth, C.; Ehlert, S.; Grimme, S. GFN2-XTB—An Accurate and Broadly Parametrized Self-Consistent Tight-Binding Quantum Chemical Method with Multipole Electrostatics and Density-Dependent Dispersion Contributions. *J Chem Theory Comput* **2019**, *15* (3), 1652–1671. https://doi.org/10.1021/acs.jctc.8b01176.

(13) Neese, F. The ORCA Program System. *WIREs Computational Molecular Science* **2012**, *2* (1), 73–78. https://doi.org/https://doi.org/10.1002/wcms.81.

(14) Neese, F. Software Update: The ORCA Program System—Version 5.0. *WIREs Computational Molecular Science* **2022**, *12* (5), e1606. https://doi.org/10.1002/wcms.1606.

(15) Ewald, P. P. Die Berechnung Optischer Und Elektrostatischer Gitterpotentiale. *Ann Phys* **1921**, *369* (3), 253–287. https://doi.org/10.1002/andp.19213690304.

(16) Paszke, A.; Gross, S.; Massa, F.; Lerer, A.; Bradbury, J.; Chanan, G.; Killeen, T.; Lin, Z.; Gimelshein, N.; Antiga, L.; others. Pytorch: An Imperative Style, High-Performance Deep Learning Library. *Adv Neural Inf Process Syst* **2019**, *32* https://doi.org/10.48550/arXiv.1912.01703.

(17) Blum, V.; Gehrke, R.; Hanke, F.; Havu, P.; Havu, V.; Ren, X.; Reuter, K.; Scheffler, M. Ab Initio Molecular Simulations with Numeric Atom-Centered Orbitals. *Comput Phys Commun* **2009**, *180* (11), 2175–2196. https://doi.org/10.1016/j.cpc.2009.06.022.

(18) Reilly, A. M.; Tkatchenko, A. Seamless and Accurate Modeling of Organic Molecular Materials. *J Phys Chem Lett* **2013**, *4* (6), 1028–1033. https://doi.org/10.1021/jz400226x.

(19) Hoja, J.; Reilly, A. M.; Tkatchenko, A. First-Principles Modeling of Molecular Crystals: Structures and Stabilities, Temperature and Pressure. *WIREs Computational Molecular Science* **2017**, *7* (1), e1294. https://doi.org/10.1002/wcms.1294.

(20) Dolgonos, G. A.; Hoja, J.; Boese, A. D. Revised Values for the X23 Benchmark Set of Molecular Crystals. *Physical Chemistry Chemical Physics* **2019**, *21* (44), 24333–24344. https://doi.org/10.1039/C9CP04488D.

(21) Hoja, J.; Tkatchenko, A. First-Principles Stability Ranking of Molecular Crystal Polymorphs with the DFT+MBD Approach. *Faraday Discuss* **2018**, *211* (0), 253–274. https://doi.org/10.1039/C8FD00066B.

(22) Otero-de-la-Roza, A.; Johnson, E. R. A Benchmark for Non-Covalent Interactions in Solids. *J Chem Phys* **2012**, *137* (5), 054103. https://doi.org/10.1063/1.4738961.

(23) Heit, Y. N.; Beran, G. J. O. How Important Is Thermal Expansion for Predicting Molecular Crystal Structures and Thermochemistry at Finite Temperatures? *Acta Crystallographica Section B* **2016**, *72* (4), 514–529. https://doi.org/10.1107/S2052520616005382.

(24) O'Connor, D.; Bier, I.; Hsieh, Y.-T.; Marom, N. Performance of Dispersion-Inclusive Density Functional Theory Methods for Energetic Materials. *J Chem Theory Comput* **2022**, *18* (7), 4456–4471. https://doi.org/10.1021/acs.jctc.2c00350.

(25) Tkatchenko, A.; DiStasio, R. A.; Car, R.; Scheffler, M. Accurate and Efficient Method for Many-Body van Der Waals Interactions. *Phys Rev Lett* **2012**, *108* (23), 236402. https://doi.org/10.1103/PhysRevLett.108.236402.

(26) Adamo, C.; Barone, V. Toward Reliable Density Functional Methods without Adjustable Parameters: The PBE0 Model. *J Chem Phys* **1999**, *110* (13), 6158–6170. https://doi.org/10.1063/1.478522.

(27) Ong, S. P.; Richards, W. D.; Jain, A.; Hautier, G.; Kocher, M.; Cholia, S.; Gunter, D.; Chevrier, V. L.; Persson, K. A.; Ceder, G. Python Materials Genomics (Pymatgen): A Robust, Open-Source Python Library for Materials Analysis. *Comput Mater Sci* **2013**, *68*, 314–319. https://doi.org/10.1016/j.commatsci.2012.10.028.

14. Group 17

Supplementary Information for The 7th CSP Blind Test, Phase 2

Hiroyuki Matsui and Kotaro Shinohara

Research Center for Organic Electronics (ROEL), Yamagata University, Jonan 4-3-16, Yonezawa, Yamagata, 992-8510 Japan

h-matsui@yz.yamagata-u.ac.jp

Ranking in the second phase was performed based on the free energy under the quasi-harmonic approximation (QHA)[1]. The difference between the harmonic approximation (HA) and the QHA is that the QHA includes the cell optimisation with the free energy.

The Gibbs free energy G was calculated as

$$G = U_{\mathrm{st}} + pV + U_{\mathrm{ph}} - TS_{\mathrm{ph}} = U_{\mathrm{st}} + pV + \sum_{\omega} k_{\mathrm{B}}T \ln\left[2\sinh\left(\frac{\hbar\omega}{2k_{\mathrm{B}}T}\right)\right]$$

where U_{st} is the static lattice energy, p the pressure, V the cell volume, U_{ph} the internal energy of phonons, T the temperature, S_{ph} the entropy of phonons, k_{B} the Boltzmann constant, $\hbar$ the reduced Planck constant, and ω the angular frequency of photons. We take the lattice vectors $\{\mathbf{a}, \mathbf{b}, \mathbf{c}\}$ as independent variables for the Gibbs energy, $G(\mathbf{a}, \mathbf{b}, \mathbf{c})$, while the coordinates of the atoms in a reference cell $\{\mathbf{r}_i\}$ were considered as dependent variables. For specific cell parameters $\{\mathbf{a}, \mathbf{b}, \mathbf{c}\}$, the atom coordinates $\{\mathbf{r}_i\}$ were optimised and then the phonon modes were calculated to obtain the Gibbs energy. This ensures that the phonon calculations were performed only at the stable positions of the atoms. Note that the function $G(\mathbf{a}, \mathbf{b}, \mathbf{c})$ is a multivalued function because there can be multiple sets of $\{\mathbf{r}_i\}$ for stable states in a cell $\{\mathbf{a}, \mathbf{b}, \mathbf{c}\}$. The QHA approach is known to reproduce thermal expansion.

Structure optimisation of all 100 structures was performed under the QHA using GULP software[2]. Temperature and pressure were set to 300 K and 1 atm respectively.

[1] J. D. Gale, "Analytical Free Energy Minimization of Silica Polymorphs", *J. Phys. Chem. B* 102 (1998) 5423-5431.

[2] General Utility Lattice Program (GULP), Version 5.2, https://gulp.curtin.edu.au/

15. Group 18

Authors & Affiliations

Zeinab M. Saeed,[1] Bhausaheb Dhokale,[1][2] Tamador Alkhidir,[1] Mubarak Almehairbi,[1] and Sharmarke Mohamed[1][3]

[1] Green Chemistry & Materials Modelling Laboratory, Khalifa University of Science and Technology, P.O. Box 127788, Abu Dhabi (UAE)

[2] Department of Chemistry, University of Wyoming, Laramie, Wyoming 82071 (USA)

[3] Center for Catalysis and Separations, Khalifa University of Science and Technology, P.O. Box 127788, Abu Dhabi (UAE)

1. Detailed Methodologies for CSP (Submission 2)

1.1 Systems XXXI and XXXIII

For XXXI and XXXIII, CrystalOptimizer (Kazantsev *et al.*, 2011) was used to refine the lattice energies of the provided crystal structures using the selected torsion angles given in Figure S1. CrystalOptimizer estimates the total lattice energy of the crystal as a function of varying selected torsional degrees of freedom. The torsion scans were performed at the B97D/6-31G(d,p) level of theory using GAUSSIAN09 (Frisch *et al.*, 2009). The dispersion-repulsion contributions towards the lattice energy were modelled using a Buckingham exp-6 function using Williams' FIT potential parameters. For more details on the FIT potential parameters, the reader is referred to our previous work (Shruti *et al.*, 2022) using this potential parameter set. Final lattice energies were estimated using a distributed-multipole model using DMACRYS (Price *et al.*, 2010). The dispersion-repulsion contributions were estimated using the same FIT potential parameter set used in the initial CSP search but the electrostatic contributions were estimated using a more accurate distributed multipole model (up to rank 4) derived from the B97D/6-31G(d,p) wave function. The DMACRYS lattice energy optimization was performed using a rigid-body approximation. For details on the clustering methodology used, the reader is referred to our previous work (Shruti *et al.*, 2022).

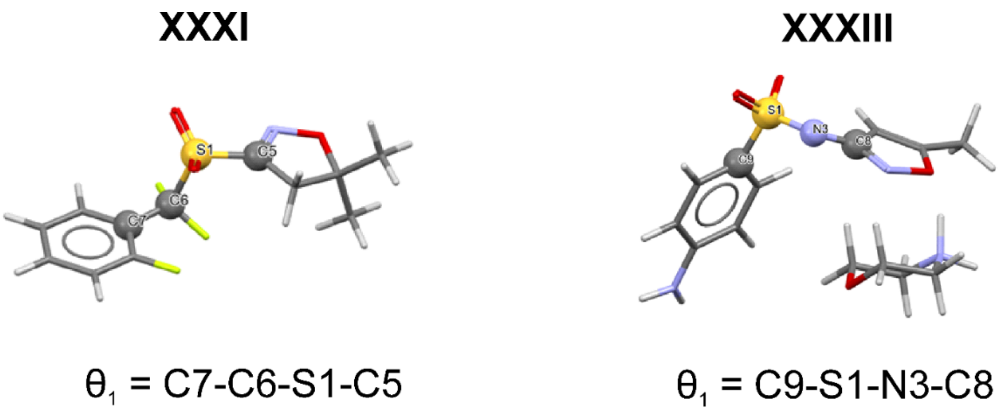

Figure S1: Molecular structures for XXXI and XXXIII drawn using a capped sticks model for all atoms except those defining the torsion scanned. The atoms of the torsions scanned as part of the CrystalOptimizer routine are shown using ball-and-stick model and are listed at the bottom of each molecular structure.

References

Frisch, M. J., Trucks, G. W., Schlegel, H. B., Scuseria, G. E., Robb, M. A., Cheeseman, J. R., Scalmani, G., Barone, V., Mennucci, B., Petersson, G. A., Nakatsuji, H., Caricato, M., Li, X., Hratchian, H. P., Izmaylov, A. F., Bloino, J., Zheng, G., Sonnenberg, J. L., Hada, M., Ehara, M., Toyota, K., Fukuda, R., Hasegawa, J., Ishida, M., Nakajima, T., Honda, Y., Kitao, O., Nakai, H., Vreven, T., Montgomery Jr., J. A., Peralta, J. E., Ogliaro, F., Bearpark, M. J., Heyd, J., Brothers, E. N., Kudin, K. N., Staroverov, V. N., Kobayashi, R., Normand, J., Raghavachari, K., Rendell, A. P., Burant, J. C., Iyengar, S. S., Tomasi, J., Cossi, M., Rega, N., Millam, N. J., Klene, M., Knox, J. E., Cross, J. B., Bakken, V., Adamo, C., Jaramillo, J., Gomperts, R., Stratmann, R. E., Yazyev, O., Austin, A. J., Cammi, R., Pomelli, C., Ochterski, J. W., Martin, R. L., Morokuma, K., Zakrzewski, V. G., Voth, G. A., Salvador, P., Dannenberg, J. J., Dapprich, S., Daniels, A. D., Farkas, Ö., Foresman, J. B., Ortiz, J. V., Cioslowski, J. & Fox, D. J. (2009). Version Revision D.01.

Kazantsev, A. V., Karamertzanis, P. G., Adjiman, C. S. & Pantelides, C. C. (2011). *J. Chem. Theory Comput.* **7**, 1998-2016.

Price, S. L., Leslie, M., Welch, G. W. A., Habgood, M., Price, L. S., Karamertzanis, P. G. & Day, G. M. (2010). *PCCP* **12**, 8478-8490.

Shruti, I., Almehairbi, M., Saeed, Z. M., Alkhidir, T., Ali, W. A., Vishwakarma, R., Mohamed, S. & Chopra, D. (2022). *Cryst. Growth Des.* **22**, 5861-5871.

16. Group 19

Supporting information statement for 7th Crystal Structure Prediction Challenge
Group: OpenEye Scientific Software
Contributors: Hari Muddana, Varsha Jain, Tom Darden, Geoff Skillman
Corresponding author: hmuddana@eyesopen.com

1. Methodology: Round2

QM optimization of crystal structures: We optimized the crystal structures using an approach referred to here as “dimer expansion”. In this approach, the total energy and gradients of the system are calculated as a sum of all dimers within the crystal. The fractional coordinates of the molecule and unit cell parameters are optimized separately in three iterations. First, we optimize the geometry of the molecule while keeping the unit cell parameters fixed, and then perform unit cell optimization while keeping the geometry of the molecule fixed. Finally, we perform another iteration of optimizing the geometry of the molecule. Conformer geometries were optimized using GeomeTRIC [1], and the unit cell parameters were optimized using NLOpt [2]. The gradients for both conformer geometry and unit cell parameters are computed using HF-3c method [3]. Specifically, we build a cluster of the crystal out to 12A and decompose that into dimers, accounting for symmetrically related pairs. We compute the energy and gradients of each dimer and sum them up to get the crystal energy and gradients, respectively. Optimization steps are continued until the large component of the gradient is less than 0.0001 Hartree/Angstrom, or a maximum number of steps has reached (shown in Table 1). Psi4 1.3.2 was used for all dimer energy and gradient calculations [4].

Scoring of crystal structures: To calculate the single point energy of the crystals after geometry optimization, we use the same “dimer expansion” approach to calculate the energy of the crystal at a higher level of theory. For short-range dimers (less than 6A), we used B3LYP-D3MBJ/6-31g*; for medium range dimers (6 to 12A), we used HF-3c. Psi4 1.3.2 was used for all dimer energy and gradient calculations.

Challenge	Optimization Method	Maximum number of steps of optimization (conformer geometry, unit cell, conformer geometry)	Scoring method
XXXI	HF-3c	50,50,50	B3LYP-D3MBJ/6-31g*//HF-3c
XXXII	HF-3c	25,25,25	B3LYP-D3MBJ/6-31g*//HF-3c
XXXIII	HF-3c	50,50,50	B3LYP-D3MBJ/6-31g*//HF-3c

Table 1. Optimization method/parameters and scoring methods used for XXXI, XXXII, and XXXIII challenges

References

[1] Wang, Lee-Ping, and Chenchen Song. "Geometry optimization made simple with translation and rotation coordinates." *The Journal of chemical physics* 144.21 (2016): 214108.
[2] Johnson, Steven G. "The NLopt nonlinear-optimization package." (2014).
[3] Sure, Rebecca, and Stefan Grimme. "Corrected small basis set Hartree-Fock method for large systems." *Journal of computational chemistry* 34.19 (2013): 1672-1685.
[4] Parrish, Robert M., et al. "Psi4 1.1: An open-source electronic structure program emphasizing automation, advanced libraries, and interoperability." *Journal of chemical theory and computation* 13.7 (2017): 3185-3197.

17. Group 20

Supporting information Group 20

Summary

The computational results of Group 20 are in full agreement with experiment. For six of the eight compounds one experimental structure corresponds to our rank 1 and if a second polymorph was found it corresponds to our rank 2. Based on polymorphism statistics on 41 pharmaceutical compounds, we expect the thermodynamically most stable crystal structure of one or two compounds in this blind test to be kinetically hindered, and indeed we predict that for compound XXXII the most stable structure has not been observed yet. For compound XXXI, the experimental structures lie within less than one standard deviation from the bottom of the energy window and therefore could correspond to the lowest free energy structures, but it cannot be ruled out that the most stable structure has not yet been experimentally observed.

Structures were energy minimised in a cascade of density functional theory (DFT) methods of increasing accuracy (for more details see, *e.g.*, Mortavi *et al*., 2019). Selection of structures to be carried forward to the final stage was supported by Machine Learning (ML) and by a tailor-made force field (TMFF, Neumann, 2008) with electrostatics described by multipoles. The ML is used to weed out structures that do not need to be considered for energy optimisation because they are predicted by the ML model to become high-energy structures with DFT despite their favourable TMFF energy, a publication with full details is in preparation. Final energies for the structures near the bottom of the energy window are the equivalent of free energies at 298.15 K with the PBE0 functional with the non-local multi-body dispersion (MBD) correction (Hermann & Tkatchenko, 2020) and a single-molecule dispersion-corrected second-order Møller-Plesset perturbation theory (MP2D) correction (Řezáč *et al*., 2018) which we refer to as the TRHu(ST) method for “Temperature- and Relative Humidity-dependent free-energies with STandard deviations”. Full details are published in Firaha *et al*., 2023. Structures higher up in the energy window have been evaluated with a less accurate energy potential but have been put on the same energy scale. Because the TRHu(ST) method was still under development when the blind test started, the energy landscapes of some blind test targets were calculated without the MP2D correction, which we refer to as PBE(0)+MBD+F_{vib}. For earlier blind tests, we used the PBE functional with the Neumann-Perrin dispersion correction, PBE+NP (Neumann & Perrin, 2005), and this is still used in the early stages of most projects. The energy method is indicated for each energy landscape.

The calculations were performed with our in-house developed software *GRACE*, which calls *VASP* (Kresse & Furthmüller, 1996, Kresse & Hafner, 1993, Kresse & Joubert, 1999) and *FHI-aims* (Blum *et al*., 2009) for DFT calculations.

The excellent reproduction of the experimental crystal structures with dispersion-corrected DFT is illustrated in the ESI of Group 20 in the Part One paper.

For XXVII (the Si compound), the experimental structure was not present among the 100 structures provided by the blind test organisers so we calculated its free energy separately and added it to the energy landscape (Figure 1). The PBE(0)+MBD+F_{vib} method was applied. The experimental structure is rank 1.

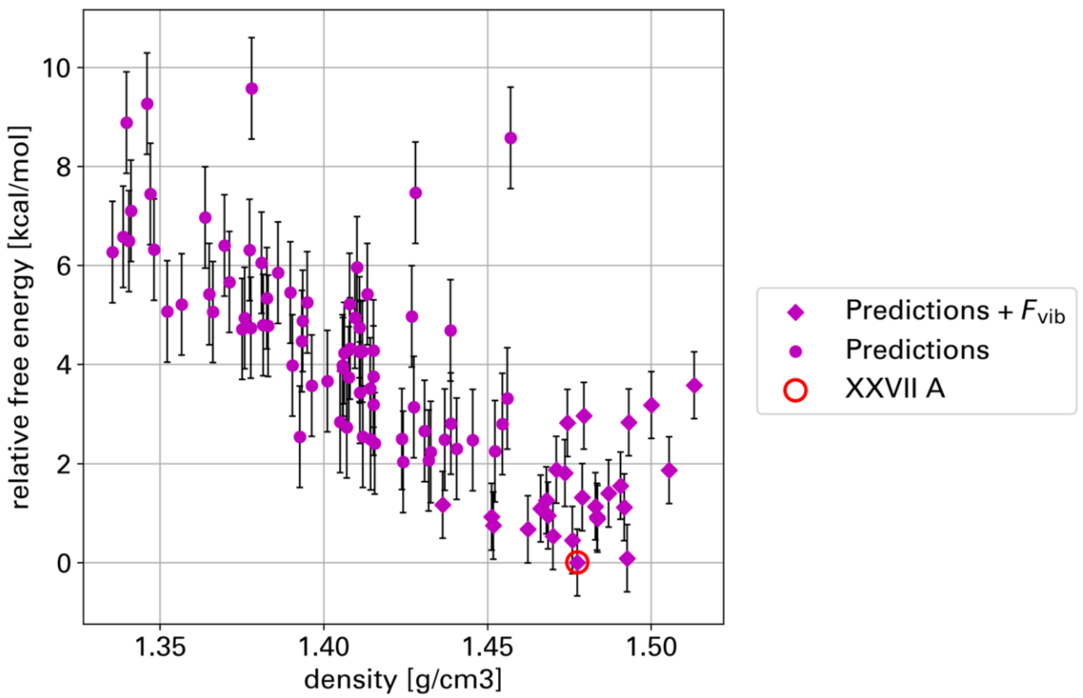

Figure 1. Energy landscape for compound XXVII with the (true) experimental structure added.

For XXVIII (the Cu compound), the experimental structure is rank 1. The PBE(0)+MBD+F_{vib} method was applied, but because no Neumann-Perrin dispersion-correction parameters were available for copper, PBE+NP was replaced by PBE+MBD throughout.

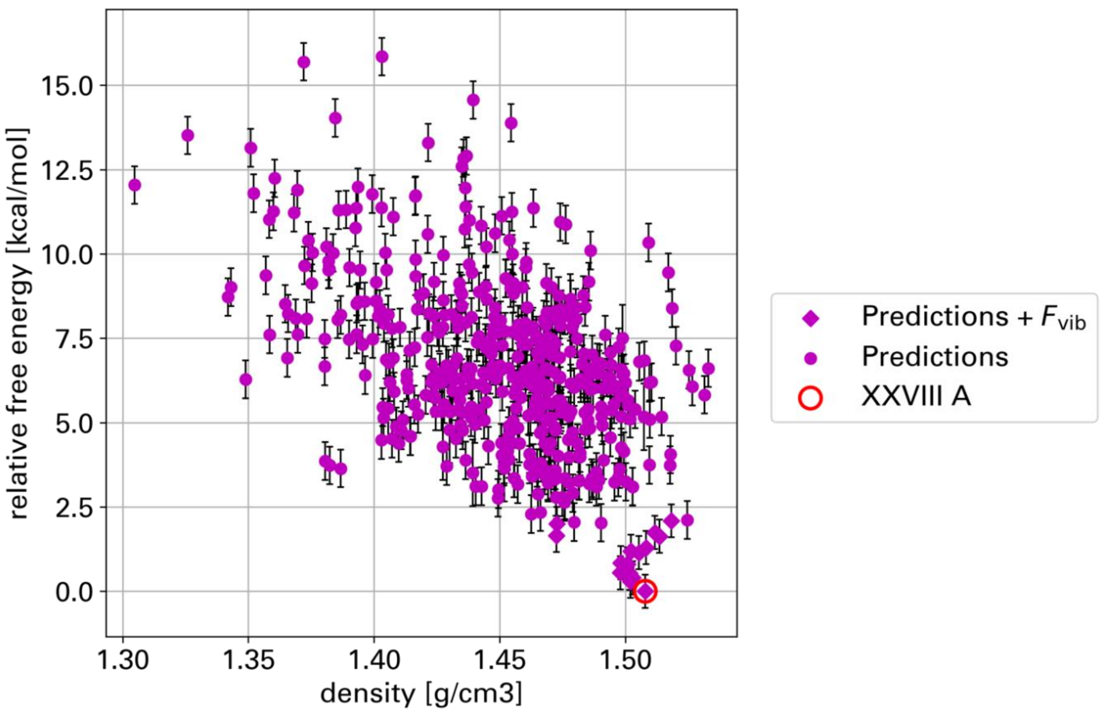

Figure 2. Energy landscape for compound XXVIII.

For XXIX (the experimentally-assisted challenge from the Structure Generation part of this blind test) the task that was given was to generate the crystal structure that matched the simulated powder diffraction pattern, and a free energy ranking was not required. The energies that we submitted were 0 K PBE+NP energies and the experimental structure was rank 1 (Figure 3). In our post analysis, we confirmed that the experimental structure is still rank 1 with the PBE(0)+MBD+F_{vib} method.

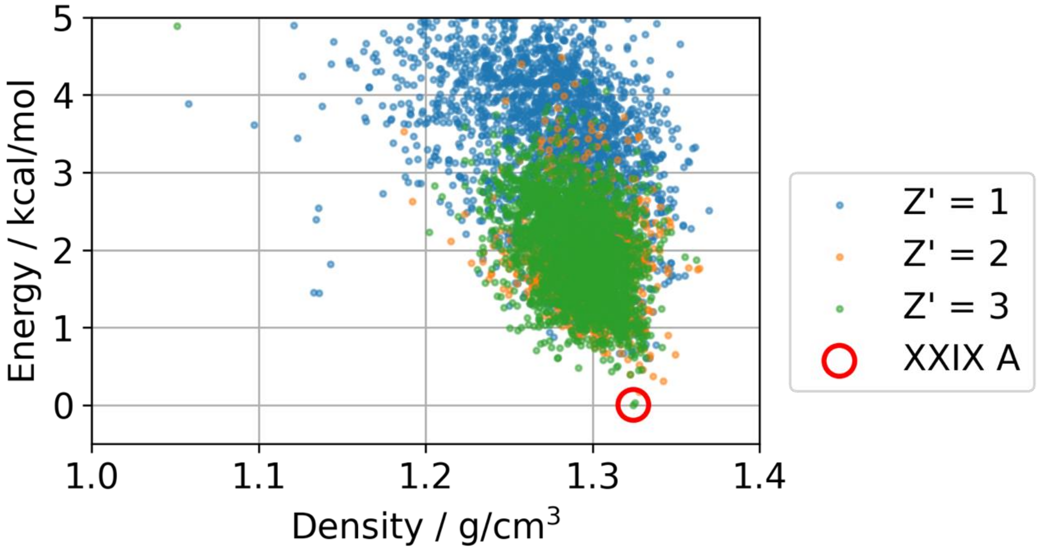

Figure 3. Energy landscape for compound XXIX.

Compound XXX was the stoichiometry challenge, consisting of, at least potentially, polymorphs of the 1:2, 1:1 and 2:1 co-crystals of cannabinol and tetramethylpyrazine. We predicted that only 1:1 and 2:1 co-crystals were thermodynamically stable, which turned out to be in agreement with experiment. One experimental crystal structure is known for the 2:1 compound, which in our calculations is rank 1 for that stoichiometry with PBE(0)+MBD+F_{vib} (Figure 4). Two experimental crystal structures are known for the 1:1 compound, which in our calculations are ranks 1 and 2 for that stoichiometry (Figure 5).

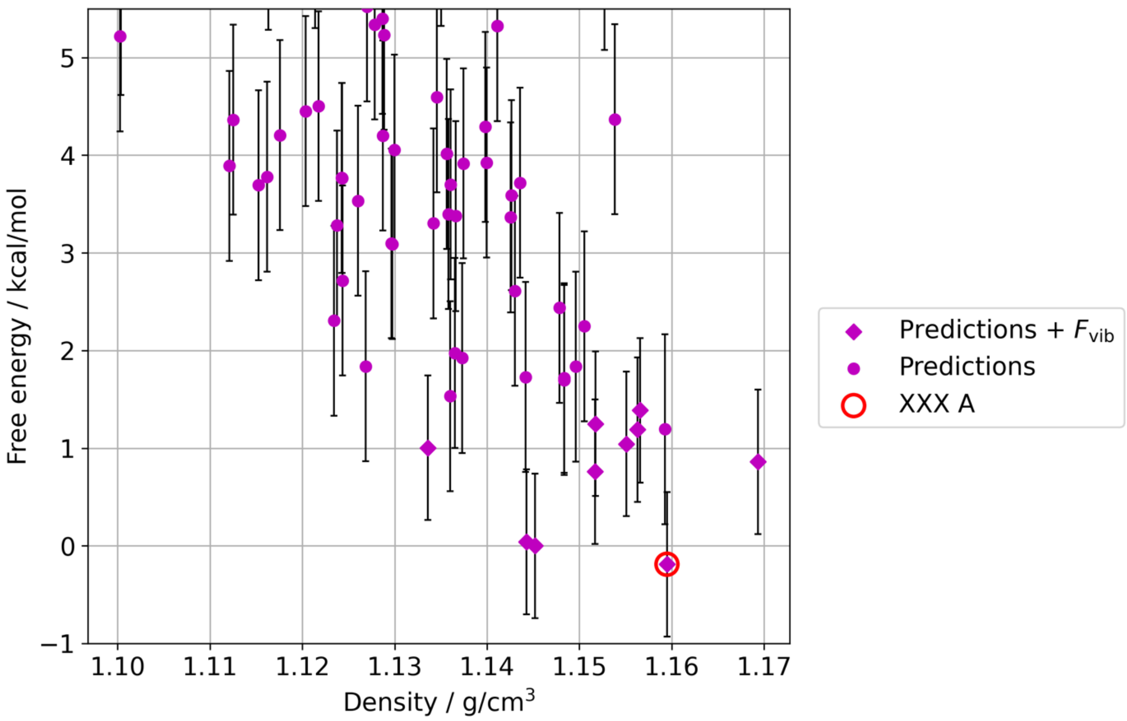

Figure 4. Energy landscape for compound XXX 2:1.

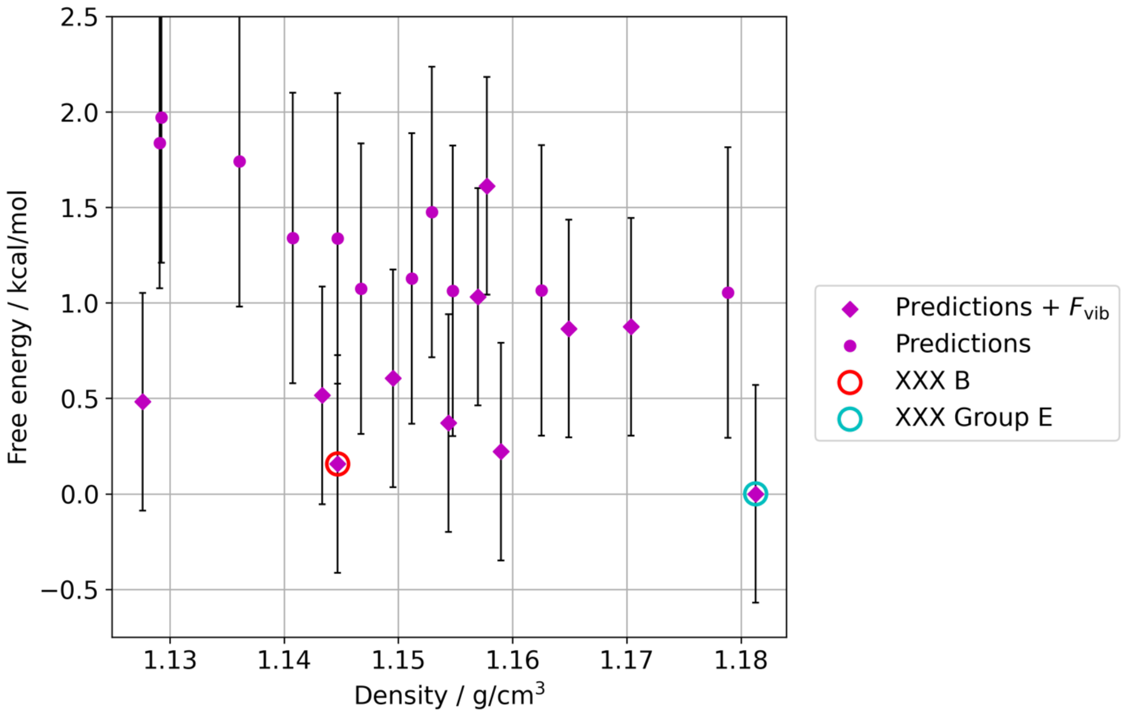

Figure 5. Energy landscape for compound XXX 1:1.

The energy landscape for XXXI at the TRHu(ST) level is shown in Figure 6. Form C, which contains large voids, has an unfavourable energy and was therefore considered thermodynamically irrelevant and in our cascade of energy methods its energy had not been

evaluated with our most accurate energy potential. In our post analysis, we recalculated Form C's free energy at the TRHu(ST) level, and this is the energy that is plotted in Figure 6.

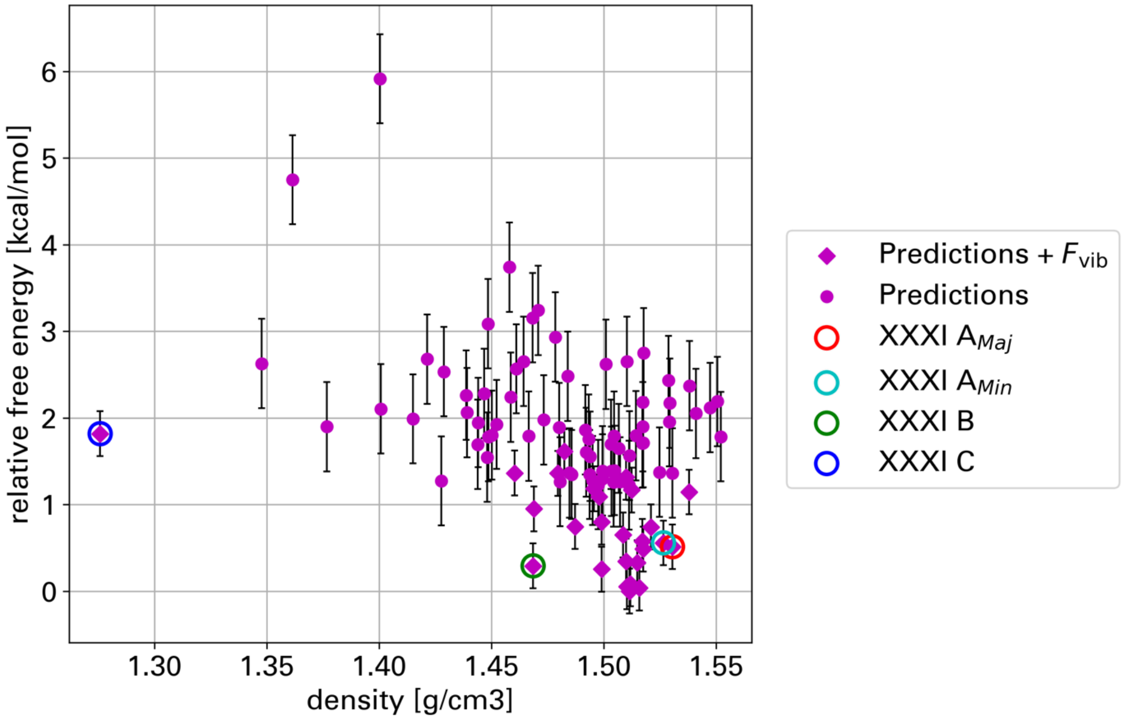

Figure 6. Energy landscape for compound XXXI.

The disorder in Form A had not been communicated by the blind test organisers when we submitted our results, and when not taken into account, the free energy of the main occupancy at 298.15 K is higher than that of Form B, in fortuitous agreement with experiment. When we recalculated the temperature-dependent free energies taking the disorder into account as part of our post analysis, the two experimentally observed phases were calculated to have the same free energy within one standard deviation over the entire temperature range from 0 K to 900 K (Figure 7). The two free energy curves are close to parallel over the entire temperature range, and within one standard deviation, the calculated free energies cannot distinguish which of the two structures is the thermodynamically more stable one.

Transferring the results from the disorder-aware free energy calculation of Form A (Figure 7) to the energy landscape (Figure 6), we see that both Form A and Form B are within one standard deviation from the global minimum.

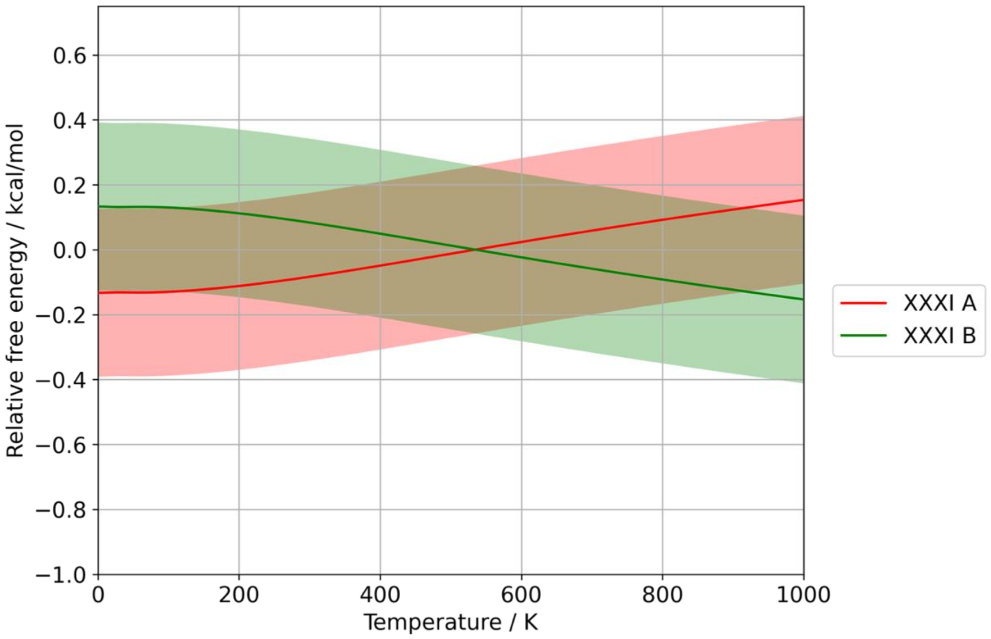

Figure 7. Enantiotropic stability relationship of the two forms A and B of XXXI as calculated with our TRHu(ST) method, taking the disorder in Form A into account.

The energy landscape for XXXII at the TRHu(ST) level is shown in Figure 8. As described in the ESI of Group 20 for Part One, we derived a better model for the room-temperature form of Form B, which was confirmed by the organisers, and the energy of the corrected model is plotted in Figure 8.

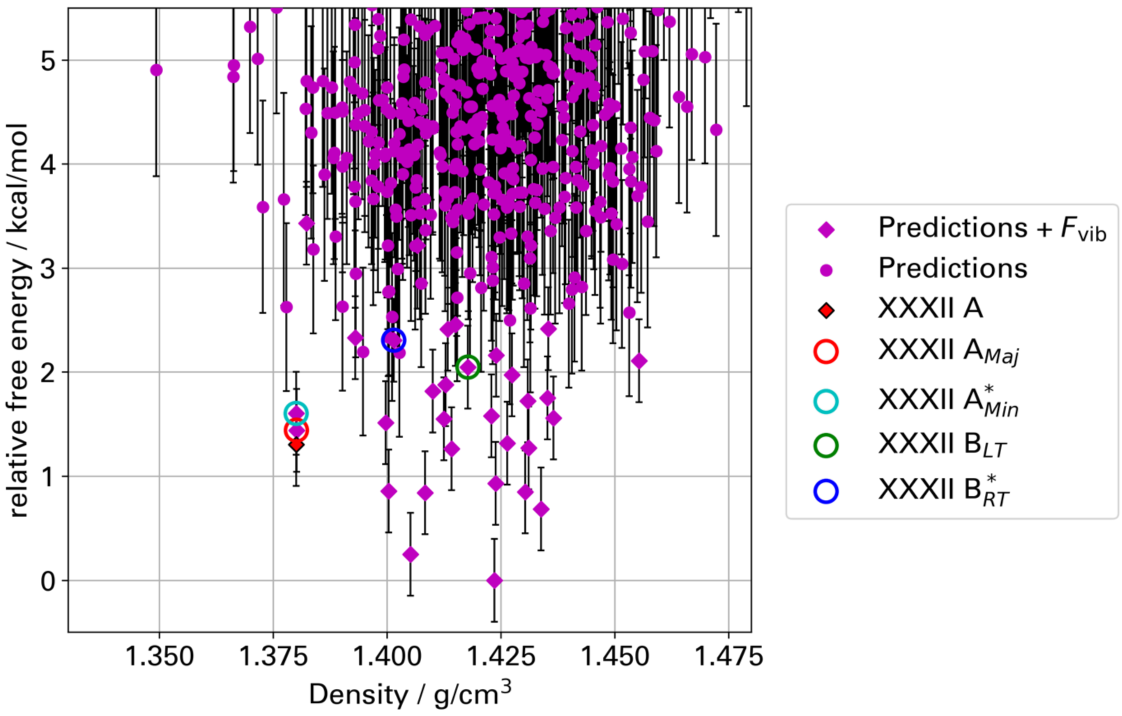

Figure 8. Energy landscape for compound XXXII.

The calculated temperature-dependent free energies for the LT and the corrected RT form of Form B show the correct enantiotropic behaviour (Figure 9).

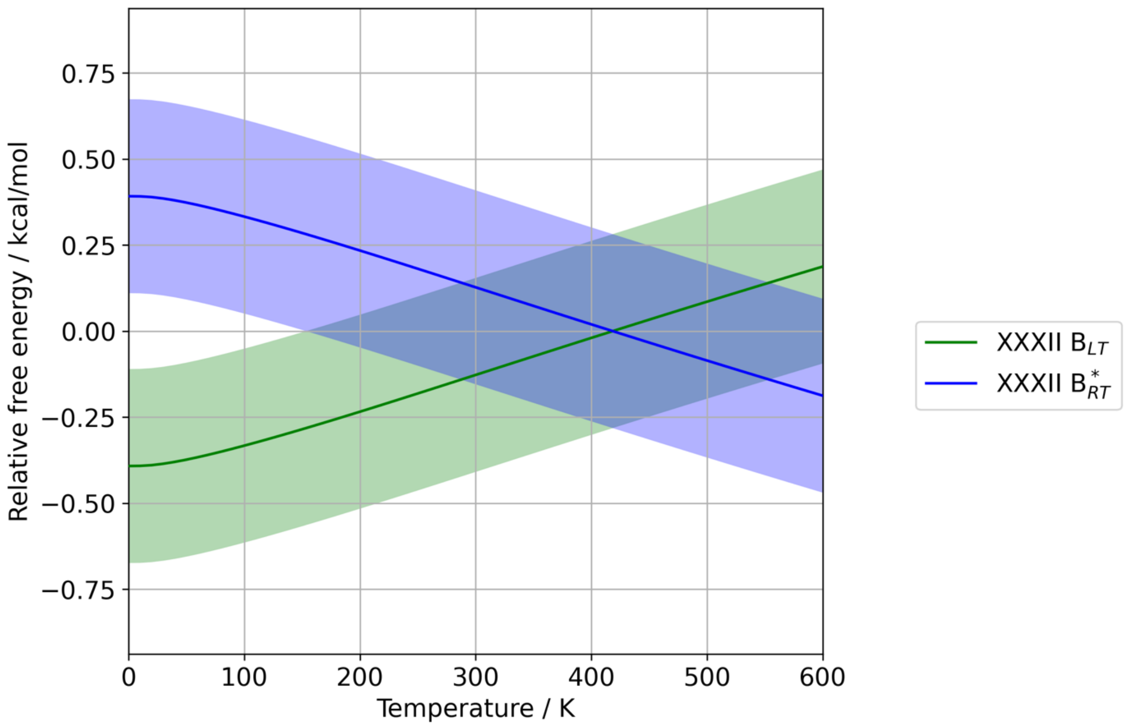

Figure 9. Enantiotropic stability relationship of the two forms of XXXII Form B as calculated with our TRHu(ST) method.

For the ranking stage, we use a cascade of increasingly more accurate energy methods with strict convergence control only for the global minimum: only the global minimum itself is guaranteed to have been treated with our most accurate energy method. Any rank other than the global minimum is treated just accurate enough to decide if within the error bar of the method used for that rank, it stands a realistic chance of becoming the global minimum if we were to treat it at the highest accuracy level. For compound XXXII, form A was treated at the highest level of accuracy, but by the time form B was processed, the distance to the global minimum was greater than 3 times the standard deviation and form B was treated at a less accurate level of theory. From these energies we therefore cannot conclude if Form A or Form B is calculated to be more stable at room temperature.

For XXXIII (the salt), the experimental structures are rank 1 and rank 2 (Figure 10) with the TRHu(ST) method.

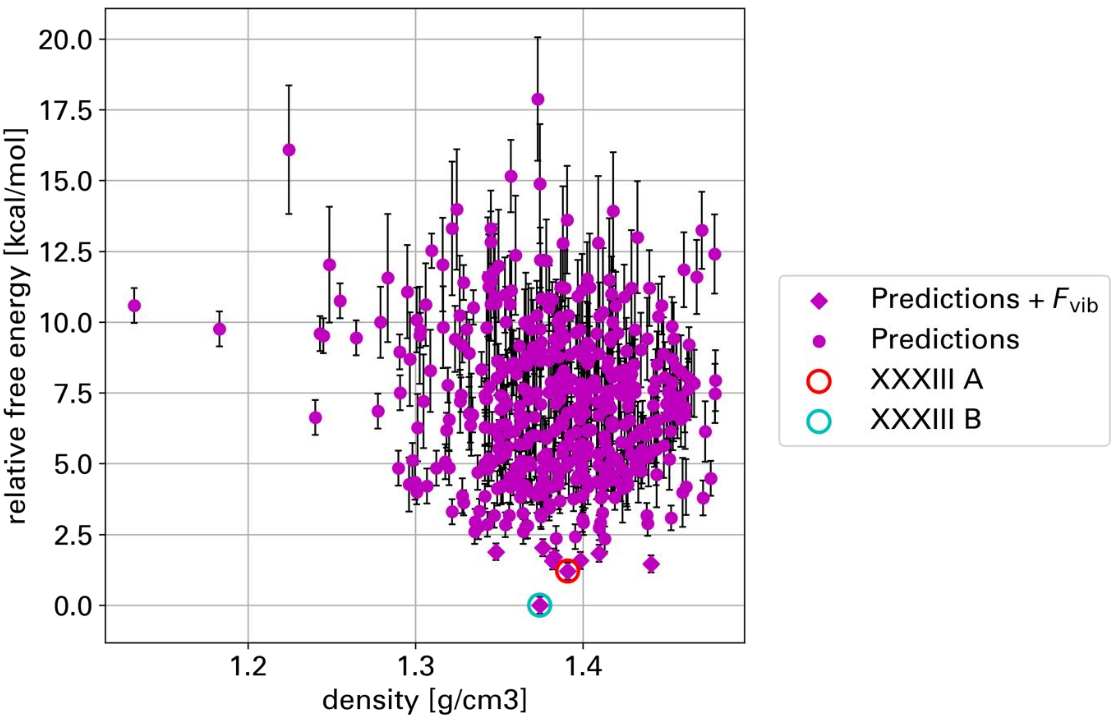

Figure 10. Energy landscape for compound XXXIII.

In summary, we ranked the experimental structures of XXVII, XXVIII, XXIX, XXX 1:1, XXX 2:1 and XXXIII as rank 1; the second polymorphs of XXX 1:1 and XXXIII we ranked as rank 2.

In our paper "How many Ritonavir cases are there still out there?" (Neumann & Van de Streek, 2018) we predict that for between 15% to 45% of all chemical compounds even after a commercial polymorph screen the thermodynamically most stable form has not yet been found because it is kinetically hindered. Counting the 1:1 and the 2:1 co-crystals as two different compounds, there were eight compounds in this blind test, and 30% of eight compounds = 2.4 compounds. We therefore predict that in this blind test, for *circa* two compounds the thermodynamically most stable structure has not yet been experimentally observed because it is kinetically hindered. Furthermore, the smaller the molecule, the easier it is to crystallise (Wicker & Cooper, 2015), so that it would be the larger and more flexible compounds XXXI and XXXII that turn out to be kinetically hindered is hardly surprising.

In our hierarchical approach, the most accurate energy calculations for compounds XXXI, XXXII and XXXIII were performed with the now published TRHu(ST) method (Firaha *et al*., 2023). Variants or precursors thereof were applied to all other compounds. The molecular-size-dependent error of the TRHu(ST) method has been carefully assessed against a large body of experimental free energy differences. For compounds, XXXI, XXXII and XXXIII the expected accuracy of lattice energy calculations per molecule for *Z'*=1 structures is 1.1 kJ/mol, 1.7 kJ/mol and 1.3 kJ/mol, respectively. The good accuracy of the method is illustrated by the fact that it places the experimental forms at the very bottom of the corresponding crystal energy landscapes of compounds XXVII (part 2, post analysis), XXVIII (part II), XXIX (part 1), XXX 1:1 (part 1, two polymorphs as ranks 1 and 2), XXX 2:1 (part 1) and XXXIII (part 2, two polymorphs as ranks 1 and 2). Errors on lattice energy differences are $\sqrt{2}$ times larger than errors on individual energies, and the change required to get the right stability ranking of forms A and B of compound XXXI amounts to one standard deviation. For compound XXXII,

the bottom of the energy window lies 2.5 standard deviations below the lowest calculated lattice energy for an experimental structure. According to the error bars of the TRHu(ST) method, it is likely that the thermodynamically stable form of compound XXXII has not been observed yet. For compound XXXI, the bottom of the energy window lies about 0.8 standard deviations below the lowest calculated lattice energy for an experimental structure, and it cannot be excluded that also for this compound the thermodynamically stable form is still missing. The finding that for 1 or 2 out of 8 compounds the most stable form may not have been observed yet even after experimental screening is in line with previous statistical analysis (Neumann & van de Streek, 2018).

In summary, assuming that for about 30% of all compounds the experimentally most stable structure is kinetically hindered and taking into account the carefully calibrated error bars of our energy method, the computational results of Group 20 and the experimental results are in full agreement.

Blum, V., Gehrke, R., Hanke, F., Havu, P., Havu, V., Ren, X., Reuter, K. & Scheffler, M. (2009). *Comput. Phys. Commun*. **180**, 2175−2196.

Firaha, D., Liu, Y. M., Van de Streek, J., Sasikumar, K., Dietrich, H., Helfferich, J., Aerts, L., Braun, D. E., Broo, A., DiPasquale, A. G., Lee, A. Y., Le Meur, S., Nilsson Lill, S. O., Lunsmann, W. J., Mattei, A., Muglia, P., Putra, O. D., Raoui, M., Reutzel-Edens, S., Rome, S., Sheikh, A. Y., Tkatchenko, A., Woollam, G. R. & Neumann, M. A. (2023). *Nature* **623**, 324-328.

Hermann, J. & Tkatchenko, A. (2020). *Phys. Rev. Lett*. **124**, 146401

Kresse, G. & Furthmüller, J. (1996). *Phys. Rev. B* **54**, 11169−11186.

Kresse, G. & Hafner, J. (1993). *Phys. Rev. B* **47**, 558−561.

Kresse, G. & Joubert, D. (1999). *Phys. Rev. B* **59**, 1758−1775.

Mortazavi, M., Hoja, J., Aerts, L., Quéré, L., Van de Streek, J., Neumann, M. A. & Tkatchenko, A. (2019). *Commun*. *Chem*. **2**, doi:10.1038/s42004-019-0171-y.

Neumann, M. A. (2008). *J. Phys. Chem. B* **112**, 9810−9829.

Neumann, M. A. & Perrin, M.-A. (2005). *J. Phys. Chem. B* **109**, 15531–15541.

Neumann, M. A. & Van de Streek, J. (2018). *Faraday Discuss*. **211**, 441-458.

Řezáč, J., Greenwell, C. & Beran, G. J. O. (2018). *J. Chem. Theory Comput.* **14**, 4711−4721.

Wicker, J. G. P. & Cooper, R. I. (2015). *CrystEngComm* **17**, 1927−1934.

18. Group 21

Supporting Information for Phase 2

Authors of submission for XXVII:

Shigeaki Obata[1,2], Yasuhiro Ikabata[2], and Hitoshi Goto[1,2]

Authors of submission for XXXI, XXXII, and XXXIII

Shigeaki Obata[1,2], Yohei Utsumi[3], Yasuhiro Ikabata[2], Koji Okuwaki[3,4], Naofumi Nakayama[1], Kaori Fukuzawa[3,4], Etsuo Yonemochi[3], and Hitoshi Goto[1,2]

[1] CONFLEX Corporation, Shinagawa Center Bldg. 6F, 3-23-17 Takanawa, Minato-ku, Tokyo 108-0074, Japan.

[2] Information and Media Center, Toyohashi University of Technology, 1-1 Hibarigaoka, Tempaku-cho, Toyohashi, Aichi 441-8580, Japan.

[3] School of Pharmacy and Pharmaceutical Sciences, Hoshi University, 2-4-41 Ebara, Shinagawa-ku, Tokyo 142-8501, Japan.

[4] Graduate School of Pharmaceutical Sciences, Osaka University 1-6 Yamadaoka, Suita, Osaka 565-0871, Japan.

E-mail: obata@conflex.co.jp, gotoh@tut.jp, fukuzawa-k@phs.osaka-u.ac.jp

Overview of prediction methods

Structure determination:

The 500 crystal structures of XXXII were optimized by own method [1, 2] and MMFF94s potential [3] under *P*1 space group symmetry. The van der Waals and electrostatic interactions were calculated by the cutoff and Ewald summation [4] techniques, respectively, with a real space cutoff of 20 Å. The optimizations were carried out using the full diagonal Newton-Raphson method. For the 500 optimized crystal structures, the root-mean-square deviation of gradients over all parameters converged to better than 1.0×10^{-6} kcal/mol/Å and the root-mean-square deviation of displacements of the parameters converged to better than 1.0×10^{-6} Å. The optimized crystal structures were confirmed whether they have no imaginary frequencies by performing a normal mode analysis.

The 100, 100, and 500 crystal structures of XXVII, XXXI, and XXXIII, respectively, were optimized by QUANTUM ESPRESSO (QE) V.6.7 [5, 6] using GGA-PBE exchange-correlation functional [7], D3 dispersion correction [8], and scalar relativistic ultrasoft pseudopotential [9] (PBE-D3) under periodic boundary condition. We used kinetic energy cutoffs of 50 Ry and 500 Ry for the wavefunction and charge density, respectively, and the *k*-point mesh spacing was set to about 0.30 $Å^{-1}$. The convergence thresholds on total energy and forces for ionic minimization and on pressure for variable cell relaxation were set to 1.0×10^{-5} a.u, 1.0×10^{-4} a.u, and 5.0×10^{-2} kbar, respectively. CIF2Cell software was used for making input files of QE [10].

In the optimization, all degrees of freedom for representing the crystal structure, that is, atomic positions and unit cell dimensions were relaxed with restriction of specified space group symmetry. Adequate space groups and lattice constant parameters of the optimized crystal structures were defined using the software PLATON [11] or Materials Studio 2018 [12]. Identical structures among all optimized crystal structures were confirmed using Crystal Packing Similarity Tool in the CSD Python API [13]. Some structures became identical to other structures due to the structure optimization. The pairs of structures are shown in each target's section. Data of the duplicate structure in the material for "structure specific data" are set to blank.

Structure ranking:

Table 1 summarizes determination and ranking methods for crystal structures of each target. The relative energy and rank of the crystal structures were determined according to their absolute lattice energy per molecule, with respect to isolated gas-phase molecule in the lowest energy conformation. The absolute lattice energy of XXVII was estimated by PBE-D3 scheme and those of XXXI, XXXII, and XXXIII were estimated by fragment molecular orbital (FMO) method [14] at the MP2 level of theory (FMO-MP2) [15-18]. The absolute lattice energy is calculated by subtracting energy of the isolated gas-phase molecule from final energy of the optimized crystal structure. Detail of determination method of the final energy of optimized crystal structure and the energy of isolated gas-phase molecule for each target is described in each target's section.

Table 1. Summary of determination and ranking methods for crystal structures of each target.

Target	Structure	Ranking
XXVII	PBE-D3	PBE-D3 energy
XXXI	PBE-D3	FMO-MP2/6-31G† energy
XXXII	MMFF94s	FMO-MP2/6-31G† energy
XXXIII	PBE-D3	FMO-MP2/6-31G(d) energy

To apply FMO method [14] to a molecular crystal with periodic structure, we employ a molecular cluster model in which molecules within a specified crystal radius of central molecule are cut out from the molecular crystal [17, 18]. The asymmetric unit is used as the center molecule. The fragmentation in FMO method [14] is performed to the molecular cluster. In FMO calculation, electron densities and energies of each fragment are calculated considering contributions from other fragments by environment electrostatic potential until the electron densities of each fragment become self-consistent, which is called as self-consistent charge (SCC) process, and them of all fragment pairs are also calculated [14, 19]. FMO energy of the molecular cluster is obtained by the energies of each fragment and fragment pair, and it is defined by

$$E_{\mathrm{crystal}} = E_{\mathrm{conf}} + \frac{1}{2}\alpha \sum_{I,J\neq I} \Delta\tilde{E}_{IJ} \qquad (1)$$

where the E_{conf} is the sum of intramolecular energies of one fragment, that is, one asymmetric unit, the second term is the sum of intermolecular interaction energies per one fragment, and the alpha is a scaling factor. In this work, the scaling factor was determined by comparing reference lattice energies [20] and the absolute lattice energies estimated by FMO-MP2 scheme, using X23 database [21] excluding ammonia and carbon dioxide molecules, and it was set to 0.70 and 0.75 for estimating PBE-D3 and MMFF94s structures, respectively.

In the material for “structure specific data”, the “N/A” means that FMO calculation couldn't estimate energy of the crystal structure because the step to calculate the SCC process was not converged.

Target XXVII

Structure determination:

XXVII_structure_61 and XXVII_structure_28 became identical in the structure optimization.

Structure ranking:

The 100 optimized crystal structures were subjected to energy calculation by PBE-D3 scheme with kinetic energy cutoffs of 80 Ry and 800 Ry for the wavefunction and charge density, respectively, and the k-point mesh spacing of about 0.20 Å^{-1}, and we determined their final energies.

The 171 conformers were obtained within 1.5 kcal/mol from the global minimum by performing the conformation search using own method [22, 23] and MMFF94s potential [3]. They were re-optimized by Gaussian 16 Rev. C.01 [24] using M06-2X functional with 6-31G(d) and DGDZVP basis sets for H, C,

and Si atoms and for I atom, respectively. The conformer with the lowest energy in M06-2X calculations was selected and arranged in cubic cell with 40 Å in length. The system containing one molecule was subjected to geometry optimization with fixed lattice parameters by PBE-D3 scheme with the same conditions about kinetic energy cutoffs and convergence thresholds on total energy and forces used for the optimization of 100 crystal structures. The energy of the system optimized was determined by PBE-D3 scheme with kinetic energy cutoffs of 80 Ry and 800 Ry for the wavefunction and charge density, respectively, and it was used as the energy of the isolated gas-phase molecule (Figure 1). In the determinations of structure and energy of the conformer, the *k*-point mesh only sampled the gamma point of the Brillouin zone.

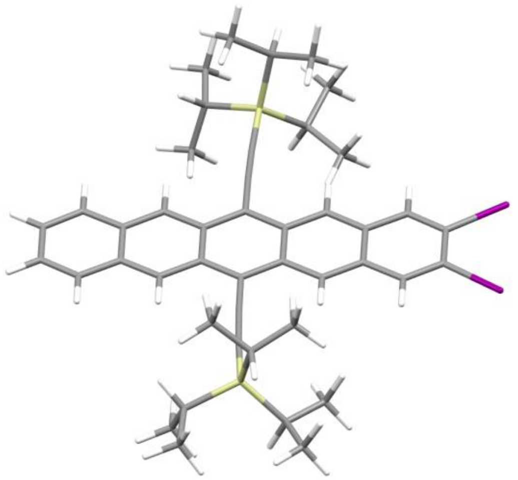

Figure 1. The lowest energy conformation of XXVII obtained by stepwise calculations with MMFF94s, M06-2X, and PBE-D3 schemes.

Target XXXI

Structure determination:

The structure of #86 with $P2_1/c$ was optimized under $P1$ space group symmetry because its initial lattice parameter does not satisfy the rule for monoclinic crystal system. XXXI_structure_17 and XXXI_structure_59 became identical in the structure optimization.

Structure ranking:

The 100 optimized crystal structures were subjected to energy calculation by FMO-MP2 with 6-31G† basis set [25, 26], and FMO energies were used as the final energies of them. In FMO calculation, the molecular clusters with crystal radius of 12 Å were used, and one molecule was treated as one fragment. FMO calculation couldn't estimate energies of the structures of #53, #66, and #87 due to the problem of convergence in some fragment pair energies. Therefore, we employed three structures optimized by PBE-D3 scheme with loose convergence thresholds (Each threshold was set to 1.0×10^{-4} a.u, 1.0×10^{-3} a.u, and 5.0×10^{-1} kbar, respectively.) in FMO calculation, resulting in getting FMO energies for the structures of #53 and #87. The energy of structure #66 couldn't be finally estimated by FMO calculation.

The 11 conformers were obtained by performing the conformation search using own method [22, 23] and MMFF94s potential [3]. They were re-optimized by Gaussian 16 Rev. C.01 [24] using MP2 level of theory with 6-31G(d) basis set. The conformer with the lowest energy in MP2 calculations was selected

and arranged in cubic cell with 30 Å in length. The system containing one molecule was subjected to geometry optimization with fixed lattice parameters by PBE-D3 scheme with the same conditions about kinetic energy cutoffs and convergence thresholds on total energy and forces used for the optimization of 100 crystal structures. The *k*-point mesh only sampled the gamma point of the Brillouin zone. The energy of the conformer optimized by PBE-D3 scheme (Figure 2) was determined by FMO-MP2 with 6-31G† basis set [25, 26], and it was used as the energy of the isolated gas-phase molecule.

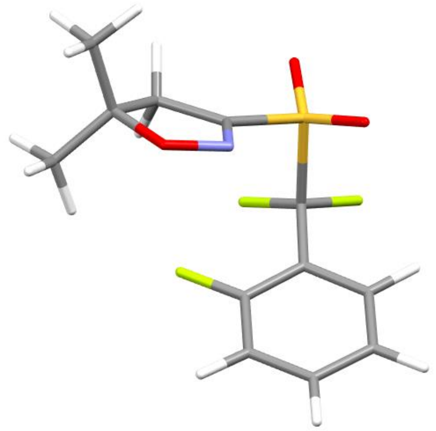

Figure 2. The lowest energy conformation of XXXI obtained by stepwise calculations with MMFF94s, MP2, and PBE-D3 schemes.

Target XXXII

Structure determination:

The pairs of structures that became identical in the structure optimization are shown below.

XXXII_structure_1 - XXXII_structure_58

XXXII_structure_66 - XXXII_structure_58

XXXII_structure_154 - XXXII_structure_105

XXXII_structure_156 - XXXII_structure_386

XXXII_structure_237 - XXXII_structure_27

XXXII_structure_300 - XXXII_structure_31

XXXII_structure_311 - XXXII_structure_91

XXXII_structure_442 - XXXII_structure_58

XXXII_structure_450 - XXXII_structure_60

XXXII_structure_485 - XXXII_structure_105

XXXII_structure_488 - XXXII_structure_169

Structure ranking:

The 500 optimized crystal structures were subjected to energy calculation by FMO-MP2 with 6-31G† basis set [25, 26], and FMO energies were used as the final energies of them. In FMO calculation, the molecular clusters with crystal radius of 10 Å were used, and one molecule was treated as two fragments (Figure 3). FMO calculation couldn't estimate energies of the 51 crystal structures (#5, #21, #30, #48, #50, #52, #79, #104, #135, #136, #157, #167, #185, #188, #208, #219, #221, #222, #223, #232, #239, #246, #247, #265, #271, #286, #296, #302, #310, #318, #321, #323, #334, #340, #343, #352, #353, #356, #358, #365, #370, #372, #401, #409, #421, #425, #437, #440, #443, #470, and #498) due to the problem of convergence in SCC process.

The 2,698 conformers were obtained within 10 kcal/mol from the global minimum by performing the conformation search using own method [22, 23] and MMFF94s potential [3]. The conformer with the lowest energy on MMFF94s potential (Figure 3) was subjected to energy calculation by FMO-MP2 with 6-31G† basis set [25, 26], and it was used as the energy of the isolated gas-phase molecule.

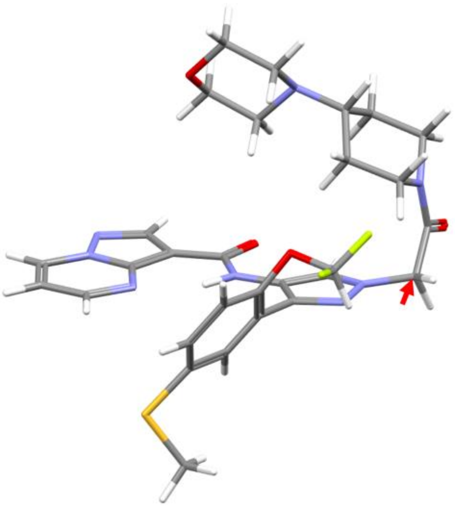

Figure 3. The lowest energy conformation of XXXII obtained by MMFF94s calculation. The red arrow indicates bond detached atom.

Post analysis:

We confirmed that FMO calculations using the 6-31G basis set with one molecule treated as a single fragment and the Anderson's SCC algorithm [27] resulted in SCC convergence for all crystal structures.

Target XXXIII

Structure determination:

The structures of #171 with $C2/c$ and #409 with $P2_1/n$ were optimized under $P1$ space group symmetry because their initial lattice parameters do not satisfy the rule for monoclinic crystal system. The pairs of structures that became identical in the structure optimization are shown below.

XXXIII_structure_148 - XXXIII_structure_462

XXXIII_structure_204 - XXXIII_structure_268

XXXIII_structure_210 - XXXIII_structure_388

XXXIII_structure_218 - XXXIII_structure_387

XXXIII_structure_265 - XXXIII_structure_103

XXXIII_structure_333 - XXXIII_structure_471

XXXIII_structure_338 - XXXIII_structure_440

XXXIII_structure_339 - XXXIII_structure_396

XXXIII_structure_415 - XXXIII_structure_426

XXXIII_structure_456 - XXXIII_structure_329

Structure ranking:

The 500 optimized crystal structures were subjected to energy calculation by FMO-MP2 with 6-31G(d) basis set. In FMO calculation, the molecular clusters with crystal radius of 12 Å were used, and one molecule was treated as one fragment. The relative energy and rank of the crystal structures were determined according to absolute lattice energy per ionic pair in case of XXXIII. FMO calculation couldn't estimate energies of the 5 crystal structures of #171, #214, #409, #430, and #497 due to the problem of convergence in SCC process.

The 6 conformers of sulfamethoxazole anion and the 4 conformers of morpholinium were obtained by the conformation search using own method [22, 23] and MMFF94s potential [3]. They were re-optimized by Gaussian 16 Rev. C.01 [24] using MP2 level of theory with 6-31G(d) basis set. Each conformer of sulfamethoxazole anion and morpholinium with the lowest energy in MP2 calculations was selected and arranged in cubic cell with 30 Å in length, respectively. Each system containing one molecule was subjected to geometry optimization with fixed lattice parameters by PBE-D3 scheme with the same conditions about kinetic energy cutoffs and convergence thresholds on total energy and forces used for the optimization of 500 crystal structures, respectively. The *k*-point mesh only sampled the gamma point of the Brillouin zone. Each energy of the conformers optimized by PBE-D3 scheme (Figure 4) was determined by FMO-MP2 with 6-31G(d) basis set, respectively, and they were used as the energies of the isolated gas-phase molecules.

Post analysis:

We confirmed that in the FMO calculations using the Anderson's algorithm [27], all five of these crystal structures converged to SCC. The energy ranks of these structures were in the lower 400s.

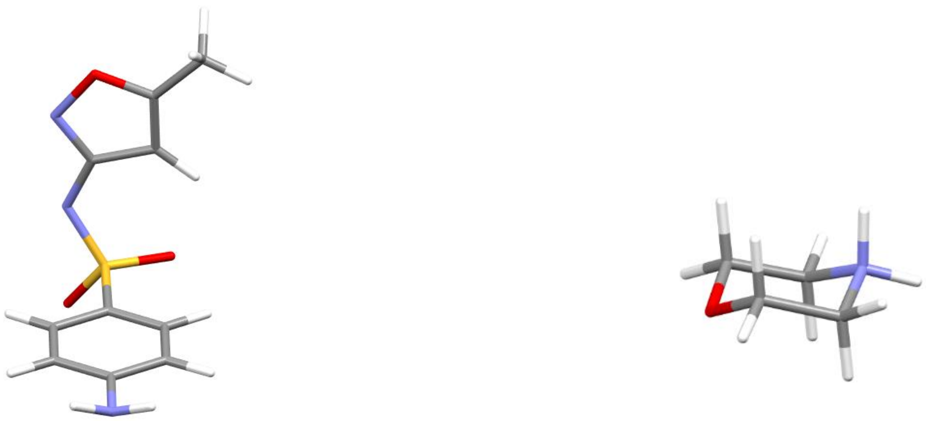

(a) (b)

Figure 4. The lowest energy conformations of (a) sulfamethoxazole anion and (b) morpholinium obtained by stepwise calculations with MMFF94s, MP2, and PBE-D3 schemes.

Computational costs

Table 2. Summary of computational costs.

Target	CPU time (h)[a]		
	MMFF94s calc.	PBE-D3 calc.	FMO calc.
XXVII	---	163,278	---
XXXI	---	44,854	596,021
XXXII	160	---	4,361,580
XXXIII	---	401,734	1,753,522

[a] The CPU time was normalized into that when using the CPU shown in the table 3.

Table 3. Summary of computational resources.

Calculation	Resource
MMFF94s calc.	Intel Xeon Gold 6258R CPU 2.70 GHz
PBE-D3 calc.	Intel Xeon Gold 6154 CPU 3.00 GHz
FMO calc.	FUJITSU A64FX CPU 2.2 GHz

Author contributions

S.O. and H.G. conceived and designed the work of XXVII. S.O. and H.G. conceived and designed the works of XXXI, XXXII, and XXXIII with K.F. and E.Y. S.O. and Y.I. determined the structures of crystal and isolated gas-phase molecule for each target and PBE-D3 energy of XXVII. Y.U. and K.O. performed FMO calculations. N.N. helped with FMO calculation. Authors of submissions for each target analyzed the results for the corresponding target. S.O. and K.F. wrote the SI and all authors approved the final version.

Acknowledgements

In this work, we used the computer resources by Research Institute for Information Technology, Kyushu University, ACCMS, Kyoto University, and Information and Media Center, Toyohashi University of Technology. Part of this work used computational resources of Fugaku supercomputer through the HPCI System Research Project (Project ID: hp220143). The FMO calculations were performed in the activities of the FMO drug design consortium (FMODD). This work was supported by JSPS KAKENHI Grant Numbers 17H06373 (H.G.), 21K05002 (Y.I.), and 21K05105 (N.N.).

References

[1] Obata, S. & Goto, H. (2015). *AIP Conf. Proc.* **1649**, 130–134.

[2] Ishii, H., Obata, S., Niitsu, N., Watanabe, S., Goto, H., Hirose, K., Kobayashi, N., Okamoto, T. & Takeya, J. (2020). *Sci. Rep.* **10**, 2524.

[3] Halgren, T. A. (1996). *J. Comput. Chem.* **17**, 490–519.

[4] Ewald, P. P. (1921). *Ann. Phys.* **369**, 253.

[5] Giannozzi, P., Baroni, S., Bonini, N., Calandra, M., Car, R., Cavazzoni, C., Ceresoli, D., Chiarotti, G. L., Cococcioni, M., Dabo, I., Dal Corso, A., de Gironcoli, S., Fabris, S., Fratesi, G., Gebauer, R., Gerstmann, U., Gougoussis, C., Kokalj, A., Lazzeri, M., Martin-Samos, L., Marzari, N., Mauri, F., Mazzarello, R., Paolini, S., Pasquarello, A., Paulatto, L., Sbraccia, C., Scandolo, S., Sclauzero, G., Seitsonen, A. P., Smogunov, A., Umari, P. & Wentzcovitch, R. M. (2009). *J. Phys. Condens. Matter* **21**, 395502.

[6] Giannozzi, P., Andreussi, O., Brumme, T., Bunau, O., Buongiorno Nardelli, M., Calandra, M., Car, R., Cavazzoni, C., Ceresoli, D., Cococcioni, M., Colonna, N., Carnimeo, I., Dal Corso, A., de Gironcoli, S., Delugas, P., DiStasio, R A., Ferretti, A., Floris, A., Fratesi, G., Fugallo, G., Gebauer, R., Gerstmann, U., Giustino, F., Gorni, T., Jia, J., Kawamura, M., Ko, H.-Y., Kokalj, A., Kucukbenli, E., Lazzeri, M., Marsili, M., Marzari, N., Mauri, F., Nguyen, N. L., Nguyen, H.-V., Otero-de-la-Roza, A., Paulatto, L., Ponce, S., Rocca, D., Sabatini, R., Santra, B., Schlipf, M., Seitsonen, A. P., Smogunov, A., Timrov, I., Thonhauser, T., Umari, P., Vast, N., Wu, X. & Baroni, S. (2017). *J. Phys. Condens. Matter* **29**, 465901.

[7] Perdew, J. P., Burke, K. & Ernzerhof, M. (1996). *Phys. Rev. Lett.* **77**, 3865–3868.

[8] Grimme, S., Antony, J., Ehrlich, S. & Krieg, H. (2010). *J. Chem. Phys.* **132**, 154104.

[9] Dal Corso, A. (2014). *Comput. Mater. Sci.* **95**, 337–350.

[10] Björkman, T. (2011). *Comput. Phys. Commun.* **182**, 1183–1186.

[11] Spek, A. L. (2009). *Acta Cryst.* **D65**, 148–155.

[12] BIOVIA, Dassault Systèmes, BIOVIA Materials Studio 2018, San Diego: Dassault Systèmes (2017).

[13] Groom, C. R., Bruno, I. J., Lightfoot, M. P. & Ward, S. C. (2016). *Acta Cryst.* **B72**, 171–179.

[14] Kitaura, K., Ikeo. E., Asada, T., Nakano, T. & Uebayasi, M. (1999). *Chem. Phys. Lett.* **313**,701–706.

[15] Mochizuki, Y., Nakano, T., Koikegami, S., Tanimori, S., Abe, Y., Nagashima, U. & Kitaura, K. (2004). *Theor. Chem. Acc.* **112**, 442–452.

[16] Mochizuki, Y., Koikegami, S., Nakano, T., Amari, S. & Kitaura, K. (2004). *Chem. Phys. Lett.* **396**(4-6), 473–479.

[17] Utsumi, Y., Umeda, D., Okuwaki, K., Obata, S., Nakayama, N., Goto, H., Furuishi, T., Fukuzawa, K. & Yonemochi, E. (2021). *J. Comput. Chem. Jpn.* **20**, 92–93.

[18] Utsumi, Y., Okuwaki, K., Umeda, D., Obata, S., Nakayama, N., Goto, H., Furuishi, T., Fukuzawa, K. & Yonemochi, E. to be submitted.

[19] Nakano, T., Kaminuma, T., Sato, T., Fukuzawa, K., Akiyama, Y., Uebayasi, M. & Kitaura, K. (2002). *Chem. Phys. Lett.* **351**(5-6), 475–480.

[20] Dolgonos, G. A., Hoja, J. & Boese, A. D. (2019). *Phys. Chem. Chem. Phys.*, **21**, 24333-24344.

[21] Reilly, A. M. & Tkatchenko, A. (2013). *J. Chem. Phys.* **139**, 024705.

[22] Goto, H. & Osawa, E. (1989). *J. Am. Chem. Soc.* **111**, 8950–8951.

[23] Goto, H. & Osawa, E. (1993). *J. Chem. Soc., Perkin Trans.* **2**, 187–198.

[24] Gaussian 16, Revision C.01, Frisch, M. J., Trucks, G. W., Schlegel, H. B., Scuseria, G. E., Robb, M. A., Cheeseman, J. R., Scalmani, G., Barone, V., Petersson, G. A., Nakatsuji, H., Li, X., Caricato, M., Marenich, A. V., Bloino, J., Janesko, B. G., Gomperts, R., Mennucci, B., Hratchian, H. P., Ortiz, J. V., Izmaylov, A. F., Sonnenberg, J. L., Williams-Young, D., Ding, F., Lipparini, F., Egidi, F., Goings, J., Peng, B., Petrone, A., Henderson, T., Ranasinghe, D., Zakrzewski, V. G., Gao, J., Rega, N., Zheng, G., Liang, W., Hada, M., Ehara, M., Toyota, K., Fukuda, R., Hasegawa, J., Ishida, M., Nakajima, T., Honda, Y., Kitao, O., Nakai, H., Vreven, T., Throssell, K., Montgomery, J. A., Jr., Peralta, J. E., Ogliaro, F., Bearpark, M. J., Heyd, J. J., Brothers, E. N., Kudin, K. N., Staroverov, V. N., Keith, T. A., Kobayashi, R., Normand, J., Raghavachari, K., Rendell, A. P., Burant, J. C., Iyengar, S. S., Tomasi, J., Cossi, M., Millam, J. M., Klene, M., Adamo, C., Cammi, R., Ochterski, J. W., Martin, R. L., Morokuma, K., Farkas, O., Foresman, J. B. & Fox, D. J. Gaussian, Inc., Wallingford CT, 2016.

[25] Petersson, G. A., Bennett, A., Tensfeldt, T. G., Al-Laham, M. A., Shirley, W. A. & Mantzaris, J. (1988). *J. Chem. Phys.* **89**, 2193–2218.

[26] Petersson, G. A. & Al-Laham, M. A. (1991). *J. Chem. Phys.* **94**, 6081–6090.

[27] Anderson, D. G. (1965). *J. ACM*, **12**, 547-560.

19. Group 22

Supplementary Information (Structure ranking) – Group 22
Oganov*, Maryewski, Momenzadeh Abardeh, Bahrami, Salimi

1. Structure generation.

For all targets except XXX (stoichiometry prediction) we presented results of two approaches in the 85/15 ratio. The 85% were obtained using an experimental version of USPEX code with symmetry-preserving variation operators for molecular crystals, and structures are given together with their energies relative to the ground state. The remaining 15% are the lowest-energy structures produced with the public version of the USPEX code – these structures were given as a separate block; since these calculations used a different force field, to avoid confusion these structures were listed without energies. Results for target XXX (stoichiometry prediction) were produced entirely using the latter methodology (public version of USPEX code and relaxations done using GULP and DREIDING force field with charges from Qeq procedure). To generate structures, we considered the five fixed compositions (1:1, 1:2, 2:1, 2:3, 3:2). The generated structures were ranked according to the energy of each structure/composition relative to the convex hull, which is the thermodynamically rigorous way of judging stability of compounds.

Let us describe the two approaches in more detail. The main features of the experimental version of USPEX, compared to an older version, are the space-group-aware heredity and mutation operators, which were previously not available. These operators, by design, preserve the space group of the structure(s) they act upon, which incurs the cost of being able to span only the region of phase space corresponding to the parent structure(s) space group – as a consequence, we had to perform a separate global optimization run for each space group that we wanted to consider. The initial random generation of structures was produced using the PyXtal library [10.1016/j.cpc.2020.107810]. For each target we chose 10-15 most common space groups according to CCDC statistics (for harder cases like target XXVII we used even fewer space groups). We did not explicitly consider point group symmetry of the molecules, therefore all molecules in all structures occupy the general position of the corresponding space group. Z' value was assumed to equal 1 everywhere, except for targets XXIX, XXXI, XXXII, where we also considered Z'=2.
For the global optimization run, we made a decision to use a realistic yet simple model, which led us to use the DREIDING force field together with Mulliken charges obtained from a single-point vacuum calculation for each considered molecule. Then, using this model, each structure in the global optimization run was carefully relaxed using the GULP code [10.1080/0892702031000104887]. Global optimization stopped when the best found structure did not change for 10 generations.

When doing structure prediction using the public version of USPEX, we again considered only the most common space groups – for each of them, we performed a random sampling run with all molecules assumed to occupy the general position of each structure's space group. For this part, a different force field was used, namely AMOEBA.

2. **Structure ranking.**

Here, we used a two-step strategy. First, the synthon approach was used to investigate the extracted CSD-structures of similar systems. Having established the most likely synthons, we analyzed different methods of energy ranking.

In the second step, we used the synthon approach to decide which energy ranking (PBE-D3, MBD-PBE, MBD-PBE0) to trust more. Using VASP code, we re-relaxed the structures using PBE-D3, MBD-PBE and MBD_PBE0 approximations. Analyzing the results we found that PBE-D3 is the best approach, placing the structures containing the expected synthons in top ranks. Our final results show PBE-D3 energy ranking. In PBE-D3 calculations, we used PAW potentials, 700 eV plane-wave kinetic energy cutoff. For Brillouin zone sampling, we used uniform grids with reciprocal-space resolution of 0.6 $Å^{-1}$.

20. Group 24

Detailed methodology for 24-SLPrice-CSPBT7 Submission 2

1 Overview

The Price group have generally used the Ψ_{mol} approach, based on the electronic structure of the molecule, including:

- Assessing the provided crystal structures and relabelling atoms according to our internal numbering scheme (this was done to ensure that previously generated databases of *ab initio* data points could be reused)
- Minimizing the lattice energy of all provided crystal structures using the final energy model used in Submission 1
- Assessing the reliability of this energy model and making improvements as seemed necessary.

In all cases, the molecular conformation and intramolecular energy penalty was calculated using GAUSSIAN09,[1] the conformation specific charge density was calculated in GAUSSIAN09[1] and represented by atomic distributed multipoles up to hexadecapole (for use in DMACRYS[2]) calculated using GDMA2.[3] Unless otherwise stated, exp-6 repulsion-dispersion parameters were taken from the FIT potential[4] including polar hydrogen parameters from Coombes's work on polar organic molecules,[5] chlorine parameters from Williams's work on perchlorohydrocarbons[6] and fluorine parameters from Williams's work on perfluorohydrocarbons.[7] Parameters for other potential types are described for each system. The form of the repulsion-dispersion potential between atom *i* in molecule *M* of type ι and atom *k* in molecule N of type κ separated by distance R_{ik} is given in Equation 1.

$$U = \sum_{i \in M, k \in N} \sqrt{A_{\iota\iota} A_{\kappa\kappa}} \exp\left(-\left(\frac{B_{\iota\iota} + B_{\kappa\kappa}}{2}\right) R_{ik}\right) - \frac{\sqrt{C_{\iota\iota} C_{\kappa\kappa}}}{R_{ik}^6}$$

Equation 1.

The total lattice energy was calculated according to Equation 2, where U_{inter} is the intermolecular energy of the crystal relative to the molecules being infinitely separated, calculated by lattice summation of the repulsion-dispersion terms in Equation 1 and the electrostatic contribution to the lattice energy for all terms in the multipole expansion up to R^{-5}, and ΔE_{intra} is the conformational energy penalty with respect to the gas phase optimized conformation.

Equation 2. $$E_{latt} = U_{inter} + \Delta E_{intra}$$

CrystalOptimizer[8] v2.4.7 was usually used (XXXI, XXXII), while v2.4.8 was used for systems which included PCM correction of the *ab initio* calculations (XXVIII, XXXIII). DMACRYS[2] v2.3.1.1 was used throughout.

The free energy model used rigid molecule supercell phonon frequencies for all systems. These were calculated using AutoFree and AutoLD, kindly provided by Jonas Nyman, for setup and analysis. DMACRYS supercell calculations were carried out using the final crystal structures from DMACRYS (XXVII) or CrystalOptimizer (XXVIII, XXXI, XXXII and XXXIII), with the associated charge density files. These were used to calculate the zero point energy and thermal energy for the crystal structures using the Epanechnikov Kernel Density Estimation.

As noted for Submission 1, the 7th test introduced some systems, such as the organometallic XXVIII and the salt XXXIII, where the assumption of the Ψ_{mol} approach, that the charge distribution of a molecule within the crystal is very similar to that of the isolated molecule, is more of an approximation. However, we tackled all of the molecules in the spirit of seeing how far adaptations to our usual methodology could take us. Thus, we have separated the report into sections detailing the assessment of the CCDC-provided set of crystal structures, the structure refinement with the same method as was used in Submission 1, any changes required for Submission 2, our degree of confidence in our submission and a post-result analysis. We note that problems encountered with relabelling the atoms and that some of the provided conformations had very high conformational energies (possibly only reflecting

small differences in bond length) meant that the results differed from those that would have been obtained if there had been only one submission and our search had generated all the experimental structures.

2 XXVII – optoelectronic compound

2.1 Structure assessment

Some of the crystal structures provided for this molecule had strangely short bond lengths. We quickly realized that it would not be possible to just use the rigid molecule lattice energy minimization we used as the final step in Submission 1, as the conformations would be energetically unfeasible with our intramolecular energy model.

It was not possible to use CrystalOptimizer for this molecule, since we did not wish to adapt the code to be able to use iodine in the GAUSSIAN steps.

2.2 Structure refinement

2.2.1 Constrained geometry optimization

For each crystal structure, each molecule was extracted as a Z-matrix. All bonds were defined as variables, and all bond angles and torsion angles were fixed at the starting values. The molecules were then optimized in GAUSSIAN with the PBE0 method and 6-311G(d,p) basis set (basis functions for iodine were downloaded from https://www.basissetexchange.org/ and read in), with an iodine radius of 1.98 Å. The partially optimized molecule was then pasted into the crystal structure, minimizing the RMS displacement of the atoms.

2.2.2 DMACRYS

For each conformation, the charge density was calculated with the computational model described in Section 2.2.1.

A repulsion-dispersion potential was constructed for the molecule as follows:

- Parameters were taken from the FIT potential[4] for the aliphatic carbon and hydrogen atoms (including the sp-hybridized carbon atoms).
- Parameters were taken from the Universal Force Field[9] for the silicon atoms.
- Parameters were taken from Graeme Day's anisotropic potential[10] for the pentacene and attached hydrogen and iodine atoms.

Structures were then optimized with DMACRYS.

2.3 Degree of confidence

We have low confidence in the final energies of these crystal structures, since we were not able to change the conformations of the molecules in response to the packing forces.

2.4 Post-result analysis

The problems encountered by the CCDC in matching crystal structures of this molecule, coupled with the CCDC not providing the correct crystal structure for work in Submission 2, and the complications from the probable dynamic disorder in this system, mean that the analysis of this system is incomplete. However, we were pleased to note that the structure provided by the CCDC that was notionally a model for the experimentally observed form, CCDC28, was the lowest ranked in our Submission 2.

The full analysis using structures 28 & 61, 38 & 59 as models with incorrect isopropyl groups, gave that 28=61 was our global minimum in free and lattice energy, 59 was 8th in both energies and 38 was 61st in both energies. As group 10 (XtalPi) also had 38 as poorly ranked (59th) the higher ranking of this approximate model is probably not a problem with our energy model. Hence our energy model is looking surprisingly good for XXVII.

3 XXVIII – metalorganic compound

3.1 Structure assessment

Most of the conformations included the structures provided for Submission 2 were also included in our Submission 1 search space. Hence, we could deduce that the experimental structure did not contain Cu···Cu or Cu···Cl···Cu

bonding, which could not be treated by the Ψ_{mol} approach. The only omission was that some structures had a tetrahedral conformation with the two chlorine atoms cis to one another, but these could be treated in the same way as the Region F conformation (see Table 1), which was square-planar with the chlorine atoms cis to one another. Some structures had atoms with bond angles at exactly 180° which cannot be handled by GAUSSIAN. In these cases it was sufficient to move a chlorine atom's fractional coordinate in the x direction by 0.0001 in the starting crystal structure.

3.2 Structure refinement

3.2.1 CrystalOptimizer

The independent degrees of freedom optimized in CrystalOptimizer are given in Table 1. There needed to be some slight differences in the definition of the independent degrees of freedom between the tetrahedral and the square-planar conformations to prevent the latter relaxing to tetrahedral in the first step.

The intramolecular conformation and energy were evaluated at the PBE/6-31G(PCM ε=11) level of theory; the charge density was calculated at the same level of theory; the repulsion-dispersion parameters were taken from the FIT potential for C, H, N, O and Cl and from the UFF[9] potential for Cu. Optimizations were run to full convergence (This was the main difference from the method used in Submission 1).

3.2.2 Better quality refinement

CrystalOptimizer was run again, with the same degrees of freedom and same computational methods, apart from evaluating the charge density at the PBE0/6-31G(d,p)(PCM ε=11) level of theory. The optimized structures did not change much, but we used the structures and energies with this higher accuracy charge density for the Submission.

3.3 Degree of confidence

We have never worked with metallorganics before. DMACRYS refinement requires an *exp-6* repulsion-dispersion potential for each atomic type, and so we needed parameters for copper before embarking on this study. Since the copper atom is probably not involved in intermolecular interactions, the lattice energies may not be particularly sensitive to the choice of parameters for this atomic centre, and so we used the Universal Force Field[9] parameters for Cu^+ with a tetrahedral geometry. The resulting exp-6 potential performed adequately for DMACRYS lattice energy minimization of 12 out of 14 related crystal structures, suggesting that this choice might be adequate.

The multipoles, which are a key part of the DMACRYS refinement, are probably of much greater importance than the choice of repulsion-dispersion potential. Initial work with related compounds showed that the most critical factor was the use of a polarizable continuum for the charge density calculations. A pseudopotential for copper (LanL2DZ) performed better than not using one (although the improvement was not worth the problems of implementing this in CrystalOptimizer), and the improvement with the PCM was even greater. Comparing different functionals (PBE, PBE0, M06, B3LYP, CAM-B3LYP, wB97xD) showed little difference, and PBE was selected as being as good as PBE0 while being significantly less costly. Given the need to use the approximation of a PCM and *ab initio* methods that were rather inferior to those that would be used in the computer modelling of organometallic complexes, we have low confidence that the flexibility of the bonding to Cu was being adequately described.

3.4 Post-result analysis

The experimental crystal structure was ranked 6th in lattice energy in our Submission, with an $RMSD_{15}$ of 0.34 Å, only 4.76 kJ mol^{-1} above the most stable of the CCDC provided structures. Given the assumptions in our methodology, this is an encouraging result, suggesting that the Ψ_{mol} approach could be used to generate crystal structures of organometallics when the metal atom is buried so that the crystal packing is determined by the organic ligands. However, the final lattice energies should be evaluated with the best periodic electronic structure method that has been properly validated for the type of organometallic compound.

Table 1. The full list of independent degrees of freedom used in the CrystalOptimizer refinement (torsion angles are given in red and bond angles are given in blue). The numbering used for this molecule in our work does not take advantage of the intramolecular symmetry, since DMACRYS only works with whole molecules and so the atomic numbering has been extended from that in the main paper.

<table>
<tr><th>Gross conformation</th><th>CrystalOptimizer degrees of freedom</th></tr>
<tr><td colspan="2">Tetrahedral conformations</td></tr>
<tr><td>A
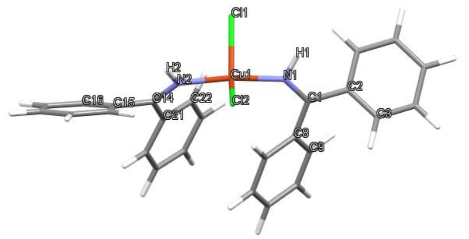
</td><td rowspan="2">Central geometry:
N1_Cu1_Cl1_Cl2, N2_Cu1_Cl1_Cl2, Cl2_Cu1_Cl1, N1_Cu1_Cl1, N2_Cu1_Cl1
Pyramidality at N atoms:
C1_N1_Cu1_H1, C14_N2_Cu1_H2, H1_N1_Cu1, H2_N2_Cu1, C1_N1_Cu1, C14_N2_Cu1
Major torsion angles:
H1_N1_Cu1_Cl2, C2_C1_N1_Cu1, C3_C2_C1_N1, C9_C8_C1_N1, H2_N2_Cu1_Cl1, C15_C14_N2_Cu1, C16_C15_C14_N2, C22_C21_C14_N2
Major bond angles
C2_C1_N1, C8_C1_N1, C15_C14_N2, C21_C14_N2</td></tr>
<tr><td>B
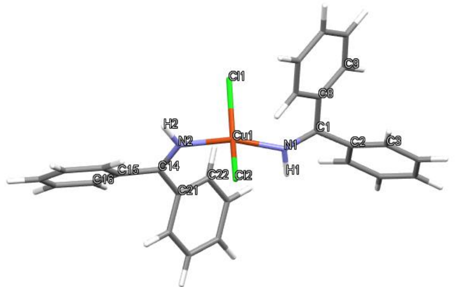
</td></tr>
<tr><td colspan="2">Square-planar-trans conformations</td></tr>
<tr><td>C
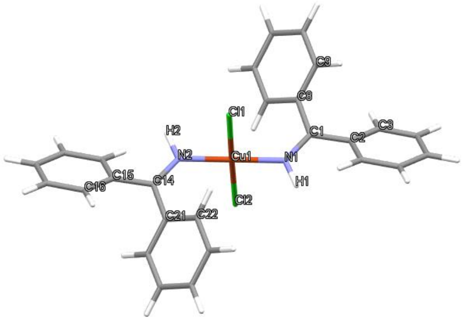
</td><td rowspan="2">Central geometry:
Cl2_Cu1_N1_Cl1, N2_Cu1_Cl1_N1, N1_Cu1_Cl1, Cl2_Cu1_N1, N2_Cu1_Cl1
Pyramidality at N atoms:
C1_N1_Cu1_H1, C14_N2_Cu1_H2, H1_N1_Cu1, H2_N2_Cu1, C1_N1_Cu1, C14_N2_Cu1
Major torsion angles:
H1_N1_Cu1_Cl2, C2_C1_N1_Cu1, C3_C2_C1_N1, C9_C8_C1_N1, H2_N2_Cu1_Cl1, C15_C14_N2_Cu1, C16_C15_C14_N2, C22_C21_C14_N2
Major bond angles:
C2_C1_N1, C8_C1_N1, C15_C14_N2, C21_C14_N2</td></tr>
<tr><td>D
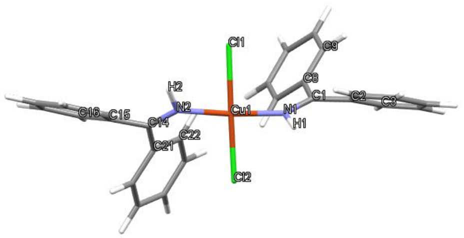
</td></tr>
<tr><td colspan="2">Square-planar-cis conformations</td></tr>
<tr><td>F
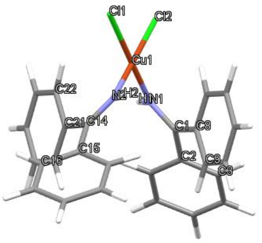
</td><td>Central geometry:
N1_Cu1_Cl2_Cl1, N2_Cu1_Cl1_Cl2, Cl2_Cu1_Cl1, N1_Cu1_Cl2, N2_Cu1_Cl1
Pyramidality at N atoms:
C1_N1_Cu1_H1, C14_N2_Cu1_H2, H1_N1_Cu1, H2_N2_Cu1, C1_N1_Cu1, C14_N2_Cu1
Major torsion angles:
H1_N1_Cu1_Cl2, C2_C1_N1_Cu1, C3_C2_C1_N1, C9_C8_C1_N1, H2_N2_Cu1_Cl1, C15_C14_N2_Cu1, C16_C15_C14_N2, C22_C21_C14_N2
Major bond angles
C2_C1_N1, C8_C1_N1, C15_C14_N2, C21_C14_N2</td></tr>
</table>

4 XXXI – agrochemical

4.1 Structure assessment

The CCDC list of 100 structures was compared with the 1500 submitted using Crystal Packing Similarity. The vast majority (62) were on our submitted list, relatively few (8%) where the best match was less than six molecules (i.e. many would have at least a layer in common with at least one structure within our Submission 1), and none overlaid fewer than three molecules, implying that we had fully covered the conformational space. Thus we continued with the same energy model used for Submission 1.

One structure stands out from its low density, CCDC89, having large voids that can accommodate a wide range of solvents that could stabilize the structure and be removed easily, as the voids are lined by aromatic and F groups.

4.2 Structure refinement

Lattice energy minimization of all structures was carried out in CrystalOptimizer. The degrees of freedom optimized were the three torsion angles of the backbone, the torsion angles defining the sulfoxide oxygen atoms, one torsion angle describing each methyl rotation, the two bond angles defining the sulfoxide oxygen atoms and the two bond angles defining the fluorine atoms on the backbone. The intramolecular conformation and energy were evaluated at the PBE0/6-31G(d,p) level of theory; the charge density was calculated at the same level of theory; repulsion-dispersion parameters were taken from the FIT potential for C, H, N, O, with parameters for the sulfoxide group taken from Scheraga's work.[11]

4.3 Degree of confidence

This small organic molecule is the sort of size we are used to dealing with, and so should have been within our capabilities. The *ab initio* method chosen for the conformational optimization and charge density was one that we have had success with in the past, and the repulsion-dispersion potential, combining the parameters for sulfoxide with the FIT potential, has also been successful, although we have noted difficulties with the F parameters in previous studies. The sensitivity of the relative ranking to the choice of repulsion-dispersion potential and the use of a polarization continuum model was such that we had only moderate confidence in the structures.

4.4 Post-result analysis

All crystal structures were retained in our lattice energy optimization, with A_{maj} and A_{min} being ranked 1st and 2nd in our free energy ranking (5th and 2nd in lattice energy), B ranked 43rd in free energy (47th in lattice energy) and solvent-stabilized form C being ranked 92nd in free energy (91st in lattice energy). However, B is found experimentally to be more stable than A at both 0 K and ambient conditions, which is not reflected in our energy ordering.

The Submission 2 results had too high a lattice energy for B to get the correct stability order. However optimizing B starting from the experimental structure of B gave the lowest energy structure (-117.61 kJ mol^{-1}) although the structure was too distorted ($RMSD_{15}$ = 1.280 Å with 40% tolerances) to count as a hit. The experimental crystal structure has significant thermal ellipsoids, and clearly the minimum found that approximated B was sensitive to starting structure.

We noted in our Submission 2 that "If we had been asked to rank structures for their likelihood of being observed, we would need to consider whether CCDC89 could have been stabilized by solvent in the voids during crystallization." Hence we spotted form C as a likely experimental structure.

5 XXXII – big pharmaceutical

5.1 Structure assessment

We renumbered the atoms within all crystal structures according to our internal numbering system. We did not take care to maintain the numbering of the piperidine rings or morpholine rings which may have caused problems similar to those with the morpholinium ion in XXXIII (see Section 6.1 below).

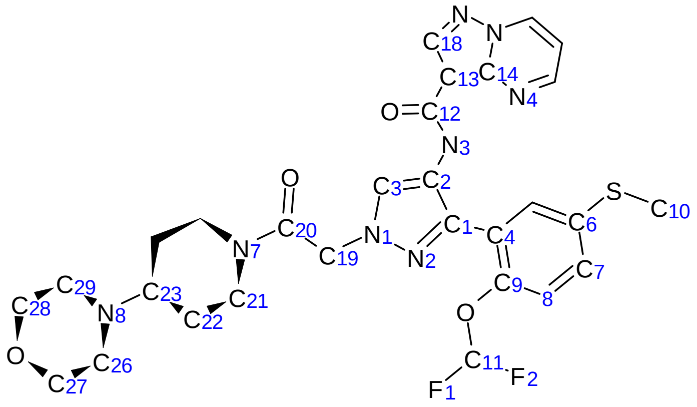

Figure 1. Numbering scheme for XXXII.

5.2 Structure refinement

5.2.1 DFTB3-D3

DFTB3-D3 was used to optimize the conformation of the molecule within the packing environment.[12] Crystal structure 208 could not be optimized with the DFTB3-D3 method, because the space group was not available.

5.2.2 DMACRYS

Each crystal structure had a unique conformation following DFTB3-D3 refinement, and the charge density for the exact conformation was calculated at the PBE0/6-31G(d,p) level of theory and the multipoles extracted. The structures were minimized with DMACRYS, keeping the molecular conformation rigid, and taking the repulsion-dispersion parameters from the FIT potential for C, H, N, O, F and from the Halgren potential[13] for sulfur. The intramolecular energy was taken from the charge density calculation.

At this stage, the intramolecular energy spanned a range of +55.7 to +181.2 kJ mol^{-1}. This was felt to be unfeasibly high. Therefore, we decided to refine these structures with a more computationally demanding and more accurate method.

5.2.3 CrystalOptimizer

CrystalOptimizer was used to refine the molecular conformations and packings simultaneously, starting from the DFTB-D3 optimized structures, to minimize the total lattice energy of each crystal structure. We tried running CrystalOptimizer for all crystal structures with the bare minimum number of independent degrees of freedom (Table 2). In a small proportion of cases, optimizing the conformation of the molecule constraining only these degrees of freedom led to large enough conformational changes that molecular clashes resulted when the molecule was put back into the crystal structure (which was done by minimizing the RMS atomic displacements). In these cases, further degrees of freedom were added to restrict the conformational change, and CrystalOptimizer started again. The energies calculated allowing different degrees of freedom to respond to the packing forces are not strictly comparable between crystal structures.

Table 2. Degrees of freedom optimized in CrystalOptimizer.

Minimum degrees of freedom			Additional degrees of freedom	
C21_N7_C20_C19	N1_C19_C20_N7	C3_N1_C19_C20	C27_C26_N8_C23	C28_C29_N8_C23
C9_C4_C1_C2	C11_O_C9_C4	F1_C11_O_C9	C22_C21_N7_C20	C2_C3_N1_C19
C10_S_C6_C7	H_C10_S_C6	C26_N8_C23_C22	N4_C14_C13_C12	C4_C1_C2_C3
C12_N3_C2_C3	C13_C12_N3_C2	C14_C13_C12_N3	C8_C9_C4_C1	C7_C8_C9_C4
			S_C6_C7_C8	

The PBE0/6-31G(d,p) level of theory was used to evaluate the intramolecular energy and the charge density, and GDMA2 was used to extract the multipoles. The repulsion-dispersion potential was as described in Section 5.2.2.

5.3 Degree of confidence

Our decision to use DFTB3-D3 to optimize the crystal structures, allowing some conformational adjustment, has been shown to be very effective for large flexible molecules.[12] The lack of reliability of the DFTB3-D3 energies meant that a single GAUSSIAN job was run for each structure to obtain the charge density for the multipoles and the intramolecular energy, with the intermolecular energy evaluated with DMACRYS. In this case, the energy range was far greater than we felt was feasible, so we had to carry out the more computationally demanding CrystalOptimizer calculations.

Following CrystalOptimizer, we still had three structures with intramolecular energies in excess of 100 kJ mol^{-1}, but this was a great improvement over the energies derived from the DFTB3-D3 structures.

Comparing the 500 crystal structures provided by the CCDC and those after DFTB3-D3 and after further CrystalOptimizer refinement (by comparing $RMSD_{15}$ only) showed generally more structures changed significantly during the CrystalOptimizer step. Assuming that the CCDC structures had been generated by a method that allowed all-atom refinement, this suggests that DFTB3-D3 is not changing the structure unfeasibly, but that CrystalOptimizer optimization of a small subset of degrees of freedom is not appropriate for this type of flexible molecule (i.e. some of the central degrees of freedom that are being modelled by isolated molecule constrained optimization may only be in error by a small amount, but this translates into a large effect on the relative positions of the functional groups at the extremes of the molecule). Thus we had low confidence that our Submission 2 had adequately represented the flexibility of this molecule.

5.4 Post-result analysis

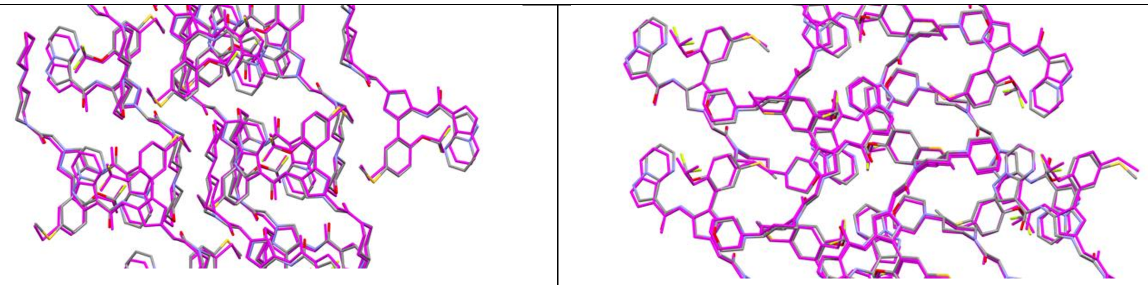

Form A_{maj} ($RMSD_{15}$=0.397 Å)<br>E_{latt}=-212.41 kJ mol^{-1} (#209)<br>FE=-234.49 kJ mol^{-1} (#195)<br>($RMSD_{15}$ exp/DFTB3-D3 = 0.386 Å)	Form B_{LT} ($RMSD_{15}$=0.347 Å)<br>E_{latt}=-223.87 kJ mol_{-1} (#41)<br>FE=-244.99 kJ mol^{-1} (#42)<br>($RMSD_{15}$ exp/DFTB3-D3 = 0.420 Å)

Figure 2. The reproduction of two of the experimental structures of XXXII as given by the $RMSD_{15}$ overlays (atomic colour experimental, magenta CrystalOptimizer structure submitted). The $RMSD_{15}$ of the overlays of the DFTB3-D3 optimized structures with the experimental structures are also given, which shows that for these structures, DFTB3-D3 all atom does not give a better overlay.

Structures of both A_{maj} and B were retained during our lattice energy optimization, with a reasonable reproduction for this size of molecule (Figure 2) with A_{maj} being ranked very poorly and B_{LT} still poorly relative to the most stable structure (with E_{latt}=–235.26 kJ mol^{-1} and FE=–256.01 kJ mol^{-1}). (With the original criteria used by CCDC in their analysis, these counted as tentative matches.) A version of A_{min} was not included in the structures provided by the CCDC. The structure of the room temperature determination of form B_{HT} was altered too much to be recognizable during our lattice energy optimization. However, after the crystal structures were released we ran CrystalOptimizer on the exact experimental structure, B_{HT}, which resulted in a substantially changed crystal structure indicating that this is not a true minimum with our computational model. Since the $RMSD_{15}$ between the two forms of B is only 0.573 Å and B_{HT} has an additional screw and glide plane to the Z'=2 B_{LT}, it seems likely that B_{HT} is a dynamical average which is a transition state on the potential energy surface.

From the experimental structures (Figure 2) it appears that the Submission 1 approach of refining the energy ranking after DFTB3-D3 with one DMACRYS optimization would not have given a better overlay for the experimental structures. It seems likely that using CrystalOptimizer on a limited number of degrees of freedom in a molecule of

this size and flexibility is probably an adequate intermediate step, but further refinement with a good all-atom relaxation, e.g. ψ_{crys} with PBE-TS, would probably give better structural overlays and energy rankings.

6 XXXIII – organic salt

6.1 Structure assessment

We renumbered all the provided crystal structures according to our in-house numbering scheme. Unfortunately, we didn't take care to maintain the configuration of the morpholinium cation. Since our databases of gas phase optimizations from Submission 1 were being reused, and the atom matching is done by the labels with the optimized molecule being pasted in to minimize atomic displacements, it can be seen from Figure 3 that not taking the care to do this correctly can lead to the molecule being altered inappropriately. The hydrogen atoms involved in hydrogen bonding would point in completely the wrong directions, and so the crystal structure would change unrecognizably on optimization. This resulted in form B being lost at the very first stage.

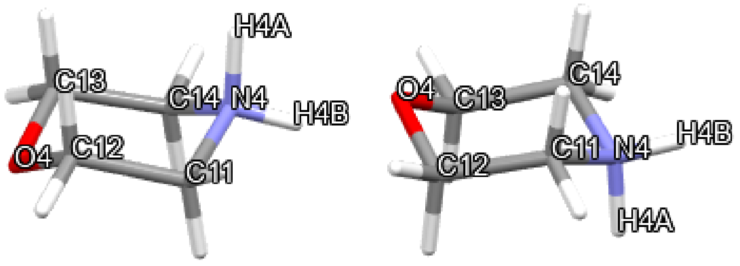

Figure 3. Comparison of (left) correctly numbered morpholinium cation and (right) incorrectly numbered morpholinium cation.

6.2 Structure refinement

Lattice energy minimization of all structures was carried out in CrystalOptimizer. The morpholinium cation was kept rigid and the degrees of freedom optimized for the sulfamethoxazole anion were the three angles of the molecular backbone, the torsion angles defining the sulfoxide oxygen atoms, the torsion angles defining the amine hydrogen atoms, one torsion angle describing the methyl rotation, the two bond angles within the linking group, the two bond angles defining the sulfoxide oxygen atoms and the two bond angles defining the amine hydrogen atoms. With the exception of the torsion angle to define the methyl rotation, gradients of the multipoles were also calculated for all degrees of freedom; this increases computational cost at each step but stops the optimization taking the wrong trajectory. Past experience led us to include torsion angles within the π-conjugated rings at points adjacent to large functional groups. Four such degrees of freedom were included, although amine and methyl groups are not usually considered large.

All molecular geometry optimizations and charge density calculations were carried out with the PBE0/6-31+G(d,p) (PCM ε=11) method. Repulsion-dispersion parameters were taken from the FIT potential, with parameters for the sulfoxide group taken from Scheraga's work.[11]

6.3 Degree of confidence

The *ab initio* calculations on ions *in vacuo* do not give a good representation of the charge distribution in the crystals, and using a basis set with diffuse functions and a polarizable continuum with a high dielectric only represents the polarization of the ions in an average fashion, not the polarization in the specific crystal structure. Hence, the Ψ_{mol} ranking of the crystal structures is unlikely to be reliable.

6.4 Post-result analysis

Form A was ranked 6th in free energy (4th in lattice energy) in our Submission, but there was no structural match for Form B.

It was only when the results were released that we realised that we had accidentally misnumbered the morpholinium cation in Form B (see Section 6.1). Evaluating the energy with the same model after the structure was

released showed that it would have been lower in lattice energy than any other structure on our energy landscape (i.e. Rank #1).

7 References

(1) *Gaussian 09, Revision D.01*; 2009.

(2) Price, S. L.; Leslie, M.; Welch, G. W. A.; Habgood, M.; Price, L. S.; Karamertzanis, P. G.; Day, G. M. Modelling Organic Crystal Structures using Distributed Multipole and Polarizability-Based Model Intermolecular Potentials. *Physical Chemistry Chemical Physics* **2010**, *12* (30), 8478-8490.

(3) Stone, A. J. GDMA: A Program for Performing Distributed Multipole Analysis of Wave Functions Calculated Using the Gaussian Program System. *GDMA2.2* **2010**.

(4) Williams, D. E.; Cox, S. R. Nonbonded Potentials For Azahydrocarbons: the Importance of the Coulombic Interaction. *Acta Crystallographica Section B - Structural Science* **1984**, *40* (8), 404-417.

(5) Coombes, D. S.; Price, S. L.; Willock, D. J.; Leslie, M. Role of Electrostatic Interactions in Determining the Crystal Structures of Polar Organic Molecules. A Distributed Multipole Study. *Journal of Physical Chemistry* **1996**, *100* (18), 7352-7360.

(6) Hsu, L. Y.; Williams, D. E. Intermolecular Potential-Function Models for Crystalline Perchlorohydrocarbons. *Acta Crystallographica Section A - Crystal Physics, Diffraction, Theoretical and General Crystallography* **1980**, *36* (MAR), 277-281.

(7) Williams, D. E.; Houpt, D. J. Fluorine Nonbonded Potential Parameters Derived From Crystalline Perfluorocarbons. *Acta Crystallographica Section B - Structural Science* **1986**, *42* (JUN), 286-295.

(8) Kazantsev, A. V.; Karamertzanis, P. G.; Adjiman, C. S.; Pantelides, C. C. CrystalOptimizer. An efficient Algorithm for Lattice Energy Minimisation of Organic Crystal using Isolated-Molecule Quantum Mechanical Calculations. In *Molecular System Engineering*, Adjiman, C. S., Galindo, A. Eds.; Process Systems Engineering, Vol. 6; WILEY-VCH Verlag GmbH & Co., 2010; pp 1-42.

(9) Rappe, A. K.; Casewit, C. J.; Colwell, K. S.; Goddard, W. A.; Skiff, W. M. UFF, a Full Periodic-Table Force-Field For Molecular Mechanics and Molecular-Dynamics Simulations. *Journal of the American Chemical Society* **1992**, *114* (25), 10024-10035.

(10) Day, G. M.; Motherwell, W. D. S.; Ammon, H. L.; Boerrigter, S. X. M.; Della Valle, R. G.; Venuti, E.; Dzyabchenko, A.; Dunitz, J. D.; Schweizer, B.; van Eijck, B. P.; et al. A third blind test of crystal structure prediction. *Acta Crystallographica Section B - Structural Science* **2005**, *61* (5), 511-527.

(11) Motherwell, W. D. S.; Ammon, H. L.; Dunitz, J. D.; Dzyabchenko, A.; Erk, P.; Gavezzotti, A.; Hofmann, D. W. M.; Leusen, F. J. J.; Lommerse, J. P. M.; Mooij, W. T. M.; et al. Crystal structure prediction of small organic molecules: a second blind test. *Acta Crystallographica Section B - Structural Science* **2002**, *58*, 647-661.

(12) Iuzzolino, L.; McCabe, P.; Price, S. L.; Brandenburg, J. G. Crystal structure prediction of flexible pharmaceutical-like molecules: density functional tight-binding as an intermediate optimisation method and for free energy estimation. *Faraday Discussions* **2018**, *211*, 275-296, 10.1039/C8FD00010G. DOI: 10.1039/C8FD00010G.

(13) Halgren, T. A. Representation of Vanderwaals (Vdw) Interactions in Molecular Mechanics Force-Fields - Potential Form, Combination Rules, and Vdw Parameters. *Journal of the American Chemical Society* **1992**, *114* (20), 7827-7843.

21. Groups 26 and 27

Supplementary Information
Groups 26 & 27: Stage 2

Rajni M. Bhardwaj, Eric J. Chan, Richard Hong, Ommair Ishaque, Aling Jing, John W. Melkumov, Rahul Nikhar, Rafał Podeszwa, Atta Rehman, Jutta Rogal, Hongxing Song, Krzysztof Szalewicz, Mark E. Tuckerman, and Leslie Vogt-Maranto

(Dated: May 23, 2024)

CONTENTS

I. Introduction 4

II. Target XXVII 7
A. Monomer reoptimizations with constraints 7
B. Improvements of intermonomer aiFFs 8
C. Crystal structure rankings 9
D. Results 10
E. Post-submission analysis 10

III. Target XXVIII 10
A. Monomer reoptimizations with constraints 10
B. Improvements of intermonomer aiFFs 11
C. Crystal structure rankings 11
D. Results 12
E. Post-submission analysis 12

IV. Target XXXI 12
A. Monomer reoptimizations with constraints 12
B. Improvements of intermonomer aiFFs 13
C. Reparametrization of empirical intramonomer FFs 14
D. Lattice-energy minimizations and molecular dynamics simulations 14
E. Results 15
F. Post-submission analysis 15

V. Target XXXII 17
A. Monomer reoptimizations with constraints 17
B. Improvements of intermonomer aiFFs 17
C. Crystal structure rankings 18
D. Results 18
E. Post-submission analysis 19

VI. Target XXXIII 19
A. Monomer reoptimizations with constraints 19
B. Improvements of intermonomer aiFFs 20
C. Reparametrization of empirical intramonomer FFs 21
D. Lattice energy minimizations with UPACK 22
E. Supercell energy minimizations and molecular dynamics simulations 22

F. pDFT+D calculations 23
G. Final energy rankings 23
H. Results 24
I. Post-submission analysis 24

VII. Summary 25

References 29

I. INTRODUCTION

The work of our team on stage 1 of 7BT was described in our SI (SI1) to the stage 1 manuscript: “The Seventh Blind Test of Crystal Structure Prediction: Structure Generation Methods”. In the present SI (SI2), we will not repeat the material covered in SI1, including the definitions of acronyms. The list of team members working on individual targets in stage 2 is given in Table I.

TABLE I: Summary of team members (represented by their initials) who worked on each target. A check-mark signifies that a person contributed in the submission for that target. ECJ and HS did not work on stage 2.

System	RMB	RH	OI	AJ	JWM	RN	RP	AR	JR	KS	MET	LVM
XXVII				✓		✓	✓			✓		
XXVIII						✓	✓	✓		✓		
XXXI			✓			✓	✓		✓	✓		✓
XXXII					✓	✓	✓	✓		✓		
XXXIII	✓	✓				✓	✓			✓	✓	✓

The goal of stage 2 of the 7BT, focused on structure ranking methods, was to rank polymorphs on the lists of 100 (targets XXVII and XXXI) or 500 (targets XXVIII, XXXII, and XXXIII) polymorphs per target prepared by CCDC. There was no information provided to participants on how these lists were prepared. Our team assumed that they were prepared in a similar way as informal lists provided by groups specializing in structure generation to groups specializing in ranking during 6BT, i.e., these were list from generations performed using empirical FFs. We also assumed that there will be no polymorph close enough to the experimental polymorph to satisfy the 7BT criteria of similarity ($\mathrm{RMSD}_{30} < 1$ Å within 25%/25° tolerances), as in most cases generations with empirical FFs do not provide predictions with such accuracy. We also assumed that the list will contain an “experimental representative”, i.e., a polymorph similar to the experimental one, but deformed to have RMSD_{30} larger than the threshold of acceptance assumed in the test, so that it should be impossible to rank this crystal well without a significant geometry optimization, including both the monomer geometry and the crystal geometry. This understanding was reinforced by the analysis of monomers' energy ranges for the targets, shown in Table II. These ranges for some systems are as large as 1000 kJ/mol. Since intermonomer contributions to lattice energies are in hundreds of kJ/mol, monomer-deformation penalties of the order of 1000 kJ/mol would make such crystals unbound. Moreover, the range of monomer energies coming from CSPs with empirical FFs are typically within dozens of kJ/mol. For example, Fig. 1 shows monomer energies from flexible-monomer CSPs of target XXX submitted by our team in

TABLE II: Ranges of energies (in kJ/mol) for the monomers from the CCDC lists relative to the given monomer equilibrium energy. The latter energies are also listed (in hartree).

	XXVII	XXVIII	XXXI	XXXII	XXXIII
lowest	10.4	4.9	13.1	6.3	1.1
highest	1310.5	991.0	554.9	1232.0	137.0
method	PBE0+D3BJ	PBE+D3BJ	PBE+D3BJ	PBE+D3BJ	PBE0+D3BJ
basis set	def2-TZVPP	aug-cc-pVTZ	aug-cc-pVTZ	def2-TZVP	aug-cc-pVTZ
E_{equil}	-2880.886276*	-3673.074491	-1441.671572	-2459.456556	-1174.512790

*The reason why monomer's XXVII total electronic energy is not the largest in magnitude despite this monomer being the largest in size and containing two iodine atoms is the use of ECPs for these atoms.

stage 1. We have extracted the monomers from the 100 top-ranked polymorphs and plotted in Fig. 1 their *ab initio* energies from PBE0+D3/aug-cc-pVTZ calculations. While the ranking was done using monomer-deformation penalties from such *ab initio* calculations and intermolecular energies from SAPT aiFFs, the structure generation was performed using OPLS-AA FF in fully flexible manner. Figure 1 shows that the range of monomer energies is less than 40 kJ/mol, in stark contrast with the ranges in Table II. Thus, the presence of monomers with such a spread of energies in the structures provided by CCDC could be only due to purposeful deformations introduced by the CCDC team. Based on these data, we assumed that stage 2 CSPs have to involve significant optimization of monomer geometries. This was a bad news for our team since our protocol uses very accurate intermonomer FFs, but relies on empirical FFs (possibly enhanced by refitting some parameters to *ab initio* data), which are much less accurate. Based on this analysis, we put very significant effort in either reoptimization of monomers or refitting empirical intramonomers FFs. This was necessary since had we used the original CCDC monomers, most of the crystal structures would have to be rejected due to monomer-deformation penalties comparable to or larger than the magnitude of the lattice energy resulting from intermonomer forces. As it will be described for individual targets and summarized in Sec. VII, we would have obtained much better predictions with significantly reduced effort using the monomers in exactly the same geometries as in the CCDC lists. This is due to the fact that, as we found after the experimental structures were revealed, the experimental representatives included in the CCDC lists were actually very close to the experimental crystals.

To account for the variability of the monomer energies in the CCDC lists, we constraint-reoptimized all monomer geometries. This was done for all targets, even targets XXXI and XXXIII for which we applied flexible-monomer CSPs since these geometries were needed for the CCfC im-

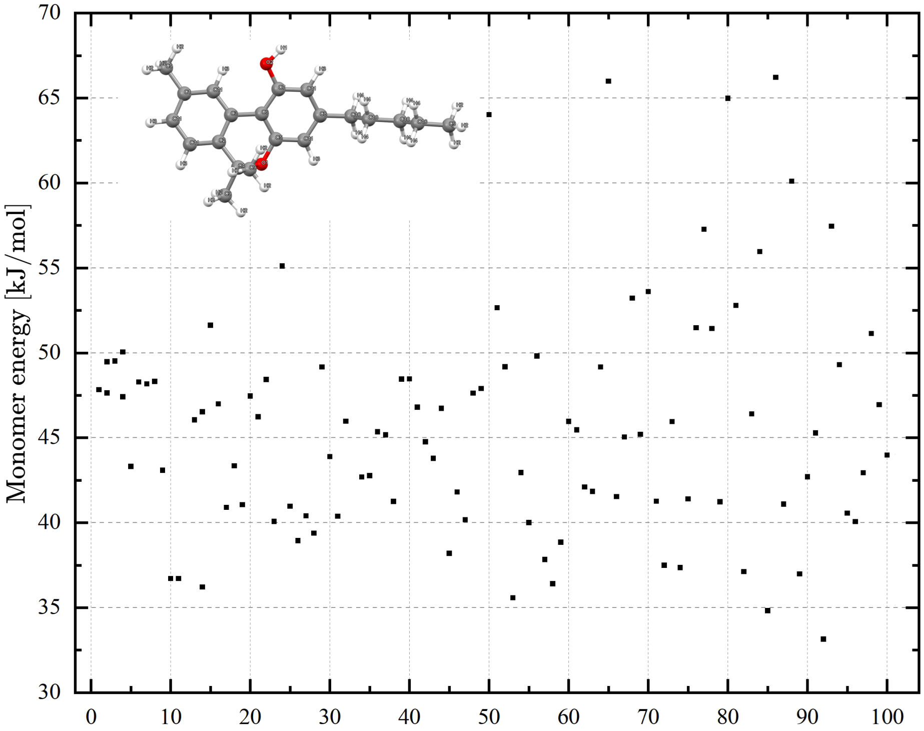

FIG. 1: Energies of monomer C of target XXX. The horizontal axis enumerates the monomers in the 100 top-ranked crystal structures submitted by our team in stage 1. Monomer geometries were generated using OPLS-AA, but the energies that are plotted were computed using PBE0+D3/aug-cc-pVTZ. All energies are given with respect to the energy of the monomer at the *ab initio* equilibrium geometry.

provements of aiFFs. The degrees of freedom constrained in the optimizations were soft dihedral angles. The rationale for this was that the CCDC team likely did not distort such angles since these distortions would have changed the shapes of monomers and lead to unreasonable crystal structures. For largest systems, we have constrained also all (parts) of hard (improper) dihedral angles for computational efficiency reasons. Test on selected monomers have shown that the energies obtained in these two ways (i.e., with only the soft versus soft plus hard dihedral angles frozen) differed in most cases by only up to 1 kJ/mol.

For all targets, the intermonomer aiFFs developed in stage 1 were improved by using the CCfC method to generate a set of new grid points (dimer configurations) that were added to the previous training sets and the fits were redone.

Since the goal was to rank the crystal structures provided by CCDC, no structure generation steps of CSPs were performed at stage 2. The structures provided were first modified by replacing the original monomers with constraint-optimized ones, and then either their lattice energies were

minimized or MD simulations were performed. In both cases, the starting crystal structures were the modified ones, except for target XXXI. In particular, for targets XXVII, XXVIII, and XXXII, we performed rigid-monomer lattice-energy minimization with the constraint-optimized monomers. To get the starting configurations for these minimizations, we embedded the constraint-optimized monomers into crystal lattices from the CCDC lists by placing the center of mass (COM) of the constraint-optimized monomer at the position of the COM of the original monomer and aligning the principal axes of inertia of both monomers. One exception was target XXVII, see Sec. II C. For targets XXXI and XXXIII, we used aiFFs for intermonomer interactions and enhanced empirical FFs for intramonomer interactions in flexible-monomer lattice-energy (0 K) minimizations and in MD simulations of crystals at finite temperatures (starting with the original monomers for XXXI and the constraint-optimized monomers for XXXIII). The enhancement consisted either in replacing the hard geometric parameters (equilibrium bond lengths and bond angles) by the values from the *ab initio* equilibrium monomer or performing such a replacement and, in addition, optimizing the force constants to *ab initio* energies for a set of monomer geometries. Note that in stage 1 we used only rigid-monomer CSPs for XXXI, so this is an extension of our previous approach. On the other hand, for XXXII we used flexible-monomer CSPs in stage 1, but rigid-monomer ones in stage 2. The reason here was that monomer XXXII is too large to perform enhancements (within the time limits of 7BT) and, on the other hand, one cannot rely on generic empirical FFs for structure ratings. For some systems, we have also performed periodic dispersion-corrected DFT calculations (pDFT+D), always only single-point calculations (fixed crystal geometry, no optimizations). These calculations were used only to compare with the results obtained as described above, although in one case we moved some polymorphs ranked high by pDFT+D up on our submission list (see Sec. II).

II. TARGET XXVII

A. Monomer reoptimizations with constraints

A considerable number of monomers from the 100 polymorphs provided by CCDC had energies significantly higher than that of our *ab initio* optimized gas-phase equilibrium monomer. Only 20% of the monomers had energies within 5 kcal/mol, 58% within 10 kcal/mol, 74% within 15 kcal/mol, and 6% exceeded 60 kcal/mol above the energy of the global minimum monomer. As discussed in Sec. I, given the presence of monomers with such high energies, we inferred that CCDC may have purposely introduced monomer deformations in the supplied structures. Therefore, we decided to optimize the CCDC-provided monomers with constraints consisting in freezing all dihedral angles, while allowing variations in bond lengths and bond angles. Thus, for this monomer, not only the soft dihedral angles were frozen, but also the hard (improper) angles. However, the latter are

locked by their chemical environment and a sampling of the CCDC monomers indicated that they are at the right values, so that this extension of the range of frozen parameters should not be consequential (and results in savings of computer time). Additionally, for monomers with energies $\leq$20 kcal/mol above the global minimum, constraints were imposed on the bond angles within the near-linear C-C≡C-Si group of atoms (two bond angles in each group) to avoid the shifts of the triisopropylsilyl (TIPS) groups. The minimization used PBE0+D3(BJ) [1, 2] with def2-TZVPP [3] basis set in the Orca-4.1.1 package [4]. After the minimization, 82% of the monomers' energies were less than 5 kcal/mol above the global minimum, with the maximum deviation being 17 kcal/mol. The minimization procedure in particular rectified unphysically short -C-Si- bonds in several extensively deformed monomers. Despite the significant changes of monomers' energies, the optimization process resulted in only modest adjustments to the molecular geometries. When we compared the root mean square deviations (RMSDs) for all the non-hydrogen atoms between the original CCDC monomers and those post-optimization, the values were consistently less than 0.3 Å, with a majority being even lower, under 0.1 Å.

B. Improvements of intermonomer aiFFs

We improved the stage 1 aiFF by adding grid points to the training set and refitting the aiFF. (*a*) We first incorporated 2279 dimer geometries extracted from the rigid-monomer-optimized crystals submitted by us in stage 1 in order to represent better previously undersampled areas. These dimers were extracted using the CCfC method. (*b*) Next, the accuracy of the stage 1 aiFF was examined on the original CCDC-provided polymorphs. We cut from each polymorph a 2x2x2-unit-cell slab and computed all dimer interaction energies from aiFF. For the dimers with most negative interaction energies, we computed the *ab initio* interaction energies. It turned out that for most dimers the agreement of the fit with the *ab initio* energies was very good, but for three polymorphs, #12, #64, and #81, aiFF had the relative errors exceeding 28%. (*c*) We have taken the monomers from these three polymorphs (the original ones, before the constrained optimizations) and used UPACK to perform CSPs in the same space symmetry groups as those of the three polymorphs. The CSPs were performed in the same way as we did in stage 1. We selected the total of 445 polymorphs from these CSPs ordered by lattice energies (including monomer deformation penalties). (*d*) We again generated 2x2x2 slabs, computed fit energies of all dimers in each slab, picked up the dimer with the most negative interaction energy, and computed *ab initio* interaction energies for these dimers, so that 445 dimers were added to the training set from this procedure. (*e*) Finally, we considered CCDC-provided crystals with the original monomers replaced by the constraint-optimized monomers. Using the same slab-based procedure as described above, we extracted 4 dimers with the most negative fit interaction energies from each polymorph, computed their *ab initio* interaction energies, and added these 400 grid points

to the training set. The fit error distribution for these dimers was essentially the same as in case (b). The complete new set of grid points consisted of 15,390 dimers, and its RMSE for the dimers with negative interaction energies was 0.66 kcal/mol, lower than 0.69 kcal/mol for the stage 1 aiFF on its training set, despite including now a much more extensive range of monomer geometries. The accuracy of the aiFF in predicting the energies of the dimers extracted from the CCDC crystals with constraint-optimized monomers also improved significantly, with only two dimers having fitting errors larger than 5%. Note that the target XXVII procedures used to improve the aiFF (except for step (a)) were different from the CCfC protocol used for all other targets.

C. Crystal structure rankings

We ranked the structures by performing rigid-monomer optimizations with constraint-minimized monomers utilizing the UPACK [5, 6] package and the aiFF described in the previous subsection. Each optimizations was started from one of the CCDC polymorphs with the original monomers replaced by the constraint-minimized ones. To preform this replacement, we aligned the new monomers with their initial counterparts in crystals by minimizing the RMSD of all corresponding non-hydrogen and non-isopropyl groups atoms (maintaining the cell parameters unchanged). This procedure is different from that applied to other targets. After the UPACK minimizations were completed, we added to the lattice energies obtained from such rigid-monomer optimizations the monomer-deformation penalties. These penalties account for the energy differences between the crystal monomers and the equilibrium (global energy minimum) monomer, all computed in gas phase with PBE0+D3(BJ)/def2-TZVPP using the ORCA package.

Concurrently, we also performed single-point (no geometry optimizations) dispersion-corrected periodic DFT (pDFT+D) calculations for the CCDC-provided polymorphs with constraint-optimized monomers. These calculations were carried out using the SCAN [7] DFT functional with the D3(BJ) dispersion correction using the VASP package [8]. We used a 700 eV cutoff for the plane-wave basis sets and a k-point grid spacing corresponding to a density of 0.15 Å^{-1}. We have also performed geometry optimization for a few polymorphs, but the geometry and energy changes were very small, so we did not continue on this path.

The UPACK/aiFF and the pDFT+D ranking agreed reasonably well for the top 4 structures. The fifth-ranked structure in pDFT+D calculation was ranked 25th in UPACK. In our final submission, we ordered the structures according to the UPACK energies, except that the structure ranked 25th was moved to the 6th place.

D. Results

The CCDC-provided polymorph #28 turned out to be the closest experimental representative. After UPACK's optimizations, it was ranked by us as number 3 with all non-hydrogen atoms $RMSD_{20} = 0.51$ Å and core-only non-hydrogen $RMSD_{30}$ of 0.437 Å. There were three other polymorphs in the CCDC set that could be considered to be experimental representatives. Structure #59 had rank 4 and $RMSD_{20} = 0.84$ Å. Structures #38 and #61 were ranked at 70 and 11 place, respectively, but had numbers of matching molecules smaller than 20 ($RMSD_{10} = 0.79$ Å and $RMSD_{16} = 0.54$ Å, respectively). We used 25%/25° tolerances in these comparisons. In the core-only comparisons with experiment, all submitted structures of experimental representatives had 30/30 molecules matching within the 30%/30° tolerances. $RMSD_{30}$ for those polymorphs are 0.44 (#28), 0.55 (#38), 0.84 (#59), and 0.47 (#61). Our predictions for XXVII were among the best within the set of participating groups.

E. Post-submission analysis

Our good predictions for target XXVII are partly due to the composition of the CCDC-provided list: structures The monomers in structures #28, #59, and #61 were very close to *ab initio* conformers and our constrained optimizations essentially did not change these monomers. Also the subsequent lattice-energy minimizations kept the core structures in place. The ranking of the original CCDC polymorphs #28, #59, and #61 was 1, 5, and 3, respectively. The ranking of these polymorphs with the monomers replaced by constraint-optimized ones was even slightly better: 1, 5, and 2, respectively. Thus, our rigid-monomer optimizations made rankings worse.

III. TARGET XXVIII

A. Monomer reoptimizations with constraints

The CCDC monomers of system XXVIII were optimized under constraints using the PBE+ D3(BJ) functional [9–11] with the aug-cc-pVTZ [12] basis set. It would have been better to use the more accurate hybrid PBE0 functional [1], but hybrid-functional optimizations are significantly more time consuming. In the optimization process, 52 out of 132 dihedral angles were constrained. There are only 8 soft dihedral angles in the monomer of target XXVIII, but to reduce computer resources, we also constrained some of the improper dihedral angles that have their values nearly fixed by chemical environments. The constrained hard dihedral angles were those formed exclusively by the carbon atoms, while those containing also the hydrogen atoms were optimized. Before the constrained optimizations, 57% of monomers had energies relative to the energy of the gas-

phase equilibrium monomer larger than 50 kJ/mol. The optimization reduced this number to just 11%. Some monomers initially had relative energies of nearly 1000 kJ/mol. After optimizations, all these energies were reduced to below 60 kJ/mol. Despite these large energetic changes, the average RMSD (non-hydrogen atoms only) between the original and constraint-optimized monomer geometries was only 0.08 Å, while the largest change was 0.27 Å.

B. Improvements of intermonomer aiFFs

The CCDC-supplied a list of 500 structures included both planar and folded monomers. In stage 1, we developed an aiFF using the global minimum monomer, which is folded. While this aiFF can be used in its flexibilized form, it may perform less well than it is possible for planar monomers. This is due to the fact that some atoms in folded monomers are "hidden" from the point of view of intermolecular interactions. By incorporating dimers made up of different monomers, the parameters of the fit take on averaged values between the values for particular monomer geometries. It is expected that such a fit performs better upon flexibilization. To improve our aiFF in this respect, 5696 dimers were derived from the CCDC structures (with the original monomers replaced by the constraint-optimized ones) using the CCfC code [6]. Their interaction energies were calculated with PBE0+D3(BJ) [1, 10] using the cc-pVTZ basis set, consistent with stage 1. These new points, which included a broad range of monomer geometries, were merged with the phase 1 training data points (8195 grid points), resulting in the training set containing $N_{\rm grid}$ = 13,891 grid points. In this improved fit, the RMSE for the 9884 dimers with $E_{\rm int} < 0$ was 0.48 kcal/mol, with the ratio of this error to the global minimum depth amounting to 2.2%. With the total number of free parameters $N_{\rm FP} = 504$, the ratio $N_{\rm grid}/N_{\rm FP}$ is 27.6.

C. Crystal structure rankings

The constraint-optimized 500 monomers were inserted into the crystal structures by matching the COM and principal axes of inertia of the original monomers. Such polymorphs were then used as starting points in rigid-monomer lattice-energy minimizations with the stage 2 aiFF using a customized version of UPACK [5, 6]. The final lattice energies were composed of intermolecular lattice energies from UPACK and intramolecular energy corrections (the deformation penalties). The latter contributions was calculated as the difference between the gas-phase energies of the monomer in the crystal and the equilibrium monomer. For these calculations, we used PBE0+D3(BJ)/cc-pVTZ in the ORCA-4.2.1 software package [4, 13]. The final ranking of the CCDC structures was based on the UPACK rigid-monomer minimized lattice energies, where not only the cell parameters are relaxed but also the orientations of monomers and their positions in the cell were also changed.

We also independently carried out single-point (no optimizations) pDFT+D calculations on the structures from the rigid-monomer lattice-energy minimizations with constraint-optimized monomers. We used the PBE+D3(BJ) functional [9, 10] implemented in the Quantum ESPRESSO (QE) package [14, 15]. The plane-wave energy cutoff and charge density cutoff used were 685.86 eV and 4435.46 eV, respectively. These calculations assisted in verifying the accuracy and convergence of the stage 2 aiFF, but were not used in the submission. The rankings from the pDFT+D calculations were consistent within the two top-ranked structures with the aiFF-based calculations.

D. Results

The experimental representative structure #144 was ranked as 1 for this target, and the $RMSD_{30}$ between the actual experimental structure and the rank 1 submitted structure was 0.20 Å. As described in main papers, the crystal structure of XXVIII was inadvertently published by a group unrelated to 7BT. We have not used the published structure in any way in our CSPs.

E. Post-submission analysis

One reason for our successful predictions in stage 1 is good quality of our aiFF. We used a triple-zeta basis set (although without diffuse functions) which is large for a monomer of this size and meticulously eliminated the 'holes' in the PES during the fitting process. Finally, the new aiFF was fitted to a diverse set of monomer geometries. The other reason is that the polymorph #144 in the CCDC set included the monomer very close to the experimental one. The single-point calculation on the exact structure #144 gives also rank 1 and $RMSD_{30} = 0.234$ Å. This monomer was changed by the constrained optimization very little, with the RMSD between the two monomers (non-hydrogen atoms only) of 0.025 Å. The use of the constraint-optimized monomer in structure #144 does not change these results.

These results confirm that the main reason our submission in stage 1 did not result in a match was that we used the (folded) equilibrium monomer not only in the stage 1 aiFF development, but also in CSPs, while the experimental monomer is almost planar (see SI1 post-submission discussion).

IV. TARGET XXXI

A. Monomer reoptimizations with constraints

The monomers from the CCDC-supplied polymorphs were optimized with constraints using the PBE+D3(BJ) functional [9–11] and the aug-cc-pVTZ [12] basis set. To maintain monomer's

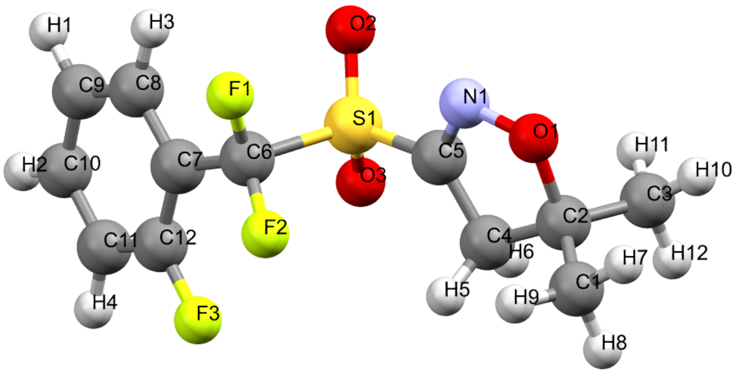

FIG. 2: Target XXXI monomer with atoms numbered.

original shape, five dihedral angles were kept fixed during the optimization: C6-S1-C5-N1, C4-C5-S1-C6, C5-S1-C6-C7, S1-C6-C7-C8, and S1-C6-C7-C12, see Fig. 2. The remaining dihedral angles, bond angles, and bond lengths were optimized. Prior to this optimization, the energies of CCDC monomers relative to the energy of the equilibrium monomer varied by up to 554.9 kJ/mol. After the optimization, this range was reduced to 13.1 kJ/mol. At the same time, the RMSD values for the optimized structures relative to their original geometries were below 0.15 Å. Thus, while the overall structures of the CCDC monomers remained largely unchanged, the modest geometry modifications were effective in significantly narrowing the energy range.

B. Improvements of intermonomer aiFFs

To improve the performance of the phase 1 aiFF on CCDC structures, 4600 dimers were extracted from crystals using the CCfC [6] code. Out of these, 2600 dimers were extracted from the unmodified CCDC structures, while 2000 dimers were extracted from the structures employing monomers optimized with constraints and embedded into crystals as described in Sec. I. These additional points were then used together with the previous 10,672 training data points to improve the fit by incorporating a broader range of monomer configurations and dimers relevant for crystal structures.

The new converged aiFF had an RMSE of 0.245 kcal/mol for the 10,162 dimer configurations in the $E_{\rm int} < 0$ region. There were a total of 378 free parameters ($N_{\rm FP}$), so the ratio of the number

of grid points to the number of free parameters ($N_{\text{grid}}/N_{\text{FP}}$) was 40. In the PES, we found 205 local minima. For points with $E_{\text{int}} < 0$, the mean signed deviation (MSD) was -0.024 kcal/mol, indicating the absence of systematic bias in the fitting. For comparison, the stage 1 aiFF had RMSE of 0.362 kcal/mol and MSD of -0.108 kcal/mol for the final set of grid points with $E_{\text{int}} < 0$, making the stage 2 aiFF significantly more accurate.

C. Reparametrization of empirical intramonomer FFs

In order to perform flexible-monomer MD simulations, we improved the General AMBER force field (GAFF) [16]. The improvement consisted in replacing the original equilibrium bond lengths and bond angles with those from the *ab initio* minimum-energy conformer. Also, the atomic partial charges were taken from aiFF. All the remaining intramonomer FF parameters including the force constants, dihedral angles parameters, and the Lennard-Jones (LJ) parameters (including scaled 1-4 interactions) were the original GAFF parameters.

D. Lattice-energy minimizations and molecular dynamics simulations

The 100 structures provided by the CCDC (the original ones) were initially optimized through a rigid-molecule minimization of their positions with the stage 2 aiFF, employing a modified UPACK version [5, 6]. The resulting structures were subsequently expanded into supercells with a minimum length of 24 Å for each cell vector. For all subsequent simulations, a fully flexible force field was utilized where the intramolecular interactions were characterized by the FF described in Sec. IV C, while the intermolecular interactions were described by the aiFF of Sec. IV B. All MD simulations were conducted with PINY-MD [17]. Structures were initially minimized and equilibrated by running MD simulations at finite temperature ($T = 150$ K) and fixed volume for 2 ps with a time step of $\Delta t = 0.5$ fs. After the initial constant number of particles, volume, and temperature (NVT) relaxation, constant number of particles, pressure, and temperature (NPT) MD simulations at temperature ($T = 150$ K) and pressure ($P = 1$ atm) using flexible cell shape were run for 100 ps to further equilibrate the structures and for another 100 ps to obtain average positions, cell parameters, and energies. For each of the 100 structures, the average positions extracted from the NPT runs were optimized using an energy minimization technique (steepest descent method included in PINY), keeping the cell parameters fixed. The resulting supercells were then reduced back to their corresponding unit cells, yielding the structures submitted in stage 2. In these structures, the positions and cell parameters reflect averages at $T = 150$ K and $P = 1$ atm. To rank the structures, we used the average enthalpy $\langle H \rangle = \langle E \rangle + P\langle V \rangle$, where $\langle E \rangle$ is the average energy from the NPT MD simulations. Note that for this system the monomer-deformation energies were given by the modified GAFF, in contrast to all other system with such

energies obtained from *ab initio* calculations.

E. Results

In our submission of 100 structures, the experimental representatives of polymorphs A_{maj} (#98), A_{min} (#1), and C (#89) were ranked at places 21, 35, and 98 with $RMSD_{30}$ = 0.420, 0.274, and 0.397 Å, respectively. Polymorph B (#25) was actually ranked as number 1 on our list, but its large $RMSD_{30}$ of 1.953 Å disqualified this ranking. As discussed in the next subsection, these poor results must have been due to insufficient quality of our enhanced GAFF which led to polymorphs' geometries further from the experimental ones than those of the experimental representatives. The use of the intramonomer modified GAFF deformation penalties in rankings could also contribute to the problem.

F. Post-submission analysis

To better understand why our predictions were rather unsatisfactory, we show in Table III results of various different ways of ranking the structures. Some of these calculations were actually performed before the submission, but since they did not include finite temperature effects were believed to be less reliable than the submitted results listed in the last set of columns. The first set of columns are results of the simplest possible predictions: take the polymorph structures exactly as sent by CCDC and just compute their lattice energies using our aiFF and monomer-deformation penalties using DFT+D. Instead of the submitted 21/35/− rankings for $A_{maj}/A_{min}/B$, this simple approach gives 2/22/14, improving the predictions. The predictions further improve if the original monomers are substituted by constraint-optimized ones, resulting in 1/5/3, which would have been the best prediction for this target. This shows that our idea to constraint-minimize the monomers is a good one and that our aiFF is quite accurate. The third set of columns shows that rigid-monomer lattice-energy minimization makes rankings slightly worse: 1/7/5, but also slightly improves all RMSDs. Since only aiFF (and DFT+D for monomer-deformation penalties) was used in the first three columns, our aiFF clearly describes the intermonomer interactions in XXXI very well. The lower accuracy of the submitted results presented in the last set of columns can most likely be attributed to the use of the modified GAFF for the intramolecular interactions in the MD simulations and probably due to not replacing the GAFF monomer-deformation energy penalties in the final rankings by *ab initio* ones. The later point is shown in Table IV, where the second set of columns includes relative lattice enthalpies at finite temperature and pressure as submitted. The first set of columns shows $T = 0$ K lattice energies for the thermally averaged structures from the MD simulations, but with monomer-deformation penalties computed *ab initio*. The polymorph approximating the form A_{min} is now of rank 1, the one approximating A_{maj} has a reasonable

ranking of 15, and the one ranked as number 1 in the submission, which cannot be considered an approximation to form B due to its large RMSD_{14}, is not bound. The energy comparisons in Table IV are, however, not rigorous since the energies in the first column are $T = 0$ K internal energies, whereas values shown in the second column are enthalpies averaged at $T = 150$ K and $P = 1$ atm in the MD simulations.

TABLE III: RMSD_{30} and rankings for target XXXI. Tolerances of 25%/25° are used for matching criteria. The RMSD in the parentheses in the last column indicates that only 14 molecules were matching out of 30. The first two columns are single-point calculations, i.e., no crystal-geometry optimizations were performed. The crystal structures in the first column are exactly as provided by CCDC, whereas in the second column, the original monomers were replaced by constraint-optimized ones. The third column shows results from rigid-monomer optimizations. The fourth column shows the ranking according to the enthalpies averaged over fully flexible MD simulations at $T = 150$ K and $P = 1$ atm. In all calculations, aiFF was used to represent intermonomer interactions.

	single-point unmodified		single-point constraint-optimized		rigid-mono opt constraint-optimized		MD fully flexible	
polymorph	rank	RMSD_{30} [Å]	rank	RMSD_{30} [Å]	rank	RMSD_{30} [Å]	rank	RMSD_{30} [Å]
$\mathrm{A}_{\mathrm{maj}}$ (#98)	2	0.180	1	0.164	1	0.161	21	0.420
$\mathrm{A}_{\mathrm{min}}$ (#1)	22	0.332	5	0.336	7	0.310	35	0.274
B (#25)	14	0.247	3	0.252	5	0.235	1	(1.953)
C (#89)	100	0.199	91	0.239	92	0.336	98	0.397

TABLE IV: Crystal lattice energies of thermally averaged structures with *ab initio* monomer deformation penalties and averaged enthalpies at $T = 150$ K and $P = 1$ atm from MD simulations. E_{latt} is the lattice energy and $\Delta E_{\mathrm{latt}}/\Delta H_{\mathrm{latt}}$ is the lattice energy/enthalpy relative to the global minimum polymorph.

Struct	E_{latt} (kJ/mol)	ΔE_{latt} (kJ/mol)	Rank	Submitted ΔH_{latt} (kJ/mol)	Rank
$\mathrm{A}_{\mathrm{maj}}$ (#98)	-71.871	36.691	15	7.366	21
$\mathrm{A}_{\mathrm{min}}$ (#1)	-108.562	0	1	9.933	35
B (#25)	34.329	142.892	43	0	1
C (#89)	1171.691	1280.253	87	21.489	98

During the test, we also performed calculations using pDFT+D. Single-point calculations were

done on the CCDC crystals with monomers replaced by constraint-optimized ones using QE [14, 15], the PBE functional [9], the D3 dispersion correction [10], and pseudopotentials [18]. We set plane-wave energy cutoff at 1224.513 eV for wave functions and 8163.422 eV for charge densities. The Monkhorst-Pack [19, 20] k-point mesh with 0.2 Å^{-1} sampling was utilized. These pDFT+D calculations were applied to rank the crystals. Forms A_{maj}, A_{min}, and B crystals were ranked within the top five, which agrees well with the results in the second column in Table III.

V. TARGET XXXII

A. Monomer reoptimizations with constraints

We employed the PBE+D3(BJ) [9, 10] method and the aug-cc-pVTZ basis set [12, 21] to optimize the monomers in the 500 polymorphs on the CCDC list for system XXXII. Our optimization strategy was to constrain 89 of the 204 dihedral angles, allowing the rest of the molecular structure—including the remaining dihedral angles, bond angles, and bond lengths—to relax. Target XXXII has 11 soft dihedral angles and these are among the 89 frozen ones. The remaining dihedral angles were frozen for computational efficiency. These are the angles involving non-hydrogen atoms in planar aromatic rings and we checked that these angles had expected values in the monomers from CCDC polymorphs. As described in Sec. I, this optimization was performed due to our observation that certain monomers in CCDC polymorphs have energies significantly higher than that of the equilibrium monomer, reaching up to 1232 kJ/mol above the energy of the equilibrium monomer. Before the constrained optimizations, 77% of monomers had energies relative to the gas-phase global equilibrium monomer larger than 50 kJ/mol. The optimization reduced this number to just 25% monomers above this threshold. Despite these large energetic changes, the average RMSD (non-hydrogen atoms only) between the original and constraint-optimized monomer geometries was only 0.11 Å, while the largest change was 0.72 Å.

In stage 1, the monomer used to generate the aiFF was not optimized using an *ab initio* method. In order to determine a more accurate global minimum monomer, we used the Conformator package [22] and generated eight conformers. These approximate conformers were further optimized using PBE+D3(BJ) with the aug-cc-pVTZ basis set implemented in the ORCA package. We used the global minimum conformer obtained in this way to compute monomer-deformation penalties in crystals, but it was not employed for the aiFF improvement.

B. Improvements of intermonomer aiFFs

We extracted additional 3,284 dimers from 500 structures provided by CCDC (with the original monomers replaced by constraint-optimized ones) to improve the performance of the stage 1 aiFF.

The clusters cut from crystals (CCfC) [6] code was used to extract the dimers. These supplementary grid-points were then merged with the training data points from phase 1 (13,852 grid points) to refine the aiFF. In the region $E_{\text{int}} < 0$, this new aiFF had the RMSE of 0.63 kcal/mol for the 10,585 dimer configurations in this region. Although this RMSE may appear to be rather significant, the ratio of this error to the global minimum depth is only 2.1%. The total number of free parameters (N_{FP}) stands at 460, so the ratio of the number of grid points to the number of free parameters, $N_{\text{grid}}/N_{\text{FP}}$, is 37.4.

C. Crystal structure rankings

In order to embed the constraint-optimized monomers into the crystal structures provided by CCDC, we matched the COMs and principal axes of rotation as described in Sec. I. After this procedure, a customized version of UPACK [6] was used to minimize the lattice energies of such polymorphs (i.e., the polymorphs obtained as described above were the starting points for such optimizations) keeping the monomers rigid. The lattice energies from such rigid-monomer minimizations included the aiFF part and the correction for the monomer-deformation relative to the equilibrium monomer. This correction was obtained by calculating the difference between the gas-phase energies of the two monomers using the PBE0+D3(BJ) method with the def2-TZVP basis set in the ORCA software package. When analyzing our results for the submission of stage 2, we found that 11 UPACK optimizations ended up with positive lattice energies. Another 9 runs, for all structures with $Z' > 1$, failed due to an input mistake. Since there was not enough time to fix these problems, we placed these structures at the end of the list (positions 481 to 500) with their original geometries as sent by CCDC. For $Z' > 1$ structures, we set the lattice energy to zero (284.19 kJ/mol above the energy of the global minimum). This led to the final rankings for target XXXII as submitted.

D. Results

CCDC decided to include in the comparisons two polymorphs of target XXXII: A_{maj} and low-temperature (LT) form B (denoted as B_{LT}). Our rank for the former, #317 on the CCDC list, was 23 with $\text{RMSD}_{30} = 0.261$ Å (with tolerances 25% and 25°) relative to the experimental crystal. Form B_{LT} turned out to be a $Z' = 2$, so it was one of the unranked structures (a structures for which UPACK calculations were incorrect, the number on the list, 487, is not a rank).

E. Post-submission analysis

Our ranking of form $A_{\rm maj}$ was reasonably good for a monomer of this size and flexibility. The structure #232 which was the representative of form $B_{\rm LT}$ was not ranked due to a mistake in running UPACK for crystals with $Z' > 1$. We repeated the runs for such crystals after the submission, obtaining rank 30 with $\mathrm{RMSD}_{30} = 0.334$ Å (tolerances 25% and 25°). The UPACK rigid-monomer optimization very significantly improved the rankings relative to those of the original CCDC structures with the monomers replaced by constraint-optimized ones from 116 to 23 for Form $A_{\rm maj}$ and 241 to 30 for Form $B_{\rm LT}$. This indicates good quality of our aiFF.

During the test period, we also calculated the lattice energies of the crystals using pDFT+D. We performed these calculations utilizing the PBE+D3(BJ) functional as implemented in the VASP software package [8]. These were single-point calculations (no geometry optimizations) on the CCDC polymorphs with monomers replaced by the constraint-optimized ones. The plane-wave energy and charge density cutoffs used were 600 eV and 4800 eV respectively. The k-spacing used is 0.15 $Å^{-1}$. This procedure was aimed to assess how the ranking from the aiFF-based UPACK minimization method correlates with pDFT+D ranking. The correlation was low, and we decided that the aiFF-based results were more reliable. Thus, pDFT+D calculations had no impact on our submission. However, the pDFT+D rankings turned out to be better. The experimental structures $A_{\rm maj}$ and $B_{\rm LT}$ were ranked by pDFT+D as numbers 12 and 13, respectively. When similar pDFT+D calculations were done on the structures after UPACK minimizations (performed for #232 after the submission), the ranking were 2 and 9, respectively. This should be compared with aiFF-based rankings, 23 and 30, for exactly the same structures. Such an improvement by pDFT+D calculations is consistent with the aiFF@CSPs protocol of Ref. [6] which includes pDFT+D single-point calculations on structures from rigid-monomer UPACK minimizations.

VI. TARGET XXXIII

A. Monomer reoptimizations with constraints

For the reasons discussed in Sec. I, we performed constrained optimizations of the anion of sulfamethoxazole (S). For the other monomer, the morpholine (M) cation, the energies of the monomer structures in the CCDC-provided polymorphs were within a reasonable range and we have not constraint-optimized this monomer. Monomer S has 3 significant soft degrees of freedom. Two of them determine the orientations of the isoxazole and benzene functional groups, and the third is the rotation around the -S1-N2- bond, see Fig. 3. Consequently, these soft torsional coordinates were initially immobilized in the optimization to maintain overall shape of S similar to the shape it had in the crystals provided by CCDC. The constrained optimization of S was

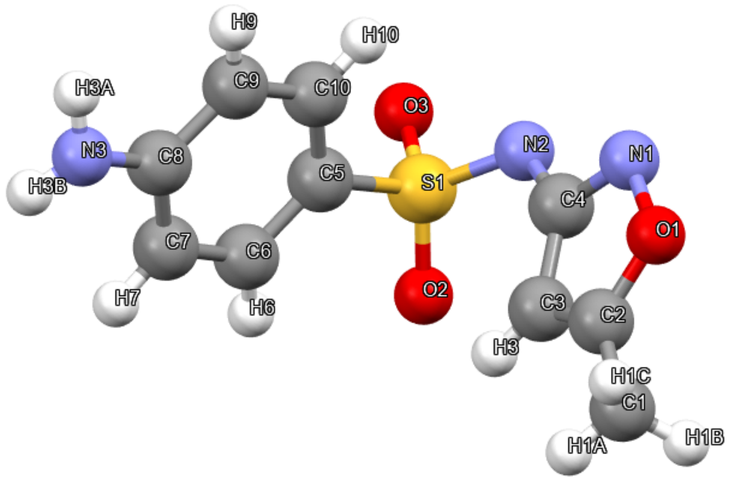

FIG. 3: Atom label scheme used by CCDC for monomer S of XXXIII.

performed in ORCA-4.2.1 [4, 13] using PBE0+D3 [1, 9, 10] with the aug-cc-pVTZ [12] basis set by keeping the five torsional angles related to these degrees of freedom fixed at the values in the CCDC-provided polymorphs: N1-C4-N2-S1, C3-C4-N2-S1, C4-N2-S1-C5, N2-S1-C5-C10, and N2-S1-C5-C6, see Fig. 3. The torsions involving the -NH_2 and -CH_3 groups were not constrained. The constrained optimizations led to the reduction of the number of monomers that had energies larger than 15 kJ/mol above the energy of the global minimum from 30% to 7%. The average RMSD change was 0.048 Å.

B. Improvements of intermonomer aiFFs

For stage 2, aiFFs developed in stage 1 were improved by adding the dimers extracted from the CCDC-provided polymorphs (with the original monomers). For the MM, SS, and MS aiFFs, the grid set used in stage 1 was augmented by 3996, 3996, and 6020 grid points. 1000 grid-points

were removed from the stage 1 SS training set since these points included way too short close-contact distances (the main reason for the very large RMSE of this fit). Consequently, the training sets used for MM, SS, and MS aiFFs in stage 2 consisted of 8496, 12996, and 19899 grid points, respectively. The RMSEs of the MM and SS aiFFs on all grid points were 0.18 and 0.70 kcal/mol, respectively. For the MS aiFF, the RMSE was 1.57 kcal/mol in the region of $E_{\rm int} < 0$. For MM, there is a significant improvement compared to stage 1 that had RMSE = 2.27 kcal/mol. For the SS aiFF, the improvement is huge since the RMSE in stage 1 was several hundreds kcal/mol. The reason for such a large RMSE in stage 1 was that the aiFF development for identically charged monomers was not included in the design of autoPES, see SI1. In particular, the grid generation algorithm produced very close-contact dimer configurations for which the fit errors were huge. Despite this problem, the stage 1 SS aiFF had an average error on the SS closest neighbour dimers extracted from the CCDC-provided polymorphs amounting to less than 11% for over 90% of these polymorphs. In the case of MS aiFF, the accuracy improvement was negligible, as this aiFF was already accurate in stage 1.

The accuracies of the aiFFs developed in stages 1 and 2 were further compared by calculating select dimer interaction energies in the CCDC-provided polymorphs. We selected all the dimers within the unit cells of these polymorphs and for polymorphs with small Z also the first-neighbour interactions outside unit cells to include interactions between all types of monomers. Both the fit and *ab initio* energies were computed for these dimers. Subsequently, mean absolute percentage errors (MAPEs) were calculated for all dimers considered from each polymorph. For stage 1 aiFFs, such MAPEs are larger than 10% for 4% of the CCDC-provided polymorphs, with the majority of the contribution to MAPE coming from inaccuracy in the SS aiFF. For stage 2 aiFFs, the MAPE is larger than 5% for only one of the CCDC-provided polymorphs and is larger than 2% for about 7% of these polymorphs. Clearly, the improvements of aiFFs were very substantial and the improved aiFFs were used in further steps of our stage 2 work.

C. Reparametrization of empirical intramonomer FFs

In our predictions of stage 1, we used the generic generalized AMBER force field (GAFF) [16] to describe the intramonmer interactions. In stage 2, we reparametrized GAFF for both monomers using a training set of *ab initio* monomer energies. First, the generic GAFF intermolecular and intramolecular parameters were assigned using the Antechamber software as implemented in AmberTools20 [23], and were used as a starting point for fitting the intramolecular force field. Point charges were taken from the aiFF intermolecular potential.

Next, hard degrees of freedom (bonds, bond angles, and improper torsions) were fitted using the Joyce intramolecular FF reparametrization package [24]. Note that this step uses only the data from an *ab initio* calculation for the equilibrium monomer: the equilibrium geometry, gradients, and

Hessian, and results in new values of both the FF equilibrium parameters and the force constants. Afterwards, both flexible torsions and improper torsions were fitted using the AMBER Paramfit software [25] (thus, improper torsions from Joyce were discarded). These parameters were fit to 2000 reference DFT energies for each monomer. Monomer geometries for these DFT calculations were taken from the CCDC-provided polymorphs as well as from the polymorphs generated by us in stage 1. All reference DFT calculations for the tailor-fit GAFF were performed using Gaussian 16 [26] with PBE0+D3 [1, 9, 10]/aug-cc-pVDZ [12]. Overall, the mean unsigned error (MUE) of the new FF monomer energies relative to the *ab initio* energies of the training sets was 1.53 kcal/mol for S and 0.30 kcal/mol for M. The relatively large errors for S may stem from the inability of the GAFF functional form to sufficiently capture conformation-dependent charge flow in the molecule, which is large due to the ionic nature of the molecule. Also, note that the Lennard-Jones parameters were not optimized, but their generic values were used. However, these energies do represent a substantial improvement compared to the standard GAFF intramolecular parameters, which exhibited an overall MUE of 7.3 kcal/mol and 0.88 kcal/mol for S and M, respectively. The tailor-fit GAFF intramolecular potentials were utilized for the subsequent crystal minimizations and MD equilibrations.

D. Lattice energy minimizations with UPACK

The CCDC-provided polymorphs with monomers replaced by constraint-optimized ones were optimized in three steps: (*i*) using the generic GAFF for intermonomer interactions and tailor-fit GAFF for intramonomer interactions, flexible-monomer minimizations were performed with UPACK [5, 6]; (*ii*) rigid-monomer UPACK minimization were then performed using aiFFs starting from the polymorph structures from step (i); (*iii*) the polymorph structures from step (ii) were subsequently employed as the starting points for supercell minimization and molecular dynamics (MD) simulations as described below.

E. Supercell energy minimizations and molecular dynamics simulations

Supercell energy minimizations and MD simulations were performed sequentially using PINY-MD [17]. We note that if a crystal simulation resulted in either unphysical close contacts or a decrease of over 200 kJ/mol in intermolecular energy after an MD simulation step, the structure was deemed to reside in an unphysical hole in the potential. In these cases, the structure was excluded from the set and the potential energy of the previous step was used for ranking. In all steps described below, aiFF was used to represent the intermonomer interactions and the tailor-fit GAFF to represent intramonomer interactions. The crystal structures were optimized using the following steps:

1. Supercell energy minimizations (0 K simulations) were performed allowing the molecules to deform, change their positions and orientations in the supercell, and the cell shape was also varied. The starting points for these minimization were the structures from Sec. VI D(ii). The resulting molecular positions and energies from this step were used for the stage 2 submission for the structures that exhibited holes during step 2 below.

2. The minimized supercells were then equilibrated using NVT MD simulations at 100 K for 20 ps using a time step of 1.0 fs. The resulting averaged molecular positions and energies from this step were used for the submission of stage 2 for the structures that exhibited holes in step 3 below.

3. Structures were then equilibrated using flexible-cell isothermal-isobaric (NPTF) MD simulations at 100 K and 1 atm for 100 ps with a time step of 1.0 fs to obtain MD average molecular positions, cell parameters, and potential energies.

4. The resulting structures (taken from the last successful step in each case) were then reduced to their corresponding P1 unit cells, and the symmetry was assigned using the PLATON software [27]. The resulting crystal structures constituted our submission for stage 2, ranked as described in Sec. VI G.

F. pDFT+D calculations

We used the pDFT+D calculations as a supplementary tool to compare with the rankings produced by the aiFFs. The pDFT+D single-point calculations, which did not include any geometry optimizations, were carried out for all CCDC-provided polymorphs with the monomers replaced by the constraint-optimized ones. We used the PBE functional [9] augmented with the D3 dispersion correction [10] and pseudopotentials [18] as implemented in QE [14, 15]. We chose a plane-wave energy cutoff of 340 eV for wave functions and 3061 eV for charge densities. The Monkhorst-Pack [19, 20] k-point mesh was used, applying a sampling rate of 0.5 Å^{-1}. The comparisons of the rankings from the two approaches were, however, inconclusive, so that the pDFT+D calculations played no role in our stage 2 submissions.

G. Final energy rankings

The final energy ranking accounts for both the intermolecular and intramolecular contributions to the total crystal energy. The structures for each crystal are taken from the last successful simulation step of Sec. VI E. The intermolecular energies reflect the aiFF contributions for each crystal using the averaged structure of the unit cell. For intramolecular energy contributions, the

average monomers in each unit cell were subjected to constrained DFT optimizations (as described above). This step was intended to partly compensate the deficiencies of the tailor-fit intramonomer GAFF. The relative DFT energies from the these minimization were then used in the final ranking to determine monomer-deformation penalties.

H. Results

The polymorphs resulting from our optimizations of the experimental representatives of forms A and B, #233 and #452 in the CCDC list, were ranked at positions 349 and 132, respectively. The $RMSD_{30}$ values relative to the experimental structures were 0.322 Å and 0.506 Å, respectively. Thus, we have correctly predicted the relative thermodynamic stability of these two forms.

I. Post-submission analysis

For target XXXIII, the experimental representatives were quite close to the experimental crystals, with $RMSD_{30}$'s of 0.387 and 0.254 Å for forms A and B, respectively. Our optimizations of these CCDC-provided polymorphs (including the separate constrained minimization of monomers) actually slightly improved the $RMSD_{30}$ value for A, to 0.322 Å, but made it worse, 0.506 Å, for B. In any case, both final structures have reasonable $RMSD_{30}$'s. However, our energy rankings were poor. In the post-submission analysis, we obtained better rankings: A at 201 and B at 24 from lattice energies computed in the same way as in the submission, but for the exact original structures provided by CCDC. Rankings were also better with constraint-optimized monomers (272 and 19). UPACK rigid-monomer minimization improved the former ranking, to 244, but made the latter one much worse, 283. Thus, our significant effort invested in generating the tailor-fitted GAFF and performing UPACK minimizations and MD simulations was counterproductive. However, even the rankings based on the original CCDC structures are not accurate enough.

Our pDFT+D calculations on the CCDC-provided structures with monomers substituted by constraint-optimized ones (see Sec. VI F) ranked the experimental polymorphs A and B at 123 and 1, respectively. Analogous calculations with the original monomers gave ranks 76 and 2, respectively. This can be compared with aiFF/DFT+D 272 and 19 ranks of the same structures. The excellent pDFT+D result for B was obtained despite using the lowest level of pDFT+D: the non-hybrid PBE functional, a coarse k-point mesh, and loose cutoffs. This result may be partly fortuitous, as indicated by not so good ranking of A, but may suggest that for these crystals the many-body polarization effects may be important due to the ionic nature of the monomers. These effects were found small in Ref. [6], but none of the monomers considered there were ionic. It is possible to generate polarizable aiFFs using autoPES and use them in UPACK.

VII. SUMMARY

To our surprise, when the experimental monomer structures were revealed, it turned out that the CCDC lists included experimental representatives whose crystals structures very close to the experimental ones, including the geometries of monomers. As discussed earlier, the CCDC lists included representative structures, i.e., the structures being intentionally reasonably close to the experimental polymorphs. This was expected, but the level of closeness was not, at least to our team.

The results for monomers from representative structures are presented in Table V. The RMDSs of the experimental representatives relative to the actual monomers in experimental crystals range between 0.0 and 0.1 Å for 10 out of the 15 monomers in experimental representatives. In the remaining 5 cases, RMSDs range between 0.17 and 0.29 Å. Also the energies of the monomers from experimental representatives are in most cases, 9 out of 15, in the range 9-22 kJ/mol, in contrast to the values ranging up to hundreds of kJ/mol shown in Table II. For the monomers of targets XXVII and XXXII, the range is 30-48 kJ/mol. These larger values could be expected and are related to the near-planar structures of these monomers, while the equilibrium monomers for these systems are folded. Also monomer S of XXXIII #452 is in this range, although in this case the monomer from the crystal is not significantly more planar than the equilibrium one. Here, the main two reasons for the relatively high energy and for the fact that this energy dropped to 13 kJ/mol after the constrained optimization with a very small change of RMSD were inaccurate X-H bond lengths and the fact that the -NH_2 group was essentially flat in the original monomer. In any case, these energies are still relatively small. An exception is #38 of XXVII, but it was an unintended experimental representative, so we do not need to count it. One more exception is the monomer in #89 of XXXI, which has energy as large as 555 kJ/mol. The reason here is that this is simply the experimental monomers, as seen from the 0.001 RMSD between non-hydrogen atoms. The energy is so large since the experimental X-H bond lengths, where X is any atom, are significantly different from *ab initio* ones. After the optimization of the X-H bond lengths only, this energy drops to 29 kJ/mol. Also, form C of XXXI was eventually not used in 7BT, so we will not discuss it anymore.

The stark contrast seen by comparing energetic differences between the monomers in experimental representatives and in the other polymorphs on the CCDC lists, shown in Tables V and II, respectively, should not be the case, in our opinion. The former monomers should be among the most distorted to make the test equally difficult for all types of methods.

Also the crystal structures of experimental representatives are very close to those of the corresponding experimental polymorphs, as shown in Table VI. The $RMSD_{30}$'s (see captions of Table VI for details) are between 0.18 and 0.53 Å (taking into account only the closest representative for target XXVII and ignoring form C (#89) of XXXI here and in all considerations below). Thus,

TABLE V: Comparisons of RMSDs of different types of monomers with respect to the experimental monomer (only non-hydrogen atoms) in Å. In column "original", the results are shown for monomers from the representative structures as provided by CCDC (structure number in parenthesis). Also the energies of these monomers relative to the *ab initio* equilibrium monomer are given in kJ/mol. In column "constraint-optimized" analogous results are given for the same monomers after constrained minimizations. The absolute energies of the latter monomers as well as the energies of the equilibrium monomers are given in the last column (in hartree). The methods and basis sets are the same as specified in Table II.

Target	Polymorph	original RMSD/ΔE	constraint-optimized RMSD/ΔE	$E_{\rm equil}/E_{\rm constr-opt}$
XXVII	A (#28)	0.260/15.302	0.262/1.097	-2880.886276/-2880.886068
	A (#38)	0.229/72.192	0.510/27.348	-2880.886276/-2880.875995
	A (#59)	0.261/14.717	0.260/0.592	-2880.886276/ -2880.886185
	A (#61)	0.285/13.977	0.287/-0.394*	-2880.886276/-2880.886561
XXVIII	A (#144)	0.021/30.198	0.025/21.475	-3673.074491/-3673.066564
XXXI	$A_{\rm maj}$ (#98)	0.083/17.539	0.070/8.700	-1441.671572/-1441.668258
	$A_{\rm min}$ (#01)	0.166/12.899	0.176/5.481	-1441.671572/-1441.669484
	B (#25)	0.061/10.011	0.062/6.348	-1441.671572/-1441.669154
	C (#89)	0.001/554.93	0.132/6.208	-1441.671572/-1441.669207
XXXII	$A_{\rm maj}$ (#317)	0.070/42.367	0.089/36.260	-2459.456556/-2459.442764
	$B_{\rm LT}$ (#232)	0.095/48.000	0.106/43.300	-2459.456556/-2459.440058
XXXIII	A (#233) M	0.024/9.200	0.016/0.200	-287.947824/-287.947903
	A (#233) S	0.110/21.922	0.107/1.895	-1174.512790/-1174.506170
	B (#452) M	0.018/13.60	0.014/0.080	-287.947824/-287.947853
	B (#452) S	0.034/40.984	0.067/13.044	-1174.512790/-1174.499590

*The reason for the negative energy difference is that the reference structures is the global-minimum monomer of stage 1, which was optimized in the def2-SVP basis set, but all monomer energies are computed here in the def2-TZVPP basis set. The reference monomer is not anymore the global minimum structure in the larger basis set.

all experimental representative crystals had RMSDs within the acceptance range. In our opinion, this should not be the case, the representative polymorphs should have required optimization to get within the range.

TABLE VI: Comparisons of ranks and $RMSD_{30}$'s (in Å) of representative polymorphs (the structure number is given in parentheses) with different types of monomers and various types of crystal lattice optimizations. RMSDs are with respect to the experimental polymorphs (only non-hydrogen atoms, except for XXVII: core-only non-hydrogen atoms). At least 30 monomers matched, except for the RMSD enclosed in parentheses where only 14 monomers matched, and the tolerances were 0.25%/25°. The column "original" shows single-point (no optimizations) rankings of the experimental representatives using the exact structures provided by CCDC. The column "constr-opt" shows single-point calculations rankings of the same crystals as in the previous column but with the monomers replaced by the constraint-optimized ones. The next two columns show results from lattice energy minimizations with rigid monomers ("rigid-mono opt") and from MD simulations with flexible monomers ("flex-mono opt").

Target	Polymorph	original rank/RMSD	constr-opt rank/RMSD	rigid-mono opt rank/RMSD	flex-mono opt rank/RMSD
XXVII	A (#28)	**1**/0.530	**1**/0.531	*3/**0.437***	
	A (#38)	87/0.804	85/0.804	*70/0.554*	
	A (#59)	5/0.828	5/0.828	*4/0.841*	
	A (#61)	3/0.569	2/0.569	*11/0.468*	
XXVIII	A (#144)	**1**/0.234	**1**/0.234	***1/0.200***	
XXXI	A_{maj} (#98)	2/0.180	**1**/0.164	**1/0.161**	*21/0.420*
	A_{min} (#01)	22/0.332	**5**/0.336	7/0.310	*35/**0.274***
	B (#25)	14/0.247	**3**/0.252	5/**0.235**	*1/(1.953)*
	C (#89)	100/**0.199**	**90**/0.239	92/0.336	*98/0.397*
XXXII	A_{maj} (#317)	201/0.264	116/0.266	***23/0.261***	
	B_{LT} (#232)	307/**0.298**	241/0.303	**30**/0.334*	
XXXIII	A (#233)	**201**/0.387	272/0.383	244/0.326	*349/**0.322***
	B (#452)	24/**0.254**	**19**/0.264	283/0.665	*132/0.506*

*Structure #232 was not ranked in the submission, see Sec. V D.

Due to the construction of the CCDC lists, our huge effort invested in CSPs with flexible monomers was counterproductive, as shown in Table VI. The columns in this table show rankings and $RMSD_{30}$'s for the following crystal structures: (*a*) the original ones in CCDC lists; (*b*) as (a) but with monomers substituted by constraint-optimized monomers; (*c*) structures from

rigid-monomer optimizations of structures (b); structures from flexible-monomer MD simulations starting from structures (a) or (b). In each case, the rankings are based on lattice energies with intermonomer contributions from aiFF and monomer deformation penalties computed *ab initio*. The only exception are flexible-monomer MD simulations for target XXXI where the penalties were from the modified GAFF. The submitted results are shown in italics and the best predictions in bold face. The predictions of method (d) are the worst.

Table VI shows that in 3 cases, the best rankings are in column (a), in 6 cases in column (b), in 4 cases in column (c), and 0 cases in column (d). The corresponding average rankings are 86, 73, 66, and 108. The averaged RMSD_{30} are 0.30, 0.25, 0.32, 0.70 Å. Since the computational effort increases with column number, the outcome is clearly not proportional to effort. Overall, the biggest gain in rankings was coming from replacing the original monomers by constraint-optimized ones: except for XXXIII #233, the rankings improved or remained the same. UPACK rigid monomer optimization led to small changes of rankings for targets XXVII, XXVIII, and XXXI (slight overall worsening), but significantly improved the rankings for XXXII. Thus, it was worth the effort, except for target XXXIII. The reason is that for this target many-body induction effects, not included in our aiFFs, are important (see the discussion in Sec. VI I). The "best-of" rankings for consecutive polymorphs in Table VI are 1, 1, 1, 5, 3, 23, 30, 201, 19, a reasonably good predictions taking into account the complexity of targets. As discussed for individual targets, these ranking could still be significantly improved by performing pDFT+D single-point calculations on these structures, in the spirit of the aiFF@CSPs method of Ref. [6].

[1] C. Adamo and V. Barone, Toward reliable density functional methods without adjustable parameters: The PBE0 model, J. Chem. Phys. **110**, 6158 (1999).
[2] S. Grimme, Density functional theory with London dispersion corrections, WIREs Comput. Mol. Sci. **1**, 211 (2011).
[3] F. Weigend and R. Ahlrichs, Balanced basis sets of split valence, triple zeta valence and quadruple zeta valence quality for H to Rn: Design and assessment of accuracy, Phys. Chem. Chem. Phys. **7**, 3297 (2005).
[4] F. Neese, Software update: the ORCA program system, version 4.0, WIREs Comput. Mol. Sci. **8**, e1327 (2018).
[5] B. P. van Eijck and J. Kroon, UPACK program package for crystal structure prediction: Force fields and crystal structure generation for small carbohydrate molecules, J. Comp. Chem. **20**, 799 (1999).
[6] R. Nikhar and K. Szalewicz, Reliable crystal structure predictions from first principles, Nature Comm. **13**, 3095 (2022).
[7] J. Sun, A. Ruzsinszky, and J. P. Perdew, Strongly constrained and appropriately normed semilocal density functional, Phys. Rev. Lett. **115**, 036402 (2015).
[8] G. Kresse and *et al.*, VASP: Vienna ab initio simulation package (2021).
[9] J. P. Perdew, K. Burke, and M. Ernzerhof, Generalized gradient approximation made simple, Phys. Rev. Lett. **77**, 3865 (1996).
[10] S. Grimme, J. Antony, S. Ehrlich, and H. Krieg, A consistent and accurate ab initio parametrization of density functional dispersion correction (DFT-D) for the 94 elements H-Pu, J. Chem. Phys. **132**, 154104 (2010).
[11] S. Grimme, S. Ehrlich, and L. Goerigk, Effect of the damping function in dispersion corrected density functional theory, J. Comp. Chem. **32**, 1456 (2011).
[12] R. A. Kendall, T. H. Dunning, Jr., and R. J. Harrison, Electron affinities of the first-row atoms revisited. Systematic basis sets and wave functions, J. Chem. Phys. **96**, 6796 (1992).
[13] F. Neese, The ORCA program system, WIREs Comp. Mol. Sci. **2**, 73 (2012).
[14] P. Giannozzi, S. Baroni, N. Bonini, M. Calandra, R. Car, C. Cavazzoni, D. Ceresoli, G. L. Chiarotti, M. Cococcioni, I. Dabo, A. D. Corso, S. de Gironcoli, S. Fabris, G. Fratesi, R. Gebauer, U. Gerstmann, C. Gougoussis, A. Kokalj, M. Lazzeri, L. Martin-Samos, N. Marzari, F. Mauri, R. Mazzarello, S. Paolini, A. Pasquarello, L. Paulatto, C. Sbraccia, S. Scandolo, G. Sclauzero, A. P. Seitsonen, A. Smogunov, P. Umari, and R. M. Wentzcovitch, QUANTUM ESPRESSO: a modular and open-source software project for quantum simulations of materials, J. Phys.: Condens. Matter **21**, 395502 (2009).
[15] P. Giannozzi, O. Andreussi, T. Brumme, O. Bunau, M. B. Nardelli, M. Calandra, R. Car, C. Cavaz-

zoni, D. Ceresoli, M. Cococcioni, N. Colonna, I. Carnimeo, A. D. Corso, S. de Gironcoli, P. Delugas, R. A. DiStasio, A. Ferretti, A. Floris, G. Fratesi, G. Fugallo, R. Gebauer, U. Gerstmann, F. Giustino, T. Gorni, J. Jia, M. Kawamura, H.-Y. Ko, A. Kokalj, E. Küçükbenli, M. Lazzeri, M. Marsili, N. Marzari, F. Mauri, N. L. Nguyen, H.-V. Nguyen, A. O. de-la Roza, L. Paulatto, S. Poncé, D. Rocca, R. Sabatini, B. Santra, M. Schlipf, A. P. Seitsonen, A. Smogunov, I. Timrov, T. Thonhauser, P. Umari, N. Vast, X. Wu, and S. Baroni, Advanced capabilities for materials modelling with Quantum ESPRESSO, J. Phys.: Condens. Matter **29**, 465901 (2017).

[16] J. Wang, R. M. Wolf, J. W. Caldwell, P. A. Kollman, and D. A. Case, Development and testing of a general Amber force field, J. Comp. Chem. **25**, 1157 (2004).

[17] M. E. Tuckerman, D. Yarne, S. O. Samuelson, A. L. Hughes, and G. J. Martyna, Exploiting multiple levels of parallelism in molecular dynamics based calculations via modern techniques and software paradigms on distributed memory computers, Comput. Phys. Commun. **128**, 333 (2000).

[18] A. M. Rappe, K. M. Rabe, E. Kaxiras, and J. D. Joannopoulos, Optimized pseudopotentials, Phys. Rev. B **41**, 1227 (1990).

[19] H. J. Monkhorst and J. D. Pack, Special points for Brillouin-zone integrations, Phys. Rev. B: Condens. Matter **13**, 5188 (1976).

[20] J. D. Pack and H. J. Monkhorst, "Special points for Brillouin-zone integrations"—a reply, Phys. Rev. B: Condens. Matter **16**, 1748 (1977).

[21] D. E. Woon and T. H. Dunning, Jr., Gaussian basis sets for use in correlated molecular calculations. III. The atoms aluminum through argon, J. Chem. Phys. **98**, 1358 (1993).

[22] N.-O. Friedrich, F. Flachsenberg, A. Meyder, K. Sommer, J. Kirchmair, and M. Rarey, Conformator: A novel method for the generation of conformer ensembles, J. Chem. Inf. Model. **59**, 731 (2019).

[23] D. A. Case, K. Belfon, I. Y. Ben-Shalom, S. R. Brozell, D. S. Cerutti, T. E. Cheatham, III, V. W. D. Cruzeiro, T. A. Darden, R. E. Duke, G. Giambasu, M. K. Gilson, H. Gohlke, A. W. Goetz, R. Harris, S. Izadi, S. A. Izmailov, K. Kasavajhala, A. Kovalenko, R. Krasny, T. Kurtzman, T. S. Lee, S. LeGrand, P. Li, C. Lin, J. Liu, T. Luchko, R. Luo, V. Man, K. M. Merz, Y. Miao, O. Mikhailovskii, G. Monard, H. Nguyen, A. Onufriev, F. Pan, S. Pantano, R. Qi, D. R. Roe, A. Roitberg, C. Sagui, S. Schott-Verdugo, J. Shen, C. L. Simmerling, N. R. Skrynnikov, J. Smith, J. Swails, R. C. Walker, J. Wang, L. Wilson, R. M. Wolf, X. Wu, Y. Xiong, Y. Xue, D. M. York, and P. A. Kollman, AMBER 2020, University of California, San Francisco.

[24] V. Barone, I. Cacelli, N. De Mitri, D. Licari, S. Monti, and G. Prampolini, Joyce and Ulysses: integrated and user-friendly tools for the parameterization of intramolecular force fields from quantum mechanical data, Phys. Chem. Chem. Phys. **15**, 3736 (2013).

[25] R. M. Betz and R. C. Walker, Paramfit: Automated optimization of force field parameters for molecular dynamics simulations, JCC **36**, 79 (2015).

[26] M. J. Frisch, G. W. Trucks, H. B. Schlegel, G. E. Scuseria, M. A. Robb, J. R. Cheeseman, G. Scal-

mani, V. Barone, G. A. Petersson, H. Nakatsuji, X. Li, M. Caricato, A. V. Marenich, J. Bloino, B. G. Janesko, R. Gomperts, B. Mennucci, H. P. Hratchian, J. V. Ortiz, A. F. Izmaylov, J. L. Sonnenberg, D. Williams-Young, F. Ding, F. Lipparini, F. Egidi, J. Goings, B. Peng, A. Petrone, T. Henderson, D. Ranasinghe, V. G. Zakrzewski, J. Gao, N. Rega, G. Zheng, W. Liang, M. Hada, M. Ehara, K. Toyota, R. Fukuda, J. Hasegawa, M. Ishida, T. Nakajima, Y. Honda, O. Kitao, H. Nakai, T. Vreven, K. Throssell, J. A. Montgomery, Jr., J. E. Peralta, F. Ogliaro, M. J. Bearpark, J. J. Heyd, E. N. Brothers, K. N. Kudin, V. N. Staroverov, T. A. Keith, R. Kobayashi, J. Normand, K. Raghavachari, A. P. Rendell, J. C. Burant, S. S. Iyengar, J. Tomasi, M. Cossi, J. M. Millam, M. Klene, C. Adamo, R. Cammi, J. W. Ochterski, R. L. Martin, K. Morokuma, O. Farkas, J. B. Foresman, and D. J. Fox, Gaussian 16 Revision C.01 (2016), Gaussian Inc. Wallingford CT.

[27] A. L. Spek, Structure validation in chemical crystallography, Acta Crystallogr. D **65**, 148 (2009).
