# Supplementary material for: The seventh blind test of crystal structure prediction: structure ranking methods
Source: Acta Crystallogr B Struct Sci Cryst Eng Mater. 2024 Oct 17;80(Pt 6):548–74. doi: 10.1107/S2052520624008679 (PMC11789160; doi:10.1107/S2052520624008679)
Supplement: Supplementary file 3 [file b-80-00548-sup3.zip › SI-C_theoretical_structures_CCDC_lists_and_predictions/ranked_structure_sets/Group24_SLPrice/XXVII-2-SLPrice_CSPBT7/7 CSP Blind Test - submission form - link Survey.pdf]

CCDC
advancing structural science

7 CSP Blind Test - submission form - link

* 1. Target compound identifier

XXVII

* 2. Submission period (1 or 2)

2

* 3. Name of the attached folder (.zip)

XXVII-2-SLPrice_CSPBT7.zip

10 of 10 answered

ORCID ID (if available)

Sarah L. Price, University College London, 0000-0002-1230-7427
Louise S. Price, University College London, 0000-0002-7633-1987

* 5. Lead author name

Sarah L Price

* 6. Primary contact email address (this is the address the CCDC will use to contact regarding the submission)

s.l.price@ucl.ac.uk

* 7. A brief description of the methodology applied.

"For each crystal structure provided by the CCDC, the molecular conformation was extracted and the bond lengths optimized with GAUSSIAN and the PBE0 method and 6-311G(d,p) basis set. The bond angles and dihedral angles were all kept fixed at the starting values. This conformation was then kept rigid throughout, and the ab initio molecular calculation used to provide the intramolecular energy contribution and multipoles. DMACRYS was then used to optimize the intermolecular lattice energy, with the atomic multipoles modelling the electrostatic contribution and a repulsion-dispersion potential with different parameters for the different parts of the molecule. For the aliphatic part of the molecule the FIT potential parameters were used with the UFF parameters for silicon, and Graeme Day's anisotropic potential parameters were used for the aromatic part of molecule.
The free energy corrections were calculated for a rigid-molecule using the harmonic approximation on the same intermolecular potential energy surface, using the codes of Nyman and Day to produce the supercells to converge the Brillouin zone. "

* 8. Computational time taken to generate the attached results (CPU hours)

1963

* 9. Outline of computational hardware utilised.

Clusters of various mixed nodes e.g. dual Xeon E5-2650v3, all more than 5 years old.

10. Supporting statement(s)

"This molecule presented challenges with assessing similarities between structures, since the highly symmetrical nature of the molecule made matching with Mercury's Crystal Packing Similarity tool problematic. For example, structures that only differed slightly in the molecular bond lengths only matched 3/15 molecules.
It was not possible to minimize the lattice energy of these crystal structures with CrystalOptimizer balancing the intra- and inter-molecular components, and so we have not been able to optimize the molecular conformations within the crystal packings. Keeping the conformations fixed as we have done has given rise to an unfeasibly large range of intramolecular energy penalties and lattice energies."

DONE

Powered by
SurveyMonkey®
See how easy it is to create a survey.

Privacy & Cookie Notice
