# Supplementary material for: The seventh blind test of crystal structure prediction: structure ranking methods
Source: Acta Crystallogr B Struct Sci Cryst Eng Mater. 2024 Oct 17;80(Pt 6):548–74. doi: 10.1107/S2052520624008679 (PMC11789160; doi:10.1107/S2052520624008679)
Supplement: Supplementary file 3 [file b-80-00548-sup3.zip › SI-C_theoretical_structures_CCDC_lists_and_predictions/ranked_structure_sets/Group24_SLPrice/XXVII-2-SLPrice_CSPBT7/XXVII-2-SLPrice_CSPBT7_Duplicates.docx]

Duplicate structures - XXVII-2-SLPrice_CSPBT7 (hydrogens not included in comparisons)

| Retained structure | Discarded structure(s) |
| --- | --- |
| XXVII_structure_28.cif | XXVII_structure_61.cif |
| XXVII_structure_77.cif | XXVII_structure_63.cif |

Output from Clustering script

structure CCDC61	matches structure CCDC28	with RMSD	15 =	7.7758200000000000E-002

structure CCDC63	matches structure CCDC77	with RMSD	15 = 0.19696000000000000

structure CCDC40	matches structure CCDC57	with RMSD	15 = 0.23133999999999999

|  |  |
| --- | --- |
| CCDC40 (by element) and CCDC57 (green/red) | CCDC63 (by element) and CCDC77 (red/green) This overlay is perfect, even though Mercury says it matches just 2 molecules. |
