# Supplementary material for: The seventh blind test of crystal structure prediction: structure ranking methods
Source: Acta Crystallogr B Struct Sci Cryst Eng Mater. 2024 Oct 17;80(Pt 6):548–74. doi: 10.1107/S2052520624008679 (PMC11789160; doi:10.1107/S2052520624008679)
Supplement: Supplementary file 3 [file b-80-00548-sup3.zip › SI-C_theoretical_structures_CCDC_lists_and_predictions/ranked_structure_sets/Group24_SLPrice/XXXI-2-SLPrice_CSPBT7/7 CSP Blind Test - submission form - link Survey.pdf]

advancing structural science

7 CSP Blind Test - submission form - link

* 1. Target compound identifier

XXXI

* 2. Submission period (1 or 2)

2

* 3. Name of the attached folder (.zip)

XXXI-2-SLPrice_CSPBT7.zip

10 of 10 answered

* 4. Author names, affiliations, addresses, emails, ORCID ID (if available)

Sarah L. Price, University College London, s.l.price@ucl.ac.uk, 0000-0002-1230-7427

* 5. Lead author name

Sarah L Price

* 6. Primary contact email address (this is the address the CCDC will use to contact regarding the submission)

s.l.price@ucl.ac.uk

* 7. A brief description of the methodology applied.

10 of 10 answered

"The same methodology used in Submission 1 was applied to the 100 structures provided, i.e. refinement by CrystalOptimizer using the PBE0/6-31G(d,p) charge distribution for the intramolecular energy penalty and distributed multipoles, and an exp-6 atom-atom potential with the FIT parameters for the other intermolecular lattice energy components.
The free energy corrections were calculated for a rigid-molecule using the harmonic approximation on the same intermolecular potential energy surface, using the codes of Nyman and Day to produce the supercells to converge the Brillouin zone. "

* 8. Computational time taken to generate the attached results (CPU hours)

1023

* 9. Outline of computational hardware utilised.

Clusters of various mixed nodes e.g. dual Xeon E5-2650v3, all more than 5 years old.

10. Supporting statement(s)

10 of 10 answered

"As the majority of the 100 structures provided were in our Submission 1, with none overlaying fewer than 3/15 molecules, the structure generation with our energy model appears to be reasonably successful. We were able to use the LAM databases of ab initio molecular calculations generated for Submission 1 to refine the structures in Submission 2, saving considerable CPU time.
However, our confidence in the relative lattice energy ranking is limited, because it is sensitive to choice of SO2 and F parameters, as well as the intrinsic limitations of basing the calculations on molecular electronic structure methods. The rigid-molecule approximation for the free energy corrections is clearly a gross approximation for such a flexible molecule, but does give an indication of possible temperature effects on reranking for little computational cost.
If we had been asked to rank structures for their likelihood of being observed, we would need to consider whether CCDC89 could have been stabilized by solvent in the voids during crystallization, and the possibility of entropic stabilization through disorder of the closely related structures CCDC11, 73, 32, 34 which have a sheet in common."

DONE

Powered by
SurveyMonkey®
See how easy it is to create a survey.

Privacy & Cookie Notice

10 of 10 answered
