# Supplementary material for: The seventh blind test of crystal structure prediction: structure ranking methods
Source: Acta Crystallogr B Struct Sci Cryst Eng Mater. 2024 Oct 17;80(Pt 6):548–74. doi: 10.1107/S2052520624008679 (PMC11789160; doi:10.1107/S2052520624008679)
Supplement: Supplementary file 3 [file b-80-00548-sup3.zip › SI-C_theoretical_structures_CCDC_lists_and_predictions/ranked_structure_sets/Group24_SLPrice/XXXI-2-SLPrice_CSPBT7/XXXI-2-SLPrice_CSPBT7_Duplicates.docx]

Duplicate structures - XXXI-2-SLPrice_CSPBT7

| Retained structure | Discarded structure(s) |
| --- | --- |
| XXXI_structure_30.cif | XXXI_structure_23.cif |
| XXXI_structure_17.cif | XXXI_structure_59.cif |
| XXXI_structure_70.cif | XXXI_structure_63.cif |
| XXXI_structure_74.cif | XXXI_structure_6.cif |

Output from Clustering script

structure 23-1	matches structure 30-1	with RMSD	15 = 0.21292300000000000

structure 59-1	matches structure 17-1	with RMSD	15 =	3.4421899999999998E-002

structure 63-1	matches structure 70-1	with RMSD	15 =	5.2520999999999998E-002

structure 6-1	matches structure 74-1	with RMSD	15 = 0.31212299999999998
