# Supplementary material for: The seventh blind test of crystal structure prediction: structure ranking methods
Source: Acta Crystallogr B Struct Sci Cryst Eng Mater. 2024 Oct 17;80(Pt 6):548–74. doi: 10.1107/S2052520624008679 (PMC11789160; doi:10.1107/S2052520624008679)
Supplement: Supplementary file 3 [file b-80-00548-sup3.zip › SI-C_theoretical_structures_CCDC_lists_and_predictions/ranked_structure_sets/Group24_SLPrice/XXXII-2-SLPrice_CSPBT7/7 CSP Blind Test - submission form - link Survey.pdf]

7 CSP Blind Test - submission form - link

* 1. Target compound identifier

XXXII

* 2. Submission period (1 or 2)

2

* 3. Name of the attached folder (.zip)

XXXII-2-SLPrice_CSPBT7.zip

10 of 10 answered

* 4. Author names, affiliations, addresses, emails, ORCID ID (if available)

Sarah L. Price, University College London, s.l.price@ucl.ac.uk, 0000-0002-1230-7427
Louise S. Price, University College London, l.s.price@ucl.ac.uk, 0000-0002-7633-1987

* 5. Lead author name

Sarah L Price

* 6. Primary contact email address (this is the address the CCDC will use to contact regarding the submission)

s.l.price@ucl.ac.uk

* 7. A brief description of the methodology applied.

10 of 10 answered

"The 500 CCDC-provided crystal structures were initially optimized with the same methodology as was used in Submission 1, i.e. periodic DFTB3-D3 optimization of the molecular conformations and unit cells, followed by DMACRYS rigid molecule optimization with distributed multipoles and intramolecular energy calculated from the PBE0/6-31G(d,p) wavefunction of the DFTB3-D3 optimized geometry for each structure, and the FIT potential for the repulsion-dispersion parameters. These lattice energies were used to assess the Submission 1 method. CrystalOptimizer refinement was then carried out to allow conformational changes within the lattice with an ab initio method. The intramolecular energy contribution was calculated at the PBE0/6-31G(d,p) level of theory, and the multipoles were extracted from this charge density. An exp-6 atom-atom potential with the FIT parameters was used for the other intermolecular lattice energy components.
The free energy corrections were calculated for a rigid-molecule using the harmonic approximation on the same intermolecular potential energy surface, using the codes of Nyman and Day to produce the supercells to converge the Brillouin zone."

* 8. Computational time taken to generate the attached results (CPU hours)

86668

* 9. Outline of computational hardware utilised.

Clusters of various mixed nodes e.g. dual Xeon E5-2650v3, all more than 5 years old.

10 of 10 answered

10. Supporting statement(s)

"The method of Submission 1 was originally applied, and gave lattice energies which were far lower than in our Submission 1. The PBE0/6-31G(d,p) conformational energy penalties were very high (56-181 kJ mol-1), which casts doubt on the DFTB3-D3 conformations. Therefore we have little confidence in the completeness and energy model of our original search method.
The more established CrystalOptimizer lattice energy optimization gave a lattice energy landscape which still had a very large intramolecular energy range (122 kJ mol-1), with only 10% of structures having conformations within 20 kJ mol-1 of the gas phase optimized conformation. There are more than eight plausible crystal structures with low energy. However, a molecule of this size has the potential to be trapped in a high energy conformation by the synthetic route, meaning that reliance on energy alone to predict crystal structures may be foolhardy."

DONE

Powered by
SurveyMonkey
See how easy it is to create a survey.

Privacy & Cookie Notice

10 of 10 answered
