# Supplementary material for: The seventh blind test of crystal structure prediction: structure ranking methods
Source: Acta Crystallogr B Struct Sci Cryst Eng Mater. 2024 Oct 17;80(Pt 6):548–74. doi: 10.1107/S2052520624008679 (PMC11789160; doi:10.1107/S2052520624008679)
Supplement: Supplementary file 3 [file b-80-00548-sup3.zip › SI-C_theoretical_structures_CCDC_lists_and_predictions/ranked_structure_sets/Group24_SLPrice/XXXII-2-SLPrice_CSPBT7/XXXII-2-SLPrice_CSPBT7_Duplicates.docx]

Duplicate structures - XXXII-2-SLPrice_CSPBT7

| Retained structure | Discarded structure(s) |
| --- | --- |
| XXXII_structure_1.cif | XXXII_structure_58.cif |
| XXXII_structure_105.cif | XXXII_structure_485.cif |
| XXXII_structure_488.cif | XXXII_structure_169.cif |
| XXXII_structure_174.cif | XXXII_structure_194.cif |
| XXXII_structure_53.cif | XXXII_structure_482.cif |
| XXXII_structure_27.cif | XXXII_structure_237.cif |

Output from Clustering script

structure 58-1	matches structure 1-1	with RMSD	20 = 0.34978799999999999

structure 485-1	matches structure 105-1	with RMSD	20 = 0.19098999999999999

structure 169-1	matches structure 488-1	with RMSD	20 = 0.27994400000000003

structure 194-1	matches structure 174-1	with RMSD	20 = 0.38179900000000000

structure 482-1	matches structure 53-1	with RMSD	20 = 0.35422300000000001

structure 237-1	matches structure 27-1	with RMSD	20 = 0.13350400000000001
