# Supplementary material for: The seventh blind test of crystal structure prediction: structure ranking methods
Source: Acta Crystallogr B Struct Sci Cryst Eng Mater. 2024 Oct 17;80(Pt 6):548–74. doi: 10.1107/S2052520624008679 (PMC11789160; doi:10.1107/S2052520624008679)
Supplement: Supplementary file 3 [file b-80-00548-sup3.zip › SI-C_theoretical_structures_CCDC_lists_and_predictions/ranked_structure_sets/Group24_SLPrice/XXXIII-2-SLPrice_CSPBT7/7 CSP Blind Test - submission form - link Survey.pdf]

advancing structural science

7 CSP Blind Test - submission form - link

* 1. Target compound identifier

XXXIII

* 2. Submission period (1 or 2)

2

* 3. Name of the attached folder (.zip)

XXXIII-2-SLPrice_CSPBT7.zip

10 of 10 answered

* 4. Author names, affiliations, addresses, emails, ORCID ID (if available)

Sarah L. Price, University College London, s.l.price@ucl.ac.uk, 0000-0002-1230-7427
Louise S. Price, University College London, l.s.price@ucl.ac.uk, 0000-0002-7633-1987
Rui Guo, University College London, 0000-0002-1129-4716

* 5. Lead author name

Sarah L Price

* 6. Primary contact email address (this is the address the CCDC will use to contact regarding the submission)

s.l.price@ucl.ac.uk

* 7. A brief description of the methodology applied.

10 of 10 answered

"The same methodology used in Submission 1 was applied to the 500 CCDC-provided structures, i.e. CrystalOptimizer with the PBE0/6-31+G(d,p)/PCM(ϵ=11.0) wavefunction to calculate the intramolecular energy penalty and distributed multipoles, and the FIT potential with Scheraga's S and O parameters for the dispersion-repulsion interactions, but iterated to convergence (which was achieved in all cases but one).
The free energy corrections were calculated for a rigid-molecule using the harmonic approximation on the same intermolecular potential energy surface, using the codes of Nyman and Day to produce the supercells to converge the Brillouin zone."

* 8. Computational time taken to generate the attached results (CPU hours)

70193

* 9. Outline of computational hardware utilised.

Clusters of various mixed nodes e.g. dual Xeon E5-2650v3, all more than 5 years old.

10. Supporting statement(s)

10 of 10 answered

"In a salt, there will be considerable polarization of the charge density of the ions within the crystal, which is not readily modelled within the Ψmol methodology.
Calculating the intramolecular energy penalty and distributed multipoles in a polarizable continuum approximates the distortion of the change density by surrounding molecules in a manner that is independent of the crystal structure.
The final lattice energy range of 116 kJ mol-1 and intramolecular energy range of 50 kJ mol-1 seem quite large, but we are submitting a set of plausible crystal structures with diverse hydrogen bonding and conformations.
Because of the omission of crystal specific polarization, we have low confidence in this submission."

DONE

Powered by
SurveyMonkey®
See how easy it is to create a survey.

Privacy & Cookie Notice

10 of 10 answered
