# Supplementary material for: The seventh blind test of crystal structure prediction: structure ranking methods
Source: Acta Crystallogr B Struct Sci Cryst Eng Mater. 2024 Oct 17;80(Pt 6):548–74. doi: 10.1107/S2052520624008679 (PMC11789160; doi:10.1107/S2052520624008679)
Supplement: Supplementary file 3 [file b-80-00548-sup3.zip › SI-C_theoretical_structures_CCDC_lists_and_predictions/ranked_structure_sets/Group24_SLPrice/XXXIII-2-SLPrice_CSPBT7/XXXIII-2-SLPrice_CSPBT7_Duplicates.docx]

Duplicate structures - XXXIII-2-SLPrice_CSPBT7

| Retained structure | Discarded structure(s) |
| --- | --- |
| XXVIII_structure_210.cif | XXVIII_structure_388.cif |
| XXVIII_structure_479.cif | XXVIII_structure_340.cif |
| XXVIII_structure_82.cif | XXVIII_structure_129.cif  XXVIII_structure_438.cif |
| XXVIII_structure_172.cif | XXVIII_structure_102.cif |
| XXVIII_structure_377.cif | XXVIII_structure_222.cif |
| XXVIII_structure_426.cif | XXVIII_structure_415.cif |
| XXVIII_structure_40.cif | XXVIII_structure_15.cif |
| XXVIII_structure_169.cif | XXVIII_structure_306.cif |
| XXVIII_structure_123.cif | XXVIII_structure_69.cif |
| XXVIII_structure_127.cif | XXVIII_structure_367.cif |

Output from Clustering script

structure CCDC388	matches structure CCDC210	with RMSD	30 = 0.17778500000000000

structure CCDC340	matches structure CCDC479	with RMSD	30 =	4.0512300000000001E-002

structure CCDC129	matches structure CCDC82	with RMSD	30 = 0.19587599999999999

structure CCDC102	matches structure CCDC172	with RMSD	30 =	3.6972800000000000E-002

structure CCDC438	matches structure CCDC82	with RMSD	30 =	7.7003299999999997E-002

structure CCDC222	matches structure CCDC377	with RMSD	30 = 0.15904699999999999

structure CCDC415	matches structure CCDC426	with RMSD	30 = 0.15821399999999999

structure CCDC15	matches structure CCDC40	with RMSD	30 = 0.17414600000000000

structure CCDC306	matches structure CCDC169	with RMSD	30 =	8.8013999999999995E-002

structure CCDC69	matches structure CCDC123	with RMSD	30 =	6.0388699999999997E-002

structure CCDC367	matches structure CCDC127	with RMSD	30 =	9.9902199999999997E-002
