# Supplementary material for: The seventh blind test of crystal structure prediction: structure ranking methods
Source: Acta Crystallogr B Struct Sci Cryst Eng Mater. 2024 Oct 17;80(Pt 6):548–74. doi: 10.1107/S2052520624008679 (PMC11789160; doi:10.1107/S2052520624008679)
Supplement: Supplementary file 3 [file b-80-00548-sup3.zip › SI-C_theoretical_structures_CCDC_lists_and_predictions/ranked_structure_sets/Group5_GDay/XXVII-2-GDay_CSPBT7_2/XXVII-2-GDay_equivalent_structures.docx]

Clustering Results for optimised XXVII-2 Structures using COMPACK.*

| **Unique ID** | **Equivalent ID** |
| --- | --- |
| XXVII_structure_61 | XXVII_structure_28 |
|  |  |

*Structures assigned equivalent if calculated to have an RMSD below 0.30 Å with 30/30 molecules matching. The lower energy structure was retained.
