# Supplementary material for: The seventh blind test of crystal structure prediction: structure ranking methods
Source: Acta Crystallogr B Struct Sci Cryst Eng Mater. 2024 Oct 17;80(Pt 6):548–74. doi: 10.1107/S2052520624008679 (PMC11789160; doi:10.1107/S2052520624008679)
Supplement: Supplementary file 3 [file b-80-00548-sup3.zip › SI-C_theoretical_structures_CCDC_lists_and_predictions/ranked_structure_sets/Group5_GDay/XXXII-2-GDay_CSPBT7_2/XXXII-2-GDay_CSPBT7/XXXII-2-GDay_equivalent_structures.pdf]

The following structures (indexed by original number as provided by the CCDC) were found to be matches according to the COMPACK comparison* as implemented in the CCDC's CSD Python API.

In all cases, the lower energy structure (after our ranking procedure) was retained.

**Unique ID (kept)**	**Equivalent ID (removed)**
XXXII_105_final_ccdc.cif	XXXII_485_final_ccdc.cif
XXXII_169_final_ccdc.cif	XXXII_488_final_ccdc.cif

*Structures were decided to be equivalent if calculated to have an RMSD below 0.30 Å with 30/30 molecules matching, using distance and angle tolerances of 0.2 Å and 30 degrees.
