# Supplementary material for: The seventh blind test of crystal structure prediction: structure ranking methods
Source: Acta Crystallogr B Struct Sci Cryst Eng Mater. 2024 Oct 17;80(Pt 6):548–74. doi: 10.1107/S2052520624008679 (PMC11789160; doi:10.1107/S2052520624008679)
Supplement: Supplementary file 3 [file b-80-00548-sup3.zip › SI-C_theoretical_structures_CCDC_lists_and_predictions/ranked_structure_sets/Group7_MTuckerman/XXXI-2-NGalanakis_1/XXXI-2-NGalanakis/NGalanakis_CSPBT7_report.pdf]

Target XXXI: Second Submission Report

Galanakis, Nikolaos[1]* & Tuckerman, Mark E.[1,2,3]*

* Correspondence to: ng1807@nyu.edu, mark.tuckerman@nyu.edu

[1] Department of Chemistry, New York University,
New York, NY 10003 USA.

[2] Courant Institute of Mathematical Sciences,
New York University, New York, NY 10012 USA.

[3] NYU-ECNU Center for Computational Chemistry at NYU Shanghai,
3663 Zhongshan Road North, Shanghai, 200062, China.

Monday, June 6th, 2022

Contents

1 Topological measures of crystal structure stability **3**

2 Target XXXI structures ranking **8**

3 Resources **8**

1 Topological measures of crystal structure stability

The ranking of the structures is based on a new topological CSP approach, based on the mathematical principle that in a stable molecular crystal, the atomic positions of highly charged atoms (with partial charge $|q| > 0.1$) correspond to the zeroes of a set of geometric functions Ξ_k. For a unit cell containing Z molecules, each having M atoms, consider the crystallographic coordinates $\mathbf{r}_1^{(i)}$ of atom (i) in the reference molecule. Let $\mathbf{r}_m^{(i)} = \mathbf{G}_j \mathbf{r}_m^{(i)}$, $m = 2, \ldots, M$ be it's $Z-1$ symmetry-related atomic positions in the unit cell, where $\mathbf{G}_j$, $j = 1, ..., Z$ is a set of space-group generators for the crystal. This scheme, proposed that the positions of certain atoms in the unit cell, namely the atoms with high partial charges, can be calculated by solving the set of equations

$$\Xi_k(\mathbf{G}_1\mathbf{r}_1^{(i)} - \mathbf{r}_p^{(i)}, ..., \mathbf{G}_Z\mathbf{r}_1^{(i)} - \mathbf{r}_p^{(i)}) = 0 \tag{1}$$

where $\mathbf{r}_p^{(i)}$ is the average of the Z positions $\mathbf{r}_m^{(i)}$. The choice of the functions Ξ_k is very important and although various choices might be suitable, we have selected the so-called Zernike parameters $Z_{n\ell m}$ defined as

$$Z_{n\ell m}(\mathbf{r}_1^{(i)}, ..., \mathbf{r}_Z^{(i)}) = \left[\frac{3n+1}{4\pi Z}\sum_{j=1}^{Z} R_{n\ell}(r_j(i))Y_{\ell,m}(\theta_j(i), \phi_j(i))\right]^2 \tag{2}$$

The solutions of these equations are crystallographic planes in the general form

$$\epsilon_z : A_{\text{zzp}}u + B_{\text{zzp}}v + C_{\text{zzp}}w = 0.25k_{\text{zzp}}, \tag{3}$$

where $A_{\text{zzp}}, B_{\text{zzp}}, C_{\text{zzp}} \in [-1, 0, 1]$, $A_{\text{zzp}} \times B_{\text{zzp}} \times C_{\text{zzp}} = 0$ and $k_{\text{zzp}} \in [0, \pm1, \pm2, \pm3, \pm4]$. The possible combinations of $A_{\text{zzp}}, B_{\text{zzp}}, C_{\text{zzp}}$ generate 9 possible crystallographic planes that are parallel to the faces of the unit cell (i.e. $u = 0.25k_{\text{zzp}}$) or along the diagonals (i.e. $u + w = 0.25k_{\text{zzp}}$).

Additional topological considerations are introduced based on the statistical analysis of the organic molecular structures included in the Crystallography Open Database (COD) and the Cambridge Structural Database (CSD). These considerations involve (1) The orientation of the molecules in terms of the eigenvector of the inertia tensor, (2) the orientation of the rings in terms of their vector normal to the average ring plane, (3) the strength of the close contacts between atoms of neighbouring molecules (atoms at distance $r_{ij} < R_{\text{vdW}}^{(i)} + R_{\text{vdW}}^{(j)}$), (4) the partial charges of atoms that are found at distance $0.25k_{\text{zzp}}$ along one of the 9 possible crystallographic planes ϵ_z.

For the ranking of the target XXXI structures, we analyzed 1075 structures in the Crystallography Open Database (COD) composed of C, H, F, N, O and S atoms. The analysis revealed a strong connection between the orientation of the principal axes of inertia and the normal ring plane vectors to the cell geometry. In detail, for each structure, the eigenvectors $\hat{e}_i$, $i = 1, 2, 3$ of the inertia tensor as well as the normal vector $\hat{k}_c$ representing the average ring plane are almost perpendicular to the crystallographic directions $\mathbf{n} = (n_u, n_v, n_w)$ with components restricted to $0, \pm1, \ldots, \pm n_{\max}$, $n_u|n_v|n_w = n_{\max}$ and $n_u n_v n_w = 0$. Figures 1-2 show respectively the distributions of the minimum angle $\omega_{\min}$ formed by the set of vectors $\mathbf{n}$ and the inertia tensor eigenvectors/normal ring plane vectors, for $n_{\max} = 1, 2, 3, 4, 5$. In each figure, the 95% confidence interval is noted. Additional lower confidence intervals were also generated to examine the proximity of each proposed structure to the peaks observed in the database. The likelihood of a structure is determined by the number of angles $\omega_{\min}$ that are found within the confidence intervals and the average deviation of the angles $\omega_{\min}$ from the 90°.

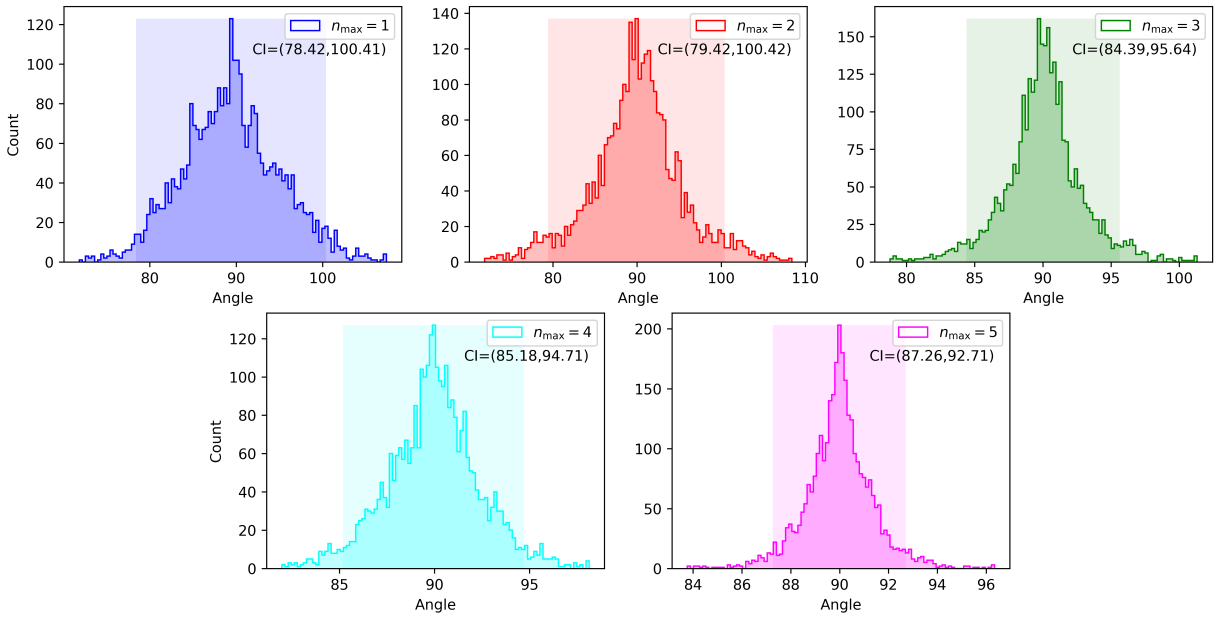

Figure 1: Distributions of the minimum angle formed by the principal axes of inertia to the crystallographic directions $\mathbf{n} = (n_u, n_v, n_w)$ with components restricted to $0, \pm 1, \ldots, \pm n_{\max}$, $n_u|n_v|n_w = n_{\max}$ and $n_u n_v n_w = 0$ for all the structures in the COD database composed of C, H, F, N, O and S atoms.

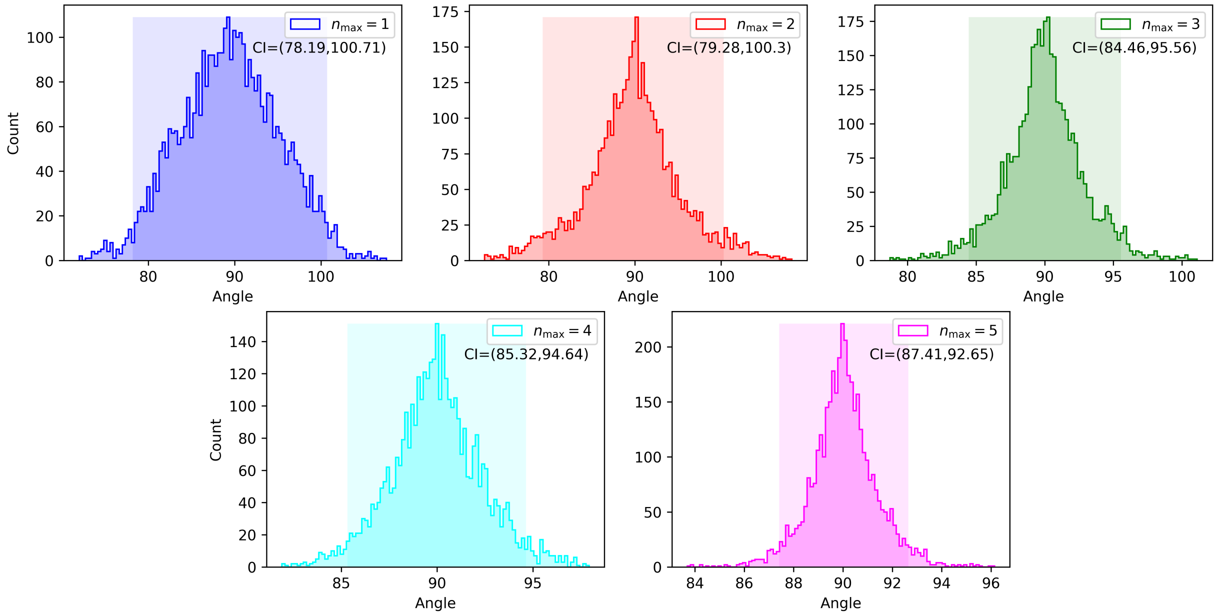

Figure 2: Distributions of the minimum angle formed by the normal ring plane vectors to the crystallographic directions $\mathbf{n} = (n_u, n_v, n_w)$ with components restricted to $0, \pm 1, \ldots, \pm n_{\max}$, $n_u|n_v|n_w = n_{\max}$ and $n_u n_v n_w = 0$ for all the structures in the COD database composed of C, H, F, N, O and S atoms.

The strength of the close contact depends of the atomic species forming the contact. In general, the strength d_s varies in the range $[0, d_s^{\max})$. In Fig. 3-4 we show the distributions of the vdW short contact strength for all the possible atomic pairs of the analyzed structures. It is clear that the strength of the close contact also depends on the existence of a hydrogen bond or not. We separate the close contacts into three categories: (1) vdW short contacts in which case none of the atoms is part of a hydrogen bond, (2) Mixed, formed by one atom that is part of a H-Bond and one atom that is not part of a H-Bond and (3) H-Bonds, in which the short contact is formed by two atoms directly related to a hydrogen bond. In the case of the target XXXI structures, all the close contacts are of vdW type. These probability allow us to determine the probability for each target XXXI candidate structure to form the set of close contacts that are specific to the structure.

In terms of the atomic separations in the reference molecule it is found that for 98.21% of the compound in the COD database, there is at least one atomic pair along each direction ϵ_z, formed by an atom with high partial charge and another atom of the molecule, so that the distance of the two atoms along the direction ϵ_z is equal to $0.25k_{\text{zzp}}$. In Fig. 5 we show the distribution of the charges for each possible pair that are used to determine the likelihood of each structure in terms of the intramolecular crystallographic atomic separations.

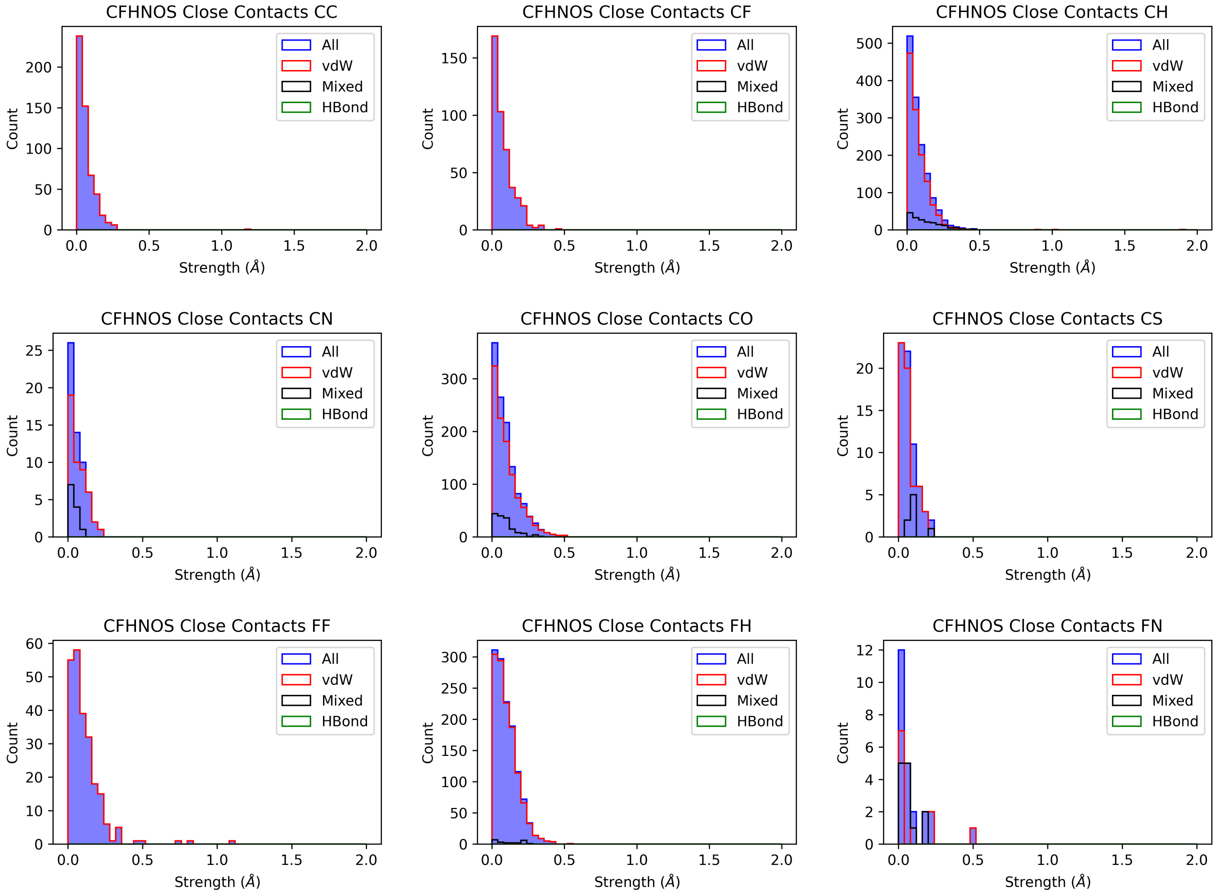

Figure 3: Distribution of the short contact strength (Part 1) for all the structures in the COD database composed of C, H, F, N, O and S atoms and for the different short contact types.

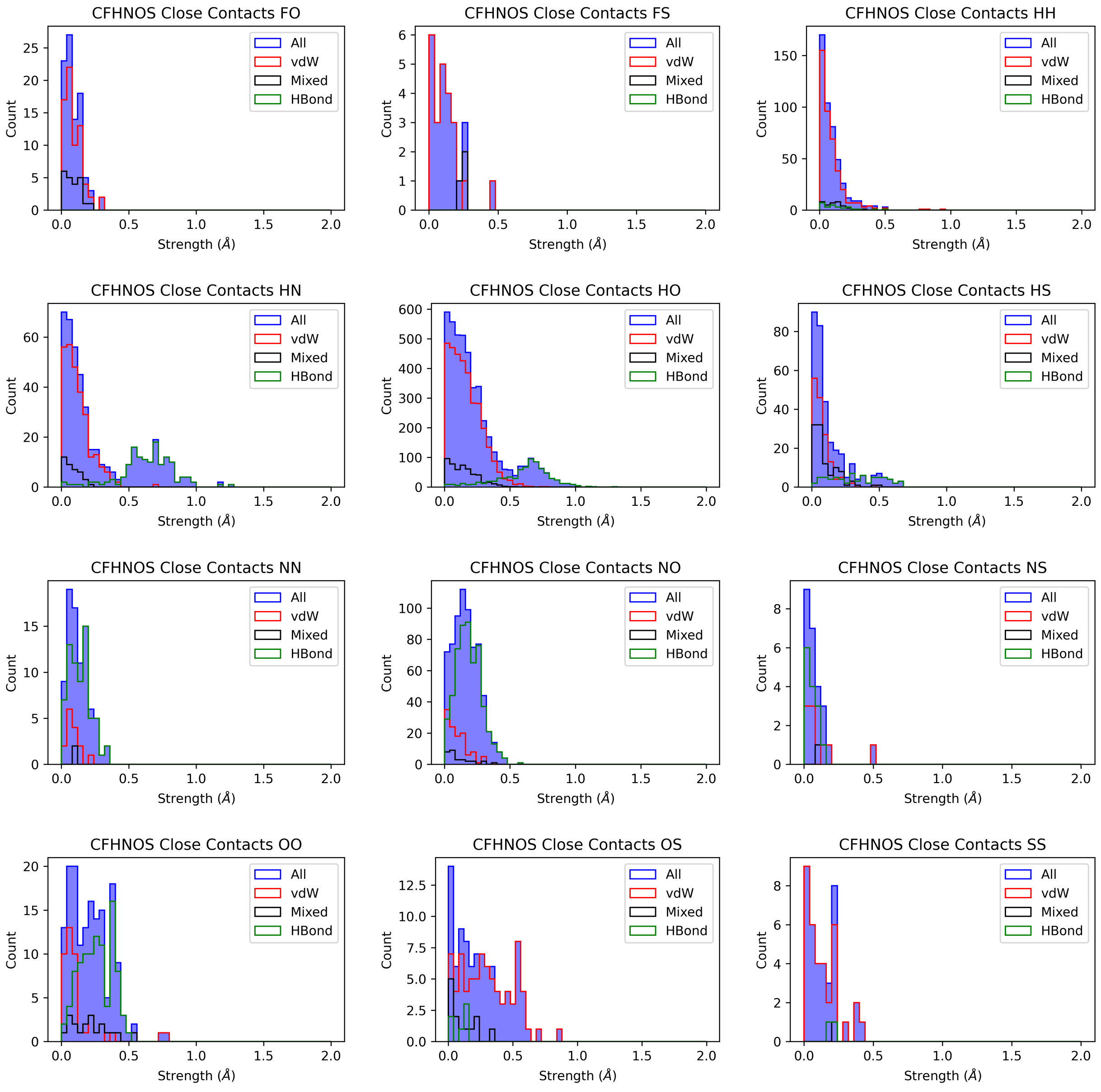

Figure 4: Distribution of the short contact strength (Part 2) for all the structures in the COD database composed of C, H, F, N, O and S atoms and for the different short contact types.

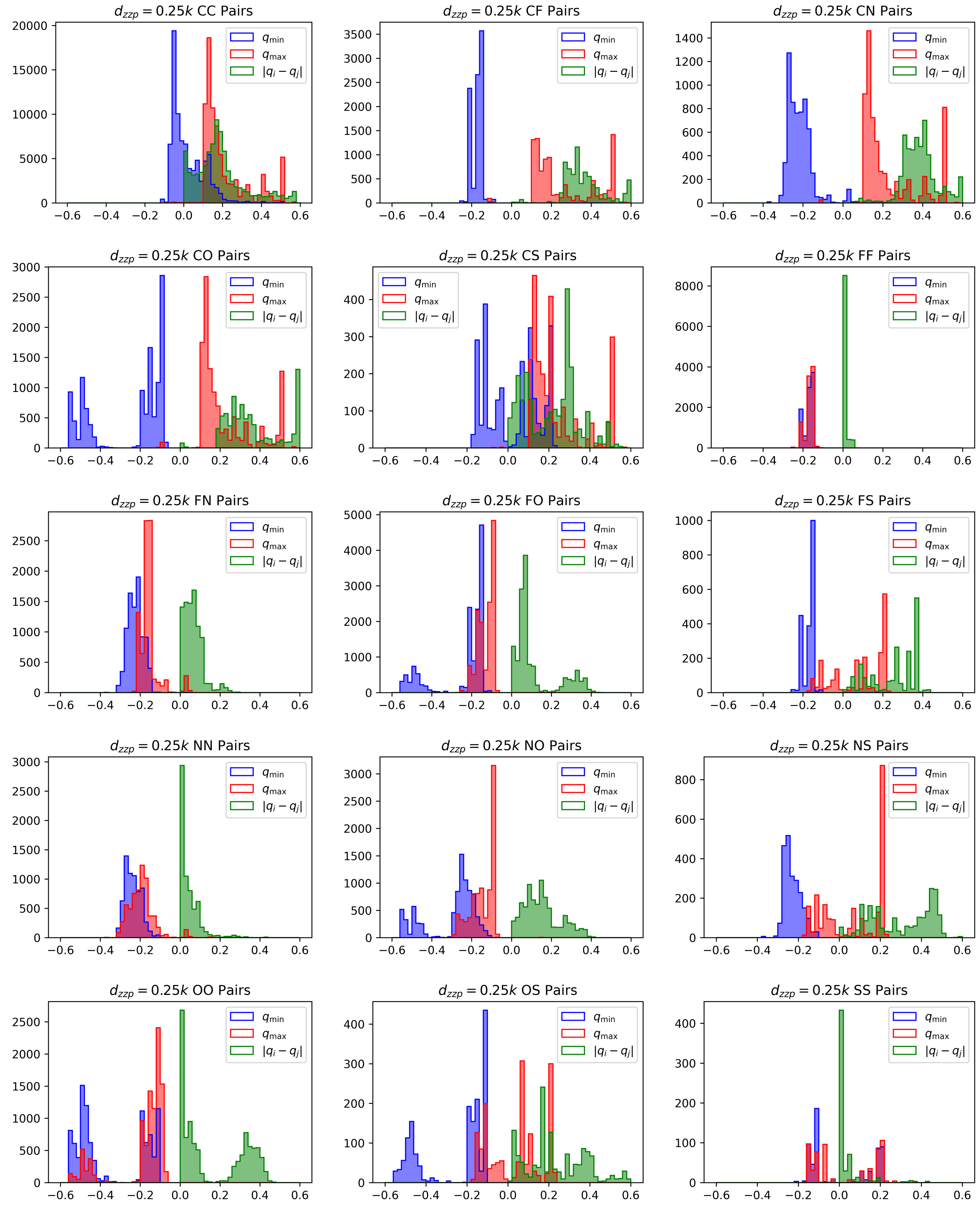

Figure 5: Distribution of the partial charges of the atoms forming pairs at distance $0.25k$ in crystallographic coordinates along the 9 possible ZZP directions.

2 Target XXXI structures ranking

The partial charges for the Target XXXI compound were calculated via the CSD Python API. The method used is that of Gasteiger J.; Marsili M., Tetrahedron 36 (22) 3219-3228 1980. The process identified 10 high-charge atoms in the structure, labeled as C_2, C_5, C_6, C_{12}, O_1, O_2, O_3, F_1, F_2 and F_3. The ranking process involves 7 steps:

1. Discarding structures for which $n_{\mathrm{C},0.01} = 0$ or $n_{\mathrm{O},0.01} = 0$, $n_{\mathrm{F},0.01} = 0$, where $n_{\mathrm{C},0.01}$, $n_{\mathrm{C},0.01}$, $n_{\mathrm{C},0.01}$ is the number of high-charge C, O and F atoms that are found at distance $d \leq 0.01$ (in crystallographic coordinates) to the nearest plane ϵ_z.

2. Discarding structures for which $n_{\mathrm{C},0.025} < n_{\mathrm{C}}$ or $n_{\mathrm{O},0.025} < n_{\mathrm{O}}$, $n_{\mathrm{F},0.025} < n_{\mathrm{F}}$, where $n_{\mathrm{C},0.025}$, $n_{\mathrm{C},0.025}$, $n_{\mathrm{C},0.025}$ is the number of high-charge C, O and F atoms that are found at distance $d \leq 0.025$ (in crystallographic coordinates) to the nearest plane ϵ_z and n_{C}, n_{O}, n_{F} is the total number of high-charge C, O, and F atoms (4, 3 and 3 respectively).

3. Discarding structures for which more than one of the angles ω_{min} formed by the eigenvectors of the inertia tensor $\hat{e}_i$ and the crystallographic directions $\mathbf{n}$ lies outside the 95% confidence intervals.

4. Discarding structures for which more than one of the angles ω_{min} formed by the normal vectors $\hat{k}_c$ of the rings and the crystallographic directions $\mathbf{n}$ lies outside the 95% confidence intervals.

5. Discarding structures for which more than 50% of the angles ω_{min} formed by the eigenvectors of the inertia tensor $\hat{e}_i$ and the crystallographic directions $\mathbf{n}$ lies outside the 50% confidence intervals.

6. Discarding structures for which more than 50% of the angles ω_{min} formed by the normal vectors $\hat{k}_c$ of the rings and the crystallographic directions $\mathbf{n}$ lies outside the 50% confidence intervals.

7. Rank the structure by calculating the combined probability:

$$P = 0.25(P_{cc} + P_{d_{\mathrm{zzp}}} + P_{\hat{e}_i} + P_{\hat{k}_c}), \tag{4}$$

   where

   - P_{cc} is the probability of a structure to form the specific set of close contacts.
   - P_{cc} is the probability of a structure to have the specific set of atomic pairs at distance $0.25k_{\mathrm{zzp}}$.
   - $P_{\hat{e}_i}$ is the probability of a structure to have the angles ω_{min} formed by the eigenvectors of the inertia tensor $\hat{e}_i$ and the crystallographic directions $\mathbf{n}$ within the confidence intervals.
   - $P_{\hat{k}_c}$ is the probability of a structure to have the angles ω_{min} formed by the normal vectors $\hat{k}_c$ of the rings and the crystallographic directions $\mathbf{n}$ within the confidence intervals.

In table 1 we list the 25 most probable structures according to the aforementioned criteria.

3 Resources

All the calculations for the ranking protocol required 10 hours of CPU usage in a mid-range laptop (Intel Core i7-10750H CPU @ 2.60GHz, 16.0 GB RAM).

Table 1: List of the 25 most probable structures ranked in order of P.

**Structure**	**Space group**	P	P_{cc}	$P_{d_{\mathrm{zzp}}}$	$P_{\hat{e}_i}$	$P_{\hat{k}_c}$
XXXI_structure_24	P21/c	0.6042	0.1045	0.3124	1.0000	1.0000
XXXI_structure_5	P21/c	0.6017	0.0949	0.3120	1.0000	1.0000
XXXI_structure_29	P21/c	0.5993	0.1018	0.2953	1.0000	1.0000
XXXI_structure_38	P21/c	0.5981	0.1022	0.2901	1.0000	1.0000
XXXI_structure_59	P21/c	0.5978	0.1010	0.2901	1.0000	1.0000
XXXI_structure_79	P21/c	0.5960	0.0823	0.3018	1.0000	1.0000
XXXI_structure_19	P21/c	0.5954	0.0810	0.3006	1.0000	1.0000
XXXI_structure_56	P21/c	0.5952	0.0904	0.2903	1.0000	1.0000
XXXI_structure_31	P21/c	0.5938	0.0943	0.2808	1.0000	1.0000
XXXI_structure_70	P21/c	0.5936	0.0756	0.2990	1.0000	1.0000
XXXI_structure_67	P21/c	0.5936	0.0764	0.2980	1.0000	1.0000
XXXI_structure_35	P212121	0.5934	0.1128	0.2607	1.0000	1.0000
XXXI_structure_42	P-1	0.5932	0.1378	0.2350	1.0000	1.0000
XXXI_structure_86	P21/c	0.5925	0.1005	0.2694	1.0000	1.0000
XXXI_structure_96	P21/c	0.5910	0.0725	0.2915	1.0000	1.0000
XXXI_structure_33	P21/c	0.5908	0.0711	0.2922	1.0000	1.0000
XXXI_structure_45	P21/c	0.5895	0.0776	0.2803	1.0000	1.0000
XXXI_structure_7	C2/c	0.5886	0.0820	0.2726	1.0000	1.0000
XXXI_structure_97	P21/n	0.5866	0.1053	0.3077	0.9333	1.0000
XXXI_structure_60	P21/c	0.5862	0.0725	0.2721	1.0000	1.0000
XXXI_structure_84	P212121	0.5859	0.0955	0.2482	1.0000	1.0000
XXXI_structure_58	C2/c	0.5857	0.0834	0.2596	1.0000	1.0000
XXXI_structure_57	P21/c	0.5855	0.0893	0.3194	0.9333	1.0000
XXXI_structure_46	P21	0.5837	0.0810	0.2538	1.0000	1.0000
XXXI_structure_22	P21/c	0.5828	0.0790	0.3187	0.9333	1.0000
